# Strong functional patterns in the evolution of eukaryotic genomes revealed by the reconstruction of ancestral protein domain repertoires

**DOI:** 10.1186/gb-2011-12-1-r4

**Published:** 2011-01-17

**Authors:** Christian M Zmasek, Adam Godzik

**Affiliations:** 1 Program in Bioinformatics and Systems Biology, Sanford-Burnham Medical Research Institute, 10901 North Torrey Pines Road, La Jolla, CA 92037, USA

## Abstract

**Background:**

Genome size and complexity, as measured by the number of genes or protein domains, is remarkably similar in most extant eukaryotes and generally exhibits no correlation with their morphological complexity. Underlying trends in the evolution of the functional content and capabilities of different eukaryotic genomes might be hidden by simultaneous gains and losses of genes.

**Results:**

We reconstructed the domain repertoires of putative ancestral species at major divergence points, including the last eukaryotic common ancestor (LECA). We show that, surprisingly, during eukaryotic evolution domain losses in general outnumber domain gains. Only at the base of the animal and the vertebrate sub-trees do domain gains outnumber domain losses. The observed gain/loss balance has a distinct functional bias, most strikingly seen during animal evolution, where most of the gains represent domains involved in regulation and most of the losses represent domains with metabolic functions. This trend is so consistent that clustering of genomes according to their functional profiles results in an organization similar to the tree of life. Furthermore, our results indicate that metabolic functions lost during animal evolution are likely being replaced by the metabolic capabilities of symbiotic organisms such as gut microbes.

**Conclusions:**

While protein domain gains and losses are common throughout eukaryote evolution, losses oftentimes outweigh gains and lead to significant differences in functional profiles. Results presented here provide additional arguments for a complex last eukaryotic common ancestor, but also show a general trend of losses in metabolic capabilities and gain in regulatory complexity during the rise of animals.

## Background

Eukaryotic organisms exhibit an enormous diversity on many different levels [1]. Besides vast variance in size, appearance, ecology, and behavior, they also display massive variation in their morphological and behavioral complexity, ranging from unicellular protists to basal animals, such as *Trichoplax adhaerens *with no internal organs and only four different cell types [2] to mammals with multiple internal organs, a complex nervous system, and around 210 different cell types [3,4]. Yet, the number of protein coding genes present in eukaryotic genomes remains remarkably constant and does not appear to correlate with perceived morphological and behavioral complexity. For example, the human genome is estimated to be composed of around 20,500 protein coding genes [5], whereas the simple roundworm *Caenorhabditis elegans *possesses about 19,000 protein coding genes [6], and the morphologically more complex fruit fly *Drosophila melanogaster *has a genome of only about 14,000 genes [7]. In order to explain this so called 'gene-number paradox' [8], numerous hypotheses have been put forward. For instance, dramatic differences in morphological complexity, given relatively similar numbers of protein coding genes, have been explained with an increasing role of non-coding RNA transcription (for example, [8,9]), alternative splicing [10], transposable elements [11], detailed transcriptional control enabling a tight temporal and spatial control of gene expression [12], the complexity of domain organization of proteins [13,14], and expansion of select gene families [15,16].

While biologists have long been enthralled by the vast diversity found amongst modern eukaryotes, the underlying evolutionary history that led to this vast diversity is at least equally fascinating and is likely to help our understanding of extant organisms and their molecular biology. An intuitive view of eukaryote evolution is that the last eukaryotic common ancestor (LECA) was 'simple' and that accretion of features over time led to complex, multicellular organisms, such as plants and animals. Recently, an increasing number of studies are surfacing that suggest that many aspects of the LECA might not have been 'simple' and that it probably already had many features commonly associated with modern eukaryotes [17]. For example, recent work suggests that the LECA already had an endomembrane system with near modern complexity (reviewed in [18]), as well as a complex cell division machinery [19]. Numerous studies show that the LECA also had a relatively large number of genes and that gene loss is a likely a significant contributor to the composition of modern genomes [16,20-22].

A succinct way to describe the functional potential of large groups of genes, such as complete genomes or metagenomes, is to list and analyze the set of recognized domains present in proteins encoded by the genes in a given group. Recently, a term 'domainome' was proposed for such sets [23]. Protein domains are minimal structural and evolutionary units in proteins, retaining their structure and usually their function even when being part of proteins with different domain architectures [24]. Information about recognized protein domains is collected in public resources such as Pfam [25] or InterPro [26], which also provide information about functions of individual domains (if available), both in the form of short narratives as well as mappings into formalized functional classifications, such as the gene ontology (GO) [27].

In this work, we investigate the evolution of the domain repertoires of eukaryotic genomes. To gain a more complete picture of this evolution, we reconstruct the domainomes of ancestral species at important branching points of the eukaryotic tree of life, such as the LECA and the Urbilateria (the last common ancestor of protostome and deuterostome animals). While parts of putative genomes for relatively recent ancestral species have been reconstructed successfully (such as for the ancestor of placental mammals [28]; reviewed in [29]), due to vastly greater evolutionary distances and such effects as domain shuffling, we chose to reconstruct ancestral protein domain sets (domainomes) as opposed to complete sets of genes or entire genomes.

## Results

### Protein domain composition of extant and ancestral genomes

**Figure 1:**
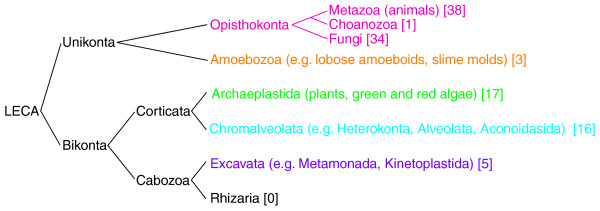
**An overview of a current model of eukaryote evolution **[30,67]. Numbers in brackets indicate the number of genomes from each branch analyzed in this work.

**Figure 2:**
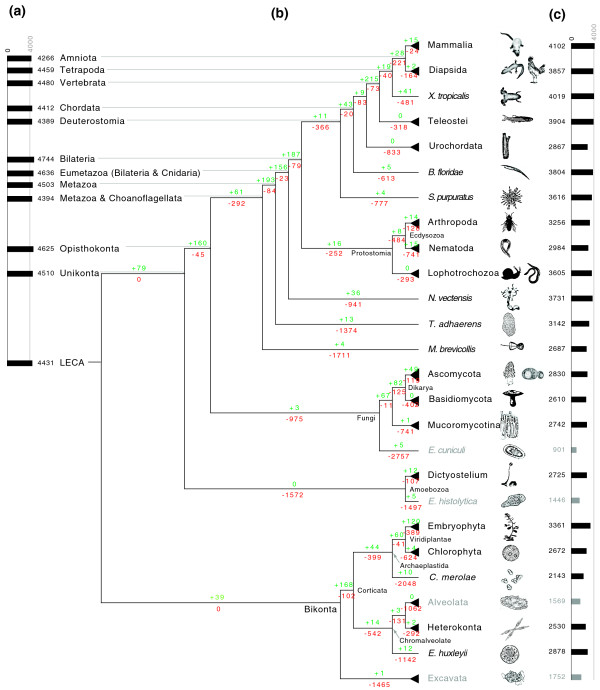
**Domain gains and losses during eukaryote evolution**. **(a) **Inferred domainome sizes for ancestral genomes on the path from the LECA to mammals are shown on the left. **(b) **The numbers of gained protein domains per branch (edge), inferred by Dollo parsimony, are shown in green, whereas inferred losses are shown in red. **(c) **The numbers of distinct domains per genome in extant species are shown on the right side; for groups of species represented as triangles, these numbers are averages. Species, or groups of species, that are mostly parasitic are shown in grey. For more detailed data see Additional files 3 and 4. This figure was made using 'gathering' cutoffs provided by Pfam; for a corresponding figure using a E-value cutoff of 10^-8^, see Additional file 13.

We analyzed complete sets of predicted proteins for 114 eukaryotic genomes, including 73 from opisthokonta (38 metazoa, 1 choanoflagellate, and 34 fungi), 3 from amoebozoa, 17 from archaeplastida, 16 from chromalveolata, and 5 from excavate, thus covering 5 of the 6 eukaryotic 'supergroups' [30,31] (we were unable to obtain any complete genomes for the 'supergroup' Rhizaria [32]), for the presence of protein domains, as defined by Pfam [25] (Figure 1; Additional file 1) The number of distinct protein domains varies from roughly 2,000 in the free living unicellular ciliate *Paramecium tetraurelia *to 3,140 in one of the simplest multicellular animals, *Trichoplax adhaerens*, to about 4,240 in humans (Figure 2c; for detailed counts see Additional files 2, 3, and 4). These numbers follow the expected trend of genomes of more complex organisms containing more domains; however, they include many apparent contradictions where more morphologically complex organisms contain fewer domains than less complex ones. To understand the evolutionary history of the observed domain distribution in extant species, we reconstructed the domain content of ancestral genomes, specifically those lying at internal nodes corresponding to major branching points in the evolution of eukaryotes. Since independent evolution of the same domain more than once is highly unlikely, we used Dollo parsimony, which, when applied to domain content, states that each domain can be gained only once, and seeks to minimize domain losses, to reconstruct the Pfam domain repertoire of ancestral eukaryotes [33-38] (Figure 2).

### The evolution of most eukaryotic groups is dominated by protein domain losses and not by domain gains

While the number of distinct domains found in extant species shows a weakly growing trend (with outliers) with the apparent morphological complexity (Figure 2c; for detailed counts see Additional file 2), comparing these numbers to those for the inferred ancestral genomes shows that the evolution of eukaryotes is defined by a balance between domain losses and gains, with the latter dominating at almost every branch of the tree of life (Figure 2b; Additional files 3 and 4). Unexpectedly, with a repertoire of about 4,400 distinct domains the LECA already had a large domain repertoire, that is, larger than any of the currently existing species. The two significant exceptions to this trend are the rise and early evolution of multicellular animals, roughly 650 to 500 million years ago, and the origin of vertebrates, around 450 million years ago losses (divergence time estimates are from [39]) - in these two cases domain gains significantly outnumber. Interestingly, the early evolution of the two major groups of bilaterians, the deuterostomes and protostomes are associated with a particularly high number in lost domains (about 366 losses and 11 gains for deuterostomes and 252 losses and 16 gains for protostomes).

### Less extensive domain losses in lophotrochozoans than in ecdysozoans

Our results show that some lineages went through a massive loss of domains. This phenomenon has been noticed previously for ecdysozoans in general, and for nematodes in particular [21,40-42]. In contrast, the other major group of protostomes, the lophotrochozoans, went through a less extreme gene loss when compared to last common ancestor of deuterostomes and protostomes (the Urbilateria). The domainome of the lophotrochozoan ancestor, reconstructed from the domainomes of three free living lophotrochozoans, two annelids (the polychaete worm *Capitella teleta *and the leech *Helobdella robusta*) and one mollusk (the snail *Lottia gigantea*) is larger than that of ecdysozoans, and the numbers of domains gained and lost relative to the Urbilateria are smaller (Table 1). This further confirms earlier speculation that lophotrochozoans are less derived from the Urbilateria than ecdysozoans [41].

**Table 1:** Protein domain gains and loss comparison between lophotrochozoans and ecdysozoans

	Ancestor domains			Extant domains		
		
	Gains	Losses	Present	Mean	Standard deviation	Genomes analyzed
**Lophotrochozoans**	16	545	4,215	3,605	320	3
Annelids	16	721	4,039	3,602	393	2
**Ecdysozoans**	24	736	4,032	3,202	190	12
Arthropods	38	864	3,918	3,256	172	9
Nematodes	39	1,477	3,306	3,039	143	3

In this table, gains and losses are relative to the last common ancestor of deuterostomes and protostomes, the Urbilateria. For the calculation of extant domain statistics, data from parasitic species is omitted (the nematode *Brugia malayi *and the flatworm *Schistosoma mansoni*).

### An unexpectedly large domainome in the sea anemone *Nematostella vectensis*

Another striking finding is the comparatively large domain repertoire of the cnidarian *Nematostella vectensis *(Starlet sea anemone) [43], especially relative to protostomes. Cnidarians are relatively simple in their morphology, having around 10 cell types [4], compared to protostomes, which are estimated to have between 30 and 50 distinct cell types [44]. This morphological simplicity of cnidarians clearly is not reflected in the genome content of *N. vectensis*, as its number of domains (approximately 3,700) is comparable to that of lophotrochozoans and surpasses all ecdysozoans analyzed here. This unexpected 'genomic' complexity (as opposed to morphological complexity) of *N. vectensis *(and likely other cnidarians as well) has also been noted on the level of regulatory networks (for example, in [45,46]). This is the best example illustrating a recurrent observation that the number of distinct protein domains is a poor predictor for morphological complexity.

### Functional consequences of domain gains and losses

As seen for the example of *Nematostella *and other outliers (Figure 2c; for detailed counts see Additional file 2), numbers of distinct domains do not correlate with complexity amongst eukaryotes. A likely explanation for this paradox may lie in the distribution of functions of domains, rather than in their numbers. To make inferences about the functional aspect of domain gains and losses, we defined functional profiles of domainomes by assigning individual domains with functions from the GO classification [27]. This allowed us to define a functional profile for each extant and inferred ancestral domainome, as well as for each set of gained and lost domains on every branch of the eukaryote tree of life (for details see the Materials and methods section). The first finding is that the functional profiles of sets of domains lost and gained at most branching points differ drastically: on the path leading from the LECA to mammals, domains with regulatory functions exhibit a net gain, while domains with metabolic functions show a net loss (Table 2). This effect is strongest for mammals and less pronounced for other metazoans. In contrast, for all other groups of eukaryotes, both regulatory domains and metabolic domains show a net loss, although with the net loss for regulatory domains being significantly smaller than that for metabolic domains. For instance, during flowering plant (Magnoliophyta) evolution, regulatory domains show an average, per branch, net loss of 5.6, and metabolic domains exhibit a net loss of 18.8. For mushrooms with complex fruiting bodies (homobasidiomycetes) [47], these values are 9.3 for net losses of regulatory domains, and 38.5 for net losses of metabolic domains.

**Table 2:** Functional differences in gained and lost domains

	Biological regulation		Metabolic process	
	
	Gains	Losses	Gains	Losses
LECA to mammals	12.0	5.2	8.1	21.6
LECA to plants	2.6	8.2	14.7	33.5
LECA to homobasidiomycetes	4.3	13.6	7.3	45.8

Average domain gain/loss counts per tree branch (edge) are shown.

**Figure 3:**
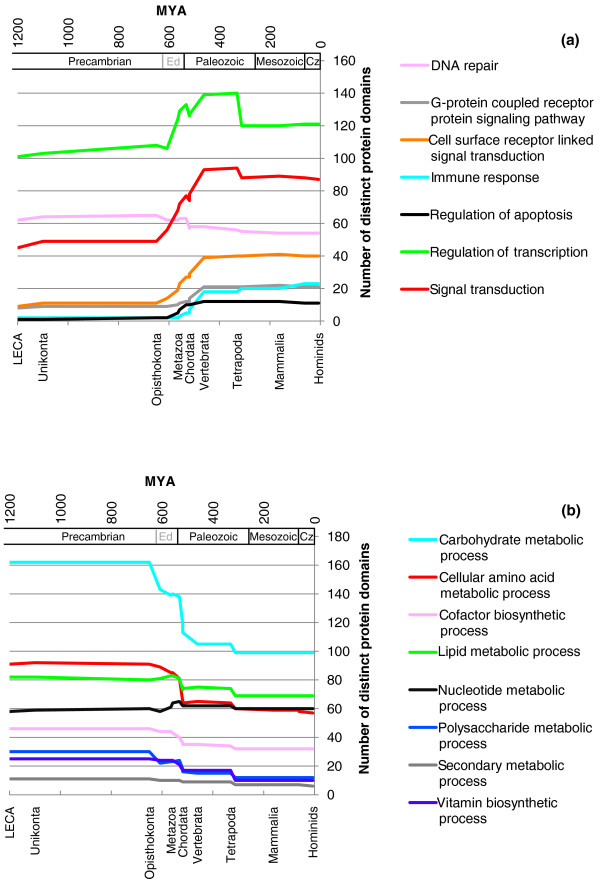
**Dynamics of genomes during animal evolution**. The functional contents of inferred ancestral genomes from the LECA to hominids (humans and great apes) are shown. **(a) **GO categories involved in various aspects of regulation. **(b) **GO categories involved in various aspects of metabolism (for detailed results see Additional files 5 and 6). Divergence time estimates are based on the fossil record and thus are minimum time constrains [39,68,69]. Geological periods are indicated on both panels ('Ed' stands for Ediacaran period and 'Cz' for Cenozoic era).

Applying GO term enrichment analysis, as commonly employed for microarray analysis [48], to the functions of lost and gained domains enabled us to obtain a more detailed view of the interplay between domain losses and gains (Tables 3 and 4). Within an overall increase in domains involved in regulation, our results show that animal evolution on a genome level is specifically associated with enrichment of protein domains involved in DNA-dependent transcriptional regulation, cell-matrix adhesion, apoptosis (programmed cell death), signal transduction (for example, G-protein coupled receptor protein signaling, mitogen-activated protein kinase kinase (MAPKK) activity), and various aspects of immune system functions (in particular cytokine and major histocompatibility complex-related domains). While most of the enriched categories can be classified as 'regulatory', some 'metabolic' categories are also enriched. In particular, a number of domains involved in mitochondrial electron transport appeared at the root of the bilaterian tree, and domains involved in lipid catabolic process appeared during the evolution of the first chordates. On the other hand, domain losses during animal evolution are predominantly associated with amino acid biosynthesis and carbohydrate metabolism. The only exception to this trend is an unexpected loss of numerous domains with functions in DNA-dependent transcriptional regulation during the evolution of the amniote ancestor. Figure 3 shows the effects of these gains and losses on the composition of the ancestral genomes during animal evolution (for lists of individual domains and their corresponding GO terms, see Additional files 5 and 6). The most drastic changes occurred around the rise of the first animals, whereas after the appearance of the first tetrapods, changes on the functional level of the genome are minimal. Most categories involved in regulation show an increase over time, with most of the effect seen during the rise of the first animals, followed by a more gradual increase. In contrast, categories involved in metabolism almost show a mirror image, an accelerated loss during the evolution of the first animals. The most drastic losses are in carbohydrate and amino acid metabolism. As expected, vitamin and cofactor biosynthesis also show significant losses. The only metabolic category that remains unchanged is nucleotide metabolism.

**Table 3:** Enriched gained and lost Gene Ontology terms along path from Unikonta to Mammalia

	Enriched gained GO terms	*P*-value	Enriched lost GO terms	*P*-value
**Unikonta**	Protein import into peroxisome matrix, docking	9.5E-3		
	*cAMP catabolic process*	1.9E-2		
	Organelle organization*	2.6E-2		
**Opisthokonta**	**Regulation of primary metabolic process**	1.3E-2	Protein-heme linkage	5.2E-3
			*Asparagine biosynthetic process*	1.0E-2
**Holozoa **(Metazoa and Choanoflagellata)	**Cell-cell signaling**	2.2E-3	*Xylan catabolic process*	1.6E-5
	**Cell surface receptor linked signal transduction**	9.2E-3	*Carbohydrate metabolic process*	3.1E-4
**Metazoa**	**Regulation of transcription, DNA-dependent**	1.2E-7	*Aromatic amino acid family biosynthetic process, prephenate pathway*	1.1E-4
			*Histidine biosynthetic process*	2.3E-3
	Cell-matrix adhesion	4.0E-4	Monosaccharide metabolic process*	6.9E-3
**Eumetazoa **(Bilaterian	Apoptosis	3.1E-4	Protein folding	1.7E-3
and Cnidaria)	Peptide cross-linking	4.7E-4	Transcription initiation	3.8E-3
**Bilateria**	Mitochondrial electron transport, NADH to ubiquinone	8.3E-6	*Branched chain family amino acid biosynthetic process*	3.3E-4
			*Histidine biosynthetic process*	2.3E-3
	**Wnt receptor signaling pathway**	2.7E-4	Water-soluble vitamin biosynthetic process*	5.0E-3
**Deuterostomia**	Protein transport	8.2E-2	*Cellular amino acid biosynthetic process*	7.0E-4
			Phosphoenolpyruvate-dependent sugar phosphotransferase system	3.2E-3
**Chordata**	*Lipid catabolic process*	3.2E-3	*Proteolysis*	2.1E-2
	**Activation of MAPKK activity**	6.7E-3		
**Urochordata and Vertebrata**	Antigen processing and presentation	5.5E-3	*Folic acid and derivative metabolic process*	2.3E-3
	Protein amino acid phosphorylation	1.8E-2	*Oligosaccharide biosynthetic process*	3.0E-3
**Vertebrata**	Immune response	4.4E-11	DNA topological change	2.0E-3
	**G-protein coupled receptor protein signaling pathway**	1.6E-5	*Carbohydrate metabolic process*	3.1E-3
**Tetrapoda**	**Regulation of growth**	1.3E-2	Valyl-tRNA aminoacylation	4.3E-3
	Synaptic transmission	2.0E-2	Response to water	8.6E-3
**Amniota**	Immune response	1.8E-3	**Regulation of transcription, DNA-dependent**	9.2E-8
			*Riboflavin biosynthetic process*	1.0E-3
	Defense response	2.0E-3	Thiamin biosynthetic process*	1.8E-3
**Mammalia**	Hemopoiesis	2.8E-3	*Aromatic amino acid family biosynthetic process*	1.1E-2
	Reciprocal meiotic recombination	8.3E-3		

The two terms with the lowest *P*-values are shown (calculated by the Ontologizer 2.0 software [63] with the Topology-Elim algorithm [64]), with the exception of the four terms marked by an asterisk, due to the relevance of these terms for this work. Prototypical regulatory terms are in bold text, prototypical metabolic terms are in italics (Additional files 5 and 6 list all gained and lost domains together with their associated GO terms and Additional file 14 summarizes the results of using different parameters in Ontologizer 2.0 software).

**Table 4:** Enriched gained and lost Gene Ontology terms for select clades

	Enriched gained GO terms	*P*-value	Enriched lost GO terms	*P*-value
Corticata	*Cobalamin biosynthetic process*	3.2E-7	**Small GTPase mediated signal transduction**	7.9E-3
	Photosynthesis	2.3E-6	Lipid transport	1.1E-2
Archaeplastida	Photosynthesis	3.1E-25	*Carbohydrate metabolic process*	9.1E-4
	Glycyl-tRNA aminoacylation	1.8E-2	*Xylan catabolic process*	4.8E-3
Viridiplantae	Photosynthesis	2.7E-3	*Tryptophan catabolic process to kynurenine*	7.1E-3
	Protein import into mitochondrial outer membrane	7.1E-3	*Sulfur compound biosynthetic process*	1.3E-2
Protostomia	Sensory perception of smell	4.3E-3	Protein secretion by the type II secretion system	3.4E-3
			Cell adhesion	4.2E-3

The two terms with the lowest *P*-values are shown (calculated by the Ontologizer 2.0 software [63] with the Topology-Elim algorithm [64]). Prototypical regulatory terms are in bold text, prototypical metabolic terms are in italics (for detailed results see Additional files 5 and 6).

### Alternative topologies of eukaryotic tree of life

It is important to stress that all the calculations presented so far critically depend upon the exact topology of the eukaryote evolutionary tree used for the parsimony based inference of ancestral domainomes. Additional files 7, 8, 9, and 10 show the results for different models for the eukaryote tree, and are discussed below.

### Classifying eukaryotes by the functional profiles of their genomes reproduces the tree of life

**Figure 4:**
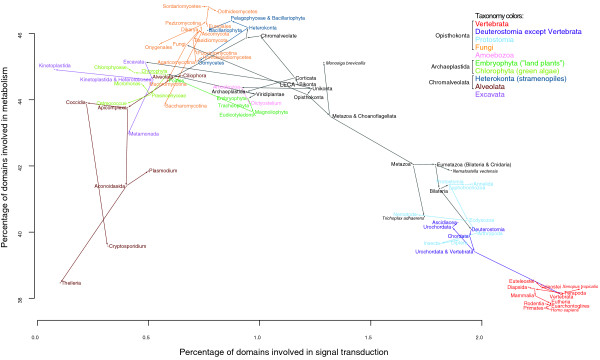
**Classifying eukaryotes by the functional profiles of their genomes**. A two-dimensional plot of regulatory function versus catalytic activity percentages for ancestral and extant domainomes.

Figure 4 shows a representation of the eukaryotic evolutionary tree in which the usual time and taxonomic axes are replaced by axes representing the percentage of domains involved in signal transduction and the percentage of domains with catalytic activity. Interestingly, this results in a graph clearly separating most major groups of eukaryotes. From this graph it is apparent that, on a functional level, vertebrate genomes (shown in red), as well as those of certain unicellular, chiefly parasitic, organisms, especially Kinetoplastida (for example, the sleeping sickness parasite *Trypanosoma brucei*) and Metamonada (for example, the Giardiasis agent *Giardia lamblia*) from the Excavata group [49] (shown in purple), and Aconoidasida (for example, the malaria parasite *Plasmodium falciparum*) from the Alveolata group (shown in brown) are the most derived relative to the LECA. On the other hand, this graph differs from the eukaryotic evolutionary tree in that some groups that are closely related appear quite distant, most strikingly seen in the large separation between fungi and animals, with fungi having the highest percentage in catalytic activity and animals having among the lowest. It is also noteworthy how similar all vertebrate genomes are to each other on this level, despite roughly 400 million years since the separation between ray-finned fish and tetrapods [39], especially compared to the big 'jumps' between vertebrates and the deuterstome ancestor and between the animal ancestor and the choanoflagellata/animal ancestor.

### Gut microbes complement human reduced metabolic capacity

One of the interesting questions one may ask is how the modern organisms compensate for the functionality of protein domains that were 'lost' compared to their ancestors, especially among basic metabolic functions. An intriguing possibility is that some of this functionality may be provided by symbiotic microbes. In a preliminary calculation we show that a 'meta-organism' containing a superset of protein domains found in the human genome and in the genomes of the two common gut commensals, *Bacteroides thetaiotaomicron *and *Eubacterium rectale*, very closely resembles the LECA in its profile of metabolic domains (Additional file 11). Interestingly, none of the known symbionts alone is able to provide such compensation, which agrees well with the observation that a 'minimal functional gut microbiome' consists of these two bacteria [50].

## Discussion

The results presented here indicate that although novel domains do appear throughout eukaryote evolution, this is offset, and usually overshadowed, by domain losses. The weak trend of the increase of the number of domains as a function of morphological complexity appears to be a consequence of larger losses for some of the morphologically simpler species. Overall, the number of distinct domains remains surprisingly constant and varies between 3,500 and 4,000 for most branches of the eukaryotic tree of life. It is important to remember that our estimates represents a lower bound for the domain repertoire for both the ancestral and extant genomes, since our analysis does not take into account extinct domains, domains not present or detected in any of the analyzed genomes nor as yet unidentified domains. Since the Pfam database does not yet cover the complete protein domain universe (especially so for domains specific to poorly studied organisms), at this point covering around 60% of most eukaryotic genomes, we expect the number of domain gains to grow with more complete versions of Pfam. However, we don't expect this would reverse our findings presented here. To test this, we compared the analysis presented here, which uses the current version of Pfam (24.0) with over 10,000 domain models, with results obtained with previous versions of Pfam. While the overall number of domains significantly increases with each release of Pfam, often by >20% with each release, overall tendencies are independent of the Pfam version used (for examples, see Additional file 12, which contains select data from an analysis using Pfam version 22.0).

### The minimal domain repertoire for a eukaryotic organism

The domain repertoires of the ciliates *Paramecium tetraurelia *and *Tetrahymena thermophila*, with about 2,080 and 2,190 distinct domains, respectively, while not the smallest of the genomes analyzed here, are the smallest of the free living organisms in this analysis, as all species with smaller domain sets are primarily parasitic (such as the cattle parasite *Theileria parva*, with of a domain repertoire size of only about 860). Interestingly, while the domain repertoire of *P. tetraurelia *is small, its gene number of around 40,000 is very high. It has been shown that the genome of *P. tetraurelia *is the result of at least three successive whole-genome duplications [51], explaining the low number of distinct domains in a large genome, containing, presumably, a high degree of redundancy. Similarly, *T. thermophila *also has a high gene count, around 27,000, yet this seems to be due to numerous small duplication events, as opposed to whole genome duplications [52]. It has also been found that *T. thermophila *shares more orthologous genes with humans than are shared between humans and the yeast *Saccharomyces cerevisiae *[52], despite fungi being phylogenetically closer to humans than ciliates - another finding supporting a genomically complex LECA and significant and lineage-specific loss of genes, and thus domains, during eukaryote evolution.

### Horizontal gene transfer

Horizontal gene transfer clearly has the potential to result in misleadingly inflated domain counts of ancestral species. Despite being more common in eukaryotes than previously thought, most known cases of horizontal gene transfer in eukaryotes involve bacteria as donors [53-55]. To avoid the possible effects of domains transferred from prokaryotes to eukaryotes, we performed the reconstruction analysis under exclusion of bacterial and archaeal genomes. Nevertheless, we cannot exclude the possibility that, especially for unicellular eukaryotes, a limited number of domains are present due to horizontal gene transfer. For this reason we focused most of our subsequent functional analyses on multicellular animals, since we are not aware of any reports showing gene transfer within animals.

### Effects of the model of eukaryote evolution

Clearly, domain content of ancestral genomes and the overall pattern of domain gains and losses are dependent on the details of the eukaryotic evolutionary tree used for the Dollo parsimony based reconstruction. There is an ongoing controversy concerning the details of the phylogenetic tree of eukaryotes (for example, [56]). In the results reported so far we have used a newly emerging paradigm according to which eukaryotes can be classified into two larger clades, the unikonts and the bikonts [57]. However, in order to assess the robustness of our results, we also performed all analyses with two alternative versions of the eukaryotic tree of life. The results for the alternative trees are presented in the additional material. The first one is a tree that follows the unikonta/bikonta deep split but differs in the animal sub-tree, where it follows the coelomata hypothesis instead of the more recent ecdysozoan hypothesis (see the 'coelomata' tree in Additional files 7 and 9) [58]. Interestingly, trees with an ecdysozoan clade consistently had a lower cost under Dollo parsimony than more traditional topologies (with a cost of 73,363 for a ecdysozoan model versus 74,433 for a coelomata model), adding further support to the ecdysozoan hypothesis. The second alternative tree, referred to in the following as 'crown group', differs more significantly, by essentially placing all protists outside of the plant/animal/fungal subtree (see Additional files 8 and 10). The domain gain and loss numbers based on the 'coelomata' tree do not show any significant differences from the results presented in the main text: the origins of deuterostomes and protostomes are still associated with large losses and lophotrochozoans appear less derived then arthropods and nematodes.

As expected, results based on the 'crown group' eukaryote tree appear to lead to strongly different domain counts for the LECA (1,825, as opposed to 4,431). However, this result is based primarily on a clade of Metamonda, namely *Giardia lamblia *and *Trichomonas vaginalis*, both human parasites, at the base of the tree. Clearly these two parasites are highly derived and unlikely to exhibit much resemblance to the LECA [59]. Moving from the LECA towards metazoans, the domain count for predicted ancestral species rapidly increases, and as a soon as a tree includes at least one free living species, the amoeba *Naegleria gruberi*, the domain count of the ancestral eukaryote (2,801) approaches the mean for extant nematodes (2,980). On the other hand, while the topology of the eukaryote tree of life used influences domain counts close to the root, it has no significant effect on the results concerning the functional dynamics of eukaryote genomes during evolution.

Finally, we would like to point out that the model shown in Figures 1 and 2 is controversial mainly due to uncertainty regarding the placement of Rhizaria. Since our analysis does not include any genomes from this group, this controversy has no bearing on the results presented here. The second controversy is regarding the placement of haptophytes (a phylum of algae), which in the model used here are considered part of Chromalveolata, but which according to recent results might form a clade with Archaeplastida [60]. In our analysis, haptophytes are represented by only one genome, *Emiliania huxleyi*, the placement of which on the tree of life has no measurable effect on the results presented here (data not shown).

### Further studies

Clearly, studies such as the one presented here will be more accurate and informative once more eukaryote genomes have been released covering the tree of life more uniformly, since there is currently still a bias towards commercially important species as well as traditional model organisms. For example, for animals, an increased coverage of lophotrochozoans would be desirable. Improved sampling over species space is also expected to go hand in hand with increased coverage of domain space by Pfam and similar databases.

## Conclusions

In this work we show that domain losses during eukaryote evolution are numerous and oftentimes outnumber domain gains. This, combined with estimates for large numbers of domains present in ancestral genomes, is an additional argument for a complex LECA. The functional profiles of gained and lost domains are very different; for instance, during animal evolution gained domains involved in regulatory functions are enriched, whereas lost domains are preferentially involved in metabolic functions, especially carbohydrate and amino acid metabolism. This makes it seem likely that animals over time outsourced a portion of their metabolic needs. Clustering inferred ancestral domainomes according to their functional profiles results in graphs remarkably similar to the eukaryotic tree of life.

## Materials and methods

Protein predictions for 114 completely sequenced eukaryotic genomes were obtained from a variety of sources; for details, as well as information regarding numbers of protein predictions, see Additional file 1.

The domain repertoire for each genome was determined by hmmscan (with default options, except for an E-value cutoff of 2.0 and 'nobias') from the HMMER 3.0b2 package [61] using hidden Markov models from Pfam 24.0 [43]. In a second step, the hmmscan results were filtered by the domain specific 'gathering' (GA) cutoff scores provided by Pfam, followed by removal of domains of obvious viral, phage, or transposon origin (such as Pfam domain 'Viral_helicase1', a viral superfamily 1 RNA helicase). In case of overlapping domains, only the domain with the lowest E-value was retained.

Based on these preprocessing steps, a list of domains was created for each of the 114 genomes and, together with each of the three eukaryotic evolutionary trees described in the text, used for a Dollo parsimony [62] based inference of ancestral domain repertoires. The results of this step are lists of gained, lost, and present domains for each ancestral species.

In order to assess the robustness of our results relative to preprocessing steps, we also performed our analyses with a variety of different parameter combinations, such as uniform E-value based cutoffs ranging from 10^-4 ^to 10^-18^, as well as domain specific 'noise' (NC) and 'trusted' (TC) cutoff values from Pfam, with or without overlap and/or viral domain removal. We were unable to find a combination of these settings that would significantly change the numbers presented here and invalidate our conclusions. For example, Additional file 12 shows select domain counts for a variety of cutoff values. While, as expected, the absolute counts of domains are dependent on the cutoff value(s) used, overall tendencies (such as the LECA having an inferred domainome similar in size to that of extant mammals, and significant domain losses at the roots of deuterstome and ecdysozoa subtrees) are independent of the cutoff values used. Additional file 13 shows detailed gain and loss numbers under a uniform E-value-based cutoff of 10^-8^.

Pfam domains (lost, gained, and present) where mapped to GO terms by using the 'pfam2go' mapping (dated 2009/10/01) provided by the GO consortium [7]. GO term enrichment analysis for gained and lost domains was performed using the Ontologizer 2.0 software [63] with the Topology-Elim algorithm [64], which integrates the graph structure of the GO in testing for group enrichment. Enrichments are calculated relative to the union of all Pfam domains (with GO annotations) present in all genomes analyzed in this work. As summarized in Additional file 14, we tested whether different calculation methods in the Ontologizer 2.0 software (such as 'Topology-Weighted', 'Parent-Child-Union' or 'Parent-Child-Intersection' instead of 'Topology-Elim' [65]), as well as different approaches for multiple testing correction, would lead to noticeable different conclusions regarding enriched GO categories at various points during animal evolution. While the level of detail is dependent on the calculation method used (for example, 'Parent-Child-Union' and 'Parent-Child-Intersection' methods in general lead to very broad terms, whereas the other methods give more specific results), the results for each setting show predominantly gains in regulatory functions and losses in metabolic processes during animal evolution.

The preprocessing steps, the Dollo parsimony approach, and basic ancestral GO term analyses, were performed by software of our own design [66].

## Abbreviations

GO: gene ontology; LECA: last eukaryotic common ancestor.

## Authors' contributions

CMZ performed the analysis; CMZ and AG contributed to the research design and discussion on the manuscript; CMZ and AG wrote the manuscript. Both authors read and approved the final manuscript.

## Supplementary Material

Additional file 1 **Table of genomes analyzed**. Click here for file

Additional file 2 **Table of Pfam domain counts in extant species**. Summary of conditions used: protein predictions as listed in Additional file 1, domain models from Pfam 24.0, analyzed with HMMER 3.0b2, Pfam 'gathering' cutoffs. Click here for file

Additional file 3 **Domain gains and loss counts during eukaryote evolution**. Inferred domainome sizes are shown in blue, domain gain counts in green, and domain loss counts in red. Numbers in brackets are average domainome sizes of all extant descendents of each node. Summary of conditions used: protein predictions as listed in Additional file 1, domain models from Pfam 24.0, analyzed with HMMER 3.0b2, Pfam 'gathering' cutoffs. Click here for file

Additional file 4 **Domain gains and losses during eukaryote evolution**. phyloXML [70] formatted file, which was used to create Figure 2 and Additional file 3, viewable with Archaeopteryx software [71]. Summary of conditions used: protein predictions as listed in Additional file 1, domain models from Pfam 24.0, analyzed with HMMER 3.0b2, Pfam 'gathering' cutoffs. Click here for file

Additional file 5 **Domain gains and corresponding GO terms during eukaryote evolution**. Summary of conditions used: protein predictions as listed in Additional file 1, model of eukaryote evolution as shown in Figure 2 (and more detailed in Additional files 3 and 4), domain models from Pfam 24.0, analyzed with HMMER 3.0b2, Pfam 'gathering' cutoffs, 'pfam2go' mappings dated 2009/10/01. GO namespaces are abbreviated as follows: B, biological process; C, cellular component; M, molecular function. Click here for file

Additional file 6 **Domain losses and corresponding GO terms during eukaryote evolution**. Summary of conditions used: protein predictions as listed in Additional file 1, model of eukaryote evolution as shown in Figure 2 (and more detailed in Additional files 3 and 4), domain models from Pfam 24.0, analyzed with HMMER 3.0b2, Pfam 'gathering' cutoffs, 'pfam2go' mappings dated 2009/10/01. GO namespaces are abbreviated as follows: B, biological process; C, cellular component; M, molecular function. Click here for file

Additional file 7 **Domain gain and loss counts during eukaryote evolution under a coelomata model**. Summary of conditions used: protein predictions as listed in Additional file 1, domain models from Pfam 24.0, analyzed with HMMER 3.0b2, Pfam 'gathering' cutoffs. Click here for file

Additional file 8 **Domain gains and loss counts during eukaryote evolution under a 'crown group' model**. Summary of conditions used: protein predictions as listed in Additional file 1, domain models from Pfam 24.0, analyzed with HMMER 3.0b2, Pfam 'gathering' cutoffs. Click here for file

Additional file 9 **Table of enriched gained and lost GO terms evolution under a coelomata model**. The two terms with the lowest *P*-values are shown. Summary of conditions used: protein predictions as listed in Additional file 1, domain models from Pfam 24.0, analyzed with HMMER 3.0b2, Pfam 'gathering' cutoffs, model of eukaryote evolution as shown in Additional file 7, 'pfam2go' mappings dated 2009/10/01, Ontologizer 2.0 with Topology-Elim algorithm. Click here for file

Additional file 10 **Table of enriched gained and lost GO terms under a 'crown group' model**. The two terms with the lowest *P*-values are shown. Summary of conditions used: protein predictions as listed in Additional file 1, domain models from Pfam 24.0, analyzed with HMMER 3.0b2, Pfam 'gathering' cutoffs, model of eukaryote evolution as shown in Additional file 8, 'pfam2go' mappings dated 2009/10/01, Ontologizer 2.0 with Topology-Elim algorithm. Click here for file

Additional file 11 **Functional analysis of the human domainome complemented with intestinal bacteria**. Summary of conditions used: protein predictions as listed in Additional file 1, model of eukaryote evolution as shown in Figure 2 (and more detailed in Additional files 3 and 4), domain models from Pfam 24.0, analyzed with HMMER 3.0b2, Pfam 'gathering' cutoffs, 'pfam2go' mappings dated 2009/10/01. Click here for file

Additional file 12 **Domain counts for a variety of cutoff values**. Click here for file

Additional file 13 **Domain gains and losses during eukaryote evolution for a E-value cutoff of 10**^**-8**^. Summary of conditions used: protein predictions as listed in Additional file 1, domain models from Pfam 24.0, analyzed with HMMER 3.0b2. Click here for file

Additional file 14 **Comparison of enriched gained and lost GO terms along path from Unikonta to Mammalia using different calculation methods and different approaches for multiple testing correction**. The two terms with the lowest *P*-values are shown (calculated by the Ontologizer 2.0 software [63]), with the exception of terms marked by an asterisk, due to the relevance of these terms for this work. Prototypical regulatory terms are in red, prototypical metabolic terms are in blue. Click here for file
