# Supplementary figures and images for: Strong functional patterns in the evolution of eukaryotic genomes revealed by the reconstruction of ancestral protein domain repertoires
Source: Genome Biol. 2011 Jan 17;12(1):R4. doi: 10.1186/gb-2011-12-1-r4 (PMC3091302; doi:10.1186/gb-2011-12-1-r4)

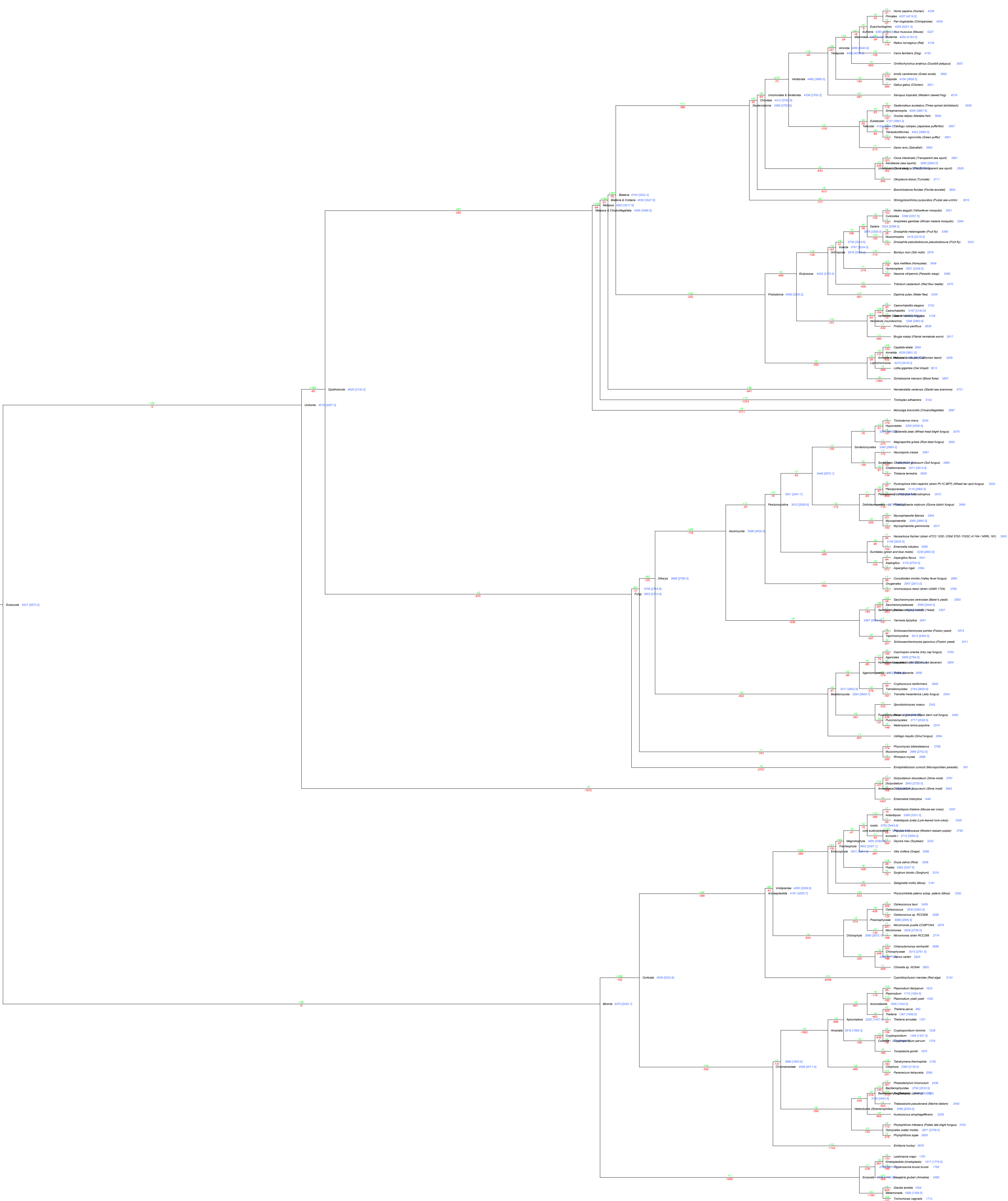

Supplement: Additional file 3 — Domain gains and loss counts during eukaryote evolution. Inferred domainome sizes are shown in blue, domain gain counts in green, and domain loss counts in red. Numbers in brackets are average domainome sizes of all extant descendents of each node. Summary of conditions used: protein predictions as listed in Additional file 1, domain models from Pfam 24.0, analyzed with HMMER 3.0b2, Pfam 'gathering' cutoffs. [file gb-2011-12-1-r4-S3.pdf]

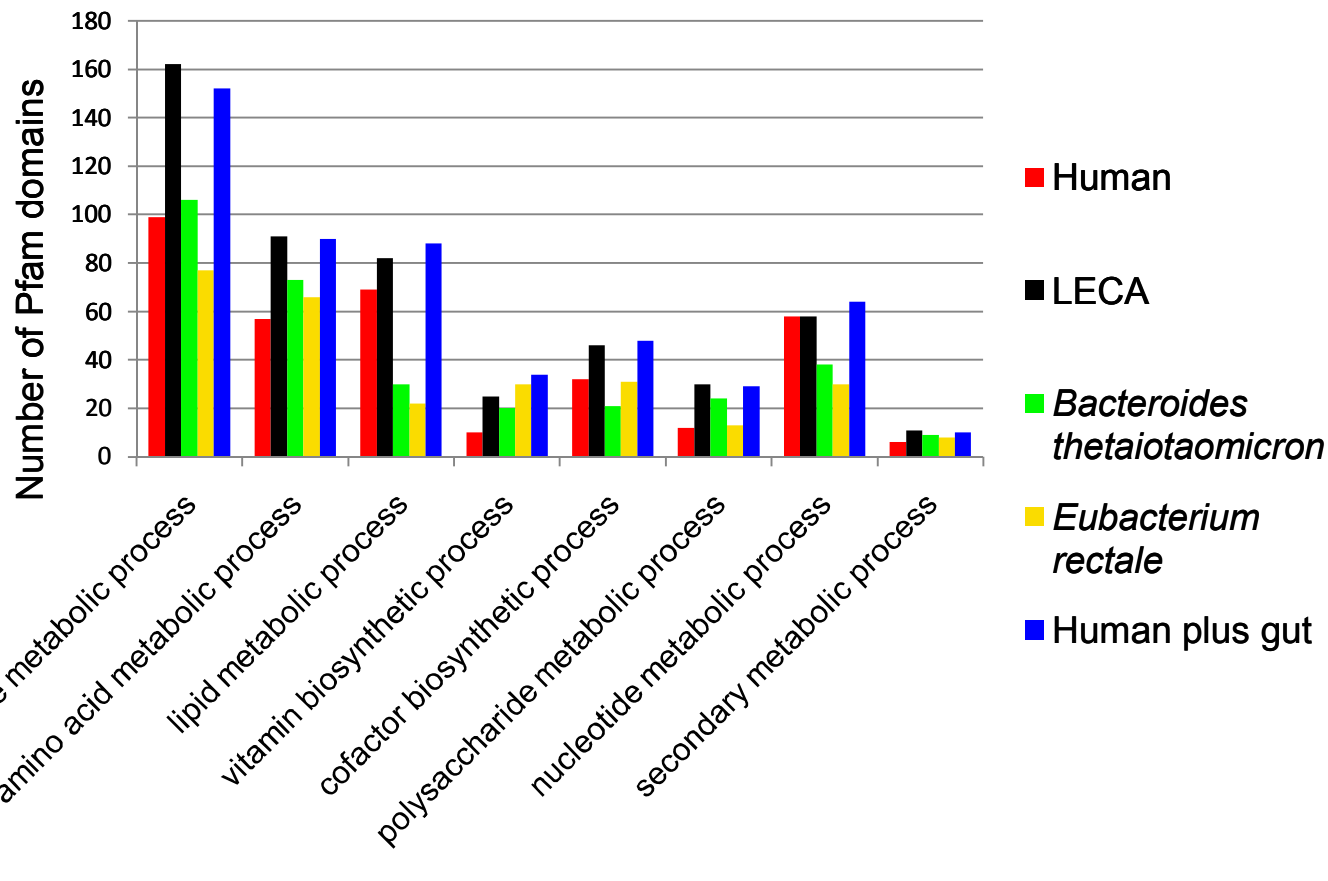

Supplement: Additional file 11 — Functional analysis of the human domainome complemented with intestinal bacteria. Summary of conditions used: protein predictions as listed in Additional file 1, model of eukaryote evolution as shown in Figure 2 (and more detailed in Additional files 3 and 4), domain models from Pfam 24.0, analyzed with HMMER 3.0b2, Pfam 'gathering' cutoffs, 'pfam2go' mappings dated 2009/10/01. [file gb-2011-12-1-r4-S11.pdf]
