# Supplementary material for: Strong functional patterns in the evolution of eukaryotic genomes revealed by the reconstruction of ancestral protein domain repertoires
Source: Genome Biol. 2011 Jan 17;12(1):R4. doi: 10.1186/gb-2011-12-1-r4 (PMC3091302; doi:10.1186/gb-2011-12-1-r4)
Supplement: Additional file 5 — Domain gains and corresponding GO terms during eukaryote evolution. Summary of conditions used: protein predictions as listed in Additional file 1, model of eukaryote evolution as shown in Figure 2 (and more detailed in Additional files 3 and 4), domain models from Pfam 24.0, analyzed with HMMER 3.0b2, Pfam 'gathering' cutoffs, 'pfam2go' mappings dated 2009/10/01. GO namespaces are abbreviated as follows: B, biological process; C, cellular component; M, molecular function. [file gb-2011-12-1-r4-S5.zip › Additional_File_5.html]

Dollo Parsimony | Gains | Domains


# Dollo Parsimony | Gains | Domains

|  |
| --- |
| Aconoidasida |
| Agaricales |
| Agaricomycotina |
| Alveolata |
| Amniota |
| Amoebozoa |
| Annelida |
| Annelida\_Mollusca |
| Apicomplexa |
| Arabidopsis |
| Archaeplastida |
| Arthropoda |
| Ascidiacea |
| Ascomycota |
| Aspergillus |
| Bacillariophycidae |
| Bacillariophyta |
| Basidiomycota |
| Bikonta |
| Bilateria |
| Bilateria\_Cnidaria |
| Caenorhabditis |
| Chaetomiaceae |
| Chlorophyceae |
| Chlorophyta |
| Chordata |
| Chromalveolate |
| Ciliophora |
| Coccidia |
| Corticata |
| Cryptosporidium |
| Culicoidea |
| Deuterostomia |
| Diapsida |
| Dictyostelium |
| Dikarya |
| Dikarya\_Mucoromycotina |
| Diptera |
| Dothideomycetes |
| Ecdysozoa |
| Embryophyta |
| Euarchontoglires |
| Eukaryota |
| Eurotiales |
| Euteleostei |
| Eutheria |
| Excavata |
| Fungi |
| Heterokonta |
| Heterokonta\_Alveolata |
| Homobasidiomycetes |
| Hymenoptera |
| Hypocreales |
| Insecta |
| Kinetoplastida |
| Kinetoplastida\_Heterolobosea |
| Lepidoptera\_Diptera |
| Lepidoptera\_Diptera\_Hymenoptera |
| Lophotrochozoa |
| Magnaporthales\_Hypocreales |
| Magnoliophyta |
| Mammalia |
| Metamonada |
| Metazoa |
| Metazoa\_Choanoflagellata |
| Micromonas |
| Mucoromycotina |
| Muscomorpha |
| Mycosphaerella |
| Nematoda |
| Neosartorya\_Emericella |
| Onygenales |
| Oomycetes |
| Opisthokonta |
| Ostreococcus |
| Pelagophyceae\_Bacillariophyta |
| Pezizomycotina |
| Plasmodium |
| Pleosporaceae |
| Pleosporales |
| Poales |
| Prasinophyceae |
| Primates |
| Protostomia |
| Pucciniomycetes |
| Pucciniomycotina |
| Pucciniomycotina\_Agaricomycotina |
| Rodentia |
| Saccharomycetaceae |
| Saccharomycotina |
| Saccharomycotina\_Taphrinomycotina |
| Smegmamorpha |
| Sordariales |
| Sordariomycetes |
| Sordariomycetes\_Dothideomycetes |
| Sordariomycetes\_Dothideomycetes\_Eurotiales |
| Taphrinomycotina |
| Teleostei |
| Tetraodontiformes |
| Tetrapoda |
| Theileria |
| Tracheophyta |
| Trebouxiophyceae\_Chlorophyceae |
| Tremellomycetes |
| Unikonta |
| Urochordata |
| Urochordata\_Vertebrata |
| Vertebrata |
| Viridiplantae |
| core eudicotyledons |
| eurosids I |
| nematode Clade V |
| rosids |

## Aconoidasida [eol|tol]

|  |  |  |  |
| --- | --- | --- | --- |
| **Pfam domain(s)** | **GO term acc** | **GO term** | **GO namespace** |
| + DUF528 | GO:0016226 | iron-sulfur cluster assembly | [B] |
| + s48\_45 |  |  |  |  |

---

## Agaricales [eol|tol]

|  |  |  |  |
| --- | --- | --- | --- |
| **Pfam domain(s)** | **GO term acc** | **GO term** | **GO namespace** |
| + Pheromone | GO:0000772 | mating pheromone activity | [M] |
|  | GO:0016020 | membrane | [C] |

---

## Agaricomycotina [eol|tol]

|  |  |  |  |
| --- | --- | --- | --- |
| **Pfam domain(s)** | **GO term acc** | **GO term** | **GO namespace** |

---

## Alveolata [eol|tol]

|  |  |  |  |
| --- | --- | --- | --- |
| **Pfam domain(s)** | **GO term acc** | **GO term** | **GO namespace** |

---

## Amniota [eol|tol]

|  |  |  |  |
| --- | --- | --- | --- |
| **Pfam domain(s)** | **GO term acc** | **GO term** | **GO namespace** |
| + Alveol-reg\_P311 |  |  |  |  |
| + ApoB100\_C |  |  |  |  |
| + BaffR-Tall\_bind |  |  |  |  |
| + BSP\_II | GO:0001503 | ossification | [B] |
|  | GO:0007155 | cell adhesion | [B] |
|  | GO:0005576 | extracellular region | [C] |
| + CAF-1\_p150 |  |  |  |  |
| + Cathelicidins | GO:0006952 | defense response | [B] |
|  | GO:0005576 | extracellular region | [C] |
| + CD47 |  |  |  |  |
| + CholecysA-Rec\_N |  |  |  |  |
| + Chon\_Sulph\_att |  |  |  |  |
| + Defensin\_beta | GO:0006952 | defense response | [B] |
|  | GO:0005576 | extracellular region | [C] |
| + DUF3699 |  |  |  |  |
| + Fanconi\_A |  |  |  |  |
| + Flt3\_lig | GO:0005125 | cytokine activity | [M] |
|  | GO:0016020 | membrane | [C] |
| + GM\_CSF | GO:0005129 | granulocyte macrophage colony-stimulating factor receptor binding | [M] |
|  | GO:0008083 | growth factor activity | [M] |
|  | GO:0006955 | immune response | [B] |
|  | GO:0005576 | extracellular region | [C] |
| + IL5 | GO:0005137 | interleukin-5 receptor binding | [M] |
|  | GO:0008083 | growth factor activity | [M] |
|  | GO:0006955 | immune response | [B] |
|  | GO:0005576 | extracellular region | [C] |
| + Motilin\_ghrelin | GO:0005179 | hormone activity | [M] |
|  | GO:0005576 | extracellular region | [C] |
| + NRIF3 |  |  |  |  |
| + PBP\_sp32 |  |  |  |  |
| + Phospholamban | GO:0005246 | calcium channel regulator activity | [M] |
|  | GO:0042030 | ATPase inhibitor activity | [M] |
|  | GO:0006816 | calcium ion transport | [B] |
|  | GO:0016020 | membrane | [C] |
| + PPAK |  |  |  |  |
| + Prion\_bPrPp |  |  |  |  |
| + Receptor\_2B4 |  |  |  |  |
| + SP\_C-Propep |  |  |  |  |
| + TACI-CRD2 |  |  |  |  |
| + Tcell\_CD4\_Cterm |  |  |  |  |
| + TCL1\_MTCP1 |  |  |  |  |
| + Treacle |  |  |  |  |
| + Uteroglobin | GO:0005488 | binding | [M] |
|  | GO:0005576 | extracellular region | [C] |

---

## Amoebozoa [eol|tol]

|  |  |  |  |
| --- | --- | --- | --- |
| **Pfam domain(s)** | **GO term acc** | **GO term** | **GO namespace** |

---

## Annelida [eol|tol]

|  |  |  |  |
| --- | --- | --- | --- |
| **Pfam domain(s)** | **GO term acc** | **GO term** | **GO namespace** |

---

## Annelida\_Mollusca

|  |  |  |  |
| --- | --- | --- | --- |
| **Pfam domain(s)** | **GO term acc** | **GO term** | **GO namespace** |

---

## Apicomplexa [eol|tol]

|  |  |  |  |
| --- | --- | --- | --- |
| **Pfam domain(s)** | **GO term acc** | **GO term** | **GO namespace** |
| + AMA-1 | GO:0009405 | pathogenesis | [B] |
|  | GO:0016020 | membrane | [C] |
| + DUF2981 |  |  |  |  |
| + DUF3273 |  |  |  |  |

---

## Arabidopsis [eol|tol]

|  |  |  |  |
| --- | --- | --- | --- |
| **Pfam domain(s)** | **GO term acc** | **GO term** | **GO namespace** |
| + DUF1163 |  |  |  |  |
| + DUF1184 |  |  |  |  |
| + DUF1985 |  |  |  |  |
| + DUF287 |  |  |  |  |
| + DUF601 |  |  |  |  |
| + DUF626 |  |  |  |  |
| + Nodulin\_late | GO:0046872 | metal ion binding | [M] |
|  | GO:0009878 | nodule morphogenesis | [B] |
| + Plant\_all\_beta |  |  |  |  |
| + TAP35\_44 |  |  |  |  |
| + Toxin\_3 | GO:0008200 | ion channel inhibitor activity | [M] |
|  | GO:0005576 | extracellular region | [C] |

---

## Archaeplastida [eol|tol]

|  |  |  |  |
| --- | --- | --- | --- |
| **Pfam domain(s)** | **GO term acc** | **GO term** | **GO namespace** |
| + Apocytochr\_F\_C | GO:0005506 | iron ion binding | [M] |
|  | GO:0009055 | electron carrier activity | [M] |
|  | GO:0020037 | heme binding | [M] |
|  | GO:0015979 | photosynthesis | [B] |
|  | GO:0031361 | integral to thylakoid membrane | [C] |
| + CemA | GO:0016021 | integral to membrane | [C] |
| + Cytochrom\_B559 | GO:0046872 | metal ion binding | [M] |
|  | GO:0015979 | photosynthesis | [B] |
|  | GO:0009523 | photosystem II | [C] |
|  | GO:0009536 | plastid | [C] |
|  | GO:0009579 | thylakoid | [C] |
|  | GO:0016021 | integral to membrane | [C] |
| + Cytochrom\_B559a | GO:0046872 | metal ion binding | [M] |
|  | GO:0015979 | photosynthesis | [B] |
|  | GO:0009523 | photosystem II | [C] |
|  | GO:0016021 | integral to membrane | [C] |
| + DUF151 |  |  |  |  |
| + DUF1730 |  |  |  |  |
| + DUF177 |  |  |  |  |
| + DUF1818 |  |  |  |  |
| + DUF2301 |  |  |  |  |
| + DUF2505 |  |  |  |  |
| + DUF2741 |  |  |  |  |
| + DUF3769 |  |  |  |  |
| + DUF561 |  |  |  |  |
| + DUF639 |  |  |  |  |
| + ExsB |  |  |  |  |
| + LdpA\_C |  |  |  |  |
| + PetG | GO:0009512 | cytochrome b6f complex | [C] |
| + PetM | GO:0009512 | cytochrome b6f complex | [C] |
| + PetN | GO:0045158 | electron transporter, transferring electrons within cytochrome b6/f complex of photosystem II activity | [M] |
|  | GO:0017004 | cytochrome complex assembly | [B] |
|  | GO:0009512 | cytochrome b6f complex | [C] |
| + Photo\_RC | GO:0045156 | electron transporter, transferring electrons within the cyclic electron transport pathway of photosynthesis activity | [M] |
|  | GO:0009772 | photosynthetic electron transport in photosystem II | [B] |
|  | GO:0019684 | photosynthesis, light reaction | [B] |
|  | GO:0030077 | plasma membrane light-harvesting complex | [C] |
| + PLATZ |  |  |  |  |
| + PsaL | GO:0015979 | photosynthesis | [B] |
|  | GO:0009538 | photosystem I reaction center | [C] |
| + PsbH | GO:0042301 | phosphate binding | [M] |
|  | GO:0050821 | protein stabilization | [B] |
|  | GO:0009523 | photosystem II | [C] |
|  | GO:0016020 | membrane | [C] |
| + PsbI | GO:0015979 | photosynthesis | [B] |
|  | GO:0009539 | photosystem II reaction center | [C] |
|  | GO:0016020 | membrane | [C] |
| + PsbJ | GO:0015979 | photosynthesis | [B] |
|  | GO:0009539 | photosystem II reaction center | [C] |
|  | GO:0016020 | membrane | [C] |
| + PsbK | GO:0015979 | photosynthesis | [B] |
|  | GO:0009539 | photosystem II reaction center | [C] |
| + PsbL | GO:0015979 | photosynthesis | [B] |
|  | GO:0009539 | photosystem II reaction center | [C] |
|  | GO:0016020 | membrane | [C] |
| + PsbN | GO:0015979 | photosynthesis | [B] |
|  | GO:0009539 | photosystem II reaction center | [C] |
|  | GO:0016020 | membrane | [C] |
| + PsbT | GO:0015979 | photosynthesis | [B] |
|  | GO:0009539 | photosystem II reaction center | [C] |
|  | GO:0016020 | membrane | [C] |
| + PsbX | GO:0015979 | photosynthesis | [B] |
|  | GO:0009523 | photosystem II | [C] |
|  | GO:0016020 | membrane | [C] |
| + PsbY | GO:0030145 | manganese ion binding | [M] |
|  | GO:0015979 | photosynthesis | [B] |
|  | GO:0009523 | photosystem II | [C] |
|  | GO:0016021 | integral to membrane | [C] |
| + PSI\_PsaJ | GO:0015979 | photosynthesis | [B] |
|  | GO:0009522 | photosystem I | [C] |
| + PSI\_PSAK | GO:0015979 | photosynthesis | [B] |
|  | GO:0009522 | photosystem I | [C] |
|  | GO:0016020 | membrane | [C] |
| + PSII | GO:0016168 | chlorophyll binding | [M] |
|  | GO:0009767 | photosynthetic electron transport chain | [B] |
|  | GO:0019684 | photosynthesis, light reaction | [B] |
|  | GO:0009521 | photosystem | [C] |
|  | GO:0016020 | membrane | [C] |
| + PSRP-3\_Ycf65 | GO:0003735 | structural constituent of ribosome | [M] |
|  | GO:0006412 | translation | [B] |
|  | GO:0005622 | intracellular | [C] |
|  | GO:0005840 | ribosome | [C] |
|  | GO:0009536 | plastid | [C] |
| + ResB |  |  |  |  |
| + RuBisCO\_large | GO:0000287 | magnesium ion binding | [M] |
|  | GO:0016984 | ribulose-bisphosphate carboxylase activity | [M] |
|  | GO:0015977 | carbon utilization by fixation of carbon dioxide | [B] |
|  | GO:0009536 | plastid | [C] |
| + RuBisCO\_large\_N | GO:0000287 | magnesium ion binding | [M] |
|  | GO:0016984 | ribulose-bisphosphate carboxylase activity | [M] |
|  | GO:0015977 | carbon utilization by fixation of carbon dioxide | [B] |
| + RuBisCO\_small | GO:0016984 | ribulose-bisphosphate carboxylase activity | [M] |
|  | GO:0015977 | carbon utilization by fixation of carbon dioxide | [B] |
| + tRNA\_synt\_2f | GO:0000166 | nucleotide binding | [M] |
|  | GO:0004820 | glycine-tRNA ligase activity | [M] |
|  | GO:0005524 | ATP binding | [M] |
|  | GO:0006412 | translation | [B] |
|  | GO:0006426 | glycyl-tRNA aminoacylation | [B] |
|  | GO:0005737 | cytoplasm | [C] |
| + Ycf4 | GO:0015979 | photosynthesis | [B] |
|  | GO:0009579 | thylakoid | [C] |
|  | GO:0016021 | integral to membrane | [C] |
| + Ycf54 |  |  |  |  |
| + Ycf9 | GO:0042549 | photosystem II stabilization | [B] |
|  | GO:0009539 | photosystem II reaction center | [C] |
| + YMF19 | GO:0015078 | hydrogen ion transmembrane transporter activity | [M] |
|  | GO:0015986 | ATP synthesis coupled proton transport | [B] |
|  | GO:0000276 | mitochondrial proton-transporting ATP synthase complex, coupling factor F(o) | [C] |

---

## Arthropoda [eol|tol]

|  |  |  |  |
| --- | --- | --- | --- |
| **Pfam domain(s)** | **GO term acc** | **GO term** | **GO namespace** |
| + Chitin\_bind\_4 | GO:0042302 | structural constituent of cuticle | [M] |
| + CLIP |  |  |  |  |
| + DM4\_12 |  |  |  |  |
| + Dscam\_C |  |  |  |  |
| + DUF1397 |  |  |  |  |
| + Eclosion | GO:0008255 | ecdysis-triggering hormone activity | [M] |
|  | GO:0007218 | neuropeptide signaling pathway | [B] |
|  | GO:0018990 | ecdysis, chitin-based cuticle | [B] |
| + Hemocyanin\_N |  |  |  |  |
| + JHBP |  |  |  |  |
| + Neuroparsin |  |  |  |  |
| + OS-D |  |  |  |  |
| + Pigment\_DH | GO:0005179 | hormone activity | [M] |
|  | GO:0009416 | response to light stimulus | [B] |
|  | GO:0005576 | extracellular region | [C] |
| + SDP\_N |  |  |  |  |
| + SspB |  |  |  |  |
| + Toxin\_9 | GO:0008200 | ion channel inhibitor activity | [M] |
|  | GO:0009405 | pathogenesis | [B] |
|  | GO:0005576 | extracellular region | [C] |

---

## Ascidiacea [eol|tol]

|  |  |  |  |
| --- | --- | --- | --- |
| **Pfam domain(s)** | **GO term acc** | **GO term** | **GO namespace** |
| + Vanabin-2 |  |  |  |  |

---

## Ascomycota [eol|tol]

|  |  |  |  |
| --- | --- | --- | --- |
| **Pfam domain(s)** | **GO term acc** | **GO term** | **GO namespace** |
| + Aft1\_HRR |  |  |  |  |
| + Alb1 | GO:0042273 | ribosomal large subunit biogenesis | [B] |
| + Apc15p | GO:0005515 | protein binding | [M] |
|  | GO:0030071 | regulation of mitotic metaphase/anaphase transition | [B] |
|  | GO:0031145 | anaphase-promoting complex-dependent proteasomal ubiquitin-dependent protein catabolic process | [B] |
|  | GO:0005680 | anaphase-promoting complex | [C] |
| + Bul1\_C |  |  |  |  |
| + Bul1\_N |  |  |  |  |
| + Candida\_ALS | GO:0007155 | cell adhesion | [B] |
| + Carb\_bind |  |  |  |  |
| + Carbpep\_Y\_N | GO:0004185 | serine-type carboxypeptidase activity | [M] |
| + Cut12 |  |  |  |  |
| + DUF1761 |  |  |  |  |
| + DUF1774 |  |  |  |  |
| + DUF2011 |  |  |  |  |
| + DUF2014 |  |  |  |  |
| + DUF2417 |  |  |  |  |
| + DUF2434 |  |  |  |  |
| + DUF2457 |  |  |  |  |
| + DUF2697 |  |  |  |  |
| + DUF2722 |  |  |  |  |
| + DUF2731 |  |  |  |  |
| + DUF3112 |  |  |  |  |
| + DUF3210 |  |  |  |  |
| + DUF3507 |  |  |  |  |
| + Fig1 |  |  |  |  |
| + Fun\_ATP-synt\_8 | GO:0015078 | hydrogen ion transmembrane transporter activity | [M] |
|  | GO:0015986 | ATP synthesis coupled proton transport | [B] |
|  | GO:0000276 | mitochondrial proton-transporting ATP synthase complex, coupling factor F(o) | [C] |
| + Gal4\_dimer |  |  |  |  |
| + Gon7 |  |  |  |  |
| + Hom\_end | GO:0003677 | DNA binding | [M] |
|  | GO:0004519 | endonuclease activity | [M] |
|  | GO:0030908 | protein splicing | [B] |
| + Ilm1 |  |  |  |  |
| + KRE9 | GO:0042546 | cell wall biogenesis | [B] |
|  | GO:0005576 | extracellular region | [C] |
| + MAT\_Alpha1 | GO:0000772 | mating pheromone activity | [M] |
|  | GO:0003677 | DNA binding | [M] |
|  | GO:0045895 | positive regulation of transcription, mating-type specific | [B] |
|  | GO:0005634 | nucleus | [C] |
| + NPCC |  |  |  |  |
| + PA14\_2 |  |  |  |  |
| + PalH |  |  |  |  |
| + PET122 | GO:0003743 | translation initiation factor activity | [M] |
|  | GO:0006413 | translational initiation | [B] |
|  | GO:0005740 | mitochondrial envelope | [C] |
| + Pga1 |  |  |  |  |
| + PGA2 |  |  |  |  |
| + PIR |  |  |  |  |
| + PMP1\_2 | GO:0006812 | cation transport | [B] |
|  | GO:0016021 | integral to membrane | [C] |
| + Rap1-DNA-bind |  |  |  |  |
| + Rick\_17kDa\_Anti | GO:0019867 | outer membrane | [C] |
| + RNA\_polI\_A14 |  |  |  |  |
| + Rox3 | GO:0016455 | RNA polymerase II transcription mediator activity | [M] |
|  | GO:0006357 | regulation of transcription from RNA polymerase II promoter | [B] |
|  | GO:0016592 | mediator complex | [C] |
| + RRM |  |  |  |  |
| + RRN9 |  |  |  |  |
| + SKG6 |  |  |  |  |
| + Stb3 |  |  |  |  |
| + Stn1 |  |  |  |  |
| + U3\_snoRNA\_assoc |  |  |  |  |
| + UAF\_Rrn10 |  |  |  |  |

---

## Aspergillus [eol|tol]

|  |  |  |  |
| --- | --- | --- | --- |
| **Pfam domain(s)** | **GO term acc** | **GO term** | **GO namespace** |

---

## Bacillariophycidae [eol|tol]

|  |  |  |  |
| --- | --- | --- | --- |
| **Pfam domain(s)** | **GO term acc** | **GO term** | **GO namespace** |
| + DUF318 |  |  |  |  |

---

## Bacillariophyta [eol|tol]

|  |  |  |  |
| --- | --- | --- | --- |
| **Pfam domain(s)** | **GO term acc** | **GO term** | **GO namespace** |
| + Lact-deh-memb | GO:0008720 | D-lactate dehydrogenase activity | [M] |
|  | GO:0050660 | FAD binding | [M] |
|  | GO:0055085 | transmembrane transport | [B] |
|  | GO:0055114 | oxidation reduction | [B] |
| + RusA | GO:0000287 | magnesium ion binding | [M] |
|  | GO:0006281 | DNA repair | [B] |
|  | GO:0006310 | DNA recombination | [B] |

---

## Basidiomycota [eol|tol]

|  |  |  |  |
| --- | --- | --- | --- |
| **Pfam domain(s)** | **GO term acc** | **GO term** | **GO namespace** |

---

## Bikonta [eol|tol]

|  |  |  |  |
| --- | --- | --- | --- |
| **Pfam domain(s)** | **GO term acc** | **GO term** | **GO namespace** |
| + 2\_5\_RNA\_ligase |  |  |  |  |
| + 3D | GO:0004553 | hydrolase activity, hydrolyzing O-glycosyl compounds | [M] |
|  | GO:0009254 | peptidoglycan turnover | [B] |
|  | GO:0019867 | outer membrane | [C] |
| + Adenosine\_kin |  |  |  |  |
| + CaATP\_NAI |  |  |  |  |
| + Cytokin-bind | GO:0019139 | cytokinin dehydrogenase activity | [M] |
|  | GO:0050660 | FAD binding | [M] |
|  | GO:0009690 | cytokinin metabolic process | [B] |
|  | GO:0055114 | oxidation reduction | [B] |
| + DUF1499 |  |  |  |  |
| + DUF1861 |  |  |  |  |
| + DUF2779 |  |  |  |  |
| + DUF2817 |  |  |  |  |
| + DUF488 |  |  |  |  |
| + DUF501 |  |  |  |  |
| + DUF677 |  |  |  |  |
| + DUF784 |  |  |  |  |
| + DUF789 |  |  |  |  |
| + DUF847 |  |  |  |  |
| + DUF901 |  |  |  |  |
| + DUF912 |  |  |  |  |
| + Ecotin |  |  |  |  |
| + ETRAMP |  |  |  |  |
| + IpgD | GO:0016791 | phosphatase activity | [M] |
|  | GO:0009405 | pathogenesis | [B] |
| + Kinesin-related |  |  |  |  |
| + OpuAC | GO:0005215 | transporter activity | [M] |
|  | GO:0005488 | binding | [M] |
|  | GO:0006810 | transport | [B] |
| + PaO | GO:0010277 | chlorophyllide a oxygenase activity | [M] |
|  | GO:0055114 | oxidation reduction | [B] |
| + PBS\_linker\_poly | GO:0015979 | photosynthesis | [B] |
|  | GO:0030089 | phycobilisome | [C] |
| + Penicil\_amidase | GO:0016787 | hydrolase activity | [M] |
|  | GO:0017000 | antibiotic biosynthetic process | [B] |
| + PLD\_C |  |  |  |  |
| + PriCT\_2 |  |  |  |  |
| + Prim-Pol |  |  |  |  |
| + PSI\_8 | GO:0015979 | photosynthesis | [B] |
|  | GO:0009522 | photosystem I | [C] |
| + RAP1 |  |  |  |  |
| + SF-assemblin | GO:0005200 | structural constituent of cytoskeleton | [M] |
| + SLH |  |  |  |  |
| + Strep\_SA\_rep |  |  |  |  |
| + Sugar\_transport | GO:0015144 | carbohydrate transmembrane transporter activity | [M] |
|  | GO:0008643 | carbohydrate transport | [B] |
|  | GO:0016021 | integral to membrane | [C] |
| + ThiI | GO:0003723 | RNA binding | [M] |
|  | GO:0009228 | thiamin biosynthetic process | [B] |
|  | GO:0005737 | cytoplasm | [C] |
| + TilS\_C |  |  |  |  |
| + Trypan\_PARP | GO:0016020 | membrane | [C] |
| + ydhR |  |  |  |  |
| + zf-LSD1 |  |  |  |  |

---

## Bilateria [eol|tol]

|  |  |  |  |
| --- | --- | --- | --- |
| **Pfam domain(s)** | **GO term acc** | **GO term** | **GO namespace** |
| + 7TM\_GPCR\_Srx |  |  |  |  |
| + Activator\_LAG-3 |  |  |  |  |
| + Adeno\_E3\_CR2 |  |  |  |  |
| + Adeno\_IVa2 | GO:0019083 | viral transcription | [B] |
| + AKAP95 | GO:0003677 | DNA binding | [M] |
|  | GO:0005634 | nucleus | [C] |
| + AMOP |  |  |  |  |
| + APC\_15aa | GO:0005515 | protein binding | [M] |
|  | GO:0016055 | Wnt receptor signaling pathway | [B] |
| + APC\_crr | GO:0016055 | Wnt receptor signaling pathway | [B] |
| + Atrophin-1 |  |  |  |  |
| + Axin\_b-cat\_bind |  |  |  |  |
| + BAMBI |  |  |  |  |
| + Basic | GO:0003677 | DNA binding | [M] |
|  | GO:0006355 | regulation of transcription, DNA-dependent | [B] |
|  | GO:0007517 | muscle organ development | [B] |
|  | GO:0005634 | nucleus | [C] |
| + Bcl-2\_BAD |  |  |  |  |
| + BCL9 |  |  |  |  |
| + Bclx\_interact |  |  |  |  |
| + BESS | GO:0003677 | DNA binding | [M] |
| + BH4 | GO:0042981 | regulation of apoptosis | [B] |
| + BPS |  |  |  |  |
| + C1-set |  |  |  |  |
| + C2-set\_2 |  |  |  |  |
| + Cadherin\_2 |  |  |  |  |
| + Cadherin\_pro |  |  |  |  |
| + CaMBD | GO:0005516 | calmodulin binding | [M] |
|  | GO:0015269 | calcium-activated potassium channel activity | [M] |
|  | GO:0006813 | potassium ion transport | [B] |
|  | GO:0016021 | integral to membrane | [C] |
| + CARM1 |  |  |  |  |
| + CFC |  |  |  |  |
| + Clc-like | GO:0016021 | integral to membrane | [C] |
| + Cmyb\_C |  |  |  |  |
| + CNPase | GO:0004113 | 2',3'-cyclic-nucleotide 3'-phosphodiesterase activity | [M] |
|  | GO:0009214 | cyclic nucleotide catabolic process | [B] |
|  | GO:0016020 | membrane | [C] |
| + COMP |  |  |  |  |
| + CortBP2 |  |  |  |  |
| + COX6C | GO:0004129 | cytochrome-c oxidase activity | [M] |
| + COX8 | GO:0004129 | cytochrome-c oxidase activity | [M] |
| + Creb\_binding | GO:0003713 | transcription coactivator activity | [M] |
|  | GO:0004402 | histone acetyltransferase activity | [M] |
|  | GO:0016573 | histone acetylation | [B] |
|  | GO:0045449 | regulation of transcription | [B] |
|  | GO:0000123 | histone acetyltransferase complex | [C] |
|  | GO:0005634 | nucleus | [C] |
| + CRF | GO:0005179 | hormone activity | [M] |
|  | GO:0005576 | extracellular region | [C] |
| + CRF-BP |  |  |  |  |
| + CRIC\_ras\_sig |  |  |  |  |
| + Crisp |  |  |  |  |
| + Crystallin | GO:0005212 | structural constituent of eye lens | [M] |
| + CTNNB1\_binding |  |  |  |  |
| + DB |  |  |  |  |
| + DC\_STAMP | GO:0016021 | integral to membrane | [C] |
| + DDDD |  |  |  |  |
| + Dishevelled | GO:0004871 | signal transducer activity | [M] |
|  | GO:0007275 | multicellular organismal development | [B] |
| + DLL\_N |  |  |  |  |
| + DNA\_pack\_N | GO:0006323 | DNA packaging | [B] |
| + Drf\_DAD |  |  |  |  |
| + Drf\_FH1 |  |  |  |  |
| + DUF1081 | GO:0005319 | lipid transporter activity | [M] |
|  | GO:0006869 | lipid transport | [B] |
| + DUF1111 |  |  |  |  |
| + DUF1239 |  |  |  |  |
| + DUF1370 |  |  |  |  |
| + DUF1679 |  |  |  |  |
| + DUF1758 |  |  |  |  |
| + DUF187 |  |  |  |  |
| + DUF1875 |  |  |  |  |
| + DUF1880 |  |  |  |  |
| + DUF1898 |  |  |  |  |
| + DUF2369 |  |  |  |  |
| + DUF2371 |  |  |  |  |
| + DUF2448 |  |  |  |  |
| + DUF2668 |  |  |  |  |
| + DUF3050 |  |  |  |  |
| + DUF3166 |  |  |  |  |
| + DUF3259 |  |  |  |  |
| + DUF3454 |  |  |  |  |
| + DUF3643 |  |  |  |  |
| + DUF3736 |  |  |  |  |
| + DUF3740 |  |  |  |  |
| + DUF646 |  |  |  |  |
| + DUF737 |  |  |  |  |
| + DUF750 | GO:0005515 | protein binding | [M] |
|  | GO:0006516 | glycoprotein catabolic process | [B] |
|  | GO:0005737 | cytoplasm | [C] |
| + DUF753 |  |  |  |  |
| + DuoxA | GO:0015031 | protein transport | [B] |
|  | GO:0005789 | endoplasmic reticulum membrane | [C] |
|  | GO:0016021 | integral to membrane | [C] |
| + EB |  |  |  |  |
| + EDC3\_LSm |  |  |  |  |
| + Endostatin | GO:0005198 | structural molecule activity | [M] |
|  | GO:0007155 | cell adhesion | [B] |
|  | GO:0031012 | extracellular matrix | [C] |
| + Engrail\_1\_C\_sig |  |  |  |  |
| + ESSS |  |  |  |  |
| + F\_actin\_bind | GO:0004715 | non-membrane spanning protein tyrosine kinase activity | [M] |
|  | GO:0005524 | ATP binding | [M] |
|  | GO:0006468 | protein amino acid phosphorylation | [B] |
| + Fanconi\_C | GO:0006281 | DNA repair | [B] |
| + FCP1\_C |  |  |  |  |
| + FGF-BP1 |  |  |  |  |
| + FOLN |  |  |  |  |
| + FXR1P\_C |  |  |  |  |
| + G2F |  |  |  |  |
| + GABP-alpha |  |  |  |  |
| + Gly\_acyl\_tr\_N |  |  |  |  |
| + GSK-3\_bind |  |  |  |  |
| + GTF2I |  |  |  |  |
| + GTPase\_binding |  |  |  |  |
| + Hepcidin | GO:0006879 | cellular iron ion homeostasis | [B] |
|  | GO:0005576 | extracellular region | [C] |
| + Hermes\_DBD |  |  |  |  |
| + HIF-1 |  |  |  |  |
| + HIF-1a\_CTAD |  |  |  |  |
| + Hormone\_3 | GO:0005179 | hormone activity | [M] |
|  | GO:0005576 | extracellular region | [C] |
| + Hormone\_5 | GO:0005185 | neurohypophyseal hormone activity | [M] |
|  | GO:0005576 | extracellular region | [C] |
| + ICAP-1\_inte\_bdg |  |  |  |  |
| + IL15 | GO:0005126 | cytokine receptor binding | [M] |
|  | GO:0006955 | immune response | [B] |
|  | GO:0005576 | extracellular region | [C] |
| + IL17 | GO:0005125 | cytokine activity | [M] |
|  | GO:0006954 | inflammatory response | [B] |
|  | GO:0005576 | extracellular region | [C] |
| + IL6Ra-bind |  |  |  |  |
| + IL7 | GO:0005126 | cytokine receptor binding | [M] |
|  | GO:0008083 | growth factor activity | [M] |
|  | GO:0006955 | immune response | [B] |
|  | GO:0005576 | extracellular region | [C] |
| + Integrin\_alpha |  |  |  |  |
| + IR1-M |  |  |  |  |
| + ITI\_HC\_C | GO:0004867 | serine-type endopeptidase inhibitor activity | [M] |
|  | GO:0030212 | hyaluronan metabolic process | [B] |
| + KASH | GO:0003779 | actin binding | [M] |
|  | GO:0016021 | integral to membrane | [C] |
| + KCNQ\_channel |  |  |  |  |
| + KN\_motif |  |  |  |  |
| + Laminin\_I | GO:0005102 | receptor binding | [M] |
|  | GO:0030155 | regulation of cell adhesion | [B] |
|  | GO:0030334 | regulation of cell migration | [B] |
|  | GO:0045995 | regulation of embryonic development | [B] |
|  | GO:0005606 | laminin-1 complex | [C] |
| + Laps |  |  |  |  |
| + Lectin\_N | GO:0005529 | sugar binding | [M] |
|  | GO:0016020 | membrane | [C] |
| + LEDGF |  |  |  |  |
| + LolA | GO:0015031 | protein transport | [B] |
|  | GO:0030288 | outer membrane-bounded periplasmic space | [C] |
| + Lys |  |  |  |  |
| + M-inducer\_phosp | GO:0004725 | protein tyrosine phosphatase activity | [M] |
|  | GO:0000087 | M phase of mitotic cell cycle | [B] |
|  | GO:0006470 | protein amino acid dephosphorylation | [B] |
|  | GO:0005622 | intracellular | [C] |
| + MAP2\_projctn |  |  |  |  |
| + MbeD\_MobD |  |  |  |  |
| + MGC-24 |  |  |  |  |
| + MRP-L51 |  |  |  |  |
| + MRP-S27 |  |  |  |  |
| + Myelin\_PLP |  |  |  |  |
| + Myf5 |  |  |  |  |
| + NADH\_dehy\_S2\_C | GO:0008137 | NADH dehydrogenase (ubiquinone) activity | [M] |
|  | GO:0006120 | mitochondrial electron transport, NADH to ubiquinone | [B] |
|  | GO:0055114 | oxidation reduction | [B] |
| + NADH\_oxidored | GO:0003954 | NADH dehydrogenase activity | [M] |
|  | GO:0005739 | mitochondrion | [C] |
| + NDUF\_B5 |  |  |  |  |
| + NDUF\_B6 |  |  |  |  |
| + NDUF\_C2 | GO:0008137 | NADH dehydrogenase (ubiquinone) activity | [M] |
|  | GO:0006120 | mitochondrial electron transport, NADH to ubiquinone | [B] |
|  | GO:0005743 | mitochondrial inner membrane | [C] |
| + NEMO |  |  |  |  |
| + Neogenin\_C | GO:0016021 | integral to membrane | [C] |
| + Neurexophilin |  |  |  |  |
| + Neuro\_bHLH |  |  |  |  |
| + NUC205 | GO:0005634 | nucleus | [C] |
| + NumbF |  |  |  |  |
| + Oxidored\_q5\_N | GO:0008137 | NADH dehydrogenase (ubiquinone) activity | [M] |
|  | GO:0006120 | mitochondrial electron transport, NADH to ubiquinone | [B] |
|  | GO:0055114 | oxidation reduction | [B] |
|  | GO:0005739 | mitochondrion | [C] |
| + P53\_TAD |  |  |  |  |
| + Period\_C |  |  |  |  |
| + Phasin\_2 |  |  |  |  |
| + Phe\_ZIP | GO:0004871 | signal transducer activity | [M] |
|  | GO:0005515 | protein binding | [M] |
|  | GO:0007242 | intracellular signaling cascade | [B] |
| + PKI | GO:0004862 | cAMP-dependent protein kinase inhibitor activity | [M] |
|  | GO:0006469 | negative regulation of protein kinase activity | [B] |
| + Plectin |  |  |  |  |
| + PMG |  |  |  |  |
| + Popeye | GO:0016020 | membrane | [C] |
| + Prokineticin |  |  |  |  |
| + Protocadherin |  |  |  |  |
| + Prox1 | GO:0003677 | DNA binding | [M] |
|  | GO:0030528 | transcription regulator activity | [M] |
|  | GO:0007275 | multicellular organismal development | [B] |
|  | GO:0045449 | regulation of transcription | [B] |
|  | GO:0005634 | nucleus | [C] |
| + PSP94 |  |  |  |  |
| + PTN\_MK\_C | GO:0008083 | growth factor activity | [M] |
| + PTN\_MK\_N | GO:0008083 | growth factor activity | [M] |
| + PV-1 |  |  |  |  |
| + Rabaptin | GO:0005096 | GTPase activator activity | [M] |
|  | GO:0008083 | growth factor activity | [M] |
| + RabGGT\_insert | GO:0004663 | Rab geranylgeranyltransferase activity | [M] |
|  | GO:0008270 | zinc ion binding | [M] |
| + RCSD |  |  |  |  |
| + Receptor\_IA-2 |  |  |  |  |
| + RNA\_pol\_Rpb1\_6 | GO:0003677 | DNA binding | [M] |
|  | GO:0003899 | DNA-directed RNA polymerase activity | [M] |
|  | GO:0006350 | transcription | [B] |
| + RTP801\_C | GO:0009968 | negative regulation of signal transduction | [B] |
|  | GO:0005737 | cytoplasm | [C] |
| + SAMP | GO:0005515 | protein binding | [M] |
|  | GO:0016055 | Wnt receptor signaling pathway | [B] |
| + SARA |  |  |  |  |
| + SCAN | GO:0003700 | transcription factor activity | [M] |
|  | GO:0006355 | regulation of transcription, DNA-dependent | [B] |
|  | GO:0005634 | nucleus | [C] |
| + SCP-1 | GO:0007130 | synaptonemal complex assembly | [B] |
|  | GO:0000795 | synaptonemal complex | [C] |
| + Secretogranin\_V | GO:0007218 | neuropeptide signaling pathway | [B] |
|  | GO:0030141 | secretory granule | [C] |
| + Smac\_DIABLO | GO:0005515 | protein binding | [M] |
|  | GO:0006917 | induction of apoptosis | [B] |
|  | GO:0006919 | activation of caspase activity | [B] |
|  | GO:0005739 | mitochondrion | [C] |
| + Smoothelin |  |  |  |  |
| + SOCS |  |  |  |  |
| + Sorb |  |  |  |  |
| + Sp38 | GO:0007339 | binding of sperm to zona pellucida | [B] |
|  | GO:0005576 | extracellular region | [C] |
| + Spin-Ssty | GO:0007276 | gamete generation | [B] |
| + STOP |  |  |  |  |
| + Stork\_head |  |  |  |  |
| + Suppressor\_APC |  |  |  |  |
| + Syndecan | GO:0008092 | cytoskeletal protein binding | [M] |
|  | GO:0016020 | membrane | [C] |
| + TF\_Otx | GO:0003700 | transcription factor activity | [M] |
|  | GO:0007275 | multicellular organismal development | [B] |
|  | GO:0005634 | nucleus | [C] |
| + TLV\_coat |  |  |  |  |
| + Tme5\_EGF\_like |  |  |  |  |
| + TPPII\_N |  |  |  |  |
| + TRH | GO:0005184 | neuropeptide hormone activity | [M] |
|  | GO:0009755 | hormone-mediated signaling pathway | [B] |
|  | GO:0005576 | extracellular region | [C] |
| + Troponin |  |  |  |  |
| + Tsg |  |  |  |  |
| + UCR\_6-4kD | GO:0008121 | ubiquinol-cytochrome-c reductase activity | [M] |
|  | GO:0009055 | electron carrier activity | [M] |
|  | GO:0006122 | mitochondrial electron transport, ubiquinol to cytochrome c | [B] |
|  | GO:0031966 | mitochondrial membrane | [C] |
| + UPF0239 | GO:0016021 | integral to membrane | [C] |
| + UPF0560 |  |  |  |  |
| + V-set\_CD47 |  |  |  |  |
| + Vert\_HS\_TF | GO:0003677 | DNA binding | [M] |
|  | GO:0003700 | transcription factor activity | [M] |
|  | GO:0006355 | regulation of transcription, DNA-dependent | [B] |
|  | GO:0005634 | nucleus | [C] |
| + Vg\_Tdu | GO:0030528 | transcription regulator activity | [M] |
|  | GO:0045449 | regulation of transcription | [B] |
|  | GO:0005634 | nucleus | [C] |
| + VGCC\_alpha2 |  |  |  |  |
| + VGCC\_beta4Aa\_N |  |  |  |  |
| + YajC |  |  |  |  |
| + YscO |  |  |  |  |
| + zf-FCS | GO:0008270 | zinc ion binding | [M] |

---

## Bilateria\_Cnidaria

|  |  |  |  |
| --- | --- | --- | --- |
| **Pfam domain(s)** | **GO term acc** | **GO term** | **GO namespace** |
| + 4\_1\_CTD | GO:0003779 | actin binding | [M] |
|  | GO:0005198 | structural molecule activity | [M] |
|  | GO:0005856 | cytoskeleton | [C] |
| + 5-nucleotidase | GO:0000166 | nucleotide binding | [M] |
|  | GO:0000287 | magnesium ion binding | [M] |
|  | GO:0008253 | 5'-nucleotidase activity | [M] |
|  | GO:0009117 | nucleotide metabolic process | [B] |
|  | GO:0005737 | cytoplasm | [C] |
| + 7tm\_7 | GO:0050909 | sensory perception of taste | [B] |
|  | GO:0016021 | integral to membrane | [C] |
| + 7TM\_GPCR\_Srw |  |  |  |  |
| + A1\_Propeptide | GO:0004190 | aspartic-type endopeptidase activity | [M] |
|  | GO:0006508 | proteolysis | [B] |
| + AceK | GO:0008772 | [isocitrate dehydrogenase (NADP+)] kinase activity | [M] |
|  | GO:0016791 | phosphatase activity | [M] |
|  | GO:0006006 | glucose metabolic process | [B] |
|  | GO:0005737 | cytoplasm | [C] |
| + Activin\_recp | GO:0004675 | transmembrane receptor protein serine/threonine kinase activity | [M] |
|  | GO:0005024 | transforming growth factor beta receptor activity | [M] |
|  | GO:0016020 | membrane | [C] |
| + Alpha-2-MRAP\_C | GO:0008201 | heparin binding | [M] |
|  | GO:0050750 | low-density lipoprotein receptor binding | [M] |
|  | GO:0005783 | endoplasmic reticulum | [C] |
| + Alpha-2-MRAP\_N |  |  |  |  |
| + APP\_amyloid |  |  |  |  |
| + ARA70 |  |  |  |  |
| + ATP-synt\_8 | GO:0015078 | hydrogen ion transmembrane transporter activity | [M] |
|  | GO:0015986 | ATP synthesis coupled proton transport | [B] |
|  | GO:0000276 | mitochondrial proton-transporting ATP synthase complex, coupling factor F(o) | [C] |
| + BEN |  |  |  |  |
| + Bile\_Hydr\_Trans | GO:0016290 | palmitoyl-CoA hydrolase activity | [M] |
|  | GO:0006629 | lipid metabolic process | [B] |
| + Biliv-reduc\_cat | GO:0004074 | biliverdin reductase activity | [M] |
|  | GO:0008270 | zinc ion binding | [M] |
|  | GO:0042167 | heme catabolic process | [B] |
|  | GO:0055114 | oxidation reduction | [B] |
| + BNIP3 | GO:0043065 | positive regulation of apoptosis | [B] |
|  | GO:0005740 | mitochondrial envelope | [C] |
|  | GO:0016021 | integral to membrane | [C] |
| + BRICHOS |  |  |  |  |
| + BSMAP |  |  |  |  |
| + Cadherin\_C | GO:0005509 | calcium ion binding | [M] |
|  | GO:0007156 | homophilic cell adhesion | [B] |
|  | GO:0016020 | membrane | [C] |
| + CaKB | GO:0015269 | calcium-activated potassium channel activity | [M] |
|  | GO:0006813 | potassium ion transport | [B] |
|  | GO:0016020 | membrane | [C] |
| + CaM\_bdg\_C0 |  |  |  |  |
| + CDK2AP |  |  |  |  |
| + CDK5\_activator | GO:0016534 | cyclin-dependent protein kinase 5 activator activity | [M] |
|  | GO:0016533 | cyclin-dependent protein kinase 5 activator complex | [C] |
| + Cenp-F\_N |  |  |  |  |
| + CHB\_HEX | GO:0030246 | carbohydrate binding | [M] |
| + CHB\_HEX\_C | GO:0004563 | beta-N-acetylhexosaminidase activity | [M] |
|  | GO:0005975 | carbohydrate metabolic process | [B] |
| + CHDNT | GO:0003677 | DNA binding | [M] |
|  | GO:0005524 | ATP binding | [M] |
|  | GO:0008270 | zinc ion binding | [M] |
|  | GO:0016818 | hydrolase activity, acting on acid anhydrides, in phosphorus-containing anhydrides | [M] |
|  | GO:0045449 | regulation of transcription | [B] |
|  | GO:0005634 | nucleus | [C] |
| + CIDE-N | GO:0005515 | protein binding | [M] |
|  | GO:0006915 | apoptosis | [B] |
|  | GO:0005622 | intracellular | [C] |
| + CITED | GO:0030528 | transcription regulator activity | [M] |
|  | GO:0045449 | regulation of transcription | [B] |
|  | GO:0005634 | nucleus | [C] |
| + Cobl |  |  |  |  |
| + CUT | GO:0003677 | DNA binding | [M] |
| + CXCXC |  |  |  |  |
| + Cys\_knot |  |  |  |  |
| + DED | GO:0005515 | protein binding | [M] |
|  | GO:0042981 | regulation of apoptosis | [B] |
| + DFF-C |  |  |  |  |
| + DFF40 | GO:0016787 | hydrolase activity | [M] |
|  | GO:0006309 | DNA fragmentation involved in apoptosis | [B] |
|  | GO:0005634 | nucleus | [C] |
|  | GO:0005737 | cytoplasm | [C] |
| + DMPK\_coil | GO:0004674 | protein serine/threonine kinase activity | [M] |
|  | GO:0005524 | ATP binding | [M] |
|  | GO:0006468 | protein amino acid phosphorylation | [B] |
| + DUF1113 |  |  |  |  |
| + DUF1151 |  |  |  |  |
| + DUF1280 |  |  |  |  |
| + DUF1387 |  |  |  |  |
| + DUF1866 | GO:0003723 | RNA binding | [M] |
|  | GO:0004439 | phosphatidylinositol-4,5-bisphosphate 5-phosphatase activity | [M] |
|  | GO:0016311 | dephosphorylation | [B] |
| + DUF1879 |  |  |  |  |
| + DUF1891 |  |  |  |  |
| + DUF3105 |  |  |  |  |
| + DUF3338 |  |  |  |  |
| + DUF3340 |  |  |  |  |
| + DUF3399 |  |  |  |  |
| + DUF3498 |  |  |  |  |
| + DUF3524 |  |  |  |  |
| + DUF3538 |  |  |  |  |
| + DUF3588 |  |  |  |  |
| + DUF3590 |  |  |  |  |
| + DUF3695 |  |  |  |  |
| + DUF3697 |  |  |  |  |
| + DUF3715 |  |  |  |  |
| + DUF3733 |  |  |  |  |
| + DUF800 |  |  |  |  |
| + EMI |  |  |  |  |
| + Ephrin | GO:0016020 | membrane | [C] |
| + ETS\_PEA3\_N | GO:0003700 | transcription factor activity | [M] |
|  | GO:0006355 | regulation of transcription, DNA-dependent | [B] |
|  | GO:0005634 | nucleus | [C] |
| + EVC2\_like |  |  |  |  |
| + FA |  |  |  |  |
| + FEZ |  |  |  |  |
| + Flg\_hook | GO:0003774 | motor activity | [M] |
|  | GO:0009296 | flagellum assembly | [B] |
|  | GO:0009424 | bacterial-type flagellum hook | [C] |
| + Focal\_AT | GO:0004713 | protein tyrosine kinase activity | [M] |
|  | GO:0004871 | signal transducer activity | [M] |
|  | GO:0006468 | protein amino acid phosphorylation | [B] |
|  | GO:0007172 | signal complex assembly | [B] |
|  | GO:0005925 | focal adhesion | [C] |
| + GDNF |  |  |  |  |
| + GFP | GO:0008218 | bioluminescence | [B] |
| + GFRP | GO:0005515 | protein binding | [M] |
|  | GO:0009890 | negative regulation of biosynthetic process | [B] |
| + GIT1\_C |  |  |  |  |
| + Glyco\_hydro\_56 | GO:0004415 | hyalurononglucosaminidase activity | [M] |
|  | GO:0005975 | carbohydrate metabolic process | [B] |
| + GumN |  |  |  |  |
| + HARP | GO:0004386 | helicase activity | [M] |
|  | GO:0005524 | ATP binding | [M] |
|  | GO:0016818 | hydrolase activity, acting on acid anhydrides, in phosphorus-containing anhydrides | [M] |
|  | GO:0016568 | chromatin modification | [B] |
|  | GO:0005634 | nucleus | [C] |
| + HEPN |  |  |  |  |
| + HH\_signal | GO:0007267 | cell-cell signaling | [B] |
|  | GO:0007275 | multicellular organismal development | [B] |
| + HJURP\_C |  |  |  |  |
| + HNF\_C |  |  |  |  |
| + Hrs\_helical |  |  |  |  |
| + IER |  |  |  |  |
| + Innexin | GO:0005921 | gap junction | [C] |
| + Insulin | GO:0005179 | hormone activity | [M] |
|  | GO:0005576 | extracellular region | [C] |
| + Integrin\_b\_cyt | GO:0004872 | receptor activity | [M] |
|  | GO:0005488 | binding | [M] |
|  | GO:0007155 | cell adhesion | [B] |
|  | GO:0007160 | cell-matrix adhesion | [B] |
|  | GO:0007229 | integrin-mediated signaling pathway | [B] |
|  | GO:0008305 | integrin complex | [C] |
| + IRF-2BP1\_2 |  |  |  |  |
| + IRF-3 | GO:0003700 | transcription factor activity | [M] |
|  | GO:0006355 | regulation of transcription, DNA-dependent | [B] |
|  | GO:0005634 | nucleus | [C] |
| + Laminin\_II | GO:0007155 | cell adhesion | [B] |
|  | GO:0005604 | basement membrane | [C] |
| + Limkain-b1 |  |  |  |  |
| + Mab-21 |  |  |  |  |
| + MamL-1 | GO:0003713 | transcription coactivator activity | [M] |
|  | GO:0007219 | Notch signaling pathway | [B] |
|  | GO:0045944 | positive regulation of transcription from RNA polymerase II promoter | [B] |
|  | GO:0016607 | nuclear speck | [C] |
| + MAP7 |  |  |  |  |
| + Mitoc\_L55 |  |  |  |  |
| + MRVI1 |  |  |  |  |
| + MtfA |  |  |  |  |
| + NCD1 | GO:0016564 | transcription repressor activity | [M] |
|  | GO:0016481 | negative regulation of transcription | [B] |
|  | GO:0005634 | nucleus | [C] |
| + Nebulin |  |  |  |  |
| + NfI\_DNAbd\_pre-N |  |  |  |  |
| + NHR2 |  |  |  |  |
| + Ninjurin | GO:0007155 | cell adhesion | [B] |
|  | GO:0042246 | tissue regeneration | [B] |
|  | GO:0016021 | integral to membrane | [C] |
| + NNMT\_PNMT\_TEMT | GO:0008168 | methyltransferase activity | [M] |
| + NOD | GO:0030154 | cell differentiation | [B] |
|  | GO:0016021 | integral to membrane | [C] |
| + NODP | GO:0007219 | Notch signaling pathway | [B] |
|  | GO:0007275 | multicellular organismal development | [B] |
|  | GO:0030154 | cell differentiation | [B] |
|  | GO:0016021 | integral to membrane | [C] |
| + Nrf1\_activ\_bdg |  |  |  |  |
| + NtA | GO:0043236 | laminin binding | [M] |
|  | GO:0007213 | muscarinic acetylcholine receptor signaling pathway | [B] |
|  | GO:0043113 | receptor clustering | [B] |
|  | GO:0005605 | basal lamina | [C] |
| + NUC201 |  |  |  |  |
| + OAD\_gamma | GO:0008948 | oxaloacetate decarboxylase activity | [M] |
|  | GO:0015081 | sodium ion transmembrane transporter activity | [M] |
|  | GO:0006814 | sodium ion transport | [B] |
|  | GO:0016020 | membrane | [C] |
| + OAR | GO:0003677 | DNA binding | [M] |
|  | GO:0007275 | multicellular organismal development | [B] |
|  | GO:0005634 | nucleus | [C] |
| + OSR1\_C |  |  |  |  |
| + Otopetrin |  |  |  |  |
| + PDE8 | GO:0004114 | 3',5'-cyclic-nucleotide phosphodiesterase activity | [M] |
|  | GO:0006198 | cAMP catabolic process | [B] |
| + PDGF | GO:0008083 | growth factor activity | [M] |
|  | GO:0016020 | membrane | [C] |
| + PepSY | GO:0008237 | metallopeptidase activity | [M] |
|  | GO:0008270 | zinc ion binding | [M] |
|  | GO:0006508 | proteolysis | [B] |
|  | GO:0005576 | extracellular region | [C] |
| + Pex26 | GO:0005515 | protein binding | [M] |
|  | GO:0045046 | protein import into peroxisome membrane | [B] |
|  | GO:0005779 | integral to peroxisomal membrane | [C] |
| + Phospho\_p8 |  |  |  |  |
| + PIP49\_C |  |  |  |  |
| + PKK |  |  |  |  |
| + PLA2\_inh | GO:0004859 | phospholipase inhibitor activity | [M] |
|  | GO:0005576 | extracellular region | [C] |
| + PLAC | GO:0008233 | peptidase activity | [M] |
| + PmbA\_TldD |  |  |  |  |
| + Preseq\_ALAS | GO:0003870 | 5-aminolevulinate synthase activity | [M] |
|  | GO:0030170 | pyridoxal phosphate binding | [M] |
|  | GO:0006778 | porphyrin metabolic process | [B] |
|  | GO:0005759 | mitochondrial matrix | [C] |
| + RecR | GO:0006281 | DNA repair | [B] |
|  | GO:0006310 | DNA recombination | [B] |
| + Resistin | GO:0005179 | hormone activity | [M] |
|  | GO:0005576 | extracellular region | [C] |
| + RHD | GO:0003700 | transcription factor activity | [M] |
|  | GO:0045449 | regulation of transcription | [B] |
|  | GO:0005634 | nucleus | [C] |
| + Rho\_Binding | GO:0004674 | protein serine/threonine kinase activity | [M] |
|  | GO:0005515 | protein binding | [M] |
|  | GO:0005524 | ATP binding | [M] |
|  | GO:0000910 | cytokinesis | [B] |
|  | GO:0006468 | protein amino acid phosphorylation | [B] |
| + RIG-I\_C-RD |  |  |  |  |
| + RILP |  |  |  |  |
| + SAA | GO:0006953 | acute-phase response | [B] |
|  | GO:0005576 | extracellular region | [C] |
| + Sarcoglycan\_2 | GO:0016012 | sarcoglycan complex | [C] |
| + SCHIP-1 |  |  |  |  |
| + SelP\_N | GO:0008430 | selenium binding | [M] |
| + Sen15 |  |  |  |  |
| + SERTA |  |  |  |  |
| + Shal-type |  |  |  |  |
| + SirA | GO:0005515 | protein binding | [M] |
|  | GO:0016783 | sulfurtransferase activity | [M] |
|  | GO:0008033 | tRNA processing | [B] |
|  | GO:0005737 | cytoplasm | [C] |
| + SOXp |  |  |  |  |
| + Sprouty | GO:0007275 | multicellular organismal development | [B] |
|  | GO:0009966 | regulation of signal transduction | [B] |
|  | GO:0016020 | membrane | [C] |
| + ST7 |  |  |  |  |
| + STAT1\_TAZ2bind |  |  |  |  |
| + stn\_TNFRSF12A |  |  |  |  |
| + SUFU\_C |  |  |  |  |
| + Tap-RNA\_bind |  |  |  |  |
| + TB | GO:0005488 | binding | [M] |
| + Tmemb\_55A |  |  |  |  |
| + TNF | GO:0005164 | tumor necrosis factor receptor binding | [M] |
|  | GO:0006955 | immune response | [B] |
|  | GO:0016020 | membrane | [C] |
| + Tower |  |  |  |  |
| + Transglut\_C | GO:0003810 | protein-glutamine gamma-glutamyltransferase activity | [M] |
|  | GO:0018149 | peptide cross-linking | [B] |
| + Transglut\_N | GO:0018149 | peptide cross-linking | [B] |
| + Trehalose\_recp |  |  |  |  |
| + TRP\_2 |  |  |  |  |
| + UPAR\_LY6 |  |  |  |  |
| + UPF0547 |  |  |  |  |
| + USP8\_interact | GO:0016881 | acid-amino acid ligase activity | [M] |
|  | GO:0031386 | protein tag | [M] |
|  | GO:0016567 | protein ubiquitination | [B] |
| + WIF | GO:0004713 | protein tyrosine kinase activity | [M] |
| + WRW |  |  |  |  |
| + Xlink | GO:0005540 | hyaluronic acid binding | [M] |
|  | GO:0007155 | cell adhesion | [B] |
| + Y\_Y\_Y |  |  |  |  |
| + zf-AD | GO:0008270 | zinc ion binding | [M] |
|  | GO:0005634 | nucleus | [C] |
| + zf-C4\_C |  |  |  |  |

---

## Caenorhabditis [eol|tol]

|  |  |  |  |
| --- | --- | --- | --- |
| **Pfam domain(s)** | **GO term acc** | **GO term** | **GO namespace** |
| + 7TM\_GPCR\_Srb | GO:0004888 | transmembrane receptor activity | [M] |
|  | GO:0007606 | sensory perception of chemical stimulus | [B] |
|  | GO:0016021 | integral to membrane | [C] |
| + 7TM\_GPCR\_Sru |  |  |  |  |
| + 7TM\_GPCR\_Srz |  |  |  |  |
| + CEP1-DNA\_bind |  |  |  |  |
| + CUB\_2 |  |  |  |  |
| + Cytochrom\_B562 | GO:0005506 | iron ion binding | [M] |
|  | GO:0009055 | electron carrier activity | [M] |
|  | GO:0020037 | heme binding | [M] |
|  | GO:0042597 | periplasmic space | [C] |
| + DivIVA |  |  |  |  |
| + DNA\_PPF | GO:0006260 | DNA replication | [B] |
| + DUF1164 |  |  |  |  |
| + DUF1179 |  |  |  |  |
| + DUF1248 |  |  |  |  |
| + DUF1265 |  |  |  |  |
| + DUF1412 |  |  |  |  |
| + DUF1459 |  |  |  |  |
| + DUF1505 |  |  |  |  |
| + DUF2700 |  |  |  |  |
| + DUF2708 |  |  |  |  |
| + DUF272 |  |  |  |  |
| + DUF281 |  |  |  |  |
| + DUF282 |  |  |  |  |
| + DUF3557 |  |  |  |  |
| + DUF508 |  |  |  |  |
| + DUF870 |  |  |  |  |
| + DX |  |  |  |  |
| + EGL-1 |  |  |  |  |
| + ET |  |  |  |  |
| + FBA\_2 |  |  |  |  |
| + Glycoprotein |  |  |  |  |
| + Lin-8 |  |  |  |  |
| + Pes-10 |  |  |  |  |
| + PHA-1 |  |  |  |  |
| + RSD-2 |  |  |  |  |
| + SPK |  |  |  |  |
| + SspO | GO:0030436 | asexual sporulation | [B] |
|  | GO:0042601 | endospore-forming forespore | [C] |
| + TRA-1\_regulated |  |  |  |  |
| + WSN |  |  |  |  |
| + Xol-1\_GHMP-like |  |  |  |  |
| + Xol-1\_N |  |  |  |  |

---

## Chaetomiaceae [eol|tol]

|  |  |  |  |
| --- | --- | --- | --- |
| **Pfam domain(s)** | **GO term acc** | **GO term** | **GO namespace** |

---

## Chlorophyceae [eol|tol]

|  |  |  |  |
| --- | --- | --- | --- |
| **Pfam domain(s)** | **GO term acc** | **GO term** | **GO namespace** |
| + 2Fe-2S\_Ferredox |  |  |  |  |
| + DUF2322 |  |  |  |  |
| + DUF3707 |  |  |  |  |

---

## Chlorophyta [eol|tol]

|  |  |  |  |
| --- | --- | --- | --- |
| **Pfam domain(s)** | **GO term acc** | **GO term** | **GO namespace** |
| + DUF1802 |  |  |  |  |
| + DUF2344 |  |  |  |  |
| + DUF3181 |  |  |  |  |
| + Peptidase\_U9 |  |  |  |  |

---

## Chordata [eol|tol]

|  |  |  |  |
| --- | --- | --- | --- |
| **Pfam domain(s)** | **GO term acc** | **GO term** | **GO namespace** |
| + Calc\_CGRP\_IAPP | GO:0005179 | hormone activity | [M] |
|  | GO:0005576 | extracellular region | [C] |
| + CART | GO:0005515 | protein binding | [M] |
|  | GO:0000186 | activation of MAPKK activity | [B] |
|  | GO:0001678 | cellular glucose homeostasis | [B] |
|  | GO:0007186 | G-protein coupled receptor protein signaling pathway | [B] |
|  | GO:0008343 | adult feeding behavior | [B] |
|  | GO:0009267 | cellular response to starvation | [B] |
|  | GO:0032099 | negative regulation of appetite | [B] |
|  | GO:0005615 | extracellular space | [C] |
| + Colipase | GO:0008047 | enzyme activator activity | [M] |
|  | GO:0007586 | digestion | [B] |
|  | GO:0016042 | lipid catabolic process | [B] |
|  | GO:0005576 | extracellular region | [C] |
| + Colipase\_C | GO:0008047 | enzyme activator activity | [M] |
|  | GO:0007586 | digestion | [B] |
|  | GO:0016042 | lipid catabolic process | [B] |
|  | GO:0005576 | extracellular region | [C] |
| + DUF294 |  |  |  |  |
| + DUF3432 |  |  |  |  |
| + DUF3547 |  |  |  |  |
| + EABR |  |  |  |  |
| + Elf-1\_N |  |  |  |  |
| + EpoR\_lig-bind |  |  |  |  |
| + fn1 | GO:0005576 | extracellular region | [C] |
| + Fox-1\_C |  |  |  |  |
| + Gla | GO:0005509 | calcium ion binding | [M] |
|  | GO:0005576 | extracellular region | [C] |
| + Glyco\_transf\_6 | GO:0016758 | transferase activity, transferring hexosyl groups | [M] |
|  | GO:0005975 | carbohydrate metabolic process | [B] |
|  | GO:0016020 | membrane | [C] |
| + HDAC4\_Gln |  |  |  |  |
| + Hox9\_act | GO:0016563 | transcription activator activity | [M] |
|  | GO:0006350 | transcription | [B] |
|  | GO:0005634 | nucleus | [C] |
| + K167R |  |  |  |  |
| + L27\_N |  |  |  |  |
| + LRRCT |  |  |  |  |
| + LST1 | GO:0000902 | cell morphogenesis | [B] |
|  | GO:0006955 | immune response | [B] |
|  | GO:0016020 | membrane | [C] |
| + Maf\_N |  |  |  |  |
| + MEF2\_binding |  |  |  |  |
| + Mesothelin |  |  |  |  |
| + MYT1 |  |  |  |  |
| + Neuregulin | GO:0005102 | receptor binding | [M] |
|  | GO:0009790 | embryonic development | [B] |
| + Noelin-1 |  |  |  |  |
| + Orexin | GO:0007218 | neuropeptide signaling pathway | [B] |
|  | GO:0007631 | feeding behavior | [B] |
| + p47\_phox\_C |  |  |  |  |
| + PAAD\_DAPIN | GO:0005515 | protein binding | [M] |
| + PAD\_M | GO:0004668 | protein-arginine deiminase activity | [M] |
|  | GO:0005509 | calcium ion binding | [M] |
|  | GO:0018101 | peptidyl-citrulline biosynthetic process from peptidyl-arginine | [B] |
|  | GO:0005737 | cytoplasm | [C] |
| + PAD\_N | GO:0004668 | protein-arginine deiminase activity | [M] |
|  | GO:0005509 | calcium ion binding | [M] |
|  | GO:0005737 | cytoplasm | [C] |
| + Parathyroid | GO:0005179 | hormone activity | [M] |
|  | GO:0005576 | extracellular region | [C] |
| + Pax2\_C |  |  |  |  |
| + RAMP | GO:0008565 | protein transporter activity | [M] |
|  | GO:0006886 | intracellular protein transport | [B] |
|  | GO:0008277 | regulation of G-protein coupled receptor protein signaling pathway | [B] |
|  | GO:0016021 | integral to membrane | [C] |
| + RFX1\_trans\_act | GO:0003677 | DNA binding | [M] |
|  | GO:0030528 | transcription regulator activity | [M] |
|  | GO:0045449 | regulation of transcription | [B] |
|  | GO:0005634 | nucleus | [C] |
| + Rhomboid\_SP |  |  |  |  |
| + RunxI |  |  |  |  |
| + SCF | GO:0005173 | stem cell factor receptor binding | [M] |
|  | GO:0007155 | cell adhesion | [B] |
|  | GO:0016020 | membrane | [C] |
| + Serum\_albumin | GO:0005615 | extracellular space | [C] |
| + Synapsin\_N |  |  |  |  |
| + Ten\_N | GO:0007165 | signal transduction | [B] |
|  | GO:0016021 | integral to membrane | [C] |
| + WT1 | GO:0003700 | transcription factor activity | [M] |
|  | GO:0006355 | regulation of transcription, DNA-dependent | [B] |
|  | GO:0005634 | nucleus | [C] |
| + zf-piccolo | GO:0046872 | metal ion binding | [M] |
|  | GO:0045202 | synapse | [C] |

---

## Chromalveolate [eol|tol]

|  |  |  |  |
| --- | --- | --- | --- |
| **Pfam domain(s)** | **GO term acc** | **GO term** | **GO namespace** |
| + AdoMet\_dc | GO:0004014 | adenosylmethionine decarboxylase activity | [M] |
|  | GO:0008295 | spermidine biosynthetic process | [B] |
| + DUF1244 |  |  |  |  |
| + DUF1501 |  |  |  |  |
| + DUF1800 |  |  |  |  |
| + DUF2086 |  |  |  |  |
| + DUF2087 |  |  |  |  |
| + DUF2997 |  |  |  |  |
| + DUF305 |  |  |  |  |
| + DUF3089 |  |  |  |  |
| + DUF3118 |  |  |  |  |
| + DUF3556 |  |  |  |  |
| + Glutaredoxin2\_C |  |  |  |  |
| + SbcD\_C |  |  |  |  |
| + Ycf34 |  |  |  |  |

---

## Ciliophora [eol|tol]

|  |  |  |  |
| --- | --- | --- | --- |
| **Pfam domain(s)** | **GO term acc** | **GO term** | **GO namespace** |
| + DUF2816 |  |  |  |  |
| + DUF3358 |  |  |  |  |

---

## Coccidia [eol|tol]

|  |  |  |  |
| --- | --- | --- | --- |
| **Pfam domain(s)** | **GO term acc** | **GO term** | **GO namespace** |

---

## Corticata [eol|tol]

|  |  |  |  |
| --- | --- | --- | --- |
| **Pfam domain(s)** | **GO term acc** | **GO term** | **GO namespace** |
| + Aconitase\_2\_N | GO:0003994 | aconitate hydratase activity | [M] |
|  | GO:0005515 | protein binding | [M] |
|  | GO:0006099 | tricarboxylic acid cycle | [B] |
| + Aconitase\_B\_N |  |  |  |  |
| + Alpha-amyl\_C2 | GO:0004556 | alpha-amylase activity | [M] |
|  | GO:0005509 | calcium ion binding | [M] |
|  | GO:0005975 | carbohydrate metabolic process | [B] |
| + Alum\_res |  |  |  |  |
| + Amido\_AtzD\_TrzD |  |  |  |  |
| + AP2 | GO:0003700 | transcription factor activity | [M] |
|  | GO:0006355 | regulation of transcription, DNA-dependent | [B] |
| + Bac\_globin | GO:0019825 | oxygen binding | [M] |
|  | GO:0015671 | oxygen transport | [B] |
| + CbiX | GO:0016829 | lyase activity | [M] |
|  | GO:0046872 | metal ion binding | [M] |
|  | GO:0009236 | cobalamin biosynthetic process | [B] |
| + CBM\_25 |  |  |  |  |
| + CcmH |  |  |  |  |
| + CCT |  |  |  |  |
| + CdCA1 |  |  |  |  |
| + Chloroa\_b-bind | GO:0009765 | photosynthesis, light harvesting | [B] |
|  | GO:0016020 | membrane | [C] |
| + CobA\_CobO\_BtuR | GO:0005524 | ATP binding | [M] |
|  | GO:0008817 | cob(I)yrinic acid a,c-diamide adenosyltransferase activity | [M] |
|  | GO:0009236 | cobalamin biosynthetic process | [B] |
| + CobD\_Cbib | GO:0009236 | cobalamin biosynthetic process | [B] |
|  | GO:0016021 | integral to membrane | [C] |
| + CobS | GO:0008818 | cobalamin 5'-phosphate synthase activity | [M] |
|  | GO:0009236 | cobalamin biosynthetic process | [B] |
| + CP12 |  |  |  |  |
| + CytB6-F\_Fe-S | GO:0009496 | plastoquinol-plastocyanin reductase activity | [M] |
|  | GO:0051537 | 2 iron, 2 sulfur cluster binding | [M] |
|  | GO:0055114 | oxidation reduction | [B] |
|  | GO:0042651 | thylakoid membrane | [C] |
| + DBI\_PRT | GO:0008939 | nicotinate-nucleotide-dimethylbenzimidazole phosphoribosyltransferase activity | [M] |
|  | GO:0009236 | cobalamin biosynthetic process | [B] |
| + Dev\_Cell\_Death |  |  |  |  |
| + DHQS |  |  |  |  |
| + DUF1012 |  |  |  |  |
| + DUF1092 |  |  |  |  |
| + DUF111 |  |  |  |  |
| + DUF1118 |  |  |  |  |
| + DUF1216 |  |  |  |  |
| + DUF1230 |  |  |  |  |
| + DUF1350 |  |  |  |  |
| + DUF1409 |  |  |  |  |
| + DUF1415 |  |  |  |  |
| + DUF1475 |  |  |  |  |
| + DUF1538 |  |  |  |  |
| + DUF1631 |  |  |  |  |
| + DUF1635 |  |  |  |  |
| + DUF1823 |  |  |  |  |
| + DUF1824 |  |  |  |  |
| + DUF1825 |  |  |  |  |
| + DUF1826 |  |  |  |  |
| + DUF1853 |  |  |  |  |
| + DUF192 |  |  |  |  |
| + DUF1967 | GO:0000166 | nucleotide binding | [M] |
| + DUF1995 |  |  |  |  |
| + DUF1997 |  |  |  |  |
| + DUF2061 |  |  |  |  |
| + DUF2062 |  |  |  |  |
| + DUF2088 |  |  |  |  |
| + DUF2214 |  |  |  |  |
| + DUF2256 |  |  |  |  |
| + DUF2499 |  |  |  |  |
| + DUF285 |  |  |  |  |
| + DUF2854 |  |  |  |  |
| + DUF2930 |  |  |  |  |
| + DUF2993 |  |  |  |  |
| + DUF3007 |  |  |  |  |
| + DUF3054 |  |  |  |  |
| + DUF3067 |  |  |  |  |
| + DUF3082 |  |  |  |  |
| + DUF3110 |  |  |  |  |
| + DUF3119 |  |  |  |  |
| + DUF3172 |  |  |  |  |
| + DUF3177 |  |  |  |  |
| + DUF3223 |  |  |  |  |
| + DUF3293 |  |  |  |  |
| + DUF3326 |  |  |  |  |
| + DUF3353 |  |  |  |  |
| + DUF3411 |  |  |  |  |
| + DUF3422 |  |  |  |  |
| + DUF3427 |  |  |  |  |
| + DUF3464 |  |  |  |  |
| + DUF347 |  |  |  |  |
| + DUF3479 |  |  |  |  |
| + DUF3490 |  |  |  |  |
| + DUF3493 |  |  |  |  |
| + DUF3529 |  |  |  |  |
| + DUF358 |  |  |  |  |
| + DUF3593 |  |  |  |  |
| + DUF3611 |  |  |  |  |
| + DUF3633 |  |  |  |  |
| + DUF3656 |  |  |  |  |
| + DUF3727 |  |  |  |  |
| + DUF45 |  |  |  |  |
| + DUF482 |  |  |  |  |
| + DUF486 |  |  |  |  |
| + DUF493 |  |  |  |  |
| + DUF519 |  |  |  |  |
| + DUF565 |  |  |  |  |
| + DUF588 |  |  |  |  |
| + DUF616 |  |  |  |  |
| + DUF633 |  |  |  |  |
| + DUF751 |  |  |  |  |
| + DUF839 |  |  |  |  |
| + DUF98 |  |  |  |  |
| + EB\_dh | GO:0020037 | heme binding | [M] |
|  | GO:0055114 | oxidation reduction | [B] |
| + FAE\_3-kCoA\_syn1 |  |  |  |  |
| + Fe\_bilin\_red | GO:0016636 | oxidoreductase activity, acting on the CH-CH group of donors, iron-sulfur protein as acceptor | [M] |
|  | GO:0050897 | cobalt ion binding | [M] |
|  | GO:0010024 | phytochromobilin biosynthetic process | [B] |
|  | GO:0055114 | oxidation reduction | [B] |
| + Fea1 |  |  |  |  |
| + FeThRed\_A | GO:0008937 | ferredoxin reductase activity | [M] |
|  | GO:0015979 | photosynthesis | [B] |
|  | GO:0009536 | plastid | [C] |
| + FeThRed\_B | GO:0008937 | ferredoxin reductase activity | [M] |
|  | GO:0055114 | oxidation reduction | [B] |
| + FKBP\_N | GO:0006457 | protein folding | [B] |
| + FrhB\_FdhB\_C |  |  |  |  |
| + FrhB\_FdhB\_N |  |  |  |  |
| + GAGA\_bind |  |  |  |  |
| + GlutR\_dimer | GO:0008883 | glutamyl-tRNA reductase activity | [M] |
|  | GO:0050661 | NADP or NADPH binding | [M] |
|  | GO:0033014 | tetrapyrrole biosynthetic process | [B] |
|  | GO:0055114 | oxidation reduction | [B] |
| + Gly\_radical | GO:0003824 | catalytic activity | [M] |
|  | GO:0008152 | metabolic process | [B] |
| + Glycos\_transf\_N | GO:0005529 | sugar binding | [M] |
|  | GO:0016740 | transferase activity | [M] |
|  | GO:0005975 | carbohydrate metabolic process | [B] |
| + GUN4 |  |  |  |  |
| + HA |  |  |  |  |
| + HopJ |  |  |  |  |
| + HpaB | GO:0016712 | oxidoreductase activity, acting on paired donors, with incorporation or reduction of molecular oxygen, reduced flavin or flavoprotein as one donor, and incorporation of one atom of oxygen | [M] |
|  | GO:0010124 | phenylacetate catabolic process | [B] |
| + HrpB\_C |  |  |  |  |
| + IMCp |  |  |  |  |
| + Lin0512\_fam |  |  |  |  |
| + LpxB | GO:0008915 | lipid-A-disaccharide synthase activity | [M] |
|  | GO:0009245 | lipid A biosynthetic process | [B] |
| + LVIVD |  |  |  |  |
| + Lycopene\_cycl | GO:0016705 | oxidoreductase activity, acting on paired donors, with incorporation or reduction of molecular oxygen | [M] |
|  | GO:0016117 | carotenoid biosynthetic process | [B] |
| + MEKHLA |  |  |  |  |
| + Methyltransf\_14 |  |  |  |  |
| + Mg-por\_mtran\_C | GO:0046406 | magnesium protoporphyrin IX methyltransferase activity | [M] |
|  | GO:0015979 | photosynthesis | [B] |
|  | GO:0015995 | chlorophyll biosynthetic process | [B] |
| + MGDG\_synth | GO:0016758 | transferase activity, transferring hexosyl groups | [M] |
|  | GO:0009247 | glycolipid biosynthetic process | [B] |
| + Mlo | GO:0008219 | cell death | [B] |
|  | GO:0016021 | integral to membrane | [C] |
| + MSP | GO:0005509 | calcium ion binding | [M] |
|  | GO:0015979 | photosynthesis | [B] |
|  | GO:0042549 | photosystem II stabilization | [B] |
|  | GO:0009654 | oxygen evolving complex | [C] |
|  | GO:0019898 | extrinsic to membrane | [C] |
| + MttA\_Hcf106 | GO:0008565 | protein transporter activity | [M] |
|  | GO:0015031 | protein transport | [B] |
| + NAF | GO:0007165 | signal transduction | [B] |
| + NusG | GO:0003711 | transcription elongation regulator activity | [M] |
|  | GO:0032968 | positive regulation of RNA elongation from RNA polymerase II promoter | [B] |
| + PAP\_fibrillin | GO:0005198 | structural molecule activity | [M] |
| + Peptidase\_M11 |  |  |  |  |
| + Peptidase\_M3\_N | GO:0008237 | metallopeptidase activity | [M] |
|  | GO:0008270 | zinc ion binding | [M] |
| + Peptidase\_S46 |  |  |  |  |
| + Peptidase\_S7 | GO:0003723 | RNA binding | [M] |
|  | GO:0003724 | RNA helicase activity | [M] |
|  | GO:0005524 | ATP binding | [M] |
| + PilN |  |  |  |  |
| + PPV\_E1\_C | GO:0003677 | DNA binding | [M] |
|  | GO:0004003 | ATP-dependent DNA helicase activity | [M] |
|  | GO:0005524 | ATP binding | [M] |
|  | GO:0006260 | DNA replication | [B] |
| + PQQ\_C | GO:0016614 | oxidoreductase activity, acting on CH-OH group of donors | [M] |
|  | GO:0055114 | oxidation reduction | [B] |
| + PRONE | GO:0005089 | Rho guanyl-nucleotide exchange factor activity | [M] |
| + PsaD | GO:0015979 | photosynthesis | [B] |
|  | GO:0009538 | photosystem I reaction center | [C] |
| + Psb28 | GO:0015979 | photosynthesis | [B] |
|  | GO:0009654 | oxygen evolving complex | [C] |
|  | GO:0016020 | membrane | [C] |
| + PsbM | GO:0019684 | photosynthesis, light reaction | [B] |
|  | GO:0009523 | photosystem II | [C] |
|  | GO:0016021 | integral to membrane | [C] |
| + PsbP | GO:0005509 | calcium ion binding | [M] |
|  | GO:0015979 | photosynthesis | [B] |
|  | GO:0009654 | oxygen evolving complex | [C] |
|  | GO:0019898 | extrinsic to membrane | [C] |
| + PsbQ | GO:0005509 | calcium ion binding | [M] |
|  | GO:0015979 | photosynthesis | [B] |
|  | GO:0009654 | oxygen evolving complex | [C] |
|  | GO:0019898 | extrinsic to membrane | [C] |
| + PsbU | GO:0042549 | photosystem II stabilization | [B] |
|  | GO:0009654 | oxygen evolving complex | [C] |
|  | GO:0019898 | extrinsic to membrane | [C] |
| + PSI\_PsaE | GO:0009538 | photosystem I reaction center | [C] |
| + PSI\_PsaF | GO:0015979 | photosynthesis | [B] |
|  | GO:0009538 | photosystem I reaction center | [C] |
| + PspA\_IM30 |  |  |  |  |
| + PTA\_PTB | GO:0008415 | acyltransferase activity | [M] |
|  | GO:0008152 | metabolic process | [B] |
| + RCC\_reductase |  |  |  |  |
| + Ribosomal\_S30AE | GO:0005488 | binding | [M] |
|  | GO:0044238 | primary metabolic process | [B] |
| + RimM | GO:0006364 | rRNA processing | [B] |
| + rRNA\_methylase |  |  |  |  |
| + Rubredoxin | GO:0009055 | electron carrier activity | [M] |
|  | GO:0046872 | metal ion binding | [M] |
| + SbmA\_BacA | GO:0005215 | transporter activity | [M] |
|  | GO:0006810 | transport | [B] |
|  | GO:0009276 | Gram-negative-bacterium-type cell wall | [C] |
|  | GO:0016021 | integral to membrane | [C] |
| + SdiA-regulated |  |  |  |  |
| + SfsA |  |  |  |  |
| + Sm\_multidrug\_ex |  |  |  |  |
| + Sod\_Ni | GO:0004784 | superoxide dismutase activity | [M] |
|  | GO:0016151 | nickel ion binding | [M] |
|  | GO:0016209 | antioxidant activity | [M] |
| + SPOUT\_MTase | GO:0008168 | methyltransferase activity | [M] |
|  | GO:0006364 | rRNA processing | [B] |
|  | GO:0005737 | cytoplasm | [C] |
| + SpoVS |  |  |  |  |
| + Sulphotransf |  |  |  |  |
| + SURNod19 |  |  |  |  |
| + ThylakoidFormat |  |  |  |  |
| + Tic22 |  |  |  |  |
| + Toprim\_N |  |  |  |  |
| + UPF0060 | GO:0016020 | membrane | [C] |
| + UPF0081 | GO:0000150 | recombinase activity | [M] |
|  | GO:0003677 | DNA binding | [M] |
|  | GO:0004518 | nuclease activity | [M] |
|  | GO:0006281 | DNA repair | [B] |
|  | GO:0006310 | DNA recombination | [B] |
|  | GO:0006974 | response to DNA damage stimulus | [B] |
| + UPF0153 |  |  |  |  |
| + VanY | GO:0008233 | peptidase activity | [M] |
|  | GO:0006508 | proteolysis | [B] |
| + VDE | GO:0046422 | violaxanthin de-epoxidase activity | [M] |
|  | GO:0055114 | oxidation reduction | [B] |
|  | GO:0009507 | chloroplast | [C] |
| + Whirly |  |  |  |  |
| + YGGT | GO:0016020 | membrane | [C] |
| + zf-CHC2 | GO:0003677 | DNA binding | [M] |
|  | GO:0003896 | DNA primase activity | [M] |
|  | GO:0008270 | zinc ion binding | [M] |
|  | GO:0006260 | DNA replication | [B] |

---

## Cryptosporidium [eol|tol]

|  |  |  |  |
| --- | --- | --- | --- |
| **Pfam domain(s)** | **GO term acc** | **GO term** | **GO namespace** |
| + DUF3352 |  |  |  |  |
| + GP40 |  |  |  |  |
| + ScpA\_ScpB |  |  |  |  |

---

## Culicoidea [eol|tol]

|  |  |  |  |
| --- | --- | --- | --- |
| **Pfam domain(s)** | **GO term acc** | **GO term** | **GO namespace** |

---

## Deuterostomia [eol|tol]

|  |  |  |  |
| --- | --- | --- | --- |
| **Pfam domain(s)** | **GO term acc** | **GO term** | **GO namespace** |
| + AChE\_tetra | GO:0004091 | carboxylesterase activity | [M] |
|  | GO:0016020 | membrane | [C] |
| + AKNA |  |  |  |  |
| + Anth\_Ig | GO:0004872 | receptor activity | [M] |
|  | GO:0016021 | integral to membrane | [C] |
| + Caprin-1\_C |  |  |  |  |
| + DUF2028 |  |  |  |  |
| + DUF3401 |  |  |  |  |
| + Lep\_receptor\_Ig |  |  |  |  |
| + NUC202 |  |  |  |  |
| + PEX-2N |  |  |  |  |
| + Rib\_recp\_KP\_reg | GO:0015031 | protein transport | [B] |
|  | GO:0030176 | integral to endoplasmic reticulum membrane | [C] |
| + Tis11B\_N |  |  |  |  |

---

## Diapsida [eol|tol]

|  |  |  |  |
| --- | --- | --- | --- |
| **Pfam domain(s)** | **GO term acc** | **GO term** | **GO namespace** |
| + Apo-VLDL-II | GO:0004857 | enzyme inhibitor activity | [M] |
|  | GO:0006629 | lipid metabolic process | [B] |
|  | GO:0042627 | chylomicron | [C] |
| + Keratin | GO:0005200 | structural constituent of cytoskeleton | [M] |
|  | GO:0005882 | intermediate filament | [C] |

---

## Dictyostelium [eol|tol]

|  |  |  |  |
| --- | --- | --- | --- |
| **Pfam domain(s)** | **GO term acc** | **GO term** | **GO namespace** |
| + Coiled |  |  |  |  |
| + Dict-STAT-coil |  |  |  |  |
| + Dicty\_CTDC |  |  |  |  |
| + Dicty\_REP |  |  |  |  |
| + Dicty\_spore\_N |  |  |  |  |
| + DUF2185 |  |  |  |  |
| + DUF2383 |  |  |  |  |
| + Endotoxin\_C |  |  |  |  |
| + Glyco\_hydro\_57 | GO:0003824 | catalytic activity | [M] |
|  | GO:0005975 | carbohydrate metabolic process | [B] |
| + Inhibitor\_I36 |  |  |  |  |
| + Thy1 | GO:0050660 | FAD binding | [M] |
|  | GO:0050797 | thymidylate synthase (FAD) activity | [M] |
|  | GO:0006231 | dTMP biosynthetic process | [B] |
| + TIR-like |  |  |  |  |

---

## Dikarya [eol|tol]

|  |  |  |  |
| --- | --- | --- | --- |
| **Pfam domain(s)** | **GO term acc** | **GO term** | **GO namespace** |
| + Ad\_cyc\_g-alpha | GO:0000287 | magnesium ion binding | [M] |
|  | GO:0004016 | adenylate cyclase activity | [M] |
|  | GO:0006171 | cAMP biosynthetic process | [B] |
| + AFT |  |  |  |  |
| + Aft1\_HRA |  |  |  |  |
| + BB1 |  |  |  |  |
| + BetaGal\_dom2 |  |  |  |  |
| + CBP4 |  |  |  |  |
| + Cerato-platanin |  |  |  |  |
| + CFEM |  |  |  |  |
| + Chitosanase |  |  |  |  |
| + Choline\_sulf\_C |  |  |  |  |
| + Clr2 |  |  |  |  |
| + Con-6 |  |  |  |  |
| + D-aminoacyl\_C | GO:0008270 | zinc ion binding | [M] |
|  | GO:0016811 | hydrolase activity, acting on carbon-nitrogen (but not peptide) bonds, in linear amides | [M] |
| + DASH\_Dad3 |  |  |  |  |
| + DASH\_Spc34 |  |  |  |  |
| + DHquinase\_II | GO:0003855 | 3-dehydroquinate dehydratase activity | [M] |
| + Dsl1\_C |  |  |  |  |
| + DUF1100 |  |  |  |  |
| + DUF1708 |  |  |  |  |
| + DUF1770 |  |  |  |  |
| + DUF1776 |  |  |  |  |
| + DUF1857 |  |  |  |  |
| + DUF1994 |  |  |  |  |
| + DUF2231 |  |  |  |  |
| + DUF2406 |  |  |  |  |
| + DUF2420 |  |  |  |  |
| + DUF2456 |  |  |  |  |
| + DUF2458 |  |  |  |  |
| + DUF2665 |  |  |  |  |
| + DUF2823 |  |  |  |  |
| + DUF2841 |  |  |  |  |
| + DUF3074 |  |  |  |  |
| + DUF3129 |  |  |  |  |
| + DUF3292 |  |  |  |  |
| + DUF3328 |  |  |  |  |
| + DUF3405 |  |  |  |  |
| + DUF3415 |  |  |  |  |
| + DUF3433 |  |  |  |  |
| + DUF3435 |  |  |  |  |
| + DUF3468 |  |  |  |  |
| + DUF3533 |  |  |  |  |
| + DUF3602 |  |  |  |  |
| + DUF3632 |  |  |  |  |
| + DUF592 | GO:0008270 | zinc ion binding | [M] |
|  | GO:0016811 | hydrolase activity, acting on carbon-nitrogen (but not peptide) bonds, in linear amides | [M] |
|  | GO:0017136 | NAD-dependent histone deacetylase activity | [M] |
|  | GO:0051287 | NAD or NADH binding | [M] |
|  | GO:0006342 | chromatin silencing | [B] |
|  | GO:0006355 | regulation of transcription, DNA-dependent | [B] |
|  | GO:0006476 | protein amino acid deacetylation | [B] |
|  | GO:0045449 | regulation of transcription | [B] |
| + DUF963 |  |  |  |  |
| + EthD |  |  |  |  |
| + FB\_lectin |  |  |  |  |
| + FRQ |  |  |  |  |
| + Glyco\_hydro\_11 | GO:0004553 | hydrolase activity, hydrolyzing O-glycosyl compounds | [M] |
|  | GO:0005975 | carbohydrate metabolic process | [B] |
| + Glyco\_hydro\_62 | GO:0046556 | alpha-N-arabinofuranosidase activity | [M] |
|  | GO:0046373 | L-arabinose metabolic process | [B] |
| + Hap4\_Hap\_bind | GO:0003677 | DNA binding | [M] |
|  | GO:0030528 | transcription regulator activity | [M] |
|  | GO:0006355 | regulation of transcription, DNA-dependent | [B] |
|  | GO:0005634 | nucleus | [C] |
| + Het-C |  |  |  |  |
| + HSP9\_HSP12 | GO:0006950 | response to stress | [B] |
| + Hydrophobin | GO:0005199 | structural constituent of cell wall | [M] |
|  | GO:0009277 | fungal-type cell wall | [C] |
| + ICE2 |  |  |  |  |
| + LIP | GO:0004806 | triglyceride lipase activity | [M] |
|  | GO:0016042 | lipid catabolic process | [B] |
| + Mad3\_BUB1\_II | GO:0000075 | cell cycle checkpoint | [B] |
|  | GO:0005634 | nucleus | [C] |
| + Mitochondr\_Som1 |  |  |  |  |
| + Mtr2 |  |  |  |  |
| + MUG2\_C |  |  |  |  |
| + NUMOD1 |  |  |  |  |
| + NUP | GO:0055085 | transmembrane transport | [B] |
| + Opy2 |  |  |  |  |
| + Pal1 |  |  |  |  |
| + Peptidase\_A4 | GO:0004190 | aspartic-type endopeptidase activity | [M] |
|  | GO:0006508 | proteolysis | [B] |
| + Peptidase\_M35 | GO:0004222 | metalloendopeptidase activity | [M] |
|  | GO:0006508 | proteolysis | [B] |
| + Phe\_hydrox\_dim |  |  |  |  |
| + Phytase | GO:0016158 | 3-phytase activity | [M] |
| + PSDC |  |  |  |  |
| + Rad9\_Rad53\_bind |  |  |  |  |
| + RNR\_inhib |  |  |  |  |
| + Rot1 |  |  |  |  |
| + Rrn6 |  |  |  |  |
| + Sed5p |  |  |  |  |
| + SET\_assoc |  |  |  |  |
| + STE2 | GO:0004932 | mating-type factor pheromone receptor activity | [M] |
|  | GO:0016020 | membrane | [C] |
| + STE3 | GO:0004932 | mating-type factor pheromone receptor activity | [M] |
|  | GO:0007186 | G-protein coupled receptor protein signaling pathway | [B] |
|  | GO:0016021 | integral to membrane | [C] |
| + Tom5 |  |  |  |  |
| + TRI12 |  |  |  |  |
| + TRI5 | GO:0045482 | trichodiene synthase activity | [M] |
|  | GO:0016106 | sesquiterpenoid biosynthetic process | [B] |
| + Trp\_DMAT |  |  |  |  |
| + UBA\_3 |  |  |  |  |

---

## Dikarya\_Mucoromycotina

|  |  |  |  |
| --- | --- | --- | --- |
| **Pfam domain(s)** | **GO term acc** | **GO term** | **GO namespace** |
| + Abp2 |  |  |  |  |
| + Aft1\_OSA |  |  |  |  |
| + Alginate\_lyase | GO:0045135 | poly(beta-D-mannuronate) lyase activity | [M] |
|  | GO:0042122 | alginic acid catabolic process | [B] |
|  | GO:0042597 | periplasmic space | [C] |
| + Arb1 |  |  |  |  |
| + ATP-synt\_J | GO:0015078 | hydrogen ion transmembrane transporter activity | [M] |
|  | GO:0015986 | ATP synthesis coupled proton transport | [B] |
|  | GO:0045263 | proton-transporting ATP synthase complex, coupling factor F(o) | [C] |
| + ATP\_sub\_h |  |  |  |  |
| + CBM\_19 | GO:0004568 | chitinase activity | [M] |
|  | GO:0006032 | chitin catabolic process | [B] |
| + CDC24 |  |  |  |  |
| + CPT | GO:0005524 | ATP binding | [M] |
|  | GO:0016740 | transferase activity | [M] |
| + CRC\_subunit |  |  |  |  |
| + CTK3\_C |  |  |  |  |
| + DASH\_Duo1 |  |  |  |  |
| + DASH\_Spc19 |  |  |  |  |
| + Dfp1\_Him1\_M |  |  |  |  |
| + DUF1687 | GO:0016491 | oxidoreductase activity | [M] |
|  | GO:0055114 | oxidation reduction | [B] |
|  | GO:0005739 | mitochondrion | [C] |
| + DUF1691 |  |  |  |  |
| + DUF1709 |  |  |  |  |
| + DUF1720 |  |  |  |  |
| + DUF1746 |  |  |  |  |
| + DUF1763 |  |  |  |  |
| + DUF2015 |  |  |  |  |
| + DUF2416 |  |  |  |  |
| + DUF2421 |  |  |  |  |
| + DUF2422 |  |  |  |  |
| + DUF2427 |  |  |  |  |
| + DUF2433 |  |  |  |  |
| + DUF2461 |  |  |  |  |
| + DUF2611 |  |  |  |  |
| + DUF3020 |  |  |  |  |
| + DUF3425 |  |  |  |  |
| + DUF3659 |  |  |  |  |
| + DUF3722 |  |  |  |  |
| + DUF3759 |  |  |  |  |
| + DUF3779 |  |  |  |  |
| + eIF\_4G1 |  |  |  |  |
| + EOS1 |  |  |  |  |
| + FLILHELTA |  |  |  |  |
| + Fmp27\_SW |  |  |  |  |
| + Fungal\_trans | GO:0003677 | DNA binding | [M] |
|  | GO:0008270 | zinc ion binding | [M] |
|  | GO:0006350 | transcription | [B] |
|  | GO:0005634 | nucleus | [C] |
| + Inhibitor\_I78 |  |  |  |  |
| + INO80\_Ies4 |  |  |  |  |
| + Ish1 |  |  |  |  |
| + KAR9 |  |  |  |  |
| + Kei1 |  |  |  |  |
| + Lyase\_8\_C | GO:0016829 | lyase activity | [M] |
|  | GO:0005576 | extracellular region | [C] |
| + Med5 | GO:0016455 | RNA polymerase II transcription mediator activity | [M] |
|  | GO:0006357 | regulation of transcription from RNA polymerase II promoter | [B] |
|  | GO:0016592 | mediator complex | [C] |
| + Mit\_ribos\_Mrp51 |  |  |  |  |
| + Pik1 |  |  |  |  |
| + QCR10 |  |  |  |  |
| + RXT2\_N |  |  |  |  |
| + SHD1 |  |  |  |  |
| + SHR3\_chaperone |  |  |  |  |
| + SOG2 |  |  |  |  |
| + Spo7 |  |  |  |  |
| + SUR7 |  |  |  |  |
| + Svf1 | GO:0006979 | response to oxidative stress | [B] |
| + TCO89 |  |  |  |  |
| + Tht1 |  |  |  |  |
| + Tim54 |  |  |  |  |
| + TOM13 | GO:0006810 | transport | [B] |
|  | GO:0005741 | mitochondrial outer membrane | [C] |
| + Trehalase\_Ca-bi | GO:0004555 | alpha,alpha-trehalase activity | [M] |
|  | GO:0005509 | calcium ion binding | [M] |
|  | GO:0005993 | trehalose catabolic process | [B] |
|  | GO:0005737 | cytoplasm | [C] |
| + Utp8 |  |  |  |  |
| + Velvet |  |  |  |  |
| + Whi5 |  |  |  |  |
| + Ydc2-catalyt |  |  |  |  |
| + Ytp1 |  |  |  |  |
| + Zds\_C |  |  |  |  |

---

## Diptera [eol|tol]

|  |  |  |  |
| --- | --- | --- | --- |
| **Pfam domain(s)** | **GO term acc** | **GO term** | **GO namespace** |
| + DUF1091 |  |  |  |  |
| + DUF1213 |  |  |  |  |
| + DUF1986 |  |  |  |  |
| + DUF725 |  |  |  |  |
| + Gypsy |  |  |  |  |
| + Metallothio\_5 | GO:0046872 | metal ion binding | [M] |

---

## Dothideomycetes [eol|tol]

|  |  |  |  |
| --- | --- | --- | --- |
| **Pfam domain(s)** | **GO term acc** | **GO term** | **GO namespace** |

---

## Ecdysozoa [eol|tol]

|  |  |  |  |
| --- | --- | --- | --- |
| **Pfam domain(s)** | **GO term acc** | **GO term** | **GO namespace** |
| + Chorion\_1 | GO:0005213 | structural constituent of chorion | [M] |
|  | GO:0007275 | multicellular organismal development | [B] |
|  | GO:0007304 | chorion-containing eggshell formation | [B] |
|  | GO:0042600 | chorion | [C] |
| + Chorion\_2 | GO:0007275 | multicellular organismal development | [B] |
|  | GO:0042600 | chorion | [C] |
| + Crust\_neurohorm | GO:0005184 | neuropeptide hormone activity | [M] |
|  | GO:0005576 | extracellular region | [C] |
| + Cuticle\_3 |  |  |  |  |
| + DUF1136 |  |  |  |  |
| + GcrA |  |  |  |  |
| + Hydrolase\_2 | GO:0016787 | hydrolase activity | [M] |
|  | GO:0009847 | spore germination | [B] |
|  | GO:0005618 | cell wall | [C] |
| + Rhabdo\_ncap | GO:0019013 | viral nucleocapsid | [C] |

---

## Embryophyta [eol|tol]

|  |  |  |  |
| --- | --- | --- | --- |
| **Pfam domain(s)** | **GO term acc** | **GO term** | **GO namespace** |
| + AdoMetDC\_leader |  |  |  |  |
| + Amidase\_3 | GO:0008745 | N-acetylmuramoyl-L-alanine amidase activity | [M] |
|  | GO:0009253 | peptidoglycan catabolic process | [B] |
| + AraC\_binding | GO:0006355 | regulation of transcription, DNA-dependent | [B] |
| + AUX\_IAA | GO:0045449 | regulation of transcription | [B] |
|  | GO:0005634 | nucleus | [C] |
| + Auxin\_inducible |  |  |  |  |
| + AvrRpt-cleavage |  |  |  |  |
| + AWPM-19 |  |  |  |  |
| + Bet\_v\_1 |  |  |  |  |
| + BURP |  |  |  |  |
| + bZIP\_C |  |  |  |  |
| + CaM\_binding |  |  |  |  |
| + CcmB | GO:0015232 | heme transporter activity | [M] |
|  | GO:0015886 | heme transport | [B] |
|  | GO:0017004 | cytochrome complex assembly | [B] |
|  | GO:0016020 | membrane | [C] |
| + CCT\_2 |  |  |  |  |
| + COBRA |  |  |  |  |
| + Cryptochrome\_C |  |  |  |  |
| + DELLA |  |  |  |  |
| + Dirigent |  |  |  |  |
| + DsbB | GO:0015035 | protein disulfide oxidoreductase activity | [M] |
|  | GO:0016020 | membrane | [C] |
| + DUF1005 |  |  |  |  |
| + DUF1068 |  |  |  |  |
| + DUF1218 |  |  |  |  |
| + DUF1423 |  |  |  |  |
| + DUF1442 |  |  |  |  |
| + DUF1639 |  |  |  |  |
| + DUF1644 |  |  |  |  |
| + DUF1645 |  |  |  |  |
| + DUF1675 |  |  |  |  |
| + DUF1677 |  |  |  |  |
| + DUF1817 |  |  |  |  |
| + DUF220 |  |  |  |  |
| + DUF2294 |  |  |  |  |
| + DUF247 |  |  |  |  |
| + DUF248 |  |  |  |  |
| + DUF260 |  |  |  |  |
| + DUF2921 |  |  |  |  |
| + DUF2996 |  |  |  |  |
| + DUF309 |  |  |  |  |
| + DUF3143 |  |  |  |  |
| + DUF3148 |  |  |  |  |
| + DUF3252 |  |  |  |  |
| + DUF3339 |  |  |  |  |
| + DUF3420 |  |  |  |  |
| + DUF3444 |  |  |  |  |
| + DUF3475 |  |  |  |  |
| + DUF3511 |  |  |  |  |
| + DUF3571 |  |  |  |  |
| + DUF3700 |  |  |  |  |
| + DUF3741 |  |  |  |  |
| + DUF538 |  |  |  |  |
| + DUF568 |  |  |  |  |
| + DUF569 |  |  |  |  |
| + DUF593 |  |  |  |  |
| + DUF617 |  |  |  |  |
| + DUF623 |  |  |  |  |
| + DUF627 |  |  |  |  |
| + DUF630 |  |  |  |  |
| + DUF632 |  |  |  |  |
| + DUF668 |  |  |  |  |
| + DUF674 |  |  |  |  |
| + DUF688 |  |  |  |  |
| + DUF761 |  |  |  |  |
| + DUF793 |  |  |  |  |
| + DUF794 |  |  |  |  |
| + DUF822 |  |  |  |  |
| + DUF868 |  |  |  |  |
| + DUF869 |  |  |  |  |
| + DUF936 |  |  |  |  |
| + DUF966 |  |  |  |  |
| + DVL |  |  |  |  |
| + ENOD93 |  |  |  |  |
| + FBA\_3 |  |  |  |  |
| + FLO\_LFY | GO:0003677 | DNA binding | [M] |
|  | GO:0045449 | regulation of transcription | [B] |
| + Frigida |  |  |  |  |
| + Glyco\_transf\_9 | GO:0016740 | transferase activity | [M] |
|  | GO:0008152 | metabolic process | [B] |
| + GRAS |  |  |  |  |
| + HisKA\_3 | GO:0000155 | two-component sensor activity | [M] |
|  | GO:0046983 | protein dimerization activity | [M] |
|  | GO:0000160 | two-component signal transduction system (phosphorelay) | [B] |
|  | GO:0016021 | integral to membrane | [C] |
| + Hs1pro-1\_C |  |  |  |  |
| + Hs1pro-1\_N |  |  |  |  |
| + Invertase\_neut |  |  |  |  |
| + KNOX2 | GO:0003677 | DNA binding | [M] |
|  | GO:0005634 | nucleus | [C] |
| + MASE1 |  |  |  |  |
| + NABP |  |  |  |  |
| + NADPH\_Ox | GO:0004601 | peroxidase activity | [M] |
|  | GO:0050664 | oxidoreductase activity, acting on NADH or NADPH, with oxygen as acceptor | [M] |
|  | GO:0055114 | oxidation reduction | [B] |
| + NAM | GO:0003677 | DNA binding | [M] |
|  | GO:0045449 | regulation of transcription | [B] |
| + NdhL | GO:0016655 | oxidoreductase activity, acting on NADH or NADPH, quinone or similar compound as acceptor | [M] |
|  | GO:0055114 | oxidation reduction | [B] |
| + NdhM |  |  |  |  |
| + NdhN |  |  |  |  |
| + NdhO |  |  |  |  |
| + NOZZLE |  |  |  |  |
| + NPR1\_like\_C |  |  |  |  |
| + Oleosin | GO:0012511 | monolayer-surrounded lipid storage body | [C] |
|  | GO:0016021 | integral to membrane | [C] |
| + PAR1 |  |  |  |  |
| + PH\_2 |  |  |  |  |
| + Plant\_zn\_clust |  |  |  |  |
| + PMEI | GO:0004857 | enzyme inhibitor activity | [M] |
|  | GO:0030599 | pectinesterase activity | [M] |
| + Pollen\_Ole\_e\_I |  |  |  |  |
| + PORR |  |  |  |  |
| + POX |  |  |  |  |
| + PPO1\_DWL |  |  |  |  |
| + PPO1\_KFDV |  |  |  |  |
| + REF |  |  |  |  |
| + Remorin\_C |  |  |  |  |
| + Rhamnogal\_lyase |  |  |  |  |
| + Root\_cap |  |  |  |  |
| + RST |  |  |  |  |
| + S1FA | GO:0003677 | DNA binding | [M] |
|  | GO:0045449 | regulation of transcription | [B] |
|  | GO:0005634 | nucleus | [C] |
| + S6PP\_C | GO:0000287 | magnesium ion binding | [M] |
|  | GO:0050307 | sucrose-phosphatase activity | [M] |
|  | GO:0005986 | sucrose biosynthetic process | [B] |
| + S\_locus\_glycop | GO:0048544 | recognition of pollen | [B] |
| + SecB | GO:0051082 | unfolded protein binding | [M] |
|  | GO:0015031 | protein transport | [B] |
|  | GO:0051262 | protein tetramerization | [B] |
| + Terpene\_synth | GO:0016829 | lyase activity | [M] |
|  | GO:0008152 | metabolic process | [B] |
| + tify |  |  |  |  |
| + tRNA\_deacylase |  |  |  |  |
| + Tryp\_alpha\_amyl |  |  |  |  |
| + VQ |  |  |  |  |
| + WCOR413 |  |  |  |  |
| + WI12 |  |  |  |  |
| + XET\_C | GO:0016762 | xyloglucan:xyloglucosyl transferase activity | [M] |
|  | GO:0006073 | cellular glucan metabolic process | [B] |
|  | GO:0005618 | cell wall | [C] |
|  | GO:0048046 | apoplast | [C] |
| + XH |  |  |  |  |
| + XS |  |  |  |  |
| + ZF-HD\_dimer |  |  |  |  |

---

## Euarchontoglires [eol|tol]

|  |  |  |  |
| --- | --- | --- | --- |
| **Pfam domain(s)** | **GO term acc** | **GO term** | **GO namespace** |
| + Adhes-Ig\_like | GO:0007155 | cell adhesion | [B] |
|  | GO:0016020 | membrane | [C] |
| + Defensin\_1 | GO:0006952 | defense response | [B] |
|  | GO:0005576 | extracellular region | [C] |
| + P120R |  |  |  |  |
| + PTP\_N |  |  |  |  |
| + Sarcolipin | GO:0030234 | enzyme regulator activity | [M] |
|  | GO:0016020 | membrane | [C] |
| + SelP\_C | GO:0008430 | selenium binding | [M] |
| + Semenogelin | GO:0005198 | structural molecule activity | [M] |
|  | GO:0019953 | sexual reproduction | [B] |
|  | GO:0005576 | extracellular region | [C] |
|  | GO:0030141 | secretory granule | [C] |
| + SNURF |  |  |  |  |

---

## Eukaryota [eol|tol]

|  |  |  |  |
| --- | --- | --- | --- |
| **Pfam domain(s)** | **GO term acc** | **GO term** | **GO namespace** |

---

## Eurotiales [eol|tol]

|  |  |  |  |
| --- | --- | --- | --- |
| **Pfam domain(s)** | **GO term acc** | **GO term** | **GO namespace** |
| + Antifungal\_pept |  |  |  |  |
| + AreA\_N | GO:0003677 | DNA binding | [M] |
|  | GO:0008270 | zinc ion binding | [M] |
|  | GO:0016563 | transcription activator activity | [M] |
|  | GO:0042128 | nitrate assimilation | [B] |
|  | GO:0005634 | nucleus | [C] |
| + DUF2990 |  |  |  |  |
| + MAAL\_C |  |  |  |  |
| + MAAL\_N |  |  |  |  |
| + WXG100 |  |  |  |  |

---

## Euteleostei [eol|tol]

|  |  |  |  |
| --- | --- | --- | --- |
| **Pfam domain(s)** | **GO term acc** | **GO term** | **GO namespace** |

---

## Eutheria [eol|tol]

|  |  |  |  |
| --- | --- | --- | --- |
| **Pfam domain(s)** | **GO term acc** | **GO term** | **GO namespace** |
| + ApoA-II | GO:0008289 | lipid binding | [M] |
|  | GO:0006869 | lipid transport | [B] |
|  | GO:0042157 | lipoprotein metabolic process | [B] |
|  | GO:0005576 | extracellular region | [C] |
| + bcl-2I13 |  |  |  |  |
| + BEX |  |  |  |  |
| + CAP18\_C |  |  |  |  |
| + Casein | GO:0005215 | transporter activity | [M] |
|  | GO:0006810 | transport | [B] |
|  | GO:0005576 | extracellular region | [C] |
| + Casein\_kappa | GO:0005576 | extracellular region | [C] |
| + CD4-extracel |  |  |  |  |
| + CD45 |  |  |  |  |
| + Doppel |  |  |  |  |
| + DUF1220 |  |  |  |  |
| + DUF2476 |  |  |  |  |
| + DUF2477 |  |  |  |  |
| + DUF3631 |  |  |  |  |
| + Feld-I\_B |  |  |  |  |
| + Filaggrin | GO:0005198 | structural molecule activity | [M] |
| + GAGE |  |  |  |  |
| + H-K\_ATPase\_N | GO:0000287 | magnesium ion binding | [M] |
|  | GO:0005524 | ATP binding | [M] |
|  | GO:0008900 | hydrogen:potassium-exchanging ATPase activity | [M] |
|  | GO:0015991 | ATP hydrolysis coupled proton transport | [B] |
|  | GO:0016020 | membrane | [C] |
| + Hist\_rich\_Ca-bd |  |  |  |  |
| + HJURP\_mid |  |  |  |  |
| + IL13 |  |  |  |  |
| + IL2 | GO:0005134 | interleukin-2 receptor binding | [M] |
|  | GO:0008083 | growth factor activity | [M] |
|  | GO:0006955 | immune response | [B] |
|  | GO:0005576 | extracellular region | [C] |
| + IL3 | GO:0005135 | interleukin-3 receptor binding | [M] |
|  | GO:0008083 | growth factor activity | [M] |
|  | GO:0006955 | immune response | [B] |
|  | GO:0005576 | extracellular region | [C] |
| + Involucrin\_N |  |  |  |  |
| + KRTAP |  |  |  |  |
| + LAP2alpha |  |  |  |  |
| + Macscav\_rec | GO:0005044 | scavenger receptor activity | [M] |
|  | GO:0006898 | receptor-mediated endocytosis | [B] |
|  | GO:0016020 | membrane | [C] |
| + MAGE\_N |  |  |  |  |
| + MAP1B\_neuraxin |  |  |  |  |
| + MHC\_I\_C | GO:0006955 | immune response | [B] |
|  | GO:0019882 | antigen processing and presentation | [B] |
|  | GO:0016020 | membrane | [C] |
|  | GO:0042612 | MHC class I protein complex | [C] |
| + Osteoregulin |  |  |  |  |
| + P19Arf\_N |  |  |  |  |
| + ProSAAS |  |  |  |  |
| + Protamine\_P2 | GO:0003677 | DNA binding | [M] |
|  | GO:0007283 | spermatogenesis | [B] |
|  | GO:0000786 | nucleosome | [C] |
|  | GO:0005634 | nucleus | [C] |
| + RDM |  |  |  |  |
| + SLAM | GO:0004872 | receptor activity | [M] |
|  | GO:0046649 | lymphocyte activation | [B] |
|  | GO:0009986 | cell surface | [C] |
|  | GO:0016021 | integral to membrane | [C] |
| + Sperm\_Ag\_HE2 | GO:0005576 | extracellular region | [C] |
| + SSXRD | GO:0003676 | nucleic acid binding | [M] |
|  | GO:0006355 | regulation of transcription, DNA-dependent | [B] |
|  | GO:0005634 | nucleus | [C] |
| + STAT2\_C |  |  |  |  |
| + TEP1\_N |  |  |  |  |
| + TP1 | GO:0003677 | DNA binding | [M] |
|  | GO:0007283 | spermatogenesis | [B] |
|  | GO:0000786 | nucleosome | [C] |
|  | GO:0005634 | nucleus | [C] |
| + TP2 | GO:0003677 | DNA binding | [M] |
|  | GO:0007283 | spermatogenesis | [B] |
|  | GO:0000786 | nucleosome | [C] |
|  | GO:0005634 | nucleus | [C] |

---

## Excavata [eol|tol]

|  |  |  |  |
| --- | --- | --- | --- |
| **Pfam domain(s)** | **GO term acc** | **GO term** | **GO namespace** |
| + ScdA\_N |  |  |  |  |

---

## Fungi [eol|tol]

|  |  |  |  |
| --- | --- | --- | --- |
| **Pfam domain(s)** | **GO term acc** | **GO term** | **GO namespace** |
| + Chs3p |  |  |  |  |
| + Gti1\_Pac2 |  |  |  |  |
| + STE | GO:0003700 | transcription factor activity | [M] |
|  | GO:0006355 | regulation of transcription, DNA-dependent | [B] |
|  | GO:0005634 | nucleus | [C] |

---

## Heterokonta [eol|tol]

|  |  |  |  |
| --- | --- | --- | --- |
| **Pfam domain(s)** | **GO term acc** | **GO term** | **GO namespace** |
| + DUF1696 |  |  |  |  |
| + DUF1974 | GO:0003995 | acyl-CoA dehydrogenase activity | [M] |
|  | GO:0033539 | fatty acid beta-oxidation using acyl-CoA dehydrogenase | [B] |
|  | GO:0055114 | oxidation reduction | [B] |

---

## Heterokonta\_Alveolata

|  |  |  |  |
| --- | --- | --- | --- |
| **Pfam domain(s)** | **GO term acc** | **GO term** | **GO namespace** |
| + Beta\_propel |  |  |  |  |
| + NUMOD4 | GO:0016788 | hydrolase activity, acting on ester bonds | [M] |
| + Paramecium\_SA |  |  |  |  |

---

## Homobasidiomycetes [eol|tol]

|  |  |  |  |
| --- | --- | --- | --- |
| **Pfam domain(s)** | **GO term acc** | **GO term** | **GO namespace** |

---

## Hymenoptera [eol|tol]

|  |  |  |  |
| --- | --- | --- | --- |
| **Pfam domain(s)** | **GO term acc** | **GO term** | **GO namespace** |
| + Apis\_Csd |  |  |  |  |

---

## Hypocreales [eol|tol]

|  |  |  |  |
| --- | --- | --- | --- |
| **Pfam domain(s)** | **GO term acc** | **GO term** | **GO namespace** |
| + Antibiotic\_NAT | GO:0046353 | aminoglycoside 3-N-acetyltransferase activity | [M] |
|  | GO:0046677 | response to antibiotic | [B] |
| + HET-s\_218-289 |  |  |  |  |
| + TcdB\_toxin\_midC |  |  |  |  |

---

## Insecta [eol|tol]

|  |  |  |  |
| --- | --- | --- | --- |
| **Pfam domain(s)** | **GO term acc** | **GO term** | **GO namespace** |
| + Abdominal-A |  |  |  |  |
| + ApoLp-III | GO:0008289 | lipid binding | [M] |
|  | GO:0006869 | lipid transport | [B] |
|  | GO:0005576 | extracellular region | [C] |
| + Argos |  |  |  |  |
| + Attacin\_C | GO:0005576 | extracellular region | [C] |
| + DSX\_dimer |  |  |  |  |
| + DUF1074 |  |  |  |  |
| + DUF1431 |  |  |  |  |
| + DUF1676 |  |  |  |  |
| + DUF243 |  |  |  |  |
| + Ins\_allergen\_rp |  |  |  |  |
| + Optomotor-blind |  |  |  |  |
| + PBAN | GO:0005184 | neuropeptide hormone activity | [M] |
|  | GO:0007218 | neuropeptide signaling pathway | [B] |
|  | GO:0042811 | pheromone biosynthetic process | [B] |
| + PBP\_GOBP | GO:0005549 | odorant binding | [M] |
|  | GO:0006810 | transport | [B] |
| + PRE\_C2HC |  |  |  |  |
| + Retinin\_C |  |  |  |  |
| + Serendipity\_A | GO:0007349 | cellularization | [B] |
|  | GO:0005737 | cytoplasm | [C] |
|  | GO:0016020 | membrane | [C] |

---

## Kinetoplastida [eol|tol]

|  |  |  |  |
| --- | --- | --- | --- |
| **Pfam domain(s)** | **GO term acc** | **GO term** | **GO namespace** |
| + CesT | GO:0009405 | pathogenesis | [B] |
|  | GO:0050708 | regulation of protein secretion | [B] |
|  | GO:0005737 | cytoplasm | [C] |
| + DUF1796 |  |  |  |  |
| + DUF1930 |  |  |  |  |
| + DUF1935 |  |  |  |  |
| + DUF3648 |  |  |  |  |
| + DUF523 |  |  |  |  |
| + dUTPase\_2 |  |  |  |  |
| + Flagellar\_rod | GO:0005516 | calmodulin binding | [M] |
|  | GO:0009434 | microtubule-based flagellum | [C] |
| + KMP11 | GO:0006952 | defense response | [B] |
|  | GO:0008284 | positive regulation of cell proliferation | [B] |

---

## Kinetoplastida\_Heterolobosea

|  |  |  |  |
| --- | --- | --- | --- |
| **Pfam domain(s)** | **GO term acc** | **GO term** | **GO namespace** |
| + DUF3592 |  |  |  |  |

---

## Lepidoptera\_Diptera

|  |  |  |  |
| --- | --- | --- | --- |
| **Pfam domain(s)** | **GO term acc** | **GO term** | **GO namespace** |
| + Attacin\_N | GO:0005576 | extracellular region | [C] |
| + Cecropin | GO:0005576 | extracellular region | [C] |
| + HyaE |  |  |  |  |

---

## Lepidoptera\_Diptera\_Hymenoptera

|  |  |  |  |
| --- | --- | --- | --- |
| **Pfam domain(s)** | **GO term acc** | **GO term** | **GO namespace** |
| + DUF3610 |  |  |  |  |
| + Methuselah\_N | GO:0004930 | G-protein coupled receptor activity | [M] |
|  | GO:0006950 | response to stress | [B] |
| + PHAT |  |  |  |  |

---

## Lophotrochozoa [eol|tol]

|  |  |  |  |
| --- | --- | --- | --- |
| **Pfam domain(s)** | **GO term acc** | **GO term** | **GO namespace** |

---

## Magnaporthales\_Hypocreales

|  |  |  |  |
| --- | --- | --- | --- |
| **Pfam domain(s)** | **GO term acc** | **GO term** | **GO namespace** |
| + Cad |  |  |  |  |

---

## Magnoliophyta [eol|tol]

|  |  |  |  |
| --- | --- | --- | --- |
| **Pfam domain(s)** | **GO term acc** | **GO term** | **GO namespace** |
| + Acyl-thio\_N |  |  |  |  |
| + ATHILA |  |  |  |  |
| + Bowman-Birk\_leg | GO:0004867 | serine-type endopeptidase inhibitor activity | [M] |
|  | GO:0005576 | extracellular region | [C] |
| + DREPP |  |  |  |  |
| + DUF1082 | GO:0016820 | hydrolase activity, acting on acid anhydrides, catalyzing transmembrane movement of substances | [M] |
|  | GO:0005739 | mitochondrion | [C] |
|  | GO:0016021 | integral to membrane | [C] |
| + DUF1117 |  |  |  |  |
| + DUF1195 |  |  |  |  |
| + DUF1221 |  |  |  |  |
| + DUF1262 |  |  |  |  |
| + DUF1278 |  |  |  |  |
| + DUF1666 |  |  |  |  |
| + DUF1950 |  |  |  |  |
| + DUF241 |  |  |  |  |
| + DUF2647 |  |  |  |  |
| + DUF2667 |  |  |  |  |
| + DUF295 |  |  |  |  |
| + DUF313 |  |  |  |  |
| + DUF3357 |  |  |  |  |
| + DUF3403 |  |  |  |  |
| + DUF3615 |  |  |  |  |
| + DUF3660 |  |  |  |  |
| + DUF3774 |  |  |  |  |
| + DUF594 |  |  |  |  |
| + DUF642 |  |  |  |  |
| + FBA\_1 |  |  |  |  |
| + FBD |  |  |  |  |
| + HCMV\_UL139 |  |  |  |  |
| + HD-ZIP\_N | GO:0016563 | transcription activator activity | [M] |
|  | GO:0006350 | transcription | [B] |
|  | GO:0005634 | nucleus | [C] |
| + Kunitz\_legume | GO:0004866 | endopeptidase inhibitor activity | [M] |
| + LEA\_6 |  |  |  |  |
| + LRR\_2 |  |  |  |  |
| + Metallothio\_2 | GO:0046872 | metal ion binding | [M] |
| + MP | GO:0003676 | nucleic acid binding | [M] |
|  | GO:0006810 | transport | [B] |
| + P\_C |  |  |  |  |
| + Pec\_lyase\_N | GO:0030570 | pectate lyase activity | [M] |
| + PMD |  |  |  |  |
| + PSK | GO:0008083 | growth factor activity | [M] |
|  | GO:0008283 | cell proliferation | [B] |
|  | GO:0005576 | extracellular region | [C] |
| + RbcS |  |  |  |  |
| + Remorin\_N |  |  |  |  |
| + Synthase\_beta |  |  |  |  |
| + Thionin | GO:0006952 | defense response | [B] |
| + TSP9 |  |  |  |  |
| + WaaY | GO:0009244 | lipopolysaccharide core region biosynthetic process | [B] |
| + WAK | GO:0004674 | protein serine/threonine kinase activity | [M] |
|  | GO:0016021 | integral to membrane | [C] |
| + WIYLD |  |  |  |  |

---

## Mammalia [eol|tol]

|  |  |  |  |
| --- | --- | --- | --- |
| **Pfam domain(s)** | **GO term acc** | **GO term** | **GO namespace** |
| + AHSP | GO:0030492 | hemoglobin binding | [M] |
|  | GO:0006457 | protein folding | [B] |
|  | GO:0020027 | hemoglobin metabolic process | [B] |
|  | GO:0030097 | hemopoiesis | [B] |
|  | GO:0050821 | protein stabilization | [B] |
| + BCMA-Tall\_bind |  |  |  |  |
| + Bim\_N |  |  |  |  |
| + CReP\_N |  |  |  |  |
| + CSF-1 | GO:0005125 | cytokine activity | [M] |
|  | GO:0008083 | growth factor activity | [M] |
|  | GO:0016021 | integral to membrane | [C] |
| + Defensin\_3 | GO:0005576 | extracellular region | [C] |
| + DMP1 | GO:0001503 | ossification | [B] |
|  | GO:0030198 | extracellular matrix organization | [B] |
| + DUF1968 |  |  |  |  |
| + IFNGR1 | GO:0019955 | cytokine binding | [M] |
|  | GO:0016020 | membrane | [C] |
| + Keratin\_matx | GO:0005198 | structural molecule activity | [M] |
|  | GO:0045095 | keratin filament | [C] |
| + Microcephalin |  |  |  |  |
| + Neurokinin\_B | GO:0007217 | tachykinin receptor signaling pathway | [B] |
| + RII\_binding\_1 |  |  |  |  |
| + SPO11\_like | GO:0003677 | DNA binding | [M] |
|  | GO:0007131 | reciprocal meiotic recombination | [B] |
| + SVA | GO:0005576 | extracellular region | [C] |

---

## Metamonada [eol|tol]

|  |  |  |  |
| --- | --- | --- | --- |
| **Pfam domain(s)** | **GO term acc** | **GO term** | **GO namespace** |

---

## Metazoa [eol|tol]

|  |  |  |  |
| --- | --- | --- | --- |
| **Pfam domain(s)** | **GO term acc** | **GO term** | **GO namespace** |
| + 53-BP1\_Tudor |  |  |  |  |
| + A4\_EXTRA | GO:0005488 | binding | [M] |
|  | GO:0016021 | integral to membrane | [C] |
| + AA\_permease\_N |  |  |  |  |
| + Abi\_HHR | GO:0005737 | cytoplasm | [C] |
| + ADAM\_CR | GO:0004222 | metalloendopeptidase activity | [M] |
|  | GO:0006508 | proteolysis | [B] |
| + ADAM\_spacer1 | GO:0004222 | metalloendopeptidase activity | [M] |
|  | GO:0031012 | extracellular matrix | [C] |
| + AF-4 |  |  |  |  |
| + ATP-synt\_F6 | GO:0015078 | hydrogen ion transmembrane transporter activity | [M] |
|  | GO:0015986 | ATP synthesis coupled proton transport | [B] |
|  | GO:0000276 | mitochondrial proton-transporting ATP synthase complex, coupling factor F(o) | [C] |
| + Avidin |  |  |  |  |
| + AXH | GO:0005488 | binding | [M] |
| + Bcl-2 | GO:0042981 | regulation of apoptosis | [B] |
| + BCL\_N |  |  |  |  |
| + Beta-TrCP\_D |  |  |  |  |
| + BNIP2 |  |  |  |  |
| + BRCA-2\_OB3 |  |  |  |  |
| + c-SKI\_SMAD\_bind | GO:0005515 | protein binding | [M] |
|  | GO:0005634 | nucleus | [C] |
| + C4 | GO:0005201 | extracellular matrix structural constituent | [M] |
|  | GO:0005581 | collagen | [C] |
| + C5-epim\_C | GO:0016857 | racemase and epimerase activity, acting on carbohydrates and derivatives | [M] |
|  | GO:0006024 | glycosaminoglycan biosynthetic process | [B] |
|  | GO:0016021 | integral to membrane | [C] |
| + C8 |  |  |  |  |
| + Ca\_chan\_IQ |  |  |  |  |
| + CALCOCO1 |  |  |  |  |
| + Calponin |  |  |  |  |
| + CARD | GO:0005515 | protein binding | [M] |
|  | GO:0042981 | regulation of apoptosis | [B] |
|  | GO:0005622 | intracellular | [C] |
| + Casc1 |  |  |  |  |
| + Caveolin |  |  |  |  |
| + CBF\_beta | GO:0003713 | transcription coactivator activity | [M] |
|  | GO:0005634 | nucleus | [C] |
| + CD20 | GO:0004872 | receptor activity | [M] |
|  | GO:0007165 | signal transduction | [B] |
|  | GO:0016021 | integral to membrane | [C] |
| + CD34\_antigen |  |  |  |  |
| + Churchill | GO:0008270 | zinc ion binding | [M] |
|  | GO:0016563 | transcription activator activity | [M] |
|  | GO:0007275 | multicellular organismal development | [B] |
|  | GO:0045941 | positive regulation of transcription | [B] |
| + CLCA\_N |  |  |  |  |
| + Cobalamin\_bind | GO:0031419 | cobalamin binding | [M] |
|  | GO:0015889 | cobalamin transport | [B] |
| + Cor1 |  |  |  |  |
| + DAN |  |  |  |  |
| + DAZAP2 |  |  |  |  |
| + Death | GO:0005515 | protein binding | [M] |
|  | GO:0007165 | signal transduction | [B] |
| + Defensin\_propep | GO:0006952 | defense response | [B] |
| + DM | GO:0003677 | DNA binding | [M] |
|  | GO:0003700 | transcription factor activity | [M] |
|  | GO:0006355 | regulation of transcription, DNA-dependent | [B] |
|  | GO:0007548 | sex differentiation | [B] |
|  | GO:0005634 | nucleus | [C] |
| + DMA |  |  |  |  |
| + DUF1041 |  |  |  |  |
| + DUF1053 | GO:0004016 | adenylate cyclase activity | [M] |
|  | GO:0009190 | cyclic nucleotide biosynthetic process | [B] |
|  | GO:0016021 | integral to membrane | [C] |
| + DUF1075 |  |  |  |  |
| + DUF1135 | GO:0007096 | regulation of exit from mitosis | [B] |
|  | GO:0005634 | nucleus | [C] |
| + DUF1154 | GO:0004435 | phosphoinositide phospholipase C activity | [M] |
|  | GO:0005509 | calcium ion binding | [M] |
|  | GO:0006629 | lipid metabolic process | [B] |
| + DUF1872 | GO:0004221 | ubiquitin thiolesterase activity | [M] |
|  | GO:0008270 | zinc ion binding | [M] |
|  | GO:0043161 | proteasomal ubiquitin-dependent protein catabolic process | [B] |
| + DUF1897 |  |  |  |  |
| + DUF1908 | GO:0000287 | magnesium ion binding | [M] |
|  | GO:0004674 | protein serine/threonine kinase activity | [M] |
|  | GO:0005524 | ATP binding | [M] |
|  | GO:0006468 | protein amino acid phosphorylation | [B] |
| + DUF1916 |  |  |  |  |
| + DUF1943 | GO:0005319 | lipid transporter activity | [M] |
|  | GO:0006869 | lipid transport | [B] |
| + DUF1973 |  |  |  |  |
| + DUF2216 |  |  |  |  |
| + DUF2353 |  |  |  |  |
| + DUF2650 |  |  |  |  |
| + DUF3028 |  |  |  |  |
| + DUF3350 |  |  |  |  |
| + DUF3480 |  |  |  |  |
| + DUF3497 |  |  |  |  |
| + DUF3504 |  |  |  |  |
| + DUF3512 |  |  |  |  |
| + DUF3513 |  |  |  |  |
| + DUF3518 |  |  |  |  |
| + DUF3534 |  |  |  |  |
| + DUF3585 |  |  |  |  |
| + DUF3652 |  |  |  |  |
| + DUF719 |  |  |  |  |
| + DUF766 |  |  |  |  |
| + DZF |  |  |  |  |
| + E3\_UbLigase\_EDD |  |  |  |  |
| + Ephrin\_lbd | GO:0005003 | ephrin receptor activity | [M] |
|  | GO:0005524 | ATP binding | [M] |
|  | GO:0016020 | membrane | [C] |
| + Ets | GO:0003700 | transcription factor activity | [M] |
|  | GO:0043565 | sequence-specific DNA binding | [M] |
|  | GO:0006355 | regulation of transcription, DNA-dependent | [B] |
| + EURL |  |  |  |  |
| + Exon\_PolB |  |  |  |  |
| + FAIM1 | GO:0043066 | negative regulation of apoptosis | [B] |
| + FAST\_2 |  |  |  |  |
| + FerA | GO:0016021 | integral to membrane | [C] |
| + FerB | GO:0016021 | integral to membrane | [C] |
| + Gasdermin |  |  |  |  |
| + Gate | GO:0001882 | nucleoside binding | [M] |
| + Geminin | GO:0008156 | negative regulation of DNA replication | [B] |
| + Glypican | GO:0043395 | heparan sulfate proteoglycan binding | [M] |
|  | GO:0005578 | proteinaceous extracellular matrix | [C] |
|  | GO:0016020 | membrane | [C] |
| + Hairy\_orange | GO:0003677 | DNA binding | [M] |
|  | GO:0006355 | regulation of transcription, DNA-dependent | [B] |
| + HDOD |  |  |  |  |
| + HNF-1\_N | GO:0016563 | transcription activator activity | [M] |
|  | GO:0045941 | positive regulation of transcription | [B] |
|  | GO:0005634 | nucleus | [C] |
| + Hormone\_recep | GO:0003700 | transcription factor activity | [M] |
|  | GO:0003707 | steroid hormone receptor activity | [M] |
|  | GO:0006355 | regulation of transcription, DNA-dependent | [B] |
|  | GO:0005634 | nucleus | [C] |
| + HRM | GO:0004930 | G-protein coupled receptor activity | [M] |
|  | GO:0016020 | membrane | [C] |
| + HSNSD |  |  |  |  |
| + Hyd\_WA |  |  |  |  |
| + ICA69 |  |  |  |  |
| + ig |  |  |  |  |
| + IGFBP | GO:0005520 | insulin-like growth factor binding | [M] |
|  | GO:0001558 | regulation of cell growth | [B] |
|  | GO:0005576 | extracellular region | [C] |
| + Integrin\_alpha2 |  |  |  |  |
| + Integrin\_B\_tail | GO:0004872 | receptor activity | [M] |
|  | GO:0005488 | binding | [M] |
|  | GO:0007155 | cell adhesion | [B] |
|  | GO:0007160 | cell-matrix adhesion | [B] |
|  | GO:0007229 | integrin-mediated signaling pathway | [B] |
|  | GO:0008305 | integrin complex | [C] |
| + Integrin\_beta | GO:0004872 | receptor activity | [M] |
|  | GO:0005488 | binding | [M] |
|  | GO:0007155 | cell adhesion | [B] |
|  | GO:0007160 | cell-matrix adhesion | [B] |
|  | GO:0007229 | integrin-mediated signaling pathway | [B] |
|  | GO:0008305 | integrin complex | [C] |
| + IRF | GO:0003700 | transcription factor activity | [M] |
|  | GO:0006355 | regulation of transcription, DNA-dependent | [B] |
| + Jnk-SapK\_ap\_N |  |  |  |  |
| + Jun |  |  |  |  |
| + L27 |  |  |  |  |
| + L27\_2 |  |  |  |  |
| + Laminin\_B | GO:0007155 | cell adhesion | [B] |
|  | GO:0031012 | extracellular matrix | [C] |
| + Laminin\_G\_1 |  |  |  |  |
| + Lamp | GO:0016020 | membrane | [C] |
| + LAP1C |  |  |  |  |
| + Ldl\_recept\_b | GO:0016020 | membrane | [C] |
| + LEM | GO:0005635 | nuclear envelope | [C] |
| + LLGL |  |  |  |  |
| + Macoilin | GO:0016021 | integral to membrane | [C] |
| + MANEC |  |  |  |  |
| + MCC-bdg\_PDZ |  |  |  |  |
| + MCLC |  |  |  |  |
| + Med25 |  |  |  |  |
| + Med30 |  |  |  |  |
| + Metallothio | GO:0046872 | metal ion binding | [M] |
| + MH1 | GO:0006355 | regulation of transcription, DNA-dependent | [B] |
|  | GO:0005622 | intracellular | [C] |
| + MNNL | GO:0007219 | Notch signaling pathway | [B] |
|  | GO:0007275 | multicellular organismal development | [B] |
|  | GO:0016021 | integral to membrane | [C] |
| + MRP-S32 |  |  |  |  |
| + Mtp | GO:0006810 | transport | [B] |
|  | GO:0016021 | integral to membrane | [C] |
| + Myc\_N | GO:0003700 | transcription factor activity | [M] |
|  | GO:0006355 | regulation of transcription, DNA-dependent | [B] |
|  | GO:0005634 | nucleus | [C] |
| + Na\_K-ATPase | GO:0005391 | sodium:potassium-exchanging ATPase activity | [M] |
|  | GO:0006754 | ATP biosynthetic process | [B] |
|  | GO:0006813 | potassium ion transport | [B] |
|  | GO:0006814 | sodium ion transport | [B] |
|  | GO:0016020 | membrane | [C] |
| + Nbs1\_C |  |  |  |  |
| + NCD3G | GO:0004930 | G-protein coupled receptor activity | [M] |
|  | GO:0007186 | G-protein coupled receptor protein signaling pathway | [B] |
| + Neil1-DNA\_bind |  |  |  |  |
| + Neuralized |  |  |  |  |
| + NIDO | GO:0007160 | cell-matrix adhesion | [B] |
| + Noggin | GO:0045596 | negative regulation of cell differentiation | [B] |
| + NOPS |  |  |  |  |
| + NPDC1 | GO:0016021 | integral to membrane | [C] |
| + Nrf1\_DNA-bind |  |  |  |  |
| + NTR |  |  |  |  |
| + OCIA |  |  |  |  |
| + Ocular\_alb | GO:0016020 | membrane | [C] |
| + P53\_tetramer | GO:0003700 | transcription factor activity | [M] |
|  | GO:0008270 | zinc ion binding | [M] |
|  | GO:0002347 | response to tumor cell | [B] |
|  | GO:0030308 | negative regulation of cell growth | [B] |
| + PBC | GO:0003700 | transcription factor activity | [M] |
|  | GO:0005634 | nucleus | [C] |
| + PBP5\_C | GO:0009002 | serine-type D-Ala-D-Ala carboxypeptidase activity | [M] |
|  | GO:0006508 | proteolysis | [B] |
| + PCAF\_N | GO:0004402 | histone acetyltransferase activity | [M] |
|  | GO:0006355 | regulation of transcription, DNA-dependent | [B] |
|  | GO:0005634 | nucleus | [C] |
| + PDCD9 | GO:0003735 | structural constituent of ribosome | [M] |
|  | GO:0006412 | translation | [B] |
|  | GO:0005739 | mitochondrion | [C] |
|  | GO:0005840 | ribosome | [C] |
| + Peptidase\_M2 | GO:0008237 | metallopeptidase activity | [M] |
|  | GO:0008241 | peptidyl-dipeptidase activity | [M] |
|  | GO:0006508 | proteolysis | [B] |
|  | GO:0016020 | membrane | [C] |
| + Pinin\_SDK\_N |  |  |  |  |
| + pKID | GO:0005515 | protein binding | [M] |
|  | GO:0006355 | regulation of transcription, DNA-dependent | [B] |
| + PLC-beta\_C | GO:0004435 | phosphoinositide phospholipase C activity | [M] |
|  | GO:0005509 | calcium ion binding | [M] |
|  | GO:0016042 | lipid catabolic process | [B] |
| + Pou | GO:0003700 | transcription factor activity | [M] |
|  | GO:0006355 | regulation of transcription, DNA-dependent | [B] |
| + PP1\_inhibitor | GO:0005515 | protein binding | [M] |
|  | GO:0042325 | regulation of phosphorylation | [B] |
|  | GO:0005737 | cytoplasm | [C] |
| + PP1c\_bdg |  |  |  |  |
| + PP2C\_C | GO:0000287 | magnesium ion binding | [M] |
|  | GO:0004721 | phosphoprotein phosphatase activity | [M] |
|  | GO:0030145 | manganese ion binding | [M] |
| + RanGAP1\_C | GO:0005098 | Ran GTPase activator activity | [M] |
|  | GO:0005515 | protein binding | [M] |
|  | GO:0007165 | signal transduction | [B] |
| + RBB1NT |  |  |  |  |
| + RGM\_C |  |  |  |  |
| + RGM\_N |  |  |  |  |
| + RGS-like |  |  |  |  |
| + Rib\_hydrolayse | GO:0003953 | NAD+ nucleosidase activity | [M] |
| + RPH3A\_effect\_N |  |  |  |  |
| + Runt | GO:0003677 | DNA binding | [M] |
|  | GO:0003700 | transcription factor activity | [M] |
|  | GO:0006355 | regulation of transcription, DNA-dependent | [B] |
| + SapA |  |  |  |  |
| + Sclerostin |  |  |  |  |
| + SEA |  |  |  |  |
| + SEFIR |  |  |  |  |
| + Sema |  |  |  |  |
| + Serine\_rich |  |  |  |  |
| + Ski\_Sno | GO:0005634 | nucleus | [C] |
| + Snurportin1 |  |  |  |  |
| + SOCS\_box | GO:0007242 | intracellular signaling cascade | [B] |
| + Sox\_N |  |  |  |  |
| + Spot\_14 |  |  |  |  |
| + STAT\_alpha | GO:0003700 | transcription factor activity | [M] |
|  | GO:0004871 | signal transducer activity | [M] |
|  | GO:0006355 | regulation of transcription, DNA-dependent | [B] |
|  | GO:0007165 | signal transduction | [B] |
|  | GO:0005634 | nucleus | [C] |
| + STAT\_int | GO:0003700 | transcription factor activity | [M] |
|  | GO:0004871 | signal transducer activity | [M] |
|  | GO:0006355 | regulation of transcription, DNA-dependent | [B] |
|  | GO:0007165 | signal transduction | [B] |
| + Stathmin | GO:0007242 | intracellular signaling cascade | [B] |
| + Strabismus | GO:0007275 | multicellular organismal development | [B] |
|  | GO:0016021 | integral to membrane | [C] |
| + T-box | GO:0003700 | transcription factor activity | [M] |
|  | GO:0006355 | regulation of transcription, DNA-dependent | [B] |
|  | GO:0005634 | nucleus | [C] |
| + TAFH | GO:0003700 | transcription factor activity | [M] |
|  | GO:0006355 | regulation of transcription, DNA-dependent | [B] |
|  | GO:0005634 | nucleus | [C] |
| + TF\_AP-2 |  |  |  |  |
| + TGF\_beta | GO:0008083 | growth factor activity | [M] |
| + TGF\_beta\_GS | GO:0004675 | transmembrane receptor protein serine/threonine kinase activity | [M] |
|  | GO:0005524 | ATP binding | [M] |
|  | GO:0006468 | protein amino acid phosphorylation | [B] |
|  | GO:0016020 | membrane | [C] |
| + TGFb\_propeptide | GO:0008083 | growth factor activity | [M] |
|  | GO:0040007 | growth | [B] |
| + THAP | GO:0003676 | nucleic acid binding | [M] |
| + Thyroglobulin\_1 |  |  |  |  |
| + TIMP | GO:0008191 | metalloendopeptidase inhibitor activity | [M] |
|  | GO:0005578 | proteinaceous extracellular matrix | [C] |
| + Tissue\_fac | GO:0007596 | blood coagulation | [B] |
|  | GO:0016021 | integral to membrane | [C] |
| + TLE\_N |  |  |  |  |
| + Tmem26 |  |  |  |  |
| + Tmemb\_18A |  |  |  |  |
| + Tmemb\_cc2 |  |  |  |  |
| + TPD52 |  |  |  |  |
| + TraG | GO:0009291 | unidirectional conjugation | [B] |
|  | GO:0016020 | membrane | [C] |
| + Tropomodulin | GO:0005523 | tropomyosin binding | [M] |
|  | GO:0005856 | cytoskeleton | [C] |
| + TSP\_C | GO:0005509 | calcium ion binding | [M] |
|  | GO:0007155 | cell adhesion | [B] |
|  | GO:0005576 | extracellular region | [C] |
| + Vitellogenin\_N | GO:0005319 | lipid transporter activity | [M] |
|  | GO:0006869 | lipid transport | [B] |
| + VWA\_N |  |  |  |  |
| + VWC |  |  |  |  |
| + wnt | GO:0004871 | signal transducer activity | [M] |
|  | GO:0007223 | Wnt receptor signaling pathway, calcium modulating pathway | [B] |
|  | GO:0007275 | multicellular organismal development | [B] |
|  | GO:0005576 | extracellular region | [C] |
| + Xylo\_C |  |  |  |  |
| + zf-C4 | GO:0003700 | transcription factor activity | [M] |
|  | GO:0008270 | zinc ion binding | [M] |
|  | GO:0043565 | sequence-specific DNA binding | [M] |
|  | GO:0006355 | regulation of transcription, DNA-dependent | [B] |
|  | GO:0005634 | nucleus | [C] |
| + zf-CpG\_bind\_C |  |  |  |  |
| + zf-nanos | GO:0003723 | RNA binding | [M] |
|  | GO:0008270 | zinc ion binding | [M] |
| + Zona\_pellucida |  |  |  |  |
| + ZU5 |  |  |  |  |

---

## Metazoa\_Choanoflagellata

|  |  |  |  |
| --- | --- | --- | --- |
| **Pfam domain(s)** | **GO term acc** | **GO term** | **GO namespace** |
| + ANKH | GO:0015114 | phosphate transmembrane transporter activity | [M] |
|  | GO:0006817 | phosphate transport | [B] |
|  | GO:0016021 | integral to membrane | [C] |
| + Arfaptin |  |  |  |  |
| + BLVR |  |  |  |  |
| + C1q |  |  |  |  |
| + Cbl\_N | GO:0004871 | signal transducer activity | [M] |
|  | GO:0007166 | cell surface receptor linked signal transduction | [B] |
|  | GO:0005634 | nucleus | [C] |
| + Cbl\_N3 | GO:0004871 | signal transducer activity | [M] |
|  | GO:0007166 | cell surface receptor linked signal transduction | [B] |
|  | GO:0005634 | nucleus | [C] |
| + CI-B14\_5a | GO:0008137 | NADH dehydrogenase (ubiquinone) activity | [M] |
|  | GO:0042773 | ATP synthesis coupled electron transport | [B] |
|  | GO:0005743 | mitochondrial inner membrane | [C] |
| + COLFI | GO:0005201 | extracellular matrix structural constituent | [M] |
|  | GO:0005581 | collagen | [C] |
| + Cul7 |  |  |  |  |
| + Dsh\_C |  |  |  |  |
| + DUF1011 |  |  |  |  |
| + DUF1180 |  |  |  |  |
| + DUF1211 |  |  |  |  |
| + DUF2152 |  |  |  |  |
| + DUF2217 |  |  |  |  |
| + DUF2352 |  |  |  |  |
| + DUF2366 |  |  |  |  |
| + DUF3398 |  |  |  |  |
| + DUF3462 |  |  |  |  |
| + DUF3668 |  |  |  |  |
| + DUF3719 |  |  |  |  |
| + DUF902 |  |  |  |  |
| + efhand\_1 |  |  |  |  |
| + efhand\_2 |  |  |  |  |
| + ERM | GO:0008092 | cytoskeletal protein binding | [M] |
|  | GO:0005737 | cytoplasm | [C] |
|  | GO:0019898 | extrinsic to membrane | [C] |
| + Furin-like | GO:0004714 | transmembrane receptor protein tyrosine kinase activity | [M] |
|  | GO:0005524 | ATP binding | [M] |
|  | GO:0006468 | protein amino acid phosphorylation | [B] |
|  | GO:0007169 | transmembrane receptor protein tyrosine kinase signaling pathway | [B] |
|  | GO:0016020 | membrane | [C] |
| + Fzo\_mitofusin | GO:0003924 | GTPase activity | [M] |
|  | GO:0008053 | mitochondrial fusion | [B] |
|  | GO:0005741 | mitochondrial outer membrane | [C] |
|  | GO:0016021 | integral to membrane | [C] |
| + GKAP | GO:0007267 | cell-cell signaling | [B] |
| + Glyco\_hydro\_59 | GO:0004336 | galactosylceramidase activity | [M] |
|  | GO:0006683 | galactosylceramide catabolic process | [B] |
| + GoLoco | GO:0005096 | GTPase activator activity | [M] |
|  | GO:0007165 | signal transduction | [B] |
| + HS1\_rep |  |  |  |  |
| + HSL\_N | GO:0016298 | lipase activity | [M] |
|  | GO:0008203 | cholesterol metabolic process | [B] |
|  | GO:0016042 | lipid catabolic process | [B] |
| + IRS | GO:0005158 | insulin receptor binding | [M] |
| + L27\_1 |  |  |  |  |
| + Laminin\_N | GO:0005578 | proteinaceous extracellular matrix | [C] |
| + MH2 | GO:0006355 | regulation of transcription, DNA-dependent | [B] |
|  | GO:0005622 | intracellular | [C] |
| + MRP-S22 |  |  |  |  |
| + MRP-S35 |  |  |  |  |
| + Mst1\_SARAH |  |  |  |  |
| + OSTMP1 |  |  |  |  |
| + P4Ha\_N | GO:0004656 | procollagen-proline 4-dioxygenase activity | [M] |
|  | GO:0016702 | oxidoreductase activity, acting on single donors with incorporation of molecular oxygen, incorporation of two atoms of oxygen | [M] |
|  | GO:0055114 | oxidation reduction | [B] |
|  | GO:0005783 | endoplasmic reticulum | [C] |
| + P53 | GO:0003677 | DNA binding | [M] |
|  | GO:0003700 | transcription factor activity | [M] |
|  | GO:0006355 | regulation of transcription, DNA-dependent | [B] |
|  | GO:0005634 | nucleus | [C] |
| + PET | GO:0008270 | zinc ion binding | [M] |
| + PI3K\_p85B | GO:0046934 | phosphatidylinositol-4,5-bisphosphate 3-kinase activity | [M] |
|  | GO:0007165 | signal transduction | [B] |
|  | GO:0005942 | phosphoinositide 3-kinase complex | [C] |
| + PID |  |  |  |  |
| + Plexin\_cytopl |  |  |  |  |
| + PMP22\_Claudin | GO:0016020 | membrane | [C] |
| + PngaseF\_C |  |  |  |  |
| + Rapsyn\_N | GO:0008270 | zinc ion binding | [M] |
|  | GO:0033130 | acetylcholine receptor binding | [M] |
|  | GO:0007268 | synaptic transmission | [B] |
|  | GO:0005856 | cytoskeleton | [C] |
|  | GO:0030054 | cell junction | [C] |
|  | GO:0045211 | postsynaptic membrane | [C] |
| + RBD | GO:0005057 | receptor signaling protein activity | [M] |
|  | GO:0007165 | signal transduction | [B] |
| + RBD-FIP | GO:0015031 | protein transport | [B] |
|  | GO:0043231 | intracellular membrane-bounded organelle | [C] |
| + Sarcoglycan\_1 | GO:0007010 | cytoskeleton organization | [B] |
|  | GO:0016012 | sarcoglycan complex | [C] |
|  | GO:0016021 | integral to membrane | [C] |
| + STAT\_bind | GO:0003700 | transcription factor activity | [M] |
|  | GO:0004871 | signal transducer activity | [M] |
|  | GO:0006355 | regulation of transcription, DNA-dependent | [B] |
|  | GO:0007165 | signal transduction | [B] |
|  | GO:0005634 | nucleus | [C] |
| + Synaphin | GO:0019905 | syntaxin binding | [M] |
|  | GO:0006836 | neurotransmitter transport | [B] |
| + Talin\_middle | GO:0005200 | structural constituent of cytoskeleton | [M] |
|  | GO:0005515 | protein binding | [M] |
|  | GO:0007016 | cytoskeletal anchoring at plasma membrane | [B] |
|  | GO:0001726 | ruffle | [C] |
|  | GO:0005925 | focal adhesion | [C] |
| + Tmemb\_9 | GO:0016021 | integral to membrane | [C] |
| + Tmpp129 |  |  |  |  |
| + TRAP-delta | GO:0005783 | endoplasmic reticulum | [C] |
|  | GO:0016021 | integral to membrane | [C] |
| + UPF0184 |  |  |  |  |
| + VASP |  |  |  |  |
| + zf-C2HC | GO:0003700 | transcription factor activity | [M] |
|  | GO:0008270 | zinc ion binding | [M] |
|  | GO:0006355 | regulation of transcription, DNA-dependent | [B] |
|  | GO:0005634 | nucleus | [C] |

---

## Micromonas [eol|tol]

|  |  |  |  |
| --- | --- | --- | --- |
| **Pfam domain(s)** | **GO term acc** | **GO term** | **GO namespace** |
| + DUF3516 |  |  |  |  |

---

## Mucoromycotina [eol|tol]

|  |  |  |  |
| --- | --- | --- | --- |
| **Pfam domain(s)** | **GO term acc** | **GO term** | **GO namespace** |
| + DUF1775 |  |  |  |  |

---

## Muscomorpha [eol|tol]

|  |  |  |  |
| --- | --- | --- | --- |
| **Pfam domain(s)** | **GO term acc** | **GO term** | **GO namespace** |
| + ACP53EA |  |  |  |  |
| + Antimicrobial10 |  |  |  |  |
| + Chorion\_3 | GO:0007275 | multicellular organismal development | [B] |
|  | GO:0042600 | chorion | [C] |
| + Chorion\_S16 | GO:0007275 | multicellular organismal development | [B] |
|  | GO:0042600 | chorion | [C] |
| + DEC-1\_C | GO:0005213 | structural constituent of chorion | [M] |
|  | GO:0007304 | chorion-containing eggshell formation | [B] |
|  | GO:0005576 | extracellular region | [C] |
|  | GO:0042600 | chorion | [C] |
| + DEC-1\_N | GO:0005213 | structural constituent of chorion | [M] |
|  | GO:0007304 | chorion-containing eggshell formation | [B] |
|  | GO:0005576 | extracellular region | [C] |
|  | GO:0042600 | chorion | [C] |
| + DIM |  |  |  |  |
| + DUF2967 |  |  |  |  |
| + DUF733 |  |  |  |  |
| + FTZ |  |  |  |  |
| + GYR |  |  |  |  |
| + L71 |  |  |  |  |
| + P53\_C |  |  |  |  |
| + Roughex |  |  |  |  |
| + S19 | GO:0007275 | multicellular organismal development | [B] |
|  | GO:0042600 | chorion | [C] |
| + Vitelline\_membr |  |  |  |  |

---

## Mycosphaerella [eol|tol]

|  |  |  |  |
| --- | --- | --- | --- |
| **Pfam domain(s)** | **GO term acc** | **GO term** | **GO namespace** |

---

## Nematoda [eol|tol]

|  |  |  |  |
| --- | --- | --- | --- |
| **Pfam domain(s)** | **GO term acc** | **GO term** | **GO namespace** |
| + 7TM\_GPCR\_Srab |  |  |  |  |
| + C6 |  |  |  |  |
| + Caenor\_Her-1 |  |  |  |  |
| + Chromadorea\_ALT |  |  |  |  |
| + Col\_cuticle\_N | GO:0042302 | structural constituent of cuticle | [M] |
| + CX |  |  |  |  |
| + DUF236 |  |  |  |  |
| + DUF290 |  |  |  |  |
| + DUF644 |  |  |  |  |
| + Gp-FAR-1 | GO:0008289 | lipid binding | [M] |
| + Ground-like |  |  |  |  |
| + Ly-6\_related |  |  |  |  |
| + MFP2b |  |  |  |  |
| + Pepsin-I3 |  |  |  |  |
| + ZYG-11\_interact |  |  |  |  |

---

## Neosartorya\_Emericella

|  |  |  |  |
| --- | --- | --- | --- |
| **Pfam domain(s)** | **GO term acc** | **GO term** | **GO namespace** |

---

## Onygenales [eol|tol]

|  |  |  |  |
| --- | --- | --- | --- |
| **Pfam domain(s)** | **GO term acc** | **GO term** | **GO namespace** |
| + DUF346 |  |  |  |  |

---

## Oomycetes [eol|tol]

|  |  |  |  |
| --- | --- | --- | --- |
| **Pfam domain(s)** | **GO term acc** | **GO term** | **GO namespace** |
| + Elicitin | GO:0006952 | defense response | [B] |
|  | GO:0009405 | pathogenesis | [B] |
|  | GO:0005576 | extracellular region | [C] |
| + PBCV\_basic\_adap |  |  |  |  |
| + PcF |  |  |  |  |

---

## Opisthokonta [eol|tol]

|  |  |  |  |
| --- | --- | --- | --- |
| **Pfam domain(s)** | **GO term acc** | **GO term** | **GO namespace** |
| + Acyl-CoA\_dh\_2 |  |  |  |  |
| + Ago\_hook |  |  |  |  |
| + AGTRAP |  |  |  |  |
| + Alpha\_GJ | GO:0019050 | suppression by virus of host apoptosis | [B] |
| + ApoO |  |  |  |  |
| + Arylesterase | GO:0004064 | arylesterase activity | [M] |
| + ASD2 |  |  |  |  |
| + ATP-synt\_S1 | GO:0046933 | hydrogen ion transporting ATP synthase activity, rotational mechanism | [M] |
|  | GO:0046961 | proton-transporting ATPase activity, rotational mechanism | [M] |
|  | GO:0015986 | ATP synthesis coupled proton transport | [B] |
|  | GO:0033180 | proton-transporting V-type ATPase, V1 domain | [C] |
| + BAR\_3\_WASP\_bdg |  |  |  |  |
| + BC10 |  |  |  |  |
| + Beta-trefoil | GO:0003700 | transcription factor activity | [M] |
|  | GO:0005515 | protein binding | [M] |
|  | GO:0045449 | regulation of transcription | [B] |
|  | GO:0005634 | nucleus | [C] |
| + bZIP\_Maf | GO:0003677 | DNA binding | [M] |
|  | GO:0006355 | regulation of transcription, DNA-dependent | [B] |
|  | GO:0005634 | nucleus | [C] |
| + C2-set | GO:0007155 | cell adhesion | [B] |
|  | GO:0016021 | integral to membrane | [C] |
| + CENP-K |  |  |  |  |
| + CENP-M |  |  |  |  |
| + CFIA\_Pcf11 |  |  |  |  |
| + Cg6151-P |  |  |  |  |
| + CHL4 |  |  |  |  |
| + Cnl2\_NKP2 |  |  |  |  |
| + Colicin\_D |  |  |  |  |
| + COMPASS-Shg1 |  |  |  |  |
| + COX5A | GO:0004129 | cytochrome-c oxidase activity | [M] |
| + COX7C | GO:0004129 | cytochrome-c oxidase activity | [M] |
| + CP2 |  |  |  |  |
| + Csm1 |  |  |  |  |
| + CTF\_NFI | GO:0003700 | transcription factor activity | [M] |
|  | GO:0006260 | DNA replication | [B] |
|  | GO:0006355 | regulation of transcription, DNA-dependent | [B] |
|  | GO:0005634 | nucleus | [C] |
| + Cu-oxidase\_4 |  |  |  |  |
| + Cytadhesin\_P30 | GO:0007157 | heterophilic cell adhesion | [B] |
|  | GO:0009405 | pathogenesis | [B] |
|  | GO:0016021 | integral to membrane | [C] |
| + Cytidylate\_kin | GO:0004127 | cytidylate kinase activity | [M] |
|  | GO:0005524 | ATP binding | [M] |
|  | GO:0006139 | nucleobase, nucleoside, nucleotide and nucleic acid metabolic process | [B] |
| + DASH\_Hsk3 |  |  |  |  |
| + Defensin\_2 | GO:0006952 | defense response | [B] |
| + Dioxygenase\_N | GO:0005506 | iron ion binding | [M] |
|  | GO:0018576 | catechol 1,2-dioxygenase activity | [M] |
|  | GO:0009712 | catechol metabolic process | [B] |
|  | GO:0055114 | oxidation reduction | [B] |
| + DMAP\_binding | GO:0008134 | transcription factor binding | [M] |
|  | GO:0005634 | nucleus | [C] |
| + DUF1167 |  |  |  |  |
| + DUF1388 |  |  |  |  |
| + DUF1510 |  |  |  |  |
| + DUF1519 |  |  |  |  |
| + DUF1602 |  |  |  |  |
| + DUF1690 |  |  |  |  |
| + DUF1703 |  |  |  |  |
| + DUF1793 |  |  |  |  |
| + DUF1907 | GO:0008270 | zinc ion binding | [M] |
|  | GO:0016788 | hydrolase activity, acting on ester bonds | [M] |
|  | GO:0005634 | nucleus | [C] |
| + DUF1932 |  |  |  |  |
| + DUF1939 |  |  |  |  |
| + DUF1965 |  |  |  |  |
| + DUF1993 |  |  |  |  |
| + DUF2241 |  |  |  |  |
| + DUF2278 |  |  |  |  |
| + DUF2315 |  |  |  |  |
| + DUF2367 |  |  |  |  |
| + DUF2370 |  |  |  |  |
| + DUF2397 |  |  |  |  |
| + DUF2405 |  |  |  |  |
| + DUF2413 |  |  |  |  |
| + DUF2855 |  |  |  |  |
| + DUF2867 |  |  |  |  |
| + DUF3106 |  |  |  |  |
| + DUF3115 |  |  |  |  |
| + DUF3295 |  |  |  |  |
| + DUF3361 |  |  |  |  |
| + DUF3471 |  |  |  |  |
| + DUF3505 |  |  |  |  |
| + DUF3694 |  |  |  |  |
| + DUF612 |  |  |  |  |
| + E\_Pc\_C |  |  |  |  |
| + Eco57I | GO:0003677 | DNA binding | [M] |
|  | GO:0003824 | catalytic activity | [M] |
|  | GO:0006304 | DNA modification | [B] |
| + EIF4E-T |  |  |  |  |
| + Endomucin |  |  |  |  |
| + Enhancin | GO:0016032 | viral reproduction | [B] |
| + EPTP |  |  |  |  |
| + ETX\_MTX2 |  |  |  |  |
| + Fmp27 |  |  |  |  |
| + Fmp27\_WPPW |  |  |  |  |
| + FTP | GO:0004222 | metalloendopeptidase activity | [M] |
|  | GO:0008270 | zinc ion binding | [M] |
| + GAGA |  |  |  |  |
| + GCM | GO:0003677 | DNA binding | [M] |
|  | GO:0006355 | regulation of transcription, DNA-dependent | [B] |
| + GETHR |  |  |  |  |
| + GIT\_SHD |  |  |  |  |
| + GLTT |  |  |  |  |
| + Glutenin\_hmw | GO:0045735 | nutrient reservoir activity | [M] |
| + Glyco\_hydro\_26 | GO:0016985 | mannan endo-1,4-beta-mannosidase activity | [M] |
|  | GO:0006080 | substituted mannan metabolic process | [B] |
| + Hamartin |  |  |  |  |
| + HbrB |  |  |  |  |
| + He\_PIG |  |  |  |  |
| + Hemocyanin\_C |  |  |  |  |
| + Hemocyanin\_M | GO:0005344 | oxygen transporter activity | [M] |
|  | GO:0006810 | transport | [B] |
| + Hemopexin |  |  |  |  |
| + Hexapep | GO:0016740 | transferase activity | [M] |
| + HMG14\_17 | GO:0003677 | DNA binding | [M] |
|  | GO:0000785 | chromatin | [C] |
|  | GO:0005634 | nucleus | [C] |
| + HPC2 |  |  |  |  |
| + HsbA |  |  |  |  |
| + JTB | GO:0016021 | integral to membrane | [C] |
| + L\_HGMIC\_fpl |  |  |  |  |
| + LAG1-DNAbind | GO:0003677 | DNA binding | [M] |
|  | GO:0003700 | transcription factor activity | [M] |
|  | GO:0006355 | regulation of transcription, DNA-dependent | [B] |
|  | GO:0005634 | nucleus | [C] |
| + Lge1 |  |  |  |  |
| + MannoseP\_isomer | GO:0016779 | nucleotidyltransferase activity | [M] |
|  | GO:0005976 | polysaccharide metabolic process | [B] |
| + MAS20 | GO:0006605 | protein targeting | [B] |
|  | GO:0006886 | intracellular protein transport | [B] |
|  | GO:0005742 | mitochondrial outer membrane translocase complex | [C] |
| + MBA1 |  |  |  |  |
| + MEA1 | GO:0007283 | spermatogenesis | [B] |
| + Med13\_N |  |  |  |  |
| + Med15\_fungi |  |  |  |  |
| + Med16 |  |  |  |  |
| + MRP-S23 |  |  |  |  |
| + Muskelin\_N |  |  |  |  |
| + N-SET |  |  |  |  |
| + NCD2 | GO:0016564 | transcription repressor activity | [M] |
|  | GO:0016481 | negative regulation of transcription | [B] |
|  | GO:0005634 | nucleus | [C] |
| + NDUF\_B4 | GO:0008137 | NADH dehydrogenase (ubiquinone) activity | [M] |
|  | GO:0005739 | mitochondrion | [C] |
| + NDUF\_B8 | GO:0003954 | NADH dehydrogenase activity | [M] |
|  | GO:0008137 | NADH dehydrogenase (ubiquinone) activity | [M] |
|  | GO:0005739 | mitochondrion | [C] |
| + Nup153 |  |  |  |  |
| + PA28\_alpha | GO:0008538 | proteasome activator activity | [M] |
|  | GO:0008537 | proteasome activator complex | [C] |
| + PACT\_coil\_coil |  |  |  |  |
| + PAD | GO:0004668 | protein-arginine deiminase activity | [M] |
|  | GO:0005509 | calcium ion binding | [M] |
|  | GO:0005737 | cytoplasm | [C] |
| + Pam17 |  |  |  |  |
| + Paramyx\_RNA\_pol | GO:0003723 | RNA binding | [M] |
|  | GO:0003968 | RNA-directed RNA polymerase activity | [M] |
|  | GO:0006350 | transcription | [B] |
| + Peptidase\_A2B |  |  |  |  |
| + Peptidase\_C1\_2 | GO:0004197 | cysteine-type endopeptidase activity | [M] |
|  | GO:0006508 | proteolysis | [B] |
| + Peptidase\_M4 | GO:0004222 | metalloendopeptidase activity | [M] |
| + Peptidase\_M4\_C | GO:0004222 | metalloendopeptidase activity | [M] |
|  | GO:0006508 | proteolysis | [B] |
|  | GO:0005576 | extracellular region | [C] |
| + Peptidase\_M57 |  |  |  |  |
| + Phospholip\_A2\_3 |  |  |  |  |
| + Podoplanin | GO:0016021 | integral to membrane | [C] |
| + PRANC |  |  |  |  |
| + Rad54\_N |  |  |  |  |
| + Rap1\_C |  |  |  |  |
| + Rbsn |  |  |  |  |
| + RCR |  |  |  |  |
| + Relaxase |  |  |  |  |
| + RFX\_DNA\_binding | GO:0003677 | DNA binding | [M] |
|  | GO:0006355 | regulation of transcription, DNA-dependent | [B] |
| + Ribonuclease | GO:0003723 | RNA binding | [M] |
|  | GO:0004521 | endoribonuclease activity | [M] |
| + Ribosomal\_L50 |  |  |  |  |
| + RNA\_pol\_I\_TF |  |  |  |  |
| + RNase\_P\_pop3 |  |  |  |  |
| + SAFF |  |  |  |  |
| + Scm3 |  |  |  |  |
| + Sec2p |  |  |  |  |
| + Securin | GO:0006259 | DNA metabolic process | [B] |
|  | GO:0051276 | chromosome organization | [B] |
|  | GO:0005634 | nucleus | [C] |
|  | GO:0005737 | cytoplasm | [C] |
| + Shugoshin\_N | GO:0045132 | meiotic chromosome segregation | [B] |
|  | GO:0000775 | chromosome, centromeric region | [C] |
|  | GO:0005634 | nucleus | [C] |
| + Slx4 |  |  |  |  |
| + Spo12 |  |  |  |  |
| + Spuma\_A9PTase | GO:0004190 | aspartic-type endopeptidase activity | [M] |
|  | GO:0006508 | proteolysis | [B] |
| + SSDP | GO:0003677 | DNA binding | [M] |
|  | GO:0005634 | nucleus | [C] |
| + Ste50p-SAM |  |  |  |  |
| + SUN |  |  |  |  |
| + SWI-SNF\_Ssr4 |  |  |  |  |
| + TAN |  |  |  |  |
| + Terminase\_5 | GO:0005524 | ATP binding | [M] |
|  | GO:0019069 | viral capsid assembly | [B] |
| + ThuA |  |  |  |  |
| + TMV\_coat | GO:0005198 | structural molecule activity | [M] |
|  | GO:0019028 | viral capsid | [C] |
| + TRAM1 |  |  |  |  |
| + TRF | GO:0005515 | protein binding | [M] |
|  | GO:0042162 | telomeric DNA binding | [M] |
|  | GO:0007004 | telomere maintenance via telomerase | [B] |
|  | GO:0000781 | chromosome, telomeric region | [C] |
|  | GO:0005634 | nucleus | [C] |
| + Tubulin-binding | GO:0007026 | negative regulation of microtubule depolymerization | [B] |
| + UBA\_2 | GO:0004674 | protein serine/threonine kinase activity | [M] |
| + UNC45-central |  |  |  |  |
| + UPF0506 |  |  |  |  |
| + UXS1\_N |  |  |  |  |
| + V-set |  |  |  |  |
| + VanZ |  |  |  |  |
| + WTX |  |  |  |  |
| + XPA\_N | GO:0003684 | damaged DNA binding | [M] |
|  | GO:0006289 | nucleotide-excision repair | [B] |
|  | GO:0005634 | nucleus | [C] |
| + YqcI\_YcgG |  |  |  |  |

---

## Ostreococcus [eol|tol]

|  |  |  |  |
| --- | --- | --- | --- |
| **Pfam domain(s)** | **GO term acc** | **GO term** | **GO namespace** |

---

## Pelagophyceae\_Bacillariophyta

|  |  |  |  |
| --- | --- | --- | --- |
| **Pfam domain(s)** | **GO term acc** | **GO term** | **GO namespace** |
| + DUF2872 |  |  |  |  |

---

## Pezizomycotina [eol|tol]

|  |  |  |  |
| --- | --- | --- | --- |
| **Pfam domain(s)** | **GO term acc** | **GO term** | **GO namespace** |
| + AflR | GO:0003677 | DNA binding | [M] |
|  | GO:0045122 | aflatoxin biosynthetic process | [B] |
|  | GO:0045449 | regulation of transcription | [B] |
|  | GO:0005634 | nucleus | [C] |
| + Bys1 |  |  |  |  |
| + DUF2293 |  |  |  |  |
| + DUF3176 |  |  |  |  |
| + DUF3517 |  |  |  |  |
| + DUF3636 |  |  |  |  |
| + DUF3716 |  |  |  |  |
| + DUF3723 |  |  |  |  |
| + Ecm33 |  |  |  |  |
| + Fungal\_lectin |  |  |  |  |
| + KGG |  |  |  |  |

---

## Plasmodium [eol|tol]

|  |  |  |  |
| --- | --- | --- | --- |
| **Pfam domain(s)** | **GO term acc** | **GO term** | **GO namespace** |
| + CRA |  |  |  |  |
| + DUF2081 |  |  |  |  |
| + Duffy\_binding | GO:0004872 | receptor activity | [M] |
|  | GO:0009405 | pathogenesis | [B] |
|  | GO:0016021 | integral to membrane | [C] |
| + EBA-175\_VI |  |  |  |  |
| + MSP1\_C | GO:0009405 | pathogenesis | [B] |
|  | GO:0016020 | membrane | [C] |
| + PfUIS3 |  |  |  |  |
| + TryThrA\_C |  |  |  |  |
| + Wx5\_PLAF3D7 |  |  |  |  |

---

## Pleosporaceae [eol|tol]

|  |  |  |  |
| --- | --- | --- | --- |
| **Pfam domain(s)** | **GO term acc** | **GO term** | **GO namespace** |

---

## Pleosporales [eol|tol]

|  |  |  |  |
| --- | --- | --- | --- |
| **Pfam domain(s)** | **GO term acc** | **GO term** | **GO namespace** |
| + Toxin\_ToxA |  |  |  |  |

---

## Poales [eol|tol]

|  |  |  |  |
| --- | --- | --- | --- |
| **Pfam domain(s)** | **GO term acc** | **GO term** | **GO namespace** |
| + BAP |  |  |  |  |
| + DUF1110 |  |  |  |  |
| + DUF1618 |  |  |  |  |
| + DUF1668 |  |  |  |  |
| + DUF1719 |  |  |  |  |
| + DUF3123 |  |  |  |  |
| + DUF3681 |  |  |  |  |
| + ELF |  |  |  |  |

---

## Prasinophyceae [eol|tol]

|  |  |  |  |
| --- | --- | --- | --- |
| **Pfam domain(s)** | **GO term acc** | **GO term** | **GO namespace** |
| + GYD |  |  |  |  |
| + SecG | GO:0015450 | P-P-bond-hydrolysis-driven protein transmembrane transporter activity | [M] |
|  | GO:0009306 | protein secretion | [B] |
|  | GO:0016021 | integral to membrane | [C] |

---

## Primates [eol|tol]

|  |  |  |  |
| --- | --- | --- | --- |
| **Pfam domain(s)** | **GO term acc** | **GO term** | **GO namespace** |
| + Cementoin |  |  |  |  |
| + DUF3625 |  |  |  |  |
| + OGFr\_III |  |  |  |  |
| + RFPL3\_antisense |  |  |  |  |
| + SPAN-X |  |  |  |  |
| + Statherin |  |  |  |  |

---

## Protostomia [eol|tol]

|  |  |  |  |
| --- | --- | --- | --- |
| **Pfam domain(s)** | **GO term acc** | **GO term** | **GO namespace** |
| + 7tm\_6 | GO:0004984 | olfactory receptor activity | [M] |
|  | GO:0005549 | odorant binding | [M] |
|  | GO:0007608 | sensory perception of smell | [B] |
|  | GO:0016020 | membrane | [C] |
| + 7TM\_GPCR\_Srbc |  |  |  |  |
| + Adipokin\_hormo | GO:0005179 | hormone activity | [M] |
|  | GO:0005576 | extracellular region | [C] |
| + CCAP |  |  |  |  |
| + DUF1261 |  |  |  |  |
| + DUF1487 |  |  |  |  |
| + DUF3609 |  |  |  |  |
| + DUF3646 |  |  |  |  |
| + FA\_synthesis | GO:0003824 | catalytic activity | [M] |
|  | GO:0006633 | fatty acid biosynthetic process | [B] |
| + LamB | GO:0005351 | sugar:hydrogen symporter activity | [M] |
|  | GO:0006810 | transport | [B] |
|  | GO:0016020 | membrane | [C] |
| + Mrr\_cat | GO:0003677 | DNA binding | [M] |
|  | GO:0004519 | endonuclease activity | [M] |
|  | GO:0009307 | DNA restriction-modification system | [B] |
| + NIL |  |  |  |  |
| + NodS | GO:0008757 | S-adenosylmethionine-dependent methyltransferase activity | [M] |
|  | GO:0009312 | oligosaccharide biosynthetic process | [B] |
|  | GO:0009877 | nodulation | [B] |
| + Parvo\_NS1 | GO:0019079 | viral genome replication | [B] |
| + PTS\_EIIB |  |  |  |  |
| + Serpentine\_r\_xa |  |  |  |  |

---

## Pucciniomycetes [eol|tol]

|  |  |  |  |
| --- | --- | --- | --- |
| **Pfam domain(s)** | **GO term acc** | **GO term** | **GO namespace** |

---

## Pucciniomycotina [eol|tol]

|  |  |  |  |
| --- | --- | --- | --- |
| **Pfam domain(s)** | **GO term acc** | **GO term** | **GO namespace** |

---

## Pucciniomycotina\_Agaricomycotina

|  |  |  |  |
| --- | --- | --- | --- |
| **Pfam domain(s)** | **GO term acc** | **GO term** | **GO namespace** |

---

## Rodentia [eol|tol]

|  |  |  |  |
| --- | --- | --- | --- |
| **Pfam domain(s)** | **GO term acc** | **GO term** | **GO namespace** |
| + DUF1438 |  |  |  |  |
| + GLYCAM-1 |  |  |  |  |
| + Involucrin2 | GO:0030216 | keratinocyte differentiation | [B] |
|  | GO:0001533 | cornified envelope | [C] |
| + Lipoprotein\_7 |  |  |  |  |
| + Orthopox\_A5L |  |  |  |  |

---

## Saccharomycetaceae [eol|tol]

|  |  |  |  |
| --- | --- | --- | --- |
| **Pfam domain(s)** | **GO term acc** | **GO term** | **GO namespace** |
| + Atg31 |  |  |  |  |
| + BAF1\_ABF1 | GO:0003677 | DNA binding | [M] |
|  | GO:0006338 | chromatin remodeling | [B] |
|  | GO:0005634 | nucleus | [C] |
| + chaperone\_DMP |  |  |  |  |
| + DSL1 |  |  |  |  |
| + Dsl1\_N |  |  |  |  |
| + DUF1892 |  |  |  |  |
| + DUF2578 |  |  |  |  |
| + DUF2702 |  |  |  |  |
| + DUP |  |  |  |  |
| + EST1\_DNA\_bind |  |  |  |  |
| + Est3 | GO:0042162 | telomeric DNA binding | [M] |
|  | GO:0007004 | telomere maintenance via telomerase | [B] |
|  | GO:0032508 | DNA duplex unwinding | [B] |
|  | GO:0000781 | chromosome, telomeric region | [C] |
|  | GO:0005697 | telomerase holoenzyme complex | [C] |
| + Flocculin | GO:0000128 | flocculation | [B] |
| + Helicase\_Sgs1 |  |  |  |  |
| + HRP1 |  |  |  |  |
| + Hyphal\_reg\_CWP |  |  |  |  |
| + MAM1 |  |  |  |  |
| + Med3 |  |  |  |  |
| + Mer2 |  |  |  |  |
| + MetRS-N |  |  |  |  |
| + MF\_alpha | GO:0000772 | mating pheromone activity | [M] |
|  | GO:0019953 | sexual reproduction | [B] |
|  | GO:0005576 | extracellular region | [C] |
| + MF\_alpha\_N | GO:0007618 | mating | [B] |
|  | GO:0005576 | extracellular region | [C] |
| + Mvb12 |  |  |  |  |
| + Nab2 |  |  |  |  |
| + Nab6\_mRNP\_bdg |  |  |  |  |
| + Nse5 |  |  |  |  |
| + Nyv1\_N |  |  |  |  |
| + Pho86 |  |  |  |  |
| + Rad33 |  |  |  |  |
| + RPM2 |  |  |  |  |
| + Sec31 |  |  |  |  |
| + Sfi1\_C |  |  |  |  |
| + She2p |  |  |  |  |
| + Sir1 |  |  |  |  |
| + SRP1\_TIP1 | GO:0006950 | response to stress | [B] |
| + THP2 |  |  |  |  |
| + Ubiq-assoc |  |  |  |  |

---

## Saccharomycotina [eol|tol]

|  |  |  |  |
| --- | --- | --- | --- |
| **Pfam domain(s)** | **GO term acc** | **GO term** | **GO namespace** |
| + DUF1689 |  |  |  |  |
| + Gsf2 |  |  |  |  |
| + Mgr1 |  |  |  |  |
| + POC1 |  |  |  |  |
| + Rtt102p |  |  |  |  |
| + SAC3 |  |  |  |  |
| + Vhr1 |  |  |  |  |

---

## Saccharomycotina\_Taphrinomycotina

|  |  |  |  |
| --- | --- | --- | --- |
| **Pfam domain(s)** | **GO term acc** | **GO term** | **GO namespace** |
| + CRF1 |  |  |  |  |
| + Flo11 |  |  |  |  |
| + LCD1 |  |  |  |  |
| + Meiotic\_rec114 | GO:0007131 | reciprocal meiotic recombination | [B] |
| + Pet20 |  |  |  |  |
| + Red1 | GO:0007059 | chromosome segregation | [B] |
|  | GO:0007131 | reciprocal meiotic recombination | [B] |
| + Saw1 |  |  |  |  |
| + SID |  |  |  |  |
| + Vel1p |  |  |  |  |

---

## Smegmamorpha [eol|tol]

|  |  |  |  |
| --- | --- | --- | --- |
| **Pfam domain(s)** | **GO term acc** | **GO term** | **GO namespace** |

---

## Sordariales [eol|tol]

|  |  |  |  |
| --- | --- | --- | --- |
| **Pfam domain(s)** | **GO term acc** | **GO term** | **GO namespace** |

---

## Sordariomycetes [eol|tol]

|  |  |  |  |
| --- | --- | --- | --- |
| **Pfam domain(s)** | **GO term acc** | **GO term** | **GO namespace** |
| + Mu-like\_Com |  |  |  |  |

---

## Sordariomycetes\_Dothideomycetes

|  |  |  |  |
| --- | --- | --- | --- |
| **Pfam domain(s)** | **GO term acc** | **GO term** | **GO namespace** |
| + 2H-phosphodiest |  |  |  |  |
| + 3-alpha |  |  |  |  |
| + Enterotoxin\_a | GO:0003824 | catalytic activity | [M] |
|  | GO:0009405 | pathogenesis | [B] |
|  | GO:0005576 | extracellular region | [C] |
| + Hydrophobin\_2 | GO:0007154 | cell communication | [B] |
|  | GO:0005576 | extracellular region | [C] |
|  | GO:0005618 | cell wall | [C] |
| + Osmo\_MPGsynth | GO:0050504 | mannosyl-3-phosphoglycerate synthase activity | [M] |
|  | GO:0051479 | mannosylglycerate biosynthetic process | [B] |
|  | GO:0005737 | cytoplasm | [C] |
| + Pro\_Al\_protease | GO:0008236 | serine-type peptidase activity | [M] |
|  | GO:0006508 | proteolysis | [B] |
|  | GO:0005576 | extracellular region | [C] |
| + TcdB\_toxin\_midN |  |  |  |  |

---

## Sordariomycetes\_Dothideomycetes\_Eurotiales

|  |  |  |  |
| --- | --- | --- | --- |
| **Pfam domain(s)** | **GO term acc** | **GO term** | **GO namespace** |
| + Antifungal\_prot |  |  |  |  |
| + CBP |  |  |  |  |
| + DAP\_C | GO:0004177 | aminopeptidase activity | [M] |
| + DUF1593 |  |  |  |  |
| + DUF1962 |  |  |  |  |
| + DUF291 |  |  |  |  |
| + DUF3492 |  |  |  |  |
| + DUF899 |  |  |  |  |
| + MlrC\_C |  |  |  |  |
| + Scytalone\_dh | GO:0030411 | scytalone dehydratase activity | [M] |
|  | GO:0006582 | melanin metabolic process | [B] |

---

## Taphrinomycotina [eol|tol]

|  |  |  |  |
| --- | --- | --- | --- |
| **Pfam domain(s)** | **GO term acc** | **GO term** | **GO namespace** |
| + DIPSY |  |  |  |  |
| + UPF0300 |  |  |  |  |

---

## Teleostei [eol|tol]

|  |  |  |  |
| --- | --- | --- | --- |
| **Pfam domain(s)** | **GO term acc** | **GO term** | **GO namespace** |

---

## Tetraodontiformes [eol|tol]

|  |  |  |  |
| --- | --- | --- | --- |
| **Pfam domain(s)** | **GO term acc** | **GO term** | **GO namespace** |

---

## Tetrapoda [eol|tol]

|  |  |  |  |
| --- | --- | --- | --- |
| **Pfam domain(s)** | **GO term acc** | **GO term** | **GO namespace** |
| + 5HT\_transporter | GO:0005335 | serotonin:sodium symporter activity | [M] |
|  | GO:0006836 | neurotransmitter transport | [B] |
|  | GO:0005887 | integral to plasma membrane | [C] |
| + Amelin | GO:0030345 | structural constituent of tooth enamel | [M] |
|  | GO:0042475 | odontogenesis of dentine-containing tooth | [B] |
| + Amelogenin | GO:0007275 | multicellular organismal development | [B] |
|  | GO:0005578 | proteinaceous extracellular matrix | [C] |
| + CXCR4\_N |  |  |  |  |
| + EVI2A | GO:0016021 | integral to membrane | [C] |
| + ITAM | GO:0004888 | transmembrane receptor activity | [M] |
|  | GO:0007166 | cell surface receptor linked signal transduction | [B] |
|  | GO:0016020 | membrane | [C] |
| + Keratin\_B2 | GO:0045095 | keratin filament | [C] |
| + KRAB | GO:0003676 | nucleic acid binding | [M] |
|  | GO:0006355 | regulation of transcription, DNA-dependent | [B] |
|  | GO:0005622 | intracellular | [C] |
| + Neuromodulin | GO:0040008 | regulation of growth | [B] |
| + P68HR | GO:0003724 | RNA helicase activity | [M] |
|  | GO:0005515 | protein binding | [M] |
|  | GO:0016818 | hydrolase activity, acting on acid anhydrides, in phosphorus-containing anhydrides | [M] |
|  | GO:0005634 | nucleus | [C] |
| + PRF |  |  |  |  |
| + Prion | GO:0051260 | protein homooligomerization | [B] |
|  | GO:0016020 | membrane | [C] |
| + RFamide\_26RFa |  |  |  |  |
| + Stonin2\_N |  |  |  |  |
| + Surfac\_D-trimer |  |  |  |  |
| + Tachykinin | GO:0007217 | tachykinin receptor signaling pathway | [B] |
|  | GO:0007268 | synaptic transmission | [B] |
| + Troponin-I\_N |  |  |  |  |
| + VitD-bind\_III |  |  |  |  |
| + zf-RAG1 |  |  |  |  |

---

## Theileria [eol|tol]

|  |  |  |  |
| --- | --- | --- | --- |
| **Pfam domain(s)** | **GO term acc** | **GO term** | **GO namespace** |
| + EMA |  |  |  |  |
| + FAINT |  |  |  |  |
| + RAP-1 |  |  |  |  |
| + Tash\_PEST |  |  |  |  |

---

## Tracheophyta [eol|tol]

|  |  |  |  |
| --- | --- | --- | --- |
| **Pfam domain(s)** | **GO term acc** | **GO term** | **GO namespace** |
| + ABA\_WDS | GO:0006950 | response to stress | [B] |
| + DUF1070 |  |  |  |  |
| + DUF1685 |  |  |  |  |
| + DUF26 |  |  |  |  |
| + DUF3049 |  |  |  |  |
| + DUF3527 |  |  |  |  |
| + DUF573 |  |  |  |  |
| + DUF620 |  |  |  |  |
| + DUF740 |  |  |  |  |
| + DUF863 |  |  |  |  |
| + KIP1 |  |  |  |  |
| + LEA\_1 | GO:0009790 | embryonic development | [B] |
| + LEA\_3 | GO:0006950 | response to stress | [B] |
| + Lir1 |  |  |  |  |
| + MatK\_N | GO:0006397 | mRNA processing | [B] |
|  | GO:0009507 | chloroplast | [C] |
| + Metallothio\_PEC | GO:0008270 | zinc ion binding | [M] |
| + MraY\_sig1 |  |  |  |  |
| + Oxidored\_q1\_C | GO:0008137 | NADH dehydrogenase (ubiquinone) activity | [M] |
|  | GO:0042773 | ATP synthesis coupled electron transport | [B] |
|  | GO:0055114 | oxidation reduction | [B] |
| + Phytochelatin\_C | GO:0016756 | glutathione gamma-glutamylcysteinyltransferase activity | [M] |
|  | GO:0046872 | metal ion binding | [M] |
|  | GO:0010038 | response to metal ion | [B] |
|  | GO:0046938 | phytochelatin biosynthetic process | [B] |
| + PQQ\_N | GO:0016614 | oxidoreductase activity, acting on CH-OH group of donors | [M] |
|  | GO:0055114 | oxidation reduction | [B] |
| + Prot\_inhib\_II | GO:0004867 | serine-type endopeptidase inhibitor activity | [M] |

---

## Trebouxiophyceae\_Chlorophyceae

|  |  |  |  |
| --- | --- | --- | --- |
| **Pfam domain(s)** | **GO term acc** | **GO term** | **GO namespace** |

---

## Tremellomycetes [eol|tol]

|  |  |  |  |
| --- | --- | --- | --- |
| **Pfam domain(s)** | **GO term acc** | **GO term** | **GO namespace** |

---

## Unikonta [eol|tol]

|  |  |  |  |
| --- | --- | --- | --- |
| **Pfam domain(s)** | **GO term acc** | **GO term** | **GO namespace** |
| + A\_deaminase\_N | GO:0005615 | extracellular space | [C] |
| + AdoMet\_MTase | GO:0008168 | methyltransferase activity | [M] |
| + Asp-B-Hydro\_N | GO:0004597 | peptide-aspartate beta-dioxygenase activity | [M] |
|  | GO:0005506 | iron ion binding | [M] |
|  | GO:0055114 | oxidation reduction | [B] |
|  | GO:0030176 | integral to endoplasmic reticulum membrane | [C] |
| + Bacillus\_HBL | GO:0009405 | pathogenesis | [B] |
|  | GO:0016020 | membrane | [C] |
| + BAR\_2 |  |  |  |  |
| + Calcipressin | GO:0019722 | calcium-mediated signaling | [B] |
| + Cbl\_N2 | GO:0004871 | signal transducer activity | [M] |
|  | GO:0007166 | cell surface receptor linked signal transduction | [B] |
|  | GO:0005634 | nucleus | [C] |
| + Cgr1 |  |  |  |  |
| + Cid2 |  |  |  |  |
| + Copper-fist | GO:0003677 | DNA binding | [M] |
|  | GO:0003700 | transcription factor activity | [M] |
|  | GO:0005507 | copper ion binding | [M] |
|  | GO:0006355 | regulation of transcription, DNA-dependent | [B] |
|  | GO:0005634 | nucleus | [C] |
| + Cortex-I\_coil |  |  |  |  |
| + COX9 | GO:0004129 | cytochrome-c oxidase activity | [M] |
|  | GO:0022904 | respiratory electron transport chain | [B] |
|  | GO:0055114 | oxidation reduction | [B] |
|  | GO:0005746 | mitochondrial respiratory chain | [C] |
| + COXG |  |  |  |  |
| + Cytochrom\_B558a | GO:0020037 | heme binding | [M] |
| + DUF1206 |  |  |  |  |
| + DUF1241 |  |  |  |  |
| + DUF162 |  |  |  |  |
| + DUF1693 |  |  |  |  |
| + DUF1748 |  |  |  |  |
| + DUF1881 |  |  |  |  |
| + DUF2133 |  |  |  |  |
| + DUF2151 |  |  |  |  |
| + DUF2205 |  |  |  |  |
| + DUF2349 |  |  |  |  |
| + DUF2418 |  |  |  |  |
| + DUF2678 |  |  |  |  |
| + DUF333 |  |  |  |  |
| + DUF619 | GO:0003991 | acetylglutamate kinase activity | [M] |
|  | GO:0006526 | arginine biosynthetic process | [B] |
| + DUF758 |  |  |  |  |
| + Dynactin |  |  |  |  |
| + Dynein\_IC2 |  |  |  |  |
| + FSA\_C |  |  |  |  |
| + G-gamma | GO:0004871 | signal transducer activity | [M] |
|  | GO:0007186 | G-protein coupled receptor protein signaling pathway | [B] |
|  | GO:0005834 | heterotrimeric G-protein complex | [C] |
| + GAS2 | GO:0007050 | cell cycle arrest | [B] |
| + Gly\_acyl\_tr\_C |  |  |  |  |
| + GPP34 |  |  |  |  |
| + HIRA\_B | GO:0003682 | chromatin binding | [M] |
|  | GO:0030528 | transcription regulator activity | [M] |
|  | GO:0006355 | regulation of transcription, DNA-dependent | [B] |
|  | GO:0016568 | chromatin modification | [B] |
|  | GO:0005634 | nucleus | [C] |
| + HR1 | GO:0007165 | signal transduction | [B] |
|  | GO:0005622 | intracellular | [C] |
| + I\_LWEQ | GO:0003779 | actin binding | [M] |
| + Intg\_mem\_TP0381 |  |  |  |  |
| + LOH1CR12 |  |  |  |  |
| + MDM31\_MDM32 | GO:0007005 | mitochondrion organization | [B] |
|  | GO:0005743 | mitochondrial inner membrane | [C] |
| + Med12-LCEWAV |  |  |  |  |
| + Med24\_N |  |  |  |  |
| + Methyltransf\_1N | GO:0003908 | methylated-DNA-[protein]-cysteine S-methyltransferase activity | [M] |
|  | GO:0006281 | DNA repair | [B] |
| + MinC\_N | GO:0042127 | regulation of cell proliferation | [B] |
| + Mis14 |  |  |  |  |
| + Mis6 |  |  |  |  |
| + Myosin\_tail\_1 | GO:0003774 | motor activity | [M] |
|  | GO:0016459 | myosin complex | [C] |
| + NAGidase |  |  |  |  |
| + NDT80\_PhoG |  |  |  |  |
| + Neugrin |  |  |  |  |
| + NKAIN |  |  |  |  |
| + NUDE\_C |  |  |  |  |
| + OAS1\_C |  |  |  |  |
| + PDEase\_I\_N | GO:0004114 | 3',5'-cyclic-nucleotide phosphodiesterase activity | [M] |
| + PDEase\_II | GO:0004115 | 3',5'-cyclic-AMP phosphodiesterase activity | [M] |
|  | GO:0006198 | cAMP catabolic process | [B] |
| + Peptidase\_C39 | GO:0005524 | ATP binding | [M] |
|  | GO:0008233 | peptidase activity | [M] |
|  | GO:0006508 | proteolysis | [B] |
|  | GO:0016021 | integral to membrane | [C] |
| + Perilipin |  |  |  |  |
| + Peroxin-13\_N | GO:0016560 | protein import into peroxisome matrix, docking | [B] |
|  | GO:0005777 | peroxisome | [C] |
|  | GO:0016021 | integral to membrane | [C] |
| + Prothymosin |  |  |  |  |
| + Rab5-bind |  |  |  |  |
| + RNA12 |  |  |  |  |
| + RNA\_helicase | GO:0003723 | RNA binding | [M] |
|  | GO:0003724 | RNA helicase activity | [M] |
| + SAM\_PNT | GO:0043565 | sequence-specific DNA binding | [M] |
|  | GO:0005634 | nucleus | [C] |
| + Spore\_YhcN\_YlaJ |  |  |  |  |
| + Spy1 |  |  |  |  |
| + Thymosin | GO:0003779 | actin binding | [M] |
|  | GO:0007010 | cytoskeleton organization | [B] |
|  | GO:0005737 | cytoplasm | [C] |
| + Tmemb\_170 |  |  |  |  |
| + TraG\_N |  |  |  |  |
| + Tuberin | GO:0005096 | GTPase activator activity | [M] |
|  | GO:0043547 | positive regulation of GTPase activity | [B] |
| + UcrQ | GO:0008121 | ubiquinol-cytochrome-c reductase activity | [M] |
| + UPF0552 |  |  |  |  |
| + UPF0556 |  |  |  |  |
| + VASP\_tetra |  |  |  |  |
| + VBS |  |  |  |  |
| + Vinculin | GO:0005198 | structural molecule activity | [M] |
|  | GO:0007155 | cell adhesion | [B] |
|  | GO:0015629 | actin cytoskeleton | [C] |
| + WAP | GO:0030414 | peptidase inhibitor activity | [M] |
|  | GO:0005576 | extracellular region | [C] |
| + XRCC1\_N | GO:0003684 | damaged DNA binding | [M] |
|  | GO:0000012 | single strand break repair | [B] |
|  | GO:0005634 | nucleus | [C] |

---

## Urochordata [eol|tol]

|  |  |  |  |
| --- | --- | --- | --- |
| **Pfam domain(s)** | **GO term acc** | **GO term** | **GO namespace** |

---

## Urochordata\_Vertebrata

|  |  |  |  |
| --- | --- | --- | --- |
| **Pfam domain(s)** | **GO term acc** | **GO term** | **GO namespace** |
| + ASD1 |  |  |  |  |
| + Bcr-Abl\_Oligo | GO:0004674 | protein serine/threonine kinase activity | [M] |
|  | GO:0005096 | GTPase activator activity | [M] |
|  | GO:0006468 | protein amino acid phosphorylation | [B] |
|  | GO:0007165 | signal transduction | [B] |
| + Calsarcin |  |  |  |  |
| + Connexin |  |  |  |  |
| + Connexin\_CCC |  |  |  |  |
| + MHC2-interact | GO:0042289 | MHC class II protein binding | [M] |
|  | GO:0006886 | intracellular protein transport | [B] |
|  | GO:0006955 | immune response | [B] |
|  | GO:0019882 | antigen processing and presentation | [B] |
|  | GO:0016020 | membrane | [C] |
| + RuvA\_C | GO:0005524 | ATP binding | [M] |
|  | GO:0009378 | four-way junction helicase activity | [M] |
|  | GO:0006281 | DNA repair | [B] |
|  | GO:0006310 | DNA recombination | [B] |
|  | GO:0009379 | Holliday junction helicase complex | [C] |
| + S\_100 |  |  |  |  |
| + SnAPC\_2\_like |  |  |  |  |

---

## Vertebrata [eol|tol]

|  |  |  |  |
| --- | --- | --- | --- |
| **Pfam domain(s)** | **GO term acc** | **GO term** | **GO namespace** |
| + ACTH\_domain |  |  |  |  |
| + Agouti | GO:0009755 | hormone-mediated signaling pathway | [B] |
|  | GO:0005576 | extracellular region | [C] |
| + AKAP7\_RIRII\_bdg |  |  |  |  |
| + AKAP\_110 |  |  |  |  |
| + AlgF |  |  |  |  |
| + AMH\_N | GO:0008083 | growth factor activity | [M] |
|  | GO:0008406 | gonad development | [B] |
| + Androgen\_recep | GO:0003677 | DNA binding | [M] |
|  | GO:0004882 | androgen receptor activity | [M] |
|  | GO:0005496 | steroid binding | [M] |
|  | GO:0006355 | regulation of transcription, DNA-dependent | [B] |
|  | GO:0005634 | nucleus | [C] |
| + ANP | GO:0005179 | hormone activity | [M] |
|  | GO:0005576 | extracellular region | [C] |
| + Ant\_C | GO:0004872 | receptor activity | [M] |
|  | GO:0016021 | integral to membrane | [C] |
| + APC\_basic | GO:0005515 | protein binding | [M] |
|  | GO:0008017 | microtubule binding | [M] |
|  | GO:0016055 | Wnt receptor signaling pathway | [B] |
| + Apo-CII | GO:0008047 | enzyme activator activity | [M] |
|  | GO:0006629 | lipid metabolic process | [B] |
|  | GO:0006869 | lipid transport | [B] |
|  | GO:0042627 | chylomicron | [C] |
| + Apo-CIII | GO:0008289 | lipid binding | [M] |
|  | GO:0006869 | lipid transport | [B] |
|  | GO:0042157 | lipoprotein metabolic process | [B] |
|  | GO:0005576 | extracellular region | [C] |
| + APOBEC\_C | GO:0008270 | zinc ion binding | [M] |
|  | GO:0016814 | hydrolase activity, acting on carbon-nitrogen (but not peptide) bonds, in cyclic amidines | [M] |
| + APOBEC\_N | GO:0008270 | zinc ion binding | [M] |
|  | GO:0016814 | hydrolase activity, acting on carbon-nitrogen (but not peptide) bonds, in cyclic amidines | [M] |
| + ApoC-I | GO:0042157 | lipoprotein metabolic process | [B] |
|  | GO:0005576 | extracellular region | [C] |
| + ApoM |  |  |  |  |
| + ATP1G1\_PLM\_MAT8 | GO:0005216 | ion channel activity | [M] |
|  | GO:0006811 | ion transport | [B] |
|  | GO:0016020 | membrane | [C] |
| + ATXN-1\_C |  |  |  |  |
| + Aurora-A\_bind |  |  |  |  |
| + BAALC\_N |  |  |  |  |
| + BASP1 |  |  |  |  |
| + BCLP |  |  |  |  |
| + BDHCT | GO:0003677 | DNA binding | [M] |
|  | GO:0005524 | ATP binding | [M] |
|  | GO:0016818 | hydrolase activity, acting on acid anhydrides, in phosphorus-containing anhydrides | [M] |
|  | GO:0006260 | DNA replication | [B] |
|  | GO:0005634 | nucleus | [C] |
| + Beta-APP | GO:0005488 | binding | [M] |
|  | GO:0016021 | integral to membrane | [C] |
| + BID | GO:0043065 | positive regulation of apoptosis | [B] |
|  | GO:0005737 | cytoplasm | [C] |
| + Bombesin | GO:0007218 | neuropeptide signaling pathway | [B] |
| + Calcyon | GO:0050780 | dopamine receptor binding | [M] |
|  | GO:0007212 | dopamine receptor signaling pathway | [B] |
|  | GO:0016021 | integral to membrane | [C] |
| + Calpain\_inhib | GO:0010859 | calcium-dependent cysteine-type endopeptidase inhibitor activity | [M] |
| + Caudal\_act | GO:0007275 | multicellular organismal development | [B] |
|  | GO:0045449 | regulation of transcription | [B] |
|  | GO:0005634 | nucleus | [C] |
| + CBFNT |  |  |  |  |
| + CD99L2 |  |  |  |  |
| + Cenp-F\_leu\_zip |  |  |  |  |
| + CNTF | GO:0040007 | growth | [B] |
|  | GO:0005737 | cytoplasm | [C] |
| + Colicin\_im | GO:0015643 | toxin binding | [M] |
|  | GO:0030153 | bacteriocin immunity | [B] |
| + Connexin43 |  |  |  |  |
| + Connexin50 | GO:0007154 | cell communication | [B] |
|  | GO:0005922 | connexon complex | [C] |
| + Cortexin |  |  |  |  |
| + COX7B | GO:0004129 | cytochrome-c oxidase activity | [M] |
|  | GO:0005746 | mitochondrial respiratory chain | [C] |
| + CtIP\_N |  |  |  |  |
| + DAP10 |  |  |  |  |
| + DARPP-32 | GO:0004864 | phosphoprotein phosphatase inhibitor activity | [M] |
|  | GO:0007165 | signal transduction | [B] |
| + Dmrt1 |  |  |  |  |
| + DUF1049 |  |  |  |  |
| + DUF1170 | GO:0009966 | regulation of signal transduction | [B] |
|  | GO:0005737 | cytoplasm | [C] |
|  | GO:0016020 | membrane | [C] |
| + DUF1466 |  |  |  |  |
| + DUF1518 | GO:0003713 | transcription coactivator activity | [M] |
|  | GO:0005634 | nucleus | [C] |
| + DUF1669 |  |  |  |  |
| + DUF1856 |  |  |  |  |
| + DUF1944 | GO:0005319 | lipid transporter activity | [M] |
|  | GO:0006869 | lipid transport | [B] |
| + DUF3314 |  |  |  |  |
| + DUF3345 |  |  |  |  |
| + DUF3371 |  |  |  |  |
| + DUF3377 |  |  |  |  |
| + DUF3436 |  |  |  |  |
| + DUF3446 |  |  |  |  |
| + DUF3481 |  |  |  |  |
| + DUF3496 |  |  |  |  |
| + DUF3528 |  |  |  |  |
| + DUF3544 |  |  |  |  |
| + DUF3776 |  |  |  |  |
| + DUF622 |  |  |  |  |
| + DUF776 |  |  |  |  |
| + DUF959 | GO:0005201 | extracellular matrix structural constituent | [M] |
|  | GO:0007155 | cell adhesion | [B] |
|  | GO:0031012 | extracellular matrix | [C] |
| + Dysbindin | GO:0005515 | protein binding | [M] |
|  | GO:0005737 | cytoplasm | [C] |
| + EB1\_binding | GO:0005515 | protein binding | [M] |
|  | GO:0016055 | Wnt receptor signaling pathway | [B] |
| + EBP50\_C-term |  |  |  |  |
| + ECM1 | GO:0005576 | extracellular region | [C] |
| + ecTbetaR2 | GO:0005026 | transforming growth factor beta receptor activity, type II | [M] |
|  | GO:0005524 | ATP binding | [M] |
|  | GO:0046872 | metal ion binding | [M] |
|  | GO:0006468 | protein amino acid phosphorylation | [B] |
|  | GO:0016020 | membrane | [C] |
| + Endothelin | GO:0019229 | regulation of vasoconstriction | [B] |
|  | GO:0005576 | extracellular region | [C] |
| + EPO\_TPO | GO:0005179 | hormone activity | [M] |
|  | GO:0005576 | extracellular region | [C] |
| + ERbeta\_N |  |  |  |  |
| + EZH2\_WD-Binding |  |  |  |  |
| + Fib\_alpha |  |  |  |  |
| + Fibrinogen\_aC |  |  |  |  |
| + Filament\_head | GO:0005882 | intermediate filament | [C] |
| + Galanin | GO:0005179 | hormone activity | [M] |
|  | GO:0005576 | extracellular region | [C] |
| + GAPT |  |  |  |  |
| + Gastrin | GO:0005179 | hormone activity | [M] |
|  | GO:0005576 | extracellular region | [C] |
| + GCR | GO:0003677 | DNA binding | [M] |
|  | GO:0004883 | glucocorticoid receptor activity | [M] |
|  | GO:0005496 | steroid binding | [M] |
|  | GO:0006355 | regulation of transcription, DNA-dependent | [B] |
|  | GO:0005634 | nucleus | [C] |
| + GluR\_Homer-bdg |  |  |  |  |
| + GMAP |  |  |  |  |
| + GnHR\_trans |  |  |  |  |
| + GnRH | GO:0005179 | hormone activity | [M] |
|  | GO:0007275 | multicellular organismal development | [B] |
|  | GO:0005576 | extracellular region | [C] |
| + Granin |  |  |  |  |
| + GSG-1 |  |  |  |  |
| + Guanylin | GO:0008047 | enzyme activator activity | [M] |
| + Hepsin-SRCR | GO:0004252 | serine-type endopeptidase activity | [M] |
|  | GO:0006508 | proteolysis | [B] |
|  | GO:0016049 | cell growth | [B] |
|  | GO:0016020 | membrane | [C] |
| + HNF-1A\_C | GO:0016563 | transcription activator activity | [M] |
|  | GO:0045941 | positive regulation of transcription | [B] |
|  | GO:0005634 | nucleus | [C] |
| + HNF-1B\_C | GO:0016563 | transcription activator activity | [M] |
|  | GO:0045941 | positive regulation of transcription | [B] |
|  | GO:0005634 | nucleus | [C] |
| + hNIFK\_binding |  |  |  |  |
| + HnRNP\_M |  |  |  |  |
| + HnRNPA1 |  |  |  |  |
| + Homez |  |  |  |  |
| + Hormone\_1 | GO:0005179 | hormone activity | [M] |
|  | GO:0005576 | extracellular region | [C] |
| + Hormone\_2 | GO:0005179 | hormone activity | [M] |
|  | GO:0005576 | extracellular region | [C] |
| + Hormone\_6 | GO:0005179 | hormone activity | [M] |
|  | GO:0005576 | extracellular region | [C] |
| + HoxA13\_N |  |  |  |  |
| + ICAM\_N |  |  |  |  |
| + IFN-gamma | GO:0005133 | interferon-gamma receptor binding | [M] |
|  | GO:0006955 | immune response | [B] |
|  | GO:0005576 | extracellular region | [C] |
| + Ig\_Tie2\_1 | GO:0004714 | transmembrane receptor protein tyrosine kinase activity | [M] |
|  | GO:0005515 | protein binding | [M] |
|  | GO:0005524 | ATP binding | [M] |
|  | GO:0006468 | protein amino acid phosphorylation | [B] |
|  | GO:0007169 | transmembrane receptor protein tyrosine kinase signaling pathway | [B] |
|  | GO:0005887 | integral to plasma membrane | [C] |
| + IGF2\_C |  |  |  |  |
| + IKKbetaNEMObind |  |  |  |  |
| + IL1 | GO:0005615 | extracellular space | [C] |
| + IL10 |  |  |  |  |
| + IL11 |  |  |  |  |
| + IL12 | GO:0005143 | interleukin-12 receptor binding | [M] |
|  | GO:0008083 | growth factor activity | [M] |
|  | GO:0006955 | immune response | [B] |
|  | GO:0005576 | extracellular region | [C] |
| + IL12p40\_C |  |  |  |  |
| + IL1\_propep | GO:0005149 | interleukin-1 receptor binding | [M] |
|  | GO:0006954 | inflammatory response | [B] |
|  | GO:0006955 | immune response | [B] |
| + IL4 | GO:0005136 | interleukin-4 receptor binding | [M] |
|  | GO:0008083 | growth factor activity | [M] |
|  | GO:0006955 | immune response | [B] |
|  | GO:0005576 | extracellular region | [C] |
| + IL4Ra\_N |  |  |  |  |
| + IL6 | GO:0005125 | cytokine activity | [M] |
|  | GO:0006955 | immune response | [B] |
|  | GO:0005576 | extracellular region | [C] |
| + IL8 | GO:0008009 | chemokine activity | [M] |
|  | GO:0006955 | immune response | [B] |
|  | GO:0005576 | extracellular region | [C] |
| + Inhibitor\_Mig-6 |  |  |  |  |
| + Integrase\_DNA | GO:0003677 | DNA binding | [M] |
|  | GO:0008907 | integrase activity | [M] |
|  | GO:0015074 | DNA integration | [B] |
| + Interferon | GO:0005126 | cytokine receptor binding | [M] |
|  | GO:0006952 | defense response | [B] |
|  | GO:0005576 | extracellular region | [C] |
| + IRK\_N |  |  |  |  |
| + ISK\_Channel | GO:0005249 | voltage-gated potassium channel activity | [M] |
|  | GO:0006811 | ion transport | [B] |
|  | GO:0016020 | membrane | [C] |
| + K\_channel\_TID | GO:0005249 | voltage-gated potassium channel activity | [M] |
|  | GO:0030955 | potassium ion binding | [M] |
|  | GO:0006813 | potassium ion transport | [B] |
|  | GO:0016021 | integral to membrane | [C] |
| + KCl\_Cotrans\_1 | GO:0005215 | transporter activity | [M] |
|  | GO:0006811 | ion transport | [B] |
|  | GO:0016020 | membrane | [C] |
| + KcnmB2\_inactiv |  |  |  |  |
| + KCNQC3-Ank-G\_bd |  |  |  |  |
| + Kv2channel | GO:0005249 | voltage-gated potassium channel activity | [M] |
|  | GO:0006813 | potassium ion transport | [B] |
|  | GO:0008076 | voltage-gated potassium channel complex | [C] |
| + L6\_membrane | GO:0016021 | integral to membrane | [C] |
| + Latexin |  |  |  |  |
| + Latrophilin | GO:0004930 | G-protein coupled receptor activity | [M] |
|  | GO:0007186 | G-protein coupled receptor protein signaling pathway | [B] |
|  | GO:0016020 | membrane | [C] |
| + LEAP-2 | GO:0042742 | defense response to bacterium | [B] |
| + Leptin | GO:0005179 | hormone activity | [M] |
|  | GO:0007165 | signal transduction | [B] |
|  | GO:0005576 | extracellular region | [C] |
| + LIF\_OSM | GO:0005125 | cytokine activity | [M] |
|  | GO:0006955 | immune response | [B] |
|  | GO:0005576 | extracellular region | [C] |
| + LSR |  |  |  |  |
| + Ly49 |  |  |  |  |
| + MAGP | GO:0001527 | microfibril | [C] |
| + MAGUK\_N\_PEST |  |  |  |  |
| + Med12-PQL |  |  |  |  |
| + Med25\_SD1 |  |  |  |  |
| + MHC\_I | GO:0006955 | immune response | [B] |
|  | GO:0019882 | antigen processing and presentation | [B] |
|  | GO:0016020 | membrane | [C] |
|  | GO:0042612 | MHC class I protein complex | [C] |
| + MHC\_II\_alpha | GO:0006955 | immune response | [B] |
|  | GO:0019882 | antigen processing and presentation | [B] |
|  | GO:0016020 | membrane | [C] |
|  | GO:0042613 | MHC class II protein complex | [C] |
| + MHC\_II\_beta | GO:0006955 | immune response | [B] |
|  | GO:0019882 | antigen processing and presentation | [B] |
|  | GO:0016020 | membrane | [C] |
|  | GO:0042613 | MHC class II protein complex | [C] |
| + MHCassoc\_trimer | GO:0042289 | MHC class II protein binding | [M] |
|  | GO:0006886 | intracellular protein transport | [B] |
|  | GO:0006955 | immune response | [B] |
|  | GO:0019882 | antigen processing and presentation | [B] |
|  | GO:0016020 | membrane | [C] |
| + Mit\_proteolip | GO:0005739 | mitochondrion | [C] |
| + MOBP\_C-Myrip |  |  |  |  |
| + Motilin\_assoc | GO:0005179 | hormone activity | [M] |
|  | GO:0005576 | extracellular region | [C] |
| + Myc-LZ | GO:0003700 | transcription factor activity | [M] |
|  | GO:0006355 | regulation of transcription, DNA-dependent | [B] |
|  | GO:0005634 | nucleus | [C] |
| + Myelin-PO\_C |  |  |  |  |
| + Myelin\_MBP | GO:0019911 | structural constituent of myelin sheath | [M] |
| + Nab1 | GO:0016564 | transcription repressor activity | [M] |
|  | GO:0016481 | negative regulation of transcription | [B] |
|  | GO:0005634 | nucleus | [C] |
| + Neural\_ProG\_Cyt |  |  |  |  |
| + Neuromodulin\_N |  |  |  |  |
| + NID |  |  |  |  |
| + nlz1 |  |  |  |  |
| + NMDAR2\_C |  |  |  |  |
| + NMU | GO:0006940 | regulation of smooth muscle contraction | [B] |
| + NPP |  |  |  |  |
| + NUC129 | GO:0005634 | nucleus | [C] |
| + Nuc\_rec\_co-act | GO:0003713 | transcription coactivator activity | [M] |
|  | GO:0035257 | nuclear hormone receptor binding | [M] |
|  | GO:0045449 | regulation of transcription | [B] |
|  | GO:0005634 | nucleus | [C] |
| + Oest\_recep | GO:0003677 | DNA binding | [M] |
|  | GO:0003707 | steroid hormone receptor activity | [M] |
|  | GO:0005496 | steroid binding | [M] |
|  | GO:0006355 | regulation of transcription, DNA-dependent | [B] |
|  | GO:0005634 | nucleus | [C] |
| + Olfactory\_mark | GO:0004871 | signal transducer activity | [M] |
|  | GO:0005515 | protein binding | [M] |
|  | GO:0007165 | signal transduction | [B] |
|  | GO:0007608 | sensory perception of smell | [B] |
| + Op\_neuropeptide |  |  |  |  |
| + Opiods\_neuropep | GO:0007218 | neuropeptide signaling pathway | [B] |
| + Orexin\_rec2 | GO:0016499 | orexin receptor activity | [M] |
|  | GO:0007186 | G-protein coupled receptor protein signaling pathway | [B] |
|  | GO:0016021 | integral to membrane | [C] |
| + Osteopontin | GO:0001503 | ossification | [B] |
|  | GO:0007155 | cell adhesion | [B] |
| + Paralemmin | GO:0008360 | regulation of cell shape | [B] |
|  | GO:0016020 | membrane | [C] |
| + Pax7 |  |  |  |  |
| + PD-C2-AF1 |  |  |  |  |
| + PDE6\_gamma | GO:0004114 | 3',5'-cyclic-nucleotide phosphodiesterase activity | [M] |
|  | GO:0030553 | cGMP binding | [M] |
|  | GO:0007601 | visual perception | [B] |
| + PDGF\_N | GO:0008083 | growth factor activity | [M] |
|  | GO:0016020 | membrane | [C] |
| + PDZ\_assoc |  |  |  |  |
| + Peptidase\_S68 |  |  |  |  |
| + Potassium\_chann |  |  |  |  |
| + PPARgamma\_N |  |  |  |  |
| + Pro-MCH | GO:0030354 | melanin-concentrating hormone activity | [M] |
|  | GO:0007268 | synaptic transmission | [B] |
| + Pro-NT\_NN | GO:0005184 | neuropeptide hormone activity | [M] |
|  | GO:0005576 | extracellular region | [C] |
| + Proho\_convert |  |  |  |  |
| + Rb-bdg\_C\_Cenp-F |  |  |  |  |
| + RBM1CTR |  |  |  |  |
| + RecQ5 |  |  |  |  |
| + RELT |  |  |  |  |
| + Rhodopsin\_N |  |  |  |  |
| + RnaseA | GO:0003676 | nucleic acid binding | [M] |
|  | GO:0004522 | pancreatic ribonuclease activity | [M] |
| + ROKNT |  |  |  |  |
| + Serglycin |  |  |  |  |
| + SIM\_C | GO:0003677 | DNA binding | [M] |
|  | GO:0003700 | transcription factor activity | [M] |
|  | GO:0006355 | regulation of transcription, DNA-dependent | [B] |
|  | GO:0005634 | nucleus | [C] |
| + SLY |  |  |  |  |
| + SNN\_cytoplasm |  |  |  |  |
| + SNN\_linker |  |  |  |  |
| + SNN\_transmemb |  |  |  |  |
| + Somatostatin | GO:0005179 | hormone activity | [M] |
|  | GO:0005576 | extracellular region | [C] |
| + Sororin |  |  |  |  |
| + Sorting\_nexin | GO:0008565 | protein transporter activity | [M] |
|  | GO:0006886 | intracellular protein transport | [B] |
| + Sp100 | GO:0005634 | nucleus | [C] |
| + Spp-24 | GO:0046849 | bone remodeling | [B] |
|  | GO:0005576 | extracellular region | [C] |
| + SRC-1 |  |  |  |  |
| + Sushi\_2 |  |  |  |  |
| + Synuclein | GO:0005737 | cytoplasm | [C] |
| + TAFA |  |  |  |  |
| + TAS2R | GO:0004930 | G-protein coupled receptor activity | [M] |
|  | GO:0007186 | G-protein coupled receptor protein signaling pathway | [B] |
|  | GO:0050909 | sensory perception of taste | [B] |
|  | GO:0016021 | integral to membrane | [C] |
| + TAT\_ubiq | GO:0004838 | L-tyrosine:2-oxoglutarate aminotransferase activity | [M] |
|  | GO:0030170 | pyridoxal phosphate binding | [M] |
|  | GO:0009074 | aromatic amino acid family catabolic process | [B] |
| + TBX |  |  |  |  |
| + TCR\_zetazeta |  |  |  |  |
| + Telethonin |  |  |  |  |
| + Telomere\_Pot1 |  |  |  |  |
| + Thrombin\_light | GO:0004252 | serine-type endopeptidase activity | [M] |
|  | GO:0006508 | proteolysis | [B] |
|  | GO:0007596 | blood coagulation | [B] |
|  | GO:0005576 | extracellular region | [C] |
| + Titin\_Z |  |  |  |  |
| + TOH\_N |  |  |  |  |
| + TRADD\_N | GO:0004871 | signal transducer activity | [M] |
|  | GO:0005515 | protein binding | [M] |
|  | GO:0006917 | induction of apoptosis | [B] |
|  | GO:0043123 | positive regulation of I-kappaB kinase/NF-kappaB cascade | [B] |
|  | GO:0005737 | cytoplasm | [C] |
| + UCN2 |  |  |  |  |
| + UPF0258 |  |  |  |  |
| + Uroplakin\_II | GO:0016044 | membrane organization | [B] |
|  | GO:0030176 | integral to endoplasmic reticulum membrane | [C] |
| + Urotensin\_II | GO:0005179 | hormone activity | [M] |
|  | GO:0005576 | extracellular region | [C] |
| + US22 |  |  |  |  |
| + V1R | GO:0016503 | pheromone receptor activity | [M] |
|  | GO:0007186 | G-protein coupled receptor protein signaling pathway | [B] |
|  | GO:0016021 | integral to membrane | [C] |
| + Vert\_IL3-reg\_TF |  |  |  |  |
| + Wos2 | GO:0030528 | transcription regulator activity | [M] |
|  | GO:0045449 | regulation of transcription | [B] |
| + WSK | GO:0005515 | protein binding | [M] |
|  | GO:0006605 | protein targeting | [B] |
|  | GO:0007165 | signal transduction | [B] |
| + Xin | GO:0003779 | actin binding | [M] |
|  | GO:0030036 | actin cytoskeleton organization | [B] |
|  | GO:0030054 | cell junction | [C] |
| + Zfx\_Zfy\_act | GO:0003677 | DNA binding | [M] |
|  | GO:0030528 | transcription regulator activity | [M] |
|  | GO:0046872 | metal ion binding | [M] |
|  | GO:0045449 | regulation of transcription | [B] |
|  | GO:0005634 | nucleus | [C] |

---

## Viridiplantae [eol|tol]

|  |  |  |  |
| --- | --- | --- | --- |
| **Pfam domain(s)** | **GO term acc** | **GO term** | **GO namespace** |
| + Allene\_ox\_cyc | GO:0016853 | isomerase activity | [M] |
|  | GO:0009507 | chloroplast | [C] |
| + Auxin\_BP | GO:0004872 | receptor activity | [M] |
|  | GO:0005788 | endoplasmic reticulum lumen | [C] |
| + Auxin\_repressed |  |  |  |  |
| + B3 | GO:0003677 | DNA binding | [M] |
|  | GO:0006355 | regulation of transcription, DNA-dependent | [B] |
| + Calmodulin\_bind |  |  |  |  |
| + CbiQ | GO:0015087 | cobalt ion transmembrane transporter activity | [M] |
|  | GO:0006824 | cobalt ion transport | [B] |
|  | GO:0009236 | cobalamin biosynthetic process | [B] |
| + COX5C | GO:0004129 | cytochrome-c oxidase activity | [M] |
|  | GO:0055114 | oxidation reduction | [B] |
|  | GO:0005746 | mitochondrial respiratory chain | [C] |
| + D5\_N |  |  |  |  |
| + DUF1138 |  |  |  |  |
| + DUF1313 |  |  |  |  |
| + DUF1664 |  |  |  |  |
| + DUF212 |  |  |  |  |
| + DUF223 |  |  |  |  |
| + DUF2232 |  |  |  |  |
| + DUF2389 |  |  |  |  |
| + DUF3372 |  |  |  |  |
| + DUF3406 |  |  |  |  |
| + DUF3531 |  |  |  |  |
| + DUF3537 |  |  |  |  |
| + DUF3594 |  |  |  |  |
| + DUF3675 |  |  |  |  |
| + DUF3685 |  |  |  |  |
| + DUF3711 |  |  |  |  |
| + DUF3755 |  |  |  |  |
| + DUF502 |  |  |  |  |
| + DUF506 |  |  |  |  |
| + DUF584 |  |  |  |  |
| + DUF615 |  |  |  |  |
| + DUF629 |  |  |  |  |
| + DUF679 |  |  |  |  |
| + DUF760 |  |  |  |  |
| + DUF828 |  |  |  |  |
| + DZC |  |  |  |  |
| + ELK | GO:0003677 | DNA binding | [M] |
|  | GO:0005634 | nucleus | [C] |
| + FBPase\_glpX | GO:0006071 | glycerol metabolic process | [B] |
| + Fer4\_NifH | GO:0005524 | ATP binding | [M] |
|  | GO:0016491 | oxidoreductase activity | [M] |
|  | GO:0055114 | oxidation reduction | [B] |
| + GCS2 | GO:0004357 | glutamate-cysteine ligase activity | [M] |
|  | GO:0006750 | glutathione biosynthetic process | [B] |
| + HR\_lesion |  |  |  |  |
| + KNOX1 | GO:0003677 | DNA binding | [M] |
|  | GO:0005634 | nucleus | [C] |
| + LEA\_5 |  |  |  |  |
| + MDMPI\_N |  |  |  |  |
| + Myosin\_HC-like |  |  |  |  |
| + P-II | GO:0030234 | enzyme regulator activity | [M] |
|  | GO:0006808 | regulation of nitrogen utilization | [B] |
| + Polysacc\_synt | GO:0000271 | polysaccharide biosynthetic process | [B] |
|  | GO:0016020 | membrane | [C] |
| + PRC |  |  |  |  |
| + PsaN | GO:0005515 | protein binding | [M] |
|  | GO:0015979 | photosynthesis | [B] |
|  | GO:0009522 | photosystem I | [C] |
|  | GO:0042651 | thylakoid membrane | [C] |
| + PsbR | GO:0015979 | photosynthesis | [B] |
|  | GO:0009654 | oxygen evolving complex | [C] |
|  | GO:0042651 | thylakoid membrane | [C] |
| + PsbW | GO:0009507 | chloroplast | [C] |
|  | GO:0009523 | photosystem II | [C] |
| + PSI\_PsaH | GO:0015979 | photosynthesis | [B] |
|  | GO:0009538 | photosystem I reaction center | [C] |
| + RcbX |  |  |  |  |
| + RecX | GO:0006282 | regulation of DNA repair | [B] |
| + Rep\_1 | GO:0003677 | DNA binding | [M] |
|  | GO:0006260 | DNA replication | [B] |
|  | GO:0005727 | extrachromosomal circular DNA | [C] |
| + RGP | GO:0008466 | glycogenin glucosyltransferase activity | [M] |
|  | GO:0016758 | transferase activity, transferring hexosyl groups | [M] |
|  | GO:0007047 | cell wall organization | [B] |
|  | GO:0030244 | cellulose biosynthetic process | [B] |
|  | GO:0005794 | Golgi apparatus | [C] |
|  | GO:0009505 | plant-type cell wall | [C] |
|  | GO:0030054 | cell junction | [C] |
| + SBP | GO:0003677 | DNA binding | [M] |
|  | GO:0005634 | nucleus | [C] |
| + Sucrose\_synth | GO:0005985 | sucrose metabolic process | [B] |
| + TniQ |  |  |  |  |
| + TOM20\_plant | GO:0005515 | protein binding | [M] |
|  | GO:0045040 | protein import into mitochondrial outer membrane | [B] |
|  | GO:0005742 | mitochondrial outer membrane translocase complex | [C] |
| + Wax2\_C |  |  |  |  |
| + WRC |  |  |  |  |
| + zf-Dof | GO:0003677 | DNA binding | [M] |
|  | GO:0008270 | zinc ion binding | [M] |
|  | GO:0045449 | regulation of transcription | [B] |

---

## core eudicotyledons [eol|tol]

|  |  |  |  |
| --- | --- | --- | --- |
| **Pfam domain(s)** | **GO term acc** | **GO term** | **GO namespace** |
| + CAP160 |  |  |  |  |
| + DUF2775 |  |  |  |  |
| + DUF577 |  |  |  |  |
| + DUF825 | GO:0005524 | ATP binding | [M] |
|  | GO:0009507 | chloroplast | [C] |
| + LRR\_3 |  |  |  |  |
| + PetL | GO:0009055 | electron carrier activity | [M] |
|  | GO:0009512 | cytochrome b6f complex | [C] |
| + RPW8 |  |  |  |  |
| + Ycf1 |  |  |  |  |
| + Ycf15 |  |  |  |  |

---

## eurosids I [eol|tol]

|  |  |  |  |
| --- | --- | --- | --- |
| **Pfam domain(s)** | **GO term acc** | **GO term** | **GO namespace** |
| + Extensin\_1 | GO:0005199 | structural constituent of cell wall | [M] |

---

## nematode Clade V [eol|tol]

|  |  |  |  |
| --- | --- | --- | --- |
| **Pfam domain(s)** | **GO term acc** | **GO term** | **GO namespace** |
| + 7TM\_GPCR\_Sra | GO:0004888 | transmembrane receptor activity | [M] |
|  | GO:0007606 | sensory perception of chemical stimulus | [B] |
|  | GO:0016021 | integral to membrane | [C] |
| + 7TM\_GPCR\_Srd |  |  |  |  |
| + 7TM\_GPCR\_Srh |  |  |  |  |
| + 7TM\_GPCR\_Sri |  |  |  |  |
| + 7TM\_GPCR\_Srj |  |  |  |  |
| + 7TM\_GPCR\_Srt |  |  |  |  |
| + 7TM\_GPCR\_Str |  |  |  |  |
| + CC |  |  |  |  |
| + DUF1096 |  |  |  |  |
| + DUF1182 |  |  |  |  |
| + DUF13 |  |  |  |  |
| + DUF267 |  |  |  |  |
| + DUF316 |  |  |  |  |
| + DUF621 |  |  |  |  |
| + DUF684 |  |  |  |  |
| + DUF713 |  |  |  |  |
| + DUF780 |  |  |  |  |
| + DUF976 |  |  |  |  |
| + Ins\_beta | GO:0005179 | hormone activity | [M] |
|  | GO:0005576 | extracellular region | [C] |
| + Sre | GO:0004888 | transmembrane receptor activity | [M] |
|  | GO:0007606 | sensory perception of chemical stimulus | [B] |
|  | GO:0016021 | integral to membrane | [C] |
| + Srg | GO:0004888 | transmembrane receptor activity | [M] |
|  | GO:0007606 | sensory perception of chemical stimulus | [B] |
|  | GO:0016020 | membrane | [C] |

---

## rosids [eol|tol]

|  |  |  |  |
| --- | --- | --- | --- |
| **Pfam domain(s)** | **GO term acc** | **GO term** | **GO namespace** |
| + PEARLI-4 |  |  |  |  |
| + SCRL | GO:0007165 | signal transduction | [B] |

---
