# Supplementary material for: Strong functional patterns in the evolution of eukaryotic genomes revealed by the reconstruction of ancestral protein domain repertoires
Source: Genome Biol. 2011 Jan 17;12(1):R4. doi: 10.1186/gb-2011-12-1-r4 (PMC3091302; doi:10.1186/gb-2011-12-1-r4)
Supplement: Additional file 6 — Domain losses and corresponding GO terms during eukaryote evolution. Summary of conditions used: protein predictions as listed in Additional file 1, model of eukaryote evolution as shown in Figure 2 (and more detailed in Additional files 3 and 4), domain models from Pfam 24.0, analyzed with HMMER 3.0b2, Pfam 'gathering' cutoffs, 'pfam2go' mappings dated 2009/10/01. GO namespaces are abbreviated as follows: B, biological process; C, cellular component; M, molecular function. [file gb-2011-12-1-r4-S6.zip › Additional_File_6.html]

Dollo Parsimony | Losses | Domains


# Dollo Parsimony | Losses | Domains

|  |
| --- |
| Aconoidasida |
| Agaricales |
| Agaricomycotina |
| Alveolata |
| Amniota |
| Amoebozoa |
| Annelida |
| Annelida\_Mollusca |
| Apicomplexa |
| Arabidopsis |
| Archaeplastida |
| Arthropoda |
| Ascidiacea |
| Ascomycota |
| Aspergillus |
| Bacillariophycidae |
| Bacillariophyta |
| Basidiomycota |
| Bikonta |
| Bilateria |
| Bilateria\_Cnidaria |
| Caenorhabditis |
| Chaetomiaceae |
| Chlorophyceae |
| Chlorophyta |
| Chordata |
| Chromalveolate |
| Ciliophora |
| Coccidia |
| Corticata |
| Cryptosporidium |
| Culicoidea |
| Deuterostomia |
| Diapsida |
| Dictyostelium |
| Dikarya |
| Dikarya\_Mucoromycotina |
| Diptera |
| Dothideomycetes |
| Ecdysozoa |
| Embryophyta |
| Euarchontoglires |
| Eukaryota |
| Eurotiales |
| Euteleostei |
| Eutheria |
| Excavata |
| Fungi |
| Heterokonta |
| Heterokonta\_Alveolata |
| Homobasidiomycetes |
| Hymenoptera |
| Hypocreales |
| Insecta |
| Kinetoplastida |
| Kinetoplastida\_Heterolobosea |
| Lepidoptera\_Diptera |
| Lepidoptera\_Diptera\_Hymenoptera |
| Lophotrochozoa |
| Magnaporthales\_Hypocreales |
| Magnoliophyta |
| Mammalia |
| Metamonada |
| Metazoa |
| Metazoa\_Choanoflagellata |
| Micromonas |
| Mucoromycotina |
| Muscomorpha |
| Mycosphaerella |
| Nematoda |
| Neosartorya\_Emericella |
| Onygenales |
| Oomycetes |
| Opisthokonta |
| Ostreococcus |
| Pelagophyceae\_Bacillariophyta |
| Pezizomycotina |
| Plasmodium |
| Pleosporaceae |
| Pleosporales |
| Poales |
| Prasinophyceae |
| Primates |
| Protostomia |
| Pucciniomycetes |
| Pucciniomycotina |
| Pucciniomycotina\_Agaricomycotina |
| Rodentia |
| Saccharomycetaceae |
| Saccharomycotina |
| Saccharomycotina\_Taphrinomycotina |
| Smegmamorpha |
| Sordariales |
| Sordariomycetes |
| Sordariomycetes\_Dothideomycetes |
| Sordariomycetes\_Dothideomycetes\_Eurotiales |
| Taphrinomycotina |
| Teleostei |
| Tetraodontiformes |
| Tetrapoda |
| Theileria |
| Tracheophyta |
| Trebouxiophyceae\_Chlorophyceae |
| Tremellomycetes |
| Unikonta |
| Urochordata |
| Urochordata\_Vertebrata |
| Vertebrata |
| Viridiplantae |
| core eudicotyledons |
| eurosids I |
| nematode Clade V |
| rosids |

## Aconoidasida [eol|tol]

|  |  |  |  |
| --- | --- | --- | --- |
| **Pfam domain(s)** | **GO term acc** | **GO term** | **GO namespace** |
| - 2-Hacid\_dh\_C | GO:0016616 | oxidoreductase activity, acting on the CH-OH group of donors, NAD or NADP as acceptor | [M] |
|  | GO:0048037 | cofactor binding | [M] |
| - 3HCDH | GO:0016491 | oxidoreductase activity | [M] |
|  | GO:0006631 | fatty acid metabolic process | [B] |
| - 3HCDH\_N | GO:0016491 | oxidoreductase activity | [M] |
|  | GO:0006631 | fatty acid metabolic process | [B] |
| - 5\_nucleotid\_C | GO:0016787 | hydrolase activity | [M] |
|  | GO:0009166 | nucleotide catabolic process | [B] |
| - A\_deamin | GO:0003723 | RNA binding | [M] |
|  | GO:0004000 | adenosine deaminase activity | [M] |
|  | GO:0006396 | RNA processing | [B] |
| - AA\_kinase | GO:0008652 | cellular amino acid biosynthetic process | [B] |
| - ABC\_membrane\_2 | GO:0006810 | transport | [B] |
|  | GO:0016020 | membrane | [C] |
| - ABC\_transp\_aux |  |  |  |  |
| - ACC\_central | GO:0003989 | acetyl-CoA carboxylase activity | [M] |
|  | GO:0005524 | ATP binding | [M] |
|  | GO:0006633 | fatty acid biosynthetic process | [B] |
| - ACOX | GO:0003997 | acyl-CoA oxidase activity | [M] |
|  | GO:0006635 | fatty acid beta-oxidation | [B] |
|  | GO:0055114 | oxidation reduction | [B] |
|  | GO:0005777 | peroxisome | [C] |
| - ACT | GO:0016597 | amino acid binding | [M] |
|  | GO:0008152 | metabolic process | [B] |
| - Acyl-CoA\_dh\_1 | GO:0016627 | oxidoreductase activity, acting on the CH-CH group of donors | [M] |
|  | GO:0055114 | oxidation reduction | [B] |
| - Acyl-CoA\_dh\_M | GO:0003995 | acyl-CoA dehydrogenase activity | [M] |
|  | GO:0055114 | oxidation reduction | [B] |
| - Acyl-CoA\_dh\_N | GO:0003995 | acyl-CoA dehydrogenase activity | [M] |
|  | GO:0055114 | oxidation reduction | [B] |
| - Acyl\_transf\_3 | GO:0016747 | transferase activity, transferring acyl groups other than amino-acyl groups | [M] |
| - AIG2 |  |  |  |  |
| - Aldedh | GO:0016491 | oxidoreductase activity | [M] |
|  | GO:0008152 | metabolic process | [B] |
|  | GO:0055114 | oxidation reduction | [B] |
| - Alg6\_Alg8 | GO:0016758 | transferase activity, transferring hexosyl groups | [M] |
|  | GO:0005789 | endoplasmic reticulum membrane | [C] |
| - Alpha-amyl\_C2 | GO:0004556 | alpha-amylase activity | [M] |
|  | GO:0005509 | calcium ion binding | [M] |
|  | GO:0005975 | carbohydrate metabolic process | [B] |
| - Alpha-amylase | GO:0003824 | catalytic activity | [M] |
|  | GO:0043169 | cation binding | [M] |
|  | GO:0005975 | carbohydrate metabolic process | [B] |
| - Alpha-amylase\_C | GO:0003824 | catalytic activity | [M] |
|  | GO:0043169 | cation binding | [M] |
|  | GO:0005975 | carbohydrate metabolic process | [B] |
| - Alpha\_adaptin\_C | GO:0005515 | protein binding | [M] |
|  | GO:0006886 | intracellular protein transport | [B] |
|  | GO:0016192 | vesicle-mediated transport | [B] |
|  | GO:0030131 | clathrin adaptor complex | [C] |
| - AMP\_N | GO:0004177 | aminopeptidase activity | [M] |
|  | GO:0030145 | manganese ion binding | [M] |
| - AOX | GO:0007585 | respiratory gaseous exchange | [B] |
|  | GO:0055114 | oxidation reduction | [B] |
|  | GO:0005740 | mitochondrial envelope | [C] |
| - ApbA |  |  |  |  |
| - ApbA\_C | GO:0016491 | oxidoreductase activity | [M] |
|  | GO:0050661 | NADP or NADPH binding | [M] |
|  | GO:0055114 | oxidation reduction | [B] |
| - APC8 | GO:0030071 | regulation of mitotic metaphase/anaphase transition | [B] |
|  | GO:0005680 | anaphase-promoting complex | [C] |
| - APG6 | GO:0006914 | autophagy | [B] |
| - APG9 |  |  |  |  |
| - APS\_kinase | GO:0005524 | ATP binding | [M] |
|  | GO:0016301 | kinase activity | [M] |
|  | GO:0016772 | transferase activity, transferring phosphorus-containing groups | [M] |
|  | GO:0000103 | sulfate assimilation | [B] |
| - Apt1 |  |  |  |  |
| - Apyrase | GO:0005509 | calcium ion binding | [M] |
|  | GO:0016462 | pyrophosphatase activity | [M] |
| - ARPC4 | GO:0030041 | actin filament polymerization | [B] |
|  | GO:0005856 | cytoskeleton | [C] |
| - Arrestin\_C |  |  |  |  |
| - Arrestin\_N |  |  |  |  |
| - ARS2 |  |  |  |  |
| - ASCH |  |  |  |  |
| - AsnA | GO:0004071 | aspartate-ammonia ligase activity | [M] |
|  | GO:0006529 | asparagine biosynthetic process | [B] |
|  | GO:0005737 | cytoplasm | [C] |
| - Asparaginase | GO:0006520 | cellular amino acid metabolic process | [B] |
| - ATG\_C |  |  |  |  |
| - ATP-gua\_Ptrans | GO:0016301 | kinase activity | [M] |
|  | GO:0016772 | transferase activity, transferring phosphorus-containing groups | [M] |
| - ATP-sulfurylase | GO:0004781 | sulfate adenylyltransferase (ATP) activity | [M] |
|  | GO:0000103 | sulfate assimilation | [B] |
| - Auxin\_resp | GO:0003677 | DNA binding | [M] |
|  | GO:0009725 | response to hormone stimulus | [B] |
|  | GO:0045449 | regulation of transcription | [B] |
|  | GO:0005634 | nucleus | [C] |
| - B9 |  |  |  |  |
| - Bap31 | GO:0006886 | intracellular protein transport | [B] |
|  | GO:0005783 | endoplasmic reticulum | [C] |
|  | GO:0016021 | integral to membrane | [C] |
| - BCDHK\_Adom3 |  |  |  |  |
| - Bestrophin |  |  |  |  |
| - BHD\_2 | GO:0003684 | damaged DNA binding | [M] |
|  | GO:0006289 | nucleotide-excision repair | [B] |
|  | GO:0005634 | nucleus | [C] |
| - Biopterin\_H | GO:0016714 | oxidoreductase activity, acting on paired donors, with incorporation or reduction of molecular oxygen, reduced pteridine as one donor, and incorporation of one atom of oxygen | [M] |
|  | GO:0055114 | oxidation reduction | [B] |
| - BPL\_N |  |  |  |  |
| - BRCA-2\_OB1 |  |  |  |  |
| - BRCA2 |  |  |  |  |
| - But2 |  |  |  |  |
| - C1\_1 | GO:0007242 | intracellular signaling cascade | [B] |
| - Calreticulin | GO:0005509 | calcium ion binding | [M] |
| - CAP |  |  |  |  |
| - CASP\_C | GO:0006891 | intra-Golgi vesicle-mediated transport | [B] |
|  | GO:0030173 | integral to Golgi membrane | [C] |
| - Catalase |  |  |  |  |
| - Catalase-rel | GO:0004096 | catalase activity | [M] |
|  | GO:0005506 | iron ion binding | [M] |
|  | GO:0020037 | heme binding | [M] |
|  | GO:0006979 | response to oxidative stress | [B] |
|  | GO:0055114 | oxidation reduction | [B] |
| - CBM\_48 | GO:0004553 | hydrolase activity, hydrolyzing O-glycosyl compounds | [M] |
|  | GO:0005975 | carbohydrate metabolic process | [B] |
| - CDC45 | GO:0006270 | DNA replication initiation | [B] |
| - Choline\_transpo |  |  |  |  |
| - CHORD |  |  |  |  |
| - Clp1 |  |  |  |  |
| - Cluap1 |  |  |  |  |
| - CN\_hydrolase | GO:0016810 | hydrolase activity, acting on carbon-nitrogen (but not peptide) bonds | [M] |
|  | GO:0006807 | nitrogen compound metabolic process | [B] |
| - COesterase |  |  |  |  |
| - Condensation |  |  |  |  |
| - COPIIcoated\_ERV |  |  |  |  |
| - CRCB | GO:0016020 | membrane | [C] |
| - Cript |  |  |  |  |
| - CRT-like |  |  |  |  |
| - Ctf8 |  |  |  |  |
| - Cu\_amine\_oxid | GO:0005507 | copper ion binding | [M] |
|  | GO:0008131 | amine oxidase activity | [M] |
|  | GO:0048038 | quinone binding | [M] |
|  | GO:0009308 | amine metabolic process | [B] |
|  | GO:0055114 | oxidation reduction | [B] |
| - Cyclin\_C | GO:0005634 | nucleus | [C] |
| - Cys\_Met\_Meta\_PP | GO:0030170 | pyridoxal phosphate binding | [M] |
|  | GO:0006520 | cellular amino acid metabolic process | [B] |
| - Dabb |  |  |  |  |
| - DAD |  |  |  |  |
| - DAGAT | GO:0016747 | transferase activity, transferring acyl groups other than amino-acyl groups | [M] |
| - DAHP\_synth\_2 | GO:0003849 | 3-deoxy-7-phosphoheptulonate synthase activity | [M] |
|  | GO:0009073 | aromatic amino acid family biosynthetic process | [B] |
| - DapB\_C | GO:0008839 | dihydrodipicolinate reductase activity | [M] |
|  | GO:0009089 | lysine biosynthetic process via diaminopimelate | [B] |
|  | GO:0055114 | oxidation reduction | [B] |
| - DapB\_N | GO:0008839 | dihydrodipicolinate reductase activity | [M] |
|  | GO:0009089 | lysine biosynthetic process via diaminopimelate | [B] |
|  | GO:0055114 | oxidation reduction | [B] |
| - DBP10CT | GO:0003723 | RNA binding | [M] |
|  | GO:0005524 | ATP binding | [M] |
|  | GO:0016818 | hydrolase activity, acting on acid anhydrides, in phosphorus-containing anhydrides | [M] |
|  | GO:0005634 | nucleus | [C] |
| - DegT\_DnrJ\_EryC1 |  |  |  |  |
| - DEP | GO:0007242 | intracellular signaling cascade | [B] |
| - DHDPS | GO:0016829 | lyase activity | [M] |
|  | GO:0008152 | metabolic process | [B] |
| - DHquinase\_I | GO:0003855 | 3-dehydroquinate dehydratase activity | [M] |
| - DIE2\_ALG10 | GO:0016758 | transferase activity, transferring hexosyl groups | [M] |
|  | GO:0016021 | integral to membrane | [C] |
| - DivIC | GO:0007049 | cell cycle | [B] |
| - DNA\_ligase\_OB | GO:0003911 | DNA ligase (NAD+) activity | [M] |
|  | GO:0006260 | DNA replication | [B] |
|  | GO:0006281 | DNA repair | [B] |
| - DNA\_pol3\_delta | GO:0003677 | DNA binding | [M] |
|  | GO:0003887 | DNA-directed DNA polymerase activity | [M] |
|  | GO:0006260 | DNA replication | [B] |
|  | GO:0009360 | DNA polymerase III complex | [C] |
| - DNA\_pol\_delta\_4 | GO:0006260 | DNA replication | [B] |
|  | GO:0005634 | nucleus | [C] |
| - DNA\_pol\_phi | GO:0003677 | DNA binding | [M] |
|  | GO:0003887 | DNA-directed DNA polymerase activity | [M] |
|  | GO:0006350 | transcription | [B] |
| - Dor1 |  |  |  |  |
| - DPM3 |  |  |  |  |
| - DPPIV\_N | GO:0006508 | proteolysis | [B] |
|  | GO:0016020 | membrane | [C] |
| - DRIM |  |  |  |  |
| - DRY\_EERY |  |  |  |  |
| - DSS1\_SEM1 |  |  |  |  |
| - DTW |  |  |  |  |
| - DUF1032 |  |  |  |  |
| - DUF1126 |  |  |  |  |
| - DUF1298 |  |  |  |  |
| - DUF1635 |  |  |  |  |
| - DUF1681 | GO:0006897 | endocytosis | [B] |
|  | GO:0016020 | membrane | [C] |
| - DUF1731 |  |  |  |  |
| - DUF1749 |  |  |  |  |
| - DUF1765 |  |  |  |  |
| - DUF1767 |  |  |  |  |
| - DUF1769 |  |  |  |  |
| - DUF1981 |  |  |  |  |
| - DUF2036 |  |  |  |  |
| - DUF2052 |  |  |  |  |
| - DUF2228 |  |  |  |  |
| - DUF23 |  |  |  |  |
| - DUF2361 |  |  |  |  |
| - DUF2399 |  |  |  |  |
| - DUF2450 |  |  |  |  |
| - DUF2763 |  |  |  |  |
| - DUF298 |  |  |  |  |
| - DUF3469 |  |  |  |  |
| - DUF3522 |  |  |  |  |
| - DUF3608 |  |  |  |  |
| - DUF3633 |  |  |  |  |
| - DUF3657 |  |  |  |  |
| - DUF3689 |  |  |  |  |
| - DUF3712 |  |  |  |  |
| - DUF384 |  |  |  |  |
| - DUF547 |  |  |  |  |
| - DUF74 |  |  |  |  |
| - DUF775 |  |  |  |  |
| - DUF789 |  |  |  |  |
| - DUF791 |  |  |  |  |
| - DUF818 |  |  |  |  |
| - DUF821 |  |  |  |  |
| - DUF872 |  |  |  |  |
| - Dymeclin |  |  |  |  |
| - E1\_DerP2\_DerF2 |  |  |  |  |
| - E2\_bind | GO:0005524 | ATP binding | [M] |
|  | GO:0016881 | acid-amino acid ligase activity | [M] |
|  | GO:0045116 | protein neddylation | [B] |
| - E2F\_TDP | GO:0003700 | transcription factor activity | [M] |
|  | GO:0006355 | regulation of transcription, DNA-dependent | [B] |
|  | GO:0005667 | transcription factor complex | [C] |
| - E3\_binding | GO:0005515 | protein binding | [M] |
|  | GO:0008415 | acyltransferase activity | [M] |
|  | GO:0008152 | metabolic process | [B] |
| - efhand\_like |  |  |  |  |
| - efThoc1 |  |  |  |  |
| - EGF\_2 |  |  |  |  |
| - EGF\_CA |  |  |  |  |
| - EI24 |  |  |  |  |
| - EKR | GO:0016903 | oxidoreductase activity, acting on the aldehyde or oxo group of donors | [M] |
|  | GO:0055114 | oxidation reduction | [B] |
| - Endonuclease\_NS | GO:0003676 | nucleic acid binding | [M] |
|  | GO:0016787 | hydrolase activity | [M] |
|  | GO:0046872 | metal ion binding | [M] |
| - EnY2 |  |  |  |  |
| - ETF |  |  |  |  |
| - ETF\_alpha |  |  |  |  |
| - ETF\_QO | GO:0004174 | electron-transferring-flavoprotein dehydrogenase activity | [M] |
|  | GO:0055114 | oxidation reduction | [B] |
| - Exostosin | GO:0016020 | membrane | [C] |
| - FA\_hydroxylase | GO:0005506 | iron ion binding | [M] |
|  | GO:0016491 | oxidoreductase activity | [M] |
|  | GO:0006633 | fatty acid biosynthetic process | [B] |
|  | GO:0055114 | oxidation reduction | [B] |
|  | GO:0005783 | endoplasmic reticulum | [C] |
| - FATC |  |  |  |  |
| - FBPase | GO:0042578 | phosphoric ester hydrolase activity | [M] |
|  | GO:0005975 | carbohydrate metabolic process | [B] |
| - Fe-ADH | GO:0016491 | oxidoreductase activity | [M] |
|  | GO:0046872 | metal ion binding | [M] |
|  | GO:0055114 | oxidation reduction | [B] |
| - FimP |  |  |  |  |
| - FKBP\_N | GO:0006457 | protein folding | [B] |
| - fn3 |  |  |  |  |
| - Folate\_rec |  |  |  |  |
| - GalKase\_gal\_bdg |  |  |  |  |
| - GBP\_C | GO:0003924 | GTPase activity | [M] |
|  | GO:0005525 | GTP binding | [M] |
| - GCFC | GO:0003677 | DNA binding | [M] |
|  | GO:0003700 | transcription factor activity | [M] |
|  | GO:0045449 | regulation of transcription | [B] |
|  | GO:0005634 | nucleus | [C] |
| - GDE\_C | GO:0004135 | amylo-alpha-1,6-glucosidase activity | [M] |
|  | GO:0005978 | glycogen biosynthetic process | [B] |
| - GFO\_IDH\_MocA | GO:0016491 | oxidoreductase activity | [M] |
| - GHMP\_kinases\_C |  |  |  |  |
| - GHMP\_kinases\_N | GO:0005524 | ATP binding | [M] |
|  | GO:0016301 | kinase activity | [M] |
|  | GO:0016310 | phosphorylation | [B] |
| - GILT |  |  |  |  |
| - GlcNAc |  |  |  |  |
| - Glucan\_synthase | GO:0003843 | 1,3-beta-glucan synthase activity | [M] |
|  | GO:0006075 | 1,3-beta-glucan biosynthetic process | [B] |
|  | GO:0000148 | 1,3-beta-glucan synthase complex | [C] |
|  | GO:0016020 | membrane | [C] |
| - Gly\_transf\_sug |  |  |  |  |
| - Glyco\_hydro\_17 | GO:0004553 | hydrolase activity, hydrolyzing O-glycosyl compounds | [M] |
|  | GO:0005975 | carbohydrate metabolic process | [B] |
| - Glyco\_hydro\_19 | GO:0004568 | chitinase activity | [M] |
|  | GO:0006032 | chitin catabolic process | [B] |
|  | GO:0016998 | cell wall macromolecule catabolic process | [B] |
| - Glyco\_hydro\_31 | GO:0004553 | hydrolase activity, hydrolyzing O-glycosyl compounds | [M] |
|  | GO:0005975 | carbohydrate metabolic process | [B] |
| - Glyco\_hydro\_63 | GO:0004573 | mannosyl-oligosaccharide glucosidase activity | [M] |
|  | GO:0009311 | oligosaccharide metabolic process | [B] |
| - Glyco\_hydro\_77 | GO:0004134 | 4-alpha-glucanotransferase activity | [M] |
|  | GO:0005975 | carbohydrate metabolic process | [B] |
| - Glyco\_hydro\_81 | GO:0033903 | endo-1,3(4)-beta-glucanase activity | [M] |
|  | GO:0016998 | cell wall macromolecule catabolic process | [B] |
| - Glyco\_transf\_17 | GO:0003830 | beta-1,4-mannosylglycoprotein 4-beta-N-acetylglucosaminyltransferase activity | [M] |
|  | GO:0006487 | protein amino acid N-linked glycosylation | [B] |
|  | GO:0016020 | membrane | [C] |
| - Glyco\_transf\_5 |  |  |  |  |
| - Glyco\_transf\_8 | GO:0016757 | transferase activity, transferring glycosyl groups | [M] |
| - GMC\_oxred\_C | GO:0016614 | oxidoreductase activity, acting on CH-OH group of donors | [M] |
| - GMC\_oxred\_N | GO:0016614 | oxidoreductase activity, acting on CH-OH group of donors | [M] |
|  | GO:0050660 | FAD binding | [M] |
| - GRIP |  |  |  |  |
| - GSH\_synthase | GO:0004363 | glutathione synthase activity | [M] |
|  | GO:0005524 | ATP binding | [M] |
|  | GO:0006750 | glutathione biosynthetic process | [B] |
| - Gtr1\_RagA | GO:0005525 | GTP binding | [M] |
|  | GO:0005634 | nucleus | [C] |
|  | GO:0005737 | cytoplasm | [C] |
| - HAT | GO:0006396 | RNA processing | [B] |
|  | GO:0005622 | intracellular | [C] |
| - HDA2-3 |  |  |  |  |
| - HDAC\_interact |  |  |  |  |
| - HHH | GO:0003677 | DNA binding | [M] |
|  | GO:0005622 | intracellular | [C] |
| - HI0933\_like |  |  |  |  |
| - Hint | GO:0008233 | peptidase activity | [M] |
|  | GO:0006508 | proteolysis | [B] |
| - HisKA | GO:0000155 | two-component sensor activity | [M] |
|  | GO:0007165 | signal transduction | [B] |
|  | GO:0016020 | membrane | [C] |
| - HMA | GO:0046872 | metal ion binding | [M] |
|  | GO:0030001 | metal ion transport | [B] |
| - HMGL-like | GO:0003824 | catalytic activity | [M] |
| - HOOK | GO:0008017 | microtubule binding | [M] |
|  | GO:0000226 | microtubule cytoskeleton organization | [B] |
|  | GO:0005737 | cytoplasm | [C] |
| - HSCB\_C | GO:0005515 | protein binding | [M] |
|  | GO:0006457 | protein folding | [B] |
| - Hydroxy-O-Methy |  |  |  |  |
| - ICL | GO:0003824 | catalytic activity | [M] |
|  | GO:0008152 | metabolic process | [B] |
| - Inositol\_P | GO:0004437 | inositol or phosphatidylinositol phosphatase activity | [M] |
| - Ion\_trans | GO:0005216 | ion channel activity | [M] |
|  | GO:0006811 | ion transport | [B] |
|  | GO:0055085 | transmembrane transport | [B] |
|  | GO:0016020 | membrane | [C] |
| - IRK |  |  |  |  |
| - Ist1 |  |  |  |  |
| - Kazal\_1 |  |  |  |  |
| - Kazal\_2 |  |  |  |  |
| - KIF1B |  |  |  |  |
| - KR |  |  |  |  |
| - Ku | GO:0003677 | DNA binding | [M] |
|  | GO:0004003 | ATP-dependent DNA helicase activity | [M] |
|  | GO:0006303 | double-strand break repair via nonhomologous end joining | [B] |
| - Ku\_N |  |  |  |  |
| - Ku\_PK\_bind | GO:0003677 | DNA binding | [M] |
|  | GO:0004003 | ATP-dependent DNA helicase activity | [M] |
|  | GO:0006303 | double-strand break repair via nonhomologous end joining | [B] |
|  | GO:0005634 | nucleus | [C] |
| - Lectin\_C | GO:0005488 | binding | [M] |
| - Leo1 |  |  |  |  |
| - Lipid\_DES | GO:0016705 | oxidoreductase activity, acting on paired donors, with incorporation or reduction of molecular oxygen | [M] |
|  | GO:0006633 | fatty acid biosynthetic process | [B] |
|  | GO:0055114 | oxidation reduction | [B] |
|  | GO:0016021 | integral to membrane | [C] |
| - LMBR1 |  |  |  |  |
| - Malectin |  |  |  |  |
| - malic | GO:0016616 | oxidoreductase activity, acting on the CH-OH group of donors, NAD or NADP as acceptor | [M] |
| - Malic\_M | GO:0016616 | oxidoreductase activity, acting on the CH-OH group of donors, NAD or NADP as acceptor | [M] |
|  | GO:0051287 | NAD or NADH binding | [M] |
| - MAM | GO:0016020 | membrane | [C] |
| - MaoC\_dehydratas | GO:0016491 | oxidoreductase activity | [M] |
|  | GO:0008152 | metabolic process | [B] |
| - MAPEG |  |  |  |  |
| - Mcm10 |  |  |  |  |
| - Mec-17 |  |  |  |  |
| - Med14 |  |  |  |  |
| - Med21 |  |  |  |  |
| - Melibiase | GO:0004553 | hydrolase activity, hydrolyzing O-glycosyl compounds | [M] |
|  | GO:0005975 | carbohydrate metabolic process | [B] |
| - Mesd |  |  |  |  |
| - MIF4G\_like | GO:0005515 | protein binding | [M] |
|  | GO:0016070 | RNA metabolic process | [B] |
| - MIP-T3 |  |  |  |  |
| - MmgE\_PrpD | GO:0047547 | 2-methylcitrate dehydratase activity | [M] |
|  | GO:0019543 | propionate catabolic process | [B] |
| - Mmp37 |  |  |  |  |
| - MMR\_HSR1\_C |  |  |  |  |
| - Mo-co\_dimer | GO:0016491 | oxidoreductase activity | [M] |
|  | GO:0030151 | molybdenum ion binding | [M] |
|  | GO:0055114 | oxidation reduction | [B] |
| - Mo25 |  |  |  |  |
| - MoaC | GO:0006777 | Mo-molybdopterin cofactor biosynthetic process | [B] |
| - MoaE | GO:0006777 | Mo-molybdopterin cofactor biosynthetic process | [B] |
| - Mob1\_phocein | GO:0005515 | protein binding | [M] |
| - Mob\_synth\_C | GO:0051539 | 4 iron, 4 sulfur cluster binding | [M] |
|  | GO:0006777 | Mo-molybdopterin cofactor biosynthetic process | [B] |
|  | GO:0019008 | molybdopterin synthase complex | [C] |
| - MoCF\_biosynth | GO:0006777 | Mo-molybdopterin cofactor biosynthetic process | [B] |
| - MoeA\_N | GO:0032324 | molybdopterin cofactor biosynthetic process | [B] |
| - MRG | GO:0005634 | nucleus | [C] |
| - MSC |  |  |  |  |
| - Mtc | GO:0008324 | cation transmembrane transporter activity | [M] |
|  | GO:0006812 | cation transport | [B] |
|  | GO:0055085 | transmembrane transport | [B] |
|  | GO:0016020 | membrane | [C] |
| - Myotub-related | GO:0016791 | phosphatase activity | [M] |
|  | GO:0016311 | dephosphorylation | [B] |
| - MyTH4 | GO:0005856 | cytoskeleton | [C] |
| - Na\_sulph\_symp | GO:0005215 | transporter activity | [M] |
|  | GO:0006814 | sodium ion transport | [B] |
|  | GO:0055085 | transmembrane transport | [B] |
|  | GO:0016020 | membrane | [C] |
| - NAD\_binding\_4 |  |  |  |  |
| - NatB\_MDM20 |  |  |  |  |
| - NC |  |  |  |  |
| - Nic96 | GO:0006810 | transport | [B] |
|  | GO:0005643 | nuclear pore | [C] |
| - NOC3p |  |  |  |  |
| - Nop14 |  |  |  |  |
| - Nop16 |  |  |  |  |
| - Nop25 |  |  |  |  |
| - Not3 | GO:0030528 | transcription regulator activity | [M] |
|  | GO:0045449 | regulation of transcription | [B] |
|  | GO:0005634 | nucleus | [C] |
| - Notch | GO:0030154 | cell differentiation | [B] |
|  | GO:0016020 | membrane | [C] |
| - NPR2 |  |  |  |  |
| - NUC130\_3NT |  |  |  |  |
| - Nup96 |  |  |  |  |
| - Ocnus |  |  |  |  |
| - Octopine\_DH | GO:0016491 | oxidoreductase activity | [M] |
|  | GO:0050662 | coenzyme binding | [M] |
|  | GO:0055114 | oxidation reduction | [B] |
| - Oxidored\_molyb | GO:0009055 | electron carrier activity | [M] |
| - p450 | GO:0004497 | monooxygenase activity | [M] |
|  | GO:0005506 | iron ion binding | [M] |
|  | GO:0009055 | electron carrier activity | [M] |
|  | GO:0020037 | heme binding | [M] |
| - PA |  |  |  |  |
| - PA26 | GO:0007050 | cell cycle arrest | [B] |
|  | GO:0005634 | nucleus | [C] |
| - PADR1 | GO:0003950 | NAD+ ADP-ribosyltransferase activity | [M] |
|  | GO:0005634 | nucleus | [C] |
| - PAH | GO:0006355 | regulation of transcription, DNA-dependent | [B] |
|  | GO:0005634 | nucleus | [C] |
| - PAL | GO:0016211 | ammonia ligase activity | [M] |
|  | GO:0009058 | biosynthetic process | [B] |
| - PALP | GO:0003824 | catalytic activity | [M] |
|  | GO:0030170 | pyridoxal phosphate binding | [M] |
|  | GO:0008152 | metabolic process | [B] |
| - PAM2 |  |  |  |  |
| - PAN\_1 |  |  |  |  |
| - PAN\_3 |  |  |  |  |
| - Pantoate\_ligase | GO:0004592 | pantoate-beta-alanine ligase activity | [M] |
|  | GO:0015940 | pantothenate biosynthetic process | [B] |
| - Pantoate\_transf | GO:0003864 | 3-methyl-2-oxobutanoate hydroxymethyltransferase activity | [M] |
|  | GO:0015940 | pantothenate biosynthetic process | [B] |
| - PAP1 |  |  |  |  |
| - ParcG |  |  |  |  |
| - PARG\_cat | GO:0004649 | poly(ADP-ribose) glycohydrolase activity | [M] |
|  | GO:0005975 | carbohydrate metabolic process | [B] |
| - PARP | GO:0003950 | NAD+ ADP-ribosyltransferase activity | [M] |
| - PARP\_reg | GO:0003950 | NAD+ ADP-ribosyltransferase activity | [M] |
|  | GO:0006471 | protein amino acid ADP-ribosylation | [B] |
| - Penicil\_amidase | GO:0016787 | hydrolase activity | [M] |
|  | GO:0017000 | antibiotic biosynthetic process | [B] |
| - Pentaxin |  |  |  |  |
| - Peptidase\_A17 |  |  |  |  |
| - Peptidase\_C11 |  |  |  |  |
| - Peptidase\_C26 | GO:0016787 | hydrolase activity | [M] |
|  | GO:0006541 | glutamine metabolic process | [B] |
| - Peptidase\_C65 |  |  |  |  |
| - Peptidase\_M13 | GO:0004222 | metalloendopeptidase activity | [M] |
|  | GO:0006508 | proteolysis | [B] |
| - Peptidase\_M13\_N | GO:0008237 | metallopeptidase activity | [M] |
|  | GO:0006508 | proteolysis | [B] |
| - Peptidase\_M28 | GO:0008233 | peptidase activity | [M] |
|  | GO:0006508 | proteolysis | [B] |
| - Peptidase\_M3\_N | GO:0008237 | metallopeptidase activity | [M] |
|  | GO:0008270 | zinc ion binding | [M] |
| - Peptidase\_M54 | GO:0008237 | metallopeptidase activity | [M] |
|  | GO:0008270 | zinc ion binding | [M] |
| - Peptidase\_S15 | GO:0004177 | aminopeptidase activity | [M] |
|  | GO:0006508 | proteolysis | [B] |
| - Peptidase\_S28 | GO:0008236 | serine-type peptidase activity | [M] |
|  | GO:0006508 | proteolysis | [B] |
| - Peptidase\_S58 |  |  |  |  |
| - Peptidase\_S9\_N | GO:0004252 | serine-type endopeptidase activity | [M] |
|  | GO:0006508 | proteolysis | [B] |
| - PepX\_C | GO:0008239 | dipeptidyl-peptidase activity | [M] |
| - Peroxin-3 | GO:0007031 | peroxisome organization | [B] |
|  | GO:0005779 | integral to peroxisomal membrane | [C] |
| - PEX11 | GO:0016559 | peroxisome fission | [B] |
|  | GO:0005778 | peroxisomal membrane | [C] |
| - Pex16 |  |  |  |  |
| - Pex2\_Pex12 | GO:0007031 | peroxisome organization | [B] |
|  | GO:0005778 | peroxisomal membrane | [C] |
| - PfkB |  |  |  |  |
| - PhoPQ\_related |  |  |  |  |
| - Phosphorylase | GO:0004645 | phosphorylase activity | [M] |
|  | GO:0005975 | carbohydrate metabolic process | [B] |
| - PI31\_Prot\_Reg |  |  |  |  |
| - PIG-F | GO:0006506 | GPI anchor biosynthetic process | [B] |
|  | GO:0005789 | endoplasmic reticulum membrane | [C] |
|  | GO:0016021 | integral to membrane | [C] |
| - Piwi |  |  |  |  |
| - PKD\_channel |  |  |  |  |
| - PLDc | GO:0003824 | catalytic activity | [M] |
|  | GO:0008152 | metabolic process | [B] |
| - Plus-3 | GO:0003677 | DNA binding | [M] |
|  | GO:0006352 | transcription initiation | [B] |
|  | GO:0016570 | histone modification | [B] |
|  | GO:0005634 | nucleus | [C] |
| - PMSR | GO:0016671 | oxidoreductase activity, acting on sulfur group of donors, disulfide as acceptor | [M] |
|  | GO:0019538 | protein metabolic process | [B] |
|  | GO:0055114 | oxidation reduction | [B] |
| - Polysacc\_deac\_1 | GO:0016810 | hydrolase activity, acting on carbon-nitrogen (but not peptide) bonds | [M] |
|  | GO:0005975 | carbohydrate metabolic process | [B] |
| - POR | GO:0016903 | oxidoreductase activity, acting on the aldehyde or oxo group of donors | [M] |
|  | GO:0055114 | oxidation reduction | [B] |
| - POR\_N | GO:0016491 | oxidoreductase activity | [M] |
|  | GO:0055114 | oxidation reduction | [B] |
| - PPDK\_N | GO:0005524 | ATP binding | [M] |
|  | GO:0016301 | kinase activity | [M] |
|  | GO:0016310 | phosphorylation | [B] |
| - PRA1 |  |  |  |  |
| - Prenylcys\_lyase | GO:0016670 | oxidoreductase activity, acting on sulfur group of donors, oxygen as acceptor | [M] |
|  | GO:0030328 | prenylcysteine catabolic process | [B] |
|  | GO:0055114 | oxidation reduction | [B] |
| - PRK | GO:0005524 | ATP binding | [M] |
|  | GO:0016301 | kinase activity | [M] |
|  | GO:0008152 | metabolic process | [B] |
| - PRKCSH | GO:0005515 | protein binding | [M] |
| - Pro\_dh | GO:0004657 | proline dehydrogenase activity | [M] |
|  | GO:0006537 | glutamate biosynthetic process | [B] |
|  | GO:0006562 | proline catabolic process | [B] |
|  | GO:0055114 | oxidation reduction | [B] |
| - PS\_pyruv\_trans | GO:0016740 | transferase activity | [M] |
| - PT |  |  |  |  |
| - PTS\_2-RNA | GO:0016772 | transferase activity, transferring phosphorus-containing groups | [M] |
|  | GO:0006388 | tRNA splicing, via endonucleolytic cleavage and ligation | [B] |
| - PYC\_OADA |  |  |  |  |
| - R3H | GO:0003676 | nucleic acid binding | [M] |
| - Rad21\_Rec8 | GO:0000228 | nuclear chromosome | [C] |
| - Rad4 | GO:0003684 | damaged DNA binding | [M] |
|  | GO:0006289 | nucleotide-excision repair | [B] |
|  | GO:0005634 | nucleus | [C] |
| - Rapamycin\_bind | GO:0016772 | transferase activity, transferring phosphorus-containing groups | [M] |
| - Rdx |  |  |  |  |
| - RED\_C |  |  |  |  |
| - Rep\_3 | GO:0003887 | DNA-directed DNA polymerase activity | [M] |
|  | GO:0006270 | DNA replication initiation | [B] |
|  | GO:0005727 | extrachromosomal circular DNA | [C] |
| - Rft-1 | GO:0005319 | lipid transporter activity | [M] |
|  | GO:0006869 | lipid transport | [B] |
|  | GO:0016021 | integral to membrane | [C] |
| - Rgp1 |  |  |  |  |
| - Rho\_N | GO:0003715 | transcription termination factor activity | [M] |
|  | GO:0006353 | transcription termination | [B] |
| - Ribophorin\_II | GO:0004579 | dolichyl-diphosphooligosaccharide-protein glycotransferase activity | [M] |
|  | GO:0018279 | protein amino acid N-linked glycosylation via asparagine | [B] |
|  | GO:0005789 | endoplasmic reticulum membrane | [C] |
|  | GO:0008250 | oligosaccharyltransferase complex | [C] |
| - RIC1 |  |  |  |  |
| - RNA\_pol\_3\_Rpc31 |  |  |  |  |
| - RNA\_pol\_Rpa2\_4 | GO:0003899 | DNA-directed RNA polymerase activity | [M] |
|  | GO:0006350 | transcription | [B] |
|  | GO:0005634 | nucleus | [C] |
| - Rod\_C |  |  |  |  |
| - Rpp20 |  |  |  |  |
| - Rrp15p |  |  |  |  |
| - RuvB\_N | GO:0009378 | four-way junction helicase activity | [M] |
|  | GO:0006281 | DNA repair | [B] |
|  | GO:0006310 | DNA recombination | [B] |
| - S6PP |  |  |  |  |
| - SAPS |  |  |  |  |
| - SbmA\_BacA | GO:0005215 | transporter activity | [M] |
|  | GO:0006810 | transport | [B] |
|  | GO:0009276 | Gram-negative-bacterium-type cell wall | [C] |
|  | GO:0016021 | integral to membrane | [C] |
| - SCP2 | GO:0005498 | sterol carrier activity | [M] |
| - Scs3p |  |  |  |  |
| - SelR | GO:0008113 | peptide-methionine-(S)-S-oxide reductase activity | [M] |
|  | GO:0055114 | oxidation reduction | [B] |
| - Semialdhyde\_dh | GO:0016620 | oxidoreductase activity, acting on the aldehyde or oxo group of donors, NAD or NADP as acceptor | [M] |
|  | GO:0051287 | NAD or NADH binding | [M] |
|  | GO:0006520 | cellular amino acid metabolic process | [B] |
|  | GO:0005737 | cytoplasm | [C] |
| - Semialdhyde\_dhC | GO:0016620 | oxidoreductase activity, acting on the aldehyde or oxo group of donors, NAD or NADP as acceptor | [M] |
|  | GO:0046983 | protein dimerization activity | [M] |
|  | GO:0008652 | cellular amino acid biosynthetic process | [B] |
|  | GO:0005737 | cytoplasm | [C] |
| - Serpin | GO:0004867 | serine-type endopeptidase inhibitor activity | [M] |
| - SGT1 |  |  |  |  |
| - SH3\_2 |  |  |  |  |
| - Shikimate\_dh\_N |  |  |  |  |
| - Sigma70\_r3 | GO:0003677 | DNA binding | [M] |
|  | GO:0003700 | transcription factor activity | [M] |
|  | GO:0016987 | sigma factor activity | [M] |
|  | GO:0006352 | transcription initiation | [B] |
|  | GO:0006355 | regulation of transcription, DNA-dependent | [B] |
| - Siva | GO:0005175 | CD27 receptor binding | [M] |
|  | GO:0043065 | positive regulation of apoptosis | [B] |
|  | GO:0005737 | cytoplasm | [C] |
| - SPT2 |  |  |  |  |
| - SRA1 |  |  |  |  |
| - SSF | GO:0005215 | transporter activity | [M] |
|  | GO:0006810 | transport | [B] |
|  | GO:0055085 | transmembrane transport | [B] |
|  | GO:0016020 | membrane | [C] |
| - STAS |  |  |  |  |
| - Sterol-sensing |  |  |  |  |
| - Stk19 |  |  |  |  |
| - Str\_synth | GO:0016844 | strictosidine synthase activity | [M] |
|  | GO:0009058 | biosynthetic process | [B] |
| - Sugar\_transport | GO:0015144 | carbohydrate transmembrane transporter activity | [M] |
|  | GO:0008643 | carbohydrate transport | [B] |
|  | GO:0016021 | integral to membrane | [C] |
| - Sulfotransfer\_1 | GO:0008146 | sulfotransferase activity | [M] |
| - SURF6 |  |  |  |  |
| - SYS1 |  |  |  |  |
| - TFIID\_20kDa | GO:0016986 | transcription initiation factor activity | [M] |
|  | GO:0006352 | transcription initiation | [B] |
|  | GO:0005669 | transcription factor TFIID complex | [C] |
| - TIG |  |  |  |  |
| - TIP120 |  |  |  |  |
| - TIP\_N |  |  |  |  |
| - TK | GO:0004797 | thymidine kinase activity | [M] |
|  | GO:0005524 | ATP binding | [M] |
| - Tmemb\_14 | GO:0016020 | membrane | [C] |
| - Transaldolase | GO:0005975 | carbohydrate metabolic process | [B] |
| - Transformer | GO:0006397 | mRNA processing | [B] |
|  | GO:0046660 | female sex differentiation | [B] |
|  | GO:0005634 | nucleus | [C] |
| - Translin | GO:0043565 | sequence-specific DNA binding | [M] |
| - Transmemb\_17 |  |  |  |  |
| - Trigger\_N | GO:0006457 | protein folding | [B] |
|  | GO:0015031 | protein transport | [B] |
| - TTKRSYEDQ |  |  |  |  |
| - Tyr-DNA\_phospho | GO:0008081 | phosphoric diester hydrolase activity | [M] |
|  | GO:0006281 | DNA repair | [B] |
|  | GO:0005634 | nucleus | [C] |
| - U3snoRNP10 |  |  |  |  |
| - UDPG\_MGDP\_dh | GO:0016616 | oxidoreductase activity, acting on the CH-OH group of donors, NAD or NADP as acceptor | [M] |
|  | GO:0051287 | NAD or NADH binding | [M] |
|  | GO:0055114 | oxidation reduction | [B] |
| - UDPG\_MGDP\_dh\_C | GO:0016616 | oxidoreductase activity, acting on the CH-OH group of donors, NAD or NADP as acceptor | [M] |
|  | GO:0051287 | NAD or NADH binding | [M] |
|  | GO:0055114 | oxidation reduction | [B] |
| - UDPG\_MGDP\_dh\_N | GO:0016616 | oxidoreductase activity, acting on the CH-OH group of donors, NAD or NADP as acceptor | [M] |
|  | GO:0051287 | NAD or NADH binding | [M] |
|  | GO:0055114 | oxidation reduction | [B] |
| - UPF0016 | GO:0016020 | membrane | [C] |
| - UPF0029 |  |  |  |  |
| - UPF0047 |  |  |  |  |
| - UPF0066 |  |  |  |  |
| - Upf2 |  |  |  |  |
| - Usp | GO:0006950 | response to stress | [B] |
| - VKOR |  |  |  |  |
| - VMA21 |  |  |  |  |
| - Voltage\_CLC | GO:0005247 | voltage-gated chloride channel activity | [M] |
|  | GO:0006821 | chloride transport | [B] |
|  | GO:0055085 | transmembrane transport | [B] |
|  | GO:0016020 | membrane | [C] |
| - Vps54 | GO:0005515 | protein binding | [M] |
|  | GO:0042147 | retrograde transport, endosome to Golgi | [B] |
| - VRR\_NUC |  |  |  |  |
| - WGR |  |  |  |  |
| - WWE |  |  |  |  |
| - Yip1 |  |  |  |  |
| - zf-C4\_Topoisom | GO:0003677 | DNA binding | [M] |
|  | GO:0003916 | DNA topoisomerase activity | [M] |
|  | GO:0006265 | DNA topological change | [B] |
|  | GO:0005694 | chromosome | [C] |
| - zf-GRF | GO:0008270 | zinc ion binding | [M] |
| - zf-LYAR |  |  |  |  |
| - zf-TRAF | GO:0008270 | zinc ion binding | [M] |
| - zf-TRM13\_CCCH |  |  |  |  |
| - Zw10 | GO:0007067 | mitosis | [B] |
|  | GO:0000775 | chromosome, centromeric region | [C] |
|  | GO:0005634 | nucleus | [C] |

---

## Agaricales [eol|tol]

|  |  |  |  |
| --- | --- | --- | --- |
| **Pfam domain(s)** | **GO term acc** | **GO term** | **GO namespace** |
| - AAT | GO:0042318 | penicillin biosynthetic process | [B] |
| - Acylphosphatase |  |  |  |  |
| - AdoHcyase\_NAD |  |  |  |  |
| - AHS1 |  |  |  |  |
| - AHS2 |  |  |  |  |
| - ALMT |  |  |  |  |
| - Arb1 |  |  |  |  |
| - Bac\_rhamnosid |  |  |  |  |
| - Bac\_rhodopsin | GO:0005216 | ion channel activity | [M] |
|  | GO:0006811 | ion transport | [B] |
|  | GO:0016020 | membrane | [C] |
| - ComA | GO:0019295 | coenzyme M biosynthetic process | [B] |
| - Cupin\_5 |  |  |  |  |
| - DNA\_pol\_B\_2 | GO:0000166 | nucleotide binding | [M] |
|  | GO:0003677 | DNA binding | [M] |
|  | GO:0003887 | DNA-directed DNA polymerase activity | [M] |
|  | GO:0008408 | 3'-5' exonuclease activity | [M] |
|  | GO:0006260 | DNA replication | [B] |
| - DUF124 |  |  |  |  |
| - DUF1421 |  |  |  |  |
| - DUF1446 |  |  |  |  |
| - DUF1680 |  |  |  |  |
| - DUF1749 |  |  |  |  |
| - DUF1814 |  |  |  |  |
| - DUF1932 |  |  |  |  |
| - DUF2401 |  |  |  |  |
| - DUF2403 |  |  |  |  |
| - DUF2411 |  |  |  |  |
| - DUF2414 |  |  |  |  |
| - DUF2416 |  |  |  |  |
| - DUF255 |  |  |  |  |
| - DUF3140 |  |  |  |  |
| - DUF3292 |  |  |  |  |
| - DUF336 |  |  |  |  |
| - DUF3474 |  |  |  |  |
| - DUF3767 |  |  |  |  |
| - DUF718 | GO:0016857 | racemase and epimerase activity, acting on carbohydrates and derivatives | [M] |
|  | GO:0019299 | rhamnose metabolic process | [B] |
|  | GO:0005737 | cytoplasm | [C] |
| - DUF917 |  |  |  |  |
| - Erythro\_esteras | GO:0046677 | response to antibiotic | [B] |
| - EST1 |  |  |  |  |
| - EthD |  |  |  |  |
| - FeoB\_N | GO:0005525 | GTP binding | [M] |
|  | GO:0015093 | ferrous iron transmembrane transporter activity | [M] |
|  | GO:0015684 | ferrous iron transport | [B] |
|  | GO:0016021 | integral to membrane | [C] |
| - FmdA\_AmdA | GO:0016811 | hydrolase activity, acting on carbon-nitrogen (but not peptide) bonds, in linear amides | [M] |
|  | GO:0008152 | metabolic process | [B] |
| - Form\_Nir\_trans | GO:0005215 | transporter activity | [M] |
|  | GO:0006810 | transport | [B] |
|  | GO:0016020 | membrane | [C] |
| - Glyco\_hydro\_2\_N | GO:0004553 | hydrolase activity, hydrolyzing O-glycosyl compounds | [M] |
|  | GO:0005975 | carbohydrate metabolic process | [B] |
| - GSPII\_E | GO:0005524 | ATP binding | [M] |
|  | GO:0006810 | transport | [B] |
|  | GO:0005622 | intracellular | [C] |
| - H2TH | GO:0003684 | damaged DNA binding | [M] |
|  | GO:0003906 | DNA-(apurinic or apyrimidinic site) lyase activity | [M] |
|  | GO:0008270 | zinc ion binding | [M] |
|  | GO:0016799 | hydrolase activity, hydrolyzing N-glycosyl compounds | [M] |
|  | GO:0006289 | nucleotide-excision repair | [B] |
| - Ifi-6-16 |  |  |  |  |
| - IIGP | GO:0005525 | GTP binding | [M] |
|  | GO:0016817 | hydrolase activity, acting on acid anhydrides | [M] |
|  | GO:0016020 | membrane | [C] |
| - INO80\_Ies4 |  |  |  |  |
| - iPGM\_N | GO:0004619 | phosphoglycerate mutase activity | [M] |
|  | GO:0030145 | manganese ion binding | [M] |
|  | GO:0006007 | glucose catabolic process | [B] |
|  | GO:0005737 | cytoplasm | [C] |
| - Jacalin |  |  |  |  |
| - LamB\_YcsF |  |  |  |  |
| - Lysine\_decarbox |  |  |  |  |
| - MACPF |  |  |  |  |
| - MgsA\_C |  |  |  |  |
| - MHYT |  |  |  |  |
| - MRJP |  |  |  |  |
| - Mss4 | GO:0005085 | guanyl-nucleotide exchange factor activity | [M] |
|  | GO:0007264 | small GTPase mediated signal transduction | [B] |
| - NAGLU |  |  |  |  |
| - Peptidase\_A4 | GO:0004190 | aspartic-type endopeptidase activity | [M] |
|  | GO:0006508 | proteolysis | [B] |
| - Peptidase\_S26 |  |  |  |  |
| - Pro\_racemase | GO:0018112 | proline racemase activity | [M] |
| - PUB |  |  |  |  |
| - PUCC |  |  |  |  |
| - RNA\_pol\_Rpb1\_7 | GO:0003677 | DNA binding | [M] |
|  | GO:0003899 | DNA-directed RNA polymerase activity | [M] |
|  | GO:0006350 | transcription | [B] |
| - RPE65 |  |  |  |  |
| - RPEL |  |  |  |  |
| - RTC\_insert |  |  |  |  |
| - STE2 | GO:0004932 | mating-type factor pheromone receptor activity | [M] |
|  | GO:0016020 | membrane | [C] |
| - Swi5 |  |  |  |  |
| - Tannase |  |  |  |  |
| - TFIIF\_alpha | GO:0003677 | DNA binding | [M] |
|  | GO:0016563 | transcription activator activity | [M] |
|  | GO:0045941 | positive regulation of transcription | [B] |
|  | GO:0005634 | nucleus | [C] |
| - TIM-br\_sig\_trns |  |  |  |  |
| - Trs120 |  |  |  |  |
| - UMPH-1 | GO:0000287 | magnesium ion binding | [M] |
|  | GO:0008253 | 5'-nucleotidase activity | [M] |
|  | GO:0005737 | cytoplasm | [C] |
| - UNC-93 |  |  |  |  |
| - UPF0089 |  |  |  |  |
| - UPF0261 |  |  |  |  |
| - Y\_phosphatase2 |  |  |  |  |
| - Yos1 |  |  |  |  |

---

## Agaricomycotina [eol|tol]

|  |  |  |  |
| --- | --- | --- | --- |
| **Pfam domain(s)** | **GO term acc** | **GO term** | **GO namespace** |
| - 7tm\_1 | GO:0007186 | G-protein coupled receptor protein signaling pathway | [B] |
|  | GO:0016021 | integral to membrane | [C] |
| - ABC\_ATPase |  |  |  |  |
| - AD |  |  |  |  |
| - ADC | GO:0016831 | carboxy-lyase activity | [M] |
| - AFT |  |  |  |  |
| - Alba | GO:0003676 | nucleic acid binding | [M] |
| - Ald\_Xan\_dh\_C | GO:0016491 | oxidoreductase activity | [M] |
|  | GO:0055114 | oxidation reduction | [B] |
| - Ald\_Xan\_dh\_C2 | GO:0016491 | oxidoreductase activity | [M] |
|  | GO:0055114 | oxidation reduction | [B] |
| - ATP-gua\_PtransN | GO:0016301 | kinase activity | [M] |
|  | GO:0016772 | transferase activity, transferring phosphorus-containing groups | [M] |
| - B\_lectin | GO:0005529 | sugar binding | [M] |
| - Branch | GO:0008375 | acetylglucosaminyltransferase activity | [M] |
|  | GO:0016020 | membrane | [C] |
| - Capsule\_synth | GO:0000271 | polysaccharide biosynthetic process | [B] |
|  | GO:0015774 | polysaccharide transport | [B] |
| - CBM\_19 | GO:0004568 | chitinase activity | [M] |
|  | GO:0006032 | chitin catabolic process | [B] |
| - CDT1 |  |  |  |  |
| - CheR | GO:0008757 | S-adenosylmethionine-dependent methyltransferase activity | [M] |
| - Chitosanase |  |  |  |  |
| - CO\_deh\_flav\_C |  |  |  |  |
| - Copper-bind | GO:0005507 | copper ion binding | [M] |
|  | GO:0009055 | electron carrier activity | [M] |
| - COX2 | GO:0004129 | cytochrome-c oxidase activity | [M] |
|  | GO:0005507 | copper ion binding | [M] |
|  | GO:0016020 | membrane | [C] |
| - COX2\_TM | GO:0004129 | cytochrome-c oxidase activity | [M] |
|  | GO:0005507 | copper ion binding | [M] |
|  | GO:0009055 | electron carrier activity | [M] |
|  | GO:0022900 | electron transport chain | [B] |
|  | GO:0016021 | integral to membrane | [C] |
| - CSD | GO:0003677 | DNA binding | [M] |
|  | GO:0006355 | regulation of transcription, DNA-dependent | [B] |
| - Csm1 |  |  |  |  |
| - Dehydrin | GO:0006950 | response to stress | [B] |
|  | GO:0009415 | response to water | [B] |
| - DGCR6 |  |  |  |  |
| - DHquinase\_II | GO:0003855 | 3-dehydroquinate dehydratase activity | [M] |
| - DUF1388 |  |  |  |  |
| - DUF1519 |  |  |  |  |
| - DUF1624 |  |  |  |  |
| - DUF171 |  |  |  |  |
| - DUF1939 |  |  |  |  |
| - DUF2008 |  |  |  |  |
| - DUF2196 |  |  |  |  |
| - DUF2231 |  |  |  |  |
| - DUF2439 |  |  |  |  |
| - DUF2823 |  |  |  |  |
| - DUF3133 |  |  |  |  |
| - DUF3184 |  |  |  |  |
| - DUF3237 |  |  |  |  |
| - DUF592 | GO:0008270 | zinc ion binding | [M] |
|  | GO:0016811 | hydrolase activity, acting on carbon-nitrogen (but not peptide) bonds, in linear amides | [M] |
|  | GO:0017136 | NAD-dependent histone deacetylase activity | [M] |
|  | GO:0051287 | NAD or NADH binding | [M] |
|  | GO:0006342 | chromatin silencing | [B] |
|  | GO:0006355 | regulation of transcription, DNA-dependent | [B] |
|  | GO:0006476 | protein amino acid deacetylation | [B] |
|  | GO:0045449 | regulation of transcription | [B] |
| - DUF604 |  |  |  |  |
| - DUF612 |  |  |  |  |
| - DUF74 |  |  |  |  |
| - eIF-4B |  |  |  |  |
| - EIF4E-T |  |  |  |  |
| - Endonuclease\_5 | GO:0004519 | endonuclease activity | [M] |
|  | GO:0006281 | DNA repair | [B] |
| - FAD\_binding\_5 | GO:0016491 | oxidoreductase activity | [M] |
|  | GO:0055114 | oxidation reduction | [B] |
| - Fer2\_2 | GO:0016491 | oxidoreductase activity | [M] |
|  | GO:0046872 | metal ion binding | [M] |
|  | GO:0055114 | oxidation reduction | [B] |
| - FRQ |  |  |  |  |
| - GET2 |  |  |  |  |
| - Git3\_C |  |  |  |  |
| - Glyco\_hydro\_26 | GO:0016985 | mannan endo-1,4-beta-mannosidase activity | [M] |
|  | GO:0006080 | substituted mannan metabolic process | [B] |
| - Glyco\_hydro\_32C |  |  |  |  |
| - Glyco\_hydro\_65m | GO:0003824 | catalytic activity | [M] |
|  | GO:0005975 | carbohydrate metabolic process | [B] |
| - Glyco\_hydro\_65N | GO:0003824 | catalytic activity | [M] |
|  | GO:0030246 | carbohydrate binding | [M] |
|  | GO:0005975 | carbohydrate metabolic process | [B] |
| - Glyco\_hydro\_81 | GO:0033903 | endo-1,3(4)-beta-glucanase activity | [M] |
|  | GO:0016998 | cell wall macromolecule catabolic process | [B] |
| - Glyco\_transf\_10 | GO:0008417 | fucosyltransferase activity | [M] |
|  | GO:0006486 | protein amino acid glycosylation | [B] |
|  | GO:0016020 | membrane | [C] |
| - Glyco\_transf\_43 | GO:0015018 | galactosylgalactosylxylosylprotein 3-beta-glucuronosyltransferase activity | [M] |
|  | GO:0016020 | membrane | [C] |
| - HIRA\_B | GO:0003682 | chromatin binding | [M] |
|  | GO:0030528 | transcription regulator activity | [M] |
|  | GO:0006355 | regulation of transcription, DNA-dependent | [B] |
|  | GO:0016568 | chromatin modification | [B] |
|  | GO:0005634 | nucleus | [C] |
| - JTB | GO:0016021 | integral to membrane | [C] |
| - K\_tetra | GO:0005249 | voltage-gated potassium channel activity | [M] |
|  | GO:0006813 | potassium ion transport | [B] |
|  | GO:0008076 | voltage-gated potassium channel complex | [C] |
|  | GO:0016020 | membrane | [C] |
| - LAGLIDADG\_2 | GO:0004519 | endonuclease activity | [M] |
| - LrgB | GO:0016020 | membrane | [C] |
| - Med1 |  |  |  |  |
| - Med8 |  |  |  |  |
| - MIP-T3 |  |  |  |  |
| - Mis14 |  |  |  |  |
| - Mis6 |  |  |  |  |
| - MuDR |  |  |  |  |
| - NADH5\_C | GO:0008137 | NADH dehydrogenase (ubiquinone) activity | [M] |
|  | GO:0042773 | ATP synthesis coupled electron transport | [B] |
|  | GO:0055114 | oxidation reduction | [B] |
| - Octopine\_DH | GO:0016491 | oxidoreductase activity | [M] |
|  | GO:0050662 | coenzyme binding | [M] |
|  | GO:0055114 | oxidation reduction | [B] |
| - Oxidored\_q1 | GO:0008137 | NADH dehydrogenase (ubiquinone) activity | [M] |
|  | GO:0042773 | ATP synthesis coupled electron transport | [B] |
|  | GO:0055114 | oxidation reduction | [B] |
| - Oxidored\_q3 | GO:0008137 | NADH dehydrogenase (ubiquinone) activity | [M] |
|  | GO:0055114 | oxidation reduction | [B] |
| - PAS\_4 |  |  |  |  |
| - PDZ | GO:0005515 | protein binding | [M] |
| - Peptidase\_S49 | GO:0008233 | peptidase activity | [M] |
|  | GO:0006508 | proteolysis | [B] |
| - PhoH | GO:0005524 | ATP binding | [M] |
| - Phytochelatin | GO:0016756 | glutathione gamma-glutamylcysteinyltransferase activity | [M] |
|  | GO:0046872 | metal ion binding | [M] |
|  | GO:0010038 | response to metal ion | [B] |
|  | GO:0046938 | phytochelatin biosynthetic process | [B] |
| - Proteasom\_PSMB |  |  |  |  |
| - RALF |  |  |  |  |
| - RuvB\_N | GO:0009378 | four-way junction helicase activity | [M] |
|  | GO:0006281 | DNA repair | [B] |
|  | GO:0006310 | DNA recombination | [B] |
| - S6PP |  |  |  |  |
| - Senescence |  |  |  |  |
| - Sina | GO:0006511 | ubiquitin-dependent protein catabolic process | [B] |
|  | GO:0007275 | multicellular organismal development | [B] |
|  | GO:0005634 | nucleus | [C] |
| - SMP |  |  |  |  |
| - Spherulin4 |  |  |  |  |
| - Tex\_N |  |  |  |  |
| - TFIIE-A\_C-term |  |  |  |  |
| - Tom5 |  |  |  |  |
| - TYW3 |  |  |  |  |
| - UPF0139 |  |  |  |  |
| - UPF0564 |  |  |  |  |
| - WWbp |  |  |  |  |
| - Wyosine\_form |  |  |  |  |
| - XG\_FTase | GO:0008107 | galactoside 2-alpha-L-fucosyltransferase activity | [M] |
|  | GO:0042546 | cell wall biogenesis | [B] |
|  | GO:0016020 | membrane | [C] |
| - zf-BED | GO:0003677 | DNA binding | [M] |

---

## Alveolata [eol|tol]

|  |  |  |  |
| --- | --- | --- | --- |
| **Pfam domain(s)** | **GO term acc** | **GO term** | **GO namespace** |
| - 2-Hacid\_dh | GO:0016616 | oxidoreductase activity, acting on the CH-OH group of donors, NAD or NADP as acceptor | [M] |
|  | GO:0051287 | NAD or NADH binding | [M] |
|  | GO:0008152 | metabolic process | [B] |
| - 3-HAO | GO:0000334 | 3-hydroxyanthranilate 3,4-dioxygenase activity | [M] |
|  | GO:0005506 | iron ion binding | [M] |
|  | GO:0008152 | metabolic process | [B] |
|  | GO:0055114 | oxidation reduction | [B] |
| - 3-PAP |  |  |  |  |
| - 7tm\_3 | GO:0004930 | G-protein coupled receptor activity | [M] |
|  | GO:0007186 | G-protein coupled receptor protein signaling pathway | [B] |
|  | GO:0016021 | integral to membrane | [C] |
| - 7TM\_GPCR\_Srsx |  |  |  |  |
| - A\_thal\_3526 |  |  |  |  |
| - AA\_permease | GO:0006810 | transport | [B] |
|  | GO:0055085 | transmembrane transport | [B] |
|  | GO:0016020 | membrane | [C] |
| - AAT | GO:0042318 | penicillin biosynthetic process | [B] |
| - AATase | GO:0004026 | alcohol O-acetyltransferase activity | [M] |
|  | GO:0006066 | alcohol metabolic process | [B] |
| - Abhydrolase\_4 |  |  |  |  |
| - Acetate\_kinase | GO:0016301 | kinase activity | [M] |
|  | GO:0016774 | phosphotransferase activity, carboxyl group as acceptor | [M] |
|  | GO:0008152 | metabolic process | [B] |
|  | GO:0016310 | phosphorylation | [B] |
|  | GO:0005622 | intracellular | [C] |
| - AcetylCoA\_hydro | GO:0003824 | catalytic activity | [M] |
|  | GO:0006084 | acetyl-CoA metabolic process | [B] |
| - Aconitase\_2\_N | GO:0003994 | aconitate hydratase activity | [M] |
|  | GO:0005515 | protein binding | [M] |
|  | GO:0006099 | tricarboxylic acid cycle | [B] |
| - Aconitase\_B\_N |  |  |  |  |
| - Acyl-ACP\_TE |  |  |  |  |
| - Acyl\_CoA\_thio | GO:0016291 | acyl-CoA thioesterase activity | [M] |
|  | GO:0006637 | acyl-CoA metabolic process | [B] |
| - AcylCoA\_DH\_N |  |  |  |  |
| - Ada3 |  |  |  |  |
| - Adenine\_glyco | GO:0008725 | DNA-3-methyladenine glycosylase I activity | [M] |
|  | GO:0006284 | base-excision repair | [B] |
| - ADSL\_C |  |  |  |  |
| - Agenet | GO:0003723 | RNA binding | [M] |
| - AHS1 |  |  |  |  |
| - AICARFT\_IMPCHas | GO:0003824 | catalytic activity | [M] |
|  | GO:0006188 | IMP biosynthetic process | [B] |
| - AIRC | GO:0004638 | phosphoribosylaminoimidazole carboxylase activity | [M] |
|  | GO:0006189 | 'de novo' IMP biosynthetic process | [B] |
| - Ala\_racemase\_C | GO:0008784 | alanine racemase activity | [M] |
|  | GO:0006522 | alanine metabolic process | [B] |
| - Ald\_Xan\_dh\_C | GO:0016491 | oxidoreductase activity | [M] |
|  | GO:0055114 | oxidation reduction | [B] |
| - Ald\_Xan\_dh\_C2 | GO:0016491 | oxidoreductase activity | [M] |
|  | GO:0055114 | oxidation reduction | [B] |
| - Aldolase | GO:0003824 | catalytic activity | [M] |
|  | GO:0008152 | metabolic process | [B] |
| - ALG3 | GO:0016758 | transferase activity, transferring hexosyl groups | [M] |
|  | GO:0005783 | endoplasmic reticulum | [C] |
|  | GO:0016021 | integral to membrane | [C] |
| - Alk\_phosphatase | GO:0016791 | phosphatase activity | [M] |
|  | GO:0008152 | metabolic process | [B] |
| - Alliinase\_C | GO:0016846 | carbon-sulfur lyase activity | [M] |
| - ALMT |  |  |  |  |
| - Alpha\_L\_fucos | GO:0004560 | alpha-L-fucosidase activity | [M] |
|  | GO:0005975 | carbohydrate metabolic process | [B] |
| - ALS\_ss\_C |  |  |  |  |
| - Amidoligase\_2 |  |  |  |  |
| - An\_peroxidase | GO:0004601 | peroxidase activity | [M] |
|  | GO:0020037 | heme binding | [M] |
|  | GO:0006979 | response to oxidative stress | [B] |
|  | GO:0055114 | oxidation reduction | [B] |
| - ANF\_receptor |  |  |  |  |
| - Annexin | GO:0005509 | calcium ion binding | [M] |
|  | GO:0005544 | calcium-dependent phospholipid binding | [M] |
| - Anp1 |  |  |  |  |
| - Anth\_synt\_I\_N | GO:0016833 | oxo-acid-lyase activity | [M] |
|  | GO:0009058 | biosynthetic process | [B] |
| - Apc13p |  |  |  |  |
| - APC2 |  |  |  |  |
| - Aph-1 | GO:0005515 | protein binding | [M] |
|  | GO:0016485 | protein processing | [B] |
|  | GO:0043085 | positive regulation of catalytic activity | [B] |
|  | GO:0016021 | integral to membrane | [C] |
| - aPHC | GO:0016811 | hydrolase activity, acting on carbon-nitrogen (but not peptide) bonds, in linear amides | [M] |
|  | GO:0006672 | ceramide metabolic process | [B] |
|  | GO:0000139 | Golgi membrane | [C] |
|  | GO:0005789 | endoplasmic reticulum membrane | [C] |
|  | GO:0016021 | integral to membrane | [C] |
| - ArabFuran-catal | GO:0046556 | alpha-N-arabinofuranosidase activity | [M] |
|  | GO:0031221 | arabinan metabolic process | [B] |
| - Arb2 |  |  |  |  |
| - Arginosuc\_synth | GO:0004055 | argininosuccinate synthase activity | [M] |
|  | GO:0005524 | ATP binding | [M] |
|  | GO:0006526 | arginine biosynthetic process | [B] |
| - ArgJ | GO:0004358 | glutamate N-acetyltransferase activity | [M] |
|  | GO:0006526 | arginine biosynthetic process | [B] |
| - Armet |  |  |  |  |
| - Arylsulfotrans |  |  |  |  |
| - Asp\_Glu\_race |  |  |  |  |
| - Asparaginase\_2 | GO:0016787 | hydrolase activity | [M] |
| - AstE\_AspA | GO:0016788 | hydrolase activity, acting on ester bonds | [M] |
|  | GO:0008152 | metabolic process | [B] |
| - AT\_hook | GO:0003677 | DNA binding | [M] |
| - ATG11 |  |  |  |  |
| - ATG22 |  |  |  |  |
| - ATP-grasp |  |  |  |  |
| - ATP-grasp\_3 |  |  |  |  |
| - ATP-synt\_10 | GO:0033615 | mitochondrial proton-transporting ATP synthase complex assembly | [B] |
|  | GO:0005743 | mitochondrial inner membrane | [C] |
| - ATP\_Ca\_trans\_C |  |  |  |  |
| - B12-binding | GO:0031419 | cobalamin binding | [M] |
|  | GO:0046872 | metal ion binding | [M] |
| - B12-binding\_2 | GO:0008705 | methionine synthase activity | [M] |
|  | GO:0031419 | cobalamin binding | [M] |
|  | GO:0046872 | metal ion binding | [M] |
|  | GO:0009086 | methionine biosynthetic process | [B] |
| - B12D |  |  |  |  |
| - Bac\_rhamnosid |  |  |  |  |
| - Bac\_rhamnosid\_N |  |  |  |  |
| - Bac\_rhodopsin | GO:0005216 | ion channel activity | [M] |
|  | GO:0006811 | ion transport | [B] |
|  | GO:0016020 | membrane | [C] |
| - Bac\_Ubq\_Cox | GO:0016491 | oxidoreductase activity | [M] |
|  | GO:0055114 | oxidation reduction | [B] |
|  | GO:0016020 | membrane | [C] |
| - BAG | GO:0005515 | protein binding | [M] |
|  | GO:0006915 | apoptosis | [B] |
| - BAR | GO:0005515 | protein binding | [M] |
|  | GO:0005737 | cytoplasm | [C] |
| - BAT2\_N |  |  |  |  |
| - BATS | GO:0004076 | biotin synthase activity | [M] |
|  | GO:0051536 | iron-sulfur cluster binding | [M] |
|  | GO:0009102 | biotin biosynthetic process | [B] |
| - BBE | GO:0016491 | oxidoreductase activity | [M] |
|  | GO:0050660 | FAD binding | [M] |
| - BCCT | GO:0005215 | transporter activity | [M] |
|  | GO:0006810 | transport | [B] |
|  | GO:0016020 | membrane | [C] |
| - Bgal\_small\_N | GO:0004565 | beta-galactosidase activity | [M] |
|  | GO:0005975 | carbohydrate metabolic process | [B] |
|  | GO:0009341 | beta-galactosidase complex | [C] |
| - Borealin |  |  |  |  |
| - BPL\_C | GO:0006464 | protein modification process | [B] |
| - Branch | GO:0008375 | acetylglucosaminyltransferase activity | [M] |
|  | GO:0016020 | membrane | [C] |
| - BRCA-2\_helical | GO:0003697 | single-stranded DNA binding | [M] |
|  | GO:0005515 | protein binding | [M] |
|  | GO:0000724 | double-strand break repair via homologous recombination | [B] |
|  | GO:0006310 | DNA recombination | [B] |
|  | GO:0007090 | regulation of S phase of mitotic cell cycle | [B] |
|  | GO:0005634 | nucleus | [C] |
| - BrkDBD |  |  |  |  |
| - BTAD |  |  |  |  |
| - BUD22 |  |  |  |  |
| - Bvg\_acc\_factor | GO:0016563 | transcription activator activity | [M] |
|  | GO:0045941 | positive regulation of transcription | [B] |
| - C4dic\_mal\_tran | GO:0055085 | transmembrane transport | [B] |
|  | GO:0016021 | integral to membrane | [C] |
| - Cadherin | GO:0005509 | calcium ion binding | [M] |
|  | GO:0007156 | homophilic cell adhesion | [B] |
|  | GO:0016020 | membrane | [C] |
| - Calx-beta | GO:0007154 | cell communication | [B] |
|  | GO:0016021 | integral to membrane | [C] |
| - CaMKII\_AD | GO:0004683 | calmodulin-dependent protein kinase activity | [M] |
|  | GO:0005516 | calmodulin binding | [M] |
|  | GO:0006468 | protein amino acid phosphorylation | [B] |
| - CAP59\_mtransfer |  |  |  |  |
| - Caps\_synth |  |  |  |  |
| - Capsule\_synth | GO:0000271 | polysaccharide biosynthetic process | [B] |
|  | GO:0015774 | polysaccharide transport | [B] |
| - CarD\_TRCF | GO:0003700 | transcription factor activity | [M] |
|  | GO:0006355 | regulation of transcription, DNA-dependent | [B] |
| - Carn\_acyltransf | GO:0008415 | acyltransferase activity | [M] |
| - Cas1p |  |  |  |  |
| - CAT | GO:0008811 | chloramphenicol O-acetyltransferase activity | [M] |
| - Cauli\_VI | GO:0016234 | inclusion body | [C] |
| - CbiX | GO:0016829 | lyase activity | [M] |
|  | GO:0046872 | metal ion binding | [M] |
|  | GO:0009236 | cobalamin biosynthetic process | [B] |
| - CBM\_1 | GO:0004553 | hydrolase activity, hydrolyzing O-glycosyl compounds | [M] |
|  | GO:0030248 | cellulose binding | [M] |
|  | GO:0005975 | carbohydrate metabolic process | [B] |
|  | GO:0005576 | extracellular region | [C] |
| - CBM\_14 | GO:0008061 | chitin binding | [M] |
|  | GO:0006030 | chitin metabolic process | [B] |
|  | GO:0005576 | extracellular region | [C] |
| - CBM\_25 |  |  |  |  |
| - CBM\_4\_9 |  |  |  |  |
| - CBM\_6 | GO:0030246 | carbohydrate binding | [M] |
| - CD225 | GO:0009607 | response to biotic stimulus | [B] |
|  | GO:0016021 | integral to membrane | [C] |
| - CDC27 |  |  |  |  |
| - CDC37\_C |  |  |  |  |
| - CDC37\_M |  |  |  |  |
| - CDC37\_N |  |  |  |  |
| - CdCA1 |  |  |  |  |
| - CDKN3 |  |  |  |  |
| - Cellulose\_synt | GO:0016760 | cellulose synthase (UDP-forming) activity | [M] |
|  | GO:0030244 | cellulose biosynthetic process | [B] |
|  | GO:0016020 | membrane | [C] |
| - CENP-B\_N | GO:0003677 | DNA binding | [M] |
|  | GO:0000775 | chromosome, centromeric region | [C] |
| - CENP-H | GO:0005515 | protein binding | [M] |
|  | GO:0043515 | kinetochore binding | [M] |
|  | GO:0007059 | chromosome segregation | [B] |
|  | GO:0051301 | cell division | [B] |
|  | GO:0000777 | condensed chromosome kinetochore | [C] |
|  | GO:0005634 | nucleus | [C] |
| - Ceramidase\_alk |  |  |  |  |
| - Chal\_sti\_synt\_C | GO:0008415 | acyltransferase activity | [M] |
| - Chal\_sti\_synt\_N | GO:0008415 | acyltransferase activity | [M] |
|  | GO:0009058 | biosynthetic process | [B] |
| - Chalcone | GO:0016872 | intramolecular lyase activity | [M] |
|  | GO:0042398 | cellular amino acid derivative biosynthetic process | [B] |
| - CHASE |  |  |  |  |
| - CheR | GO:0008757 | S-adenosylmethionine-dependent methyltransferase activity | [M] |
| - CHGN | GO:0016758 | transferase activity, transferring hexosyl groups | [M] |
|  | GO:0032580 | Golgi cisterna membrane | [C] |
| - Chitin\_bind\_1 | GO:0008061 | chitin binding | [M] |
| - Chitin\_bind\_3 | GO:0019028 | viral capsid | [C] |
| - Chitin\_synth\_1N |  |  |  |  |
| - Chloroa\_b-bind | GO:0009765 | photosynthesis, light harvesting | [B] |
|  | GO:0016020 | membrane | [C] |
| - CHRD |  |  |  |  |
| - CinA |  |  |  |  |
| - CitMHS | GO:0015137 | citrate transmembrane transporter activity | [M] |
|  | GO:0015746 | citrate transport | [B] |
|  | GO:0055085 | transmembrane transport | [B] |
|  | GO:0016021 | integral to membrane | [C] |
| - Clathrin-link | GO:0005198 | structural molecule activity | [M] |
|  | GO:0005515 | protein binding | [M] |
|  | GO:0006886 | intracellular protein transport | [B] |
|  | GO:0016192 | vesicle-mediated transport | [B] |
|  | GO:0030130 | clathrin coat of trans-Golgi network vesicle | [C] |
|  | GO:0030132 | clathrin coat of coated pit | [C] |
| - CM\_2 | GO:0046417 | chorismate metabolic process | [B] |
| - CmcH\_NodU | GO:0003824 | catalytic activity | [M] |
|  | GO:0009058 | biosynthetic process | [B] |
| - CmcI | GO:0008168 | methyltransferase activity | [M] |
|  | GO:0008610 | lipid biosynthetic process | [B] |
| - CMD |  |  |  |  |
| - Cna\_B |  |  |  |  |
| - CO\_deh\_flav\_C |  |  |  |  |
| - Cob\_adeno\_trans | GO:0005524 | ATP binding | [M] |
|  | GO:0008817 | cob(I)yrinic acid a,c-diamide adenosyltransferase activity | [M] |
|  | GO:0009236 | cobalamin biosynthetic process | [B] |
| - CobA\_CobO\_BtuR | GO:0005524 | ATP binding | [M] |
|  | GO:0008817 | cob(I)yrinic acid a,c-diamide adenosyltransferase activity | [M] |
|  | GO:0009236 | cobalamin biosynthetic process | [B] |
| - CobD\_Cbib | GO:0009236 | cobalamin biosynthetic process | [B] |
|  | GO:0016021 | integral to membrane | [C] |
| - CobN-Mg\_chel | GO:0009058 | biosynthetic process | [B] |
| - COBRA1 | GO:0016481 | negative regulation of transcription | [B] |
|  | GO:0005634 | nucleus | [C] |
| - COG5 |  |  |  |  |
| - COG7 |  |  |  |  |
| - Copper-bind | GO:0005507 | copper ion binding | [M] |
|  | GO:0009055 | electron carrier activity | [M] |
| - COQ7 | GO:0006744 | ubiquinone biosynthetic process | [B] |
|  | GO:0019538 | protein metabolic process | [B] |
| - CorC\_HlyC |  |  |  |  |
| - Cornichon | GO:0007242 | intracellular signaling cascade | [B] |
|  | GO:0016020 | membrane | [C] |
| - CotH |  |  |  |  |
| - COX4 | GO:0004129 | cytochrome-c oxidase activity | [M] |
| - COX6A | GO:0004129 | cytochrome-c oxidase activity | [M] |
|  | GO:0005743 | mitochondrial inner membrane | [C] |
|  | GO:0005751 | mitochondrial respiratory chain complex IV | [C] |
| - CP12 |  |  |  |  |
| - CPDase | GO:0004112 | cyclic-nucleotide phosphodiesterase activity | [M] |
| - CpeT |  |  |  |  |
| - CreA |  |  |  |  |
| - CRS1\_YhbY | GO:0003723 | RNA binding | [M] |
| - CTP\_transf\_3 | GO:0009103 | lipopolysaccharide biosynthetic process | [B] |
| - Cu-oxidase | GO:0016491 | oxidoreductase activity | [M] |
|  | GO:0055114 | oxidation reduction | [B] |
| - Cu-oxidase\_2 | GO:0005507 | copper ion binding | [M] |
|  | GO:0016491 | oxidoreductase activity | [M] |
|  | GO:0055114 | oxidation reduction | [B] |
| - Cu-oxidase\_3 | GO:0005507 | copper ion binding | [M] |
|  | GO:0016491 | oxidoreductase activity | [M] |
|  | GO:0055114 | oxidation reduction | [B] |
| - Cu2\_monoox\_C |  |  |  |  |
| - Cu2\_monooxygen | GO:0004497 | monooxygenase activity | [M] |
|  | GO:0005507 | copper ion binding | [M] |
| - Cu\_amine\_oxidN2 | GO:0005507 | copper ion binding | [M] |
|  | GO:0008131 | amine oxidase activity | [M] |
|  | GO:0048038 | quinone binding | [M] |
|  | GO:0009308 | amine metabolic process | [B] |
|  | GO:0055114 | oxidation reduction | [B] |
| - Cu\_amine\_oxidN3 | GO:0005507 | copper ion binding | [M] |
|  | GO:0008131 | amine oxidase activity | [M] |
|  | GO:0048038 | quinone binding | [M] |
|  | GO:0009308 | amine metabolic process | [B] |
|  | GO:0055114 | oxidation reduction | [B] |
| - Cupin\_1 | GO:0045735 | nutrient reservoir activity | [M] |
| - Cupin\_2 |  |  |  |  |
| - Cupin\_3 |  |  |  |  |
| - Cyanate\_lyase | GO:0008824 | cyanate hydratase activity | [M] |
|  | GO:0009439 | cyanate metabolic process | [B] |
| - CybS | GO:0005506 | iron ion binding | [M] |
|  | GO:0020037 | heme binding | [M] |
|  | GO:0006099 | tricarboxylic acid cycle | [B] |
|  | GO:0005740 | mitochondrial envelope | [C] |
|  | GO:0016021 | integral to membrane | [C] |
| - CytB6-F\_Fe-S | GO:0009496 | plastoquinol-plastocyanin reductase activity | [M] |
|  | GO:0051537 | 2 iron, 2 sulfur cluster binding | [M] |
|  | GO:0055114 | oxidation reduction | [B] |
|  | GO:0042651 | thylakoid membrane | [C] |
| - CYTH | GO:0004016 | adenylate cyclase activity | [M] |
|  | GO:0006171 | cAMP biosynthetic process | [B] |
| - CytochromB561\_N |  |  |  |  |
| - DAHP\_synth\_1 | GO:0009058 | biosynthetic process | [B] |
| - Dala\_Dala\_lig\_C | GO:0008716 | D-alanine-D-alanine ligase activity | [M] |
|  | GO:0009252 | peptidoglycan biosynthetic process | [B] |
| - Dala\_Dala\_lig\_N | GO:0008716 | D-alanine-D-alanine ligase activity | [M] |
|  | GO:0009252 | peptidoglycan biosynthetic process | [B] |
|  | GO:0005618 | cell wall | [C] |
| - DALR\_2 | GO:0000166 | nucleotide binding | [M] |
|  | GO:0004817 | cysteine-tRNA ligase activity | [M] |
|  | GO:0005524 | ATP binding | [M] |
|  | GO:0006412 | translation | [B] |
|  | GO:0006423 | cysteinyl-tRNA aminoacylation | [B] |
|  | GO:0005737 | cytoplasm | [C] |
| - DASH\_Ask1 |  |  |  |  |
| - DASH\_Dad1 |  |  |  |  |
| - DASH\_Dad2 |  |  |  |  |
| - DASH\_Dad4 |  |  |  |  |
| - DASH\_Dam1 |  |  |  |  |
| - DBI\_PRT | GO:0008939 | nicotinate-nucleotide-dimethylbenzimidazole phosphoribosyltransferase activity | [M] |
|  | GO:0009236 | cobalamin biosynthetic process | [B] |
| - DDA1 |  |  |  |  |
| - Ded\_cyto | GO:0005085 | guanyl-nucleotide exchange factor activity | [M] |
|  | GO:0005525 | GTP binding | [M] |
|  | GO:0051020 | GTPase binding | [M] |
| - Dehydrin | GO:0006950 | response to stress | [B] |
|  | GO:0009415 | response to water | [B] |
| - Desulfoferrodox | GO:0005506 | iron ion binding | [M] |
|  | GO:0016491 | oxidoreductase activity | [M] |
|  | GO:0055114 | oxidation reduction | [B] |
| - Dev\_Cell\_Death |  |  |  |  |
| - DGCR6 |  |  |  |  |
| - DHBP\_synthase | GO:0008686 | 3,4-dihydroxy-2-butanone-4-phosphate synthase activity | [M] |
|  | GO:0009231 | riboflavin biosynthetic process | [B] |
| - DHHA1 | GO:0003676 | nucleic acid binding | [M] |
| - DHQS |  |  |  |  |
| - Dioxygenase\_C | GO:0003824 | catalytic activity | [M] |
|  | GO:0008199 | ferric iron binding | [M] |
|  | GO:0006725 | cellular aromatic compound metabolic process | [B] |
|  | GO:0055114 | oxidation reduction | [B] |
| - Disintegrin |  |  |  |  |
| - DIT1\_PvcA |  |  |  |  |
| - DMAP1 | GO:0016481 | negative regulation of transcription | [B] |
|  | GO:0005634 | nucleus | [C] |
| - DMRL\_synthase | GO:0009231 | riboflavin biosynthetic process | [B] |
|  | GO:0009349 | riboflavin synthase complex | [C] |
| - DNA\_binding\_1 | GO:0003824 | catalytic activity | [M] |
|  | GO:0006281 | DNA repair | [B] |
| - DnaB\_C | GO:0003678 | DNA helicase activity | [M] |
|  | GO:0005524 | ATP binding | [M] |
|  | GO:0006260 | DNA replication | [B] |
| - DPBB\_1 |  |  |  |  |
| - Dpoe2NT |  |  |  |  |
| - Drc1-Sld2 |  |  |  |  |
| - DREV |  |  |  |  |
| - Drf\_FH3 | GO:0003779 | actin binding | [M] |
|  | GO:0016043 | cellular component organization | [B] |
| - Drmip\_Hesp |  |  |  |  |
| - DRTGG |  |  |  |  |
| - DSBA | GO:0015035 | protein disulfide oxidoreductase activity | [M] |
|  | GO:0030288 | outer membrane-bounded periplasmic space | [C] |
| - DsbD | GO:0017004 | cytochrome complex assembly | [B] |
|  | GO:0055114 | oxidation reduction | [B] |
|  | GO:0016020 | membrane | [C] |
| - DTHCT | GO:0003677 | DNA binding | [M] |
|  | GO:0003918 | DNA topoisomerase (ATP-hydrolyzing) activity | [M] |
|  | GO:0005524 | ATP binding | [M] |
|  | GO:0005634 | nucleus | [C] |
| - DUF1012 |  |  |  |  |
| - DUF1022 |  |  |  |  |
| - DUF108 | GO:0016491 | oxidoreductase activity | [M] |
|  | GO:0006742 | NADP catabolic process | [B] |
|  | GO:0019363 | pyridine nucleotide biosynthetic process | [B] |
|  | GO:0055114 | oxidation reduction | [B] |
| - DUF1092 |  |  |  |  |
| - DUF111 |  |  |  |  |
| - DUF1118 |  |  |  |  |
| - DUF1208 |  |  |  |  |
| - DUF1212 |  |  |  |  |
| - DUF1230 |  |  |  |  |
| - DUF1234 |  |  |  |  |
| - DUF1244 |  |  |  |  |
| - DUF1255 |  |  |  |  |
| - DUF126 |  |  |  |  |
| - DUF1275 |  |  |  |  |
| - DUF1289 |  |  |  |  |
| - DUF1301 |  |  |  |  |
| - DUF1325 |  |  |  |  |
| - DUF1330 |  |  |  |  |
| - DUF1336 |  |  |  |  |
| - DUF1350 |  |  |  |  |
| - DUF1365 |  |  |  |  |
| - DUF1394 |  |  |  |  |
| - DUF1399 |  |  |  |  |
| - DUF140 |  |  |  |  |
| - DUF1409 |  |  |  |  |
| - DUF1415 |  |  |  |  |
| - DUF1421 |  |  |  |  |
| - DUF1445 |  |  |  |  |
| - DUF1446 |  |  |  |  |
| - DUF1479 |  |  |  |  |
| - DUF149 |  |  |  |  |
| - DUF1499 |  |  |  |  |
| - DUF1501 |  |  |  |  |
| - DUF1524 |  |  |  |  |
| - DUF1537 |  |  |  |  |
| - DUF1640 |  |  |  |  |
| - DUF1688 |  |  |  |  |
| - DUF1697 |  |  |  |  |
| - DUF1713 |  |  |  |  |
| - DUF1729 |  |  |  |  |
| - DUF1736 |  |  |  |  |
| - DUF1742 |  |  |  |  |
| - DUF1751 |  |  |  |  |
| - DUF1752 |  |  |  |  |
| - DUF1762 |  |  |  |  |
| - DUF1771 |  |  |  |  |
| - DUF1783 |  |  |  |  |
| - DUF1785 |  |  |  |  |
| - DUF1800 |  |  |  |  |
| - DUF1823 |  |  |  |  |
| - DUF1824 |  |  |  |  |
| - DUF1825 |  |  |  |  |
| - DUF1826 |  |  |  |  |
| - DUF1838 |  |  |  |  |
| - DUF1853 |  |  |  |  |
| - DUF1855 |  |  |  |  |
| - DUF1903 |  |  |  |  |
| - DUF1929 |  |  |  |  |
| - DUF1949 |  |  |  |  |
| - DUF1989 |  |  |  |  |
| - DUF1990 |  |  |  |  |
| - DUF1992 |  |  |  |  |
| - DUF1995 |  |  |  |  |
| - DUF1996 |  |  |  |  |
| - DUF1997 |  |  |  |  |
| - DUF1998 |  |  |  |  |
| - DUF2003 |  |  |  |  |
| - DUF2008 |  |  |  |  |
| - DUF2034 |  |  |  |  |
| - DUF2046 |  |  |  |  |
| - DUF2048 |  |  |  |  |
| - DUF2054 |  |  |  |  |
| - DUF2061 |  |  |  |  |
| - DUF2062 |  |  |  |  |
| - DUF2064 |  |  |  |  |
| - DUF208 |  |  |  |  |
| - DUF2086 |  |  |  |  |
| - DUF2087 |  |  |  |  |
| - DUF2088 |  |  |  |  |
| - DUF2181 |  |  |  |  |
| - DUF2183 |  |  |  |  |
| - DUF2196 |  |  |  |  |
| - DUF2214 |  |  |  |  |
| - DUF2215 |  |  |  |  |
| - DUF2233 |  |  |  |  |
| - DUF2246 |  |  |  |  |
| - DUF2252 |  |  |  |  |
| - DUF2256 |  |  |  |  |
| - DUF2263 |  |  |  |  |
| - DUF227 |  |  |  |  |
| - DUF231 |  |  |  |  |
| - DUF2346 |  |  |  |  |
| - DUF2347 |  |  |  |  |
| - DUF2355 |  |  |  |  |
| - DUF2358 |  |  |  |  |
| - DUF2372 |  |  |  |  |
| - DUF2373 |  |  |  |  |
| - DUF2401 |  |  |  |  |
| - DUF2403 |  |  |  |  |
| - DUF2407 |  |  |  |  |
| - DUF2411 |  |  |  |  |
| - DUF2435 |  |  |  |  |
| - DUF2464 |  |  |  |  |
| - DUF2470 |  |  |  |  |
| - DUF2499 |  |  |  |  |
| - DUF262 |  |  |  |  |
| - DUF265 | GO:0005524 | ATP binding | [M] |
|  | GO:0016740 | transferase activity | [M] |
| - DUF268 |  |  |  |  |
| - DUF2723 |  |  |  |  |
| - DUF273 |  |  |  |  |
| - DUF2779 |  |  |  |  |
| - DUF2780 |  |  |  |  |
| - DUF2781 |  |  |  |  |
| - DUF2786 |  |  |  |  |
| - DUF28 |  |  |  |  |
| - DUF2807 |  |  |  |  |
| - DUF2817 |  |  |  |  |
| - DUF2828 |  |  |  |  |
| - DUF2838 |  |  |  |  |
| - DUF285 |  |  |  |  |
| - DUF2854 |  |  |  |  |
| - DUF288 |  |  |  |  |
| - DUF2930 |  |  |  |  |
| - DUF2961 |  |  |  |  |
| - DUF2985 |  |  |  |  |
| - DUF299 | GO:0005524 | ATP binding | [M] |
|  | GO:0016772 | transferase activity, transferring phosphorus-containing groups | [M] |
| - DUF2993 |  |  |  |  |
| - DUF2997 |  |  |  |  |
| - DUF3007 |  |  |  |  |
| - DUF303 |  |  |  |  |
| - DUF305 |  |  |  |  |
| - DUF3054 |  |  |  |  |
| - DUF3067 |  |  |  |  |
| - DUF3082 |  |  |  |  |
| - DUF3089 |  |  |  |  |
| - DUF3110 |  |  |  |  |
| - DUF3118 |  |  |  |  |
| - DUF3119 |  |  |  |  |
| - DUF3128 |  |  |  |  |
| - DUF3172 |  |  |  |  |
| - DUF3184 |  |  |  |  |
| - DUF3237 |  |  |  |  |
| - DUF328 |  |  |  |  |
| - DUF3294 |  |  |  |  |
| - DUF3317 |  |  |  |  |
| - DUF3321 |  |  |  |  |
| - DUF3337 |  |  |  |  |
| - DUF3342 |  |  |  |  |
| - DUF3353 |  |  |  |  |
| - DUF3354 |  |  |  |  |
| - DUF336 |  |  |  |  |
| - DUF3384 |  |  |  |  |
| - DUF3411 |  |  |  |  |
| - DUF3414 |  |  |  |  |
| - DUF3419 |  |  |  |  |
| - DUF3427 |  |  |  |  |
| - DUF3453 |  |  |  |  |
| - DUF3456 |  |  |  |  |
| - DUF3464 |  |  |  |  |
| - DUF347 |  |  |  |  |
| - DUF3479 |  |  |  |  |
| - DUF3490 |  |  |  |  |
| - DUF3493 |  |  |  |  |
| - DUF3494 |  |  |  |  |
| - DUF3506 |  |  |  |  |
| - DUF3529 |  |  |  |  |
| - DUF3535 |  |  |  |  |
| - DUF3543 |  |  |  |  |
| - DUF3554 |  |  |  |  |
| - DUF3556 |  |  |  |  |
| - DUF3593 |  |  |  |  |
| - DUF3598 |  |  |  |  |
| - DUF3605 |  |  |  |  |
| - DUF3611 |  |  |  |  |
| - DUF3641 |  |  |  |  |
| - DUF3656 |  |  |  |  |
| - DUF3677 |  |  |  |  |
| - DUF3684 |  |  |  |  |
| - DUF37 |  |  |  |  |
| - DUF3727 |  |  |  |  |
| - DUF3730 |  |  |  |  |
| - DUF3754 |  |  |  |  |
| - DUF3767 |  |  |  |  |
| - DUF377 |  |  |  |  |
| - DUF389 |  |  |  |  |
| - DUF399 |  |  |  |  |
| - DUF482 |  |  |  |  |
| - DUF493 |  |  |  |  |
| - DUF500 |  |  |  |  |
| - DUF519 |  |  |  |  |
| - DUF521 |  |  |  |  |
| - DUF540 | GO:0019344 | cysteine biosynthetic process | [B] |
|  | GO:0009276 | Gram-negative-bacterium-type cell wall | [C] |
|  | GO:0016021 | integral to membrane | [C] |
| - DUF548 |  |  |  |  |
| - DUF55 |  |  |  |  |
| - DUF563 | GO:0016757 | transferase activity, transferring glycosyl groups | [M] |
| - DUF567 |  |  |  |  |
| - DUF583 |  |  |  |  |
| - DUF588 |  |  |  |  |
| - DUF599 |  |  |  |  |
| - DUF606 |  |  |  |  |
| - DUF608 | GO:0004348 | glucosylceramidase activity | [M] |
|  | GO:0006665 | sphingolipid metabolic process | [B] |
|  | GO:0016021 | integral to membrane | [C] |
| - DUF616 |  |  |  |  |
| - DUF633 |  |  |  |  |
| - DUF647 |  |  |  |  |
| - DUF659 |  |  |  |  |
| - DUF662 |  |  |  |  |
| - DUF672 |  |  |  |  |
| - DUF706 | GO:0005506 | iron ion binding | [M] |
|  | GO:0050113 | inositol oxygenase activity | [M] |
|  | GO:0019310 | inositol catabolic process | [B] |
|  | GO:0055114 | oxidation reduction | [B] |
|  | GO:0005737 | cytoplasm | [C] |
| - DUF711 |  |  |  |  |
| - DUF718 | GO:0016857 | racemase and epimerase activity, acting on carbohydrates and derivatives | [M] |
|  | GO:0019299 | rhamnose metabolic process | [B] |
|  | GO:0005737 | cytoplasm | [C] |
| - DUF72 |  |  |  |  |
| - DUF781 |  |  |  |  |
| - DUF820 |  |  |  |  |
| - DUF829 |  |  |  |  |
| - DUF839 |  |  |  |  |
| - DUF849 |  |  |  |  |
| - DUF88 |  |  |  |  |
| - DUF885 |  |  |  |  |
| - DUF889 |  |  |  |  |
| - DUF901 |  |  |  |  |
| - DUF92 |  |  |  |  |
| - DUF938 |  |  |  |  |
| - DUF946 |  |  |  |  |
| - DUF952 |  |  |  |  |
| - Dynactin\_p22 |  |  |  |  |
| - EAF |  |  |  |  |
| - EB\_dh | GO:0020037 | heme binding | [M] |
|  | GO:0055114 | oxidation reduction | [B] |
| - Ectoine\_synth | GO:0016836 | hydro-lyase activity | [M] |
|  | GO:0006596 | polyamine biosynthetic process | [B] |
| - eIF-4B |  |  |  |  |
| - eIF3\_N |  |  |  |  |
| - ELH | GO:0005179 | hormone activity | [M] |
|  | GO:0007275 | multicellular organismal development | [B] |
|  | GO:0005576 | extracellular region | [C] |
| - ELL | GO:0016944 | RNA polymerase II transcription elongation factor activity | [M] |
|  | GO:0006368 | RNA elongation from RNA polymerase II promoter | [B] |
|  | GO:0008023 | transcription elongation factor complex | [C] |
| - Endonuclease\_1 | GO:0004518 | nuclease activity | [M] |
| - Endosulfine |  |  |  |  |
| - ERp29 | GO:0005783 | endoplasmic reticulum | [C] |
| - Erv26 |  |  |  |  |
| - Erythro\_esteras | GO:0046677 | response to antibiotic | [B] |
| - EST1 |  |  |  |  |
| - Exo70 | GO:0006887 | exocytosis | [B] |
|  | GO:0000145 | exocyst | [C] |
| - F\_bP\_aldolase | GO:0004332 | fructose-bisphosphate aldolase activity | [M] |
|  | GO:0008270 | zinc ion binding | [M] |
|  | GO:0006096 | glycolysis | [B] |
| - FA\_desaturase\_2 | GO:0045300 | acyl-[acyl-carrier-protein] desaturase activity | [M] |
|  | GO:0006631 | fatty acid metabolic process | [B] |
|  | GO:0055114 | oxidation reduction | [B] |
| - FA\_FANCE |  |  |  |  |
| - FAD\_binding\_5 | GO:0016491 | oxidoreductase activity | [M] |
|  | GO:0055114 | oxidation reduction | [B] |
| - FAE1\_CUT1\_RppA | GO:0016747 | transferase activity, transferring acyl groups other than amino-acyl groups | [M] |
|  | GO:0006633 | fatty acid biosynthetic process | [B] |
|  | GO:0016020 | membrane | [C] |
| - FAE\_3-kCoA\_syn1 |  |  |  |  |
| - Fapy\_DNA\_glyco | GO:0003684 | damaged DNA binding | [M] |
|  | GO:0003906 | DNA-(apurinic or apyrimidinic site) lyase activity | [M] |
|  | GO:0008270 | zinc ion binding | [M] |
|  | GO:0016799 | hydrolase activity, hydrolyzing N-glycosyl compounds | [M] |
|  | GO:0006284 | base-excision repair | [B] |
| - FAR1 |  |  |  |  |
| - Fascin | GO:0030674 | protein binding, bridging | [M] |
|  | GO:0051015 | actin filament binding | [M] |
| - FBA | GO:0005515 | protein binding | [M] |
|  | GO:0030163 | protein catabolic process | [B] |
| - Fe\_bilin\_red | GO:0016636 | oxidoreductase activity, acting on the CH-CH group of donors, iron-sulfur protein as acceptor | [M] |
|  | GO:0050897 | cobalt ion binding | [M] |
|  | GO:0010024 | phytochromobilin biosynthetic process | [B] |
|  | GO:0055114 | oxidation reduction | [B] |
| - Fe\_hyd\_SSU |  |  |  |  |
| - Fea1 |  |  |  |  |
| - Fer2\_2 | GO:0016491 | oxidoreductase activity | [M] |
|  | GO:0046872 | metal ion binding | [M] |
|  | GO:0055114 | oxidation reduction | [B] |
| - Fer2\_BFD |  |  |  |  |
| - Ferric\_reduct | GO:0005506 | iron ion binding | [M] |
|  | GO:0009055 | electron carrier activity | [M] |
|  | GO:0016491 | oxidoreductase activity | [M] |
|  | GO:0050660 | FAD binding | [M] |
|  | GO:0016021 | integral to membrane | [C] |
| - FeThRed\_B | GO:0008937 | ferredoxin reductase activity | [M] |
|  | GO:0055114 | oxidation reduction | [B] |
| - FG-GAP |  |  |  |  |
| - FGE-sulfatase |  |  |  |  |
| - Fibrinogen\_C | GO:0005102 | receptor binding | [M] |
|  | GO:0007165 | signal transduction | [B] |
| - Fic |  |  |  |  |
| - Filament |  |  |  |  |
| - FIST |  |  |  |  |
| - FIST\_C |  |  |  |  |
| - Flavin\_Reduct |  |  |  |  |
| - Flavodoxin\_2 | GO:0009055 | electron carrier activity | [M] |
|  | GO:0016491 | oxidoreductase activity | [M] |
|  | GO:0050662 | coenzyme binding | [M] |
| - FLYWCH |  |  |  |  |
| - FmdA\_AmdA | GO:0016811 | hydrolase activity, acting on carbon-nitrogen (but not peptide) bonds, in linear amides | [M] |
|  | GO:0008152 | metabolic process | [B] |
| - FMN\_red |  |  |  |  |
| - fn2 |  |  |  |  |
| - Foie-gras\_1 |  |  |  |  |
| - Folate\_carrier | GO:0005542 | folic acid binding | [M] |
|  | GO:0008518 | reduced folate carrier activity | [M] |
|  | GO:0006810 | transport | [B] |
|  | GO:0016020 | membrane | [C] |
| - Fork\_head\_N |  |  |  |  |
| - FPN1 | GO:0005381 | iron ion transmembrane transporter activity | [M] |
|  | GO:0006826 | iron ion transport | [B] |
|  | GO:0016021 | integral to membrane | [C] |
| - FR47 |  |  |  |  |
| - FragX\_IP |  |  |  |  |
| - FrhB\_FdhB\_C |  |  |  |  |
| - FrhB\_FdhB\_N |  |  |  |  |
| - Frizzled | GO:0004926 | non-G-protein coupled 7TM receptor activity | [M] |
|  | GO:0007166 | cell surface receptor linked signal transduction | [B] |
|  | GO:0016020 | membrane | [C] |
| - Fructosamin\_kin |  |  |  |  |
| - FTCD | GO:0005542 | folic acid binding | [M] |
|  | GO:0016740 | transferase activity | [M] |
|  | GO:0008152 | metabolic process | [B] |
| - FTCD\_C | GO:0003824 | catalytic activity | [M] |
|  | GO:0044237 | cellular metabolic process | [B] |
| - FTCD\_N | GO:0005542 | folic acid binding | [M] |
|  | GO:0016740 | transferase activity | [M] |
|  | GO:0008152 | metabolic process | [B] |
| - FTHFS | GO:0004329 | formate-tetrahydrofolate ligase activity | [M] |
|  | GO:0005524 | ATP binding | [M] |
|  | GO:0009396 | folic acid and derivative biosynthetic process | [B] |
| - FTR1 | GO:0055085 | transmembrane transport | [B] |
|  | GO:0016020 | membrane | [C] |
| - FtsZ\_C |  |  |  |  |
| - Fucokinase |  |  |  |  |
| - FUSC |  |  |  |  |
| - G-alpha | GO:0004871 | signal transducer activity | [M] |
|  | GO:0019001 | guanyl nucleotide binding | [M] |
|  | GO:0007186 | G-protein coupled receptor protein signaling pathway | [B] |
| - G\_glu\_transpept | GO:0003840 | gamma-glutamyltransferase activity | [M] |
| - Gal-3-0\_sulfotr | GO:0001733 | galactosylceramide sulfotransferase activity | [M] |
|  | GO:0009058 | biosynthetic process | [B] |
|  | GO:0005794 | Golgi apparatus | [C] |
|  | GO:0016021 | integral to membrane | [C] |
| - Gal-bind\_lectin | GO:0005529 | sugar binding | [M] |
| - Galactosyl\_T | GO:0008378 | galactosyltransferase activity | [M] |
|  | GO:0006486 | protein amino acid glycosylation | [B] |
|  | GO:0016020 | membrane | [C] |
| - Galactosyl\_T\_2 | GO:0016757 | transferase activity, transferring glycosyl groups | [M] |
|  | GO:0005975 | carbohydrate metabolic process | [B] |
| - GalP\_UDP\_transf | GO:0008108 | UDP-glucose:hexose-1-phosphate uridylyltransferase activity | [M] |
|  | GO:0006012 | galactose metabolic process | [B] |
| - GARS\_A |  |  |  |  |
| - GARS\_C | GO:0004637 | phosphoribosylamine-glycine ligase activity | [M] |
|  | GO:0009113 | purine base biosynthetic process | [B] |
| - GARS\_N | GO:0004637 | phosphoribosylamine-glycine ligase activity | [M] |
|  | GO:0009113 | purine base biosynthetic process | [B] |
| - GATase\_3 | GO:0003824 | catalytic activity | [M] |
|  | GO:0009236 | cobalamin biosynthetic process | [B] |
| - GBA2\_N |  |  |  |  |
| - GCK |  |  |  |  |
| - GCR1\_C |  |  |  |  |
| - GD\_AH\_C | GO:0016836 | hydro-lyase activity | [M] |
| - GerE | GO:0003700 | transcription factor activity | [M] |
|  | GO:0043565 | sequence-specific DNA binding | [M] |
|  | GO:0006355 | regulation of transcription, DNA-dependent | [B] |
|  | GO:0005622 | intracellular | [C] |
| - GFO\_IDH\_MocA\_C | GO:0016491 | oxidoreductase activity | [M] |
|  | GO:0008152 | metabolic process | [B] |
|  | GO:0055114 | oxidation reduction | [B] |
| - GH3 |  |  |  |  |
| - GidB | GO:0008649 | rRNA methyltransferase activity | [M] |
|  | GO:0006364 | rRNA processing | [B] |
|  | GO:0005737 | cytoplasm | [C] |
| - GlcNAc\_2-epim | GO:0004476 | mannose-6-phosphate isomerase activity | [M] |
|  | GO:0006013 | mannose metabolic process | [B] |
| - GLF | GO:0008767 | UDP-galactopyranose mutase activity | [M] |
| - Globin | GO:0005506 | iron ion binding | [M] |
|  | GO:0020037 | heme binding | [M] |
| - Glu-tRNAGln | GO:0006450 | regulation of translational fidelity | [B] |
| - Glutaminase | GO:0004359 | glutaminase activity | [M] |
|  | GO:0006541 | glutamine metabolic process | [B] |
| - Glutaredoxin2\_C |  |  |  |  |
| - GlutR\_dimer | GO:0008883 | glutamyl-tRNA reductase activity | [M] |
|  | GO:0050661 | NADP or NADPH binding | [M] |
|  | GO:0033014 | tetrapyrrole biosynthetic process | [B] |
|  | GO:0055114 | oxidation reduction | [B] |
| - GlutR\_N | GO:0008883 | glutamyl-tRNA reductase activity | [M] |
|  | GO:0050661 | NADP or NADPH binding | [M] |
|  | GO:0033014 | tetrapyrrole biosynthetic process | [B] |
|  | GO:0055114 | oxidation reduction | [B] |
| - Gly\_radical | GO:0003824 | catalytic activity | [M] |
|  | GO:0008152 | metabolic process | [B] |
| - Glyco\_hydro\_10 | GO:0004553 | hydrolase activity, hydrolyzing O-glycosyl compounds | [M] |
|  | GO:0005975 | carbohydrate metabolic process | [B] |
| - Glyco\_hydro\_12 | GO:0008810 | cellulase activity | [M] |
|  | GO:0000272 | polysaccharide catabolic process | [B] |
| - Glyco\_hydro\_28 | GO:0004650 | polygalacturonase activity | [M] |
|  | GO:0005975 | carbohydrate metabolic process | [B] |
| - Glyco\_hydro\_2\_C | GO:0004553 | hydrolase activity, hydrolyzing O-glycosyl compounds | [M] |
|  | GO:0005975 | carbohydrate metabolic process | [B] |
| - Glyco\_hydro\_30 | GO:0004348 | glucosylceramidase activity | [M] |
|  | GO:0006665 | sphingolipid metabolic process | [B] |
|  | GO:0007040 | lysosome organization | [B] |
|  | GO:0005764 | lysosome | [C] |
| - Glyco\_hydro\_32C |  |  |  |  |
| - Glyco\_hydro\_32N |  |  |  |  |
| - Glyco\_hydro\_39 | GO:0004553 | hydrolase activity, hydrolyzing O-glycosyl compounds | [M] |
|  | GO:0005975 | carbohydrate metabolic process | [B] |
| - Glyco\_hydro\_43 | GO:0004553 | hydrolase activity, hydrolyzing O-glycosyl compounds | [M] |
|  | GO:0005975 | carbohydrate metabolic process | [B] |
| - Glyco\_hydro\_47 | GO:0004571 | mannosyl-oligosaccharide 1,2-alpha-mannosidase activity | [M] |
|  | GO:0005509 | calcium ion binding | [M] |
|  | GO:0016020 | membrane | [C] |
| - Glyco\_hydro\_53 | GO:0015926 | glucosidase activity | [M] |
| - Glyco\_hydro\_6 | GO:0004553 | hydrolase activity, hydrolyzing O-glycosyl compounds | [M] |
|  | GO:0030245 | cellulose catabolic process | [B] |
| - Glyco\_hydro\_67C | GO:0046559 | alpha-glucuronidase activity | [M] |
|  | GO:0045493 | xylan catabolic process | [B] |
|  | GO:0005576 | extracellular region | [C] |
| - Glyco\_hydro\_67M | GO:0046559 | alpha-glucuronidase activity | [M] |
|  | GO:0045493 | xylan catabolic process | [B] |
|  | GO:0005576 | extracellular region | [C] |
| - Glyco\_hydro\_7 | GO:0004553 | hydrolase activity, hydrolyzing O-glycosyl compounds | [M] |
|  | GO:0005975 | carbohydrate metabolic process | [B] |
| - Glyco\_hydro\_72 |  |  |  |  |
| - Glyco\_hydro\_79n | GO:0016798 | hydrolase activity, acting on glycosyl bonds | [M] |
|  | GO:0016020 | membrane | [C] |
| - Glyco\_hydro\_88 |  |  |  |  |
| - Glyco\_hydro\_9 | GO:0004553 | hydrolase activity, hydrolyzing O-glycosyl compounds | [M] |
|  | GO:0005975 | carbohydrate metabolic process | [B] |
| - Glyco\_hydro\_92 |  |  |  |  |
| - Glyco\_hydro\_cc |  |  |  |  |
| - Glyco\_transf\_10 | GO:0008417 | fucosyltransferase activity | [M] |
|  | GO:0006486 | protein amino acid glycosylation | [B] |
|  | GO:0016020 | membrane | [C] |
| - Glyco\_transf\_15 | GO:0004377 | glycolipid 2-alpha-mannosyltransferase activity | [M] |
|  | GO:0006486 | protein amino acid glycosylation | [B] |
|  | GO:0016020 | membrane | [C] |
| - Glyco\_transf\_25 | GO:0009103 | lipopolysaccharide biosynthetic process | [B] |
| - Glyco\_transf\_28 | GO:0016758 | transferase activity, transferring hexosyl groups | [M] |
|  | GO:0005975 | carbohydrate metabolic process | [B] |
|  | GO:0030259 | lipid glycosylation | [B] |
| - Glyco\_transf\_34 | GO:0016758 | transferase activity, transferring hexosyl groups | [M] |
|  | GO:0016021 | integral to membrane | [C] |
| - Glyco\_transf\_64 | GO:0016758 | transferase activity, transferring hexosyl groups | [M] |
|  | GO:0031227 | intrinsic to endoplasmic reticulum membrane | [C] |
| - Glycophorin\_A | GO:0016021 | integral to membrane | [C] |
| - Glycos\_transf\_N | GO:0005529 | sugar binding | [M] |
|  | GO:0016740 | transferase activity | [M] |
|  | GO:0005975 | carbohydrate metabolic process | [B] |
| - GNT-I | GO:0003827 | alpha-1,3-mannosylglycoprotein 2-beta-N-acetylglucosaminyltransferase activity | [M] |
|  | GO:0006487 | protein amino acid N-linked glycosylation | [B] |
|  | GO:0000139 | Golgi membrane | [C] |
| - GRIM-19 |  |  |  |  |
| - GRP |  |  |  |  |
| - Grp1\_Fun34\_YaaH | GO:0016020 | membrane | [C] |
| - GSDH | GO:0016901 | oxidoreductase activity, acting on the CH-OH group of donors, quinone or similar compound as acceptor | [M] |
|  | GO:0048038 | quinone binding | [M] |
|  | GO:0005975 | carbohydrate metabolic process | [B] |
| - GSIII\_N |  |  |  |  |
| - GTP\_CH\_N |  |  |  |  |
| - GTP\_cyclohydro2 | GO:0003935 | GTP cyclohydrolase II activity | [M] |
|  | GO:0009231 | riboflavin biosynthetic process | [B] |
| - GtrA | GO:0000271 | polysaccharide biosynthetic process | [B] |
|  | GO:0006810 | transport | [B] |
|  | GO:0016021 | integral to membrane | [C] |
| - Guanylate\_cyc\_2 |  |  |  |  |
| - GUN4 |  |  |  |  |
| - H2TH | GO:0003684 | damaged DNA binding | [M] |
|  | GO:0003906 | DNA-(apurinic or apyrimidinic site) lyase activity | [M] |
|  | GO:0008270 | zinc ion binding | [M] |
|  | GO:0016799 | hydrolase activity, hydrolyzing N-glycosyl compounds | [M] |
|  | GO:0006289 | nucleotide-excision repair | [B] |
| - HA |  |  |  |  |
| - HAND | GO:0031491 | nucleosome binding | [M] |
|  | GO:0043044 | ATP-dependent chromatin remodeling | [B] |
|  | GO:0016585 | chromatin remodeling complex | [C] |
| - HCO3\_cotransp | GO:0015380 | anion exchanger activity | [M] |
|  | GO:0006820 | anion transport | [B] |
|  | GO:0016021 | integral to membrane | [C] |
| - HCR | GO:0030154 | cell differentiation | [B] |
|  | GO:0005634 | nucleus | [C] |
|  | GO:0005737 | cytoplasm | [C] |
| - HEAT\_PBS |  |  |  |  |
| - Hep\_Hag | GO:0009405 | pathogenesis | [B] |
|  | GO:0019867 | outer membrane | [C] |
| - HIG\_1\_N |  |  |  |  |
| - His\_biosynth | GO:0000105 | histidine biosynthetic process | [B] |
| - HisG | GO:0003879 | ATP phosphoribosyltransferase activity | [M] |
|  | GO:0000105 | histidine biosynthetic process | [B] |
|  | GO:0005737 | cytoplasm | [C] |
| - HisG\_C | GO:0000287 | magnesium ion binding | [M] |
|  | GO:0003879 | ATP phosphoribosyltransferase activity | [M] |
|  | GO:0000105 | histidine biosynthetic process | [B] |
|  | GO:0005737 | cytoplasm | [C] |
| - Histidinol\_dh | GO:0004399 | histidinol dehydrogenase activity | [M] |
|  | GO:0008270 | zinc ion binding | [M] |
|  | GO:0051287 | NAD or NADH binding | [M] |
|  | GO:0000105 | histidine biosynthetic process | [B] |
| - HLH | GO:0030528 | transcription regulator activity | [M] |
|  | GO:0045449 | regulation of transcription | [B] |
| - HNOB | GO:0004383 | guanylate cyclase activity | [M] |
|  | GO:0020037 | heme binding | [M] |
|  | GO:0006182 | cGMP biosynthetic process | [B] |
| - HNOBA | GO:0004383 | guanylate cyclase activity | [M] |
|  | GO:0006182 | cGMP biosynthetic process | [B] |
| - Homoserine\_dh | GO:0006520 | cellular amino acid metabolic process | [B] |
| - HopJ |  |  |  |  |
| - HpaB | GO:0016712 | oxidoreductase activity, acting on paired donors, with incorporation or reduction of molecular oxygen, reduced flavin or flavoprotein as one donor, and incorporation of one atom of oxygen | [M] |
|  | GO:0010124 | phenylacetate catabolic process | [B] |
| - HpaB\_N |  |  |  |  |
| - HpcH\_HpaI | GO:0016830 | carbon-carbon lyase activity | [M] |
|  | GO:0006725 | cellular aromatic compound metabolic process | [B] |
| - Hpt | GO:0004871 | signal transducer activity | [M] |
|  | GO:0000160 | two-component signal transduction system (phosphorelay) | [B] |
| - HrpB\_C |  |  |  |  |
| - HtrL\_YibB |  |  |  |  |
| - HYR |  |  |  |  |
| - I-set |  |  |  |  |
| - IATP | GO:0004857 | enzyme inhibitor activity | [M] |
|  | GO:0045980 | negative regulation of nucleotide metabolic process | [B] |
|  | GO:0005739 | mitochondrion | [C] |
| - IDH | GO:0004450 | isocitrate dehydrogenase (NADP+) activity | [M] |
|  | GO:0006099 | tricarboxylic acid cycle | [B] |
|  | GO:0055114 | oxidation reduction | [B] |
| - IF2\_N | GO:0003743 | translation initiation factor activity | [M] |
|  | GO:0006413 | translational initiation | [B] |
| - IF\_tail |  |  |  |  |
| - Ifi-6-16 |  |  |  |  |
| - IFRD |  |  |  |  |
| - IGPD | GO:0004424 | imidazoleglycerol-phosphate dehydratase activity | [M] |
|  | GO:0000105 | histidine biosynthetic process | [B] |
| - IlvC | GO:0004455 | ketol-acid reductoisomerase activity | [M] |
|  | GO:0009082 | branched chain family amino acid biosynthetic process | [B] |
|  | GO:0055114 | oxidation reduction | [B] |
| - ILVD\_EDD | GO:0003824 | catalytic activity | [M] |
|  | GO:0008152 | metabolic process | [B] |
| - IlvN | GO:0004455 | ketol-acid reductoisomerase activity | [M] |
|  | GO:0008652 | cellular amino acid biosynthetic process | [B] |
|  | GO:0055114 | oxidation reduction | [B] |
| - Init\_tRNA\_PT | GO:0016763 | transferase activity, transferring pentosyl groups | [M] |
| - Inos-1-P\_synth |  |  |  |  |
| - INSIG |  |  |  |  |
| - Interfer-bind |  |  |  |  |
| - Ipi1\_N |  |  |  |  |
| - IstB | GO:0005524 | ATP binding | [M] |
| - K-box | GO:0003700 | transcription factor activity | [M] |
|  | GO:0006355 | regulation of transcription, DNA-dependent | [B] |
|  | GO:0005634 | nucleus | [C] |
| - KAT11 |  |  |  |  |
| - Kinetochor\_Ybp2 |  |  |  |  |
| - Kua-UEV1\_localn |  |  |  |  |
| - Lactate\_perm | GO:0015129 | lactate transmembrane transporter activity | [M] |
|  | GO:0015727 | lactate transport | [B] |
| - Laminin\_EGF |  |  |  |  |
| - LBP\_BPI\_CETP | GO:0008289 | lipid binding | [M] |
| - LBR\_tudor |  |  |  |  |
| - Ldh\_2 | GO:0016491 | oxidoreductase activity | [M] |
|  | GO:0008152 | metabolic process | [B] |
|  | GO:0055114 | oxidation reduction | [B] |
| - LEA\_2 | GO:0009269 | response to desiccation | [B] |
| - LEA\_4 |  |  |  |  |
| - Lectin\_legB | GO:0005488 | binding | [M] |
| - LEH |  |  |  |  |
| - LeuA\_dimer | GO:0003852 | 2-isopropylmalate synthase activity | [M] |
|  | GO:0009098 | leucine biosynthetic process | [B] |
| - LicD |  |  |  |  |
| - Lig\_chan-Glu\_bd |  |  |  |  |
| - Lin0512\_fam |  |  |  |  |
| - Linker\_histone | GO:0003677 | DNA binding | [M] |
|  | GO:0006334 | nucleosome assembly | [B] |
|  | GO:0000786 | nucleosome | [C] |
|  | GO:0005634 | nucleus | [C] |
| - Lip\_A\_acyltrans | GO:0008415 | acyltransferase activity | [M] |
|  | GO:0009244 | lipopolysaccharide core region biosynthetic process | [B] |
|  | GO:0016021 | integral to membrane | [C] |
| - Lipase |  |  |  |  |
| - Lipocalin | GO:0005488 | binding | [M] |
| - Lipoxygenase | GO:0016702 | oxidoreductase activity, acting on single donors with incorporation of molecular oxygen, incorporation of two atoms of oxygen | [M] |
|  | GO:0046872 | metal ion binding | [M] |
|  | GO:0055114 | oxidation reduction | [B] |
| - LMWPc | GO:0004725 | protein tyrosine phosphatase activity | [M] |
|  | GO:0006470 | protein amino acid dephosphorylation | [B] |
| - LpxC | GO:0008759 | UDP-3-O-[3-hydroxymyristoyl] N-acetylglucosamine deacetylase activity | [M] |
|  | GO:0009245 | lipid A biosynthetic process | [B] |
| - LpxK | GO:0005524 | ATP binding | [M] |
|  | GO:0009029 | tetraacyldisaccharide 4'-kinase activity | [M] |
|  | GO:0009245 | lipid A biosynthetic process | [B] |
| - LrgB | GO:0016020 | membrane | [C] |
| - LRRNT |  |  |  |  |
| - LRRNT\_2 |  |  |  |  |
| - LtrA |  |  |  |  |
| - LTV |  |  |  |  |
| - Lum\_binding |  |  |  |  |
| - Lycopene\_cycl | GO:0016705 | oxidoreductase activity, acting on paired donors, with incorporation or reduction of molecular oxygen | [M] |
|  | GO:0016117 | carotenoid biosynthetic process | [B] |
| - LysE | GO:0006865 | amino acid transport | [B] |
|  | GO:0016020 | membrane | [C] |
| - Mac |  |  |  |  |
| - MAD |  |  |  |  |
| - MADF\_DNA\_bdg |  |  |  |  |
| - MAGE |  |  |  |  |
| - Mannitol\_dh | GO:0016491 | oxidoreductase activity | [M] |
|  | GO:0055114 | oxidation reduction | [B] |
| - Mannitol\_dh\_C | GO:0016491 | oxidoreductase activity | [M] |
|  | GO:0050662 | coenzyme binding | [M] |
|  | GO:0055114 | oxidation reduction | [B] |
| - Mannosyl\_trans3 |  |  |  |  |
| - MARVEL | GO:0016020 | membrane | [C] |
| - MBD | GO:0003677 | DNA binding | [M] |
|  | GO:0005634 | nucleus | [C] |
| - MCD | GO:0050080 | malonyl-CoA decarboxylase activity | [M] |
|  | GO:0006633 | fatty acid biosynthetic process | [B] |
| - Med10 | GO:0016455 | RNA polymerase II transcription mediator activity | [M] |
|  | GO:0006357 | regulation of transcription from RNA polymerase II promoter | [B] |
|  | GO:0016592 | mediator complex | [C] |
| - Med11 | GO:0016455 | RNA polymerase II transcription mediator activity | [M] |
|  | GO:0006357 | regulation of transcription from RNA polymerase II promoter | [B] |
|  | GO:0016592 | mediator complex | [C] |
| - Med13\_C |  |  |  |  |
| - Med17 |  |  |  |  |
| - Med18 | GO:0016455 | RNA polymerase II transcription mediator activity | [M] |
|  | GO:0006357 | regulation of transcription from RNA polymerase II promoter | [B] |
|  | GO:0016592 | mediator complex | [C] |
| - Med2 |  |  |  |  |
| - Med20 |  |  |  |  |
| - Med22 |  |  |  |  |
| - Med27 |  |  |  |  |
| - Med4 |  |  |  |  |
| - Med8 |  |  |  |  |
| - MEKHLA |  |  |  |  |
| - Menin | GO:0005634 | nucleus | [C] |
| - Met\_synt\_B12 | GO:0008705 | methionine synthase activity | [M] |
|  | GO:0009086 | methionine biosynthetic process | [B] |
|  | GO:0005622 | intracellular | [C] |
| - META |  |  |  |  |
| - Metallopep |  |  |  |  |
| - Meth\_synt\_1 | GO:0003871 | 5-methyltetrahydropteroyltriglutamate-homocysteine S-methyltransferase activity | [M] |
|  | GO:0008270 | zinc ion binding | [M] |
|  | GO:0008652 | cellular amino acid biosynthetic process | [B] |
| - Meth\_synt\_2 | GO:0003871 | 5-methyltetrahydropteroyltriglutamate-homocysteine S-methyltransferase activity | [M] |
|  | GO:0009086 | methionine biosynthetic process | [B] |
| - Methyltransf\_2 | GO:0008171 | O-methyltransferase activity | [M] |
| - Methyltransf\_3 | GO:0008171 | O-methyltransferase activity | [M] |
| - Methyltransf\_6 |  |  |  |  |
| - Methyltransf\_7 | GO:0008168 | methyltransferase activity | [M] |
| - Methyltransf\_FA |  |  |  |  |
| - Mg-por\_mtran\_C | GO:0046406 | magnesium protoporphyrin IX methyltransferase activity | [M] |
|  | GO:0015979 | photosynthesis | [B] |
|  | GO:0015995 | chlorophyll biosynthetic process | [B] |
| - Mg\_chelatase | GO:0016851 | magnesium chelatase activity | [M] |
|  | GO:0015979 | photosynthesis | [B] |
|  | GO:0015995 | chlorophyll biosynthetic process | [B] |
| - MGAT2 | GO:0008455 | alpha-1,6-mannosylglycoprotein 2-beta-N-acetylglucosaminyltransferase activity | [M] |
|  | GO:0009312 | oligosaccharide biosynthetic process | [B] |
|  | GO:0005795 | Golgi stack | [C] |
|  | GO:0016021 | integral to membrane | [C] |
| - MGDG\_synth | GO:0016758 | transferase activity, transferring hexosyl groups | [M] |
|  | GO:0009247 | glycolipid biosynthetic process | [B] |
| - MgtC | GO:0016020 | membrane | [C] |
| - MgtE | GO:0008324 | cation transmembrane transporter activity | [M] |
|  | GO:0006812 | cation transport | [B] |
| - MHYT |  |  |  |  |
| - MIB\_HERC2 | GO:0004842 | ubiquitin-protein ligase activity | [M] |
|  | GO:0046872 | metal ion binding | [M] |
|  | GO:0016567 | protein ubiquitination | [B] |
| - Microtub\_assoc |  |  |  |  |
| - Mif2 |  |  |  |  |
| - MinC\_C |  |  |  |  |
| - MinE | GO:0032955 | regulation of barrier septum formation | [B] |
|  | GO:0051301 | cell division | [B] |
| - Mis12 | GO:0007049 | cell cycle | [B] |
|  | GO:0007067 | mitosis | [B] |
|  | GO:0000775 | chromosome, centromeric region | [C] |
|  | GO:0005634 | nucleus | [C] |
| - Mis12\_component |  |  |  |  |
| - Mitofilin | GO:0031305 | integral to mitochondrial inner membrane | [C] |
| - MitoNEET\_N | GO:0051537 | 2 iron, 2 sulfur cluster binding | [M] |
|  | GO:0043231 | intracellular membrane-bounded organelle | [C] |
| - MM\_CoA\_mutase | GO:0016866 | intramolecular transferase activity | [M] |
|  | GO:0031419 | cobalamin binding | [M] |
|  | GO:0008152 | metabolic process | [B] |
| - MMS19\_N |  |  |  |  |
| - MoeA\_C | GO:0032324 | molybdopterin cofactor biosynthetic process | [B] |
| - MOFRL |  |  |  |  |
| - MOSC | GO:0003824 | catalytic activity | [M] |
|  | GO:0030151 | molybdenum ion binding | [M] |
|  | GO:0030170 | pyridoxal phosphate binding | [M] |
| - MOSC\_N |  |  |  |  |
| - MPP6 |  |  |  |  |
| - MPPN |  |  |  |  |
| - MR\_MLE |  |  |  |  |
| - MR\_MLE\_N |  |  |  |  |
| - MRC1 |  |  |  |  |
| - MRP-L28 |  |  |  |  |
| - MRP-S33 |  |  |  |  |
| - MRP\_L53 |  |  |  |  |
| - MscL | GO:0005216 | ion channel activity | [M] |
|  | GO:0006810 | transport | [B] |
|  | GO:0016021 | integral to membrane | [C] |
| - MSP | GO:0005509 | calcium ion binding | [M] |
|  | GO:0015979 | photosynthesis | [B] |
|  | GO:0042549 | photosystem II stabilization | [B] |
|  | GO:0009654 | oxygen evolving complex | [C] |
|  | GO:0019898 | extrinsic to membrane | [C] |
| - MTHFR | GO:0004489 | methylenetetrahydrofolate reductase (NADPH) activity | [M] |
|  | GO:0006555 | methionine metabolic process | [B] |
|  | GO:0055114 | oxidation reduction | [B] |
| - MTP18 |  |  |  |  |
| - MttA\_Hcf106 | GO:0008565 | protein transporter activity | [M] |
|  | GO:0015031 | protein transport | [B] |
| - MuDR |  |  |  |  |
| - Multi\_Drug\_Res | GO:0016021 | integral to membrane | [C] |
| - Mur\_ligase\_C | GO:0005524 | ATP binding | [M] |
|  | GO:0016874 | ligase activity | [M] |
|  | GO:0009058 | biosynthetic process | [B] |
| - MutS\_IV | GO:0005524 | ATP binding | [M] |
|  | GO:0030983 | mismatched DNA binding | [M] |
|  | GO:0006298 | mismatch repair | [B] |
| - Myb\_DNA-bind\_2 |  |  |  |  |
| - Myosin\_N | GO:0003774 | motor activity | [M] |
|  | GO:0005524 | ATP binding | [M] |
|  | GO:0016459 | myosin complex | [C] |
| - N1221 |  |  |  |  |
| - Na\_H\_antiport\_1 | GO:0006814 | sodium ion transport | [B] |
|  | GO:0006885 | regulation of pH | [B] |
|  | GO:0016021 | integral to membrane | [C] |
| - Na\_H\_antiporter | GO:0015385 | sodium:hydrogen antiporter activity | [M] |
|  | GO:0006814 | sodium ion transport | [B] |
|  | GO:0006885 | regulation of pH | [B] |
|  | GO:0016021 | integral to membrane | [C] |
| - Na\_Pi\_cotrans | GO:0015321 | sodium-dependent phosphate transmembrane transporter activity | [M] |
|  | GO:0006817 | phosphate transport | [B] |
|  | GO:0016020 | membrane | [C] |
| - NAD\_binding\_3 | GO:0016491 | oxidoreductase activity | [M] |
|  | GO:0050661 | NADP or NADPH binding | [M] |
| - NadA | GO:0008987 | quinolinate synthetase A activity | [M] |
|  | GO:0009435 | NAD biosynthetic process | [B] |
| - NADH-u\_ox-rdase |  |  |  |  |
| - NADH\_ub\_rd\_NUML |  |  |  |  |
| - NAF | GO:0007165 | signal transduction | [B] |
| - NARG2\_C |  |  |  |  |
| - NARP1 |  |  |  |  |
| - Nbl1\_Borealin\_N |  |  |  |  |
| - NCA2 |  |  |  |  |
| - Nckap1 |  |  |  |  |
| - Ndc1\_Nup |  |  |  |  |
| - NDUF\_B7 | GO:0003954 | NADH dehydrogenase activity | [M] |
|  | GO:0008137 | NADH dehydrogenase (ubiquinone) activity | [M] |
|  | GO:0005739 | mitochondrion | [C] |
| - NDUFB10 |  |  |  |  |
| - Ndufs5 |  |  |  |  |
| - Nefa\_Nip30\_N |  |  |  |  |
| - NeuB | GO:0016051 | carbohydrate biosynthetic process | [B] |
| - Neur\_chan\_LBD | GO:0005230 | extracellular ligand-gated ion channel activity | [M] |
|  | GO:0006810 | transport | [B] |
|  | GO:0016020 | membrane | [C] |
| - Neur\_chan\_memb | GO:0006811 | ion transport | [B] |
|  | GO:0016020 | membrane | [C] |
| - Neurochondrin |  |  |  |  |
| - Nexin\_C |  |  |  |  |
| - NHase\_alpha | GO:0003824 | catalytic activity | [M] |
|  | GO:0046914 | transition metal ion binding | [M] |
|  | GO:0006807 | nitrogen compound metabolic process | [B] |
| - NHL |  |  |  |  |
| - Ni\_hydr\_CYTB | GO:0009055 | electron carrier activity | [M] |
|  | GO:0016021 | integral to membrane | [C] |
| - Nicastrin | GO:0016485 | protein processing | [B] |
|  | GO:0016021 | integral to membrane | [C] |
| - NicO | GO:0046872 | metal ion binding | [M] |
|  | GO:0030001 | metal ion transport | [B] |
|  | GO:0055085 | transmembrane transport | [B] |
|  | GO:0016021 | integral to membrane | [C] |
| - NIPSNAP |  |  |  |  |
| - NIR\_SIR | GO:0016491 | oxidoreductase activity | [M] |
|  | GO:0020037 | heme binding | [M] |
|  | GO:0051536 | iron-sulfur cluster binding | [M] |
|  | GO:0055114 | oxidation reduction | [B] |
| - NIR\_SIR\_ferr | GO:0016491 | oxidoreductase activity | [M] |
|  | GO:0055114 | oxidation reduction | [B] |
| - NIT |  |  |  |  |
| - NMT1 |  |  |  |  |
| - Nnf1 |  |  |  |  |
| - NO\_synthase | GO:0004517 | nitric-oxide synthase activity | [M] |
|  | GO:0006809 | nitric oxide biosynthetic process | [B] |
|  | GO:0055114 | oxidation reduction | [B] |
| - Nodulin-like |  |  |  |  |
| - NodZ | GO:0016758 | transferase activity, transferring hexosyl groups | [M] |
|  | GO:0009312 | oligosaccharide biosynthetic process | [B] |
|  | GO:0009877 | nodulation | [B] |
| - NPP1 |  |  |  |  |
| - NPR3 |  |  |  |  |
| - NT5C | GO:0016791 | phosphatase activity | [M] |
| - NTPase\_I-T |  |  |  |  |
| - Nuc\_H\_symport | GO:0005337 | nucleoside transmembrane transporter activity | [M] |
|  | GO:0015858 | nucleoside transport | [B] |
|  | GO:0016021 | integral to membrane | [C] |
| - Nucleoporin\_C |  |  |  |  |
| - Nucleotid\_trans |  |  |  |  |
| - Nup160 |  |  |  |  |
| - NUP50 |  |  |  |  |
| - Nup84\_Nup100 | GO:0006810 | transport | [B] |
|  | GO:0005643 | nuclear pore | [C] |
| - Nup88 |  |  |  |  |
| - NusB | GO:0003723 | RNA binding | [M] |
|  | GO:0006355 | regulation of transcription, DNA-dependent | [B] |
| - NusG | GO:0003711 | transcription elongation regulator activity | [M] |
|  | GO:0032968 | positive regulation of RNA elongation from RNA polymerase II promoter | [B] |
| - OCD\_Mu\_crystall |  |  |  |  |
| - ODV-E18 | GO:0019031 | viral envelope | [C] |
| - Ofd1\_CTDD | GO:0005506 | iron ion binding | [M] |
|  | GO:0016706 | oxidoreductase activity, acting on paired donors, with incorporation or reduction of molecular oxygen, 2-oxoglutarate as one donor, and incorporation of one atom each of oxygen into both donors | [M] |
|  | GO:0031418 | L-ascorbic acid binding | [M] |
|  | GO:0055114 | oxidation reduction | [B] |
| - OHCU\_decarbox |  |  |  |  |
| - OmpA | GO:0009279 | cell outer membrane | [C] |
| - Opi1 |  |  |  |  |
| - OPT | GO:0055085 | transmembrane transport | [B] |
| - OpuAC | GO:0005215 | transporter activity | [M] |
|  | GO:0005488 | binding | [M] |
|  | GO:0006810 | transport | [B] |
| - Orai-1 |  |  |  |  |
| - ORMDL | GO:0016021 | integral to membrane | [C] |
| - ox\_reductase\_C |  |  |  |  |
| - Oxidored\_q4 | GO:0008137 | NADH dehydrogenase (ubiquinone) activity | [M] |
|  | GO:0055114 | oxidation reduction | [B] |
| - Oxidored\_q6 | GO:0008137 | NADH dehydrogenase (ubiquinone) activity | [M] |
|  | GO:0048038 | quinone binding | [M] |
|  | GO:0051539 | 4 iron, 4 sulfur cluster binding | [M] |
|  | GO:0055114 | oxidation reduction | [B] |
| - P-mevalo\_kinase | GO:0004631 | phosphomevalonate kinase activity | [M] |
|  | GO:0006695 | cholesterol biosynthetic process | [B] |
|  | GO:0005737 | cytoplasm | [C] |
| - P2X\_receptor | GO:0004872 | receptor activity | [M] |
|  | GO:0005216 | ion channel activity | [M] |
|  | GO:0005524 | ATP binding | [M] |
|  | GO:0006811 | ion transport | [B] |
|  | GO:0016020 | membrane | [C] |
| - P\_proprotein | GO:0004252 | serine-type endopeptidase activity | [M] |
|  | GO:0006508 | proteolysis | [B] |
| - PA\_decarbox | GO:0016831 | carboxy-lyase activity | [M] |
| - PAN\_2 |  |  |  |  |
| - PaO | GO:0010277 | chlorophyllide a oxygenase activity | [M] |
|  | GO:0055114 | oxidation reduction | [B] |
| - PAP\_fibrillin | GO:0005198 | structural molecule activity | [M] |
| - ParBc | GO:0003677 | DNA binding | [M] |
| - PAS\_2 | GO:0008020 | G-protein coupled photoreceptor activity | [M] |
|  | GO:0007600 | sensory perception | [B] |
|  | GO:0018298 | protein-chromophore linkage | [B] |
|  | GO:0045449 | regulation of transcription | [B] |
| - PAS\_3 |  |  |  |  |
| - PAS\_4 |  |  |  |  |
| - PAT1 |  |  |  |  |
| - PD40 |  |  |  |  |
| - PDH | GO:0004665 | prephenate dehydrogenase (NADP+) activity | [M] |
|  | GO:0006571 | tyrosine biosynthetic process | [B] |
| - PDR\_assoc |  |  |  |  |
| - PDR\_CDR | GO:0005524 | ATP binding | [M] |
|  | GO:0042626 | ATPase activity, coupled to transmembrane movement of substances | [M] |
|  | GO:0006810 | transport | [B] |
|  | GO:0016021 | integral to membrane | [C] |
| - PDT | GO:0004664 | prephenate dehydratase activity | [M] |
|  | GO:0009094 | L-phenylalanine biosynthetic process | [B] |
| - Pec\_lyase\_C |  |  |  |  |
| - Pectate\_lyase | GO:0030570 | pectate lyase activity | [M] |
|  | GO:0005576 | extracellular region | [C] |
| - Pectinesterase | GO:0030599 | pectinesterase activity | [M] |
|  | GO:0042545 | cell wall modification | [B] |
|  | GO:0005618 | cell wall | [C] |
| - Pellino |  |  |  |  |
| - PEMT | GO:0008170 | N-methyltransferase activity | [M] |
|  | GO:0006644 | phospholipid metabolic process | [B] |
| - PEN-2 |  |  |  |  |
| - Peptidase\_C69 | GO:0016805 | dipeptidase activity | [M] |
|  | GO:0006508 | proteolysis | [B] |
| - Peptidase\_M11 |  |  |  |  |
| - Peptidase\_M19 | GO:0008235 | metalloexopeptidase activity | [M] |
|  | GO:0008239 | dipeptidyl-peptidase activity | [M] |
|  | GO:0016805 | dipeptidase activity | [M] |
|  | GO:0006508 | proteolysis | [B] |
| - Peptidase\_M23 |  |  |  |  |
| - Peptidase\_M32 | GO:0004181 | metallocarboxypeptidase activity | [M] |
|  | GO:0006508 | proteolysis | [B] |
| - Peptidase\_M36 |  |  |  |  |
| - Peptidase\_M43 |  |  |  |  |
| - Peptidase\_M6 | GO:0008233 | peptidase activity | [M] |
|  | GO:0006508 | proteolysis | [B] |
| - Peptidase\_M64 |  |  |  |  |
| - Peptidase\_M66 |  |  |  |  |
| - Peptidase\_S41 | GO:0008236 | serine-type peptidase activity | [M] |
|  | GO:0006508 | proteolysis | [B] |
| - Peptidase\_S46 |  |  |  |  |
| - Peptidase\_S49\_N | GO:0004252 | serine-type endopeptidase activity | [M] |
|  | GO:0005886 | plasma membrane | [C] |
| - Peptidase\_S51 | GO:0008236 | serine-type peptidase activity | [M] |
|  | GO:0006508 | proteolysis | [B] |
| - Peptidase\_S66 |  |  |  |  |
| - Peptidase\_U32 | GO:0008233 | peptidase activity | [M] |
|  | GO:0006508 | proteolysis | [B] |
| - Peripla\_BP\_2 | GO:0005381 | iron ion transmembrane transporter activity | [M] |
|  | GO:0006827 | high-affinity iron ion transport | [B] |
| - Peroxidase\_2 | GO:0004601 | peroxidase activity | [M] |
| - PEX-1N | GO:0005515 | protein binding | [M] |
|  | GO:0005524 | ATP binding | [M] |
|  | GO:0007031 | peroxisome organization | [B] |
|  | GO:0005777 | peroxisome | [C] |
| - PFL | GO:0008861 | formate C-acetyltransferase activity | [M] |
|  | GO:0006006 | glucose metabolic process | [B] |
|  | GO:0005737 | cytoplasm | [C] |
| - PGA\_cap |  |  |  |  |
| - PGPGW |  |  |  |  |
| - Phe\_tRNA-synt\_N | GO:0000166 | nucleotide binding | [M] |
|  | GO:0004826 | phenylalanine-tRNA ligase activity | [M] |
|  | GO:0005524 | ATP binding | [M] |
|  | GO:0006412 | translation | [B] |
|  | GO:0006432 | phenylalanyl-tRNA aminoacylation | [B] |
|  | GO:0005737 | cytoplasm | [C] |
| - Pho88 |  |  |  |  |
| - PHP | GO:0003824 | catalytic activity | [M] |
| - Phtf-FEM1B\_bdg |  |  |  |  |
| - Phytochrome | GO:0004872 | receptor activity | [M] |
|  | GO:0008020 | G-protein coupled photoreceptor activity | [M] |
|  | GO:0006355 | regulation of transcription, DNA-dependent | [B] |
|  | GO:0007600 | sensory perception | [B] |
|  | GO:0018298 | protein-chromophore linkage | [B] |
| - PhzC-PhzF | GO:0003824 | catalytic activity | [M] |
|  | GO:0009058 | biosynthetic process | [B] |
| - PI3K\_1B\_p101 |  |  |  |  |
| - PilN |  |  |  |  |
| - PKD |  |  |  |  |
| - Pkr1 |  |  |  |  |
| - PLA2\_B | GO:0004620 | phospholipase activity | [M] |
|  | GO:0009395 | phospholipid catabolic process | [B] |
| - PLA2G12 | GO:0004623 | phospholipase A2 activity | [M] |
|  | GO:0005509 | calcium ion binding | [M] |
|  | GO:0016042 | lipid catabolic process | [B] |
|  | GO:0005576 | extracellular region | [C] |
| - PLAC8 |  |  |  |  |
| - Plant\_tran |  |  |  |  |
| - PLD\_C |  |  |  |  |
| - PLD\_envelope |  |  |  |  |
| - PNGaseA |  |  |  |  |
| - PNPase | GO:0000175 | 3'-5'-exoribonuclease activity | [M] |
|  | GO:0003723 | RNA binding | [M] |
|  | GO:0006396 | RNA processing | [B] |
| - PNPOx\_C | GO:0016638 | oxidoreductase activity, acting on the CH-NH2 group of donors | [M] |
|  | GO:0055114 | oxidation reduction | [B] |
| - Pol\_alpha\_B\_N |  |  |  |  |
| - Polyketide\_cyc2 |  |  |  |  |
| - POTRA\_2 |  |  |  |  |
| - PPV\_E1\_C | GO:0003677 | DNA binding | [M] |
|  | GO:0004003 | ATP-dependent DNA helicase activity | [M] |
|  | GO:0005524 | ATP binding | [M] |
|  | GO:0006260 | DNA replication | [B] |
| - PRA-CH | GO:0004635 | phosphoribosyl-AMP cyclohydrolase activity | [M] |
|  | GO:0000105 | histidine biosynthetic process | [B] |
| - PRA-PH | GO:0004636 | phosphoribosyl-ATP diphosphatase activity | [M] |
|  | GO:0000105 | histidine biosynthetic process | [B] |
| - PRAI | GO:0004640 | phosphoribosylanthranilate isomerase activity | [M] |
|  | GO:0006568 | tryptophan metabolic process | [B] |
| - Presenilin | GO:0007242 | intracellular signaling cascade | [B] |
|  | GO:0016021 | integral to membrane | [C] |
| - PRiA4\_ORF3 |  |  |  |  |
| - Prismane | GO:0016491 | oxidoreductase activity | [M] |
|  | GO:0055114 | oxidation reduction | [B] |
|  | GO:0005737 | cytoplasm | [C] |
| - Pro-kuma\_activ | GO:0008236 | serine-type peptidase activity | [M] |
| - Pro\_racemase | GO:0018112 | proline racemase activity | [M] |
| - Prominin | GO:0016021 | integral to membrane | [C] |
| - PrpF |  |  |  |  |
| - Psb28 | GO:0015979 | photosynthesis | [B] |
|  | GO:0009654 | oxygen evolving complex | [C] |
|  | GO:0016020 | membrane | [C] |
| - PsbM | GO:0019684 | photosynthesis, light reaction | [B] |
|  | GO:0009523 | photosystem II | [C] |
|  | GO:0016021 | integral to membrane | [C] |
| - PsbP | GO:0005509 | calcium ion binding | [M] |
|  | GO:0015979 | photosynthesis | [B] |
|  | GO:0009654 | oxygen evolving complex | [C] |
|  | GO:0019898 | extrinsic to membrane | [C] |
| - PsbQ | GO:0005509 | calcium ion binding | [M] |
|  | GO:0015979 | photosynthesis | [B] |
|  | GO:0009654 | oxygen evolving complex | [C] |
|  | GO:0019898 | extrinsic to membrane | [C] |
| - PsbU | GO:0042549 | photosystem II stabilization | [B] |
|  | GO:0009654 | oxygen evolving complex | [C] |
|  | GO:0019898 | extrinsic to membrane | [C] |
| - PSI\_8 | GO:0015979 | photosynthesis | [B] |
|  | GO:0009522 | photosystem I | [C] |
| - PSI\_PsaE | GO:0009538 | photosystem I reaction center | [C] |
| - PSI\_PsaF | GO:0015979 | photosynthesis | [B] |
|  | GO:0009538 | photosystem I reaction center | [C] |
| - PSP1 |  |  |  |  |
| - PspA\_IM30 |  |  |  |  |
| - PTA\_PTB | GO:0008415 | acyltransferase activity | [M] |
|  | GO:0008152 | metabolic process | [B] |
| - PTR2 | GO:0005215 | transporter activity | [M] |
|  | GO:0006857 | oligopeptide transport | [B] |
|  | GO:0016020 | membrane | [C] |
| - PUCC |  |  |  |  |
| - PXA |  |  |  |  |
| - Pyridox\_oxidase | GO:0010181 | FMN binding | [M] |
| - QRPTase\_C | GO:0004514 | nicotinate-nucleotide diphosphorylase (carboxylating) activity | [M] |
|  | GO:0009435 | NAD biosynthetic process | [B] |
| - QRPTase\_N | GO:0004514 | nicotinate-nucleotide diphosphorylase (carboxylating) activity | [M] |
|  | GO:0009435 | NAD biosynthetic process | [B] |
| - Queuosine\_synth | GO:0016740 | transferase activity | [M] |
|  | GO:0016853 | isomerase activity | [M] |
|  | GO:0008616 | queuosine biosynthetic process | [B] |
| - Rad50\_zn\_hook | GO:0004518 | nuclease activity | [M] |
|  | GO:0005524 | ATP binding | [M] |
|  | GO:0008270 | zinc ion binding | [M] |
|  | GO:0006281 | DNA repair | [B] |
| - Rad52\_Rad22 | GO:0006281 | DNA repair | [B] |
|  | GO:0006310 | DNA recombination | [B] |
| - Raffinose\_syn |  |  |  |  |
| - RAI1 |  |  |  |  |
| - RAI16-like |  |  |  |  |
| - RasGEF\_N | GO:0005085 | guanyl-nucleotide exchange factor activity | [M] |
|  | GO:0051056 | regulation of small GTPase mediated signal transduction | [B] |
|  | GO:0005622 | intracellular | [C] |
| - Rb\_C |  |  |  |  |
| - RbsD\_FucU | GO:0008643 | carbohydrate transport | [B] |
| - RDD |  |  |  |  |
| - RecA | GO:0003697 | single-stranded DNA binding | [M] |
|  | GO:0005524 | ATP binding | [M] |
|  | GO:0006281 | DNA repair | [B] |
|  | GO:0005737 | cytoplasm | [C] |
| - RelA\_SpoT | GO:0015969 | guanosine tetraphosphate metabolic process | [B] |
| - Reprolysin | GO:0004222 | metalloendopeptidase activity | [M] |
|  | GO:0006508 | proteolysis | [B] |
| - RGS | GO:0004871 | signal transducer activity | [M] |
| - RhgB\_N | GO:0016837 | carbon-oxygen lyase activity, acting on polysaccharides | [M] |
|  | GO:0030246 | carbohydrate binding | [M] |
|  | GO:0005975 | carbohydrate metabolic process | [B] |
| - RibD\_C | GO:0008703 | 5-amino-6-(5-phosphoribosylamino)uracil reductase activity | [M] |
|  | GO:0009231 | riboflavin biosynthetic process | [B] |
| - Ribosomal\_L25p | GO:0003735 | structural constituent of ribosome | [M] |
|  | GO:0008097 | 5S rRNA binding | [M] |
|  | GO:0006412 | translation | [B] |
|  | GO:0005622 | intracellular | [C] |
|  | GO:0005840 | ribosome | [C] |
| - Ribosomal\_L34 | GO:0003735 | structural constituent of ribosome | [M] |
|  | GO:0006412 | translation | [B] |
|  | GO:0005622 | intracellular | [C] |
|  | GO:0005840 | ribosome | [C] |
| - Ribosomal\_L41 | GO:0003735 | structural constituent of ribosome | [M] |
|  | GO:0006412 | translation | [B] |
|  | GO:0005840 | ribosome | [C] |
| - Ribosomal\_L9\_C |  |  |  |  |
| - Ribosomal\_S20p | GO:0003723 | RNA binding | [M] |
|  | GO:0003735 | structural constituent of ribosome | [M] |
|  | GO:0006412 | translation | [B] |
|  | GO:0005622 | intracellular | [C] |
|  | GO:0005840 | ribosome | [C] |
| - Ribosomal\_S21 | GO:0003735 | structural constituent of ribosome | [M] |
|  | GO:0006412 | translation | [B] |
|  | GO:0005622 | intracellular | [C] |
|  | GO:0005840 | ribosome | [C] |
| - Ric8 |  |  |  |  |
| - Rif1\_N |  |  |  |  |
| - RmuC |  |  |  |  |
| - RNA\_ligase | GO:0003972 | RNA ligase (ATP) activity | [M] |
|  | GO:0005524 | ATP binding | [M] |
|  | GO:0016874 | ligase activity | [M] |
| - RNA\_pol\_Rpb1\_7 | GO:0003677 | DNA binding | [M] |
|  | GO:0003899 | DNA-directed RNA polymerase activity | [M] |
|  | GO:0006350 | transcription | [B] |
| - RNA\_pol\_Rpc82 | GO:0003677 | DNA binding | [M] |
|  | GO:0003899 | DNA-directed RNA polymerase activity | [M] |
|  | GO:0006350 | transcription | [B] |
| - Rogdi\_lz |  |  |  |  |
| - RPA\_C |  |  |  |  |
| - RPEL |  |  |  |  |
| - RRM\_3 |  |  |  |  |
| - rRNA\_methylase |  |  |  |  |
| - RUN |  |  |  |  |
| - RWP-RK |  |  |  |  |
| - RyR |  |  |  |  |
| - SAICAR\_synt | GO:0004639 | phosphoribosylaminoimidazolesuccinocarboxamide synthase activity | [M] |
|  | GO:0006164 | purine nucleotide biosynthetic process | [B] |
| - SapB\_2 |  |  |  |  |
| - SATase\_N | GO:0009001 | serine O-acetyltransferase activity | [M] |
|  | GO:0006535 | cysteine biosynthetic process from serine | [B] |
|  | GO:0005737 | cytoplasm | [C] |
| - SbcD\_C |  |  |  |  |
| - SBF | GO:0008508 | bile acid:sodium symporter activity | [M] |
|  | GO:0006814 | sodium ion transport | [B] |
|  | GO:0016020 | membrane | [C] |
| - SBP\_bac\_1 | GO:0005215 | transporter activity | [M] |
|  | GO:0006810 | transport | [B] |
| - SBP\_bac\_3 | GO:0005215 | transporter activity | [M] |
|  | GO:0006810 | transport | [B] |
|  | GO:0030288 | outer membrane-bounded periplasmic space | [C] |
| - SBP\_bac\_5 | GO:0005215 | transporter activity | [M] |
|  | GO:0006810 | transport | [B] |
| - Sdh\_cyt | GO:0000104 | succinate dehydrogenase activity | [M] |
|  | GO:0009055 | electron carrier activity | [M] |
|  | GO:0006099 | tricarboxylic acid cycle | [B] |
|  | GO:0016020 | membrane | [C] |
| - Sec39 |  |  |  |  |
| - SecA\_SW | GO:0017038 | protein import | [B] |
|  | GO:0016020 | membrane | [C] |
| - Sep15\_SelM |  |  |  |  |
| - SfsA |  |  |  |  |
| - Sgf11 |  |  |  |  |
| - SH3\_3 |  |  |  |  |
| - She9\_MDM33 |  |  |  |  |
| - ShK |  |  |  |  |
| - Shugoshin\_C | GO:0045132 | meiotic chromosome segregation | [B] |
|  | GO:0000775 | chromosome, centromeric region | [C] |
|  | GO:0005634 | nucleus | [C] |
| - Sigma70\_r1\_2 | GO:0003677 | DNA binding | [M] |
|  | GO:0003700 | transcription factor activity | [M] |
|  | GO:0016987 | sigma factor activity | [M] |
|  | GO:0006352 | transcription initiation | [B] |
|  | GO:0006355 | regulation of transcription, DNA-dependent | [B] |
| - Sigma70\_r2 | GO:0003677 | DNA binding | [M] |
|  | GO:0003700 | transcription factor activity | [M] |
|  | GO:0016987 | sigma factor activity | [M] |
|  | GO:0006352 | transcription initiation | [B] |
|  | GO:0006355 | regulation of transcription, DNA-dependent | [B] |
| - Sigma70\_r4 | GO:0003677 | DNA binding | [M] |
|  | GO:0003700 | transcription factor activity | [M] |
|  | GO:0016987 | sigma factor activity | [M] |
|  | GO:0006352 | transcription initiation | [B] |
|  | GO:0006355 | regulation of transcription, DNA-dependent | [B] |
| - Sigma70\_r4\_2 | GO:0003677 | DNA binding | [M] |
|  | GO:0003700 | transcription factor activity | [M] |
|  | GO:0016987 | sigma factor activity | [M] |
|  | GO:0006352 | transcription initiation | [B] |
|  | GO:0006355 | regulation of transcription, DNA-dependent | [B] |
| - Silic\_transp |  |  |  |  |
| - SK\_channel |  |  |  |  |
| - SKN1 |  |  |  |  |
| - Sm\_multidrug\_ex |  |  |  |  |
| - SMC\_Nse1 |  |  |  |  |
| - SmpB | GO:0003723 | RNA binding | [M] |
|  | GO:0006412 | translation | [B] |
| - SnoaL |  |  |  |  |
| - Sod\_Ni | GO:0004784 | superoxide dismutase activity | [M] |
|  | GO:0016151 | nickel ion binding | [M] |
|  | GO:0016209 | antioxidant activity | [M] |
| - SOUL |  |  |  |  |
| - Spherulin4 |  |  |  |  |
| - Spond\_N |  |  |  |  |
| - SPOUT\_MTase | GO:0008168 | methyltransferase activity | [M] |
|  | GO:0006364 | rRNA processing | [B] |
|  | GO:0005737 | cytoplasm | [C] |
| - SpoVS |  |  |  |  |
| - SR-25 |  |  |  |  |
| - Ssu72 | GO:0004721 | phosphoprotein phosphatase activity | [M] |
|  | GO:0006397 | mRNA processing | [B] |
|  | GO:0005634 | nucleus | [C] |
| - Sterol\_MT\_C | GO:0008168 | methyltransferase activity | [M] |
|  | GO:0006694 | steroid biosynthetic process | [B] |
| - Suc\_Fer-like |  |  |  |  |
| - Sulfotransfer\_2 | GO:0008146 | sulfotransferase activity | [M] |
|  | GO:0016051 | carbohydrate biosynthetic process | [B] |
|  | GO:0005794 | Golgi apparatus | [C] |
|  | GO:0016021 | integral to membrane | [C] |
| - Sulphotransf |  |  |  |  |
| - SURF4 |  |  |  |  |
| - SURNod19 |  |  |  |  |
| - TAF4 | GO:0016986 | transcription initiation factor activity | [M] |
|  | GO:0006352 | transcription initiation | [B] |
|  | GO:0005669 | transcription factor TFIID complex | [C] |
| - TAF8\_C |  |  |  |  |
| - TAFII28 | GO:0003702 | RNA polymerase II transcription factor activity | [M] |
|  | GO:0006367 | transcription initiation from RNA polymerase II promoter | [B] |
|  | GO:0005634 | nucleus | [C] |
| - Tannase |  |  |  |  |
| - Tcp11 |  |  |  |  |
| - TEA | GO:0003700 | transcription factor activity | [M] |
|  | GO:0006355 | regulation of transcription, DNA-dependent | [B] |
|  | GO:0005634 | nucleus | [C] |
| - TehB |  |  |  |  |
| - TENA\_THI-4 |  |  |  |  |
| - TerC | GO:0016021 | integral to membrane | [C] |
| - Tex\_N |  |  |  |  |
| - TFIID-18kDa | GO:0003702 | RNA polymerase II transcription factor activity | [M] |
|  | GO:0006366 | transcription from RNA polymerase II promoter | [B] |
| - TFIIF\_alpha | GO:0003677 | DNA binding | [M] |
|  | GO:0016563 | transcription activator activity | [M] |
|  | GO:0045941 | positive regulation of transcription | [B] |
|  | GO:0005634 | nucleus | [C] |
| - TFIIH\_BTF\_p62\_N |  |  |  |  |
| - TFIIIC\_subunit |  |  |  |  |
| - TFR\_dimer |  |  |  |  |
| - Thaumatin |  |  |  |  |
| - Thi4 | GO:0009228 | thiamin biosynthetic process | [B] |
| - ThiC | GO:0009228 | thiamin biosynthetic process | [B] |
| - ThiG | GO:0009228 | thiamin biosynthetic process | [B] |
| - Thioesterase | GO:0016788 | hydrolase activity, acting on ester bonds | [M] |
|  | GO:0009058 | biosynthetic process | [B] |
| - ThiS | GO:0006790 | sulfur metabolic process | [B] |
| - THUMP |  |  |  |  |
| - ThylakoidFormat |  |  |  |  |
| - TIL |  |  |  |  |
| - TIM21 |  |  |  |  |
| - TIR | GO:0004888 | transmembrane receptor activity | [M] |
|  | GO:0007165 | signal transduction | [B] |
|  | GO:0045087 | innate immune response | [B] |
|  | GO:0031224 | intrinsic to membrane | [C] |
| - TLC | GO:0005471 | ATP:ADP antiporter activity | [M] |
|  | GO:0005524 | ATP binding | [M] |
|  | GO:0006810 | transport | [B] |
|  | GO:0016021 | integral to membrane | [C] |
| - Tmemb\_161AB |  |  |  |  |
| - TMF\_TATA\_bd |  |  |  |  |
| - Tom37\_C |  |  |  |  |
| - TonB\_dep\_Rec | GO:0004872 | receptor activity | [M] |
|  | GO:0005215 | transporter activity | [M] |
|  | GO:0006810 | transport | [B] |
|  | GO:0016020 | membrane | [C] |
| - Topo-VIb\_trans | GO:0003677 | DNA binding | [M] |
|  | GO:0003918 | DNA topoisomerase (ATP-hydrolyzing) activity | [M] |
|  | GO:0006265 | DNA topological change | [B] |
|  | GO:0005694 | chromosome | [C] |
| - Torsin | GO:0005524 | ATP binding | [M] |
|  | GO:0051085 | chaperone mediated protein folding requiring cofactor | [B] |
|  | GO:0005783 | endoplasmic reticulum | [C] |
| - TPMT | GO:0008119 | thiopurine S-methyltransferase activity | [M] |
|  | GO:0008152 | metabolic process | [B] |
|  | GO:0005737 | cytoplasm | [C] |
| - TPPII |  |  |  |  |
| - TPR\_MLP1\_2 | GO:0006606 | protein import into nucleus | [B] |
|  | GO:0005643 | nuclear pore | [C] |
| - TPX2\_importin |  |  |  |  |
| - Transferase | GO:0016747 | transferase activity, transferring acyl groups other than amino-acyl groups | [M] |
| - Transp\_cyt\_pur | GO:0015205 | nucleobase transmembrane transporter activity | [M] |
|  | GO:0015931 | nucleobase, nucleoside, nucleotide and nucleic acid transport | [B] |
|  | GO:0055085 | transmembrane transport | [B] |
|  | GO:0016020 | membrane | [C] |
| - Transthyretin |  |  |  |  |
| - TRCF | GO:0003684 | damaged DNA binding | [M] |
|  | GO:0004386 | helicase activity | [M] |
|  | GO:0005524 | ATP binding | [M] |
|  | GO:0006281 | DNA repair | [B] |
| - Trefoil |  |  |  |  |
| - TRIC |  |  |  |  |
| - TrkA\_C | GO:0008324 | cation transmembrane transporter activity | [M] |
|  | GO:0006813 | potassium ion transport | [B] |
| - TrkA\_N | GO:0006813 | potassium ion transport | [B] |
| - TrkH | GO:0008324 | cation transmembrane transporter activity | [M] |
|  | GO:0006812 | cation transport | [B] |
|  | GO:0055085 | transmembrane transport | [B] |
| - Trp\_syntA | GO:0004834 | tryptophan synthase activity | [M] |
|  | GO:0006568 | tryptophan metabolic process | [B] |
| - Trp\_Tyr\_perm |  |  |  |  |
| - Trs120 |  |  |  |  |
| - TspO\_MBR | GO:0016021 | integral to membrane | [C] |
| - TUG |  |  |  |  |
| - Turandot |  |  |  |  |
| - Tweety |  |  |  |  |
| - TylF |  |  |  |  |
| - Tyrosinase | GO:0016491 | oxidoreductase activity | [M] |
|  | GO:0008152 | metabolic process | [B] |
| - UbiD |  |  |  |  |
| - UDP-g\_GGTase | GO:0003980 | UDP-glucose:glycoprotein glucosyltransferase activity | [M] |
|  | GO:0006486 | protein amino acid glycosylation | [B] |
| - UDPGT | GO:0016758 | transferase activity, transferring hexosyl groups | [M] |
|  | GO:0008152 | metabolic process | [B] |
| - UIM |  |  |  |  |
| - UME | GO:0004674 | protein serine/threonine kinase activity | [M] |
| - UNC-50 |  |  |  |  |
| - UPF0014 |  |  |  |  |
| - UPF0052 |  |  |  |  |
| - UPF0057 | GO:0016021 | integral to membrane | [C] |
| - UPF0060 | GO:0016020 | membrane | [C] |
| - UPF0079 |  |  |  |  |
| - UPF0089 |  |  |  |  |
| - UPF0126 |  |  |  |  |
| - UPF0153 |  |  |  |  |
| - UPF0183 |  |  |  |  |
| - UPF0220 |  |  |  |  |
| - UPF0240 |  |  |  |  |
| - Urb2 |  |  |  |  |
| - Urease\_alpha | GO:0009039 | urease activity | [M] |
|  | GO:0016151 | nickel ion binding | [M] |
|  | GO:0019627 | urea metabolic process | [B] |
| - Urease\_beta | GO:0009039 | urease activity | [M] |
|  | GO:0016151 | nickel ion binding | [M] |
|  | GO:0006807 | nitrogen compound metabolic process | [B] |
| - Urease\_gamma | GO:0009039 | urease activity | [M] |
|  | GO:0016151 | nickel ion binding | [M] |
|  | GO:0006807 | nitrogen compound metabolic process | [B] |
| - UreD | GO:0016151 | nickel ion binding | [M] |
|  | GO:0006807 | nitrogen compound metabolic process | [B] |
| - UreF | GO:0016151 | nickel ion binding | [M] |
|  | GO:0006807 | nitrogen compound metabolic process | [B] |
| - Ureidogly\_hydro | GO:0004848 | ureidoglycolate hydrolase activity | [M] |
|  | GO:0000256 | allantoin catabolic process | [B] |
| - Uricase | GO:0016491 | oxidoreductase activity | [M] |
|  | GO:0006144 | purine base metabolic process | [B] |
|  | GO:0055114 | oxidation reduction | [B] |
| - Uso1\_p115\_head | GO:0006886 | intracellular protein transport | [B] |
|  | GO:0048280 | vesicle fusion with Golgi apparatus | [B] |
|  | GO:0000139 | Golgi membrane | [C] |
|  | GO:0005737 | cytoplasm | [C] |
| - USP7 |  |  |  |  |
| - UT | GO:0015204 | urea transmembrane transporter activity | [M] |
|  | GO:0015840 | urea transport | [B] |
|  | GO:0016021 | integral to membrane | [C] |
| - UvrB |  |  |  |  |
| - UvrC\_HhH\_N | GO:0003677 | DNA binding | [M] |
|  | GO:0004518 | nuclease activity | [M] |
|  | GO:0006289 | nucleotide-excision repair | [B] |
| - UxuA | GO:0008927 | mannonate dehydratase activity | [M] |
|  | GO:0006064 | glucuronate catabolic process | [B] |
| - Val\_tRNA-synt\_C | GO:0000166 | nucleotide binding | [M] |
|  | GO:0004832 | valine-tRNA ligase activity | [M] |
|  | GO:0005524 | ATP binding | [M] |
|  | GO:0006412 | translation | [B] |
|  | GO:0006438 | valyl-tRNA aminoacylation | [B] |
|  | GO:0005737 | cytoplasm | [C] |
| - VDE | GO:0046422 | violaxanthin de-epoxidase activity | [M] |
|  | GO:0055114 | oxidation reduction | [B] |
|  | GO:0009507 | chloroplast | [C] |
| - VKG\_Carbox | GO:0008488 | gamma-glutamyl carboxylase activity | [M] |
|  | GO:0017187 | peptidyl-glutamic acid carboxylation | [B] |
| - Vps36\_ESCRT-II |  |  |  |  |
| - Vps39\_1 |  |  |  |  |
| - Vps5 |  |  |  |  |
| - WD-3 |  |  |  |  |
| - WHEP-TRS | GO:0004812 | aminoacyl-tRNA ligase activity | [M] |
|  | GO:0005524 | ATP binding | [M] |
|  | GO:0006418 | tRNA aminoacylation for protein translation | [B] |
| - WSC |  |  |  |  |
| - X8 |  |  |  |  |
| - Xan\_ur\_permease | GO:0005215 | transporter activity | [M] |
|  | GO:0006810 | transport | [B] |
|  | GO:0055085 | transmembrane transport | [B] |
|  | GO:0016020 | membrane | [C] |
| - XendoU |  |  |  |  |
| - XFP | GO:0016832 | aldehyde-lyase activity | [M] |
|  | GO:0005975 | carbohydrate metabolic process | [B] |
| - XFP\_C | GO:0016832 | aldehyde-lyase activity | [M] |
|  | GO:0005975 | carbohydrate metabolic process | [B] |
| - XFP\_N |  |  |  |  |
| - XG\_FTase | GO:0008107 | galactoside 2-alpha-L-fucosyltransferase activity | [M] |
|  | GO:0042546 | cell wall biogenesis | [B] |
|  | GO:0016020 | membrane | [C] |
| - XPA\_C | GO:0003684 | damaged DNA binding | [M] |
|  | GO:0006289 | nucleotide-excision repair | [B] |
|  | GO:0005634 | nucleus | [C] |
| - XRCC4 | GO:0003677 | DNA binding | [M] |
|  | GO:0005515 | protein binding | [M] |
|  | GO:0006302 | double-strand break repair | [B] |
|  | GO:0006310 | DNA recombination | [B] |
|  | GO:0005634 | nucleus | [C] |
| - Y\_phosphatase2 |  |  |  |  |
| - Yae1\_N |  |  |  |  |
| - Ycf34 |  |  |  |  |
| - YDG\_SRA |  |  |  |  |
| - YHS |  |  |  |  |
| - Yos1 |  |  |  |  |
| - zf-C3H1 |  |  |  |  |
| - zf-C4H2 |  |  |  |  |
| - zf-CHC2 | GO:0003677 | DNA binding | [M] |
|  | GO:0003896 | DNA primase activity | [M] |
|  | GO:0008270 | zinc ion binding | [M] |
|  | GO:0006260 | DNA replication | [B] |
| - zf-CXXC | GO:0003677 | DNA binding | [M] |
|  | GO:0008270 | zinc ion binding | [M] |
| - zf-H2C2 |  |  |  |  |
| - zf-LSD1 |  |  |  |  |
| - zf-NADH-PPase | GO:0016787 | hydrolase activity | [M] |
|  | GO:0046872 | metal ion binding | [M] |
| - zf-TAZ | GO:0003712 | transcription cofactor activity | [M] |
|  | GO:0004402 | histone acetyltransferase activity | [M] |
|  | GO:0008270 | zinc ion binding | [M] |
|  | GO:0006355 | regulation of transcription, DNA-dependent | [B] |
|  | GO:0005634 | nucleus | [C] |
| - Zn\_clus | GO:0003700 | transcription factor activity | [M] |
|  | GO:0008270 | zinc ion binding | [M] |
|  | GO:0006355 | regulation of transcription, DNA-dependent | [B] |
|  | GO:0005634 | nucleus | [C] |

---

## Amniota [eol|tol]

|  |  |  |  |
| --- | --- | --- | --- |
| **Pfam domain(s)** | **GO term acc** | **GO term** | **GO namespace** |
| - ACCA |  |  |  |  |
| - Acetate\_kinase | GO:0016301 | kinase activity | [M] |
|  | GO:0016774 | phosphotransferase activity, carboxyl group as acceptor | [M] |
|  | GO:0008152 | metabolic process | [B] |
|  | GO:0016310 | phosphorylation | [B] |
|  | GO:0005622 | intracellular | [C] |
| - AcetylCoA\_hydro | GO:0003824 | catalytic activity | [M] |
|  | GO:0006084 | acetyl-CoA metabolic process | [B] |
| - ACR\_tran | GO:0005215 | transporter activity | [M] |
|  | GO:0006810 | transport | [B] |
|  | GO:0016020 | membrane | [C] |
| - Acyl-CoA\_dh\_2 |  |  |  |  |
| - ApbA |  |  |  |  |
| - ApbA\_C | GO:0016491 | oxidoreductase activity | [M] |
|  | GO:0050661 | NADP or NADPH binding | [M] |
|  | GO:0055114 | oxidation reduction | [B] |
| - ArsC |  |  |  |  |
| - Asp\_Glu\_race |  |  |  |  |
| - Bac\_DNA\_binding | GO:0003677 | DNA binding | [M] |
| - Bac\_DnaA |  |  |  |  |
| - Bac\_export\_2 | GO:0009306 | protein secretion | [B] |
|  | GO:0016020 | membrane | [C] |
| - Bac\_luciferase |  |  |  |  |
| - Bac\_transf |  |  |  |  |
| - BCA\_ABC\_TP\_C |  |  |  |  |
| - BPD\_transp\_1 | GO:0005215 | transporter activity | [M] |
|  | GO:0006810 | transport | [B] |
|  | GO:0016020 | membrane | [C] |
| - BPD\_transp\_2 | GO:0005215 | transporter activity | [M] |
|  | GO:0006810 | transport | [B] |
|  | GO:0016020 | membrane | [C] |
| - Bre5 |  |  |  |  |
| - Bug | GO:0030288 | outer membrane-bounded periplasmic space | [C] |
| - But2 |  |  |  |  |
| - Bvg\_acc\_factor | GO:0016563 | transcription activator activity | [M] |
|  | GO:0045941 | positive regulation of transcription | [B] |
| - CheR\_N | GO:0008757 | S-adenosylmethionine-dependent methyltransferase activity | [M] |
| - CheW | GO:0004871 | signal transducer activity | [M] |
|  | GO:0006935 | chemotaxis | [B] |
|  | GO:0007165 | signal transduction | [B] |
|  | GO:0005622 | intracellular | [C] |
| - Chorismate\_bind |  |  |  |  |
| - Competence\_A |  |  |  |  |
| - Condensation |  |  |  |  |
| - CorC\_HlyC |  |  |  |  |
| - COXG |  |  |  |  |
| - Creatininase |  |  |  |  |
| - Crp | GO:0003700 | transcription factor activity | [M] |
|  | GO:0006355 | regulation of transcription, DNA-dependent | [B] |
|  | GO:0005622 | intracellular | [C] |
| - Cyclase |  |  |  |  |
| - Cytochrom\_D1 |  |  |  |  |
| - DAP\_epimerase | GO:0008837 | diaminopimelate epimerase activity | [M] |
|  | GO:0009089 | lysine biosynthetic process via diaminopimelate | [B] |
|  | GO:0005737 | cytoplasm | [C] |
| - DegT\_DnrJ\_EryC1 |  |  |  |  |
| - DHBP\_synthase | GO:0008686 | 3,4-dihydroxy-2-butanone-4-phosphate synthase activity | [M] |
|  | GO:0009231 | riboflavin biosynthetic process | [B] |
| - Dioxygenase\_C | GO:0003824 | catalytic activity | [M] |
|  | GO:0008199 | ferric iron binding | [M] |
|  | GO:0006725 | cellular aromatic compound metabolic process | [B] |
|  | GO:0055114 | oxidation reduction | [B] |
| - Dioxygenase\_N | GO:0005506 | iron ion binding | [M] |
|  | GO:0018576 | catechol 1,2-dioxygenase activity | [M] |
|  | GO:0009712 | catechol metabolic process | [B] |
|  | GO:0055114 | oxidation reduction | [B] |
| - DNA\_pol3\_gamma3 |  |  |  |  |
| - DNA\_pol\_B\_2 | GO:0000166 | nucleotide binding | [M] |
|  | GO:0003677 | DNA binding | [M] |
|  | GO:0003887 | DNA-directed DNA polymerase activity | [M] |
|  | GO:0008408 | 3'-5' exonuclease activity | [M] |
|  | GO:0006260 | DNA replication | [B] |
| - DsbD | GO:0017004 | cytochrome complex assembly | [B] |
|  | GO:0055114 | oxidation reduction | [B] |
|  | GO:0016020 | membrane | [C] |
| - DUF1078 | GO:0019861 | flagellum | [C] |
| - DUF1111 |  |  |  |  |
| - DUF1173 |  |  |  |  |
| - DUF1239 |  |  |  |  |
| - DUF126 |  |  |  |  |
| - DUF1759 |  |  |  |  |
| - DUF1814 |  |  |  |  |
| - DUF1881 |  |  |  |  |
| - DUF1932 |  |  |  |  |
| - DUF205 | GO:0005886 | plasma membrane | [C] |
| - DUF23 |  |  |  |  |
| - DUF2405 |  |  |  |  |
| - DUF2843 |  |  |  |  |
| - DUF2867 |  |  |  |  |
| - DUF3105 |  |  |  |  |
| - DUF336 |  |  |  |  |
| - DUF395 |  |  |  |  |
| - DUF411 |  |  |  |  |
| - DUF455 |  |  |  |  |
| - DUF484 |  |  |  |  |
| - DUF520 |  |  |  |  |
| - DUF521 |  |  |  |  |
| - DUF659 |  |  |  |  |
| - DUF820 |  |  |  |  |
| - DUF849 |  |  |  |  |
| - DUF917 |  |  |  |  |
| - DXP\_redisom\_C | GO:0030604 | 1-deoxy-D-xylulose-5-phosphate reductoisomerase activity | [M] |
|  | GO:0046872 | metal ion binding | [M] |
|  | GO:0008299 | isoprenoid biosynthetic process | [B] |
|  | GO:0055114 | oxidation reduction | [B] |
| - DXP\_reductoisom | GO:0030604 | 1-deoxy-D-xylulose-5-phosphate reductoisomerase activity | [M] |
|  | GO:0046872 | metal ion binding | [M] |
|  | GO:0008299 | isoprenoid biosynthetic process | [B] |
|  | GO:0055114 | oxidation reduction | [B] |
| - EAL |  |  |  |  |
| - EPSP\_synthase | GO:0016765 | transferase activity, transferring alkyl or aryl (other than methyl) groups | [M] |
| - ExbD | GO:0005215 | transporter activity | [M] |
|  | GO:0006810 | transport | [B] |
|  | GO:0016020 | membrane | [C] |
| - Exonuc\_VII\_L |  |  |  |  |
| - FCD |  |  |  |  |
| - FecR |  |  |  |  |
| - FGase |  |  |  |  |
| - FHIPEP | GO:0009306 | protein secretion | [B] |
|  | GO:0016020 | membrane | [C] |
| - FIST |  |  |  |  |
| - Flg\_bb\_rod | GO:0003774 | motor activity | [M] |
|  | GO:0005198 | structural molecule activity | [M] |
|  | GO:0001539 | ciliary or flagellar motility | [B] |
|  | GO:0009288 | bacterial-type flagellum | [C] |
| - FtsX | GO:0016020 | membrane | [C] |
| - GcpE | GO:0046429 | 4-hydroxy-3-methylbut-2-en-1-yl diphosphate synthase activity | [M] |
|  | GO:0016114 | terpenoid biosynthetic process | [B] |
|  | GO:0055114 | oxidation reduction | [B] |
| - GGDEF |  |  |  |  |
| - Glu\_cys\_ligase | GO:0004357 | glutamate-cysteine ligase activity | [M] |
|  | GO:0006750 | glutathione biosynthetic process | [B] |
| - GlutR\_N | GO:0008883 | glutamyl-tRNA reductase activity | [M] |
|  | GO:0050661 | NADP or NADPH binding | [M] |
|  | GO:0033014 | tetrapyrrole biosynthetic process | [B] |
|  | GO:0055114 | oxidation reduction | [B] |
| - Glyco\_hydro\_3 | GO:0004553 | hydrolase activity, hydrolyzing O-glycosyl compounds | [M] |
|  | GO:0005975 | carbohydrate metabolic process | [B] |
| - Glyco\_hydro\_3\_C | GO:0004553 | hydrolase activity, hydrolyzing O-glycosyl compounds | [M] |
|  | GO:0005975 | carbohydrate metabolic process | [B] |
| - Glyco\_hydro\_68 | GO:0050053 | levansucrase activity | [M] |
|  | GO:0007587 | sugar utilization | [B] |
| - GntR | GO:0003700 | transcription factor activity | [M] |
|  | GO:0006355 | regulation of transcription, DNA-dependent | [B] |
|  | GO:0005622 | intracellular | [C] |
| - GreA\_GreB | GO:0003677 | DNA binding | [M] |
|  | GO:0003711 | transcription elongation regulator activity | [M] |
|  | GO:0006355 | regulation of transcription, DNA-dependent | [B] |
| - GreA\_GreB\_N | GO:0003677 | DNA binding | [M] |
|  | GO:0003711 | transcription elongation regulator activity | [M] |
|  | GO:0006355 | regulation of transcription, DNA-dependent | [B] |
| - GSPII\_E | GO:0005524 | ATP binding | [M] |
|  | GO:0006810 | transport | [B] |
|  | GO:0005622 | intracellular | [C] |
| - GSPII\_E\_N | GO:0005524 | ATP binding | [M] |
|  | GO:0006810 | transport | [B] |
| - GSPII\_F |  |  |  |  |
| - GSPII\_G |  |  |  |  |
| - GTP\_cyclohydro2 | GO:0003935 | GTP cyclohydrolase II activity | [M] |
|  | GO:0009231 | riboflavin biosynthetic process | [B] |
| - Haemagg\_act | GO:0005488 | binding | [M] |
| - HAMP | GO:0004871 | signal transducer activity | [M] |
|  | GO:0007165 | signal transduction | [B] |
|  | GO:0016021 | integral to membrane | [C] |
| - HipA\_C |  |  |  |  |
| - HipA\_N |  |  |  |  |
| - HisKA | GO:0000155 | two-component sensor activity | [M] |
|  | GO:0007165 | signal transduction | [B] |
|  | GO:0016020 | membrane | [C] |
| - Histidinol\_dh | GO:0004399 | histidinol dehydrogenase activity | [M] |
|  | GO:0008270 | zinc ion binding | [M] |
|  | GO:0051287 | NAD or NADH binding | [M] |
|  | GO:0000105 | histidine biosynthetic process | [B] |
| - HpaB\_N |  |  |  |  |
| - Hpt | GO:0004871 | signal transducer activity | [M] |
|  | GO:0000160 | two-component signal transduction system (phosphorelay) | [B] |
| - HTH\_1 | GO:0003700 | transcription factor activity | [M] |
|  | GO:0006355 | regulation of transcription, DNA-dependent | [B] |
| - HTH\_6 | GO:0003700 | transcription factor activity | [M] |
|  | GO:0006355 | regulation of transcription, DNA-dependent | [B] |
| - HTH\_8 | GO:0003700 | transcription factor activity | [M] |
|  | GO:0006355 | regulation of transcription, DNA-dependent | [B] |
| - HTH\_AraC | GO:0003700 | transcription factor activity | [M] |
|  | GO:0043565 | sequence-specific DNA binding | [M] |
|  | GO:0006355 | regulation of transcription, DNA-dependent | [B] |
|  | GO:0005622 | intracellular | [C] |
| - HTH\_IclR | GO:0003677 | DNA binding | [M] |
|  | GO:0006355 | regulation of transcription, DNA-dependent | [B] |
| - HxlR |  |  |  |  |
| - ICL | GO:0003824 | catalytic activity | [M] |
|  | GO:0008152 | metabolic process | [B] |
| - IclR |  |  |  |  |
| - IDH | GO:0004450 | isocitrate dehydrogenase (NADP+) activity | [M] |
|  | GO:0006099 | tricarboxylic acid cycle | [B] |
|  | GO:0055114 | oxidation reduction | [B] |
| - IGPS |  |  |  |  |
| - ILVD\_EDD | GO:0003824 | catalytic activity | [M] |
|  | GO:0008152 | metabolic process | [B] |
| - IstB | GO:0005524 | ATP binding | [M] |
| - IU\_nuc\_hydro |  |  |  |  |
| - LacI | GO:0003700 | transcription factor activity | [M] |
|  | GO:0006355 | regulation of transcription, DNA-dependent | [B] |
|  | GO:0005622 | intracellular | [C] |
| - Ldh\_2 | GO:0016491 | oxidoreductase activity | [M] |
|  | GO:0008152 | metabolic process | [B] |
|  | GO:0055114 | oxidation reduction | [B] |
| - LEA\_2 | GO:0009269 | response to desiccation | [B] |
| - LolA | GO:0015031 | protein transport | [B] |
|  | GO:0030288 | outer membrane-bounded periplasmic space | [C] |
| - LpxC | GO:0008759 | UDP-3-O-[3-hydroxymyristoyl] N-acetylglucosamine deacetylase activity | [M] |
|  | GO:0009245 | lipid A biosynthetic process | [B] |
| - LpxK | GO:0005524 | ATP binding | [M] |
|  | GO:0009029 | tetraacyldisaccharide 4'-kinase activity | [M] |
|  | GO:0009245 | lipid A biosynthetic process | [B] |
| - Lysine\_decarbox |  |  |  |  |
| - LysR\_substrate |  |  |  |  |
| - LytTR |  |  |  |  |
| - MCPsignal | GO:0004871 | signal transducer activity | [M] |
|  | GO:0006935 | chemotaxis | [B] |
|  | GO:0007165 | signal transduction | [B] |
|  | GO:0016020 | membrane | [C] |
| - MerR | GO:0003700 | transcription factor activity | [M] |
|  | GO:0006355 | regulation of transcription, DNA-dependent | [B] |
| - MerR-DNA-bind |  |  |  |  |
| - MerT | GO:0015097 | mercury ion transmembrane transporter activity | [M] |
|  | GO:0015694 | mercury ion transport | [B] |
|  | GO:0016020 | membrane | [C] |
| - MG1 |  |  |  |  |
| - Molydop\_binding | GO:0016491 | oxidoreductase activity | [M] |
|  | GO:0030151 | molybdenum ion binding | [M] |
| - MotA\_ExbB | GO:0008565 | protein transporter activity | [M] |
|  | GO:0006810 | transport | [B] |
|  | GO:0016020 | membrane | [C] |
| - MtfA |  |  |  |  |
| - Muc\_lac\_enz |  |  |  |  |
| - MurB\_C | GO:0008762 | UDP-N-acetylmuramate dehydrogenase activity | [M] |
|  | GO:0009252 | peptidoglycan biosynthetic process | [B] |
|  | GO:0055114 | oxidation reduction | [B] |
| - N6\_Mtase | GO:0003677 | DNA binding | [M] |
|  | GO:0008170 | N-methyltransferase activity | [M] |
|  | GO:0006306 | DNA methylation | [B] |
| - N6\_N4\_Mtase | GO:0003677 | DNA binding | [M] |
|  | GO:0008170 | N-methyltransferase activity | [M] |
|  | GO:0006306 | DNA methylation | [B] |
| - N\_methyl |  |  |  |  |
| - NadA | GO:0008987 | quinolinate synthetase A activity | [M] |
|  | GO:0009435 | NAD biosynthetic process | [B] |
| - Ni\_hydr\_CYTB | GO:0009055 | electron carrier activity | [M] |
|  | GO:0016021 | integral to membrane | [C] |
| - NikM |  |  |  |  |
| - NMT1 |  |  |  |  |
| - NPD | GO:0018580 | 2-nitropropane dioxygenase activity | [M] |
|  | GO:0055114 | oxidation reduction | [B] |
| - NrfD |  |  |  |  |
| - NusB | GO:0003723 | RNA binding | [M] |
|  | GO:0006355 | regulation of transcription, DNA-dependent | [B] |
| - OEP | GO:0005215 | transporter activity | [M] |
|  | GO:0006810 | transport | [B] |
| - oligo\_HPY | GO:0000166 | nucleotide binding | [M] |
|  | GO:0005524 | ATP binding | [M] |
|  | GO:0015833 | peptide transport | [B] |
| - OmpA | GO:0009279 | cell outer membrane | [C] |
| - OmpH | GO:0005515 | protein binding | [M] |
| - OsmC | GO:0006950 | response to stress | [B] |
| - PD40 |  |  |  |  |
| - Peptidase\_A17 |  |  |  |  |
| - Peptidase\_C11 |  |  |  |  |
| - Peptidase\_M75 |  |  |  |  |
| - Peptidase\_S11 | GO:0009002 | serine-type D-Ala-D-Ala carboxypeptidase activity | [M] |
|  | GO:0006508 | proteolysis | [B] |
| - Peptidase\_S49 | GO:0008233 | peptidase activity | [M] |
|  | GO:0006508 | proteolysis | [B] |
| - Peripla\_BP\_1 |  |  |  |  |
| - Peripla\_BP\_2 | GO:0005381 | iron ion transmembrane transporter activity | [M] |
|  | GO:0006827 | high-affinity iron ion transport | [B] |
| - Phasin\_2 |  |  |  |  |
| - Phos\_pyr\_kin |  |  |  |  |
| - Pilin | GO:0007155 | cell adhesion | [B] |
|  | GO:0009289 | pilus | [C] |
| - PIN |  |  |  |  |
| - Plug | GO:0004872 | receptor activity | [M] |
|  | GO:0005215 | transporter activity | [M] |
|  | GO:0006810 | transport | [B] |
|  | GO:0016020 | membrane | [C] |
| - PmbA\_TldD |  |  |  |  |
| - Porin\_1 | GO:0005215 | transporter activity | [M] |
|  | GO:0006810 | transport | [B] |
|  | GO:0016020 | membrane | [C] |
| - PPK2 |  |  |  |  |
| - Pro\_CA | GO:0004089 | carbonate dehydratase activity | [M] |
|  | GO:0008270 | zinc ion binding | [M] |
|  | GO:0015976 | carbon utilization | [B] |
| - PSP1 |  |  |  |  |
| - PUCC |  |  |  |  |
| - Queuosine\_synth | GO:0016740 | transferase activity | [M] |
|  | GO:0016853 | isomerase activity | [M] |
|  | GO:0008616 | queuosine biosynthetic process | [B] |
| - RdgC | GO:0006310 | DNA recombination | [B] |
| - RelA\_SpoT | GO:0015969 | guanosine tetraphosphate metabolic process | [B] |
| - Rep\_3 | GO:0003887 | DNA-directed DNA polymerase activity | [M] |
|  | GO:0006270 | DNA replication initiation | [B] |
|  | GO:0005727 | extrachromosomal circular DNA | [C] |
| - Resolvase | GO:0000150 | recombinase activity | [M] |
|  | GO:0003677 | DNA binding | [M] |
|  | GO:0006310 | DNA recombination | [B] |
| - Rho\_N | GO:0003715 | transcription termination factor activity | [M] |
|  | GO:0006353 | transcription termination | [B] |
| - RibD\_C | GO:0008703 | 5-amino-6-(5-phosphoribosylamino)uracil reductase activity | [M] |
|  | GO:0009231 | riboflavin biosynthetic process | [B] |
| - Ribonuclease | GO:0003723 | RNA binding | [M] |
|  | GO:0004521 | endoribonuclease activity | [M] |
| - Ribosomal\_L25p | GO:0003735 | structural constituent of ribosome | [M] |
|  | GO:0008097 | 5S rRNA binding | [M] |
|  | GO:0006412 | translation | [B] |
|  | GO:0005622 | intracellular | [C] |
|  | GO:0005840 | ribosome | [C] |
| - Ribosomal\_L31 | GO:0003735 | structural constituent of ribosome | [M] |
|  | GO:0006412 | translation | [B] |
|  | GO:0005622 | intracellular | [C] |
|  | GO:0005840 | ribosome | [C] |
| - Ring\_hydroxyl\_A | GO:0005506 | iron ion binding | [M] |
|  | GO:0016708 | oxidoreductase activity, acting on paired donors, with incorporation or reduction of molecular oxygen, NADH or NADPH as one donor, and incorporation of two atoms of oxygen into one donor | [M] |
|  | GO:0051537 | 2 iron, 2 sulfur cluster binding | [M] |
|  | GO:0019439 | aromatic compound catabolic process | [B] |
|  | GO:0055114 | oxidation reduction | [B] |
| - RmuC |  |  |  |  |
| - RNA\_pol\_A\_CTD | GO:0003677 | DNA binding | [M] |
|  | GO:0003899 | DNA-directed RNA polymerase activity | [M] |
|  | GO:0006350 | transcription | [B] |
| - RNase\_E\_G |  |  |  |  |
| - RuvC | GO:0004520 | endodeoxyribonuclease activity | [M] |
|  | GO:0006281 | DNA repair | [B] |
|  | GO:0006310 | DNA recombination | [B] |
| - SBP\_bac\_1 | GO:0005215 | transporter activity | [M] |
|  | GO:0006810 | transport | [B] |
| - SBP\_bac\_5 | GO:0005215 | transporter activity | [M] |
|  | GO:0006810 | transport | [B] |
| - SBP\_bac\_7 | GO:0006810 | transport | [B] |
|  | GO:0030288 | outer membrane-bounded periplasmic space | [C] |
| - Sec\_GG | GO:0008565 | protein transporter activity | [M] |
|  | GO:0015628 | protein secretion by the type II secretion system | [B] |
|  | GO:0015627 | type II protein secretion system complex | [C] |
| - SecD\_SecF | GO:0008565 | protein transporter activity | [M] |
|  | GO:0015628 | protein secretion by the type II secretion system | [B] |
|  | GO:0015627 | type II protein secretion system complex | [C] |
| - Secretin | GO:0009306 | protein secretion | [B] |
| - Shikimate\_DH | GO:0004764 | shikimate 5-dehydrogenase activity | [M] |
|  | GO:0055114 | oxidation reduction | [B] |
|  | GO:0005737 | cytoplasm | [C] |
| - Shikimate\_dh\_N |  |  |  |  |
| - Sigma54\_activat | GO:0005524 | ATP binding | [M] |
|  | GO:0008134 | transcription factor binding | [M] |
|  | GO:0006355 | regulation of transcription, DNA-dependent | [B] |
|  | GO:0005622 | intracellular | [C] |
| - Sigma70\_r2 | GO:0003677 | DNA binding | [M] |
|  | GO:0003700 | transcription factor activity | [M] |
|  | GO:0016987 | sigma factor activity | [M] |
|  | GO:0006352 | transcription initiation | [B] |
|  | GO:0006355 | regulation of transcription, DNA-dependent | [B] |
| - Sigma70\_r3 | GO:0003677 | DNA binding | [M] |
|  | GO:0003700 | transcription factor activity | [M] |
|  | GO:0016987 | sigma factor activity | [M] |
|  | GO:0006352 | transcription initiation | [B] |
|  | GO:0006355 | regulation of transcription, DNA-dependent | [B] |
| - Sigma70\_r4 | GO:0003677 | DNA binding | [M] |
|  | GO:0003700 | transcription factor activity | [M] |
|  | GO:0016987 | sigma factor activity | [M] |
|  | GO:0006352 | transcription initiation | [B] |
|  | GO:0006355 | regulation of transcription, DNA-dependent | [B] |
| - Sigma70\_r4\_2 | GO:0003677 | DNA binding | [M] |
|  | GO:0003700 | transcription factor activity | [M] |
|  | GO:0016987 | sigma factor activity | [M] |
|  | GO:0006352 | transcription initiation | [B] |
|  | GO:0006355 | regulation of transcription, DNA-dependent | [B] |
| - SirA | GO:0005515 | protein binding | [M] |
|  | GO:0016783 | sulfurtransferase activity | [M] |
|  | GO:0008033 | tRNA processing | [B] |
|  | GO:0005737 | cytoplasm | [C] |
| - SpoA |  |  |  |  |
| - Sugar-bind | GO:0030246 | carbohydrate binding | [M] |
|  | GO:0030528 | transcription regulator activity | [M] |
| - TauE |  |  |  |  |
| - TerC | GO:0016021 | integral to membrane | [C] |
| - Terminase\_GpA |  |  |  |  |
| - TetR\_N | GO:0003700 | transcription factor activity | [M] |
|  | GO:0006355 | regulation of transcription, DNA-dependent | [B] |
| - ThiC | GO:0009228 | thiamin biosynthetic process | [B] |
| - ThiG | GO:0009228 | thiamin biosynthetic process | [B] |
| - Thr\_dehydrat\_C | GO:0004794 | L-threonine ammonia-lyase activity | [M] |
|  | GO:0009097 | isoleucine biosynthetic process | [B] |
| - TMP-TENI | GO:0004789 | thiamin-phosphate diphosphorylase activity | [M] |
|  | GO:0009228 | thiamin biosynthetic process | [B] |
| - TOBE\_2 | GO:0005215 | transporter activity | [M] |
|  | GO:0005524 | ATP binding | [M] |
|  | GO:0016820 | hydrolase activity, acting on acid anhydrides, catalyzing transmembrane movement of substances | [M] |
|  | GO:0006810 | transport | [B] |
|  | GO:0043190 | ATP-binding cassette (ABC) transporter complex | [C] |
| - TolB\_N | GO:0015031 | protein transport | [B] |
|  | GO:0042597 | periplasmic space | [C] |
| - TonB | GO:0005381 | iron ion transmembrane transporter activity | [M] |
|  | GO:0006826 | iron ion transport | [B] |
|  | GO:0030288 | outer membrane-bounded periplasmic space | [C] |
| - TraG\_N |  |  |  |  |
| - Trans\_reg\_C | GO:0000156 | two-component response regulator activity | [M] |
|  | GO:0003677 | DNA binding | [M] |
|  | GO:0000160 | two-component signal transduction system (phosphorelay) | [B] |
|  | GO:0006355 | regulation of transcription, DNA-dependent | [B] |
| - Transformer | GO:0006397 | mRNA processing | [B] |
|  | GO:0046660 | female sex differentiation | [B] |
|  | GO:0005634 | nucleus | [C] |
| - Transgly | GO:0003824 | catalytic activity | [M] |
|  | GO:0009252 | peptidoglycan biosynthetic process | [B] |
|  | GO:0009274 | peptidoglycan-based cell wall | [C] |
| - Trp\_syntA | GO:0004834 | tryptophan synthase activity | [M] |
|  | GO:0006568 | tryptophan metabolic process | [B] |
| - UPF0075 | GO:0005524 | ATP binding | [M] |
|  | GO:0016773 | phosphotransferase activity, alcohol group as acceptor | [M] |
|  | GO:0006040 | amino sugar metabolic process | [B] |
|  | GO:0009254 | peptidoglycan turnover | [B] |
| - UPF0227 |  |  |  |  |
| - Usp | GO:0006950 | response to stress | [B] |
| - YaeQ |  |  |  |  |
| - YajC |  |  |  |  |
| - YceI |  |  |  |  |

---

## Amoebozoa [eol|tol]

|  |  |  |  |
| --- | --- | --- | --- |
| **Pfam domain(s)** | **GO term acc** | **GO term** | **GO namespace** |
| - 2CSK\_N |  |  |  |  |
| - 3-dmu-9\_3-mt |  |  |  |  |
| - 3-PAP |  |  |  |  |
| - 5-FTHF\_cyc-lig | GO:0005524 | ATP binding | [M] |
|  | GO:0030272 | 5-formyltetrahydrofolate cyclo-ligase activity | [M] |
|  | GO:0009396 | folic acid and derivative biosynthetic process | [B] |
| - 5\_nucleotid\_C | GO:0016787 | hydrolase activity | [M] |
|  | GO:0009166 | nucleotide catabolic process | [B] |
| - 7TM\_GPCR\_Srsx |  |  |  |  |
| - 7TM\_GPCR\_Srv |  |  |  |  |
| - 7TMR-DISM\_7TM |  |  |  |  |
| - A2M\_comp | GO:0005615 | extracellular space | [C] |
| - A2M\_recep | GO:0005515 | protein binding | [M] |
|  | GO:0005576 | extracellular region | [C] |
| - AAA-ATPase\_like |  |  |  |  |
| - AAL\_decarboxy | GO:0047605 | acetolactate decarboxylase activity | [M] |
|  | GO:0019751 | polyol metabolic process | [B] |
|  | GO:0005789 | endoplasmic reticulum membrane | [C] |
| - AATase | GO:0004026 | alcohol O-acetyltransferase activity | [M] |
|  | GO:0006066 | alcohol metabolic process | [B] |
| - ABC\_ATPase |  |  |  |  |
| - ABC\_transp\_aux |  |  |  |  |
| - AbfB | GO:0046556 | alpha-N-arabinofuranosidase activity | [M] |
|  | GO:0046373 | L-arabinose metabolic process | [B] |
| - ACCA |  |  |  |  |
| - Acid\_phosphat\_B | GO:0003993 | acid phosphatase activity | [M] |
| - ACP\_syn\_III | GO:0004315 | 3-oxoacyl-[acyl-carrier-protein] synthase activity | [M] |
|  | GO:0006633 | fatty acid biosynthetic process | [B] |
| - ACR\_tran | GO:0005215 | transporter activity | [M] |
|  | GO:0006810 | transport | [B] |
|  | GO:0016020 | membrane | [C] |
| - ACT | GO:0016597 | amino acid binding | [M] |
|  | GO:0008152 | metabolic process | [B] |
| - Acyl-ACP\_TE |  |  |  |  |
| - Acyl\_CoA\_thio | GO:0016291 | acyl-CoA thioesterase activity | [M] |
|  | GO:0006637 | acyl-CoA metabolic process | [B] |
| - AcylCoA\_DH\_N |  |  |  |  |
| - Acylphosphatase |  |  |  |  |
| - Ada\_Zn\_binding | GO:0003677 | DNA binding | [M] |
|  | GO:0008168 | methyltransferase activity | [M] |
|  | GO:0008270 | zinc ion binding | [M] |
|  | GO:0006281 | DNA repair | [B] |
|  | GO:0006355 | regulation of transcription, DNA-dependent | [B] |
| - ADC | GO:0016831 | carboxy-lyase activity | [M] |
| - Adeno\_E1B\_55K |  |  |  |  |
| - ADIP |  |  |  |  |
| - ADK\_lid | GO:0004017 | adenylate kinase activity | [M] |
| - ADSL\_C |  |  |  |  |
| - Aegerolysin | GO:0019836 | hemolysis by symbiont of host erythrocytes | [B] |
|  | GO:0030582 | fruiting body development | [B] |
| - Aerolysin | GO:0009405 | pathogenesis | [B] |
|  | GO:0005576 | extracellular region | [C] |
| - Agenet | GO:0003723 | RNA binding | [M] |
| - AHS1 |  |  |  |  |
| - AHS2 |  |  |  |  |
| - AIP3 |  |  |  |  |
| - Ala\_racemase\_C | GO:0008784 | alanine racemase activity | [M] |
|  | GO:0006522 | alanine metabolic process | [B] |
| - AlcB |  |  |  |  |
| - Aldolase | GO:0003824 | catalytic activity | [M] |
|  | GO:0008152 | metabolic process | [B] |
| - Alginate\_lyase2 |  |  |  |  |
| - Alliinase\_C | GO:0016846 | carbon-sulfur lyase activity | [M] |
| - ALO | GO:0003885 | D-arabinono-1,4-lactone oxidase activity | [M] |
|  | GO:0055114 | oxidation reduction | [B] |
|  | GO:0016020 | membrane | [C] |
| - Alpha-L-AF\_C | GO:0046556 | alpha-N-arabinofuranosidase activity | [M] |
|  | GO:0046373 | L-arabinose metabolic process | [B] |
| - ALS\_ss\_C |  |  |  |  |
| - Amastin |  |  |  |  |
| - Amidase\_2 | GO:0008745 | N-acetylmuramoyl-L-alanine amidase activity | [M] |
|  | GO:0009253 | peptidoglycan catabolic process | [B] |
| - Amidohydro\_3 |  |  |  |  |
| - Amidoligase\_2 |  |  |  |  |
| - ANATO | GO:0005576 | extracellular region | [C] |
| - Anemone\_cytotox | GO:0015267 | channel activity | [M] |
|  | GO:0006812 | cation transport | [B] |
|  | GO:0046931 | pore complex assembly | [B] |
|  | GO:0052331 | hemolysis of cells in other organism during symbiotic interaction | [B] |
|  | GO:0046930 | pore complex | [C] |
| - Angiomotin\_C |  |  |  |  |
| - Anp1 |  |  |  |  |
| - Anth\_synt\_I\_N | GO:0016833 | oxo-acid-lyase activity | [M] |
|  | GO:0009058 | biosynthetic process | [B] |
| - Antistasin | GO:0004867 | serine-type endopeptidase inhibitor activity | [M] |
| - ApbE | GO:0009228 | thiamin biosynthetic process | [B] |
| - ApoL | GO:0008289 | lipid binding | [M] |
|  | GO:0006869 | lipid transport | [B] |
|  | GO:0042157 | lipoprotein metabolic process | [B] |
|  | GO:0005576 | extracellular region | [C] |
| - Apolipoprotein | GO:0008289 | lipid binding | [M] |
|  | GO:0006869 | lipid transport | [B] |
|  | GO:0042157 | lipoprotein metabolic process | [B] |
|  | GO:0005576 | extracellular region | [C] |
| - ArabFuran-catal | GO:0046556 | alpha-N-arabinofuranosidase activity | [M] |
|  | GO:0031221 | arabinan metabolic process | [B] |
| - AraC\_E\_bind |  |  |  |  |
| - AraC\_N |  |  |  |  |
| - Arginosuc\_synth | GO:0004055 | argininosuccinate synthase activity | [M] |
|  | GO:0005524 | ATP binding | [M] |
|  | GO:0006526 | arginine biosynthetic process | [B] |
| - ARL2\_Bind\_BART |  |  |  |  |
| - Armet |  |  |  |  |
| - ArsB | GO:0015105 | arsenite transmembrane transporter activity | [M] |
|  | GO:0016021 | integral to membrane | [C] |
| - ArsC |  |  |  |  |
| - ART | GO:0003956 | NAD(P)+-protein-arginine ADP-ribosyltransferase activity | [M] |
|  | GO:0006471 | protein amino acid ADP-ribosylation | [B] |
| - Arylsulfotrans |  |  |  |  |
| - ASC | GO:0005272 | sodium channel activity | [M] |
|  | GO:0006814 | sodium ion transport | [B] |
|  | GO:0016020 | membrane | [C] |
| - AsmA |  |  |  |  |
| - AsnC\_trans\_reg | GO:0003700 | transcription factor activity | [M] |
|  | GO:0043565 | sequence-specific DNA binding | [M] |
|  | GO:0006355 | regulation of transcription, DNA-dependent | [B] |
|  | GO:0005622 | intracellular | [C] |
| - Asp\_Arg\_Hydrox | GO:0004597 | peptide-aspartate beta-dioxygenase activity | [M] |
|  | GO:0018193 | peptidyl-amino acid modification | [B] |
|  | GO:0055114 | oxidation reduction | [B] |
|  | GO:0030176 | integral to endoplasmic reticulum membrane | [C] |
| - Asp\_Glu\_race |  |  |  |  |
| - Asparaginase\_II |  |  |  |  |
| - Astacin | GO:0004222 | metalloendopeptidase activity | [M] |
|  | GO:0006508 | proteolysis | [B] |
| - AstE\_AspA | GO:0016788 | hydrolase activity, acting on ester bonds | [M] |
|  | GO:0008152 | metabolic process | [B] |
| - ATG11 |  |  |  |  |
| - ATP-grasp\_3 |  |  |  |  |
| - ATP-gua\_Ptrans | GO:0016301 | kinase activity | [M] |
|  | GO:0016772 | transferase activity, transferring phosphorus-containing groups | [M] |
| - ATP-gua\_PtransN | GO:0016301 | kinase activity | [M] |
|  | GO:0016772 | transferase activity, transferring phosphorus-containing groups | [M] |
| - ATP-synt\_B | GO:0015078 | hydrogen ion transmembrane transporter activity | [M] |
|  | GO:0015986 | ATP synthesis coupled proton transport | [B] |
|  | GO:0045263 | proton-transporting ATP synthase complex, coupling factor F(o) | [C] |
| - ATP-synt\_DE | GO:0046933 | hydrogen ion transporting ATP synthase activity, rotational mechanism | [M] |
|  | GO:0046961 | proton-transporting ATPase activity, rotational mechanism | [M] |
|  | GO:0015986 | ATP synthesis coupled proton transport | [B] |
|  | GO:0045261 | proton-transporting ATP synthase complex, catalytic core F(1) | [C] |
| - ATP-synt\_E | GO:0015078 | hydrogen ion transmembrane transporter activity | [M] |
|  | GO:0015986 | ATP synthesis coupled proton transport | [B] |
|  | GO:0000276 | mitochondrial proton-transporting ATP synthase complex, coupling factor F(o) | [C] |
| - ATP\_bind\_2 | GO:0005524 | ATP binding | [M] |
| - ATP\_Ca\_trans\_C |  |  |  |  |
| - ATP\_synt\_H | GO:0015078 | hydrogen ion transmembrane transporter activity | [M] |
|  | GO:0015991 | ATP hydrolysis coupled proton transport | [B] |
|  | GO:0033179 | proton-transporting V-type ATPase, V0 domain | [C] |
| - ATS3 |  |  |  |  |
| - Autotransporter |  |  |  |  |
| - Ax\_dynein\_light |  |  |  |  |
| - AXE1 |  |  |  |  |
| - B12D |  |  |  |  |
| - B9 |  |  |  |  |
| - Bac\_DNA\_binding | GO:0003677 | DNA binding | [M] |
| - Bac\_DnaA |  |  |  |  |
| - Bac\_export\_1 | GO:0006605 | protein targeting | [B] |
|  | GO:0016020 | membrane | [C] |
| - Bac\_export\_2 | GO:0009306 | protein secretion | [B] |
|  | GO:0016020 | membrane | [C] |
| - Bac\_luciferase |  |  |  |  |
| - Bac\_rhamnosid |  |  |  |  |
| - Bac\_rhamnosid\_N |  |  |  |  |
| - Bac\_rhodopsin | GO:0005216 | ion channel activity | [M] |
|  | GO:0006811 | ion transport | [B] |
|  | GO:0016020 | membrane | [C] |
| - Bac\_transf |  |  |  |  |
| - Bac\_Ubq\_Cox | GO:0016491 | oxidoreductase activity | [M] |
|  | GO:0055114 | oxidation reduction | [B] |
|  | GO:0016020 | membrane | [C] |
| - BAF | GO:0003677 | DNA binding | [M] |
| - BAG | GO:0005515 | protein binding | [M] |
|  | GO:0006915 | apoptosis | [B] |
| - Band\_3\_cyto | GO:0008509 | anion transmembrane transporter activity | [M] |
|  | GO:0006820 | anion transport | [B] |
|  | GO:0016021 | integral to membrane | [C] |
| - Barwin | GO:0042742 | defense response to bacterium | [B] |
|  | GO:0050832 | defense response to fungus | [B] |
| - Baseplate\_J |  |  |  |  |
| - BAT2\_N |  |  |  |  |
| - BATS | GO:0004076 | biotin synthase activity | [M] |
|  | GO:0051536 | iron-sulfur cluster binding | [M] |
|  | GO:0009102 | biotin biosynthetic process | [B] |
| - BCA\_ABC\_TP\_C |  |  |  |  |
| - BCCT | GO:0005215 | transporter activity | [M] |
|  | GO:0006810 | transport | [B] |
|  | GO:0016020 | membrane | [C] |
| - BCDHK\_Adom3 |  |  |  |  |
| - Bestrophin |  |  |  |  |
| - Bgal\_small\_N | GO:0004565 | beta-galactosidase activity | [M] |
|  | GO:0005975 | carbohydrate metabolic process | [B] |
|  | GO:0009341 | beta-galactosidase complex | [C] |
| - BicD | GO:0005515 | protein binding | [M] |
|  | GO:0006810 | transport | [B] |
|  | GO:0005794 | Golgi apparatus | [C] |
| - BIR | GO:0005622 | intracellular | [C] |
| - BLUF |  |  |  |  |
| - BON |  |  |  |  |
| - Borealin |  |  |  |  |
| - Borrelia\_P83 |  |  |  |  |
| - Bot1p |  |  |  |  |
| - BPD\_transp\_1 | GO:0005215 | transporter activity | [M] |
|  | GO:0006810 | transport | [B] |
|  | GO:0016020 | membrane | [C] |
| - BPD\_transp\_2 | GO:0005215 | transporter activity | [M] |
|  | GO:0006810 | transport | [B] |
|  | GO:0016020 | membrane | [C] |
| - BRCA2 |  |  |  |  |
| - BRE |  |  |  |  |
| - BRE1 |  |  |  |  |
| - Bre5 |  |  |  |  |
| - BRK |  |  |  |  |
| - BrkDBD |  |  |  |  |
| - BSP |  |  |  |  |
| - BT1 |  |  |  |  |
| - BTAD |  |  |  |  |
| - BTK | GO:0007242 | intracellular signaling cascade | [B] |
| - Btz |  |  |  |  |
| - BUD22 |  |  |  |  |
| - Bug | GO:0030288 | outer membrane-bounded periplasmic space | [C] |
| - Bvg\_acc\_factor | GO:0016563 | transcription activator activity | [M] |
|  | GO:0045941 | positive regulation of transcription | [B] |
| - C1\_3 |  |  |  |  |
| - C6\_DPF |  |  |  |  |
| - Cache\_1 | GO:0016020 | membrane | [C] |
| - Cadherin | GO:0005509 | calcium ion binding | [M] |
|  | GO:0007156 | homophilic cell adhesion | [B] |
|  | GO:0016020 | membrane | [C] |
| - CagX |  |  |  |  |
| - Caleosin |  |  |  |  |
| - Calsequestrin | GO:0005509 | calcium ion binding | [M] |
| - Calx-beta | GO:0007154 | cell communication | [B] |
|  | GO:0016021 | integral to membrane | [C] |
| - CaMKII\_AD | GO:0004683 | calmodulin-dependent protein kinase activity | [M] |
|  | GO:0005516 | calmodulin binding | [M] |
|  | GO:0006468 | protein amino acid phosphorylation | [B] |
| - CAMSAP\_CH |  |  |  |  |
| - CAMSAP\_CKK |  |  |  |  |
| - CAP59\_mtransfer |  |  |  |  |
| - Caps\_synth |  |  |  |  |
| - Capsule\_synth | GO:0000271 | polysaccharide biosynthetic process | [B] |
|  | GO:0015774 | polysaccharide transport | [B] |
| - Carb\_anhydrase |  |  |  |  |
| - CarD\_TRCF | GO:0003700 | transcription factor activity | [M] |
|  | GO:0006355 | regulation of transcription, DNA-dependent | [B] |
| - Carn\_acyltransf | GO:0008415 | acyltransferase activity | [M] |
| - Cas1p |  |  |  |  |
| - Cas\_Cas4 |  |  |  |  |
| - Cast |  |  |  |  |
| - CAT | GO:0008811 | chloramphenicol O-acetyltransferase activity | [M] |
| - CathepsinC\_exc |  |  |  |  |
| - CBAH |  |  |  |  |
| - CbiC | GO:0016993 | precorrin-8X methylmutase activity | [M] |
|  | GO:0009236 | cobalamin biosynthetic process | [B] |
| - CBM\_1 | GO:0004553 | hydrolase activity, hydrolyzing O-glycosyl compounds | [M] |
|  | GO:0030248 | cellulose binding | [M] |
|  | GO:0005975 | carbohydrate metabolic process | [B] |
|  | GO:0005576 | extracellular region | [C] |
| - CBM\_2 | GO:0004553 | hydrolase activity, hydrolyzing O-glycosyl compounds | [M] |
|  | GO:0030246 | carbohydrate binding | [M] |
|  | GO:0005975 | carbohydrate metabolic process | [B] |
| - CBM\_21 |  |  |  |  |
| - CBM\_5\_12 | GO:0004553 | hydrolase activity, hydrolyzing O-glycosyl compounds | [M] |
|  | GO:0030246 | carbohydrate binding | [M] |
|  | GO:0005975 | carbohydrate metabolic process | [B] |
|  | GO:0005576 | extracellular region | [C] |
| - CBM\_6 | GO:0030246 | carbohydrate binding | [M] |
| - CD225 | GO:0009607 | response to biotic stimulus | [B] |
|  | GO:0016021 | integral to membrane | [C] |
| - CDC14 |  |  |  |  |
| - CDC37\_N |  |  |  |  |
| - Cdc6\_C |  |  |  |  |
| - CDI | GO:0004861 | cyclin-dependent protein kinase inhibitor activity | [M] |
|  | GO:0007050 | cell cycle arrest | [B] |
|  | GO:0005634 | nucleus | [C] |
| - CDT1 |  |  |  |  |
| - Cenp-B\_dimeris | GO:0003677 | DNA binding | [M] |
|  | GO:0003682 | chromatin binding | [M] |
|  | GO:0045449 | regulation of transcription | [B] |
|  | GO:0000775 | chromosome, centromeric region | [C] |
|  | GO:0005634 | nucleus | [C] |
| - CENP-B\_N | GO:0003677 | DNA binding | [M] |
|  | GO:0000775 | chromosome, centromeric region | [C] |
| - CG-1 | GO:0005516 | calmodulin binding | [M] |
|  | GO:0030528 | transcription regulator activity | [M] |
|  | GO:0045449 | regulation of transcription | [B] |
|  | GO:0005634 | nucleus | [C] |
| - CHAP |  |  |  |  |
| - CHD5 |  |  |  |  |
| - CHDCT2 | GO:0003677 | DNA binding | [M] |
|  | GO:0005524 | ATP binding | [M] |
|  | GO:0008270 | zinc ion binding | [M] |
|  | GO:0016818 | hydrolase activity, acting on acid anhydrides, in phosphorus-containing anhydrides | [M] |
|  | GO:0045449 | regulation of transcription | [B] |
|  | GO:0005634 | nucleus | [C] |
| - CheR | GO:0008757 | S-adenosylmethionine-dependent methyltransferase activity | [M] |
| - CheR\_N | GO:0008757 | S-adenosylmethionine-dependent methyltransferase activity | [M] |
| - CheW | GO:0004871 | signal transducer activity | [M] |
|  | GO:0006935 | chemotaxis | [B] |
|  | GO:0007165 | signal transduction | [B] |
|  | GO:0005622 | intracellular | [C] |
| - CHGN | GO:0016758 | transferase activity, transferring hexosyl groups | [M] |
|  | GO:0032580 | Golgi cisterna membrane | [C] |
| - Chitin\_bind\_1 | GO:0008061 | chitin binding | [M] |
| - Chitin\_synth\_1 | GO:0004100 | chitin synthase activity | [M] |
|  | GO:0006031 | chitin biosynthetic process | [B] |
| - Chitin\_synth\_1N |  |  |  |  |
| - Chlorophyllase | GO:0047746 | chlorophyllase activity | [M] |
|  | GO:0015996 | chlorophyll catabolic process | [B] |
| - Choline\_kin\_N |  |  |  |  |
| - Chorismate\_bind |  |  |  |  |
| - Chorismate\_synt | GO:0004107 | chorismate synthase activity | [M] |
|  | GO:0009073 | aromatic amino acid family biosynthetic process | [B] |
| - CHRD |  |  |  |  |
| - Chromate\_transp | GO:0015109 | chromate transmembrane transporter activity | [M] |
|  | GO:0015703 | chromate transport | [B] |
| - CHZ |  |  |  |  |
| - Cir\_Bir\_Yir |  |  |  |  |
| - CK1gamma\_C |  |  |  |  |
| - CLP\_protease | GO:0004252 | serine-type endopeptidase activity | [M] |
|  | GO:0006508 | proteolysis | [B] |
| - ClpS | GO:0030163 | protein catabolic process | [B] |
| - Cluap1 |  |  |  |  |
| - Clusterin | GO:0008219 | cell death | [B] |
| - CM\_2 | GO:0046417 | chorismate metabolic process | [B] |
| - CmcH\_NodU | GO:0003824 | catalytic activity | [M] |
|  | GO:0009058 | biosynthetic process | [B] |
| - CmcI | GO:0008168 | methyltransferase activity | [M] |
|  | GO:0008610 | lipid biosynthetic process | [B] |
| - CMD |  |  |  |  |
| - CobN-Mg\_chel | GO:0009058 | biosynthetic process | [B] |
| - Coiled-coil\_56 |  |  |  |  |
| - Competence\_A |  |  |  |  |
| - Condensation |  |  |  |  |
| - Cons\_hypoth698 | GO:0016021 | integral to membrane | [C] |
| - Cons\_hypoth95 |  |  |  |  |
| - Copper-bind | GO:0005507 | copper ion binding | [M] |
|  | GO:0009055 | electron carrier activity | [M] |
| - Coprinus\_mating | GO:0003677 | DNA binding | [M] |
|  | GO:0045449 | regulation of transcription | [B] |
|  | GO:0005634 | nucleus | [C] |
| - CorC\_HlyC |  |  |  |  |
| - Cornifin |  |  |  |  |
| - CotH |  |  |  |  |
| - COX4 | GO:0004129 | cytochrome-c oxidase activity | [M] |
| - COX6A | GO:0004129 | cytochrome-c oxidase activity | [M] |
|  | GO:0005743 | mitochondrial inner membrane | [C] |
|  | GO:0005751 | mitochondrial respiratory chain complex IV | [C] |
| - COX7a | GO:0004129 | cytochrome-c oxidase activity | [M] |
|  | GO:0009055 | electron carrier activity | [M] |
|  | GO:0005746 | mitochondrial respiratory chain | [C] |
| - CPDase | GO:0004112 | cyclic-nucleotide phosphodiesterase activity | [M] |
| - CpeT |  |  |  |  |
| - CPW\_WPC |  |  |  |  |
| - CR6\_interact | GO:0007049 | cell cycle | [B] |
|  | GO:0005634 | nucleus | [C] |
| - CRAM\_rpt |  |  |  |  |
| - CRCB | GO:0016020 | membrane | [C] |
| - CreA |  |  |  |  |
| - Creatininase |  |  |  |  |
| - Cript |  |  |  |  |
| - Crp | GO:0003700 | transcription factor activity | [M] |
|  | GO:0006355 | regulation of transcription, DNA-dependent | [B] |
|  | GO:0005622 | intracellular | [C] |
| - CRS1\_YhbY | GO:0003723 | RNA binding | [M] |
| - CrtC | GO:0016491 | oxidoreductase activity | [M] |
|  | GO:0015979 | photosynthesis | [B] |
|  | GO:0015995 | chlorophyll biosynthetic process | [B] |
|  | GO:0016117 | carotenoid biosynthetic process | [B] |
|  | GO:0055114 | oxidation reduction | [B] |
| - Crystall |  |  |  |  |
| - CsbD |  |  |  |  |
| - CSD | GO:0003677 | DNA binding | [M] |
|  | GO:0006355 | regulation of transcription, DNA-dependent | [B] |
| - CstA | GO:0009267 | cellular response to starvation | [B] |
|  | GO:0016020 | membrane | [C] |
| - CTK3 |  |  |  |  |
| - CTP\_transf\_3 | GO:0009103 | lipopolysaccharide biosynthetic process | [B] |
| - Cu-binding\_MopE |  |  |  |  |
| - Cu-oxidase | GO:0016491 | oxidoreductase activity | [M] |
|  | GO:0055114 | oxidation reduction | [B] |
| - Cu-oxidase\_2 | GO:0005507 | copper ion binding | [M] |
|  | GO:0016491 | oxidoreductase activity | [M] |
|  | GO:0055114 | oxidation reduction | [B] |
| - Cu-oxidase\_3 | GO:0005507 | copper ion binding | [M] |
|  | GO:0016491 | oxidoreductase activity | [M] |
|  | GO:0055114 | oxidation reduction | [B] |
| - Cu2\_monoox\_C |  |  |  |  |
| - Cu2\_monooxygen | GO:0004497 | monooxygenase activity | [M] |
|  | GO:0005507 | copper ion binding | [M] |
| - Cu\_amine\_oxid | GO:0005507 | copper ion binding | [M] |
|  | GO:0008131 | amine oxidase activity | [M] |
|  | GO:0048038 | quinone binding | [M] |
|  | GO:0009308 | amine metabolic process | [B] |
|  | GO:0055114 | oxidation reduction | [B] |
| - Cu\_amine\_oxidN2 | GO:0005507 | copper ion binding | [M] |
|  | GO:0008131 | amine oxidase activity | [M] |
|  | GO:0048038 | quinone binding | [M] |
|  | GO:0009308 | amine metabolic process | [B] |
|  | GO:0055114 | oxidation reduction | [B] |
| - Cu\_amine\_oxidN3 | GO:0005507 | copper ion binding | [M] |
|  | GO:0008131 | amine oxidase activity | [M] |
|  | GO:0048038 | quinone binding | [M] |
|  | GO:0009308 | amine metabolic process | [B] |
|  | GO:0055114 | oxidation reduction | [B] |
| - Cu\_bind\_like | GO:0005507 | copper ion binding | [M] |
|  | GO:0009055 | electron carrier activity | [M] |
| - CUB |  |  |  |  |
| - Cupin\_1 | GO:0045735 | nutrient reservoir activity | [M] |
| - Cupin\_3 |  |  |  |  |
| - CutA1 | GO:0010038 | response to metal ion | [B] |
| - Cutinase | GO:0016787 | hydrolase activity | [M] |
|  | GO:0008152 | metabolic process | [B] |
| - CVNH |  |  |  |  |
| - cwf21 |  |  |  |  |
| - Cyanate\_lyase | GO:0008824 | cyanate hydratase activity | [M] |
|  | GO:0009439 | cyanate metabolic process | [B] |
| - Cyclase |  |  |  |  |
| - Cys\_rich\_FGFR | GO:0016020 | membrane | [C] |
| - Cyto\_ox\_2 | GO:0055114 | oxidation reduction | [B] |
|  | GO:0016020 | membrane | [C] |
| - Cytochrom\_C\_asm | GO:0006461 | protein complex assembly | [B] |
|  | GO:0008535 | respiratory chain complex IV assembly | [B] |
|  | GO:0016020 | membrane | [C] |
| - DAGK\_prokar | GO:0004143 | diacylglycerol kinase activity | [M] |
|  | GO:0008654 | phospholipid biosynthetic process | [B] |
|  | GO:0016020 | membrane | [C] |
| - DAHP\_synth\_1 | GO:0009058 | biosynthetic process | [B] |
| - DAHP\_synth\_2 | GO:0003849 | 3-deoxy-7-phosphoheptulonate synthase activity | [M] |
|  | GO:0009073 | aromatic amino acid family biosynthetic process | [B] |
| - Dala\_Dala\_lig\_C | GO:0008716 | D-alanine-D-alanine ligase activity | [M] |
|  | GO:0009252 | peptidoglycan biosynthetic process | [B] |
| - Dala\_Dala\_lig\_N | GO:0008716 | D-alanine-D-alanine ligase activity | [M] |
|  | GO:0009252 | peptidoglycan biosynthetic process | [B] |
|  | GO:0005618 | cell wall | [C] |
| - DALR\_2 | GO:0000166 | nucleotide binding | [M] |
|  | GO:0004817 | cysteine-tRNA ligase activity | [M] |
|  | GO:0005524 | ATP binding | [M] |
|  | GO:0006412 | translation | [B] |
|  | GO:0006423 | cysteinyl-tRNA aminoacylation | [B] |
|  | GO:0005737 | cytoplasm | [C] |
| - DapB\_C | GO:0008839 | dihydrodipicolinate reductase activity | [M] |
|  | GO:0009089 | lysine biosynthetic process via diaminopimelate | [B] |
|  | GO:0055114 | oxidation reduction | [B] |
| - DapB\_N | GO:0008839 | dihydrodipicolinate reductase activity | [M] |
|  | GO:0009089 | lysine biosynthetic process via diaminopimelate | [B] |
|  | GO:0055114 | oxidation reduction | [B] |
| - DASH\_Ask1 |  |  |  |  |
| - DASH\_Dad1 |  |  |  |  |
| - DASH\_Dad2 |  |  |  |  |
| - DASH\_Dad4 |  |  |  |  |
| - DASH\_Dam1 |  |  |  |  |
| - Daxx |  |  |  |  |
| - DbpA |  |  |  |  |
| - DctM |  |  |  |  |
| - DegT\_DnrJ\_EryC1 |  |  |  |  |
| - Dehydrin | GO:0006950 | response to stress | [B] |
|  | GO:0009415 | response to water | [B] |
| - DEK\_C |  |  |  |  |
| - Destabilase | GO:0003796 | lysozyme activity | [M] |
| - Desulfoferrodox | GO:0005506 | iron ion binding | [M] |
|  | GO:0016491 | oxidoreductase activity | [M] |
|  | GO:0055114 | oxidation reduction | [B] |
| - DHBP\_synthase | GO:0008686 | 3,4-dihydroxy-2-butanone-4-phosphate synthase activity | [M] |
|  | GO:0009231 | riboflavin biosynthetic process | [B] |
| - DHDPS | GO:0016829 | lyase activity | [M] |
|  | GO:0008152 | metabolic process | [B] |
| - DHHA2 | GO:0016462 | pyrophosphatase activity | [M] |
|  | GO:0005737 | cytoplasm | [C] |
| - DHQ\_synthase | GO:0003856 | 3-dehydroquinate synthase activity | [M] |
|  | GO:0009073 | aromatic amino acid family biosynthetic process | [B] |
| - DHquinase\_I | GO:0003855 | 3-dehydroquinate dehydratase activity | [M] |
| - Dimerisation | GO:0008168 | methyltransferase activity | [M] |
|  | GO:0046983 | protein dimerization activity | [M] |
| - DinB |  |  |  |  |
| - Dioxygenase\_C | GO:0003824 | catalytic activity | [M] |
|  | GO:0008199 | ferric iron binding | [M] |
|  | GO:0006725 | cellular aromatic compound metabolic process | [B] |
|  | GO:0055114 | oxidation reduction | [B] |
| - Disintegrin |  |  |  |  |
| - DIT1\_PvcA |  |  |  |  |
| - DivIC | GO:0007049 | cell cycle | [B] |
| - DIX | GO:0004871 | signal transducer activity | [M] |
|  | GO:0007275 | multicellular organismal development | [B] |
|  | GO:0005622 | intracellular | [C] |
| - DM13 |  |  |  |  |
| - DMAP1 | GO:0016481 | negative regulation of transcription | [B] |
|  | GO:0005634 | nucleus | [C] |
| - DMRL\_synthase | GO:0009231 | riboflavin biosynthetic process | [B] |
|  | GO:0009349 | riboflavin synthase complex | [C] |
| - DNA\_gyraseA\_C | GO:0003677 | DNA binding | [M] |
|  | GO:0003916 | DNA topoisomerase activity | [M] |
|  | GO:0005524 | ATP binding | [M] |
|  | GO:0006265 | DNA topological change | [B] |
|  | GO:0005694 | chromosome | [C] |
| - DNA\_gyraseB\_C | GO:0003677 | DNA binding | [M] |
|  | GO:0003918 | DNA topoisomerase (ATP-hydrolyzing) activity | [M] |
|  | GO:0005524 | ATP binding | [M] |
|  | GO:0006265 | DNA topological change | [B] |
|  | GO:0005694 | chromosome | [C] |
| - DNA\_ligase\_ZBD | GO:0003911 | DNA ligase (NAD+) activity | [M] |
|  | GO:0006260 | DNA replication | [B] |
|  | GO:0006281 | DNA repair | [B] |
| - DNA\_photolyase | GO:0003913 | DNA photolyase activity | [M] |
|  | GO:0006281 | DNA repair | [B] |
| - DNA\_pol3\_alpha | GO:0008408 | 3'-5' exonuclease activity | [M] |
|  | GO:0006260 | DNA replication | [B] |
|  | GO:0005737 | cytoplasm | [C] |
| - DNA\_pol3\_beta | GO:0003677 | DNA binding | [M] |
|  | GO:0003887 | DNA-directed DNA polymerase activity | [M] |
|  | GO:0008408 | 3'-5' exonuclease activity | [M] |
|  | GO:0006260 | DNA replication | [B] |
| - DNA\_pol3\_gamma3 |  |  |  |  |
| - DNA\_pol\_alpha\_N |  |  |  |  |
| - DNA\_pol\_delta\_4 | GO:0006260 | DNA replication | [B] |
|  | GO:0005634 | nucleus | [C] |
| - DnaB | GO:0003678 | DNA helicase activity | [M] |
|  | GO:0005524 | ATP binding | [M] |
|  | GO:0006260 | DNA replication | [B] |
| - DNMT1-RFD |  |  |  |  |
| - DOPA\_dioxygen |  |  |  |  |
| - DoxX |  |  |  |  |
| - Dpy-30 |  |  |  |  |
| - Dpy19 |  |  |  |  |
| - Drc1-Sld2 |  |  |  |  |
| - DRTGG |  |  |  |  |
| - DRY\_EERY |  |  |  |  |
| - DsbD | GO:0017004 | cytochrome complex assembly | [B] |
|  | GO:0055114 | oxidation reduction | [B] |
|  | GO:0016020 | membrane | [C] |
| - DSL | GO:0007154 | cell communication | [B] |
|  | GO:0016020 | membrane | [C] |
| - dsRNA\_bind |  |  |  |  |
| - dTDP\_sugar\_isom | GO:0008830 | dTDP-4-dehydrorhamnose 3,5-epimerase activity | [M] |
|  | GO:0009103 | lipopolysaccharide biosynthetic process | [B] |
| - DTHCT | GO:0003677 | DNA binding | [M] |
|  | GO:0003918 | DNA topoisomerase (ATP-hydrolyzing) activity | [M] |
|  | GO:0005524 | ATP binding | [M] |
|  | GO:0005634 | nucleus | [C] |
| - DUF1008 |  |  |  |  |
| - DUF1016 |  |  |  |  |
| - DUF1022 |  |  |  |  |
| - DUF1023 |  |  |  |  |
| - DUF1034 | GO:0004252 | serine-type endopeptidase activity | [M] |
|  | GO:0005618 | cell wall | [C] |
|  | GO:0016020 | membrane | [C] |
| - DUF1042 |  |  |  |  |
| - DUF1078 | GO:0019861 | flagellum | [C] |
| - DUF108 | GO:0016491 | oxidoreductase activity | [M] |
|  | GO:0006742 | NADP catabolic process | [B] |
|  | GO:0019363 | pyridine nucleotide biosynthetic process | [B] |
|  | GO:0055114 | oxidation reduction | [B] |
| - DUF1080 |  |  |  |  |
| - DUF1083 | GO:0004553 | hydrolase activity, hydrolyzing O-glycosyl compounds | [M] |
|  | GO:0030246 | carbohydrate binding | [M] |
|  | GO:0016052 | carbohydrate catabolic process | [B] |
| - DUF1086 |  |  |  |  |
| - DUF1087 |  |  |  |  |
| - DUF1126 |  |  |  |  |
| - DUF1143 | GO:0005634 | nucleus | [C] |
| - DUF1173 |  |  |  |  |
| - DUF1181 |  |  |  |  |
| - DUF1191 |  |  |  |  |
| - DUF1193 |  |  |  |  |
| - DUF1208 |  |  |  |  |
| - DUF1214 |  |  |  |  |
| - DUF1223 |  |  |  |  |
| - DUF1232 |  |  |  |  |
| - DUF1234 |  |  |  |  |
| - DUF1237 |  |  |  |  |
| - DUF1254 |  |  |  |  |
| - DUF1255 |  |  |  |  |
| - DUF1258 |  |  |  |  |
| - DUF126 |  |  |  |  |
| - DUF1264 |  |  |  |  |
| - DUF1275 |  |  |  |  |
| - DUF1296 |  |  |  |  |
| - DUF1298 |  |  |  |  |
| - DUF1301 |  |  |  |  |
| - DUF1304 |  |  |  |  |
| - DUF1308 |  |  |  |  |
| - DUF1309 |  |  |  |  |
| - DUF1330 |  |  |  |  |
| - DUF1336 |  |  |  |  |
| - DUF1338 |  |  |  |  |
| - DUF1343 |  |  |  |  |
| - DUF1349 |  |  |  |  |
| - DUF1352 |  |  |  |  |
| - DUF1356 |  |  |  |  |
| - DUF1358 |  |  |  |  |
| - DUF1395 |  |  |  |  |
| - DUF1399 |  |  |  |  |
| - DUF140 |  |  |  |  |
| - DUF1445 |  |  |  |  |
| - DUF1448 |  |  |  |  |
| - DUF1468 |  |  |  |  |
| - DUF148 |  |  |  |  |
| - DUF1485 |  |  |  |  |
| - DUF149 |  |  |  |  |
| - DUF150 |  |  |  |  |
| - DUF1517 |  |  |  |  |
| - DUF1524 |  |  |  |  |
| - DUF1537 |  |  |  |  |
| - DUF159 |  |  |  |  |
| - DUF1619 |  |  |  |  |
| - DUF1625 |  |  |  |  |
| - DUF1640 |  |  |  |  |
| - DUF1647 |  |  |  |  |
| - DUF1680 |  |  |  |  |
| - DUF1688 |  |  |  |  |
| - DUF1704 |  |  |  |  |
| - DUF1725 |  |  |  |  |
| - DUF1729 |  |  |  |  |
| - DUF1742 |  |  |  |  |
| - DUF1752 |  |  |  |  |
| - DUF1757 |  |  |  |  |
| - DUF1759 |  |  |  |  |
| - DUF1762 |  |  |  |  |
| - DUF1769 |  |  |  |  |
| - DUF1783 |  |  |  |  |
| - DUF1785 |  |  |  |  |
| - DUF1814 |  |  |  |  |
| - DUF1838 |  |  |  |  |
| - DUF1855 |  |  |  |  |
| - DUF19 |  |  |  |  |
| - DUF1903 |  |  |  |  |
| - DUF1917 |  |  |  |  |
| - DUF1929 |  |  |  |  |
| - DUF1949 |  |  |  |  |
| - DUF1966 | GO:0004556 | alpha-amylase activity | [M] |
|  | GO:0005509 | calcium ion binding | [M] |
|  | GO:0016052 | carbohydrate catabolic process | [B] |
| - DUF1982 | GO:0016651 | oxidoreductase activity, acting on NADH or NADPH | [M] |
|  | GO:0051536 | iron-sulfur cluster binding | [M] |
|  | GO:0055114 | oxidation reduction | [B] |
| - DUF1989 |  |  |  |  |
| - DUF1992 |  |  |  |  |
| - DUF1996 |  |  |  |  |
| - DUF1998 |  |  |  |  |
| - DUF2045 |  |  |  |  |
| - DUF2048 |  |  |  |  |
| - DUF205 | GO:0005886 | plasma membrane | [C] |
| - DUF2051 |  |  |  |  |
| - DUF2052 |  |  |  |  |
| - DUF2064 |  |  |  |  |
| - DUF2075 |  |  |  |  |
| - DUF208 |  |  |  |  |
| - DUF2103 |  |  |  |  |
| - DUF2156 |  |  |  |  |
| - DUF2169 |  |  |  |  |
| - DUF2181 |  |  |  |  |
| - DUF2183 |  |  |  |  |
| - DUF2196 |  |  |  |  |
| - DUF2215 |  |  |  |  |
| - DUF2228 |  |  |  |  |
| - DUF2233 |  |  |  |  |
| - DUF2235 |  |  |  |  |
| - DUF2236 |  |  |  |  |
| - DUF2237 |  |  |  |  |
| - DUF2252 |  |  |  |  |
| - DUF2257 |  |  |  |  |
| - DUF2260 |  |  |  |  |
| - DUF2263 |  |  |  |  |
| - DUF2264 |  |  |  |  |
| - DUF23 |  |  |  |  |
| - DUF2306 |  |  |  |  |
| - DUF231 |  |  |  |  |
| - DUF2346 |  |  |  |  |
| - DUF2355 |  |  |  |  |
| - DUF2358 |  |  |  |  |
| - DUF2359 |  |  |  |  |
| - DUF2361 |  |  |  |  |
| - DUF2368 |  |  |  |  |
| - DUF239 |  |  |  |  |
| - DUF2401 |  |  |  |  |
| - DUF2403 |  |  |  |  |
| - DUF2407 |  |  |  |  |
| - DUF2408 |  |  |  |  |
| - DUF2414 |  |  |  |  |
| - DUF2415 |  |  |  |  |
| - DUF2423 |  |  |  |  |
| - DUF2424 |  |  |  |  |
| - DUF2452 |  |  |  |  |
| - DUF2453 |  |  |  |  |
| - DUF2454 |  |  |  |  |
| - DUF2464 |  |  |  |  |
| - DUF2466 | GO:0006281 | DNA repair | [B] |
| - DUF2470 |  |  |  |  |
| - DUF2475 |  |  |  |  |
| - DUF2561 |  |  |  |  |
| - DUF2615 |  |  |  |  |
| - DUF262 |  |  |  |  |
| - DUF265 | GO:0005524 | ATP binding | [M] |
|  | GO:0016740 | transferase activity | [M] |
| - DUF268 |  |  |  |  |
| - DUF273 |  |  |  |  |
| - DUF2738 |  |  |  |  |
| - DUF2780 |  |  |  |  |
| - DUF2781 |  |  |  |  |
| - DUF2786 |  |  |  |  |
| - DUF28 |  |  |  |  |
| - DUF2807 |  |  |  |  |
| - DUF2843 |  |  |  |  |
| - DUF2870 |  |  |  |  |
| - DUF288 |  |  |  |  |
| - DUF2945 |  |  |  |  |
| - DUF296 |  |  |  |  |
| - DUF2961 |  |  |  |  |
| - DUF297 |  |  |  |  |
| - DUF2985 |  |  |  |  |
| - DUF299 | GO:0005524 | ATP binding | [M] |
|  | GO:0016772 | transferase activity, transferring phosphorus-containing groups | [M] |
| - DUF3011 |  |  |  |  |
| - DUF302 |  |  |  |  |
| - DUF303 |  |  |  |  |
| - DUF304 |  |  |  |  |
| - DUF3133 |  |  |  |  |
| - DUF3140 |  |  |  |  |
| - DUF3161 |  |  |  |  |
| - DUF3168 |  |  |  |  |
| - DUF3228 |  |  |  |  |
| - DUF3237 |  |  |  |  |
| - DUF3245 |  |  |  |  |
| - DUF3250 |  |  |  |  |
| - DUF3253 |  |  |  |  |
| - DUF328 |  |  |  |  |
| - DUF3294 |  |  |  |  |
| - DUF330 |  |  |  |  |
| - DUF3312 |  |  |  |  |
| - DUF3317 |  |  |  |  |
| - DUF3321 |  |  |  |  |
| - DUF3336 |  |  |  |  |
| - DUF3342 |  |  |  |  |
| - DUF336 |  |  |  |  |
| - DUF3362 |  |  |  |  |
| - DUF3419 |  |  |  |  |
| - DUF3421 |  |  |  |  |
| - DUF3429 |  |  |  |  |
| - DUF3431 |  |  |  |  |
| - DUF3445 |  |  |  |  |
| - DUF3451 |  |  |  |  |
| - DUF3455 |  |  |  |  |
| - DUF3456 |  |  |  |  |
| - DUF3458 |  |  |  |  |
| - DUF3469 |  |  |  |  |
| - DUF3472 |  |  |  |  |
| - DUF3474 |  |  |  |  |
| - DUF3494 |  |  |  |  |
| - DUF3500 |  |  |  |  |
| - DUF3506 |  |  |  |  |
| - DUF3508 |  |  |  |  |
| - DUF3523 |  |  |  |  |
| - DUF3552 |  |  |  |  |
| - DUF3583 |  |  |  |  |
| - DUF3586 |  |  |  |  |
| - DUF3598 |  |  |  |  |
| - DUF3635 |  |  |  |  |
| - DUF3638 |  |  |  |  |
| - DUF3639 |  |  |  |  |
| - DUF3641 |  |  |  |  |
| - DUF3645 |  |  |  |  |
| - DUF3661 |  |  |  |  |
| - DUF3669 |  |  |  |  |
| - DUF3684 |  |  |  |  |
| - DUF37 |  |  |  |  |
| - DUF3704 |  |  |  |  |
| - DUF3712 |  |  |  |  |
| - DUF3743 |  |  |  |  |
| - DUF3754 |  |  |  |  |
| - DUF3758 |  |  |  |  |
| - DUF389 |  |  |  |  |
| - DUF399 |  |  |  |  |
| - DUF411 |  |  |  |  |
| - DUF427 |  |  |  |  |
| - DUF445 |  |  |  |  |
| - DUF448 |  |  |  |  |
| - DUF455 |  |  |  |  |
| - DUF461 |  |  |  |  |
| - DUF465 |  |  |  |  |
| - DUF477 |  |  |  |  |
| - DUF484 |  |  |  |  |
| - DUF490 |  |  |  |  |
| - DUF500 |  |  |  |  |
| - DUF520 |  |  |  |  |
| - DUF521 |  |  |  |  |
| - DUF540 | GO:0019344 | cysteine biosynthetic process | [B] |
|  | GO:0009276 | Gram-negative-bacterium-type cell wall | [C] |
|  | GO:0016021 | integral to membrane | [C] |
| - DUF55 |  |  |  |  |
| - DUF563 | GO:0016757 | transferase activity, transferring glycosyl groups | [M] |
| - DUF566 |  |  |  |  |
| - DUF579 |  |  |  |  |
| - DUF581 |  |  |  |  |
| - DUF582 |  |  |  |  |
| - DUF583 |  |  |  |  |
| - DUF604 |  |  |  |  |
| - DUF606 |  |  |  |  |
| - DUF640 |  |  |  |  |
| - DUF641 |  |  |  |  |
| - DUF659 |  |  |  |  |
| - DUF662 |  |  |  |  |
| - DUF667 |  |  |  |  |
| - DUF672 |  |  |  |  |
| - DUF692 |  |  |  |  |
| - DUF702 |  |  |  |  |
| - DUF707 |  |  |  |  |
| - DUF711 |  |  |  |  |
| - DUF716 |  |  |  |  |
| - DUF72 |  |  |  |  |
| - DUF724 |  |  |  |  |
| - DUF729 |  |  |  |  |
| - DUF74 |  |  |  |  |
| - DUF745 |  |  |  |  |
| - DUF748 |  |  |  |  |
| - DUF77 |  |  |  |  |
| - DUF772 |  |  |  |  |
| - DUF803 |  |  |  |  |
| - DUF815 |  |  |  |  |
| - DUF818 |  |  |  |  |
| - DUF819 |  |  |  |  |
| - DUF821 |  |  |  |  |
| - DUF827 |  |  |  |  |
| - DUF837 |  |  |  |  |
| - DUF842 |  |  |  |  |
| - DUF849 |  |  |  |  |
| - DUF853 |  |  |  |  |
| - DUF872 |  |  |  |  |
| - DUF89 |  |  |  |  |
| - DUF894 |  |  |  |  |
| - DUF908 |  |  |  |  |
| - DUF913 |  |  |  |  |
| - DUF917 |  |  |  |  |
| - DUF924 |  |  |  |  |
| - DUF938 |  |  |  |  |
| - DUF939 |  |  |  |  |
| - DUF946 |  |  |  |  |
| - DUF952 |  |  |  |  |
| - DXP\_redisom\_C | GO:0030604 | 1-deoxy-D-xylulose-5-phosphate reductoisomerase activity | [M] |
|  | GO:0046872 | metal ion binding | [M] |
|  | GO:0008299 | isoprenoid biosynthetic process | [B] |
|  | GO:0055114 | oxidation reduction | [B] |
| - DXP\_reductoisom | GO:0030604 | 1-deoxy-D-xylulose-5-phosphate reductoisomerase activity | [M] |
|  | GO:0046872 | metal ion binding | [M] |
|  | GO:0008299 | isoprenoid biosynthetic process | [B] |
|  | GO:0055114 | oxidation reduction | [B] |
| - EAL |  |  |  |  |
| - ECSIT |  |  |  |  |
| - Ectoine\_synth | GO:0016836 | hydro-lyase activity | [M] |
|  | GO:0006596 | polyamine biosynthetic process | [B] |
| - Eeig1 |  |  |  |  |
| - EFP | GO:0003746 | translation elongation factor activity | [M] |
|  | GO:0006414 | translational elongation | [B] |
| - EGF |  |  |  |  |
| - EGF\_alliinase | GO:0016846 | carbon-sulfur lyase activity | [M] |
| - EHN | GO:0004301 | epoxide hydrolase activity | [M] |
|  | GO:0009636 | response to toxin | [B] |
|  | GO:0016020 | membrane | [C] |
| - eIF-4B |  |  |  |  |
| - EIN3 | GO:0030528 | transcription regulator activity | [M] |
|  | GO:0005634 | nucleus | [C] |
| - ELH | GO:0005179 | hormone activity | [M] |
|  | GO:0007275 | multicellular organismal development | [B] |
|  | GO:0005576 | extracellular region | [C] |
| - ELM2 |  |  |  |  |
| - Elong-fact-P\_C |  |  |  |  |
| - EndIII\_4Fe-2S | GO:0004519 | endonuclease activity | [M] |
|  | GO:0051539 | 4 iron, 4 sulfur cluster binding | [M] |
| - Endonuclease\_1 | GO:0004518 | nuclease activity | [M] |
| - Endonuclease\_7 | GO:0004519 | endonuclease activity | [M] |
| - ENT |  |  |  |  |
| - Epimerase\_2 | GO:0008761 | UDP-N-acetylglucosamine 2-epimerase activity | [M] |
|  | GO:0006047 | UDP-N-acetylglucosamine metabolic process | [B] |
|  | GO:0009103 | lipopolysaccharide biosynthetic process | [B] |
| - EPSP\_synthase | GO:0016765 | transferase activity, transferring alkyl or aryl (other than methyl) groups | [M] |
| - ERp29\_N | GO:0009306 | protein secretion | [B] |
|  | GO:0005788 | endoplasmic reticulum lumen | [C] |
| - Erythro\_esteras | GO:0046677 | response to antibiotic | [B] |
| - EST1 |  |  |  |  |
| - EutB | GO:0008851 | ethanolamine ammonia-lyase activity | [M] |
|  | GO:0006520 | cellular amino acid metabolic process | [B] |
| - EutQ |  |  |  |  |
| - ExbD | GO:0005215 | transporter activity | [M] |
|  | GO:0006810 | transport | [B] |
|  | GO:0016020 | membrane | [C] |
| - Exonuc\_V\_gamma | GO:0008854 | exodeoxyribonuclease V activity | [M] |
|  | GO:0009338 | exodeoxyribonuclease V complex | [C] |
| - Exonuc\_VII\_L |  |  |  |  |
| - Exostosin | GO:0016020 | membrane | [C] |
| - Extensin\_2 | GO:0005199 | structural constituent of cell wall | [M] |
|  | GO:0009664 | plant-type cell wall organization | [B] |
| - F1F0-ATPsyn\_F |  |  |  |  |
| - FA\_desaturase\_2 | GO:0045300 | acyl-[acyl-carrier-protein] desaturase activity | [M] |
|  | GO:0006631 | fatty acid metabolic process | [B] |
|  | GO:0055114 | oxidation reduction | [B] |
| - FabA |  |  |  |  |
| - FAD\_binding\_7 | GO:0003913 | DNA photolyase activity | [M] |
|  | GO:0006281 | DNA repair | [B] |
| - FAD\_binding\_9 |  |  |  |  |
| - FAD\_syn | GO:0003919 | FMN adenylyltransferase activity | [M] |
|  | GO:0009231 | riboflavin biosynthetic process | [B] |
| - FANCF |  |  |  |  |
| - Fapy\_DNA\_glyco | GO:0003684 | damaged DNA binding | [M] |
|  | GO:0003906 | DNA-(apurinic or apyrimidinic site) lyase activity | [M] |
|  | GO:0008270 | zinc ion binding | [M] |
|  | GO:0016799 | hydrolase activity, hydrolyzing N-glycosyl compounds | [M] |
|  | GO:0006284 | base-excision repair | [B] |
| - Fasciclin |  |  |  |  |
| - FAST\_1 | GO:0004672 | protein kinase activity | [M] |
|  | GO:0005524 | ATP binding | [M] |
|  | GO:0006915 | apoptosis | [B] |
| - FBA | GO:0005515 | protein binding | [M] |
|  | GO:0030163 | protein catabolic process | [B] |
| - FCD |  |  |  |  |
| - FecCD | GO:0005215 | transporter activity | [M] |
|  | GO:0006810 | transport | [B] |
|  | GO:0016020 | membrane | [C] |
| - FecR |  |  |  |  |
| - FeoB\_N | GO:0005525 | GTP binding | [M] |
|  | GO:0015093 | ferrous iron transmembrane transporter activity | [M] |
|  | GO:0015684 | ferrous iron transport | [B] |
|  | GO:0016021 | integral to membrane | [C] |
| - FerI |  |  |  |  |
| - Ferritin | GO:0008199 | ferric iron binding | [M] |
|  | GO:0006879 | cellular iron ion homeostasis | [B] |
| - FeS | GO:0051536 | iron-sulfur cluster binding | [M] |
| - Fez1 | GO:0005737 | cytoplasm | [C] |
|  | GO:0016020 | membrane | [C] |
| - FGase |  |  |  |  |
| - FGF | GO:0008083 | growth factor activity | [M] |
| - FHIPEP | GO:0009306 | protein secretion | [B] |
|  | GO:0016020 | membrane | [C] |
| - Fibrinogen\_BP |  |  |  |  |
| - Fibrinogen\_C | GO:0005102 | receptor binding | [M] |
|  | GO:0007165 | signal transduction | [B] |
| - Filament |  |  |  |  |
| - Fimbrial |  |  |  |  |
| - FIST |  |  |  |  |
| - FIST\_C |  |  |  |  |
| - Flagellin\_C | GO:0005198 | structural molecule activity | [M] |
|  | GO:0001539 | ciliary or flagellar motility | [B] |
|  | GO:0009288 | bacterial-type flagellum | [C] |
| - Flagellin\_N | GO:0005198 | structural molecule activity | [M] |
|  | GO:0001539 | ciliary or flagellar motility | [B] |
|  | GO:0009420 | bacterial-type flagellum filament | [C] |
| - Flg\_bb\_rod | GO:0003774 | motor activity | [M] |
|  | GO:0005198 | structural molecule activity | [M] |
|  | GO:0001539 | ciliary or flagellar motility | [B] |
|  | GO:0009288 | bacterial-type flagellum | [C] |
| - FliE | GO:0003774 | motor activity | [M] |
|  | GO:0005198 | structural molecule activity | [M] |
|  | GO:0001539 | ciliary or flagellar motility | [B] |
|  | GO:0009288 | bacterial-type flagellum | [C] |
| - FliH |  |  |  |  |
| - FLYWCH |  |  |  |  |
| - FmdA\_AmdA | GO:0016811 | hydrolase activity, acting on carbon-nitrogen (but not peptide) bonds, in linear amides | [M] |
|  | GO:0008152 | metabolic process | [B] |
| - FMN\_bind\_2 |  |  |  |  |
| - Fmp27\_GFWDK |  |  |  |  |
| - fn2 |  |  |  |  |
| - fn3 |  |  |  |  |
| - Folate\_rec |  |  |  |  |
| - FOP\_dimer | GO:0034453 | microtubule anchoring | [B] |
|  | GO:0005813 | centrosome | [C] |
| - Fork\_head | GO:0003700 | transcription factor activity | [M] |
|  | GO:0043565 | sequence-specific DNA binding | [M] |
|  | GO:0006355 | regulation of transcription, DNA-dependent | [B] |
|  | GO:0005634 | nucleus | [C] |
| - Fork\_head\_N |  |  |  |  |
| - Formyl\_trans\_C | GO:0016742 | hydroxymethyl-, formyl- and related transferase activity | [M] |
|  | GO:0009058 | biosynthetic process | [B] |
| - FRG1 |  |  |  |  |
| - Fringe | GO:0016757 | transferase activity, transferring glycosyl groups | [M] |
|  | GO:0016020 | membrane | [C] |
| - Fructosamin\_kin |  |  |  |  |
| - FTH |  |  |  |  |
| - FTR1 | GO:0055085 | transmembrane transport | [B] |
|  | GO:0016020 | membrane | [C] |
| - FtsA | GO:0007049 | cell cycle | [B] |
| - Ftsk\_gamma |  |  |  |  |
| - FTSW\_RODA\_SPOVE | GO:0007049 | cell cycle | [B] |
|  | GO:0016021 | integral to membrane | [C] |
| - FUR | GO:0003700 | transcription factor activity | [M] |
|  | GO:0006355 | regulation of transcription, DNA-dependent | [B] |
| - FUSC |  |  |  |  |
| - FYRC | GO:0003677 | DNA binding | [M] |
|  | GO:0005634 | nucleus | [C] |
| - FYRN | GO:0003677 | DNA binding | [M] |
|  | GO:0005634 | nucleus | [C] |
| - FYTT |  |  |  |  |
| - G\_glu\_transpept | GO:0003840 | gamma-glutamyltransferase activity | [M] |
| - Gal-3-0\_sulfotr | GO:0001733 | galactosylceramide sulfotransferase activity | [M] |
|  | GO:0009058 | biosynthetic process | [B] |
|  | GO:0005794 | Golgi apparatus | [C] |
|  | GO:0016021 | integral to membrane | [C] |
| - Gal-bind\_lectin | GO:0005529 | sugar binding | [M] |
| - Gal\_Lectin | GO:0005529 | sugar binding | [M] |
| - Galactosyl\_T\_2 | GO:0016757 | transferase activity, transferring glycosyl groups | [M] |
|  | GO:0005975 | carbohydrate metabolic process | [B] |
| - GalP\_UDP\_tr\_C | GO:0008108 | UDP-glucose:hexose-1-phosphate uridylyltransferase activity | [M] |
|  | GO:0006012 | galactose metabolic process | [B] |
| - GalP\_UDP\_transf | GO:0008108 | UDP-glucose:hexose-1-phosphate uridylyltransferase activity | [M] |
|  | GO:0006012 | galactose metabolic process | [B] |
| - Gamma-thionin | GO:0006952 | defense response | [B] |
| - GASA |  |  |  |  |
| - GATA-N | GO:0003677 | DNA binding | [M] |
|  | GO:0008270 | zinc ion binding | [M] |
|  | GO:0016563 | transcription activator activity | [M] |
|  | GO:0045941 | positive regulation of transcription | [B] |
|  | GO:0005634 | nucleus | [C] |
| - GATase\_3 | GO:0003824 | catalytic activity | [M] |
|  | GO:0009236 | cobalamin biosynthetic process | [B] |
| - Gb3\_synth | GO:0008378 | galactosyltransferase activity | [M] |
|  | GO:0005795 | Golgi stack | [C] |
| - GCK |  |  |  |  |
| - GcpE | GO:0046429 | 4-hydroxy-3-methylbut-2-en-1-yl diphosphate synthase activity | [M] |
|  | GO:0016114 | terpenoid biosynthetic process | [B] |
|  | GO:0055114 | oxidation reduction | [B] |
| - GCR1\_C |  |  |  |  |
| - GD\_AH\_C | GO:0016836 | hydro-lyase activity | [M] |
| - GDA1\_CD39 | GO:0016787 | hydrolase activity | [M] |
| - Gemin6 | GO:0000245 | spliceosome assembly | [B] |
|  | GO:0005634 | nucleus | [C] |
| - Gemin7 |  |  |  |  |
| - GerE | GO:0003700 | transcription factor activity | [M] |
|  | GO:0043565 | sequence-specific DNA binding | [M] |
|  | GO:0006355 | regulation of transcription, DNA-dependent | [B] |
|  | GO:0005622 | intracellular | [C] |
| - GET2 |  |  |  |  |
| - GH3 |  |  |  |  |
| - GidB | GO:0008649 | rRNA methyltransferase activity | [M] |
|  | GO:0006364 | rRNA processing | [B] |
|  | GO:0005737 | cytoplasm | [C] |
| - GIDE |  |  |  |  |
| - GILT |  |  |  |  |
| - Git3 |  |  |  |  |
| - GlcNAc\_2-epim | GO:0004476 | mannose-6-phosphate isomerase activity | [M] |
|  | GO:0006013 | mannose metabolic process | [B] |
| - GLF | GO:0008767 | UDP-galactopyranose mutase activity | [M] |
| - Gln-synt\_N | GO:0004356 | glutamate-ammonia ligase activity | [M] |
|  | GO:0006542 | glutamine biosynthetic process | [B] |
|  | GO:0006807 | nitrogen compound metabolic process | [B] |
| - Glt\_symporter | GO:0015501 | glutamate:sodium symporter activity | [M] |
|  | GO:0015813 | L-glutamate transport | [B] |
|  | GO:0016021 | integral to membrane | [C] |
| - GLTP | GO:0017089 | glycolipid transporter activity | [M] |
|  | GO:0051861 | glycolipid binding | [M] |
|  | GO:0046836 | glycolipid transport | [B] |
|  | GO:0005737 | cytoplasm | [C] |
| - Glu\_cys\_ligase | GO:0004357 | glutamate-cysteine ligase activity | [M] |
|  | GO:0006750 | glutathione biosynthetic process | [B] |
| - Glu\_syn\_central | GO:0015930 | glutamate synthase activity | [M] |
|  | GO:0006807 | nitrogen compound metabolic process | [B] |
|  | GO:0055114 | oxidation reduction | [B] |
| - Glu\_synthase | GO:0015930 | glutamate synthase activity | [M] |
|  | GO:0006537 | glutamate biosynthetic process | [B] |
| - Glucan\_synthase | GO:0003843 | 1,3-beta-glucan synthase activity | [M] |
|  | GO:0006075 | 1,3-beta-glucan biosynthetic process | [B] |
|  | GO:0000148 | 1,3-beta-glucan synthase complex | [C] |
|  | GO:0016020 | membrane | [C] |
| - GlutR\_N | GO:0008883 | glutamyl-tRNA reductase activity | [M] |
|  | GO:0050661 | NADP or NADPH binding | [M] |
|  | GO:0033014 | tetrapyrrole biosynthetic process | [B] |
|  | GO:0055114 | oxidation reduction | [B] |
| - Gly\_transf\_sug |  |  |  |  |
| - Glyco\_hydro\_1 | GO:0004553 | hydrolase activity, hydrolyzing O-glycosyl compounds | [M] |
|  | GO:0005975 | carbohydrate metabolic process | [B] |
| - Glyco\_hydro\_10 | GO:0004553 | hydrolase activity, hydrolyzing O-glycosyl compounds | [M] |
|  | GO:0005975 | carbohydrate metabolic process | [B] |
| - Glyco\_hydro\_12 | GO:0008810 | cellulase activity | [M] |
|  | GO:0000272 | polysaccharide catabolic process | [B] |
| - Glyco\_hydro\_17 | GO:0004553 | hydrolase activity, hydrolyzing O-glycosyl compounds | [M] |
|  | GO:0005975 | carbohydrate metabolic process | [B] |
| - Glyco\_hydro\_19 | GO:0004568 | chitinase activity | [M] |
|  | GO:0006032 | chitin catabolic process | [B] |
|  | GO:0016998 | cell wall macromolecule catabolic process | [B] |
| - Glyco\_hydro\_28 | GO:0004650 | polygalacturonase activity | [M] |
|  | GO:0005975 | carbohydrate metabolic process | [B] |
| - Glyco\_hydro\_30 | GO:0004348 | glucosylceramidase activity | [M] |
|  | GO:0006665 | sphingolipid metabolic process | [B] |
|  | GO:0007040 | lysosome organization | [B] |
|  | GO:0005764 | lysosome | [C] |
| - Glyco\_hydro\_32C |  |  |  |  |
| - Glyco\_hydro\_32N |  |  |  |  |
| - Glyco\_hydro\_42M | GO:0004565 | beta-galactosidase activity | [M] |
|  | GO:0005975 | carbohydrate metabolic process | [B] |
| - Glyco\_hydro\_43 | GO:0004553 | hydrolase activity, hydrolyzing O-glycosyl compounds | [M] |
|  | GO:0005975 | carbohydrate metabolic process | [B] |
| - Glyco\_hydro\_45 | GO:0008810 | cellulase activity | [M] |
|  | GO:0005975 | carbohydrate metabolic process | [B] |
| - Glyco\_hydro\_46 | GO:0016977 | chitosanase activity | [M] |
|  | GO:0005975 | carbohydrate metabolic process | [B] |
|  | GO:0005576 | extracellular region | [C] |
| - Glyco\_hydro\_49 |  |  |  |  |
| - Glyco\_hydro\_53 | GO:0015926 | glucosidase activity | [M] |
| - Glyco\_hydro\_6 | GO:0004553 | hydrolase activity, hydrolyzing O-glycosyl compounds | [M] |
|  | GO:0030245 | cellulose catabolic process | [B] |
| - Glyco\_hydro\_61 |  |  |  |  |
| - Glyco\_hydro\_65C |  |  |  |  |
| - Glyco\_hydro\_65N | GO:0003824 | catalytic activity | [M] |
|  | GO:0030246 | carbohydrate binding | [M] |
|  | GO:0005975 | carbohydrate metabolic process | [B] |
| - Glyco\_hydro\_67C | GO:0046559 | alpha-glucuronidase activity | [M] |
|  | GO:0045493 | xylan catabolic process | [B] |
|  | GO:0005576 | extracellular region | [C] |
| - Glyco\_hydro\_67M | GO:0046559 | alpha-glucuronidase activity | [M] |
|  | GO:0045493 | xylan catabolic process | [B] |
|  | GO:0005576 | extracellular region | [C] |
| - Glyco\_hydro\_67N | GO:0046559 | alpha-glucuronidase activity | [M] |
|  | GO:0045493 | xylan catabolic process | [B] |
| - Glyco\_hydro\_68 | GO:0050053 | levansucrase activity | [M] |
|  | GO:0007587 | sugar utilization | [B] |
| - Glyco\_hydro\_71 |  |  |  |  |
| - Glyco\_hydro\_72 |  |  |  |  |
| - Glyco\_hydro\_76 |  |  |  |  |
| - Glyco\_hydro\_79n | GO:0016798 | hydrolase activity, acting on glycosyl bonds | [M] |
|  | GO:0016020 | membrane | [C] |
| - Glyco\_hydro\_8 | GO:0004553 | hydrolase activity, hydrolyzing O-glycosyl compounds | [M] |
|  | GO:0005975 | carbohydrate metabolic process | [B] |
| - Glyco\_hydro\_81 | GO:0033903 | endo-1,3(4)-beta-glucanase activity | [M] |
|  | GO:0016998 | cell wall macromolecule catabolic process | [B] |
| - Glyco\_hydro\_88 |  |  |  |  |
| - Glyco\_transf\_11 | GO:0008107 | galactoside 2-alpha-L-fucosyltransferase activity | [M] |
|  | GO:0005975 | carbohydrate metabolic process | [B] |
|  | GO:0005794 | Golgi apparatus | [C] |
|  | GO:0016020 | membrane | [C] |
| - Glyco\_transf\_15 | GO:0004377 | glycolipid 2-alpha-mannosyltransferase activity | [M] |
|  | GO:0006486 | protein amino acid glycosylation | [B] |
|  | GO:0016020 | membrane | [C] |
| - Glyco\_transf\_25 | GO:0009103 | lipopolysaccharide biosynthetic process | [B] |
| - Glyco\_transf\_29 | GO:0008373 | sialyltransferase activity | [M] |
|  | GO:0006486 | protein amino acid glycosylation | [B] |
|  | GO:0030173 | integral to Golgi membrane | [C] |
| - Glyco\_transf\_34 | GO:0016758 | transferase activity, transferring hexosyl groups | [M] |
|  | GO:0016021 | integral to membrane | [C] |
| - Glyco\_transf\_36 |  |  |  |  |
| - Glyco\_transf\_43 | GO:0015018 | galactosylgalactosylxylosylprotein 3-beta-glucuronosyltransferase activity | [M] |
|  | GO:0016020 | membrane | [C] |
| - Glyco\_transf\_64 | GO:0016758 | transferase activity, transferring hexosyl groups | [M] |
|  | GO:0031227 | intrinsic to endoplasmic reticulum membrane | [C] |
| - Glycophorin\_A | GO:0016021 | integral to membrane | [C] |
| - Glycos\_trans\_3N |  |  |  |  |
| - Glycos\_transf\_3 | GO:0016757 | transferase activity, transferring glycosyl groups | [M] |
|  | GO:0008152 | metabolic process | [B] |
| - Glyoxal\_oxid\_N |  |  |  |  |
| - GMP\_PDE\_delta |  |  |  |  |
| - GntP\_permease | GO:0015128 | gluconate transmembrane transporter activity | [M] |
|  | GO:0015725 | gluconate transport | [B] |
|  | GO:0016020 | membrane | [C] |
| - GntR | GO:0003700 | transcription factor activity | [M] |
|  | GO:0006355 | regulation of transcription, DNA-dependent | [B] |
|  | GO:0005622 | intracellular | [C] |
| - GON | GO:0004222 | metalloendopeptidase activity | [M] |
|  | GO:0008270 | zinc ion binding | [M] |
|  | GO:0005578 | proteinaceous extracellular matrix | [C] |
| - GPW\_gp25 |  |  |  |  |
| - GRAB |  |  |  |  |
| - GreA\_GreB | GO:0003677 | DNA binding | [M] |
|  | GO:0003711 | transcription elongation regulator activity | [M] |
|  | GO:0006355 | regulation of transcription, DNA-dependent | [B] |
| - GreA\_GreB\_N | GO:0003677 | DNA binding | [M] |
|  | GO:0003711 | transcription elongation regulator activity | [M] |
|  | GO:0006355 | regulation of transcription, DNA-dependent | [B] |
| - GRP |  |  |  |  |
| - Grp1\_Fun34\_YaaH | GO:0016020 | membrane | [C] |
| - GSDH | GO:0016901 | oxidoreductase activity, acting on the CH-OH group of donors, quinone or similar compound as acceptor | [M] |
|  | GO:0048038 | quinone binding | [M] |
|  | GO:0005975 | carbohydrate metabolic process | [B] |
| - GSH-S\_N | GO:0004363 | glutathione synthase activity | [M] |
|  | GO:0006750 | glutathione biosynthetic process | [B] |
| - GSH\_synthase | GO:0004363 | glutathione synthase activity | [M] |
|  | GO:0005524 | ATP binding | [M] |
|  | GO:0006750 | glutathione biosynthetic process | [B] |
| - GSHPx | GO:0004602 | glutathione peroxidase activity | [M] |
|  | GO:0006979 | response to oxidative stress | [B] |
|  | GO:0055114 | oxidation reduction | [B] |
| - GSP\_synth |  |  |  |  |
| - GSPII\_E | GO:0005524 | ATP binding | [M] |
|  | GO:0006810 | transport | [B] |
|  | GO:0005622 | intracellular | [C] |
| - GSPII\_E\_N | GO:0005524 | ATP binding | [M] |
|  | GO:0006810 | transport | [B] |
| - GSPII\_F |  |  |  |  |
| - GSPII\_G |  |  |  |  |
| - GSPII\_IJ | GO:0008565 | protein transporter activity | [M] |
|  | GO:0015628 | protein secretion by the type II secretion system | [B] |
|  | GO:0015627 | type II protein secretion system complex | [C] |
| - GspK | GO:0009306 | protein secretion | [B] |
|  | GO:0016021 | integral to membrane | [C] |
| - GT36\_AF |  |  |  |  |
| - GTP\_CH\_N |  |  |  |  |
| - GTP\_cyclohydro2 | GO:0003935 | GTP cyclohydrolase II activity | [M] |
|  | GO:0009231 | riboflavin biosynthetic process | [B] |
| - GUCT | GO:0003723 | RNA binding | [M] |
|  | GO:0004386 | helicase activity | [M] |
|  | GO:0005524 | ATP binding | [M] |
|  | GO:0005634 | nucleus | [C] |
| - GXGXG | GO:0016491 | oxidoreductase activity | [M] |
|  | GO:0008152 | metabolic process | [B] |
|  | GO:0055114 | oxidation reduction | [B] |
| - H2TH | GO:0003684 | damaged DNA binding | [M] |
|  | GO:0003906 | DNA-(apurinic or apyrimidinic site) lyase activity | [M] |
|  | GO:0008270 | zinc ion binding | [M] |
|  | GO:0016799 | hydrolase activity, hydrolyzing N-glycosyl compounds | [M] |
|  | GO:0006289 | nucleotide-excision repair | [B] |
| - H\_PPase | GO:0004427 | inorganic diphosphatase activity | [M] |
|  | GO:0009678 | hydrogen-translocating pyrophosphatase activity | [M] |
|  | GO:0015992 | proton transport | [B] |
|  | GO:0016020 | membrane | [C] |
| - Haemagg\_act | GO:0005488 | binding | [M] |
| - HALZ | GO:0003677 | DNA binding | [M] |
|  | GO:0006355 | regulation of transcription, DNA-dependent | [B] |
|  | GO:0005634 | nucleus | [C] |
| - HC2 | GO:0003677 | DNA binding | [M] |
|  | GO:0030261 | chromosome condensation | [B] |
| - HCR | GO:0030154 | cell differentiation | [B] |
|  | GO:0005634 | nucleus | [C] |
|  | GO:0005737 | cytoplasm | [C] |
| - HDA2-3 |  |  |  |  |
| - HELP |  |  |  |  |
| - Heme\_oxygenase | GO:0004392 | heme oxygenase (decyclizing) activity | [M] |
|  | GO:0006788 | heme oxidation | [B] |
|  | GO:0055114 | oxidation reduction | [B] |
| - Hemerythrin |  |  |  |  |
| - Hepar\_II\_III |  |  |  |  |
| - HET |  |  |  |  |
| - HI0933\_like |  |  |  |  |
| - HIN |  |  |  |  |
| - Hint | GO:0008233 | peptidase activity | [M] |
|  | GO:0006508 | proteolysis | [B] |
| - HipA\_C |  |  |  |  |
| - HipA\_N |  |  |  |  |
| - HIRAN | GO:0003676 | nucleic acid binding | [M] |
|  | GO:0008270 | zinc ion binding | [M] |
|  | GO:0016818 | hydrolase activity, acting on acid anhydrides, in phosphorus-containing anhydrides | [M] |
| - His\_biosynth | GO:0000105 | histidine biosynthetic process | [B] |
| - His\_kinase | GO:0000155 | two-component sensor activity | [M] |
|  | GO:0000160 | two-component signal transduction system (phosphorelay) | [B] |
|  | GO:0016021 | integral to membrane | [C] |
| - HisG | GO:0003879 | ATP phosphoribosyltransferase activity | [M] |
|  | GO:0000105 | histidine biosynthetic process | [B] |
|  | GO:0005737 | cytoplasm | [C] |
| - HisG\_C | GO:0000287 | magnesium ion binding | [M] |
|  | GO:0003879 | ATP phosphoribosyltransferase activity | [M] |
|  | GO:0000105 | histidine biosynthetic process | [B] |
|  | GO:0005737 | cytoplasm | [C] |
| - Histidinol\_dh | GO:0004399 | histidinol dehydrogenase activity | [M] |
|  | GO:0008270 | zinc ion binding | [M] |
|  | GO:0051287 | NAD or NADH binding | [M] |
|  | GO:0000105 | histidine biosynthetic process | [B] |
| - Histone\_HNS | GO:0003677 | DNA binding | [M] |
|  | GO:0006355 | regulation of transcription, DNA-dependent | [B] |
|  | GO:0005622 | intracellular | [C] |
| - HK | GO:0004417 | hydroxyethylthiazole kinase activity | [M] |
|  | GO:0009228 | thiamin biosynthetic process | [B] |
| - HLH | GO:0030528 | transcription regulator activity | [M] |
|  | GO:0045449 | regulation of transcription | [B] |
| - HlyD | GO:0008565 | protein transporter activity | [M] |
|  | GO:0009306 | protein secretion | [B] |
|  | GO:0016020 | membrane | [C] |
| - HNOB | GO:0004383 | guanylate cyclase activity | [M] |
|  | GO:0020037 | heme binding | [M] |
|  | GO:0006182 | cGMP biosynthetic process | [B] |
| - HNOBA | GO:0004383 | guanylate cyclase activity | [M] |
|  | GO:0006182 | cGMP biosynthetic process | [B] |
| - Hom\_end\_hint | GO:0003677 | DNA binding | [M] |
|  | GO:0004519 | endonuclease activity | [M] |
|  | GO:0030908 | protein splicing | [B] |
| - Homoserine\_dh | GO:0006520 | cellular amino acid metabolic process | [B] |
| - HpaB\_N |  |  |  |  |
| - HTH\_1 | GO:0003700 | transcription factor activity | [M] |
|  | GO:0006355 | regulation of transcription, DNA-dependent | [B] |
| - HTH\_11 |  |  |  |  |
| - HTH\_5 | GO:0003700 | transcription factor activity | [M] |
|  | GO:0006355 | regulation of transcription, DNA-dependent | [B] |
|  | GO:0005622 | intracellular | [C] |
| - HTH\_6 | GO:0003700 | transcription factor activity | [M] |
|  | GO:0006355 | regulation of transcription, DNA-dependent | [B] |
| - HTH\_8 | GO:0003700 | transcription factor activity | [M] |
|  | GO:0006355 | regulation of transcription, DNA-dependent | [B] |
| - HTH\_AraC | GO:0003700 | transcription factor activity | [M] |
|  | GO:0043565 | sequence-specific DNA binding | [M] |
|  | GO:0006355 | regulation of transcription, DNA-dependent | [B] |
|  | GO:0005622 | intracellular | [C] |
| - HTH\_IclR | GO:0003677 | DNA binding | [M] |
|  | GO:0006355 | regulation of transcription, DNA-dependent | [B] |
| - HTH\_psq | GO:0003677 | DNA binding | [M] |
| - HtrL\_YibB |  |  |  |  |
| - HxlR |  |  |  |  |
| - HYR |  |  |  |  |
| - I-set |  |  |  |  |
| - IclR |  |  |  |  |
| - IDH | GO:0004450 | isocitrate dehydrogenase (NADP+) activity | [M] |
|  | GO:0006099 | tricarboxylic acid cycle | [B] |
|  | GO:0055114 | oxidation reduction | [B] |
| - IDO | GO:0020037 | heme binding | [M] |
| - IF2\_N | GO:0003743 | translation initiation factor activity | [M] |
|  | GO:0006413 | translational initiation | [B] |
| - IF\_tail |  |  |  |  |
| - IFP\_35\_N |  |  |  |  |
| - IFRD\_C |  |  |  |  |
| - IFT46\_B\_C |  |  |  |  |
| - IFT57 |  |  |  |  |
| - IGPD | GO:0004424 | imidazoleglycerol-phosphate dehydratase activity | [M] |
|  | GO:0000105 | histidine biosynthetic process | [B] |
| - IGPS |  |  |  |  |
| - IIGP | GO:0005525 | GTP binding | [M] |
|  | GO:0016817 | hydrolase activity, acting on acid anhydrides | [M] |
|  | GO:0016020 | membrane | [C] |
| - IlvC | GO:0004455 | ketol-acid reductoisomerase activity | [M] |
|  | GO:0009082 | branched chain family amino acid biosynthetic process | [B] |
|  | GO:0055114 | oxidation reduction | [B] |
| - ILVD\_EDD | GO:0003824 | catalytic activity | [M] |
|  | GO:0008152 | metabolic process | [B] |
| - IlvN | GO:0004455 | ketol-acid reductoisomerase activity | [M] |
|  | GO:0008652 | cellular amino acid biosynthetic process | [B] |
|  | GO:0055114 | oxidation reduction | [B] |
| - IMS\_HHH |  |  |  |  |
| - INCENP\_N |  |  |  |  |
| - Inhibitor\_I9 | GO:0004252 | serine-type endopeptidase activity | [M] |
|  | GO:0042802 | identical protein binding | [M] |
|  | GO:0043086 | negative regulation of catalytic activity | [B] |
| - Inos-1-P\_synth |  |  |  |  |
| - INSIG |  |  |  |  |
| - Integrase | GO:0003676 | nucleic acid binding | [M] |
|  | GO:0008907 | integrase activity | [M] |
| - Integrase\_Zn | GO:0003677 | DNA binding | [M] |
|  | GO:0008270 | zinc ion binding | [M] |
|  | GO:0008907 | integrase activity | [M] |
|  | GO:0015074 | DNA integration | [B] |
| - Interfer-bind |  |  |  |  |
| - Intron\_maturas2 | GO:0008380 | RNA splicing | [B] |
| - Ion\_trans\_N |  |  |  |  |
| - iPGM\_N | GO:0004619 | phosphoglycerate mutase activity | [M] |
|  | GO:0030145 | manganese ion binding | [M] |
|  | GO:0006007 | glucose catabolic process | [B] |
|  | GO:0005737 | cytoplasm | [C] |
| - IRK |  |  |  |  |
| - Iron\_permease | GO:0055085 | transmembrane transport | [B] |
| - ISN1 | GO:0000287 | magnesium ion binding | [M] |
|  | GO:0016791 | phosphatase activity | [M] |
|  | GO:0009117 | nucleotide metabolic process | [B] |
| - IspD | GO:0003824 | catalytic activity | [M] |
|  | GO:0008299 | isoprenoid biosynthetic process | [B] |
| - Jacalin |  |  |  |  |
| - KaiC |  |  |  |  |
| - KAP\_NTPase |  |  |  |  |
| - KAT11 |  |  |  |  |
| - Kazal\_1 |  |  |  |  |
| - Kazal\_2 |  |  |  |  |
| - Kdo | GO:0005524 | ATP binding | [M] |
|  | GO:0016773 | phosphotransferase activity, alcohol group as acceptor | [M] |
|  | GO:0009103 | lipopolysaccharide biosynthetic process | [B] |
|  | GO:0016020 | membrane | [C] |
| - KilA-N |  |  |  |  |
| - KIX | GO:0003712 | transcription cofactor activity | [M] |
|  | GO:0005515 | protein binding | [M] |
|  | GO:0006355 | regulation of transcription, DNA-dependent | [B] |
| - Kp4 |  |  |  |  |
| - Kringle |  |  |  |  |
| - Kunitz\_BPTI | GO:0004867 | serine-type endopeptidase inhibitor activity | [M] |
| - L31 |  |  |  |  |
| - LacI | GO:0003700 | transcription factor activity | [M] |
|  | GO:0006355 | regulation of transcription, DNA-dependent | [B] |
|  | GO:0005622 | intracellular | [C] |
| - Lactate\_perm | GO:0015129 | lactate transmembrane transporter activity | [M] |
|  | GO:0015727 | lactate transport | [B] |
| - LacY\_symp | GO:0006810 | transport | [B] |
|  | GO:0016020 | membrane | [C] |
| - LamB\_YcsF |  |  |  |  |
| - Laminin\_EGF |  |  |  |  |
| - Laminin\_G\_2 |  |  |  |  |
| - LANC\_like |  |  |  |  |
| - LBR\_tudor |  |  |  |  |
| - LCCL |  |  |  |  |
| - Ldl\_recept\_a |  |  |  |  |
| - LEA\_2 | GO:0009269 | response to desiccation | [B] |
| - LEA\_4 |  |  |  |  |
| - Lectin\_C | GO:0005488 | binding | [M] |
| - LEH |  |  |  |  |
| - Leu\_Phe\_trans | GO:0008914 | leucyltransferase activity | [M] |
|  | GO:0030163 | protein catabolic process | [B] |
| - LeuA\_dimer | GO:0003852 | 2-isopropylmalate synthase activity | [M] |
|  | GO:0009098 | leucine biosynthetic process | [B] |
| - Lgl\_C |  |  |  |  |
| - LicD |  |  |  |  |
| - Lig\_chan | GO:0004970 | ionotropic glutamate receptor activity | [M] |
|  | GO:0005234 | extracellular-glutamate-gated ion channel activity | [M] |
|  | GO:0016020 | membrane | [C] |
| - Lig\_chan-Glu\_bd |  |  |  |  |
| - Lip\_A\_acyltrans | GO:0008415 | acyltransferase activity | [M] |
|  | GO:0009244 | lipopolysaccharide core region biosynthetic process | [B] |
|  | GO:0016021 | integral to membrane | [C] |
| - Lipase |  |  |  |  |
| - Lipase3\_N | GO:0004091 | carboxylesterase activity | [M] |
|  | GO:0016042 | lipid catabolic process | [B] |
| - Lipase\_2 |  |  |  |  |
| - Lipid\_DES | GO:0016705 | oxidoreductase activity, acting on paired donors, with incorporation or reduction of molecular oxygen | [M] |
|  | GO:0006633 | fatty acid biosynthetic process | [B] |
|  | GO:0055114 | oxidation reduction | [B] |
|  | GO:0016021 | integral to membrane | [C] |
| - Lipocalin | GO:0005488 | binding | [M] |
| - Lipoprotein\_9 |  |  |  |  |
| - LpxC | GO:0008759 | UDP-3-O-[3-hydroxymyristoyl] N-acetylglucosamine deacetylase activity | [M] |
|  | GO:0009245 | lipid A biosynthetic process | [B] |
| - LpxK | GO:0005524 | ATP binding | [M] |
|  | GO:0009029 | tetraacyldisaccharide 4'-kinase activity | [M] |
|  | GO:0009245 | lipid A biosynthetic process | [B] |
| - LrgB | GO:0016020 | membrane | [C] |
| - LRR\_1 | GO:0005515 | protein binding | [M] |
| - LRRNT |  |  |  |  |
| - LRS4 |  |  |  |  |
| - Lsm\_interact |  |  |  |  |
| - LtrA |  |  |  |  |
| - LTV |  |  |  |  |
| - Lum\_binding |  |  |  |  |
| - Lyase\_8 | GO:0016829 | lyase activity | [M] |
|  | GO:0005576 | extracellular region | [C] |
| - Lyase\_8\_N |  |  |  |  |
| - LysE | GO:0006865 | amino acid transport | [B] |
|  | GO:0016020 | membrane | [C] |
| - LysR\_substrate |  |  |  |  |
| - Lysyl\_oxidase | GO:0005507 | copper ion binding | [M] |
|  | GO:0016641 | oxidoreductase activity, acting on the CH-NH2 group of donors, oxygen as acceptor | [M] |
|  | GO:0055114 | oxidation reduction | [B] |
| - LYTB | GO:0019288 | isopentenyl diphosphate biosynthetic process, mevalonate-independent pathway | [B] |
|  | GO:0055114 | oxidation reduction | [B] |
| - LytTR |  |  |  |  |
| - M20\_dimer | GO:0016787 | hydrolase activity | [M] |
|  | GO:0046983 | protein dimerization activity | [M] |
| - MADF\_DNA\_bdg |  |  |  |  |
| - Malectin |  |  |  |  |
| - MAM | GO:0016020 | membrane | [C] |
| - MAM33 | GO:0005759 | mitochondrial matrix | [C] |
| - Mannitol\_dh | GO:0016491 | oxidoreductase activity | [M] |
|  | GO:0055114 | oxidation reduction | [B] |
| - Mannitol\_dh\_C | GO:0016491 | oxidoreductase activity | [M] |
|  | GO:0050662 | coenzyme binding | [M] |
|  | GO:0055114 | oxidation reduction | [B] |
| - Mannosyl\_trans3 |  |  |  |  |
| - MAR\_sialic\_bdg |  |  |  |  |
| - MARCKS | GO:0005516 | calmodulin binding | [M] |
| - MarR | GO:0003700 | transcription factor activity | [M] |
|  | GO:0006355 | regulation of transcription, DNA-dependent | [B] |
|  | GO:0005622 | intracellular | [C] |
| - MARVEL | GO:0016020 | membrane | [C] |
| - Matrilin\_ccoil |  |  |  |  |
| - MazG |  |  |  |  |
| - MBD | GO:0003677 | DNA binding | [M] |
|  | GO:0005634 | nucleus | [C] |
| - MBF1 |  |  |  |  |
| - MBT | GO:0045449 | regulation of transcription | [B] |
|  | GO:0005634 | nucleus | [C] |
| - MCE |  |  |  |  |
| - Mcm10 |  |  |  |  |
| - MCPsignal | GO:0004871 | signal transducer activity | [M] |
|  | GO:0006935 | chemotaxis | [B] |
|  | GO:0007165 | signal transduction | [B] |
|  | GO:0016020 | membrane | [C] |
| - Mec-17 |  |  |  |  |
| - Meckelin |  |  |  |  |
| - Med19 | GO:0016455 | RNA polymerase II transcription mediator activity | [M] |
|  | GO:0006357 | regulation of transcription from RNA polymerase II promoter | [B] |
|  | GO:0016592 | mediator complex | [C] |
| - Med2 |  |  |  |  |
| - Med25\_NR-box |  |  |  |  |
| - Med28 |  |  |  |  |
| - Med29 |  |  |  |  |
| - Med8 |  |  |  |  |
| - Med9 |  |  |  |  |
| - Membr\_traf\_MHD |  |  |  |  |
| - Menin | GO:0005634 | nucleus | [C] |
| - MENTAL |  |  |  |  |
| - MerC |  |  |  |  |
| - MerR | GO:0003700 | transcription factor activity | [M] |
|  | GO:0006355 | regulation of transcription, DNA-dependent | [B] |
| - MerR-DNA-bind |  |  |  |  |
| - MerT | GO:0015097 | mercury ion transmembrane transporter activity | [M] |
|  | GO:0015694 | mercury ion transport | [B] |
|  | GO:0016020 | membrane | [C] |
| - Mesd |  |  |  |  |
| - META |  |  |  |  |
| - Metallopep |  |  |  |  |
| - Meth\_synt\_1 | GO:0003871 | 5-methyltetrahydropteroyltriglutamate-homocysteine S-methyltransferase activity | [M] |
|  | GO:0008270 | zinc ion binding | [M] |
|  | GO:0008652 | cellular amino acid biosynthetic process | [B] |
| - Methyltransf\_6 |  |  |  |  |
| - Methyltransf\_FA |  |  |  |  |
| - MethyltransfD12 | GO:0009007 | site-specific DNA-methyltransferase (adenine-specific) activity | [M] |
|  | GO:0006306 | DNA methylation | [B] |
| - MetW |  |  |  |  |
| - MG1 |  |  |  |  |
| - Mg\_chelatase | GO:0016851 | magnesium chelatase activity | [M] |
|  | GO:0015979 | photosynthesis | [B] |
|  | GO:0015995 | chlorophyll biosynthetic process | [B] |
| - Mga |  |  |  |  |
| - MGAT2 | GO:0008455 | alpha-1,6-mannosylglycoprotein 2-beta-N-acetylglucosaminyltransferase activity | [M] |
|  | GO:0009312 | oligosaccharide biosynthetic process | [B] |
|  | GO:0005795 | Golgi stack | [C] |
|  | GO:0016021 | integral to membrane | [C] |
| - MgtC | GO:0016020 | membrane | [C] |
| - MgtE | GO:0008324 | cation transmembrane transporter activity | [M] |
|  | GO:0006812 | cation transport | [B] |
| - MgtE\_N |  |  |  |  |
| - MHYT |  |  |  |  |
| - MiAMP1 | GO:0006952 | defense response | [B] |
|  | GO:0045926 | negative regulation of growth | [B] |
| - Mid2 |  |  |  |  |
| - Mif2 |  |  |  |  |
| - Milton |  |  |  |  |
| - Mis12\_component |  |  |  |  |
| - MitoNEET\_N | GO:0051537 | 2 iron, 2 sulfur cluster binding | [M] |
|  | GO:0043231 | intracellular membrane-bounded organelle | [C] |
| - Mlf1IP |  |  |  |  |
| - MMS1 |  |  |  |  |
| - MMtag |  |  |  |  |
| - MOFRL |  |  |  |  |
| - Molybdop\_Fe4S4 | GO:0016491 | oxidoreductase activity | [M] |
| - Molydop\_binding | GO:0016491 | oxidoreductase activity | [M] |
|  | GO:0030151 | molybdenum ion binding | [M] |
| - Morph\_protein1 |  |  |  |  |
| - MotA\_ExbB | GO:0008565 | protein transporter activity | [M] |
|  | GO:0006810 | transport | [B] |
|  | GO:0016020 | membrane | [C] |
| - Mqo | GO:0008924 | malate dehydrogenase (acceptor) activity | [M] |
|  | GO:0006099 | tricarboxylic acid cycle | [B] |
|  | GO:0055114 | oxidation reduction | [B] |
| - MR\_MLE |  |  |  |  |
| - MR\_MLE\_N |  |  |  |  |
| - MRC1 |  |  |  |  |
| - MreB\_Mbl | GO:0000902 | cell morphogenesis | [B] |
| - MRJP |  |  |  |  |
| - MRP |  |  |  |  |
| - MRP-L28 |  |  |  |  |
| - MRP\_L53 |  |  |  |  |
| - MscL | GO:0005216 | ion channel activity | [M] |
|  | GO:0006810 | transport | [B] |
|  | GO:0016021 | integral to membrane | [C] |
| - MT-A70 | GO:0008168 | methyltransferase activity | [M] |
|  | GO:0006139 | nucleobase, nucleoside, nucleotide and nucleic acid metabolic process | [B] |
| - Mt\_ATP-synt\_B | GO:0015078 | hydrogen ion transmembrane transporter activity | [M] |
|  | GO:0015986 | ATP synthesis coupled proton transport | [B] |
|  | GO:0000276 | mitochondrial proton-transporting ATP synthase complex, coupling factor F(o) | [C] |
| - Mt\_ATP-synt\_D | GO:0015078 | hydrogen ion transmembrane transporter activity | [M] |
|  | GO:0015986 | ATP synthesis coupled proton transport | [B] |
|  | GO:0000276 | mitochondrial proton-transporting ATP synthase complex, coupling factor F(o) | [C] |
| - mTERF |  |  |  |  |
| - MTP18 |  |  |  |  |
| - Mu-like\_gpT |  |  |  |  |
| - Muc\_lac\_enz |  |  |  |  |
| - MuDR |  |  |  |  |
| - Multi\_Drug\_Res | GO:0016021 | integral to membrane | [C] |
| - Mur\_ligase | GO:0005524 | ATP binding | [M] |
|  | GO:0009058 | biosynthetic process | [B] |
| - Mur\_ligase\_C | GO:0005524 | ATP binding | [M] |
|  | GO:0016874 | ligase activity | [M] |
|  | GO:0009058 | biosynthetic process | [B] |
| - MurB\_C | GO:0008762 | UDP-N-acetylmuramate dehydrogenase activity | [M] |
|  | GO:0009252 | peptidoglycan biosynthetic process | [B] |
|  | GO:0055114 | oxidation reduction | [B] |
| - Mus7 |  |  |  |  |
| - Musclin |  |  |  |  |
| - MutS\_IV | GO:0005524 | ATP binding | [M] |
|  | GO:0030983 | mismatched DNA binding | [M] |
|  | GO:0006298 | mismatch repair | [B] |
| - MVIN |  |  |  |  |
| - Myb\_DNA-bind\_2 |  |  |  |  |
| - N6\_Mtase | GO:0003677 | DNA binding | [M] |
|  | GO:0008170 | N-methyltransferase activity | [M] |
|  | GO:0006306 | DNA methylation | [B] |
| - N6\_N4\_Mtase | GO:0003677 | DNA binding | [M] |
|  | GO:0008170 | N-methyltransferase activity | [M] |
|  | GO:0006306 | DNA methylation | [B] |
| - N\_methyl |  |  |  |  |
| - Na\_H\_antiport\_1 | GO:0006814 | sodium ion transport | [B] |
|  | GO:0006885 | regulation of pH | [B] |
|  | GO:0016021 | integral to membrane | [C] |
| - Na\_H\_antiporter | GO:0015385 | sodium:hydrogen antiporter activity | [M] |
|  | GO:0006814 | sodium ion transport | [B] |
|  | GO:0006885 | regulation of pH | [B] |
|  | GO:0016021 | integral to membrane | [C] |
| - Na\_Pi\_cotrans | GO:0015321 | sodium-dependent phosphate transmembrane transporter activity | [M] |
|  | GO:0006817 | phosphate transport | [B] |
|  | GO:0016020 | membrane | [C] |
| - Na\_sulph\_symp | GO:0005215 | transporter activity | [M] |
|  | GO:0006814 | sodium ion transport | [B] |
|  | GO:0055085 | transmembrane transport | [B] |
|  | GO:0016020 | membrane | [C] |
| - Na\_trans\_assoc | GO:0005248 | voltage-gated sodium channel activity | [M] |
|  | GO:0006814 | sodium ion transport | [B] |
|  | GO:0001518 | voltage-gated sodium channel complex | [C] |
| - NAcGluc\_Transf |  |  |  |  |
| - NAD-GH |  |  |  |  |
| - NAD\_binding\_3 | GO:0016491 | oxidoreductase activity | [M] |
|  | GO:0050661 | NADP or NADPH binding | [M] |
| - NAD\_Gly3P\_dh\_C | GO:0016614 | oxidoreductase activity, acting on CH-OH group of donors | [M] |
|  | GO:0005975 | carbohydrate metabolic process | [B] |
|  | GO:0055114 | oxidation reduction | [B] |
| - NAD\_Gly3P\_dh\_N | GO:0016616 | oxidoreductase activity, acting on the CH-OH group of donors, NAD or NADP as acceptor | [M] |
|  | GO:0051287 | NAD or NADH binding | [M] |
|  | GO:0046168 | glycerol-3-phosphate catabolic process | [B] |
|  | GO:0055114 | oxidation reduction | [B] |
|  | GO:0005737 | cytoplasm | [C] |
| - NadA | GO:0008987 | quinolinate synthetase A activity | [M] |
|  | GO:0009435 | NAD biosynthetic process | [B] |
| - NADH-u\_ox-rdase |  |  |  |  |
| - NADH\_ub\_rd\_NUML |  |  |  |  |
| - NARP1 |  |  |  |  |
| - NAS | GO:0030410 | nicotianamine synthase activity | [M] |
|  | GO:0030418 | nicotianamine biosynthetic process | [B] |
| - NC |  |  |  |  |
| - NDUFB10 |  |  |  |  |
| - Ndufs5 |  |  |  |  |
| - Neisseria\_PilC |  |  |  |  |
| - NERD |  |  |  |  |
| - NESP55 |  |  |  |  |
| - NeuB | GO:0016051 | carbohydrate biosynthetic process | [B] |
| - Neur\_chan\_LBD | GO:0005230 | extracellular ligand-gated ion channel activity | [M] |
|  | GO:0006810 | transport | [B] |
|  | GO:0016020 | membrane | [C] |
| - Neur\_chan\_memb | GO:0006811 | ion transport | [B] |
|  | GO:0016020 | membrane | [C] |
| - Nexin\_C |  |  |  |  |
| - NGF | GO:0005102 | receptor binding | [M] |
| - NHase\_alpha | GO:0003824 | catalytic activity | [M] |
|  | GO:0046914 | transition metal ion binding | [M] |
|  | GO:0006807 | nitrogen compound metabolic process | [B] |
| - NHase\_beta | GO:0018822 | nitrile hydratase activity | [M] |
|  | GO:0046914 | transition metal ion binding | [M] |
|  | GO:0006807 | nitrogen compound metabolic process | [B] |
| - Ni\_hydr\_CYTB | GO:0009055 | electron carrier activity | [M] |
|  | GO:0016021 | integral to membrane | [C] |
| - NICE-3 |  |  |  |  |
| - NicO | GO:0046872 | metal ion binding | [M] |
|  | GO:0030001 | metal ion transport | [B] |
|  | GO:0055085 | transmembrane transport | [B] |
|  | GO:0016021 | integral to membrane | [C] |
| - NikM |  |  |  |  |
| - NIR\_SIR | GO:0016491 | oxidoreductase activity | [M] |
|  | GO:0020037 | heme binding | [M] |
|  | GO:0051536 | iron-sulfur cluster binding | [M] |
|  | GO:0055114 | oxidation reduction | [B] |
| - NIR\_SIR\_ferr | GO:0016491 | oxidoreductase activity | [M] |
|  | GO:0055114 | oxidation reduction | [B] |
| - NIT |  |  |  |  |
| - NMT1 |  |  |  |  |
| - NnrU |  |  |  |  |
| - NO\_synthase | GO:0004517 | nitric-oxide synthase activity | [M] |
|  | GO:0006809 | nitric oxide biosynthetic process | [B] |
|  | GO:0055114 | oxidation reduction | [B] |
| - NOA36 | GO:0008270 | zinc ion binding | [M] |
|  | GO:0005634 | nucleus | [C] |
| - NodZ | GO:0016758 | transferase activity, transferring hexosyl groups | [M] |
|  | GO:0009312 | oligosaccharide biosynthetic process | [B] |
|  | GO:0009877 | nodulation | [B] |
| - NPH3 | GO:0004871 | signal transducer activity | [M] |
|  | GO:0009416 | response to light stimulus | [B] |
| - NPIP |  |  |  |  |
| - NPP1 |  |  |  |  |
| - NrfD |  |  |  |  |
| - NT5C | GO:0016791 | phosphatase activity | [M] |
| - Nuc\_deoxyrib\_tr |  |  |  |  |
| - Nuc\_H\_symport | GO:0005337 | nucleoside transmembrane transporter activity | [M] |
|  | GO:0015858 | nucleoside transport | [B] |
|  | GO:0016021 | integral to membrane | [C] |
| - Nucleoplasmin | GO:0003676 | nucleic acid binding | [M] |
| - Nucleos\_tra2\_C |  |  |  |  |
| - Nucleos\_tra2\_N | GO:0005415 | nucleoside:sodium symporter activity | [M] |
|  | GO:0006810 | transport | [B] |
|  | GO:0016020 | membrane | [C] |
| - NUDIX-like | GO:0016787 | hydrolase activity | [M] |
| - Nup188 |  |  |  |  |
| - NUP50 |  |  |  |  |
| - NusB | GO:0003723 | RNA binding | [M] |
|  | GO:0006355 | regulation of transcription, DNA-dependent | [B] |
| - O-FucT |  |  |  |  |
| - OATP | GO:0005215 | transporter activity | [M] |
|  | GO:0006810 | transport | [B] |
|  | GO:0016020 | membrane | [C] |
| - OB\_RNB |  |  |  |  |
| - Occludin\_ELL |  |  |  |  |
| - Octopine\_DH | GO:0016491 | oxidoreductase activity | [M] |
|  | GO:0050662 | coenzyme binding | [M] |
|  | GO:0055114 | oxidation reduction | [B] |
| - ODC\_AZ | GO:0004857 | enzyme inhibitor activity | [M] |
|  | GO:0008073 | ornithine decarboxylase inhibitor activity | [M] |
| - ODV-E18 | GO:0019031 | viral envelope | [C] |
| - OEP | GO:0005215 | transporter activity | [M] |
|  | GO:0006810 | transport | [B] |
| - Ofd1\_CTDD | GO:0005506 | iron ion binding | [M] |
|  | GO:0016706 | oxidoreductase activity, acting on paired donors, with incorporation or reduction of molecular oxygen, 2-oxoglutarate as one donor, and incorporation of one atom each of oxygen into both donors | [M] |
|  | GO:0031418 | L-ascorbic acid binding | [M] |
|  | GO:0055114 | oxidation reduction | [B] |
| - OLF |  |  |  |  |
| - oligo\_HPY | GO:0000166 | nucleotide binding | [M] |
|  | GO:0005524 | ATP binding | [M] |
|  | GO:0015833 | peptide transport | [B] |
| - OmpA | GO:0009279 | cell outer membrane | [C] |
| - OmpH | GO:0005515 | protein binding | [M] |
| - OmpW | GO:0019867 | outer membrane | [C] |
| - Opi1 |  |  |  |  |
| - OprB | GO:0005215 | transporter activity | [M] |
|  | GO:0006810 | transport | [B] |
|  | GO:0016021 | integral to membrane | [C] |
| - Orai-1 |  |  |  |  |
| - Oscp1 |  |  |  |  |
| - OstA\_C | GO:0010033 | response to organic substance | [B] |
|  | GO:0016044 | membrane organization | [B] |
|  | GO:0019867 | outer membrane | [C] |
| - ox\_reductase\_C |  |  |  |  |
| - Oxidored\_nitro | GO:0016491 | oxidoreductase activity | [M] |
|  | GO:0055114 | oxidation reduction | [B] |
| - P-mevalo\_kinase | GO:0004631 | phosphomevalonate kinase activity | [M] |
|  | GO:0006695 | cholesterol biosynthetic process | [B] |
|  | GO:0005737 | cytoplasm | [C] |
| - p25-alpha |  |  |  |  |
| - P\_proprotein | GO:0004252 | serine-type endopeptidase activity | [M] |
|  | GO:0006508 | proteolysis | [B] |
| - PA\_decarbox | GO:0016831 | carboxy-lyase activity | [M] |
| - PaaA\_PaaC |  |  |  |  |
| - Pacifastin\_I | GO:0030414 | peptidase inhibitor activity | [M] |
| - PAD\_porph |  |  |  |  |
| - PAE |  |  |  |  |
| - PAF-AH\_p\_II | GO:0003847 | 1-alkyl-2-acetylglycerophosphocholine esterase activity | [M] |
|  | GO:0016042 | lipid catabolic process | [B] |
| - PAN\_2 |  |  |  |  |
| - PAN\_3 |  |  |  |  |
| - PAP1 |  |  |  |  |
| - PAPA-1 |  |  |  |  |
| - ParcG |  |  |  |  |
| - PARP\_regulatory | GO:0004483 | mRNA (nucleoside-2'-O-)-methyltransferase activity | [M] |
|  | GO:0006370 | mRNA capping | [B] |
|  | GO:0006397 | mRNA processing | [B] |
| - PAS\_2 | GO:0008020 | G-protein coupled photoreceptor activity | [M] |
|  | GO:0007600 | sensory perception | [B] |
|  | GO:0018298 | protein-chromophore linkage | [B] |
|  | GO:0045449 | regulation of transcription | [B] |
| - PAS\_4 |  |  |  |  |
| - PAX | GO:0003677 | DNA binding | [M] |
|  | GO:0045449 | regulation of transcription | [B] |
| - Paxillin | GO:0007160 | cell-matrix adhesion | [B] |
|  | GO:0005856 | cytoskeleton | [C] |
| - PBP\_dimer | GO:0008658 | penicillin binding | [M] |
| - PCIF1\_WW |  |  |  |  |
| - PD40 |  |  |  |  |
| - PDH | GO:0004665 | prephenate dehydrogenase (NADP+) activity | [M] |
|  | GO:0006571 | tyrosine biosynthetic process | [B] |
| - PDT | GO:0004664 | prephenate dehydratase activity | [M] |
|  | GO:0009094 | L-phenylalanine biosynthetic process | [B] |
| - PduV-EutP |  |  |  |  |
| - PdxA | GO:0050570 | 4-hydroxythreonine-4-phosphate dehydrogenase activity | [M] |
|  | GO:0051287 | NAD or NADH binding | [M] |
|  | GO:0008615 | pyridoxine biosynthetic process | [B] |
|  | GO:0055114 | oxidation reduction | [B] |
| - Pec\_lyase\_C |  |  |  |  |
| - Pecanex\_C | GO:0016021 | integral to membrane | [C] |
| - Pectate\_lyase | GO:0030570 | pectate lyase activity | [M] |
|  | GO:0005576 | extracellular region | [C] |
| - Pectinesterase | GO:0030599 | pectinesterase activity | [M] |
|  | GO:0042545 | cell wall modification | [B] |
|  | GO:0005618 | cell wall | [C] |
| - Pedibin |  |  |  |  |
| - PEGA |  |  |  |  |
| - Pellino |  |  |  |  |
| - Pentapeptide\_2 |  |  |  |  |
| - Pentaxin |  |  |  |  |
| - Pep\_M12B\_propep | GO:0004222 | metalloendopeptidase activity | [M] |
|  | GO:0008270 | zinc ion binding | [M] |
|  | GO:0006508 | proteolysis | [B] |
| - Peptidase\_A17 |  |  |  |  |
| - Peptidase\_A3 | GO:0004190 | aspartic-type endopeptidase activity | [M] |
|  | GO:0006508 | proteolysis | [B] |
| - Peptidase\_A8 | GO:0004190 | aspartic-type endopeptidase activity | [M] |
|  | GO:0006508 | proteolysis | [B] |
|  | GO:0016020 | membrane | [C] |
| - Peptidase\_C11 |  |  |  |  |
| - Peptidase\_C65 |  |  |  |  |
| - Peptidase\_C69 | GO:0016805 | dipeptidase activity | [M] |
|  | GO:0006508 | proteolysis | [B] |
| - Peptidase\_M10 | GO:0004222 | metalloendopeptidase activity | [M] |
|  | GO:0006508 | proteolysis | [B] |
|  | GO:0005578 | proteinaceous extracellular matrix | [C] |
| - Peptidase\_M13 | GO:0004222 | metalloendopeptidase activity | [M] |
|  | GO:0006508 | proteolysis | [B] |
| - Peptidase\_M13\_N | GO:0008237 | metallopeptidase activity | [M] |
|  | GO:0006508 | proteolysis | [B] |
| - Peptidase\_M15\_3 |  |  |  |  |
| - Peptidase\_M19 | GO:0008235 | metalloexopeptidase activity | [M] |
|  | GO:0008239 | dipeptidyl-peptidase activity | [M] |
|  | GO:0016805 | dipeptidase activity | [M] |
|  | GO:0006508 | proteolysis | [B] |
| - Peptidase\_M29 | GO:0004177 | aminopeptidase activity | [M] |
|  | GO:0006508 | proteolysis | [B] |
| - Peptidase\_M32 | GO:0004181 | metallocarboxypeptidase activity | [M] |
|  | GO:0006508 | proteolysis | [B] |
| - Peptidase\_M36 |  |  |  |  |
| - Peptidase\_M43 |  |  |  |  |
| - Peptidase\_M54 | GO:0008237 | metallopeptidase activity | [M] |
|  | GO:0008270 | zinc ion binding | [M] |
| - Peptidase\_M6 | GO:0008233 | peptidase activity | [M] |
|  | GO:0006508 | proteolysis | [B] |
| - Peptidase\_M64 |  |  |  |  |
| - Peptidase\_M75 |  |  |  |  |
| - Peptidase\_S11 | GO:0009002 | serine-type D-Ala-D-Ala carboxypeptidase activity | [M] |
|  | GO:0006508 | proteolysis | [B] |
| - Peptidase\_S15 | GO:0004177 | aminopeptidase activity | [M] |
|  | GO:0006508 | proteolysis | [B] |
| - Peptidase\_S26 |  |  |  |  |
| - Peptidase\_S41 | GO:0008236 | serine-type peptidase activity | [M] |
|  | GO:0006508 | proteolysis | [B] |
| - Peptidase\_S49\_N | GO:0004252 | serine-type endopeptidase activity | [M] |
|  | GO:0005886 | plasma membrane | [C] |
| - Peptidase\_S51 | GO:0008236 | serine-type peptidase activity | [M] |
|  | GO:0006508 | proteolysis | [B] |
| - Peptidase\_S58 |  |  |  |  |
| - Peptidase\_S64 |  |  |  |  |
| - Peptidase\_S66 |  |  |  |  |
| - Peptidase\_U32 | GO:0008233 | peptidase activity | [M] |
|  | GO:0006508 | proteolysis | [B] |
| - PepX\_C | GO:0008239 | dipeptidyl-peptidase activity | [M] |
| - Per1 |  |  |  |  |
| - Peripla\_BP\_1 |  |  |  |  |
| - Peripla\_BP\_2 | GO:0005381 | iron ion transmembrane transporter activity | [M] |
|  | GO:0006827 | high-affinity iron ion transport | [B] |
| - peroxidase | GO:0004601 | peroxidase activity | [M] |
|  | GO:0020037 | heme binding | [M] |
|  | GO:0006979 | response to oxidative stress | [B] |
|  | GO:0055114 | oxidation reduction | [B] |
| - Peroxidase\_2 | GO:0004601 | peroxidase activity | [M] |
| - Pex24p |  |  |  |  |
| - PFL | GO:0008861 | formate C-acetyltransferase activity | [M] |
|  | GO:0006006 | glucose metabolic process | [B] |
|  | GO:0005737 | cytoplasm | [C] |
| - PGA\_cap |  |  |  |  |
| - PGPGW |  |  |  |  |
| - PhaC\_N | GO:0008415 | acyltransferase activity | [M] |
|  | GO:0042619 | poly-hydroxybutyrate biosynthetic process | [B] |
| - Phe\_tRNA-synt\_N | GO:0000166 | nucleotide binding | [M] |
|  | GO:0004826 | phenylalanine-tRNA ligase activity | [M] |
|  | GO:0005524 | ATP binding | [M] |
|  | GO:0006412 | translation | [B] |
|  | GO:0006432 | phenylalanyl-tRNA aminoacylation | [B] |
|  | GO:0005737 | cytoplasm | [C] |
| - Phenol\_Hydrox | GO:0006725 | cellular aromatic compound metabolic process | [B] |
|  | GO:0055114 | oxidation reduction | [B] |
| - Phi\_1 |  |  |  |  |
| - PhnA |  |  |  |  |
| - PHO4 | GO:0005315 | inorganic phosphate transmembrane transporter activity | [M] |
|  | GO:0006817 | phosphate transport | [B] |
|  | GO:0016020 | membrane | [C] |
| - PhoH | GO:0005524 | ATP binding | [M] |
| - Phosphoesterase | GO:0016788 | hydrolase activity, acting on ester bonds | [M] |
| - Phospholip\_A2\_1 | GO:0004623 | phospholipase A2 activity | [M] |
|  | GO:0005509 | calcium ion binding | [M] |
|  | GO:0016042 | lipid catabolic process | [B] |
| - Phospholip\_A2\_2 | GO:0004623 | phospholipase A2 activity | [M] |
|  | GO:0005509 | calcium ion binding | [M] |
|  | GO:0006644 | phospholipid metabolic process | [B] |
|  | GO:0005576 | extracellular region | [C] |
| - PhoU |  |  |  |  |
| - PhoU\_div |  |  |  |  |
| - PHR |  |  |  |  |
| - Phtf-FEM1B\_bdg |  |  |  |  |
| - Phytochrome | GO:0004872 | receptor activity | [M] |
|  | GO:0008020 | G-protein coupled photoreceptor activity | [M] |
|  | GO:0006355 | regulation of transcription, DNA-dependent | [B] |
|  | GO:0007600 | sensory perception | [B] |
|  | GO:0018298 | protein-chromophore linkage | [B] |
| - PhzC-PhzF | GO:0003824 | catalytic activity | [M] |
|  | GO:0009058 | biosynthetic process | [B] |
| - Pilin | GO:0007155 | cell adhesion | [B] |
|  | GO:0009289 | pilus | [C] |
| - PilP |  |  |  |  |
| - PIN |  |  |  |  |
| - PKD |  |  |  |  |
| - PLA1 | GO:0004620 | phospholipase activity | [M] |
|  | GO:0006629 | lipid metabolic process | [B] |
|  | GO:0016020 | membrane | [C] |
| - PLA2\_B | GO:0004620 | phospholipase activity | [M] |
|  | GO:0009395 | phospholipid catabolic process | [B] |
| - PLA2G12 | GO:0004623 | phospholipase A2 activity | [M] |
|  | GO:0005509 | calcium ion binding | [M] |
|  | GO:0016042 | lipid catabolic process | [B] |
|  | GO:0005576 | extracellular region | [C] |
| - Plant\_NMP1 |  |  |  |  |
| - Plant\_tran |  |  |  |  |
| - Plasmod\_Pvs28 | GO:0009986 | cell surface | [C] |
|  | GO:0016020 | membrane | [C] |
| - Plasmodium\_HRP |  |  |  |  |
| - PLAT |  |  |  |  |
| - PLU-1 |  |  |  |  |
| - Plug | GO:0004872 | receptor activity | [M] |
|  | GO:0005215 | transporter activity | [M] |
|  | GO:0006810 | transport | [B] |
|  | GO:0016020 | membrane | [C] |
| - PNGaseA |  |  |  |  |
| - PNPase | GO:0000175 | 3'-5'-exoribonuclease activity | [M] |
|  | GO:0003723 | RNA binding | [M] |
|  | GO:0006396 | RNA processing | [B] |
| - Pol\_alpha\_B\_N |  |  |  |  |
| - Polysacc\_synt\_2 | GO:0009058 | biosynthetic process | [B] |
| - Porin\_1 | GO:0005215 | transporter activity | [M] |
|  | GO:0006810 | transport | [B] |
|  | GO:0016020 | membrane | [C] |
| - potato\_inhibit | GO:0004867 | serine-type endopeptidase inhibitor activity | [M] |
|  | GO:0009611 | response to wounding | [B] |
| - POTRA\_2 |  |  |  |  |
| - PPK2 |  |  |  |  |
| - Ppx-GppA |  |  |  |  |
| - PQQ |  |  |  |  |
| - PRA-CH | GO:0004635 | phosphoribosyl-AMP cyclohydrolase activity | [M] |
|  | GO:0000105 | histidine biosynthetic process | [B] |
| - PRA-PH | GO:0004636 | phosphoribosyl-ATP diphosphatase activity | [M] |
|  | GO:0000105 | histidine biosynthetic process | [B] |
| - PRAI | GO:0004640 | phosphoribosylanthranilate isomerase activity | [M] |
|  | GO:0006568 | tryptophan metabolic process | [B] |
| - PRiA4\_ORF3 |  |  |  |  |
| - Pro\_racemase | GO:0018112 | proline racemase activity | [M] |
| - Prog\_receptor | GO:0003677 | DNA binding | [M] |
|  | GO:0003707 | steroid hormone receptor activity | [M] |
|  | GO:0005496 | steroid binding | [M] |
|  | GO:0006355 | regulation of transcription, DNA-dependent | [B] |
|  | GO:0005634 | nucleus | [C] |
| - Prominin | GO:0016021 | integral to membrane | [C] |
| - Propep\_M14 | GO:0004180 | carboxypeptidase activity | [M] |
|  | GO:0006508 | proteolysis | [B] |
| - Protamine\_P1 | GO:0003677 | DNA binding | [M] |
|  | GO:0007283 | spermatogenesis | [B] |
|  | GO:0000786 | nucleosome | [C] |
|  | GO:0005634 | nucleus | [C] |
| - PrpF |  |  |  |  |
| - PRT\_C |  |  |  |  |
| - PS\_pyruv\_trans | GO:0016740 | transferase activity | [M] |
| - PsaA\_PsaB | GO:0015979 | photosynthesis | [B] |
|  | GO:0009522 | photosystem I | [C] |
|  | GO:0009579 | thylakoid | [C] |
|  | GO:0016021 | integral to membrane | [C] |
| - PSP1 |  |  |  |  |
| - PTB |  |  |  |  |
| - PTS\_EIIA\_2 | GO:0005351 | sugar:hydrogen symporter activity | [M] |
|  | GO:0006810 | transport | [B] |
|  | GO:0009401 | phosphoenolpyruvate-dependent sugar phosphotransferase system | [B] |
| - PTS\_EIIC | GO:0005351 | sugar:hydrogen symporter activity | [M] |
|  | GO:0008982 | protein-N(PI)-phosphohistidine-sugar phosphotransferase activity | [M] |
|  | GO:0009401 | phosphoenolpyruvate-dependent sugar phosphotransferase system | [B] |
|  | GO:0016020 | membrane | [C] |
| - PTS\_IIB | GO:0005351 | sugar:hydrogen symporter activity | [M] |
|  | GO:0009401 | phosphoenolpyruvate-dependent sugar phosphotransferase system | [B] |
| - PUB |  |  |  |  |
| - PUD | GO:0030246 | carbohydrate binding | [M] |
|  | GO:0005975 | carbohydrate metabolic process | [B] |
| - Pur\_DNA\_glyco | GO:0003677 | DNA binding | [M] |
|  | GO:0003905 | alkylbase DNA N-glycosylase activity | [M] |
|  | GO:0006284 | base-excision repair | [B] |
| - PurA |  |  |  |  |
| - Put\_Phosphatase | GO:0016791 | phosphatase activity | [M] |
| - PXA |  |  |  |  |
| - PYNP\_C | GO:0016763 | transferase activity, transferring pentosyl groups | [M] |
|  | GO:0006213 | pyrimidine nucleoside metabolic process | [B] |
| - Queuosine\_synth | GO:0016740 | transferase activity | [M] |
|  | GO:0016853 | isomerase activity | [M] |
|  | GO:0008616 | queuosine biosynthetic process | [B] |
| - Rad50\_zn\_hook | GO:0004518 | nuclease activity | [M] |
|  | GO:0005524 | ATP binding | [M] |
|  | GO:0008270 | zinc ion binding | [M] |
|  | GO:0006281 | DNA repair | [B] |
| - Radial\_spoke |  |  |  |  |
| - Radial\_spoke\_3 |  |  |  |  |
| - Raffinose\_syn |  |  |  |  |
| - RAG2 | GO:0003677 | DNA binding | [M] |
|  | GO:0006310 | DNA recombination | [B] |
|  | GO:0005634 | nucleus | [C] |
| - RALF |  |  |  |  |
| - Ran-binding |  |  |  |  |
| - RAP |  |  |  |  |
| - Rb\_C |  |  |  |  |
| - RBFA | GO:0006364 | rRNA processing | [B] |
| - RbsD\_FucU | GO:0008643 | carbohydrate transport | [B] |
| - RDD |  |  |  |  |
| - RdgC | GO:0006310 | DNA recombination | [B] |
| - Rdx |  |  |  |  |
| - Recep\_L\_domain | GO:0016020 | membrane | [C] |
| - RED\_C |  |  |  |  |
| - Reeler |  |  |  |  |
| - Renin\_r | GO:0004872 | receptor activity | [M] |
|  | GO:0016021 | integral to membrane | [C] |
| - Rep-A\_N | GO:0003677 | DNA binding | [M] |
|  | GO:0006260 | DNA replication | [B] |
|  | GO:0005634 | nucleus | [C] |
| - Rep\_3 | GO:0003887 | DNA-directed DNA polymerase activity | [M] |
|  | GO:0006270 | DNA replication initiation | [B] |
|  | GO:0005727 | extrachromosomal circular DNA | [C] |
| - Rep\_fac-A\_3 |  |  |  |  |
| - Reprolysin | GO:0004222 | metalloendopeptidase activity | [M] |
|  | GO:0006508 | proteolysis | [B] |
| - RhaT | GO:0015153 | rhamnose transmembrane transporter activity | [M] |
|  | GO:0015762 | rhamnose transport | [B] |
|  | GO:0016021 | integral to membrane | [C] |
| - RhgB\_N | GO:0016837 | carbon-oxygen lyase activity, acting on polysaccharides | [M] |
|  | GO:0030246 | carbohydrate binding | [M] |
|  | GO:0005975 | carbohydrate metabolic process | [B] |
| - Rho\_N | GO:0003715 | transcription termination factor activity | [M] |
|  | GO:0006353 | transcription termination | [B] |
| - RHS |  |  |  |  |
| - RHS\_repeat |  |  |  |  |
| - RHSP |  |  |  |  |
| - RIB43A |  |  |  |  |
| - RibD\_C | GO:0008703 | 5-amino-6-(5-phosphoribosylamino)uracil reductase activity | [M] |
|  | GO:0009231 | riboflavin biosynthetic process | [B] |
| - Ribonuclease\_BN | GO:0004540 | ribonuclease activity | [M] |
| - Ribonuclease\_P | GO:0000049 | tRNA binding | [M] |
|  | GO:0004526 | ribonuclease P activity | [M] |
|  | GO:0008033 | tRNA processing | [B] |
| - Ribosomal\_L19 | GO:0003735 | structural constituent of ribosome | [M] |
|  | GO:0006412 | translation | [B] |
|  | GO:0005622 | intracellular | [C] |
|  | GO:0005840 | ribosome | [C] |
| - Ribosomal\_L31 | GO:0003735 | structural constituent of ribosome | [M] |
|  | GO:0006412 | translation | [B] |
|  | GO:0005622 | intracellular | [C] |
|  | GO:0005840 | ribosome | [C] |
| - Ribosomal\_L35p | GO:0003735 | structural constituent of ribosome | [M] |
|  | GO:0006412 | translation | [B] |
|  | GO:0005622 | intracellular | [C] |
|  | GO:0005840 | ribosome | [C] |
| - Ribosomal\_L41 | GO:0003735 | structural constituent of ribosome | [M] |
|  | GO:0006412 | translation | [B] |
|  | GO:0005840 | ribosome | [C] |
| - Ribosomal\_L6e\_N | GO:0003735 | structural constituent of ribosome | [M] |
|  | GO:0006412 | translation | [B] |
|  | GO:0005622 | intracellular | [C] |
|  | GO:0005840 | ribosome | [C] |
| - Ribosomal\_S16 | GO:0003735 | structural constituent of ribosome | [M] |
|  | GO:0006412 | translation | [B] |
|  | GO:0005622 | intracellular | [C] |
|  | GO:0005840 | ribosome | [C] |
| - Ribosomal\_S20p | GO:0003723 | RNA binding | [M] |
|  | GO:0003735 | structural constituent of ribosome | [M] |
|  | GO:0006412 | translation | [B] |
|  | GO:0005622 | intracellular | [C] |
|  | GO:0005840 | ribosome | [C] |
| - Ribosomal\_S21 | GO:0003735 | structural constituent of ribosome | [M] |
|  | GO:0006412 | translation | [B] |
|  | GO:0005622 | intracellular | [C] |
|  | GO:0005840 | ribosome | [C] |
| - Ribosomal\_S23p |  |  |  |  |
| - Ribosomal\_S6 | GO:0003735 | structural constituent of ribosome | [M] |
|  | GO:0019843 | rRNA binding | [M] |
|  | GO:0006412 | translation | [B] |
|  | GO:0005840 | ribosome | [C] |
| - RIIa | GO:0008603 | cAMP-dependent protein kinase regulator activity | [M] |
|  | GO:0007165 | signal transduction | [B] |
| - Ring\_hydroxyl\_A | GO:0005506 | iron ion binding | [M] |
|  | GO:0016708 | oxidoreductase activity, acting on paired donors, with incorporation or reduction of molecular oxygen, NADH or NADPH as one donor, and incorporation of two atoms of oxygen into one donor | [M] |
|  | GO:0051537 | 2 iron, 2 sulfur cluster binding | [M] |
|  | GO:0019439 | aromatic compound catabolic process | [B] |
|  | GO:0055114 | oxidation reduction | [B] |
| - RIP | GO:0030598 | rRNA N-glycosylase activity | [M] |
|  | GO:0017148 | negative regulation of translation | [B] |
| - RmuC |  |  |  |  |
| - RNA\_bind | GO:0003723 | RNA binding | [M] |
|  | GO:0004535 | poly(A)-specific ribonuclease activity | [M] |
|  | GO:0046872 | metal ion binding | [M] |
|  | GO:0006402 | mRNA catabolic process | [B] |
|  | GO:0005634 | nucleus | [C] |
|  | GO:0005737 | cytoplasm | [C] |
| - RNA\_GG\_bind |  |  |  |  |
| - RNA\_Me\_trans |  |  |  |  |
| - RNA\_pol\_A\_CTD | GO:0003677 | DNA binding | [M] |
|  | GO:0003899 | DNA-directed RNA polymerase activity | [M] |
|  | GO:0006350 | transcription | [B] |
| - RNA\_pol\_Rpb1\_7 | GO:0003677 | DNA binding | [M] |
|  | GO:0003899 | DNA-directed RNA polymerase activity | [M] |
|  | GO:0006350 | transcription | [B] |
| - RNA\_pol\_Rpb2\_2 | GO:0003677 | DNA binding | [M] |
|  | GO:0003899 | DNA-directed RNA polymerase activity | [M] |
|  | GO:0006350 | transcription | [B] |
| - RNA\_pol\_Rpb2\_45 | GO:0003899 | DNA-directed RNA polymerase activity | [M] |
|  | GO:0006351 | transcription, DNA-dependent | [B] |
| - RNA\_pol\_Rpc82 | GO:0003677 | DNA binding | [M] |
|  | GO:0003899 | DNA-directed RNA polymerase activity | [M] |
|  | GO:0006350 | transcription | [B] |
| - RNase\_E\_G |  |  |  |  |
| - Rod\_C |  |  |  |  |
| - RPA\_C |  |  |  |  |
| - RPE65 |  |  |  |  |
| - RPEL |  |  |  |  |
| - Rpp20 |  |  |  |  |
| - RR\_TM4-6 | GO:0005219 | ryanodine-sensitive calcium-release channel activity | [M] |
|  | GO:0006874 | cellular calcium ion homeostasis | [B] |
|  | GO:0016021 | integral to membrane | [C] |
| - RTC\_insert |  |  |  |  |
| - RuvC | GO:0004520 | endodeoxyribonuclease activity | [M] |
|  | GO:0006281 | DNA repair | [B] |
|  | GO:0006310 | DNA recombination | [B] |
| - Rxt3 |  |  |  |  |
| - RyR |  |  |  |  |
| - S-antigen |  |  |  |  |
| - S1-P1\_nuclease | GO:0003676 | nucleic acid binding | [M] |
|  | GO:0004519 | endonuclease activity | [M] |
|  | GO:0006308 | DNA catabolic process | [B] |
| - S6PP |  |  |  |  |
| - SAB | GO:0008092 | cytoskeletal protein binding | [M] |
|  | GO:0030866 | cortical actin cytoskeleton organization | [B] |
|  | GO:0005856 | cytoskeleton | [C] |
| - Saccharop\_dh\_N |  |  |  |  |
| - SAF |  |  |  |  |
| - SAND | GO:0003677 | DNA binding | [M] |
|  | GO:0005634 | nucleus | [C] |
| - SANTA |  |  |  |  |
| - SBF | GO:0008508 | bile acid:sodium symporter activity | [M] |
|  | GO:0006814 | sodium ion transport | [B] |
|  | GO:0016020 | membrane | [C] |
| - SBP56 | GO:0008430 | selenium binding | [M] |
| - SBP\_bac\_1 | GO:0005215 | transporter activity | [M] |
|  | GO:0006810 | transport | [B] |
| - SBP\_bac\_3 | GO:0005215 | transporter activity | [M] |
|  | GO:0006810 | transport | [B] |
|  | GO:0030288 | outer membrane-bounded periplasmic space | [C] |
| - SBP\_bac\_5 | GO:0005215 | transporter activity | [M] |
|  | GO:0006810 | transport | [B] |
| - SBP\_bac\_7 | GO:0006810 | transport | [B] |
|  | GO:0030288 | outer membrane-bounded periplasmic space | [C] |
| - SCA7 |  |  |  |  |
| - Scs3p |  |  |  |  |
| - SDF | GO:0017153 | sodium:dicarboxylate symporter activity | [M] |
|  | GO:0006835 | dicarboxylic acid transport | [B] |
|  | GO:0016020 | membrane | [C] |
| - SDH\_alpha | GO:0003941 | L-serine ammonia-lyase activity | [M] |
|  | GO:0051539 | 4 iron, 4 sulfur cluster binding | [M] |
|  | GO:0006094 | gluconeogenesis | [B] |
| - SDH\_beta | GO:0003941 | L-serine ammonia-lyase activity | [M] |
|  | GO:0051539 | 4 iron, 4 sulfur cluster binding | [M] |
|  | GO:0006094 | gluconeogenesis | [B] |
| - Sec62 | GO:0008565 | protein transporter activity | [M] |
|  | GO:0015031 | protein transport | [B] |
|  | GO:0016021 | integral to membrane | [C] |
| - Sec66 |  |  |  |  |
| - Sec\_GG | GO:0008565 | protein transporter activity | [M] |
|  | GO:0015628 | protein secretion by the type II secretion system | [B] |
|  | GO:0015627 | type II protein secretion system complex | [C] |
| - SecA\_SW | GO:0017038 | protein import | [B] |
|  | GO:0016020 | membrane | [C] |
| - SecD\_SecF | GO:0008565 | protein transporter activity | [M] |
|  | GO:0015628 | protein secretion by the type II secretion system | [B] |
|  | GO:0015627 | type II protein secretion system complex | [C] |
| - Secretin | GO:0009306 | protein secretion | [B] |
| - Secretin\_N |  |  |  |  |
| - Selenoprotein\_S | GO:0008430 | selenium binding | [M] |
|  | GO:0006886 | intracellular protein transport | [B] |
|  | GO:0030176 | integral to endoplasmic reticulum membrane | [C] |
| - Self-incomp\_S1 |  |  |  |  |
| - Semialdhyde\_dhC | GO:0016620 | oxidoreductase activity, acting on the aldehyde or oxo group of donors, NAD or NADP as acceptor | [M] |
|  | GO:0046983 | protein dimerization activity | [M] |
|  | GO:0008652 | cellular amino acid biosynthetic process | [B] |
|  | GO:0005737 | cytoplasm | [C] |
| - Sfi1 |  |  |  |  |
| - SH3\_3 |  |  |  |  |
| - SH3BP5 |  |  |  |  |
| - She9\_MDM33 |  |  |  |  |
| - Shikimate\_DH | GO:0004764 | shikimate 5-dehydrogenase activity | [M] |
|  | GO:0055114 | oxidation reduction | [B] |
|  | GO:0005737 | cytoplasm | [C] |
| - Shikimate\_dh\_N |  |  |  |  |
| - ShK |  |  |  |  |
| - SHNi-TPR |  |  |  |  |
| - Shugoshin\_C | GO:0045132 | meiotic chromosome segregation | [B] |
|  | GO:0000775 | chromosome, centromeric region | [C] |
|  | GO:0005634 | nucleus | [C] |
| - Siah-Interact\_N |  |  |  |  |
| - Sigma54\_activat | GO:0005524 | ATP binding | [M] |
|  | GO:0008134 | transcription factor binding | [M] |
|  | GO:0006355 | regulation of transcription, DNA-dependent | [B] |
|  | GO:0005622 | intracellular | [C] |
| - Sigma70\_ner | GO:0003677 | DNA binding | [M] |
|  | GO:0003700 | transcription factor activity | [M] |
|  | GO:0016987 | sigma factor activity | [M] |
|  | GO:0006352 | transcription initiation | [B] |
|  | GO:0006355 | regulation of transcription, DNA-dependent | [B] |
| - Sigma70\_r1\_1 | GO:0003677 | DNA binding | [M] |
|  | GO:0030528 | transcription regulator activity | [M] |
|  | GO:0045449 | regulation of transcription | [B] |
| - Sigma70\_r1\_2 | GO:0003677 | DNA binding | [M] |
|  | GO:0003700 | transcription factor activity | [M] |
|  | GO:0016987 | sigma factor activity | [M] |
|  | GO:0006352 | transcription initiation | [B] |
|  | GO:0006355 | regulation of transcription, DNA-dependent | [B] |
| - Sigma70\_r2 | GO:0003677 | DNA binding | [M] |
|  | GO:0003700 | transcription factor activity | [M] |
|  | GO:0016987 | sigma factor activity | [M] |
|  | GO:0006352 | transcription initiation | [B] |
|  | GO:0006355 | regulation of transcription, DNA-dependent | [B] |
| - Sigma70\_r3 | GO:0003677 | DNA binding | [M] |
|  | GO:0003700 | transcription factor activity | [M] |
|  | GO:0016987 | sigma factor activity | [M] |
|  | GO:0006352 | transcription initiation | [B] |
|  | GO:0006355 | regulation of transcription, DNA-dependent | [B] |
| - Sigma70\_r4 | GO:0003677 | DNA binding | [M] |
|  | GO:0003700 | transcription factor activity | [M] |
|  | GO:0016987 | sigma factor activity | [M] |
|  | GO:0006352 | transcription initiation | [B] |
|  | GO:0006355 | regulation of transcription, DNA-dependent | [B] |
| - Sigma70\_r4\_2 | GO:0003677 | DNA binding | [M] |
|  | GO:0003700 | transcription factor activity | [M] |
|  | GO:0016987 | sigma factor activity | [M] |
|  | GO:0006352 | transcription initiation | [B] |
|  | GO:0006355 | regulation of transcription, DNA-dependent | [B] |
| - Silic\_transp |  |  |  |  |
| - SIMPL |  |  |  |  |
| - Siva | GO:0005175 | CD27 receptor binding | [M] |
|  | GO:0043065 | positive regulation of apoptosis | [B] |
|  | GO:0005737 | cytoplasm | [C] |
| - SK\_channel |  |  |  |  |
| - SKN1 |  |  |  |  |
| - SLD3 |  |  |  |  |
| - SLR1-BP |  |  |  |  |
| - SMI1\_KNR4 |  |  |  |  |
| - SmpB | GO:0003723 | RNA binding | [M] |
|  | GO:0006412 | translation | [B] |
| - SNAP-25 |  |  |  |  |
| - SNDH |  |  |  |  |
| - SNF | GO:0005328 | neurotransmitter:sodium symporter activity | [M] |
|  | GO:0006836 | neurotransmitter transport | [B] |
|  | GO:0005887 | integral to plasma membrane | [C] |
|  | GO:0016020 | membrane | [C] |
| - SnoaL |  |  |  |  |
| - Somatomedin\_B | GO:0005044 | scavenger receptor activity | [M] |
|  | GO:0030247 | polysaccharide binding | [M] |
|  | GO:0006955 | immune response | [B] |
| - SOUL |  |  |  |  |
| - SoxE |  |  |  |  |
| - Spc24 |  |  |  |  |
| - Spc42p |  |  |  |  |
| - Spc7 |  |  |  |  |
| - Spherulin4 |  |  |  |  |
| - SPO22 |  |  |  |  |
| - SpoA |  |  |  |  |
| - SPOC |  |  |  |  |
| - SpoIIID |  |  |  |  |
| - Spond\_N |  |  |  |  |
| - Sporozoite\_P67 |  |  |  |  |
| - SRA1 |  |  |  |  |
| - SRCR | GO:0005044 | scavenger receptor activity | [M] |
|  | GO:0016020 | membrane | [C] |
| - SSB | GO:0003697 | single-stranded DNA binding | [M] |
| - SSF | GO:0005215 | transporter activity | [M] |
|  | GO:0006810 | transport | [B] |
|  | GO:0055085 | transmembrane transport | [B] |
|  | GO:0016020 | membrane | [C] |
| - Stanniocalcin | GO:0005179 | hormone activity | [M] |
|  | GO:0005576 | extracellular region | [C] |
| - Sterol-sensing |  |  |  |  |
| - Stig1 |  |  |  |  |
| - Strep\_67kDa\_ant |  |  |  |  |
| - SufE |  |  |  |  |
| - SUFU |  |  |  |  |
| - Sugar-bind | GO:0030246 | carbohydrate binding | [M] |
|  | GO:0030528 | transcription regulator activity | [M] |
| - Sulfatase | GO:0008484 | sulfuric ester hydrolase activity | [M] |
|  | GO:0008152 | metabolic process | [B] |
| - SurA\_N |  |  |  |  |
| - SurE | GO:0016787 | hydrolase activity | [M] |
| - SURF4 |  |  |  |  |
| - Surf\_Ag\_VNR | GO:0019867 | outer membrane | [C] |
| - Sushi |  |  |  |  |
| - T5orf172 |  |  |  |  |
| - TACC |  |  |  |  |
| - Tannase |  |  |  |  |
| - TatC |  |  |  |  |
| - TauD | GO:0016491 | oxidoreductase activity | [M] |
|  | GO:0055114 | oxidation reduction | [B] |
| - Tautomerase | GO:0016853 | isomerase activity | [M] |
|  | GO:0006725 | cellular aromatic compound metabolic process | [B] |
| - TB2\_DP1\_HVA22 |  |  |  |  |
| - TBP-binding |  |  |  |  |
| - TCP |  |  |  |  |
| - Tcp10\_C |  |  |  |  |
| - Tcp11 |  |  |  |  |
| - TEA | GO:0003700 | transcription factor activity | [M] |
|  | GO:0006355 | regulation of transcription, DNA-dependent | [B] |
|  | GO:0005634 | nucleus | [C] |
| - Tektin | GO:0000226 | microtubule cytoskeleton organization | [B] |
|  | GO:0005874 | microtubule | [C] |
| - Telo\_bind | GO:0003677 | DNA binding | [M] |
|  | GO:0000723 | telomere maintenance | [B] |
|  | GO:0000784 | nuclear chromosome, telomeric region | [C] |
| - TENA\_THI-4 |  |  |  |  |
| - TerC | GO:0016021 | integral to membrane | [C] |
| - Terminase\_4 |  |  |  |  |
| - Terminase\_6 |  |  |  |  |
| - Terminase\_GpA |  |  |  |  |
| - TetR\_C\_2 | GO:0003677 | DNA binding | [M] |
| - TetR\_N | GO:0003700 | transcription factor activity | [M] |
|  | GO:0006355 | regulation of transcription, DNA-dependent | [B] |
| - Tex\_N |  |  |  |  |
| - TFIIE-A\_C-term |  |  |  |  |
| - TFR\_dimer |  |  |  |  |
| - Thaumatin |  |  |  |  |
| - ThiC | GO:0009228 | thiamin biosynthetic process | [B] |
| - ThiG | GO:0009228 | thiamin biosynthetic process | [B] |
| - Thioesterase | GO:0016788 | hydrolase activity, acting on ester bonds | [M] |
|  | GO:0009058 | biosynthetic process | [B] |
| - Thiol-ester\_cl |  |  |  |  |
| - Thr\_dehydrat\_C | GO:0004794 | L-threonine ammonia-lyase activity | [M] |
|  | GO:0009097 | isoleucine biosynthetic process | [B] |
| - Thymidylat\_synt | GO:0004799 | thymidylate synthase activity | [M] |
|  | GO:0006231 | dTMP biosynthetic process | [B] |
| - Thymopoietin |  |  |  |  |
| - TIL |  |  |  |  |
| - TIM-br\_sig\_trns |  |  |  |  |
| - Tim44 | GO:0015450 | P-P-bond-hydrolysis-driven protein transmembrane transporter activity | [M] |
|  | GO:0006886 | intracellular protein transport | [B] |
|  | GO:0005744 | mitochondrial inner membrane presequence translocase complex | [C] |
| - TLC | GO:0005471 | ATP:ADP antiporter activity | [M] |
|  | GO:0005524 | ATP binding | [M] |
|  | GO:0006810 | transport | [B] |
|  | GO:0016021 | integral to membrane | [C] |
| - TMC | GO:0016021 | integral to membrane | [C] |
| - Tmemb\_161AB |  |  |  |  |
| - Tmemb\_40 |  |  |  |  |
| - TMP-TENI | GO:0004789 | thiamin-phosphate diphosphorylase activity | [M] |
|  | GO:0009228 | thiamin biosynthetic process | [B] |
| - Tmp39 |  |  |  |  |
| - TNFR\_c6 | GO:0004872 | receptor activity | [M] |
| - TniB |  |  |  |  |
| - TOBE\_2 | GO:0005215 | transporter activity | [M] |
|  | GO:0005524 | ATP binding | [M] |
|  | GO:0016820 | hydrolase activity, acting on acid anhydrides, catalyzing transmembrane movement of substances | [M] |
|  | GO:0006810 | transport | [B] |
|  | GO:0043190 | ATP-binding cassette (ABC) transporter complex | [C] |
| - TolB\_N | GO:0015031 | protein transport | [B] |
|  | GO:0042597 | periplasmic space | [C] |
| - Tom22 | GO:0015450 | P-P-bond-hydrolysis-driven protein transmembrane transporter activity | [M] |
|  | GO:0006886 | intracellular protein transport | [B] |
|  | GO:0005741 | mitochondrial outer membrane | [C] |
| - Tom37 | GO:0006626 | protein targeting to mitochondrion | [B] |
|  | GO:0005741 | mitochondrial outer membrane | [C] |
| - Tom37\_C |  |  |  |  |
| - TonB | GO:0005381 | iron ion transmembrane transporter activity | [M] |
|  | GO:0006826 | iron ion transport | [B] |
|  | GO:0030288 | outer membrane-bounded periplasmic space | [C] |
| - Topo-VIb\_trans | GO:0003677 | DNA binding | [M] |
|  | GO:0003918 | DNA topoisomerase (ATP-hydrolyzing) activity | [M] |
|  | GO:0006265 | DNA topological change | [B] |
|  | GO:0005694 | chromosome | [C] |
| - Torsin | GO:0005524 | ATP binding | [M] |
|  | GO:0051085 | chaperone mediated protein folding requiring cofactor | [B] |
|  | GO:0005783 | endoplasmic reticulum | [C] |
| - Toxin\_1 | GO:0005576 | extracellular region | [C] |
| - TPMT | GO:0008119 | thiopurine S-methyltransferase activity | [M] |
|  | GO:0008152 | metabolic process | [B] |
|  | GO:0005737 | cytoplasm | [C] |
| - TPPII |  |  |  |  |
| - TPX2 |  |  |  |  |
| - TPX2\_importin |  |  |  |  |
| - Trans\_reg\_C | GO:0000156 | two-component response regulator activity | [M] |
|  | GO:0003677 | DNA binding | [M] |
|  | GO:0000160 | two-component signal transduction system (phosphorelay) | [B] |
|  | GO:0006355 | regulation of transcription, DNA-dependent | [B] |
| - Transferrin | GO:0008199 | ferric iron binding | [M] |
|  | GO:0006826 | iron ion transport | [B] |
|  | GO:0006879 | cellular iron ion homeostasis | [B] |
|  | GO:0005576 | extracellular region | [C] |
| - Transformer | GO:0006397 | mRNA processing | [B] |
|  | GO:0046660 | female sex differentiation | [B] |
|  | GO:0005634 | nucleus | [C] |
| - Transgly | GO:0003824 | catalytic activity | [M] |
|  | GO:0009252 | peptidoglycan biosynthetic process | [B] |
|  | GO:0009274 | peptidoglycan-based cell wall | [C] |
| - Transmemb\_17 |  |  |  |  |
| - Transp\_cyt\_pur | GO:0015205 | nucleobase transmembrane transporter activity | [M] |
|  | GO:0015931 | nucleobase, nucleoside, nucleotide and nucleic acid transport | [B] |
|  | GO:0055085 | transmembrane transport | [B] |
|  | GO:0016020 | membrane | [C] |
| - Transp\_Tc5\_C |  |  |  |  |
| - Transpeptidase | GO:0008658 | penicillin binding | [M] |
|  | GO:0009273 | peptidoglycan-based cell wall biogenesis | [B] |
| - TraU |  |  |  |  |
| - TrbI | GO:0009291 | unidirectional conjugation | [B] |
| - TRCF | GO:0003684 | damaged DNA binding | [M] |
|  | GO:0004386 | helicase activity | [M] |
|  | GO:0005524 | ATP binding | [M] |
|  | GO:0006281 | DNA repair | [B] |
| - Trefoil |  |  |  |  |
| - TRIC |  |  |  |  |
| - Trigger\_C | GO:0006457 | protein folding | [B] |
|  | GO:0015031 | protein transport | [B] |
| - Trigger\_N | GO:0006457 | protein folding | [B] |
|  | GO:0015031 | protein transport | [B] |
| - TrkA\_C | GO:0008324 | cation transmembrane transporter activity | [M] |
|  | GO:0006813 | potassium ion transport | [B] |
| - TrkA\_N | GO:0006813 | potassium ion transport | [B] |
| - TrmB |  |  |  |  |
| - tRNA-synt\_2e | GO:0000166 | nucleotide binding | [M] |
|  | GO:0004820 | glycine-tRNA ligase activity | [M] |
|  | GO:0005524 | ATP binding | [M] |
|  | GO:0006412 | translation | [B] |
|  | GO:0006426 | glycyl-tRNA aminoacylation | [B] |
|  | GO:0005737 | cytoplasm | [C] |
| - tRNA\_lig\_CPD | GO:0003972 | RNA ligase (ATP) activity | [M] |
|  | GO:0005524 | ATP binding | [M] |
|  | GO:0006388 | tRNA splicing, via endonucleolytic cleavage and ligation | [B] |
| - tRNA\_lig\_kinase | GO:0003972 | RNA ligase (ATP) activity | [M] |
|  | GO:0005524 | ATP binding | [M] |
|  | GO:0006388 | tRNA splicing, via endonucleolytic cleavage and ligation | [B] |
| - Tropomyosin |  |  |  |  |
| - TRP |  |  |  |  |
| - Trp\_syntA | GO:0004834 | tryptophan synthase activity | [M] |
|  | GO:0006568 | tryptophan metabolic process | [B] |
| - TSC22 | GO:0003700 | transcription factor activity | [M] |
|  | GO:0006355 | regulation of transcription, DNA-dependent | [B] |
| - TSP\_1 |  |  |  |  |
| - TspO\_MBR | GO:0016021 | integral to membrane | [C] |
| - Tub |  |  |  |  |
| - Turandot |  |  |  |  |
| - TylF |  |  |  |  |
| - TYW3 |  |  |  |  |
| - UbiD |  |  |  |  |
| - Ubiq-Cytc-red\_N | GO:0008121 | ubiquinol-cytochrome-c reductase activity | [M] |
|  | GO:0055114 | oxidation reduction | [B] |
| - UDPG\_MGDP\_dh | GO:0016616 | oxidoreductase activity, acting on the CH-OH group of donors, NAD or NADP as acceptor | [M] |
|  | GO:0051287 | NAD or NADH binding | [M] |
|  | GO:0055114 | oxidation reduction | [B] |
| - UDPG\_MGDP\_dh\_C | GO:0016616 | oxidoreductase activity, acting on the CH-OH group of donors, NAD or NADP as acceptor | [M] |
|  | GO:0051287 | NAD or NADH binding | [M] |
|  | GO:0055114 | oxidation reduction | [B] |
| - UDPG\_MGDP\_dh\_N | GO:0016616 | oxidoreductase activity, acting on the CH-OH group of donors, NAD or NADP as acceptor | [M] |
|  | GO:0051287 | NAD or NADH binding | [M] |
|  | GO:0055114 | oxidation reduction | [B] |
| - UIM |  |  |  |  |
| - UK |  |  |  |  |
| - UMPH-1 | GO:0000287 | magnesium ion binding | [M] |
|  | GO:0008253 | 5'-nucleotidase activity | [M] |
|  | GO:0005737 | cytoplasm | [C] |
| - UN\_NPL4 |  |  |  |  |
| - UPF0014 |  |  |  |  |
| - UPF0016 | GO:0016020 | membrane | [C] |
| - UPF0051 | GO:0005515 | protein binding | [M] |
|  | GO:0016226 | iron-sulfur cluster assembly | [B] |
| - UPF0052 |  |  |  |  |
| - UPF0054 | GO:0008237 | metallopeptidase activity | [M] |
|  | GO:0008270 | zinc ion binding | [M] |
| - UPF0061 |  |  |  |  |
| - UPF0079 |  |  |  |  |
| - UPF0089 |  |  |  |  |
| - UPF0114 |  |  |  |  |
| - UPF0126 |  |  |  |  |
| - UPF0149 |  |  |  |  |
| - UPF0193 |  |  |  |  |
| - UPF0227 |  |  |  |  |
| - UPF0240 |  |  |  |  |
| - UPF0261 |  |  |  |  |
| - UPF0561 |  |  |  |  |
| - UPF0564 |  |  |  |  |
| - UPF0565 |  |  |  |  |
| - Urease\_alpha | GO:0009039 | urease activity | [M] |
|  | GO:0016151 | nickel ion binding | [M] |
|  | GO:0019627 | urea metabolic process | [B] |
| - Urease\_beta | GO:0009039 | urease activity | [M] |
|  | GO:0016151 | nickel ion binding | [M] |
|  | GO:0006807 | nitrogen compound metabolic process | [B] |
| - Urease\_gamma | GO:0009039 | urease activity | [M] |
|  | GO:0016151 | nickel ion binding | [M] |
|  | GO:0006807 | nitrogen compound metabolic process | [B] |
| - UreD | GO:0016151 | nickel ion binding | [M] |
|  | GO:0006807 | nitrogen compound metabolic process | [B] |
| - UreF | GO:0016151 | nickel ion binding | [M] |
|  | GO:0006807 | nitrogen compound metabolic process | [B] |
| - Ureidogly\_hydro | GO:0004848 | ureidoglycolate hydrolase activity | [M] |
|  | GO:0000256 | allantoin catabolic process | [B] |
| - Uso1\_p115\_C | GO:0008565 | protein transporter activity | [M] |
|  | GO:0006886 | intracellular protein transport | [B] |
|  | GO:0005737 | cytoplasm | [C] |
|  | GO:0016020 | membrane | [C] |
| - USP7 |  |  |  |  |
| - UTRA | GO:0003677 | DNA binding | [M] |
|  | GO:0030528 | transcription regulator activity | [M] |
|  | GO:0045449 | regulation of transcription | [B] |
| - UvdE | GO:0004519 | endonuclease activity | [M] |
|  | GO:0006289 | nucleotide-excision repair | [B] |
| - UvrB |  |  |  |  |
| - UvrC\_HhH\_N | GO:0003677 | DNA binding | [M] |
|  | GO:0004518 | nuclease activity | [M] |
|  | GO:0006289 | nucleotide-excision repair | [B] |
| - UxuA | GO:0008927 | mannonate dehydratase activity | [M] |
|  | GO:0006064 | glucuronate catabolic process | [B] |
| - Val\_tRNA-synt\_C | GO:0000166 | nucleotide binding | [M] |
|  | GO:0004832 | valine-tRNA ligase activity | [M] |
|  | GO:0005524 | ATP binding | [M] |
|  | GO:0006412 | translation | [B] |
|  | GO:0006438 | valyl-tRNA aminoacylation | [B] |
|  | GO:0005737 | cytoplasm | [C] |
| - Varsurf\_PPLC | GO:0047396 | glycosylphosphatidylinositol diacylglycerol-lyase activity | [M] |
|  | GO:0006650 | glycerophospholipid metabolic process | [B] |
| - VEFS-Box |  |  |  |  |
| - VHL | GO:0016567 | protein ubiquitination | [B] |
|  | GO:0005634 | nucleus | [C] |
| - VOMI | GO:0008316 | structural constituent of vitelline membrane | [M] |
|  | GO:0030704 | vitelline membrane formation | [B] |
| - VTC |  |  |  |  |
| - VWD |  |  |  |  |
| - WAPL |  |  |  |  |
| - WBP-1 |  |  |  |  |
| - WCCH |  |  |  |  |
| - WDYHV |  |  |  |  |
| - WHEP-TRS | GO:0004812 | aminoacyl-tRNA ligase activity | [M] |
|  | GO:0005524 | ATP binding | [M] |
|  | GO:0006418 | tRNA aminoacylation for protein translation | [B] |
| - WLM |  |  |  |  |
| - Wound\_ind |  |  |  |  |
| - WWbp |  |  |  |  |
| - Wyosine\_form |  |  |  |  |
| - X8 |  |  |  |  |
| - XendoU |  |  |  |  |
| - XFP | GO:0016832 | aldehyde-lyase activity | [M] |
|  | GO:0005975 | carbohydrate metabolic process | [B] |
| - XFP\_C | GO:0016832 | aldehyde-lyase activity | [M] |
|  | GO:0005975 | carbohydrate metabolic process | [B] |
| - XFP\_N |  |  |  |  |
| - XG\_FTase | GO:0008107 | galactoside 2-alpha-L-fucosyltransferase activity | [M] |
|  | GO:0042546 | cell wall biogenesis | [B] |
|  | GO:0016020 | membrane | [C] |
| - XK-related |  |  |  |  |
| - XkdW |  |  |  |  |
| - XPA\_C | GO:0003684 | damaged DNA binding | [M] |
|  | GO:0006289 | nucleotide-excision repair | [B] |
|  | GO:0005634 | nucleus | [C] |
| - XYPPX |  |  |  |  |
| - YABBY |  |  |  |  |
| - YaeQ |  |  |  |  |
| - YccV-like |  |  |  |  |
| - YceG |  |  |  |  |
| - YceI |  |  |  |  |
| - Ycf66\_N |  |  |  |  |
| - YCII |  |  |  |  |
| - YDG\_SRA |  |  |  |  |
| - YdjC |  |  |  |  |
| - YgbB | GO:0008685 | 2-C-methyl-D-erythritol 2,4-cyclodiphosphate synthase activity | [M] |
|  | GO:0016114 | terpenoid biosynthetic process | [B] |
| - YHS |  |  |  |  |
| - YjgP\_YjgQ | GO:0016021 | integral to membrane | [C] |
| - YqeY |  |  |  |  |
| - YTH |  |  |  |  |
| - z-alpha | GO:0003723 | RNA binding | [M] |
|  | GO:0003726 | double-stranded RNA adenosine deaminase activity | [M] |
| - zf-BED | GO:0003677 | DNA binding | [M] |
| - zf-C4\_Topoisom | GO:0003677 | DNA binding | [M] |
|  | GO:0003916 | DNA topoisomerase activity | [M] |
|  | GO:0006265 | DNA topological change | [B] |
|  | GO:0005694 | chromosome | [C] |
| - zf-C5HC2 | GO:0005634 | nucleus | [C] |
| - zf-CW | GO:0008270 | zinc ion binding | [M] |
| - zf-CXXC | GO:0003677 | DNA binding | [M] |
|  | GO:0008270 | zinc ion binding | [M] |
| - zf-FPG\_IleRS | GO:0003824 | catalytic activity | [M] |
| - zf-H2C2 |  |  |  |  |
| - zf-NADH-PPase | GO:0016787 | hydrolase activity | [M] |
|  | GO:0046872 | metal ion binding | [M] |
| - zf-NPL4 |  |  |  |  |
| - zf-primase |  |  |  |  |
| - zf-RING-like |  |  |  |  |
| - zf-RNPHF |  |  |  |  |
| - zf-TAZ | GO:0003712 | transcription cofactor activity | [M] |
|  | GO:0004402 | histone acetyltransferase activity | [M] |
|  | GO:0008270 | zinc ion binding | [M] |
|  | GO:0006355 | regulation of transcription, DNA-dependent | [B] |
|  | GO:0005634 | nucleus | [C] |
| - zf-XS |  |  |  |  |
| - Zot |  |  |  |  |

---

## Annelida [eol|tol]

|  |  |  |  |
| --- | --- | --- | --- |
| **Pfam domain(s)** | **GO term acc** | **GO term** | **GO namespace** |
| - 2-Hacid\_dh | GO:0016616 | oxidoreductase activity, acting on the CH-OH group of donors, NAD or NADP as acceptor | [M] |
|  | GO:0051287 | NAD or NADH binding | [M] |
|  | GO:0008152 | metabolic process | [B] |
| - 3-PAP |  |  |  |  |
| - 7tm\_6 | GO:0004984 | olfactory receptor activity | [M] |
|  | GO:0005549 | odorant binding | [M] |
|  | GO:0007608 | sensory perception of smell | [B] |
|  | GO:0016020 | membrane | [C] |
| - A1\_Propeptide | GO:0004190 | aspartic-type endopeptidase activity | [M] |
|  | GO:0006508 | proteolysis | [B] |
| - A\_deaminase\_N | GO:0005615 | extracellular space | [C] |
| - Acetyltransf\_2 | GO:0016407 | acetyltransferase activity | [M] |
|  | GO:0008152 | metabolic process | [B] |
| - Adaptin\_binding |  |  |  |  |
| - Adeno\_IVa2 | GO:0019083 | viral transcription | [B] |
| - AIG1 | GO:0005525 | GTP binding | [M] |
| - Alpha-2-MRAP\_C | GO:0008201 | heparin binding | [M] |
|  | GO:0050750 | low-density lipoprotein receptor binding | [M] |
|  | GO:0005783 | endoplasmic reticulum | [C] |
| - Alpha-2-MRAP\_N |  |  |  |  |
| - Angiomotin\_C |  |  |  |  |
| - ARA70 |  |  |  |  |
| - BBE | GO:0016491 | oxidoreductase activity | [M] |
|  | GO:0050660 | FAD binding | [M] |
| - Bclx\_interact |  |  |  |  |
| - BH4 | GO:0042981 | regulation of apoptosis | [B] |
| - Big\_2 |  |  |  |  |
| - BrkDBD |  |  |  |  |
| - CBM\_2 | GO:0004553 | hydrolase activity, hydrolyzing O-glycosyl compounds | [M] |
|  | GO:0030246 | carbohydrate binding | [M] |
|  | GO:0005975 | carbohydrate metabolic process | [B] |
| - CBM\_6 | GO:0030246 | carbohydrate binding | [M] |
| - Chlorophyllase | GO:0047746 | chlorophyllase activity | [M] |
|  | GO:0015996 | chlorophyll catabolic process | [B] |
| - CHRD |  |  |  |  |
| - Cor1 |  |  |  |  |
| - CpeT |  |  |  |  |
| - Cu-binding\_MopE |  |  |  |  |
| - Cu\_amine\_oxid | GO:0005507 | copper ion binding | [M] |
|  | GO:0008131 | amine oxidase activity | [M] |
|  | GO:0048038 | quinone binding | [M] |
|  | GO:0009308 | amine metabolic process | [B] |
|  | GO:0055114 | oxidation reduction | [B] |
| - Cu\_amine\_oxidN2 | GO:0005507 | copper ion binding | [M] |
|  | GO:0008131 | amine oxidase activity | [M] |
|  | GO:0048038 | quinone binding | [M] |
|  | GO:0009308 | amine metabolic process | [B] |
|  | GO:0055114 | oxidation reduction | [B] |
| - Cu\_amine\_oxidN3 | GO:0005507 | copper ion binding | [M] |
|  | GO:0008131 | amine oxidase activity | [M] |
|  | GO:0048038 | quinone binding | [M] |
|  | GO:0009308 | amine metabolic process | [B] |
|  | GO:0055114 | oxidation reduction | [B] |
| - Cupin\_5 |  |  |  |  |
| - Cyclase |  |  |  |  |
| - Dsh\_C |  |  |  |  |
| - DUF1083 | GO:0004553 | hydrolase activity, hydrolyzing O-glycosyl compounds | [M] |
|  | GO:0030246 | carbohydrate binding | [M] |
|  | GO:0016052 | carbohydrate catabolic process | [B] |
| - DUF1742 |  |  |  |  |
| - DUF1758 |  |  |  |  |
| - DUF1794 |  |  |  |  |
| - DUF1838 |  |  |  |  |
| - DUF1875 |  |  |  |  |
| - DUF1903 |  |  |  |  |
| - DUF1965 |  |  |  |  |
| - DUF218 |  |  |  |  |
| - DUF2305 |  |  |  |  |
| - DUF2356 |  |  |  |  |
| - DUF2432 |  |  |  |  |
| - DUF255 |  |  |  |  |
| - DUF2668 |  |  |  |  |
| - DUF297 |  |  |  |  |
| - DUF3250 |  |  |  |  |
| - DUF3421 |  |  |  |  |
| - DUF3451 |  |  |  |  |
| - DUF3609 |  |  |  |  |
| - DUF3697 |  |  |  |  |
| - DUF3736 |  |  |  |  |
| - DUF393 |  |  |  |  |
| - DUF640 |  |  |  |  |
| - DUF917 |  |  |  |  |
| - ELH | GO:0005179 | hormone activity | [M] |
|  | GO:0007275 | multicellular organismal development | [B] |
|  | GO:0005576 | extracellular region | [C] |
| - EMI |  |  |  |  |
| - EXS | GO:0016021 | integral to membrane | [C] |
| - Extensin\_2 | GO:0005199 | structural constituent of cell wall | [M] |
|  | GO:0009664 | plant-type cell wall organization | [B] |
| - FANCF |  |  |  |  |
| - FGF-BP1 |  |  |  |  |
| - FMN\_red |  |  |  |  |
| - fn2 |  |  |  |  |
| - Fork\_head\_N |  |  |  |  |
| - FTP | GO:0004222 | metalloendopeptidase activity | [M] |
|  | GO:0008270 | zinc ion binding | [M] |
| - FtsK\_SpoIIIE | GO:0000166 | nucleotide binding | [M] |
|  | GO:0003677 | DNA binding | [M] |
|  | GO:0005524 | ATP binding | [M] |
|  | GO:0007049 | cell cycle | [B] |
|  | GO:0007059 | chromosome segregation | [B] |
|  | GO:0051301 | cell division | [B] |
|  | GO:0016021 | integral to membrane | [C] |
| - Gasdermin |  |  |  |  |
| - GlcNAc\_2-epim | GO:0004476 | mannose-6-phosphate isomerase activity | [M] |
|  | GO:0006013 | mannose metabolic process | [B] |
| - Gly\_acyl\_tr\_N |  |  |  |  |
| - Glyco\_hydro\_26 | GO:0016985 | mannan endo-1,4-beta-mannosidase activity | [M] |
|  | GO:0006080 | substituted mannan metabolic process | [B] |
| - Glyco\_hydro\_30 | GO:0004348 | glucosylceramidase activity | [M] |
|  | GO:0006665 | sphingolipid metabolic process | [B] |
|  | GO:0007040 | lysosome organization | [B] |
|  | GO:0005764 | lysosome | [C] |
| - Glyco\_hydro\_49 |  |  |  |  |
| - Glyco\_hydro\_81 | GO:0033903 | endo-1,3(4)-beta-glucanase activity | [M] |
|  | GO:0016998 | cell wall macromolecule catabolic process | [B] |
| - Glyco\_hydro\_9 | GO:0004553 | hydrolase activity, hydrolyzing O-glycosyl compounds | [M] |
|  | GO:0005975 | carbohydrate metabolic process | [B] |
| - Glyco\_hydro\_cc |  |  |  |  |
| - Grp1\_Fun34\_YaaH | GO:0016020 | membrane | [C] |
| - GSK-3\_bind |  |  |  |  |
| - H\_lectin | GO:0005515 | protein binding | [M] |
|  | GO:0005529 | sugar binding | [M] |
|  | GO:0007155 | cell adhesion | [B] |
| - HDA2-3 |  |  |  |  |
| - HIRAN | GO:0003676 | nucleic acid binding | [M] |
|  | GO:0008270 | zinc ion binding | [M] |
|  | GO:0016818 | hydrolase activity, acting on acid anhydrides, in phosphorus-containing anhydrides | [M] |
| - ICL | GO:0003824 | catalytic activity | [M] |
|  | GO:0008152 | metabolic process | [B] |
| - Ifi-6-16 |  |  |  |  |
| - L27\_2 |  |  |  |  |
| - Lys |  |  |  |  |
| - MEA1 | GO:0007283 | spermatogenesis | [B] |
| - Methyltransf\_7 | GO:0008168 | methyltransferase activity | [M] |
| - Neurexophilin |  |  |  |  |
| - NLPC\_P60 |  |  |  |  |
| - Nodulin-like |  |  |  |  |
| - NUC201 |  |  |  |  |
| - Orthoreo\_P10 |  |  |  |  |
| - P53\_TAD |  |  |  |  |
| - ParBc | GO:0003677 | DNA binding | [M] |
| - Peptidase\_M4 | GO:0004222 | metalloendopeptidase activity | [M] |
| - Peptidase\_M4\_C | GO:0004222 | metalloendopeptidase activity | [M] |
|  | GO:0006508 | proteolysis | [B] |
|  | GO:0005576 | extracellular region | [C] |
| - Peptidase\_M54 | GO:0008237 | metallopeptidase activity | [M] |
|  | GO:0008270 | zinc ion binding | [M] |
| - Pex24p |  |  |  |  |
| - Phospholip\_A2\_1 | GO:0004623 | phospholipase A2 activity | [M] |
|  | GO:0005509 | calcium ion binding | [M] |
|  | GO:0016042 | lipid catabolic process | [B] |
| - Phospholip\_A2\_3 |  |  |  |  |
| - PKI | GO:0004862 | cAMP-dependent protein kinase inhibitor activity | [M] |
|  | GO:0006469 | negative regulation of protein kinase activity | [B] |
| - PLA2\_B | GO:0004620 | phospholipase activity | [M] |
|  | GO:0009395 | phospholipid catabolic process | [B] |
| - PLA2\_inh | GO:0004859 | phospholipase inhibitor activity | [M] |
|  | GO:0005576 | extracellular region | [C] |
| - PMG |  |  |  |  |
| - Pollen\_allerg\_1 |  |  |  |  |
| - Pox\_C4\_C10 |  |  |  |  |
| - Prokineticin |  |  |  |  |
| - PSP94 |  |  |  |  |
| - PTE | GO:0008270 | zinc ion binding | [M] |
|  | GO:0016788 | hydrolase activity, acting on ester bonds | [M] |
|  | GO:0009056 | catabolic process | [B] |
| - RAG2 | GO:0003677 | DNA binding | [M] |
|  | GO:0006310 | DNA recombination | [B] |
|  | GO:0005634 | nucleus | [C] |
| - RBB1NT |  |  |  |  |
| - RBFA | GO:0006364 | rRNA processing | [B] |
| - Rho\_Binding | GO:0004674 | protein serine/threonine kinase activity | [M] |
|  | GO:0005515 | protein binding | [M] |
|  | GO:0005524 | ATP binding | [M] |
|  | GO:0000910 | cytokinesis | [B] |
|  | GO:0006468 | protein amino acid phosphorylation | [B] |
| - Rho\_N | GO:0003715 | transcription termination factor activity | [M] |
|  | GO:0006353 | transcription termination | [B] |
| - RNA\_polI\_A34 |  |  |  |  |
| - RNase\_P\_pop3 |  |  |  |  |
| - Rsm1 |  |  |  |  |
| - SelP\_N | GO:0008430 | selenium binding | [M] |
| - Senescence |  |  |  |  |
| - Sfi1 |  |  |  |  |
| - SH3\_3 |  |  |  |  |
| - Sorb |  |  |  |  |
| - SOUL |  |  |  |  |
| - SPB\_interacting |  |  |  |  |
| - Spc24 |  |  |  |  |
| - Spot\_14 |  |  |  |  |
| - STOP |  |  |  |  |
| - TAN |  |  |  |  |
| - TF\_Otx | GO:0003700 | transcription factor activity | [M] |
|  | GO:0007275 | multicellular organismal development | [B] |
|  | GO:0005634 | nucleus | [C] |
| - UK |  |  |  |  |
| - UnbV\_ASPIC |  |  |  |  |
| - UPF0547 |  |  |  |  |
| - UPF0556 |  |  |  |  |
| - UVR | GO:0003677 | DNA binding | [M] |
|  | GO:0004518 | nuclease activity | [M] |
|  | GO:0006289 | nucleotide-excision repair | [B] |
| - Y\_Y\_Y |  |  |  |  |
| - YbaK |  |  |  |  |
| - zf-C3HC | GO:0008270 | zinc ion binding | [M] |
|  | GO:0005634 | nucleus | [C] |
| - zf-FCS | GO:0008270 | zinc ion binding | [M] |

---

## Annelida\_Mollusca

|  |  |  |  |
| --- | --- | --- | --- |
| **Pfam domain(s)** | **GO term acc** | **GO term** | **GO namespace** |
| - ADK\_lid | GO:0004017 | adenylate kinase activity | [M] |
| - ATP-synt\_E | GO:0015078 | hydrogen ion transmembrane transporter activity | [M] |
|  | GO:0015986 | ATP synthesis coupled proton transport | [B] |
|  | GO:0000276 | mitochondrial proton-transporting ATP synthase complex, coupling factor F(o) | [C] |
| - CAT | GO:0008811 | chloramphenicol O-acetyltransferase activity | [M] |
| - CDT1 |  |  |  |  |
| - Dioxygenase\_C | GO:0003824 | catalytic activity | [M] |
|  | GO:0008199 | ferric iron binding | [M] |
|  | GO:0006725 | cellular aromatic compound metabolic process | [B] |
|  | GO:0055114 | oxidation reduction | [B] |
| - DNA\_ligase\_aden | GO:0003911 | DNA ligase (NAD+) activity | [M] |
| - DNA\_ligase\_OB | GO:0003911 | DNA ligase (NAD+) activity | [M] |
|  | GO:0006260 | DNA replication | [B] |
|  | GO:0006281 | DNA repair | [B] |
| - DNA\_ligase\_ZBD | GO:0003911 | DNA ligase (NAD+) activity | [M] |
|  | GO:0006260 | DNA replication | [B] |
|  | GO:0006281 | DNA repair | [B] |
| - DUF1016 |  |  |  |  |
| - DUF1181 |  |  |  |  |
| - DUF1487 |  |  |  |  |
| - DUF2423 |  |  |  |  |
| - DUF2462 |  |  |  |  |
| - DUF2763 |  |  |  |  |
| - GAGA |  |  |  |  |
| - Glyco\_hydro\_32N |  |  |  |  |
| - GSH\_synthase | GO:0004363 | glutathione synthase activity | [M] |
|  | GO:0005524 | ATP binding | [M] |
|  | GO:0006750 | glutathione biosynthetic process | [B] |
| - HTH\_IclR | GO:0003677 | DNA binding | [M] |
|  | GO:0006355 | regulation of transcription, DNA-dependent | [B] |
| - IRF-3 | GO:0003700 | transcription factor activity | [M] |
|  | GO:0006355 | regulation of transcription, DNA-dependent | [B] |
|  | GO:0005634 | nucleus | [C] |
| - Kdo | GO:0005524 | ATP binding | [M] |
|  | GO:0016773 | phosphotransferase activity, alcohol group as acceptor | [M] |
|  | GO:0009103 | lipopolysaccharide biosynthetic process | [B] |
|  | GO:0016020 | membrane | [C] |
| - Lge1 |  |  |  |  |
| - MreB\_Mbl | GO:0000902 | cell morphogenesis | [B] |
| - MRP\_L53 |  |  |  |  |
| - NB-ARC | GO:0005524 | ATP binding | [M] |
|  | GO:0006915 | apoptosis | [B] |
| - Parvo\_NS1 | GO:0019079 | viral genome replication | [B] |
| - Phasin\_2 |  |  |  |  |
| - Plasmod\_Pvs28 | GO:0009986 | cell surface | [C] |
|  | GO:0016020 | membrane | [C] |
| - Rad54\_N |  |  |  |  |
| - Rep\_3 | GO:0003887 | DNA-directed DNA polymerase activity | [M] |
|  | GO:0006270 | DNA replication initiation | [B] |
|  | GO:0005727 | extrachromosomal circular DNA | [C] |
| - RHSP |  |  |  |  |
| - Ribosomal\_L40e | GO:0003735 | structural constituent of ribosome | [M] |
|  | GO:0006412 | translation | [B] |
|  | GO:0005622 | intracellular | [C] |
|  | GO:0005840 | ribosome | [C] |
| - Ribosomal\_S27 | GO:0003735 | structural constituent of ribosome | [M] |
|  | GO:0006412 | translation | [B] |
|  | GO:0005622 | intracellular | [C] |
|  | GO:0005840 | ribosome | [C] |
| - RimK |  |  |  |  |
| - Serpentine\_r\_xa |  |  |  |  |
| - TMF\_DNA\_bd |  |  |  |  |
| - TMF\_TATA\_bd |  |  |  |  |
| - UPF1\_Zn\_bind | GO:0003677 | DNA binding | [M] |
|  | GO:0004386 | helicase activity | [M] |
|  | GO:0005524 | ATP binding | [M] |
|  | GO:0008270 | zinc ion binding | [M] |
|  | GO:0000184 | nuclear-transcribed mRNA catabolic process, nonsense-mediated decay | [B] |
|  | GO:0005737 | cytoplasm | [C] |
| - Varsurf\_PPLC | GO:0047396 | glycosylphosphatidylinositol diacylglycerol-lyase activity | [M] |
|  | GO:0006650 | glycerophospholipid metabolic process | [B] |
| - Ycf66\_N |  |  |  |  |

---

## Apicomplexa [eol|tol]

|  |  |  |  |
| --- | --- | --- | --- |
| **Pfam domain(s)** | **GO term acc** | **GO term** | **GO namespace** |
| - 2\_5\_RNA\_ligase |  |  |  |  |
| - 3Beta\_HSD | GO:0003854 | 3-beta-hydroxy-delta5-steroid dehydrogenase activity | [M] |
|  | GO:0006694 | steroid biosynthetic process | [B] |
| - 5\_nucleotid |  |  |  |  |
| - 6PF2K | GO:0003873 | 6-phosphofructo-2-kinase activity | [M] |
|  | GO:0005524 | ATP binding | [M] |
|  | GO:0006000 | fructose metabolic process | [B] |
| - 7tm\_1 | GO:0007186 | G-protein coupled receptor protein signaling pathway | [B] |
|  | GO:0016021 | integral to membrane | [C] |
| - 7tm\_2 | GO:0004930 | G-protein coupled receptor activity | [M] |
|  | GO:0007186 | G-protein coupled receptor protein signaling pathway | [B] |
|  | GO:0016020 | membrane | [C] |
| - AAA\_4 | GO:0005524 | ATP binding | [M] |
| - ABM | GO:0016491 | oxidoreductase activity | [M] |
|  | GO:0017000 | antibiotic biosynthetic process | [B] |
|  | GO:0005737 | cytoplasm | [C] |
| - Acetyltransf\_2 | GO:0016407 | acetyltransferase activity | [M] |
|  | GO:0008152 | metabolic process | [B] |
| - Act-Frag\_cataly |  |  |  |  |
| - ADC | GO:0016831 | carboxy-lyase activity | [M] |
| - ADIP |  |  |  |  |
| - AdoHcyase\_NAD |  |  |  |  |
| - AdoMet\_dc | GO:0004014 | adenosylmethionine decarboxylase activity | [M] |
|  | GO:0008295 | spermidine biosynthetic process | [B] |
| - ADP\_PFK\_GK | GO:0016773 | phosphotransferase activity, alcohol group as acceptor | [M] |
|  | GO:0005975 | carbohydrate metabolic process | [B] |
| - ADP\_ribosyl\_GH |  |  |  |  |
| - AIG1 | GO:0005525 | GTP binding | [M] |
| - Aldolase\_II | GO:0046872 | metal ion binding | [M] |
| - Aldose\_epim | GO:0016853 | isomerase activity | [M] |
|  | GO:0005975 | carbohydrate metabolic process | [B] |
| - ALO | GO:0003885 | D-arabinono-1,4-lactone oxidase activity | [M] |
|  | GO:0055114 | oxidation reduction | [B] |
|  | GO:0016020 | membrane | [C] |
| - Alpha-mann\_mid | GO:0004553 | hydrolase activity, hydrolyzing O-glycosyl compounds | [M] |
|  | GO:0008270 | zinc ion binding | [M] |
| - Alpha\_kinase | GO:0004674 | protein serine/threonine kinase activity | [M] |
|  | GO:0005524 | ATP binding | [M] |
|  | GO:0006468 | protein amino acid phosphorylation | [B] |
| - Amidinotransf | GO:0016813 | hydrolase activity, acting on carbon-nitrogen (but not peptide) bonds, in linear amidines | [M] |
|  | GO:0005737 | cytoplasm | [C] |
| - Amidohydro\_3 |  |  |  |  |
| - Ammonium\_transp | GO:0008519 | ammonium transmembrane transporter activity | [M] |
|  | GO:0006810 | transport | [B] |
|  | GO:0055085 | transmembrane transport | [B] |
|  | GO:0016020 | membrane | [C] |
| - AMPKBI |  |  |  |  |
| - ANATO | GO:0005576 | extracellular region | [C] |
| - Anoctamin |  |  |  |  |
| - ANTH | GO:0005543 | phospholipid binding | [M] |
| - APC\_CDC26 |  |  |  |  |
| - APG17 |  |  |  |  |
| - APH |  |  |  |  |
| - AraC\_E\_bind |  |  |  |  |
| - ARD | GO:0046872 | metal ion binding | [M] |
|  | GO:0051213 | dioxygenase activity | [M] |
|  | GO:0019509 | methionine salvage | [B] |
|  | GO:0055114 | oxidation reduction | [B] |
| - ArgK |  |  |  |  |
| - ARL2\_Bind\_BART |  |  |  |  |
| - ART | GO:0003956 | NAD(P)+-protein-arginine ADP-ribosyltransferase activity | [M] |
|  | GO:0006471 | protein amino acid ADP-ribosylation | [B] |
| - Asp\_Arg\_Hydrox | GO:0004597 | peptide-aspartate beta-dioxygenase activity | [M] |
|  | GO:0018193 | peptidyl-amino acid modification | [B] |
|  | GO:0055114 | oxidation reduction | [B] |
|  | GO:0030176 | integral to endoplasmic reticulum membrane | [C] |
| - Astacin | GO:0004222 | metalloendopeptidase activity | [M] |
|  | GO:0006508 | proteolysis | [B] |
| - ATE\_C | GO:0004057 | arginyltransferase activity | [M] |
|  | GO:0016598 | protein arginylation | [B] |
| - ATE\_N | GO:0004057 | arginyltransferase activity | [M] |
|  | GO:0016598 | protein arginylation | [B] |
| - ATG13 |  |  |  |  |
| - ATG27 |  |  |  |  |
| - ATP-gua\_PtransN | GO:0016301 | kinase activity | [M] |
|  | GO:0016772 | transferase activity, transferring phosphorus-containing groups | [M] |
| - ATP\_transf |  |  |  |  |
| - B\_lectin | GO:0005529 | sugar binding | [M] |
| - BAAT\_C |  |  |  |  |
| - Bac\_globin | GO:0019825 | oxygen binding | [M] |
|  | GO:0015671 | oxygen transport | [B] |
| - BAH | GO:0003677 | DNA binding | [M] |
| - Beta\_elim\_lyase | GO:0016829 | lyase activity | [M] |
|  | GO:0006520 | cellular amino acid metabolic process | [B] |
| - Beta\_propel |  |  |  |  |
| - Big\_2 |  |  |  |  |
| - BLOC1\_2 |  |  |  |  |
| - Borrelia\_orfA |  |  |  |  |
| - Bot1p |  |  |  |  |
| - BP28CT |  |  |  |  |
| - BRAP2 |  |  |  |  |
| - Bromo\_TP |  |  |  |  |
| - bZIP\_1 | GO:0003700 | transcription factor activity | [M] |
|  | GO:0043565 | sequence-specific DNA binding | [M] |
|  | GO:0046983 | protein dimerization activity | [M] |
|  | GO:0006355 | regulation of transcription, DNA-dependent | [B] |
| - bZIP\_2 | GO:0003700 | transcription factor activity | [M] |
|  | GO:0043565 | sequence-specific DNA binding | [M] |
|  | GO:0046983 | protein dimerization activity | [M] |
|  | GO:0006355 | regulation of transcription, DNA-dependent | [B] |
|  | GO:0005634 | nucleus | [C] |
| - C1\_3 |  |  |  |  |
| - Cache\_1 | GO:0016020 | membrane | [C] |
| - Caldesmon |  |  |  |  |
| - Calsequestrin | GO:0005509 | calcium ion binding | [M] |
| - CAP\_N | GO:0003779 | actin binding | [M] |
|  | GO:0007010 | cytoskeleton organization | [B] |
| - CBAH |  |  |  |  |
| - CcmE | GO:0017003 | protein-heme linkage | [B] |
|  | GO:0017004 | cytochrome complex assembly | [B] |
|  | GO:0005886 | plasma membrane | [C] |
| - CcmH |  |  |  |  |
| - CCT |  |  |  |  |
| - CD36 | GO:0007155 | cell adhesion | [B] |
|  | GO:0016020 | membrane | [C] |
| - CDO\_I | GO:0005506 | iron ion binding | [M] |
|  | GO:0017172 | cysteine dioxygenase activity | [M] |
|  | GO:0046439 | L-cysteine metabolic process | [B] |
|  | GO:0055114 | oxidation reduction | [B] |
| - Cellulase | GO:0004553 | hydrolase activity, hydrolyzing O-glycosyl compounds | [M] |
|  | GO:0005975 | carbohydrate metabolic process | [B] |
| - CG-1 | GO:0005516 | calmodulin binding | [M] |
|  | GO:0030528 | transcription regulator activity | [M] |
|  | GO:0045449 | regulation of transcription | [B] |
|  | GO:0005634 | nucleus | [C] |
| - ChaC |  |  |  |  |
| - CHAP |  |  |  |  |
| - CHD5 |  |  |  |  |
| - Chitin\_synth\_1 | GO:0004100 | chitin synthase activity | [M] |
|  | GO:0006031 | chitin biosynthetic process | [B] |
| - Chitin\_synth\_2 | GO:0016758 | transferase activity, transferring hexosyl groups | [M] |
| - Chromate\_transp | GO:0015109 | chromate transmembrane transporter activity | [M] |
|  | GO:0015703 | chromate transport | [B] |
| - CIA30 |  |  |  |  |
| - CLN3 | GO:0016020 | membrane | [C] |
| - Clusterin | GO:0008219 | cell death | [B] |
| - CoA\_trans | GO:0008410 | CoA-transferase activity | [M] |
|  | GO:0008152 | metabolic process | [B] |
| - CoA\_transf\_3 | GO:0003824 | catalytic activity | [M] |
|  | GO:0008152 | metabolic process | [B] |
| - CobW\_C |  |  |  |  |
| - Complex1\_24kDa | GO:0016491 | oxidoreductase activity | [M] |
|  | GO:0051287 | NAD or NADH binding | [M] |
|  | GO:0055114 | oxidation reduction | [B] |
| - Complex1\_51K | GO:0010181 | FMN binding | [M] |
|  | GO:0016651 | oxidoreductase activity, acting on NADH or NADPH | [M] |
|  | GO:0051287 | NAD or NADH binding | [M] |
|  | GO:0051539 | 4 iron, 4 sulfur cluster binding | [M] |
| - Complex1\_LYR |  |  |  |  |
| - Cons\_hypoth698 | GO:0016021 | integral to membrane | [C] |
| - Copine |  |  |  |  |
| - Coprinus\_mating | GO:0003677 | DNA binding | [M] |
|  | GO:0045449 | regulation of transcription | [B] |
|  | GO:0005634 | nucleus | [C] |
| - COQ9 |  |  |  |  |
| - CPL | GO:0003723 | RNA binding | [M] |
| - CRT10 |  |  |  |  |
| - Crystall |  |  |  |  |
| - CUE |  |  |  |  |
| - Cupin\_5 |  |  |  |  |
| - CutC | GO:0005507 | copper ion binding | [M] |
|  | GO:0055070 | copper ion homeostasis | [B] |
| - Cutinase | GO:0016787 | hydrolase activity | [M] |
|  | GO:0008152 | metabolic process | [B] |
| - CXC |  |  |  |  |
| - Cystatin | GO:0004869 | cysteine-type endopeptidase inhibitor activity | [M] |
| - Cytochrom\_B561 | GO:0016021 | integral to membrane | [C] |
| - Cytokin\_check\_N |  |  |  |  |
| - Dak1 | GO:0004371 | glycerone kinase activity | [M] |
|  | GO:0006071 | glycerol metabolic process | [B] |
| - Dak2 | GO:0004371 | glycerone kinase activity | [M] |
|  | GO:0006071 | glycerol metabolic process | [B] |
| - DAP\_epimerase | GO:0008837 | diaminopimelate epimerase activity | [M] |
|  | GO:0009089 | lysine biosynthetic process via diaminopimelate | [B] |
|  | GO:0005737 | cytoplasm | [C] |
| - DcpS | GO:0005515 | protein binding | [M] |
|  | GO:0016787 | hydrolase activity | [M] |
|  | GO:0000290 | deadenylation-dependent decapping of nuclear-transcribed mRNA | [B] |
| - DDE | GO:0003676 | nucleic acid binding | [M] |
| - dDENN |  |  |  |  |
| - DDT |  |  |  |  |
| - DENN |  |  |  |  |
| - DHHA2 | GO:0016462 | pyrophosphatase activity | [M] |
|  | GO:0005737 | cytoplasm | [C] |
| - Di19 |  |  |  |  |
| - Dickkopf\_N | GO:0007275 | multicellular organismal development | [B] |
|  | GO:0030178 | negative regulation of Wnt receptor signaling pathway | [B] |
|  | GO:0005576 | extracellular region | [C] |
| - Dicty\_CAR | GO:0004930 | G-protein coupled receptor activity | [M] |
|  | GO:0030552 | cAMP binding | [M] |
|  | GO:0007186 | G-protein coupled receptor protein signaling pathway | [B] |
|  | GO:0016021 | integral to membrane | [C] |
| - DinB |  |  |  |  |
| - DIRP |  |  |  |  |
| - DLH | GO:0016787 | hydrolase activity | [M] |
| - Dna2 | GO:0003677 | DNA binding | [M] |
|  | GO:0004003 | ATP-dependent DNA helicase activity | [M] |
|  | GO:0005524 | ATP binding | [M] |
|  | GO:0006260 | DNA replication | [B] |
| - DNA\_alkylation |  |  |  |  |
| - DNA\_ligase\_IV |  |  |  |  |
| - DNA\_pol\_B\_2 | GO:0000166 | nucleotide binding | [M] |
|  | GO:0003677 | DNA binding | [M] |
|  | GO:0003887 | DNA-directed DNA polymerase activity | [M] |
|  | GO:0008408 | 3'-5' exonuclease activity | [M] |
|  | GO:0006260 | DNA replication | [B] |
| - DNA\_pol\_lambd\_f |  |  |  |  |
| - DNase\_II | GO:0004531 | deoxyribonuclease II activity | [M] |
|  | GO:0006259 | DNA metabolic process | [B] |
| - dNK | GO:0005524 | ATP binding | [M] |
|  | GO:0016773 | phosphotransferase activity, alcohol group as acceptor | [M] |
|  | GO:0006139 | nucleobase, nucleoside, nucleotide and nucleic acid metabolic process | [B] |
| - DOMON | GO:0004500 | dopamine beta-monooxygenase activity | [M] |
|  | GO:0006548 | histidine catabolic process | [B] |
| - DOPA\_dioxygen |  |  |  |  |
| - DoxX |  |  |  |  |
| - DP |  |  |  |  |
| - DPM2 | GO:0009059 | macromolecule biosynthetic process | [B] |
|  | GO:0030176 | integral to endoplasmic reticulum membrane | [C] |
| - Drf\_GBD | GO:0003779 | actin binding | [M] |
|  | GO:0017048 | Rho GTPase binding | [M] |
|  | GO:0030036 | actin cytoskeleton organization | [B] |
| - dsrm | GO:0003725 | double-stranded RNA binding | [M] |
|  | GO:0005622 | intracellular | [C] |
| - dsRNA\_bind |  |  |  |  |
| - DUF1023 |  |  |  |  |
| - DUF1083 | GO:0004553 | hydrolase activity, hydrolyzing O-glycosyl compounds | [M] |
|  | GO:0030246 | carbohydrate binding | [M] |
|  | GO:0016052 | carbohydrate catabolic process | [B] |
| - DUF1087 |  |  |  |  |
| - DUF1216 |  |  |  |  |
| - DUF1232 |  |  |  |  |
| - DUF1237 |  |  |  |  |
| - DUF124 |  |  |  |  |
| - DUF1294 |  |  |  |  |
| - DUF1295 |  |  |  |  |
| - DUF1309 |  |  |  |  |
| - DUF1343 |  |  |  |  |
| - DUF1348 |  |  |  |  |
| - DUF1349 |  |  |  |  |
| - DUF1448 |  |  |  |  |
| - DUF1517 |  |  |  |  |
| - DUF1565 |  |  |  |  |
| - DUF1619 |  |  |  |  |
| - DUF1624 |  |  |  |  |
| - DUF1630 |  |  |  |  |
| - DUF1631 |  |  |  |  |
| - DUF1632 |  |  |  |  |
| - DUF1637 | GO:0047800 | cysteamine dioxygenase activity | [M] |
|  | GO:0055114 | oxidation reduction | [B] |
| - DUF1649 |  |  |  |  |
| - DUF1683 |  |  |  |  |
| - DUF1740 |  |  |  |  |
| - DUF1757 |  |  |  |  |
| - DUF1772 |  |  |  |  |
| - DUF1794 |  |  |  |  |
| - DUF185 |  |  |  |  |
| - DUF19 |  |  |  |  |
| - DUF1969 | GO:0004334 | fumarylacetoacetase activity | [M] |
|  | GO:0009072 | aromatic amino acid family metabolic process | [B] |
| - DUF1982 | GO:0016651 | oxidoreductase activity, acting on NADH or NADPH | [M] |
|  | GO:0051536 | iron-sulfur cluster binding | [M] |
|  | GO:0055114 | oxidation reduction | [B] |
| - DUF2010 |  |  |  |  |
| - DUF2013 |  |  |  |  |
| - DUF2039 |  |  |  |  |
| - DUF2051 |  |  |  |  |
| - DUF218 |  |  |  |  |
| - DUF2237 |  |  |  |  |
| - DUF2257 |  |  |  |  |
| - DUF229 |  |  |  |  |
| - DUF2305 |  |  |  |  |
| - DUF2306 |  |  |  |  |
| - DUF2348 |  |  |  |  |
| - DUF2356 |  |  |  |  |
| - DUF2360 |  |  |  |  |
| - DUF2363 |  |  |  |  |
| - DUF2368 |  |  |  |  |
| - DUF2404 |  |  |  |  |
| - DUF2414 |  |  |  |  |
| - DUF2419 |  |  |  |  |
| - DUF2428 |  |  |  |  |
| - DUF2432 |  |  |  |  |
| - DUF2451 |  |  |  |  |
| - DUF2475 |  |  |  |  |
| - DUF258 | GO:0003924 | GTPase activity | [M] |
|  | GO:0005525 | GTP binding | [M] |
| - DUF2615 |  |  |  |  |
| - DUF2778 |  |  |  |  |
| - DUF2804 |  |  |  |  |
| - DUF296 |  |  |  |  |
| - DUF297 |  |  |  |  |
| - DUF3250 |  |  |  |  |
| - DUF3385 |  |  |  |  |
| - DUF3402 |  |  |  |  |
| - DUF3424 |  |  |  |  |
| - DUF3429 |  |  |  |  |
| - DUF3452 |  |  |  |  |
| - DUF3472 |  |  |  |  |
| - DUF3508 |  |  |  |  |
| - DUF3595 |  |  |  |  |
| - DUF393 |  |  |  |  |
| - DUF395 |  |  |  |  |
| - DUF423 |  |  |  |  |
| - DUF455 |  |  |  |  |
| - DUF498 |  |  |  |  |
| - DUF525 |  |  |  |  |
| - DUF543 |  |  |  |  |
| - DUF582 |  |  |  |  |
| - DUF607 |  |  |  |  |
| - DUF727 |  |  |  |  |
| - DUF812 |  |  |  |  |
| - DUF815 |  |  |  |  |
| - DUF82 |  |  |  |  |
| - DUF833 |  |  |  |  |
| - DUF836 |  |  |  |  |
| - DUF837 |  |  |  |  |
| - DUF847 |  |  |  |  |
| - DUF912 |  |  |  |  |
| - DUF917 |  |  |  |  |
| - DUF924 |  |  |  |  |
| - DUF953 |  |  |  |  |
| - DUF962 |  |  |  |  |
| - DUF98 |  |  |  |  |
| - Dyp\_perox |  |  |  |  |
| - EBP | GO:0047750 | cholestenol delta-isomerase activity | [M] |
|  | GO:0016125 | sterol metabolic process | [B] |
|  | GO:0005783 | endoplasmic reticulum | [C] |
|  | GO:0016021 | integral to membrane | [C] |
| - EF-1\_beta\_acid |  |  |  |  |
| - EF\_assoc\_1 |  |  |  |  |
| - EF\_assoc\_2 |  |  |  |  |
| - Endonuclease\_5 | GO:0004519 | endonuclease activity | [M] |
|  | GO:0006281 | DNA repair | [B] |
| - Endonuclease\_7 | GO:0004519 | endonuclease activity | [M] |
| - Erf4 |  |  |  |  |
| - ERG4\_ERG24 | GO:0016020 | membrane | [C] |
| - Esterase |  |  |  |  |
| - Esterase\_phd | GO:0005576 | extracellular region | [C] |
| - ETC\_C1\_NDUFA4 | GO:0016651 | oxidoreductase activity, acting on NADH or NADPH | [M] |
|  | GO:0022900 | electron transport chain | [B] |
|  | GO:0005743 | mitochondrial inner membrane | [C] |
| - ETC\_C1\_NDUFA5 | GO:0016651 | oxidoreductase activity, acting on NADH or NADPH | [M] |
|  | GO:0022904 | respiratory electron transport chain | [B] |
|  | GO:0005743 | mitochondrial inner membrane | [C] |
| - FAA\_hydrolase | GO:0003824 | catalytic activity | [M] |
|  | GO:0008152 | metabolic process | [B] |
| - FAD-oxidase\_C | GO:0003824 | catalytic activity | [M] |
|  | GO:0050660 | FAD binding | [M] |
| - FAD\_binding\_4 | GO:0016491 | oxidoreductase activity | [M] |
|  | GO:0050660 | FAD binding | [M] |
| - FAD\_binding\_8 |  |  |  |  |
| - Far-17a\_AIG1 | GO:0016021 | integral to membrane | [C] |
| - FERM\_M |  |  |  |  |
| - Ferritin | GO:0008199 | ferric iron binding | [M] |
|  | GO:0006879 | cellular iron ion homeostasis | [B] |
| - Fes1 |  |  |  |  |
| - FGase |  |  |  |  |
| - FMN\_dh | GO:0016491 | oxidoreductase activity | [M] |
| - FMO-like | GO:0004499 | flavin-containing monooxygenase activity | [M] |
|  | GO:0050660 | FAD binding | [M] |
|  | GO:0050661 | NADP or NADPH binding | [M] |
|  | GO:0055114 | oxidation reduction | [B] |
| - Fmp27\_GFWDK |  |  |  |  |
| - FOP\_dimer | GO:0034453 | microtubule anchoring | [B] |
|  | GO:0005813 | centrosome | [C] |
| - Frag1 |  |  |  |  |
| - FRG1 |  |  |  |  |
| - FtsX | GO:0016020 | membrane | [C] |
| - FumaraseC\_C | GO:0016829 | lyase activity | [M] |
|  | GO:0006099 | tricarboxylic acid cycle | [B] |
| - FYRC | GO:0003677 | DNA binding | [M] |
|  | GO:0005634 | nucleus | [C] |
| - FYRN | GO:0003677 | DNA binding | [M] |
|  | GO:0005634 | nucleus | [C] |
| - Fz |  |  |  |  |
| - G8 |  |  |  |  |
| - GAGA\_bind |  |  |  |  |
| - GAT | GO:0006886 | intracellular protein transport | [B] |
|  | GO:0005622 | intracellular | [C] |
| - GCN5L1 |  |  |  |  |
| - GDC-P |  |  |  |  |
| - GFA | GO:0016846 | carbon-sulfur lyase activity | [M] |
|  | GO:0008152 | metabolic process | [B] |
| - Git3 |  |  |  |  |
| - Git3\_C |  |  |  |  |
| - Glucokinase | GO:0004340 | glucokinase activity | [M] |
|  | GO:0005524 | ATP binding | [M] |
|  | GO:0006096 | glycolysis | [B] |
| - Glyco\_hydro\_16 | GO:0004553 | hydrolase activity, hydrolyzing O-glycosyl compounds | [M] |
|  | GO:0005975 | carbohydrate metabolic process | [B] |
| - Glyco\_hydro\_2 | GO:0004553 | hydrolase activity, hydrolyzing O-glycosyl compounds | [M] |
|  | GO:0005975 | carbohydrate metabolic process | [B] |
| - Glyco\_hydro\_20 | GO:0004553 | hydrolase activity, hydrolyzing O-glycosyl compounds | [M] |
| - Glyco\_hydro\_20b | GO:0004563 | beta-N-acetylhexosaminidase activity | [M] |
|  | GO:0005975 | carbohydrate metabolic process | [B] |
| - Glyco\_hydro\_25 | GO:0003796 | lysozyme activity | [M] |
|  | GO:0009253 | peptidoglycan catabolic process | [B] |
|  | GO:0016998 | cell wall macromolecule catabolic process | [B] |
| - Glyco\_hydro\_2\_N | GO:0004553 | hydrolase activity, hydrolyzing O-glycosyl compounds | [M] |
|  | GO:0005975 | carbohydrate metabolic process | [B] |
| - Glyco\_hydro\_3 | GO:0004553 | hydrolase activity, hydrolyzing O-glycosyl compounds | [M] |
|  | GO:0005975 | carbohydrate metabolic process | [B] |
| - Glyco\_hydro\_38 | GO:0004559 | alpha-mannosidase activity | [M] |
|  | GO:0005975 | carbohydrate metabolic process | [B] |
| - Glyco\_hydro\_38C | GO:0015923 | mannosidase activity | [M] |
|  | GO:0006013 | mannose metabolic process | [B] |
| - Glyco\_hydro\_3\_C | GO:0004553 | hydrolase activity, hydrolyzing O-glycosyl compounds | [M] |
|  | GO:0005975 | carbohydrate metabolic process | [B] |
| - Glyco\_hydro\_45 | GO:0008810 | cellulase activity | [M] |
|  | GO:0005975 | carbohydrate metabolic process | [B] |
| - Glyco\_hydro\_76 |  |  |  |  |
| - Glyco\_hydro\_85 | GO:0033925 | mannosyl-glycoprotein endo-beta-N-acetylglucosaminidase activity | [M] |
|  | GO:0005737 | cytoplasm | [C] |
| - Glycos\_trans\_3N |  |  |  |  |
| - Glycos\_transf\_3 | GO:0016757 | transferase activity, transferring glycosyl groups | [M] |
|  | GO:0008152 | metabolic process | [B] |
| - GMP\_PDE\_delta |  |  |  |  |
| - GPS | GO:0007218 | neuropeptide signaling pathway | [B] |
|  | GO:0016020 | membrane | [C] |
| - GRAB |  |  |  |  |
| - GSP\_synth |  |  |  |  |
| - GSPII\_E | GO:0005524 | ATP binding | [M] |
|  | GO:0006810 | transport | [B] |
|  | GO:0005622 | intracellular | [C] |
| - H\_lectin | GO:0005515 | protein binding | [M] |
|  | GO:0005529 | sugar binding | [M] |
|  | GO:0007155 | cell adhesion | [B] |
| - HAMP | GO:0004871 | signal transducer activity | [M] |
|  | GO:0007165 | signal transduction | [B] |
|  | GO:0016021 | integral to membrane | [C] |
| - Hap2\_elong |  |  |  |  |
| - hATC | GO:0046983 | protein dimerization activity | [M] |
| - HCaRG |  |  |  |  |
| - HEAT |  |  |  |  |
| - HELP |  |  |  |  |
| - HemN\_C | GO:0004109 | coproporphyrinogen oxidase activity | [M] |
|  | GO:0006779 | porphyrin biosynthetic process | [B] |
|  | GO:0055114 | oxidation reduction | [B] |
|  | GO:0005737 | cytoplasm | [C] |
| - HgmA | GO:0004411 | homogentisate 1,2-dioxygenase activity | [M] |
|  | GO:0006559 | L-phenylalanine catabolic process | [B] |
|  | GO:0006570 | tyrosine metabolic process | [B] |
|  | GO:0055114 | oxidation reduction | [B] |
| - HIN |  |  |  |  |
| - Hira | GO:0030528 | transcription regulator activity | [M] |
|  | GO:0045449 | regulation of transcription | [B] |
|  | GO:0005634 | nucleus | [C] |
| - HIRAN | GO:0003676 | nucleic acid binding | [M] |
|  | GO:0008270 | zinc ion binding | [M] |
|  | GO:0016818 | hydrolase activity, acting on acid anhydrides, in phosphorus-containing anhydrides | [M] |
| - HMG-CoA\_red | GO:0004420 | hydroxymethylglutaryl-CoA reductase (NADPH) activity | [M] |
|  | GO:0050662 | coenzyme binding | [M] |
|  | GO:0015936 | coenzyme A metabolic process | [B] |
|  | GO:0055114 | oxidation reduction | [B] |
| - HMG\_CoA\_synt\_C | GO:0004421 | hydroxymethylglutaryl-CoA synthase activity | [M] |
|  | GO:0008299 | isoprenoid biosynthetic process | [B] |
| - HMG\_CoA\_synt\_N | GO:0004421 | hydroxymethylglutaryl-CoA synthase activity | [M] |
|  | GO:0008299 | isoprenoid biosynthetic process | [B] |
| - Homeobox | GO:0003700 | transcription factor activity | [M] |
|  | GO:0043565 | sequence-specific DNA binding | [M] |
|  | GO:0006355 | regulation of transcription, DNA-dependent | [B] |
| - HSF\_DNA-bind | GO:0003700 | transcription factor activity | [M] |
|  | GO:0043565 | sequence-specific DNA binding | [M] |
|  | GO:0006355 | regulation of transcription, DNA-dependent | [B] |
|  | GO:0005634 | nucleus | [C] |
| - HSP33 | GO:0051082 | unfolded protein binding | [M] |
|  | GO:0006457 | protein folding | [B] |
|  | GO:0005737 | cytoplasm | [C] |
| - HTH\_5 | GO:0003700 | transcription factor activity | [M] |
|  | GO:0006355 | regulation of transcription, DNA-dependent | [B] |
|  | GO:0005622 | intracellular | [C] |
| - HTH\_psq | GO:0003677 | DNA binding | [M] |
| - Hydant\_A\_N |  |  |  |  |
| - Hydantoinase\_A | GO:0016787 | hydrolase activity | [M] |
| - Hydantoinase\_B | GO:0003824 | catalytic activity | [M] |
| - IDO | GO:0020037 | heme binding | [M] |
| - IFT46\_B\_C |  |  |  |  |
| - IFT57 |  |  |  |  |
| - IGR |  |  |  |  |
| - IKI3 |  |  |  |  |
| - IMD | GO:0008093 | cytoskeletal adaptor activity | [M] |
|  | GO:0017124 | SH3 domain binding | [M] |
|  | GO:0007165 | signal transduction | [B] |
|  | GO:0046847 | filopodium assembly | [B] |
| - IML2 |  |  |  |  |
| - IMS\_HHH |  |  |  |  |
| - Indigoidine\_A |  |  |  |  |
| - Ins134\_P3\_kin | GO:0000287 | magnesium ion binding | [M] |
|  | GO:0005524 | ATP binding | [M] |
|  | GO:0035300 | inositol-1,3,4-trisphosphate 5/6-kinase activity | [M] |
|  | GO:0047325 | inositol tetrakisphosphate 1-kinase activity | [M] |
|  | GO:0032957 | inositol trisphosphate metabolic process | [B] |
|  | GO:0005622 | intracellular | [C] |
| - Ins145\_P3\_rec |  |  |  |  |
| - Ion\_trans\_N |  |  |  |  |
| - IpgD | GO:0016791 | phosphatase activity | [M] |
|  | GO:0009405 | pathogenesis | [B] |
| - IU\_nuc\_hydro |  |  |  |  |
| - Jacalin |  |  |  |  |
| - K\_trans | GO:0015079 | potassium ion transmembrane transporter activity | [M] |
|  | GO:0006813 | potassium ion transport | [B] |
|  | GO:0016020 | membrane | [C] |
| - KA1 | GO:0004674 | protein serine/threonine kinase activity | [M] |
|  | GO:0005524 | ATP binding | [M] |
|  | GO:0006468 | protein amino acid phosphorylation | [B] |
| - KAP | GO:0019894 | kinesin binding | [M] |
|  | GO:0005871 | kinesin complex | [C] |
| - KOG2701 |  |  |  |  |
| - Ku\_C | GO:0003677 | DNA binding | [M] |
|  | GO:0004003 | ATP-dependent DNA helicase activity | [M] |
|  | GO:0006303 | double-strand break repair via nonhomologous end joining | [B] |
|  | GO:0005634 | nucleus | [C] |
| - LacAB\_rpiB | GO:0005975 | carbohydrate metabolic process | [B] |
| - LacY\_symp | GO:0006810 | transport | [B] |
|  | GO:0016020 | membrane | [C] |
| - LBP\_BPI\_CETP\_C | GO:0008289 | lipid binding | [M] |
| - Leuk-A4-hydro\_C | GO:0008237 | metallopeptidase activity | [M] |
|  | GO:0008270 | zinc ion binding | [M] |
|  | GO:0019370 | leukotriene biosynthetic process | [B] |
| - Lig\_chan | GO:0004970 | ionotropic glutamate receptor activity | [M] |
|  | GO:0005234 | extracellular-glutamate-gated ion channel activity | [M] |
|  | GO:0016020 | membrane | [C] |
| - LigB | GO:0008198 | ferrous iron binding | [M] |
|  | GO:0016491 | oxidoreductase activity | [M] |
|  | GO:0006725 | cellular aromatic compound metabolic process | [B] |
| - LIM | GO:0008270 | zinc ion binding | [M] |
| - Lipase\_2 |  |  |  |  |
| - Lipase\_GDSL | GO:0016788 | hydrolase activity, acting on ester bonds | [M] |
|  | GO:0006629 | lipid metabolic process | [B] |
| - Lipocalin\_2 |  |  |  |  |
| - LpxB | GO:0008915 | lipid-A-disaccharide synthase activity | [M] |
|  | GO:0009245 | lipid A biosynthetic process | [B] |
| - LVIVD |  |  |  |  |
| - LysM | GO:0016998 | cell wall macromolecule catabolic process | [B] |
| - LytTR |  |  |  |  |
| - M20\_dimer | GO:0016787 | hydrolase activity | [M] |
|  | GO:0046983 | protein dimerization activity | [M] |
| - Mad3\_BUB1\_I |  |  |  |  |
| - Malate\_synthase | GO:0003824 | catalytic activity | [M] |
| - MAP65\_ASE1 |  |  |  |  |
| - MAPKK1\_Int |  |  |  |  |
| - MazG |  |  |  |  |
| - MBT | GO:0045449 | regulation of transcription | [B] |
|  | GO:0005634 | nucleus | [C] |
| - Meckelin |  |  |  |  |
| - Med1 |  |  |  |  |
| - Med25\_VWA |  |  |  |  |
| - Mei5 |  |  |  |  |
| - Mem\_trans | GO:0055085 | transmembrane transport | [B] |
|  | GO:0016021 | integral to membrane | [C] |
| - Metalloenzyme | GO:0003824 | catalytic activity | [M] |
|  | GO:0046872 | metal ion binding | [M] |
| - MIG-14\_Wnt-bd |  |  |  |  |
| - MIR | GO:0016020 | membrane | [C] |
| - Mlf1IP |  |  |  |  |
| - Mlo | GO:0008219 | cell death | [B] |
|  | GO:0016021 | integral to membrane | [C] |
| - MMS1 |  |  |  |  |
| - Mod\_r |  |  |  |  |
| - Molybdopterin | GO:0016491 | oxidoreductase activity | [M] |
| - Mt\_ATP-synt\_D | GO:0015078 | hydrogen ion transmembrane transporter activity | [M] |
|  | GO:0015986 | ATP synthesis coupled proton transport | [B] |
|  | GO:0000276 | mitochondrial proton-transporting ATP synthase complex, coupling factor F(o) | [C] |
| - Muc\_lac\_enz |  |  |  |  |
| - MVP\_shoulder |  |  |  |  |
| - Myosin\_TH1 | GO:0003774 | motor activity | [M] |
|  | GO:0016459 | myosin complex | [C] |
| - N6-adenineMlase |  |  |  |  |
| - NAcGluc\_Transf |  |  |  |  |
| - NACHT |  |  |  |  |
| - NAD\_binding\_6 |  |  |  |  |
| - NADH-G\_4Fe-4S\_3 |  |  |  |  |
| - NADH\_4Fe-4S |  |  |  |  |
| - NAGLU |  |  |  |  |
| - NB-ARC | GO:0005524 | ATP binding | [M] |
|  | GO:0006915 | apoptosis | [B] |
| - NDUFA12 | GO:0008137 | NADH dehydrogenase (ubiquinone) activity | [M] |
|  | GO:0009055 | electron carrier activity | [M] |
|  | GO:0016020 | membrane | [C] |
| - Nfu\_N | GO:0005506 | iron ion binding | [M] |
| - NLPC\_P60 |  |  |  |  |
| - NmrA | GO:0016564 | transcription repressor activity | [M] |
|  | GO:0006808 | regulation of nitrogen utilization | [B] |
| - NPD | GO:0018580 | 2-nitropropane dioxygenase activity | [M] |
|  | GO:0055114 | oxidation reduction | [B] |
| - Nuc-transf |  |  |  |  |
| - NUC194 | GO:0003677 | DNA binding | [M] |
|  | GO:0004677 | DNA-dependent protein kinase activity | [M] |
|  | GO:0005524 | ATP binding | [M] |
|  | GO:0006303 | double-strand break repair via nonhomologous end joining | [B] |
|  | GO:0005634 | nucleus | [C] |
| - Nuc\_deoxyrib\_tr |  |  |  |  |
| - Nucleopor\_Nup85 |  |  |  |  |
| - Nucleoporin\_N |  |  |  |  |
| - NUMOD4 | GO:0016788 | hydrolase activity, acting on ester bonds | [M] |
| - ODC\_AZ | GO:0004857 | enzyme inhibitor activity | [M] |
|  | GO:0008073 | ornithine decarboxylase inhibitor activity | [M] |
| - OGFr\_N | GO:0004872 | receptor activity | [M] |
|  | GO:0016020 | membrane | [C] |
| - OLF |  |  |  |  |
| - Oscp1 |  |  |  |  |
| - OsmC | GO:0006950 | response to stress | [B] |
| - Ost4 |  |  |  |  |
| - Oxidored-like |  |  |  |  |
| - Oxidored\_FMN | GO:0010181 | FMN binding | [M] |
|  | GO:0016491 | oxidoreductase activity | [M] |
| - P16-Arc | GO:0030833 | regulation of actin filament polymerization | [B] |
|  | GO:0005856 | cytoskeleton | [C] |
| - P21-Arc | GO:0030833 | regulation of actin filament polymerization | [B] |
|  | GO:0005856 | cytoskeleton | [C] |
| - PAC2 |  |  |  |  |
| - PAD\_porph |  |  |  |  |
| - PAE |  |  |  |  |
| - PAF-AH\_p\_II | GO:0003847 | 1-alkyl-2-acetylglycerophosphocholine esterase activity | [M] |
|  | GO:0016042 | lipid catabolic process | [B] |
| - Palm\_thioest | GO:0008474 | palmitoyl-(protein) hydrolase activity | [M] |
|  | GO:0006464 | protein modification process | [B] |
| - PAPA-1 |  |  |  |  |
| - Paramecium\_SA |  |  |  |  |
| - PARP\_regulatory | GO:0004483 | mRNA (nucleoside-2'-O-)-methyltransferase activity | [M] |
|  | GO:0006370 | mRNA capping | [B] |
|  | GO:0006397 | mRNA processing | [B] |
| - PAXNEB | GO:0006357 | regulation of transcription from RNA polymerase II promoter | [B] |
|  | GO:0033588 | Elongator holoenzyme complex | [C] |
| - PAZ |  |  |  |  |
| - PB1 |  |  |  |  |
| - PBD |  |  |  |  |
| - PC4 | GO:0003677 | DNA binding | [M] |
|  | GO:0003713 | transcription coactivator activity | [M] |
|  | GO:0006355 | regulation of transcription, DNA-dependent | [B] |
| - Pcc1 |  |  |  |  |
| - Pecanex\_C | GO:0016021 | integral to membrane | [C] |
| - Pedibin |  |  |  |  |
| - PEP-utilizers | GO:0016772 | transferase activity, transferring phosphorus-containing groups | [M] |
|  | GO:0016310 | phosphorylation | [B] |
| - PEP-utilizers\_C | GO:0016772 | transferase activity, transferring phosphorus-containing groups | [M] |
|  | GO:0016310 | phosphorylation | [B] |
| - Peptidase\_C15 | GO:0006508 | proteolysis | [B] |
| - Peptidase\_M10 | GO:0004222 | metalloendopeptidase activity | [M] |
|  | GO:0006508 | proteolysis | [B] |
|  | GO:0005578 | proteinaceous extracellular matrix | [C] |
| - Peptidase\_M15 | GO:0008237 | metallopeptidase activity | [M] |
|  | GO:0016805 | dipeptidase activity | [M] |
|  | GO:0006508 | proteolysis | [B] |
|  | GO:0005618 | cell wall | [C] |
| - Peptidase\_M20 | GO:0008237 | metallopeptidase activity | [M] |
|  | GO:0006508 | proteolysis | [B] |
| - Peptidase\_M49 | GO:0008239 | dipeptidyl-peptidase activity | [M] |
|  | GO:0006508 | proteolysis | [B] |
|  | GO:0005737 | cytoplasm | [C] |
| - Peptidase\_M8 | GO:0004222 | metalloendopeptidase activity | [M] |
|  | GO:0008270 | zinc ion binding | [M] |
|  | GO:0006508 | proteolysis | [B] |
|  | GO:0007155 | cell adhesion | [B] |
|  | GO:0016020 | membrane | [C] |
| - Peptidase\_S10 | GO:0004185 | serine-type carboxypeptidase activity | [M] |
|  | GO:0006508 | proteolysis | [B] |
| - Peptidase\_S11 | GO:0009002 | serine-type D-Ala-D-Ala carboxypeptidase activity | [M] |
|  | GO:0006508 | proteolysis | [B] |
| - Peptidase\_S49 | GO:0008233 | peptidase activity | [M] |
|  | GO:0006508 | proteolysis | [B] |
| - peroxidase | GO:0004601 | peroxidase activity | [M] |
|  | GO:0020037 | heme binding | [M] |
|  | GO:0006979 | response to oxidative stress | [B] |
|  | GO:0055114 | oxidation reduction | [B] |
| - Pet127 |  |  |  |  |
| - Pex14\_N | GO:0005777 | peroxisome | [C] |
|  | GO:0016020 | membrane | [C] |
| - Pex19 | GO:0005777 | peroxisome | [C] |
| - Pex24p |  |  |  |  |
| - PG\_binding\_1 | GO:0008152 | metabolic process | [B] |
| - PhoH | GO:0005524 | ATP binding | [M] |
| - PhosphMutase |  |  |  |  |
| - Phosphoesterase | GO:0016788 | hydrolase activity, acting on ester bonds | [M] |
| - Phospholip\_B |  |  |  |  |
| - PHR |  |  |  |  |
| - Phytochelatin | GO:0016756 | glutathione gamma-glutamylcysteinyltransferase activity | [M] |
|  | GO:0046872 | metal ion binding | [M] |
|  | GO:0010038 | response to metal ion | [B] |
|  | GO:0046938 | phytochelatin biosynthetic process | [B] |
| - PI3K\_rbd | GO:0016303 | 1-phosphatidylinositol-3-kinase activity | [M] |
|  | GO:0005942 | phosphoinositide 3-kinase complex | [C] |
| - PIG-X | GO:0006506 | GPI anchor biosynthetic process | [B] |
|  | GO:0005789 | endoplasmic reticulum membrane | [C] |
| - PigN | GO:0016740 | transferase activity | [M] |
|  | GO:0006506 | GPI anchor biosynthetic process | [B] |
|  | GO:0005789 | endoplasmic reticulum membrane | [C] |
| - Pirin |  |  |  |  |
| - Pirin\_C |  |  |  |  |
| - PMC2NT | GO:0006396 | RNA processing | [B] |
|  | GO:0000176 | nuclear exosome (RNase complex) | [C] |
| - Pmp24 |  |  |  |  |
| - POLO\_box | GO:0004674 | protein serine/threonine kinase activity | [M] |
|  | GO:0007049 | cell cycle | [B] |
| - potato\_inhibit | GO:0004867 | serine-type endopeptidase inhibitor activity | [M] |
|  | GO:0009611 | response to wounding | [B] |
| - Pox\_A32 | GO:0005524 | ATP binding | [M] |
| - Pro\_CA | GO:0004089 | carbonate dehydratase activity | [M] |
|  | GO:0008270 | zinc ion binding | [M] |
|  | GO:0015976 | carbon utilization | [B] |
| - Propeptide\_C1 | GO:0004197 | cysteine-type endopeptidase activity | [M] |
|  | GO:0050790 | regulation of catalytic activity | [B] |
| - PRT\_C |  |  |  |  |
| - PSI | GO:0016020 | membrane | [C] |
| - Pterin\_4a | GO:0008124 | 4-alpha-hydroxytetrahydrobiopterin dehydratase activity | [M] |
|  | GO:0006729 | tetrahydrobiopterin biosynthetic process | [B] |
| - Put\_Phosphatase | GO:0016791 | phosphatase activity | [M] |
| - PWWP |  |  |  |  |
| - PYNP\_C | GO:0016763 | transferase activity, transferring pentosyl groups | [M] |
|  | GO:0006213 | pyrimidine nucleoside metabolic process | [B] |
| - Pyr\_redox | GO:0016491 | oxidoreductase activity | [M] |
|  | GO:0050660 | FAD binding | [M] |
|  | GO:0055114 | oxidation reduction | [B] |
| - Pyridoxal\_deC | GO:0016831 | carboxy-lyase activity | [M] |
|  | GO:0030170 | pyridoxal phosphate binding | [M] |
|  | GO:0019752 | carboxylic acid metabolic process | [B] |
| - QLQ | GO:0005524 | ATP binding | [M] |
|  | GO:0016818 | hydrolase activity, acting on acid anhydrides, in phosphorus-containing anhydrides | [M] |
|  | GO:0045449 | regulation of transcription | [B] |
|  | GO:0005634 | nucleus | [C] |
| - RA | GO:0007165 | signal transduction | [B] |
| - Rap\_GAP | GO:0005096 | GTPase activator activity | [M] |
|  | GO:0051056 | regulation of small GTPase mediated signal transduction | [B] |
|  | GO:0005622 | intracellular | [C] |
| - RasGAP | GO:0005096 | GTPase activator activity | [M] |
|  | GO:0051056 | regulation of small GTPase mediated signal transduction | [B] |
|  | GO:0005622 | intracellular | [C] |
| - RasGEF | GO:0005085 | guanyl-nucleotide exchange factor activity | [M] |
|  | GO:0007264 | small GTPase mediated signal transduction | [B] |
|  | GO:0005622 | intracellular | [C] |
| - RB\_A |  |  |  |  |
| - RB\_B |  |  |  |  |
| - RdRP |  |  |  |  |
| - REJ |  |  |  |  |
| - Rep-A\_N | GO:0003677 | DNA binding | [M] |
|  | GO:0006260 | DNA replication | [B] |
|  | GO:0005634 | nucleus | [C] |
| - Rho\_GDI | GO:0005094 | Rho GDP-dissociation inhibitor activity | [M] |
|  | GO:0005737 | cytoplasm | [C] |
| - RhoGEF | GO:0005089 | Rho guanyl-nucleotide exchange factor activity | [M] |
|  | GO:0035023 | regulation of Rho protein signal transduction | [B] |
|  | GO:0005622 | intracellular | [C] |
| - RIB43A |  |  |  |  |
| - Ribonuc\_2-5A | GO:0016891 | endoribonuclease activity, producing 5'-phosphomonoesters | [M] |
|  | GO:0006397 | mRNA processing | [B] |
| - Ribonuc\_L-PSP |  |  |  |  |
| - Ribonuclease\_3 | GO:0003723 | RNA binding | [M] |
|  | GO:0004525 | ribonuclease III activity | [M] |
|  | GO:0006396 | RNA processing | [B] |
| - Ribonuclease\_T2 | GO:0003723 | RNA binding | [M] |
|  | GO:0033897 | ribonuclease T2 activity | [M] |
| - RIH\_assoc |  |  |  |  |
| - RmlD\_sub\_bind | GO:0008831 | dTDP-4-dehydrorhamnose reductase activity | [M] |
|  | GO:0045226 | extracellular polysaccharide biosynthetic process | [B] |
| - RNA\_lig\_T4\_1 |  |  |  |  |
| - RNA\_pol\_Rpb2\_2 | GO:0003677 | DNA binding | [M] |
|  | GO:0003899 | DNA-directed RNA polymerase activity | [M] |
|  | GO:0006350 | transcription | [B] |
| - RNA\_polI\_A34 |  |  |  |  |
| - ROK |  |  |  |  |
| - RPE65 |  |  |  |  |
| - Rsm1 |  |  |  |  |
| - Rubis-subs-bind |  |  |  |  |
| - Rxt3 |  |  |  |  |
| - RYDR\_ITPR | GO:0005262 | calcium channel activity | [M] |
|  | GO:0006816 | calcium ion transport | [B] |
|  | GO:0016020 | membrane | [C] |
| - Saccharop\_dh\_N |  |  |  |  |
| - SapB\_1 | GO:0006629 | lipid metabolic process | [B] |
| - SBP56 | GO:0008430 | selenium binding | [M] |
| - SCAMP | GO:0015031 | protein transport | [B] |
|  | GO:0016021 | integral to membrane | [C] |
| - Sds3 |  |  |  |  |
| - Sec10 | GO:0006887 | exocytosis | [B] |
|  | GO:0048278 | vesicle docking | [B] |
|  | GO:0005737 | cytoplasm | [C] |
| - Sec15 | GO:0006904 | vesicle docking during exocytosis | [B] |
|  | GO:0000145 | exocyst | [C] |
| - Sec3 |  |  |  |  |
| - Sec34 | GO:0006886 | intracellular protein transport | [B] |
|  | GO:0005801 | cis-Golgi network | [C] |
|  | GO:0016020 | membrane | [C] |
| - Sec6 |  |  |  |  |
| - Sec8\_exocyst | GO:0006904 | vesicle docking during exocytosis | [B] |
|  | GO:0015031 | protein transport | [B] |
|  | GO:0000145 | exocyst | [C] |
| - SecA\_DEAD | GO:0005524 | ATP binding | [M] |
|  | GO:0017038 | protein import | [B] |
|  | GO:0016020 | membrane | [C] |
| - Senescence |  |  |  |  |
| - Septin | GO:0005525 | GTP binding | [M] |
|  | GO:0007049 | cell cycle | [B] |
| - Serinc | GO:0016020 | membrane | [C] |
| - SGL |  |  |  |  |
| - SH2 | GO:0005515 | protein binding | [M] |
| - SH3\_1 |  |  |  |  |
| - SH3BGR |  |  |  |  |
| - Shikimate\_DH | GO:0004764 | shikimate 5-dehydrogenase activity | [M] |
|  | GO:0055114 | oxidation reduction | [B] |
|  | GO:0005737 | cytoplasm | [C] |
| - SIMPL |  |  |  |  |
| - SIN1 |  |  |  |  |
| - Sina | GO:0006511 | ubiquitin-dependent protein catabolic process | [B] |
|  | GO:0007275 | multicellular organismal development | [B] |
|  | GO:0005634 | nucleus | [C] |
| - SIP1 | GO:0000245 | spliceosome assembly | [B] |
|  | GO:0000398 | nuclear mRNA splicing, via spliceosome | [B] |
| - SLBB |  |  |  |  |
| - SMN | GO:0003723 | RNA binding | [M] |
|  | GO:0000245 | spliceosome assembly | [B] |
|  | GO:0006397 | mRNA processing | [B] |
|  | GO:0005634 | nucleus | [C] |
|  | GO:0005737 | cytoplasm | [C] |
| - Smr |  |  |  |  |
| - SNF5 | GO:0006338 | chromatin remodeling | [B] |
|  | GO:0000228 | nuclear chromosome | [C] |
| - Sod\_Cu | GO:0046872 | metal ion binding | [M] |
|  | GO:0006801 | superoxide metabolic process | [B] |
|  | GO:0055114 | oxidation reduction | [B] |
| - SPB\_interacting |  |  |  |  |
| - Spectrin |  |  |  |  |
| - SQS\_PSY | GO:0016740 | transferase activity | [M] |
|  | GO:0009058 | biosynthetic process | [B] |
| - SRF-TF | GO:0003700 | transcription factor activity | [M] |
|  | GO:0043565 | sequence-specific DNA binding | [M] |
|  | GO:0006355 | regulation of transcription, DNA-dependent | [B] |
|  | GO:0005634 | nucleus | [C] |
| - Stanniocalcin | GO:0005179 | hormone activity | [M] |
|  | GO:0005576 | extracellular region | [C] |
| - Sterile |  |  |  |  |
| - Strep\_SA\_rep |  |  |  |  |
| - Striatin |  |  |  |  |
| - Strumpellin |  |  |  |  |
| - Sulfatase | GO:0008484 | sulfuric ester hydrolase activity | [M] |
|  | GO:0008152 | metabolic process | [B] |
| - SURF2 |  |  |  |  |
| - Swi3 | GO:0006974 | response to DNA damage stimulus | [B] |
|  | GO:0007049 | cell cycle | [B] |
|  | GO:0048478 | replication fork protection | [B] |
|  | GO:0005634 | nucleus | [C] |
| - Swi5 |  |  |  |  |
| - T4\_deiodinase | GO:0004800 | thyroxine 5'-deiodinase activity | [M] |
|  | GO:0055114 | oxidation reduction | [B] |
| - TauD | GO:0016491 | oxidoreductase activity | [M] |
|  | GO:0055114 | oxidation reduction | [B] |
| - Taxilin |  |  |  |  |
| - Telo\_bind | GO:0003677 | DNA binding | [M] |
|  | GO:0000723 | telomere maintenance | [B] |
|  | GO:0000784 | nuclear chromosome, telomeric region | [C] |
| - Telomere\_reg-2 |  |  |  |  |
| - TerD | GO:0006950 | response to stress | [B] |
| - Tetraspannin | GO:0016021 | integral to membrane | [C] |
| - TFCD\_C |  |  |  |  |
| - TFIIA\_gamma\_N | GO:0003702 | RNA polymerase II transcription factor activity | [M] |
|  | GO:0006367 | transcription initiation from RNA polymerase II promoter | [B] |
|  | GO:0005672 | transcription factor TFIIA complex | [C] |
| - TFIID-31kDa | GO:0006352 | transcription initiation | [B] |
| - THOC7 |  |  |  |  |
| - Thr\_dehydrat\_C | GO:0004794 | L-threonine ammonia-lyase activity | [M] |
|  | GO:0009097 | isoleucine biosynthetic process | [B] |
| - TIMELESS |  |  |  |  |
| - TIMELESS\_C |  |  |  |  |
| - TIP41 |  |  |  |  |
| - Tmemb\_185A |  |  |  |  |
| - Tmp39 |  |  |  |  |
| - Tom7 | GO:0015450 | P-P-bond-hydrolysis-driven protein transmembrane transporter activity | [M] |
|  | GO:0006886 | intracellular protein transport | [B] |
|  | GO:0005741 | mitochondrial outer membrane | [C] |
| - TPX2 |  |  |  |  |
| - TraB |  |  |  |  |
| - Trehalase | GO:0004555 | alpha,alpha-trehalase activity | [M] |
|  | GO:0005991 | trehalose metabolic process | [B] |
| - TrmB |  |  |  |  |
| - tRNA\_synt\_1c\_R1 | GO:0000166 | nucleotide binding | [M] |
|  | GO:0004812 | aminoacyl-tRNA ligase activity | [M] |
|  | GO:0005524 | ATP binding | [M] |
|  | GO:0006412 | translation | [B] |
|  | GO:0006418 | tRNA aminoacylation for protein translation | [B] |
|  | GO:0005737 | cytoplasm | [C] |
| - tRNA\_synt\_1c\_R2 | GO:0000166 | nucleotide binding | [M] |
|  | GO:0004819 | glutamine-tRNA ligase activity | [M] |
|  | GO:0005524 | ATP binding | [M] |
|  | GO:0006412 | translation | [B] |
|  | GO:0006425 | glutaminyl-tRNA aminoacylation | [B] |
|  | GO:0005737 | cytoplasm | [C] |
| - TROVE | GO:0003723 | RNA binding | [M] |
|  | GO:0030529 | ribonucleoprotein complex | [C] |
| - TRP |  |  |  |  |
| - Tup\_N |  |  |  |  |
| - uDENN |  |  |  |  |
| - UMPH-1 | GO:0000287 | magnesium ion binding | [M] |
|  | GO:0008253 | 5'-nucleotidase activity | [M] |
|  | GO:0005737 | cytoplasm | [C] |
| - UNC-93 |  |  |  |  |
| - UPF0061 |  |  |  |  |
| - UPF0075 | GO:0005524 | ATP binding | [M] |
|  | GO:0016773 | phosphotransferase activity, alcohol group as acceptor | [M] |
|  | GO:0006040 | amino sugar metabolic process | [B] |
|  | GO:0009254 | peptidoglycan turnover | [B] |
| - UPF0081 | GO:0000150 | recombinase activity | [M] |
|  | GO:0003677 | DNA binding | [M] |
|  | GO:0004518 | nuclease activity | [M] |
|  | GO:0006281 | DNA repair | [B] |
|  | GO:0006310 | DNA recombination | [B] |
|  | GO:0006974 | response to DNA damage stimulus | [B] |
| - UPF0118 |  |  |  |  |
| - UPF0139 |  |  |  |  |
| - UPF0157 |  |  |  |  |
| - UPF0193 |  |  |  |  |
| - UPF0197 |  |  |  |  |
| - UPF0227 |  |  |  |  |
| - UPF0564 |  |  |  |  |
| - Urocanase | GO:0016153 | urocanate hydratase activity | [M] |
|  | GO:0006548 | histidine catabolic process | [B] |
| - Use1 |  |  |  |  |
| - UvdE | GO:0004519 | endonuclease activity | [M] |
|  | GO:0006289 | nucleotide-excision repair | [B] |
| - UVR | GO:0003677 | DNA binding | [M] |
|  | GO:0004518 | nuclease activity | [M] |
|  | GO:0006289 | nucleotide-excision repair | [B] |
| - VanY | GO:0008233 | peptidase activity | [M] |
|  | GO:0006508 | proteolysis | [B] |
| - Vault |  |  |  |  |
| - VEFS-Box |  |  |  |  |
| - VHP | GO:0003779 | actin binding | [M] |
|  | GO:0007010 | cytoskeleton organization | [B] |
| - VHS | GO:0006886 | intracellular protein transport | [B] |
| - VIT |  |  |  |  |
| - Vps23\_core |  |  |  |  |
| - VSP |  |  |  |  |
| - VWA\_CoxE |  |  |  |  |
| - WBP-1 |  |  |  |  |
| - WDYHV |  |  |  |  |
| - WH1 |  |  |  |  |
| - WH2 |  |  |  |  |
| - Whirly |  |  |  |  |
| - XLF |  |  |  |  |
| - Y\_phosphatase | GO:0004725 | protein tyrosine phosphatase activity | [M] |
|  | GO:0006470 | protein amino acid dephosphorylation | [B] |
| - YCII |  |  |  |  |
| - YdjC |  |  |  |  |
| - YhhN | GO:0016021 | integral to membrane | [C] |
| - YqaJ |  |  |  |  |
| - zf-4CXXC\_R1 |  |  |  |  |
| - zf-BED | GO:0003677 | DNA binding | [M] |
| - zf-CCHH |  |  |  |  |
| - zf-CHCC |  |  |  |  |
| - zf-DBF | GO:0003676 | nucleic acid binding | [M] |
|  | GO:0008270 | zinc ion binding | [M] |
| - zf-U11-48K |  |  |  |  |

---

## Arabidopsis [eol|tol]

|  |  |  |  |
| --- | --- | --- | --- |
| **Pfam domain(s)** | **GO term acc** | **GO term** | **GO namespace** |
| - 2CSK\_N |  |  |  |  |
| - 3-dmu-9\_3-mt |  |  |  |  |
| - 7TMR-DISM\_7TM |  |  |  |  |
| - A2M\_N\_2 |  |  |  |  |
| - AAA\_3 | GO:0005524 | ATP binding | [M] |
|  | GO:0016887 | ATPase activity | [M] |
| - AAT | GO:0042318 | penicillin biosynthetic process | [B] |
| - ABA\_WDS | GO:0006950 | response to stress | [B] |
| - ABM | GO:0016491 | oxidoreductase activity | [M] |
|  | GO:0017000 | antibiotic biosynthetic process | [B] |
|  | GO:0005737 | cytoplasm | [C] |
| - Acetate\_kinase | GO:0016301 | kinase activity | [M] |
|  | GO:0016774 | phosphotransferase activity, carboxyl group as acceptor | [M] |
|  | GO:0008152 | metabolic process | [B] |
|  | GO:0016310 | phosphorylation | [B] |
|  | GO:0005622 | intracellular | [C] |
| - Aconitase\_2\_N | GO:0003994 | aconitate hydratase activity | [M] |
|  | GO:0005515 | protein binding | [M] |
|  | GO:0006099 | tricarboxylic acid cycle | [B] |
| - Aconitase\_B\_N |  |  |  |  |
| - ACR\_tran | GO:0005215 | transporter activity | [M] |
|  | GO:0006810 | transport | [B] |
|  | GO:0016020 | membrane | [C] |
| - Acyl\_transf\_3 | GO:0016747 | transferase activity, transferring acyl groups other than amino-acyl groups | [M] |
| - AcylCoA\_DH\_N |  |  |  |  |
| - Ada\_Zn\_binding | GO:0003677 | DNA binding | [M] |
|  | GO:0008168 | methyltransferase activity | [M] |
|  | GO:0008270 | zinc ion binding | [M] |
|  | GO:0006281 | DNA repair | [B] |
|  | GO:0006355 | regulation of transcription, DNA-dependent | [B] |
| - ADC | GO:0016831 | carboxy-lyase activity | [M] |
| - Adenosine\_kin |  |  |  |  |
| - AdoMetDC\_leader |  |  |  |  |
| - AHS1 |  |  |  |  |
| - AlcB |  |  |  |  |
| - Aldolase | GO:0003824 | catalytic activity | [M] |
|  | GO:0008152 | metabolic process | [B] |
| - Alginate\_lyase2 |  |  |  |  |
| - Alum\_res |  |  |  |  |
| - Amidase\_3 | GO:0008745 | N-acetylmuramoyl-L-alanine amidase activity | [M] |
|  | GO:0009253 | peptidoglycan catabolic process | [B] |
| - ApbA |  |  |  |  |
| - ApbA\_C | GO:0016491 | oxidoreductase activity | [M] |
|  | GO:0050661 | NADP or NADPH binding | [M] |
|  | GO:0055114 | oxidation reduction | [B] |
| - AraC\_binding | GO:0006355 | regulation of transcription, DNA-dependent | [B] |
| - Arch\_ATPase | GO:0005524 | ATP binding | [M] |
| - ArsB | GO:0015105 | arsenite transmembrane transporter activity | [M] |
|  | GO:0016021 | integral to membrane | [C] |
| - ArsC |  |  |  |  |
| - AsmA |  |  |  |  |
| - AsnC\_trans\_reg | GO:0003700 | transcription factor activity | [M] |
|  | GO:0043565 | sequence-specific DNA binding | [M] |
|  | GO:0006355 | regulation of transcription, DNA-dependent | [B] |
|  | GO:0005622 | intracellular | [C] |
| - ATP-synt\_E | GO:0015078 | hydrogen ion transmembrane transporter activity | [M] |
|  | GO:0015986 | ATP synthesis coupled proton transport | [B] |
|  | GO:0000276 | mitochondrial proton-transporting ATP synthase complex, coupling factor F(o) | [C] |
| - Autotransporter |  |  |  |  |
| - B12-binding | GO:0031419 | cobalamin binding | [M] |
|  | GO:0046872 | metal ion binding | [M] |
| - B12-binding\_2 | GO:0008705 | methionine synthase activity | [M] |
|  | GO:0031419 | cobalamin binding | [M] |
|  | GO:0046872 | metal ion binding | [M] |
|  | GO:0009086 | methionine biosynthetic process | [B] |
| - Bac\_DNA\_binding | GO:0003677 | DNA binding | [M] |
| - Bac\_DnaA |  |  |  |  |
| - Bac\_export\_1 | GO:0006605 | protein targeting | [B] |
|  | GO:0016020 | membrane | [C] |
| - Bac\_export\_2 | GO:0009306 | protein secretion | [B] |
|  | GO:0016020 | membrane | [C] |
| - Bac\_luciferase |  |  |  |  |
| - Bac\_Ubq\_Cox | GO:0016491 | oxidoreductase activity | [M] |
|  | GO:0055114 | oxidation reduction | [B] |
|  | GO:0016020 | membrane | [C] |
| - Baseplate\_J |  |  |  |  |
| - BAT2\_N |  |  |  |  |
| - BCA\_ABC\_TP\_C |  |  |  |  |
| - BCCT | GO:0005215 | transporter activity | [M] |
|  | GO:0006810 | transport | [B] |
|  | GO:0016020 | membrane | [C] |
| - Big\_2 |  |  |  |  |
| - Bmp | GO:0008289 | lipid binding | [M] |
| - BON |  |  |  |  |
| - Bot1p |  |  |  |  |
| - Bowman-Birk\_leg | GO:0004867 | serine-type endopeptidase inhibitor activity | [M] |
|  | GO:0005576 | extracellular region | [C] |
| - BPD\_transp\_1 | GO:0005215 | transporter activity | [M] |
|  | GO:0006810 | transport | [B] |
|  | GO:0016020 | membrane | [C] |
| - BPD\_transp\_2 | GO:0005215 | transporter activity | [M] |
|  | GO:0006810 | transport | [B] |
|  | GO:0016020 | membrane | [C] |
| - Bre5 |  |  |  |  |
| - Bug | GO:0030288 | outer membrane-bounded periplasmic space | [C] |
| - Cache\_1 | GO:0016020 | membrane | [C] |
| - CagX |  |  |  |  |
| - Capsule\_synth | GO:0000271 | polysaccharide biosynthetic process | [B] |
|  | GO:0015774 | polysaccharide transport | [B] |
| - Cauli\_VI | GO:0016234 | inclusion body | [C] |
| - CDO\_I | GO:0005506 | iron ion binding | [M] |
|  | GO:0017172 | cysteine dioxygenase activity | [M] |
|  | GO:0046439 | L-cysteine metabolic process | [B] |
|  | GO:0055114 | oxidation reduction | [B] |
| - CENP-B\_N | GO:0003677 | DNA binding | [M] |
|  | GO:0000775 | chromosome, centromeric region | [C] |
| - CHDCT2 | GO:0003677 | DNA binding | [M] |
|  | GO:0005524 | ATP binding | [M] |
|  | GO:0008270 | zinc ion binding | [M] |
|  | GO:0016818 | hydrolase activity, acting on acid anhydrides, in phosphorus-containing anhydrides | [M] |
|  | GO:0045449 | regulation of transcription | [B] |
|  | GO:0005634 | nucleus | [C] |
| - CheR | GO:0008757 | S-adenosylmethionine-dependent methyltransferase activity | [M] |
| - CheR\_N | GO:0008757 | S-adenosylmethionine-dependent methyltransferase activity | [M] |
| - CheW | GO:0004871 | signal transducer activity | [M] |
|  | GO:0006935 | chemotaxis | [B] |
|  | GO:0007165 | signal transduction | [B] |
|  | GO:0005622 | intracellular | [C] |
| - Chromate\_transp | GO:0015109 | chromate transmembrane transporter activity | [M] |
|  | GO:0015703 | chromate transport | [B] |
| - CinA |  |  |  |  |
| - CMD |  |  |  |  |
| - CoA\_trans | GO:0008410 | CoA-transferase activity | [M] |
|  | GO:0008152 | metabolic process | [B] |
| - CoA\_transf\_3 | GO:0003824 | catalytic activity | [M] |
|  | GO:0008152 | metabolic process | [B] |
| - CobA\_CobO\_BtuR | GO:0005524 | ATP binding | [M] |
|  | GO:0008817 | cob(I)yrinic acid a,c-diamide adenosyltransferase activity | [M] |
|  | GO:0009236 | cobalamin biosynthetic process | [B] |
| - Competence\_A |  |  |  |  |
| - Condensation |  |  |  |  |
| - Cons\_hypoth698 | GO:0016021 | integral to membrane | [C] |
| - Cornifin |  |  |  |  |
| - CPL | GO:0003723 | RNA binding | [M] |
| - Creatininase |  |  |  |  |
| - Crp | GO:0003700 | transcription factor activity | [M] |
|  | GO:0006355 | regulation of transcription, DNA-dependent | [B] |
|  | GO:0005622 | intracellular | [C] |
| - CsbD |  |  |  |  |
| - CstA | GO:0009267 | cellular response to starvation | [B] |
|  | GO:0016020 | membrane | [C] |
| - CTK3 |  |  |  |  |
| - Cyto\_ox\_2 | GO:0055114 | oxidation reduction | [B] |
|  | GO:0016020 | membrane | [C] |
| - Cytochrom\_D1 |  |  |  |  |
| - DAGK\_prokar | GO:0004143 | diacylglycerol kinase activity | [M] |
|  | GO:0008654 | phospholipid biosynthetic process | [B] |
|  | GO:0016020 | membrane | [C] |
| - DBI\_PRT | GO:0008939 | nicotinate-nucleotide-dimethylbenzimidazole phosphoribosyltransferase activity | [M] |
|  | GO:0009236 | cobalamin biosynthetic process | [B] |
| - DctM |  |  |  |  |
| - DDE | GO:0003676 | nucleic acid binding | [M] |
| - DegT\_DnrJ\_EryC1 |  |  |  |  |
| - DHHA2 | GO:0016462 | pyrophosphatase activity | [M] |
|  | GO:0005737 | cytoplasm | [C] |
| - DivIC | GO:0007049 | cell cycle | [B] |
| - DNA\_pol3\_alpha | GO:0008408 | 3'-5' exonuclease activity | [M] |
|  | GO:0006260 | DNA replication | [B] |
|  | GO:0005737 | cytoplasm | [C] |
| - DNA\_pol3\_beta | GO:0003677 | DNA binding | [M] |
|  | GO:0003887 | DNA-directed DNA polymerase activity | [M] |
|  | GO:0008408 | 3'-5' exonuclease activity | [M] |
|  | GO:0006260 | DNA replication | [B] |
| - DNA\_pol\_B\_2 | GO:0000166 | nucleotide binding | [M] |
|  | GO:0003677 | DNA binding | [M] |
|  | GO:0003887 | DNA-directed DNA polymerase activity | [M] |
|  | GO:0008408 | 3'-5' exonuclease activity | [M] |
|  | GO:0006260 | DNA replication | [B] |
| - DnaB | GO:0003678 | DNA helicase activity | [M] |
|  | GO:0005524 | ATP binding | [M] |
|  | GO:0006260 | DNA replication | [B] |
| - DrsE |  |  |  |  |
| - DsbB | GO:0015035 | protein disulfide oxidoreductase activity | [M] |
|  | GO:0016020 | membrane | [C] |
| - dTDP\_sugar\_isom | GO:0008830 | dTDP-4-dehydrorhamnose 3,5-epimerase activity | [M] |
|  | GO:0009103 | lipopolysaccharide biosynthetic process | [B] |
| - DUF1016 |  |  |  |  |
| - DUF1078 | GO:0019861 | flagellum | [C] |
| - DUF1173 |  |  |  |  |
| - DUF1214 |  |  |  |  |
| - DUF1234 |  |  |  |  |
| - DUF1254 |  |  |  |  |
| - DUF1255 |  |  |  |  |
| - DUF1275 |  |  |  |  |
| - DUF1338 |  |  |  |  |
| - DUF1348 |  |  |  |  |
| - DUF1468 |  |  |  |  |
| - DUF1631 |  |  |  |  |
| - DUF1725 |  |  |  |  |
| - DUF1730 |  |  |  |  |
| - DUF1759 |  |  |  |  |
| - DUF1814 |  |  |  |  |
| - DUF1903 |  |  |  |  |
| - DUF192 |  |  |  |  |
| - DUF205 | GO:0005886 | plasma membrane | [C] |
| - DUF2075 |  |  |  |  |
| - DUF2169 |  |  |  |  |
| - DUF2365 |  |  |  |  |
| - DUF2415 |  |  |  |  |
| - DUF2419 |  |  |  |  |
| - DUF2466 | GO:0006281 | DNA repair | [B] |
| - DUF2647 |  |  |  |  |
| - DUF2843 |  |  |  |  |
| - DUF297 |  |  |  |  |
| - DUF304 |  |  |  |  |
| - DUF3119 |  |  |  |  |
| - DUF3168 |  |  |  |  |
| - DUF330 |  |  |  |  |
| - DUF3326 |  |  |  |  |
| - DUF336 |  |  |  |  |
| - DUF3362 |  |  |  |  |
| - DUF3422 |  |  |  |  |
| - DUF347 |  |  |  |  |
| - DUF3615 |  |  |  |  |
| - DUF3651 |  |  |  |  |
| - DUF377 |  |  |  |  |
| - DUF411 |  |  |  |  |
| - DUF45 |  |  |  |  |
| - DUF461 |  |  |  |  |
| - DUF484 |  |  |  |  |
| - DUF519 |  |  |  |  |
| - DUF520 |  |  |  |  |
| - DUF561 |  |  |  |  |
| - DUF72 |  |  |  |  |
| - DUF727 |  |  |  |  |
| - DUF729 |  |  |  |  |
| - DUF745 |  |  |  |  |
| - DUF77 |  |  |  |  |
| - DUF815 |  |  |  |  |
| - DUF847 |  |  |  |  |
| - DUF917 |  |  |  |  |
| - DUF924 |  |  |  |  |
| - Dyp\_perox |  |  |  |  |
| - EAL |  |  |  |  |
| - EGF |  |  |  |  |
| - Endonuclease\_1 | GO:0004518 | nuclease activity | [M] |
| - Endonuclease\_NS | GO:0003676 | nucleic acid binding | [M] |
|  | GO:0016787 | hydrolase activity | [M] |
|  | GO:0046872 | metal ion binding | [M] |
| - Esterase\_phd | GO:0005576 | extracellular region | [C] |
| - EutB | GO:0008851 | ethanolamine ammonia-lyase activity | [M] |
|  | GO:0006520 | cellular amino acid metabolic process | [B] |
| - ExbD | GO:0005215 | transporter activity | [M] |
|  | GO:0006810 | transport | [B] |
|  | GO:0016020 | membrane | [C] |
| - Exonuc\_V\_gamma | GO:0008854 | exodeoxyribonuclease V activity | [M] |
|  | GO:0009338 | exodeoxyribonuclease V complex | [C] |
| - FAD\_binding\_9 |  |  |  |  |
| - Fascin | GO:0030674 | protein binding, bridging | [M] |
|  | GO:0051015 | actin filament binding | [M] |
| - FCD |  |  |  |  |
| - FecCD | GO:0005215 | transporter activity | [M] |
|  | GO:0006810 | transport | [B] |
|  | GO:0016020 | membrane | [C] |
| - FecR |  |  |  |  |
| - Fer2\_BFD |  |  |  |  |
| - FeS | GO:0051536 | iron-sulfur cluster binding | [M] |
| - FGase |  |  |  |  |
| - FHIPEP | GO:0009306 | protein secretion | [B] |
|  | GO:0016020 | membrane | [C] |
| - FhuF |  |  |  |  |
| - Fimbrial |  |  |  |  |
| - Flagellin\_C | GO:0005198 | structural molecule activity | [M] |
|  | GO:0001539 | ciliary or flagellar motility | [B] |
|  | GO:0009288 | bacterial-type flagellum | [C] |
| - Flagellin\_N | GO:0005198 | structural molecule activity | [M] |
|  | GO:0001539 | ciliary or flagellar motility | [B] |
|  | GO:0009420 | bacterial-type flagellum filament | [C] |
| - Flavodoxin\_2 | GO:0009055 | electron carrier activity | [M] |
|  | GO:0016491 | oxidoreductase activity | [M] |
|  | GO:0050662 | coenzyme binding | [M] |
| - Flg\_bb\_rod | GO:0003774 | motor activity | [M] |
|  | GO:0005198 | structural molecule activity | [M] |
|  | GO:0001539 | ciliary or flagellar motility | [B] |
|  | GO:0009288 | bacterial-type flagellum | [C] |
| - FliE | GO:0003774 | motor activity | [M] |
|  | GO:0005198 | structural molecule activity | [M] |
|  | GO:0001539 | ciliary or flagellar motility | [B] |
|  | GO:0009288 | bacterial-type flagellum | [C] |
| - FR47 |  |  |  |  |
| - Fringe | GO:0016757 | transferase activity, transferring glycosyl groups | [M] |
|  | GO:0016020 | membrane | [C] |
| - FTR1 | GO:0055085 | transmembrane transport | [B] |
|  | GO:0016020 | membrane | [C] |
| - Ftsk\_gamma |  |  |  |  |
| - FtsK\_SpoIIIE | GO:0000166 | nucleotide binding | [M] |
|  | GO:0003677 | DNA binding | [M] |
|  | GO:0005524 | ATP binding | [M] |
|  | GO:0007049 | cell cycle | [B] |
|  | GO:0007059 | chromosome segregation | [B] |
|  | GO:0051301 | cell division | [B] |
|  | GO:0016021 | integral to membrane | [C] |
| - FTSW\_RODA\_SPOVE | GO:0007049 | cell cycle | [B] |
|  | GO:0016021 | integral to membrane | [C] |
| - FtsX | GO:0016020 | membrane | [C] |
| - FUR | GO:0003700 | transcription factor activity | [M] |
|  | GO:0006355 | regulation of transcription, DNA-dependent | [B] |
| - GATase\_3 | GO:0003824 | catalytic activity | [M] |
|  | GO:0009236 | cobalamin biosynthetic process | [B] |
| - GD\_AH\_C | GO:0016836 | hydro-lyase activity | [M] |
| - GerE | GO:0003700 | transcription factor activity | [M] |
|  | GO:0043565 | sequence-specific DNA binding | [M] |
|  | GO:0006355 | regulation of transcription, DNA-dependent | [B] |
|  | GO:0005622 | intracellular | [C] |
| - GGDEF |  |  |  |  |
| - Glt\_symporter | GO:0015501 | glutamate:sodium symporter activity | [M] |
|  | GO:0015813 | L-glutamate transport | [B] |
|  | GO:0016021 | integral to membrane | [C] |
| - Glu\_cys\_ligase | GO:0004357 | glutamate-cysteine ligase activity | [M] |
|  | GO:0006750 | glutathione biosynthetic process | [B] |
| - Glyco\_hydro\_68 | GO:0050053 | levansucrase activity | [M] |
|  | GO:0007587 | sugar utilization | [B] |
| - Glyco\_transf\_9 | GO:0016740 | transferase activity | [M] |
|  | GO:0008152 | metabolic process | [B] |
| - GntP\_permease | GO:0015128 | gluconate transmembrane transporter activity | [M] |
|  | GO:0015725 | gluconate transport | [B] |
|  | GO:0016020 | membrane | [C] |
| - GntR | GO:0003700 | transcription factor activity | [M] |
|  | GO:0006355 | regulation of transcription, DNA-dependent | [B] |
|  | GO:0005622 | intracellular | [C] |
| - GPW\_gp25 |  |  |  |  |
| - GreA\_GreB | GO:0003677 | DNA binding | [M] |
|  | GO:0003711 | transcription elongation regulator activity | [M] |
|  | GO:0006355 | regulation of transcription, DNA-dependent | [B] |
| - GreA\_GreB\_N | GO:0003677 | DNA binding | [M] |
|  | GO:0003711 | transcription elongation regulator activity | [M] |
|  | GO:0006355 | regulation of transcription, DNA-dependent | [B] |
| - GSH-S\_ATP | GO:0004363 | glutathione synthase activity | [M] |
|  | GO:0005524 | ATP binding | [M] |
|  | GO:0006750 | glutathione biosynthetic process | [B] |
| - GSH-S\_N | GO:0004363 | glutathione synthase activity | [M] |
|  | GO:0006750 | glutathione biosynthetic process | [B] |
| - GSPII\_E | GO:0005524 | ATP binding | [M] |
|  | GO:0006810 | transport | [B] |
|  | GO:0005622 | intracellular | [C] |
| - GSPII\_E\_N | GO:0005524 | ATP binding | [M] |
|  | GO:0006810 | transport | [B] |
| - GSPII\_F |  |  |  |  |
| - GSPII\_IJ | GO:0008565 | protein transporter activity | [M] |
|  | GO:0015628 | protein secretion by the type II secretion system | [B] |
|  | GO:0015627 | type II protein secretion system complex | [C] |
| - GspK | GO:0009306 | protein secretion | [B] |
|  | GO:0016021 | integral to membrane | [C] |
| - Haemagg\_act | GO:0005488 | binding | [M] |
| - HAMP | GO:0004871 | signal transducer activity | [M] |
|  | GO:0007165 | signal transduction | [B] |
|  | GO:0016021 | integral to membrane | [C] |
| - HCaRG |  |  |  |  |
| - HCR | GO:0030154 | cell differentiation | [B] |
|  | GO:0005634 | nucleus | [C] |
|  | GO:0005737 | cytoplasm | [C] |
| - HHH | GO:0003677 | DNA binding | [M] |
|  | GO:0005622 | intracellular | [C] |
| - HipA\_C |  |  |  |  |
| - HipA\_N |  |  |  |  |
| - His\_kinase | GO:0000155 | two-component sensor activity | [M] |
|  | GO:0000160 | two-component signal transduction system (phosphorelay) | [B] |
|  | GO:0016021 | integral to membrane | [C] |
| - HisKA\_3 | GO:0000155 | two-component sensor activity | [M] |
|  | GO:0046983 | protein dimerization activity | [M] |
|  | GO:0000160 | two-component signal transduction system (phosphorelay) | [B] |
|  | GO:0016021 | integral to membrane | [C] |
| - Histone\_HNS | GO:0003677 | DNA binding | [M] |
|  | GO:0006355 | regulation of transcription, DNA-dependent | [B] |
|  | GO:0005622 | intracellular | [C] |
| - HlyD | GO:0008565 | protein transporter activity | [M] |
|  | GO:0009306 | protein secretion | [B] |
|  | GO:0016020 | membrane | [C] |
| - HTH\_1 | GO:0003700 | transcription factor activity | [M] |
|  | GO:0006355 | regulation of transcription, DNA-dependent | [B] |
| - HTH\_11 |  |  |  |  |
| - HTH\_5 | GO:0003700 | transcription factor activity | [M] |
|  | GO:0006355 | regulation of transcription, DNA-dependent | [B] |
|  | GO:0005622 | intracellular | [C] |
| - HTH\_6 | GO:0003700 | transcription factor activity | [M] |
|  | GO:0006355 | regulation of transcription, DNA-dependent | [B] |
| - HTH\_8 | GO:0003700 | transcription factor activity | [M] |
|  | GO:0006355 | regulation of transcription, DNA-dependent | [B] |
| - HTH\_AraC | GO:0003700 | transcription factor activity | [M] |
|  | GO:0043565 | sequence-specific DNA binding | [M] |
|  | GO:0006355 | regulation of transcription, DNA-dependent | [B] |
|  | GO:0005622 | intracellular | [C] |
| - HTH\_IclR | GO:0003677 | DNA binding | [M] |
|  | GO:0006355 | regulation of transcription, DNA-dependent | [B] |
| - HxlR |  |  |  |  |
| - IclR |  |  |  |  |
| - IDH | GO:0004450 | isocitrate dehydrogenase (NADP+) activity | [M] |
|  | GO:0006099 | tricarboxylic acid cycle | [B] |
|  | GO:0055114 | oxidation reduction | [B] |
| - IMS\_HHH |  |  |  |  |
| - iPGM\_N | GO:0004619 | phosphoglycerate mutase activity | [M] |
|  | GO:0030145 | manganese ion binding | [M] |
|  | GO:0006007 | glucose catabolic process | [B] |
|  | GO:0005737 | cytoplasm | [C] |
| - IucA\_IucC | GO:0015343 | siderophore-iron transmembrane transporter activity | [M] |
|  | GO:0019290 | siderophore biosynthetic process | [B] |
| - KIX | GO:0003712 | transcription cofactor activity | [M] |
|  | GO:0005515 | protein binding | [M] |
|  | GO:0006355 | regulation of transcription, DNA-dependent | [B] |
| - LacI | GO:0003700 | transcription factor activity | [M] |
|  | GO:0006355 | regulation of transcription, DNA-dependent | [B] |
|  | GO:0005622 | intracellular | [C] |
| - LAGLIDADG\_1 | GO:0003677 | DNA binding | [M] |
|  | GO:0004519 | endonuclease activity | [M] |
|  | GO:0006314 | intron homing | [B] |
| - Ldh\_2 | GO:0016491 | oxidoreductase activity | [M] |
|  | GO:0008152 | metabolic process | [B] |
|  | GO:0055114 | oxidation reduction | [B] |
| - LdpA\_C |  |  |  |  |
| - Lip\_A\_acyltrans | GO:0008415 | acyltransferase activity | [M] |
|  | GO:0009244 | lipopolysaccharide core region biosynthetic process | [B] |
|  | GO:0016021 | integral to membrane | [C] |
| - Lipoprotein\_9 |  |  |  |  |
| - LRR\_1 | GO:0005515 | protein binding | [M] |
| - LysE | GO:0006865 | amino acid transport | [B] |
|  | GO:0016020 | membrane | [C] |
| - LysR\_substrate |  |  |  |  |
| - LytTR |  |  |  |  |
| - M20\_dimer | GO:0016787 | hydrolase activity | [M] |
|  | GO:0046983 | protein dimerization activity | [M] |
| - MarR | GO:0003700 | transcription factor activity | [M] |
|  | GO:0006355 | regulation of transcription, DNA-dependent | [B] |
|  | GO:0005622 | intracellular | [C] |
| - MASE1 |  |  |  |  |
| - MCPsignal | GO:0004871 | signal transducer activity | [M] |
|  | GO:0006935 | chemotaxis | [B] |
|  | GO:0007165 | signal transduction | [B] |
|  | GO:0016020 | membrane | [C] |
| - MDMPI\_N |  |  |  |  |
| - Med13\_C |  |  |  |  |
| - MerR | GO:0003700 | transcription factor activity | [M] |
|  | GO:0006355 | regulation of transcription, DNA-dependent | [B] |
| - MerR-DNA-bind |  |  |  |  |
| - MerT | GO:0015097 | mercury ion transmembrane transporter activity | [M] |
|  | GO:0015694 | mercury ion transport | [B] |
|  | GO:0016020 | membrane | [C] |
| - Met\_synt\_B12 | GO:0008705 | methionine synthase activity | [M] |
|  | GO:0009086 | methionine biosynthetic process | [B] |
|  | GO:0005622 | intracellular | [C] |
| - MethyltransfD12 | GO:0009007 | site-specific DNA-methyltransferase (adenine-specific) activity | [M] |
|  | GO:0006306 | DNA methylation | [B] |
| - MetW |  |  |  |  |
| - MgtC | GO:0016020 | membrane | [C] |
| - MinC\_C |  |  |  |  |
| - MM\_CoA\_mutase | GO:0016866 | intramolecular transferase activity | [M] |
|  | GO:0031419 | cobalamin binding | [M] |
|  | GO:0008152 | metabolic process | [B] |
| - MmgE\_PrpD | GO:0047547 | 2-methylcitrate dehydratase activity | [M] |
|  | GO:0019543 | propionate catabolic process | [B] |
| - Molybdop\_Fe4S4 | GO:0016491 | oxidoreductase activity | [M] |
| - Molydop\_binding | GO:0016491 | oxidoreductase activity | [M] |
|  | GO:0030151 | molybdenum ion binding | [M] |
| - MotA\_ExbB | GO:0008565 | protein transporter activity | [M] |
|  | GO:0006810 | transport | [B] |
|  | GO:0016020 | membrane | [C] |
| - MreB\_Mbl | GO:0000902 | cell morphogenesis | [B] |
| - MurB\_C | GO:0008762 | UDP-N-acetylmuramate dehydrogenase activity | [M] |
|  | GO:0009252 | peptidoglycan biosynthetic process | [B] |
|  | GO:0055114 | oxidation reduction | [B] |
| - N\_methyl |  |  |  |  |
| - NACHT |  |  |  |  |
| - Neisseria\_PilC |  |  |  |  |
| - NikM |  |  |  |  |
| - NIT |  |  |  |  |
| - NLPC\_P60 |  |  |  |  |
| - NMT1 |  |  |  |  |
| - NrfD |  |  |  |  |
| - Nucleoplasmin | GO:0003676 | nucleic acid binding | [M] |
| - OATP | GO:0005215 | transporter activity | [M] |
|  | GO:0006810 | transport | [B] |
|  | GO:0016020 | membrane | [C] |
| - OEP | GO:0005215 | transporter activity | [M] |
|  | GO:0006810 | transport | [B] |
| - OKR\_DC\_1 | GO:0003824 | catalytic activity | [M] |
| - OKR\_DC\_1\_C | GO:0003824 | catalytic activity | [M] |
| - oligo\_HPY | GO:0000166 | nucleotide binding | [M] |
|  | GO:0005524 | ATP binding | [M] |
|  | GO:0015833 | peptide transport | [B] |
| - OmpA | GO:0009279 | cell outer membrane | [C] |
| - OmpW | GO:0019867 | outer membrane | [C] |
| - OprB | GO:0005215 | transporter activity | [M] |
|  | GO:0006810 | transport | [B] |
|  | GO:0016021 | integral to membrane | [C] |
| - OstA\_C | GO:0010033 | response to organic substance | [B] |
|  | GO:0016044 | membrane organization | [B] |
|  | GO:0019867 | outer membrane | [C] |
| - PaaA\_PaaC |  |  |  |  |
| - PAS\_4 |  |  |  |  |
| - PdxA | GO:0050570 | 4-hydroxythreonine-4-phosphate dehydrogenase activity | [M] |
|  | GO:0051287 | NAD or NADH binding | [M] |
|  | GO:0008615 | pyridoxine biosynthetic process | [B] |
|  | GO:0055114 | oxidation reduction | [B] |
| - PEGA |  |  |  |  |
| - Penicil\_amidase | GO:0016787 | hydrolase activity | [M] |
|  | GO:0017000 | antibiotic biosynthetic process | [B] |
| - PEPCK | GO:0004611 | phosphoenolpyruvate carboxykinase activity | [M] |
|  | GO:0005525 | GTP binding | [M] |
|  | GO:0006094 | gluconeogenesis | [B] |
| - Peptidase\_A3 | GO:0004190 | aspartic-type endopeptidase activity | [M] |
|  | GO:0006508 | proteolysis | [B] |
| - Peptidase\_A8 | GO:0004190 | aspartic-type endopeptidase activity | [M] |
|  | GO:0006508 | proteolysis | [B] |
|  | GO:0016020 | membrane | [C] |
| - Peptidase\_M15\_3 |  |  |  |  |
| - Peptidase\_M23 |  |  |  |  |
| - Peptidase\_S11 | GO:0009002 | serine-type D-Ala-D-Ala carboxypeptidase activity | [M] |
|  | GO:0006508 | proteolysis | [B] |
| - Peptidase\_S13 | GO:0004185 | serine-type carboxypeptidase activity | [M] |
|  | GO:0006508 | proteolysis | [B] |
| - Peptidase\_S49\_N | GO:0004252 | serine-type endopeptidase activity | [M] |
|  | GO:0005886 | plasma membrane | [C] |
| - Peptidase\_S58 |  |  |  |  |
| - Peptidase\_S66 |  |  |  |  |
| - PepX\_C | GO:0008239 | dipeptidyl-peptidase activity | [M] |
| - Peripla\_BP\_2 | GO:0005381 | iron ion transmembrane transporter activity | [M] |
|  | GO:0006827 | high-affinity iron ion transport | [B] |
| - PhaC\_N | GO:0008415 | acyltransferase activity | [M] |
|  | GO:0042619 | poly-hydroxybutyrate biosynthetic process | [B] |
| - PhnA |  |  |  |  |
| - PhoU\_div |  |  |  |  |
| - Pilin | GO:0007155 | cell adhesion | [B] |
|  | GO:0009289 | pilus | [C] |
| - PilN |  |  |  |  |
| - PilP |  |  |  |  |
| - PLA1 | GO:0004620 | phospholipase activity | [M] |
|  | GO:0006629 | lipid metabolic process | [B] |
|  | GO:0016020 | membrane | [C] |
| - Plug | GO:0004872 | receptor activity | [M] |
|  | GO:0005215 | transporter activity | [M] |
|  | GO:0006810 | transport | [B] |
|  | GO:0016020 | membrane | [C] |
| - PMC2NT | GO:0006396 | RNA processing | [B] |
|  | GO:0000176 | nuclear exosome (RNase complex) | [C] |
| - PNTB | GO:0008746 | NAD(P) transhydrogenase activity | [M] |
|  | GO:0055114 | oxidation reduction | [B] |
| - Polysacc\_synt | GO:0000271 | polysaccharide biosynthetic process | [B] |
|  | GO:0016020 | membrane | [C] |
| - Polysacc\_synt\_2 | GO:0009058 | biosynthetic process | [B] |
| - POR | GO:0016903 | oxidoreductase activity, acting on the aldehyde or oxo group of donors | [M] |
|  | GO:0055114 | oxidation reduction | [B] |
| - Porin\_1 | GO:0005215 | transporter activity | [M] |
|  | GO:0006810 | transport | [B] |
|  | GO:0016020 | membrane | [C] |
| - POTRA\_2 |  |  |  |  |
| - PP\_kinase | GO:0008976 | polyphosphate kinase activity | [M] |
|  | GO:0006799 | polyphosphate biosynthetic process | [B] |
|  | GO:0009358 | polyphosphate kinase complex | [C] |
| - PPK2 |  |  |  |  |
| - PPO1\_DWL |  |  |  |  |
| - PPO1\_KFDV |  |  |  |  |
| - PPV\_E1\_C | GO:0003677 | DNA binding | [M] |
|  | GO:0004003 | ATP-dependent DNA helicase activity | [M] |
|  | GO:0005524 | ATP binding | [M] |
|  | GO:0006260 | DNA replication | [B] |
| - PqiA |  |  |  |  |
| - PrpF |  |  |  |  |
| - PTA\_PTB | GO:0008415 | acyltransferase activity | [M] |
|  | GO:0008152 | metabolic process | [B] |
| - PTB |  |  |  |  |
| - PTS-HPr | GO:0005351 | sugar:hydrogen symporter activity | [M] |
|  | GO:0009401 | phosphoenolpyruvate-dependent sugar phosphotransferase system | [B] |
| - PTS\_EIIA\_2 | GO:0005351 | sugar:hydrogen symporter activity | [M] |
|  | GO:0006810 | transport | [B] |
|  | GO:0009401 | phosphoenolpyruvate-dependent sugar phosphotransferase system | [B] |
| - PTS\_EIIC | GO:0005351 | sugar:hydrogen symporter activity | [M] |
|  | GO:0008982 | protein-N(PI)-phosphohistidine-sugar phosphotransferase activity | [M] |
|  | GO:0009401 | phosphoenolpyruvate-dependent sugar phosphotransferase system | [B] |
|  | GO:0016020 | membrane | [C] |
| - Queuosine\_synth | GO:0016740 | transferase activity | [M] |
|  | GO:0016853 | isomerase activity | [M] |
|  | GO:0008616 | queuosine biosynthetic process | [B] |
| - RdgC | GO:0006310 | DNA recombination | [B] |
| - Rep\_3 | GO:0003887 | DNA-directed DNA polymerase activity | [M] |
|  | GO:0006270 | DNA replication initiation | [B] |
|  | GO:0005727 | extrachromosomal circular DNA | [C] |
| - Resolvase | GO:0000150 | recombinase activity | [M] |
|  | GO:0003677 | DNA binding | [M] |
|  | GO:0006310 | DNA recombination | [B] |
| - Ret\_tiss |  |  |  |  |
| - RHS |  |  |  |  |
| - Ribonuclease\_BN | GO:0004540 | ribonuclease activity | [M] |
| - Rif1\_N |  |  |  |  |
| - RIP | GO:0030598 | rRNA N-glycosylase activity | [M] |
|  | GO:0017148 | negative regulation of translation | [B] |
| - RmuC |  |  |  |  |
| - Robl\_LC7 |  |  |  |  |
| - RuvB\_N | GO:0009378 | four-way junction helicase activity | [M] |
|  | GO:0006281 | DNA repair | [B] |
|  | GO:0006310 | DNA recombination | [B] |
| - SAB | GO:0008092 | cytoskeletal protein binding | [M] |
|  | GO:0030866 | cortical actin cytoskeleton organization | [B] |
|  | GO:0005856 | cytoskeleton | [C] |
| - SBF2 |  |  |  |  |
| - SBP\_bac\_5 | GO:0005215 | transporter activity | [M] |
|  | GO:0006810 | transport | [B] |
| - SBP\_bac\_7 | GO:0006810 | transport | [B] |
|  | GO:0030288 | outer membrane-bounded periplasmic space | [C] |
| - SDF | GO:0017153 | sodium:dicarboxylate symporter activity | [M] |
|  | GO:0006835 | dicarboxylic acid transport | [B] |
|  | GO:0016020 | membrane | [C] |
| - SDH\_alpha | GO:0003941 | L-serine ammonia-lyase activity | [M] |
|  | GO:0051539 | 4 iron, 4 sulfur cluster binding | [M] |
|  | GO:0006094 | gluconeogenesis | [B] |
| - SDH\_beta | GO:0003941 | L-serine ammonia-lyase activity | [M] |
|  | GO:0051539 | 4 iron, 4 sulfur cluster binding | [M] |
|  | GO:0006094 | gluconeogenesis | [B] |
| - Sec\_GG | GO:0008565 | protein transporter activity | [M] |
|  | GO:0015628 | protein secretion by the type II secretion system | [B] |
|  | GO:0015627 | type II protein secretion system complex | [C] |
| - SecB | GO:0051082 | unfolded protein binding | [M] |
|  | GO:0015031 | protein transport | [B] |
|  | GO:0051262 | protein tetramerization | [B] |
| - SecD\_SecF | GO:0008565 | protein transporter activity | [M] |
|  | GO:0015628 | protein secretion by the type II secretion system | [B] |
|  | GO:0015627 | type II protein secretion system complex | [C] |
| - Secretin | GO:0009306 | protein secretion | [B] |
| - Secretin\_N |  |  |  |  |
| - SHQ1 |  |  |  |  |
| - Sigma54\_activat | GO:0005524 | ATP binding | [M] |
|  | GO:0008134 | transcription factor binding | [M] |
|  | GO:0006355 | regulation of transcription, DNA-dependent | [B] |
|  | GO:0005622 | intracellular | [C] |
| - Sigma70\_ner | GO:0003677 | DNA binding | [M] |
|  | GO:0003700 | transcription factor activity | [M] |
|  | GO:0016987 | sigma factor activity | [M] |
|  | GO:0006352 | transcription initiation | [B] |
|  | GO:0006355 | regulation of transcription, DNA-dependent | [B] |
| - Sigma70\_r1\_1 | GO:0003677 | DNA binding | [M] |
|  | GO:0030528 | transcription regulator activity | [M] |
|  | GO:0045449 | regulation of transcription | [B] |
| - Sigma70\_r4\_2 | GO:0003677 | DNA binding | [M] |
|  | GO:0003700 | transcription factor activity | [M] |
|  | GO:0016987 | sigma factor activity | [M] |
|  | GO:0006352 | transcription initiation | [B] |
|  | GO:0006355 | regulation of transcription, DNA-dependent | [B] |
| - SMC\_hinge | GO:0005515 | protein binding | [M] |
|  | GO:0005524 | ATP binding | [M] |
|  | GO:0051276 | chromosome organization | [B] |
|  | GO:0005694 | chromosome | [C] |
| - SMN | GO:0003723 | RNA binding | [M] |
|  | GO:0000245 | spliceosome assembly | [B] |
|  | GO:0006397 | mRNA processing | [B] |
|  | GO:0005634 | nucleus | [C] |
|  | GO:0005737 | cytoplasm | [C] |
| - SnoaL |  |  |  |  |
| - SpoA |  |  |  |  |
| - Sterol-sensing |  |  |  |  |
| - Sugar-bind | GO:0030246 | carbohydrate binding | [M] |
|  | GO:0030528 | transcription regulator activity | [M] |
| - SurA\_N |  |  |  |  |
| - Tannase |  |  |  |  |
| - Terminase\_4 |  |  |  |  |
| - Terminase\_GpA |  |  |  |  |
| - TetR\_C\_2 | GO:0003677 | DNA binding | [M] |
| - TetR\_N | GO:0003700 | transcription factor activity | [M] |
|  | GO:0006355 | regulation of transcription, DNA-dependent | [B] |
| - TFIIE\_beta | GO:0003702 | RNA polymerase II transcription factor activity | [M] |
|  | GO:0006367 | transcription initiation from RNA polymerase II promoter | [B] |
|  | GO:0005673 | transcription factor TFIIE complex | [C] |
| - TGT | GO:0008479 | queuine tRNA-ribosyltransferase activity | [M] |
|  | GO:0006400 | tRNA modification | [B] |
|  | GO:0008616 | queuosine biosynthetic process | [B] |
| - ThiG | GO:0009228 | thiamin biosynthetic process | [B] |
| - Thioesterase | GO:0016788 | hydrolase activity, acting on ester bonds | [M] |
|  | GO:0009058 | biosynthetic process | [B] |
| - TNFR\_c6 | GO:0004872 | receptor activity | [M] |
| - TniB |  |  |  |  |
| - TniQ |  |  |  |  |
| - TOBE\_2 | GO:0005215 | transporter activity | [M] |
|  | GO:0005524 | ATP binding | [M] |
|  | GO:0016820 | hydrolase activity, acting on acid anhydrides, catalyzing transmembrane movement of substances | [M] |
|  | GO:0006810 | transport | [B] |
|  | GO:0043190 | ATP-binding cassette (ABC) transporter complex | [C] |
| - TolB\_N | GO:0015031 | protein transport | [B] |
|  | GO:0042597 | periplasmic space | [C] |
| - TonB | GO:0005381 | iron ion transmembrane transporter activity | [M] |
|  | GO:0006826 | iron ion transport | [B] |
|  | GO:0030288 | outer membrane-bounded periplasmic space | [C] |
| - TonB\_dep\_Rec | GO:0004872 | receptor activity | [M] |
|  | GO:0005215 | transporter activity | [M] |
|  | GO:0006810 | transport | [B] |
|  | GO:0016020 | membrane | [C] |
| - Trans\_reg\_C | GO:0000156 | two-component response regulator activity | [M] |
|  | GO:0003677 | DNA binding | [M] |
|  | GO:0000160 | two-component signal transduction system (phosphorelay) | [B] |
|  | GO:0006355 | regulation of transcription, DNA-dependent | [B] |
| - Transgly | GO:0003824 | catalytic activity | [M] |
|  | GO:0009252 | peptidoglycan biosynthetic process | [B] |
|  | GO:0009274 | peptidoglycan-based cell wall | [C] |
| - Transpeptidase | GO:0008658 | penicillin binding | [M] |
|  | GO:0009273 | peptidoglycan-based cell wall biogenesis | [B] |
| - TraU |  |  |  |  |
| - TrbI | GO:0009291 | unidirectional conjugation | [B] |
| - Tropomyosin |  |  |  |  |
| - Trp\_halogenase |  |  |  |  |
| - Tyrosinase | GO:0016491 | oxidoreductase activity | [M] |
|  | GO:0008152 | metabolic process | [B] |
| - UPF0060 | GO:0016020 | membrane | [C] |
| - UPF0075 | GO:0005524 | ATP binding | [M] |
|  | GO:0016773 | phosphotransferase activity, alcohol group as acceptor | [M] |
|  | GO:0006040 | amino sugar metabolic process | [B] |
|  | GO:0009254 | peptidoglycan turnover | [B] |
| - UPF0149 |  |  |  |  |
| - Urocanase | GO:0016153 | urocanate hydratase activity | [M] |
|  | GO:0006548 | histidine catabolic process | [B] |
| - UTRA | GO:0003677 | DNA binding | [M] |
|  | GO:0030528 | transcription regulator activity | [M] |
|  | GO:0045449 | regulation of transcription | [B] |
| - UvrC\_HhH\_N | GO:0003677 | DNA binding | [M] |
|  | GO:0004518 | nuclease activity | [M] |
|  | GO:0006289 | nucleotide-excision repair | [B] |
| - XkdW |  |  |  |  |
| - YaeQ |  |  |  |  |
| - YceI |  |  |  |  |
| - YhhN | GO:0016021 | integral to membrane | [C] |
| - YHS |  |  |  |  |
| - zf-H2C2 |  |  |  |  |

---

## Archaeplastida [eol|tol]

|  |  |  |  |
| --- | --- | --- | --- |
| **Pfam domain(s)** | **GO term acc** | **GO term** | **GO namespace** |
| - 3-PAP |  |  |  |  |
| - 7tm\_1 | GO:0007186 | G-protein coupled receptor protein signaling pathway | [B] |
|  | GO:0016021 | integral to membrane | [C] |
| - 7tm\_2 | GO:0004930 | G-protein coupled receptor activity | [M] |
|  | GO:0007186 | G-protein coupled receptor protein signaling pathway | [B] |
|  | GO:0016020 | membrane | [C] |
| - 7tm\_3 | GO:0004930 | G-protein coupled receptor activity | [M] |
|  | GO:0007186 | G-protein coupled receptor protein signaling pathway | [B] |
|  | GO:0016021 | integral to membrane | [C] |
| - 7TM\_GPCR\_Srsx |  |  |  |  |
| - AAA\_4 | GO:0005524 | ATP binding | [M] |
| - ADP\_PFK\_GK | GO:0016773 | phosphotransferase activity, alcohol group as acceptor | [M] |
|  | GO:0005975 | carbohydrate metabolic process | [B] |
| - Ala\_racemase\_C | GO:0008784 | alanine racemase activity | [M] |
|  | GO:0006522 | alanine metabolic process | [B] |
| - Allantoicase | GO:0004037 | allantoicase activity | [M] |
| - Amastin |  |  |  |  |
| - Amidoligase\_2 |  |  |  |  |
| - ANATO | GO:0005576 | extracellular region | [C] |
| - Antistasin | GO:0004867 | serine-type endopeptidase inhibitor activity | [M] |
| - APC\_CDC26 |  |  |  |  |
| - ArabFuran-catal | GO:0046556 | alpha-N-arabinofuranosidase activity | [M] |
|  | GO:0031221 | arabinan metabolic process | [B] |
| - AraC\_E\_bind |  |  |  |  |
| - ART | GO:0003956 | NAD(P)+-protein-arginine ADP-ribosyltransferase activity | [M] |
|  | GO:0006471 | protein amino acid ADP-ribosylation | [B] |
| - Arylsulfotrans |  |  |  |  |
| - AsnA | GO:0004071 | aspartate-ammonia ligase activity | [M] |
|  | GO:0006529 | asparagine biosynthetic process | [B] |
|  | GO:0005737 | cytoplasm | [C] |
| - Astacin | GO:0004222 | metalloendopeptidase activity | [M] |
|  | GO:0006508 | proteolysis | [B] |
| - AstE\_AspA | GO:0016788 | hydrolase activity, acting on ester bonds | [M] |
|  | GO:0008152 | metabolic process | [B] |
| - ATP-gua\_Ptrans | GO:0016301 | kinase activity | [M] |
|  | GO:0016772 | transferase activity, transferring phosphorus-containing groups | [M] |
| - ATP-gua\_PtransN | GO:0016301 | kinase activity | [M] |
|  | GO:0016772 | transferase activity, transferring phosphorus-containing groups | [M] |
| - ATP\_Ca\_trans\_C |  |  |  |  |
| - Bac\_GDH |  |  |  |  |
| - BAF | GO:0003677 | DNA binding | [M] |
| - BCS1\_N |  |  |  |  |
| - BioY |  |  |  |  |
| - Borealin |  |  |  |  |
| - Borrelia\_orfA |  |  |  |  |
| - BrkDBD |  |  |  |  |
| - BTG |  |  |  |  |
| - But2 |  |  |  |  |
| - Bvg\_acc\_factor | GO:0016563 | transcription activator activity | [M] |
|  | GO:0045941 | positive regulation of transcription | [B] |
| - Cadherin | GO:0005509 | calcium ion binding | [M] |
|  | GO:0007156 | homophilic cell adhesion | [B] |
|  | GO:0016020 | membrane | [C] |
| - Caldesmon |  |  |  |  |
| - Calsequestrin | GO:0005509 | calcium ion binding | [M] |
| - Carn\_acyltransf | GO:0008415 | acyltransferase activity | [M] |
| - CAT | GO:0008811 | chloramphenicol O-acetyltransferase activity | [M] |
| - CathepsinC\_exc |  |  |  |  |
| - CBM\_2 | GO:0004553 | hydrolase activity, hydrolyzing O-glycosyl compounds | [M] |
|  | GO:0030246 | carbohydrate binding | [M] |
|  | GO:0005975 | carbohydrate metabolic process | [B] |
| - CBM\_5\_12 | GO:0004553 | hydrolase activity, hydrolyzing O-glycosyl compounds | [M] |
|  | GO:0030246 | carbohydrate binding | [M] |
|  | GO:0005975 | carbohydrate metabolic process | [B] |
|  | GO:0005576 | extracellular region | [C] |
| - CD225 | GO:0009607 | response to biotic stimulus | [B] |
|  | GO:0016021 | integral to membrane | [C] |
| - CENP-H | GO:0005515 | protein binding | [M] |
|  | GO:0043515 | kinetochore binding | [M] |
|  | GO:0007059 | chromosome segregation | [B] |
|  | GO:0051301 | cell division | [B] |
|  | GO:0000777 | condensed chromosome kinetochore | [C] |
|  | GO:0005634 | nucleus | [C] |
| - Chagasin\_I42 |  |  |  |  |
| - CHAP |  |  |  |  |
| - CHGN | GO:0016758 | transferase activity, transferring hexosyl groups | [M] |
|  | GO:0032580 | Golgi cisterna membrane | [C] |
| - Chitin\_synth\_2 | GO:0016758 | transferase activity, transferring hexosyl groups | [M] |
| - Cir\_Bir\_Yir |  |  |  |  |
| - CLAG |  |  |  |  |
| - Clusterin | GO:0008219 | cell death | [B] |
| - CmcH\_NodU | GO:0003824 | catalytic activity | [M] |
|  | GO:0009058 | biosynthetic process | [B] |
| - Cna\_B |  |  |  |  |
| - COX4 | GO:0004129 | cytochrome-c oxidase activity | [M] |
| - CPW\_WPC |  |  |  |  |
| - CRT10 |  |  |  |  |
| - Crystall |  |  |  |  |
| - Cytokin\_check\_N |  |  |  |  |
| - DASH\_Ask1 |  |  |  |  |
| - DASH\_Dad1 |  |  |  |  |
| - DASH\_Dad2 |  |  |  |  |
| - DASH\_Dad4 |  |  |  |  |
| - DASH\_Dam1 |  |  |  |  |
| - DCX | GO:0007242 | intracellular signaling cascade | [B] |
| - DeoC | GO:0016829 | lyase activity | [M] |
| - Desulfoferrodox | GO:0005506 | iron ion binding | [M] |
|  | GO:0016491 | oxidoreductase activity | [M] |
|  | GO:0055114 | oxidation reduction | [B] |
| - DGCR6 |  |  |  |  |
| - Dickkopf\_N | GO:0007275 | multicellular organismal development | [B] |
|  | GO:0030178 | negative regulation of Wnt receptor signaling pathway | [B] |
|  | GO:0005576 | extracellular region | [C] |
| - DIX | GO:0004871 | signal transducer activity | [M] |
|  | GO:0007275 | multicellular organismal development | [B] |
|  | GO:0005622 | intracellular | [C] |
| - DNA\_ligase\_IV |  |  |  |  |
| - Dpy19 |  |  |  |  |
| - Drc1-Sld2 |  |  |  |  |
| - Drf\_FH3 | GO:0003779 | actin binding | [M] |
|  | GO:0016043 | cellular component organization | [B] |
| - DTHCT | GO:0003677 | DNA binding | [M] |
|  | GO:0003918 | DNA topoisomerase (ATP-hydrolyzing) activity | [M] |
|  | GO:0005524 | ATP binding | [M] |
|  | GO:0005634 | nucleus | [C] |
| - DUF1008 |  |  |  |  |
| - DUF1023 |  |  |  |  |
| - DUF1045 |  |  |  |  |
| - DUF1057 |  |  |  |  |
| - DUF108 | GO:0016491 | oxidoreductase activity | [M] |
|  | GO:0006742 | NADP catabolic process | [B] |
|  | GO:0019363 | pyridine nucleotide biosynthetic process | [B] |
|  | GO:0055114 | oxidation reduction | [B] |
| - DUF1083 | GO:0004553 | hydrolase activity, hydrolyzing O-glycosyl compounds | [M] |
|  | GO:0030246 | carbohydrate binding | [M] |
|  | GO:0016052 | carbohydrate catabolic process | [B] |
| - DUF1208 |  |  |  |  |
| - DUF126 |  |  |  |  |
| - DUF1289 |  |  |  |  |
| - DUF1349 |  |  |  |  |
| - DUF1394 |  |  |  |  |
| - DUF1445 |  |  |  |  |
| - DUF1485 |  |  |  |  |
| - DUF1565 |  |  |  |  |
| - DUF1688 |  |  |  |  |
| - DUF1697 |  |  |  |  |
| - DUF1704 |  |  |  |  |
| - DUF1729 |  |  |  |  |
| - DUF1742 |  |  |  |  |
| - DUF1765 |  |  |  |  |
| - DUF1783 |  |  |  |  |
| - DUF1810 |  |  |  |  |
| - DUF1899 |  |  |  |  |
| - DUF19 |  |  |  |  |
| - DUF1900 |  |  |  |  |
| - DUF1989 |  |  |  |  |
| - DUF1996 |  |  |  |  |
| - DUF2003 |  |  |  |  |
| - DUF2013 |  |  |  |  |
| - DUF2051 |  |  |  |  |
| - DUF208 |  |  |  |  |
| - DUF2183 |  |  |  |  |
| - DUF2228 |  |  |  |  |
| - DUF2233 |  |  |  |  |
| - DUF2236 |  |  |  |  |
| - DUF2263 |  |  |  |  |
| - DUF2347 |  |  |  |  |
| - DUF2355 |  |  |  |  |
| - DUF2362 |  |  |  |  |
| - DUF2368 |  |  |  |  |
| - DUF2399 |  |  |  |  |
| - DUF2401 |  |  |  |  |
| - DUF2403 |  |  |  |  |
| - DUF2407 |  |  |  |  |
| - DUF2411 |  |  |  |  |
| - DUF2423 |  |  |  |  |
| - DUF2424 |  |  |  |  |
| - DUF2432 |  |  |  |  |
| - DUF2435 |  |  |  |  |
| - DUF2452 |  |  |  |  |
| - DUF2464 |  |  |  |  |
| - DUF2475 |  |  |  |  |
| - DUF2615 |  |  |  |  |
| - DUF2638 |  |  |  |  |
| - DUF268 |  |  |  |  |
| - DUF2778 |  |  |  |  |
| - DUF2779 |  |  |  |  |
| - DUF2780 |  |  |  |  |
| - DUF2786 |  |  |  |  |
| - DUF2804 |  |  |  |  |
| - DUF2817 |  |  |  |  |
| - DUF2961 |  |  |  |  |
| - DUF3011 |  |  |  |  |
| - DUF3161 |  |  |  |  |
| - DUF3184 |  |  |  |  |
| - DUF3237 |  |  |  |  |
| - DUF3294 |  |  |  |  |
| - DUF3384 |  |  |  |  |
| - DUF3402 |  |  |  |  |
| - DUF3469 |  |  |  |  |
| - DUF3494 |  |  |  |  |
| - DUF3500 |  |  |  |  |
| - DUF3608 |  |  |  |  |
| - DUF3684 |  |  |  |  |
| - DUF3704 |  |  |  |  |
| - DUF3712 |  |  |  |  |
| - DUF3767 |  |  |  |  |
| - DUF521 |  |  |  |  |
| - DUF548 |  |  |  |  |
| - DUF582 |  |  |  |  |
| - DUF583 |  |  |  |  |
| - DUF606 |  |  |  |  |
| - DUF692 |  |  |  |  |
| - DUF748 |  |  |  |  |
| - DUF772 |  |  |  |  |
| - DUF781 |  |  |  |  |
| - DUF820 |  |  |  |  |
| - DUF837 |  |  |  |  |
| - DUF849 |  |  |  |  |
| - DUF853 |  |  |  |  |
| - DUF885 |  |  |  |  |
| - DUF912 |  |  |  |  |
| - Dynactin\_p22 |  |  |  |  |
| - Dynactin\_p62 |  |  |  |  |
| - Dynamitin | GO:0007017 | microtubule-based process | [B] |
|  | GO:0005869 | dynactin complex | [C] |
| - Ecotin |  |  |  |  |
| - Ectoine\_synth | GO:0016836 | hydro-lyase activity | [M] |
|  | GO:0006596 | polyamine biosynthetic process | [B] |
| - efhand\_Ca\_insen |  |  |  |  |
| - ELH | GO:0005179 | hormone activity | [M] |
|  | GO:0007275 | multicellular organismal development | [B] |
|  | GO:0005576 | extracellular region | [C] |
| - ELL | GO:0016944 | RNA polymerase II transcription elongation factor activity | [M] |
|  | GO:0006368 | RNA elongation from RNA polymerase II promoter | [B] |
|  | GO:0008023 | transcription elongation factor complex | [C] |
| - Endonuclease\_7 | GO:0004519 | endonuclease activity | [M] |
| - Erf4 |  |  |  |  |
| - ETRAMP |  |  |  |  |
| - EutQ |  |  |  |  |
| - F1F0-ATPsyn\_F |  |  |  |  |
| - FCH |  |  |  |  |
| - FIBP | GO:0017134 | fibroblast growth factor binding | [M] |
| - Fibrinogen\_BP |  |  |  |  |
| - Fibrinogen\_C | GO:0005102 | receptor binding | [M] |
|  | GO:0007165 | signal transduction | [B] |
| - Filament |  |  |  |  |
| - FLYWCH |  |  |  |  |
| - fn2 |  |  |  |  |
| - Fork\_head\_N |  |  |  |  |
| - Frizzled | GO:0004926 | non-G-protein coupled 7TM receptor activity | [M] |
|  | GO:0007166 | cell surface receptor linked signal transduction | [B] |
|  | GO:0016020 | membrane | [C] |
| - FTCD\_C | GO:0003824 | catalytic activity | [M] |
|  | GO:0044237 | cellular metabolic process | [B] |
| - GCR1\_C |  |  |  |  |
| - GDE\_C | GO:0004135 | amylo-alpha-1,6-glucosidase activity | [M] |
|  | GO:0005978 | glycogen biosynthetic process | [B] |
| - Git3 |  |  |  |  |
| - Glutaminase | GO:0004359 | glutaminase activity | [M] |
|  | GO:0006541 | glutamine metabolic process | [B] |
| - Glyco\_hydro\_12 | GO:0008810 | cellulase activity | [M] |
|  | GO:0000272 | polysaccharide catabolic process | [B] |
| - Glyco\_hydro\_30 | GO:0004348 | glucosylceramidase activity | [M] |
|  | GO:0006665 | sphingolipid metabolic process | [B] |
|  | GO:0007040 | lysosome organization | [B] |
|  | GO:0005764 | lysosome | [C] |
| - Glyco\_hydro\_39 | GO:0004553 | hydrolase activity, hydrolyzing O-glycosyl compounds | [M] |
|  | GO:0005975 | carbohydrate metabolic process | [B] |
| - Glyco\_hydro\_45 | GO:0008810 | cellulase activity | [M] |
|  | GO:0005975 | carbohydrate metabolic process | [B] |
| - Glyco\_hydro\_49 |  |  |  |  |
| - Glyco\_hydro\_6 | GO:0004553 | hydrolase activity, hydrolyzing O-glycosyl compounds | [M] |
|  | GO:0030245 | cellulose catabolic process | [B] |
| - Glyco\_hydro\_61 |  |  |  |  |
| - Glyco\_hydro\_67C | GO:0046559 | alpha-glucuronidase activity | [M] |
|  | GO:0045493 | xylan catabolic process | [B] |
|  | GO:0005576 | extracellular region | [C] |
| - Glyco\_hydro\_67M | GO:0046559 | alpha-glucuronidase activity | [M] |
|  | GO:0045493 | xylan catabolic process | [B] |
|  | GO:0005576 | extracellular region | [C] |
| - Glyco\_hydro\_7 | GO:0004553 | hydrolase activity, hydrolyzing O-glycosyl compounds | [M] |
|  | GO:0005975 | carbohydrate metabolic process | [B] |
| - Glyco\_hydro\_72 |  |  |  |  |
| - Glyco\_hydro\_8 | GO:0004553 | hydrolase activity, hydrolyzing O-glycosyl compounds | [M] |
|  | GO:0005975 | carbohydrate metabolic process | [B] |
| - Glyco\_hydro\_92 |  |  |  |  |
| - Glycophorin\_A | GO:0016021 | integral to membrane | [C] |
| - GPS | GO:0007218 | neuropeptide signaling pathway | [B] |
|  | GO:0016020 | membrane | [C] |
| - GRAB |  |  |  |  |
| - GRASP55\_65 |  |  |  |  |
| - GSP\_synth |  |  |  |  |
| - GTP\_CH\_N |  |  |  |  |
| - GtrA | GO:0000271 | polysaccharide biosynthetic process | [B] |
|  | GO:0006810 | transport | [B] |
|  | GO:0016021 | integral to membrane | [C] |
| - HIN |  |  |  |  |
| - HOOK | GO:0008017 | microtubule binding | [M] |
|  | GO:0000226 | microtubule cytoskeleton organization | [B] |
|  | GO:0005737 | cytoplasm | [C] |
| - HtrL\_YibB |  |  |  |  |
| - HutD |  |  |  |  |
| - I-set |  |  |  |  |
| - IF\_tail |  |  |  |  |
| - IMD | GO:0008093 | cytoskeletal adaptor activity | [M] |
|  | GO:0017124 | SH3 domain binding | [M] |
|  | GO:0007165 | signal transduction | [B] |
|  | GO:0046847 | filopodium assembly | [B] |
| - Inos-1-P\_synth |  |  |  |  |
| - Integrase | GO:0003676 | nucleic acid binding | [M] |
|  | GO:0008907 | integrase activity | [M] |
| - Integrase\_Zn | GO:0003677 | DNA binding | [M] |
|  | GO:0008270 | zinc ion binding | [M] |
|  | GO:0008907 | integrase activity | [M] |
|  | GO:0015074 | DNA integration | [B] |
| - Interfer-bind |  |  |  |  |
| - IpgD | GO:0016791 | phosphatase activity | [M] |
|  | GO:0009405 | pathogenesis | [B] |
| - IstB | GO:0005524 | ATP binding | [M] |
| - KAP\_NTPase |  |  |  |  |
| - KIF1B |  |  |  |  |
| - Lactate\_perm | GO:0015129 | lactate transmembrane transporter activity | [M] |
|  | GO:0015727 | lactate transport | [B] |
| - LacY\_symp | GO:0006810 | transport | [B] |
|  | GO:0016020 | membrane | [C] |
| - LamB\_YcsF |  |  |  |  |
| - Lectin\_leg-like | GO:0016020 | membrane | [C] |
| - LEH |  |  |  |  |
| - Leu\_Phe\_trans | GO:0008914 | leucyltransferase activity | [M] |
|  | GO:0030163 | protein catabolic process | [B] |
| - Lig\_chan-Glu\_bd |  |  |  |  |
| - Lip\_prot\_lig\_C |  |  |  |  |
| - Lipase |  |  |  |  |
| - Lipocalin | GO:0005488 | binding | [M] |
| - LRRNT |  |  |  |  |
| - LtrA |  |  |  |  |
| - LTV |  |  |  |  |
| - MAM | GO:0016020 | membrane | [C] |
| - MAPKK1\_Int |  |  |  |  |
| - MAR\_sialic\_bdg |  |  |  |  |
| - MBT | GO:0045449 | regulation of transcription | [B] |
|  | GO:0005634 | nucleus | [C] |
| - Med1 |  |  |  |  |
| - Med15 |  |  |  |  |
| - Med2 |  |  |  |  |
| - Mei5 |  |  |  |  |
| - META |  |  |  |  |
| - MG1 |  |  |  |  |
| - MHYT |  |  |  |  |
| - Mif2 |  |  |  |  |
| - MIG-14\_Wnt-bd |  |  |  |  |
| - Mis12\_component |  |  |  |  |
| - MitoNEET\_N | GO:0051537 | 2 iron, 2 sulfur cluster binding | [M] |
|  | GO:0043231 | intracellular membrane-bounded organelle | [C] |
| - MKT1\_C |  |  |  |  |
| - MKT1\_N |  |  |  |  |
| - MMS1 |  |  |  |  |
| - MOFRL |  |  |  |  |
| - MVP\_shoulder |  |  |  |  |
| - Myb\_DNA-bind\_2 |  |  |  |  |
| - N1221 |  |  |  |  |
| - Na\_H\_antiporter | GO:0015385 | sodium:hydrogen antiporter activity | [M] |
|  | GO:0006814 | sodium ion transport | [B] |
|  | GO:0006885 | regulation of pH | [B] |
|  | GO:0016021 | integral to membrane | [C] |
| - NADH\_ub\_rd\_NUML |  |  |  |  |
| - NARG2\_C |  |  |  |  |
| - Nha1\_C | GO:0015385 | sodium:hydrogen antiporter activity | [M] |
|  | GO:0006814 | sodium ion transport | [B] |
|  | GO:0016020 | membrane | [C] |
| - NHase\_alpha | GO:0003824 | catalytic activity | [M] |
|  | GO:0046914 | transition metal ion binding | [M] |
|  | GO:0006807 | nitrogen compound metabolic process | [B] |
| - NHase\_beta | GO:0018822 | nitrile hydratase activity | [M] |
|  | GO:0046914 | transition metal ion binding | [M] |
|  | GO:0006807 | nitrogen compound metabolic process | [B] |
| - Ni\_hydr\_CYTB | GO:0009055 | electron carrier activity | [M] |
|  | GO:0016021 | integral to membrane | [C] |
| - NIF3 |  |  |  |  |
| - NodZ | GO:0016758 | transferase activity, transferring hexosyl groups | [M] |
|  | GO:0009312 | oligosaccharide biosynthetic process | [B] |
|  | GO:0009877 | nodulation | [B] |
| - NPR2 |  |  |  |  |
| - NPR3 |  |  |  |  |
| - Nuc-transf |  |  |  |  |
| - Nuc\_deoxyrib\_tr |  |  |  |  |
| - Nucleos\_tra2\_C |  |  |  |  |
| - Nucleos\_tra2\_N | GO:0005415 | nucleoside:sodium symporter activity | [M] |
|  | GO:0006810 | transport | [B] |
|  | GO:0016020 | membrane | [C] |
| - ODC\_AZ | GO:0004857 | enzyme inhibitor activity | [M] |
|  | GO:0008073 | ornithine decarboxylase inhibitor activity | [M] |
| - ODV-E18 | GO:0019031 | viral envelope | [C] |
| - OGFr\_N | GO:0004872 | receptor activity | [M] |
|  | GO:0016020 | membrane | [C] |
| - OLF |  |  |  |  |
| - Opi1 |  |  |  |  |
| - OpuAC | GO:0005215 | transporter activity | [M] |
|  | GO:0005488 | binding | [M] |
|  | GO:0006810 | transport | [B] |
| - ox\_reductase\_C |  |  |  |  |
| - P-mevalo\_kinase | GO:0004631 | phosphomevalonate kinase activity | [M] |
|  | GO:0006695 | cholesterol biosynthetic process | [B] |
|  | GO:0005737 | cytoplasm | [C] |
| - P\_proprotein | GO:0004252 | serine-type endopeptidase activity | [M] |
|  | GO:0006508 | proteolysis | [B] |
| - PA26 | GO:0007050 | cell cycle arrest | [B] |
|  | GO:0005634 | nucleus | [C] |
| - PA28\_beta | GO:0008538 | proteasome activator activity | [M] |
|  | GO:0008537 | proteasome activator complex | [C] |
| - PA\_decarbox | GO:0016831 | carboxy-lyase activity | [M] |
| - Pacifastin\_I | GO:0030414 | peptidase inhibitor activity | [M] |
| - PAP1 |  |  |  |  |
| - PARP\_regulatory | GO:0004483 | mRNA (nucleoside-2'-O-)-methyltransferase activity | [M] |
|  | GO:0006370 | mRNA capping | [B] |
|  | GO:0006397 | mRNA processing | [B] |
| - PduV-EutP |  |  |  |  |
| - Pecanex\_C | GO:0016021 | integral to membrane | [C] |
| - Pectate\_lyase | GO:0030570 | pectate lyase activity | [M] |
|  | GO:0005576 | extracellular region | [C] |
| - Pedibin |  |  |  |  |
| - Pellino |  |  |  |  |
| - Peptidase\_M15 | GO:0008237 | metallopeptidase activity | [M] |
|  | GO:0016805 | dipeptidase activity | [M] |
|  | GO:0006508 | proteolysis | [B] |
|  | GO:0005618 | cell wall | [C] |
| - Peptidase\_M19 | GO:0008235 | metalloexopeptidase activity | [M] |
|  | GO:0008239 | dipeptidyl-peptidase activity | [M] |
|  | GO:0016805 | dipeptidase activity | [M] |
|  | GO:0006508 | proteolysis | [B] |
| - Peptidase\_M36 |  |  |  |  |
| - Peptidase\_M54 | GO:0008237 | metallopeptidase activity | [M] |
|  | GO:0008270 | zinc ion binding | [M] |
| - Peptidase\_M64 |  |  |  |  |
| - Peptidase\_S51 | GO:0008236 | serine-type peptidase activity | [M] |
|  | GO:0006508 | proteolysis | [B] |
| - Peroxidase\_2 | GO:0004601 | peroxidase activity | [M] |
| - PGPGW |  |  |  |  |
| - Phe\_tRNA-synt\_N | GO:0000166 | nucleotide binding | [M] |
|  | GO:0004826 | phenylalanine-tRNA ligase activity | [M] |
|  | GO:0005524 | ATP binding | [M] |
|  | GO:0006412 | translation | [B] |
|  | GO:0006432 | phenylalanyl-tRNA aminoacylation | [B] |
|  | GO:0005737 | cytoplasm | [C] |
| - Phenol\_Hydrox | GO:0006725 | cellular aromatic compound metabolic process | [B] |
|  | GO:0055114 | oxidation reduction | [B] |
| - Phtf-FEM1B\_bdg |  |  |  |  |
| - PI3K\_1B\_p101 |  |  |  |  |
| - PI3K\_rbd | GO:0016303 | 1-phosphatidylinositol-3-kinase activity | [M] |
|  | GO:0005942 | phosphoinositide 3-kinase complex | [C] |
| - PKD |  |  |  |  |
| - Pkr1 |  |  |  |  |
| - Plasmod\_Pvs28 | GO:0009986 | cell surface | [C] |
|  | GO:0016020 | membrane | [C] |
| - Plasmodium\_HRP |  |  |  |  |
| - Pox\_A32 | GO:0005524 | ATP binding | [M] |
| - Propep\_M14 | GO:0004180 | carboxypeptidase activity | [M] |
|  | GO:0006508 | proteolysis | [B] |
| - PSP1 |  |  |  |  |
| - PT |  |  |  |  |
| - PTE | GO:0008270 | zinc ion binding | [M] |
|  | GO:0016788 | hydrolase activity, acting on ester bonds | [M] |
|  | GO:0009056 | catabolic process | [B] |
| - PYNP\_C | GO:0016763 | transferase activity, transferring pentosyl groups | [M] |
|  | GO:0006213 | pyrimidine nucleoside metabolic process | [B] |
| - RA | GO:0007165 | signal transduction | [B] |
| - RAI16-like |  |  |  |  |
| - RAP1 |  |  |  |  |
| - RasGAP | GO:0005096 | GTPase activator activity | [M] |
|  | GO:0051056 | regulation of small GTPase mediated signal transduction | [B] |
|  | GO:0005622 | intracellular | [C] |
| - RasGEF | GO:0005085 | guanyl-nucleotide exchange factor activity | [M] |
|  | GO:0007264 | small GTPase mediated signal transduction | [B] |
|  | GO:0005622 | intracellular | [C] |
| - RasGEF\_N | GO:0005085 | guanyl-nucleotide exchange factor activity | [M] |
|  | GO:0051056 | regulation of small GTPase mediated signal transduction | [B] |
|  | GO:0005622 | intracellular | [C] |
| - Rb\_C |  |  |  |  |
| - RbsD\_FucU | GO:0008643 | carbohydrate transport | [B] |
| - RDD |  |  |  |  |
| - Renin\_r | GO:0004872 | receptor activity | [M] |
|  | GO:0016021 | integral to membrane | [C] |
| - RhgB\_N | GO:0016837 | carbon-oxygen lyase activity, acting on polysaccharides | [M] |
|  | GO:0030246 | carbohydrate binding | [M] |
|  | GO:0005975 | carbohydrate metabolic process | [B] |
| - Ric8 |  |  |  |  |
| - RNA\_bind | GO:0003723 | RNA binding | [M] |
|  | GO:0004535 | poly(A)-specific ribonuclease activity | [M] |
|  | GO:0046872 | metal ion binding | [M] |
|  | GO:0006402 | mRNA catabolic process | [B] |
|  | GO:0005634 | nucleus | [C] |
|  | GO:0005737 | cytoplasm | [C] |
| - RNA\_lig\_T4\_1 |  |  |  |  |
| - RNA\_Me\_trans |  |  |  |  |
| - Rod\_C |  |  |  |  |
| - Rogdi\_lz |  |  |  |  |
| - RPEL |  |  |  |  |
| - Rpp20 |  |  |  |  |
| - RR\_TM4-6 | GO:0005219 | ryanodine-sensitive calcium-release channel activity | [M] |
|  | GO:0006874 | cellular calcium ion homeostasis | [B] |
|  | GO:0016021 | integral to membrane | [C] |
| - RyR |  |  |  |  |
| - S-antigen |  |  |  |  |
| - Scs3p |  |  |  |  |
| - Sds3 |  |  |  |  |
| - Sec66 |  |  |  |  |
| - SH2 | GO:0005515 | protein binding | [M] |
| - Silic\_transp |  |  |  |  |
| - SIN1 |  |  |  |  |
| - Siva | GO:0005175 | CD27 receptor binding | [M] |
|  | GO:0043065 | positive regulation of apoptosis | [B] |
|  | GO:0005737 | cytoplasm | [C] |
| - SK\_channel |  |  |  |  |
| - Somatomedin\_B | GO:0005044 | scavenger receptor activity | [M] |
|  | GO:0030247 | polysaccharide binding | [M] |
|  | GO:0006955 | immune response | [B] |
| - Spectrin |  |  |  |  |
| - Sporozoite\_P67 |  |  |  |  |
| - SR-25 |  |  |  |  |
| - Stanniocalcin | GO:0005179 | hormone activity | [M] |
|  | GO:0005576 | extracellular region | [C] |
| - Strep\_SA\_rep |  |  |  |  |
| - Striatin |  |  |  |  |
| - SusD |  |  |  |  |
| - Sushi |  |  |  |  |
| - Swi5 |  |  |  |  |
| - T4\_deiodinase | GO:0004800 | thyroxine 5'-deiodinase activity | [M] |
|  | GO:0055114 | oxidation reduction | [B] |
| - TEA | GO:0003700 | transcription factor activity | [M] |
|  | GO:0006355 | regulation of transcription, DNA-dependent | [B] |
|  | GO:0005634 | nucleus | [C] |
| - TerD | GO:0006950 | response to stress | [B] |
| - TIL |  |  |  |  |
| - TilS\_C |  |  |  |  |
| - Tmp39 |  |  |  |  |
| - Tom37\_C |  |  |  |  |
| - TrmB |  |  |  |  |
| - TTKRSYEDQ |  |  |  |  |
| - Tup\_N |  |  |  |  |
| - Turandot |  |  |  |  |
| - Tweety |  |  |  |  |
| - UbiD |  |  |  |  |
| - UK |  |  |  |  |
| - UnbV\_ASPIC |  |  |  |  |
| - UPF0157 |  |  |  |  |
| - UPF0227 |  |  |  |  |
| - UPF0240 |  |  |  |  |
| - UT | GO:0015204 | urea transmembrane transporter activity | [M] |
|  | GO:0015840 | urea transport | [B] |
|  | GO:0016021 | integral to membrane | [C] |
| - UxuA | GO:0008927 | mannonate dehydratase activity | [M] |
|  | GO:0006064 | glucuronate catabolic process | [B] |
| - Vault |  |  |  |  |
| - VKG\_Carbox | GO:0008488 | gamma-glutamyl carboxylase activity | [M] |
|  | GO:0017187 | peptidyl-glutamic acid carboxylation | [B] |
| - VSP |  |  |  |  |
| - VWD |  |  |  |  |
| - WBP-1 |  |  |  |  |
| - WH1 |  |  |  |  |
| - WH2 |  |  |  |  |
| - XFP | GO:0016832 | aldehyde-lyase activity | [M] |
|  | GO:0005975 | carbohydrate metabolic process | [B] |
| - XFP\_C | GO:0016832 | aldehyde-lyase activity | [M] |
|  | GO:0005975 | carbohydrate metabolic process | [B] |
| - XFP\_N |  |  |  |  |
| - XLF |  |  |  |  |
| - YdjC |  |  |  |  |
| - zf-C4H2 |  |  |  |  |
| - zf-CCHH |  |  |  |  |
| - zf-CXXC | GO:0003677 | DNA binding | [M] |
|  | GO:0008270 | zinc ion binding | [M] |
| - zf-NPL4 |  |  |  |  |
| - Zn\_clus | GO:0003700 | transcription factor activity | [M] |
|  | GO:0008270 | zinc ion binding | [M] |
|  | GO:0006355 | regulation of transcription, DNA-dependent | [B] |
|  | GO:0005634 | nucleus | [C] |
| - Zot |  |  |  |  |

---

## Arthropoda [eol|tol]

|  |  |  |  |
| --- | --- | --- | --- |
| **Pfam domain(s)** | **GO term acc** | **GO term** | **GO namespace** |
| - 7TM\_GPCR\_Srbc |  |  |  |  |
| - 7TM\_GPCR\_Srsx |  |  |  |  |
| - 7TM\_GPCR\_Srv |  |  |  |  |
| - 7TM\_GPCR\_Srx |  |  |  |  |
| - AAA\_3 | GO:0005524 | ATP binding | [M] |
|  | GO:0016887 | ATPase activity | [M] |
| - Acyl-CoA\_dh\_2 |  |  |  |  |
| - Acyl\_CoA\_thio | GO:0016291 | acyl-CoA thioesterase activity | [M] |
|  | GO:0006637 | acyl-CoA metabolic process | [B] |
| - Amastin |  |  |  |  |
| - Amidohydro\_3 |  |  |  |  |
| - Arylesterase | GO:0004064 | arylesterase activity | [M] |
| - Auxin\_resp | GO:0003677 | DNA binding | [M] |
|  | GO:0009725 | response to hormone stimulus | [B] |
|  | GO:0045449 | regulation of transcription | [B] |
|  | GO:0005634 | nucleus | [C] |
| - BAAT\_C |  |  |  |  |
| - BH4 | GO:0042981 | regulation of apoptosis | [B] |
| - Bile\_Hydr\_Trans | GO:0016290 | palmitoyl-CoA hydrolase activity | [M] |
|  | GO:0006629 | lipid metabolic process | [B] |
| - Cadherin\_pro |  |  |  |  |
| - Calsequestrin | GO:0005509 | calcium ion binding | [M] |
| - CAT | GO:0008811 | chloramphenicol O-acetyltransferase activity | [M] |
| - CBM49 | GO:0030246 | carbohydrate binding | [M] |
|  | GO:0005576 | extracellular region | [C] |
| - CD34\_antigen |  |  |  |  |
| - CMAS | GO:0008825 | cyclopropane-fatty-acyl-phospholipid synthase activity | [M] |
|  | GO:0008610 | lipid biosynthetic process | [B] |
| - Condensation |  |  |  |  |
| - Cyanate\_lyase | GO:0008824 | cyanate hydratase activity | [M] |
|  | GO:0009439 | cyanate metabolic process | [B] |
| - DivIC | GO:0007049 | cell cycle | [B] |
| - DLL\_N |  |  |  |  |
| - DNA\_pol3\_beta | GO:0003677 | DNA binding | [M] |
|  | GO:0003887 | DNA-directed DNA polymerase activity | [M] |
|  | GO:0008408 | 3'-5' exonuclease activity | [M] |
|  | GO:0006260 | DNA replication | [B] |
| - DUF1057 |  |  |  |  |
| - DUF1234 |  |  |  |  |
| - DUF1261 |  |  |  |  |
| - DUF1280 |  |  |  |  |
| - DUF1356 |  |  |  |  |
| - DUF1387 |  |  |  |  |
| - DUF1399 |  |  |  |  |
| - DUF148 |  |  |  |  |
| - DUF1632 |  |  |  |  |
| - DUF1690 |  |  |  |  |
| - DUF1757 |  |  |  |  |
| - DUF1768 |  |  |  |  |
| - DUF19 |  |  |  |  |
| - DUF2424 |  |  |  |  |
| - DUF2650 |  |  |  |  |
| - DUF268 |  |  |  |  |
| - DUF273 |  |  |  |  |
| - DUF288 |  |  |  |  |
| - DUF3050 |  |  |  |  |
| - DUF3106 |  |  |  |  |
| - DUF3133 |  |  |  |  |
| - DUF3250 |  |  |  |  |
| - DUF3474 |  |  |  |  |
| - DUF3715 |  |  |  |  |
| - DUF477 |  |  |  |  |
| - DUF612 |  |  |  |  |
| - DUF724 |  |  |  |  |
| - DUF815 |  |  |  |  |
| - EBP | GO:0047750 | cholestenol delta-isomerase activity | [M] |
|  | GO:0016125 | sterol metabolic process | [B] |
|  | GO:0005783 | endoplasmic reticulum | [C] |
|  | GO:0016021 | integral to membrane | [C] |
| - ERG2\_Sigma1R | GO:0000247 | C-8 sterol isomerase activity | [M] |
|  | GO:0006696 | ergosterol biosynthetic process | [B] |
|  | GO:0005783 | endoplasmic reticulum | [C] |
| - ETX\_MTX2 |  |  |  |  |
| - Exonuc\_VII\_L |  |  |  |  |
| - FerA | GO:0016021 | integral to membrane | [C] |
| - FTH |  |  |  |  |
| - GETHR |  |  |  |  |
| - GFRP | GO:0005515 | protein binding | [M] |
|  | GO:0009890 | negative regulation of biosynthetic process | [B] |
| - GLTT |  |  |  |  |
| - Gly\_acyl\_tr\_C |  |  |  |  |
| - Glyco\_hydro\_25 | GO:0003796 | lysozyme activity | [M] |
|  | GO:0009253 | peptidoglycan catabolic process | [B] |
|  | GO:0016998 | cell wall macromolecule catabolic process | [B] |
| - Glyco\_hydro\_59 | GO:0004336 | galactosylceramidase activity | [M] |
|  | GO:0006683 | galactosylceramide catabolic process | [B] |
| - Glyco\_hydro\_88 |  |  |  |  |
| - Hepcidin | GO:0006879 | cellular iron ion homeostasis | [B] |
|  | GO:0005576 | extracellular region | [C] |
| - HtrL\_YibB |  |  |  |  |
| - ICAP-1\_inte\_bdg |  |  |  |  |
| - ICL | GO:0003824 | catalytic activity | [M] |
|  | GO:0008152 | metabolic process | [B] |
| - IDO | GO:0020037 | heme binding | [M] |
| - IIGP | GO:0005525 | GTP binding | [M] |
|  | GO:0016817 | hydrolase activity, acting on acid anhydrides | [M] |
|  | GO:0016020 | membrane | [C] |
| - Inos-1-P\_synth |  |  |  |  |
| - Integrase | GO:0003676 | nucleic acid binding | [M] |
|  | GO:0008907 | integrase activity | [M] |
| - Integrase\_Zn | GO:0003677 | DNA binding | [M] |
|  | GO:0008270 | zinc ion binding | [M] |
|  | GO:0008907 | integrase activity | [M] |
|  | GO:0015074 | DNA integration | [B] |
| - iPGM\_N | GO:0004619 | phosphoglycerate mutase activity | [M] |
|  | GO:0030145 | manganese ion binding | [M] |
|  | GO:0006007 | glucose catabolic process | [B] |
|  | GO:0005737 | cytoplasm | [C] |
| - K\_trans | GO:0015079 | potassium ion transmembrane transporter activity | [M] |
|  | GO:0006813 | potassium ion transport | [B] |
|  | GO:0016020 | membrane | [C] |
| - LBP\_BPI\_CETP | GO:0008289 | lipid binding | [M] |
| - LBP\_BPI\_CETP\_C | GO:0008289 | lipid binding | [M] |
| - Lipase\_2 |  |  |  |  |
| - MAP2\_projctn |  |  |  |  |
| - MbeD\_MobD |  |  |  |  |
| - Metalloenzyme | GO:0003824 | catalytic activity | [M] |
|  | GO:0046872 | metal ion binding | [M] |
| - Methyltransf\_3 | GO:0008171 | O-methyltransferase activity | [M] |
| - MgtE\_N |  |  |  |  |
| - MreB\_Mbl | GO:0000902 | cell morphogenesis | [B] |
| - Mur\_ligase\_C | GO:0005524 | ATP binding | [M] |
|  | GO:0016874 | ligase activity | [M] |
|  | GO:0009058 | biosynthetic process | [B] |
| - Nha1\_C | GO:0015385 | sodium:hydrogen antiporter activity | [M] |
|  | GO:0006814 | sodium ion transport | [B] |
|  | GO:0016020 | membrane | [C] |
| - NodS | GO:0008757 | S-adenosylmethionine-dependent methyltransferase activity | [M] |
|  | GO:0009312 | oligosaccharide biosynthetic process | [B] |
|  | GO:0009877 | nodulation | [B] |
| - Nucleotid\_trans |  |  |  |  |
| - OAD\_gamma | GO:0008948 | oxaloacetate decarboxylase activity | [M] |
|  | GO:0015081 | sodium ion transmembrane transporter activity | [M] |
|  | GO:0006814 | sodium ion transport | [B] |
|  | GO:0016020 | membrane | [C] |
| - Oxidored\_FMN | GO:0010181 | FMN binding | [M] |
|  | GO:0016491 | oxidoreductase activity | [M] |
| - PAN\_2 |  |  |  |  |
| - PAN\_3 |  |  |  |  |
| - Peptidase\_C39 | GO:0005524 | ATP binding | [M] |
|  | GO:0008233 | peptidase activity | [M] |
|  | GO:0006508 | proteolysis | [B] |
|  | GO:0016021 | integral to membrane | [C] |
| - Peptidase\_M18 | GO:0004177 | aminopeptidase activity | [M] |
|  | GO:0008270 | zinc ion binding | [M] |
|  | GO:0006508 | proteolysis | [B] |
|  | GO:0005773 | vacuole | [C] |
| - PGPGW |  |  |  |  |
| - Phytochelatin | GO:0016756 | glutathione gamma-glutamylcysteinyltransferase activity | [M] |
|  | GO:0046872 | metal ion binding | [M] |
|  | GO:0010038 | response to metal ion | [B] |
|  | GO:0046938 | phytochelatin biosynthetic process | [B] |
| - Protocadherin |  |  |  |  |
| - PTS\_EIIB |  |  |  |  |
| - RCSD |  |  |  |  |
| - RdRP |  |  |  |  |
| - RIG-I\_C-RD |  |  |  |  |
| - RimK |  |  |  |  |
| - S-antigen |  |  |  |  |
| - SANTA |  |  |  |  |
| - Self-incomp\_S1 |  |  |  |  |
| - Serpentine\_r\_xa |  |  |  |  |
| - SKI | GO:0004765 | shikimate kinase activity | [M] |
|  | GO:0005524 | ATP binding | [M] |
| - SLR1-BP |  |  |  |  |
| - Slx4 |  |  |  |  |
| - SnoaL |  |  |  |  |
| - Spo12 |  |  |  |  |
| - Stanniocalcin | GO:0005179 | hormone activity | [M] |
|  | GO:0005576 | extracellular region | [C] |
| - Ste50p-SAM |  |  |  |  |
| - SURF2 |  |  |  |  |
| - TauE |  |  |  |  |
| - TLV\_coat |  |  |  |  |
| - Tme5\_EGF\_like |  |  |  |  |
| - Toxin\_1 | GO:0005576 | extracellular region | [C] |
| - Transp\_Tc5\_C |  |  |  |  |
| - TrmB |  |  |  |  |
| - Tyrosinase | GO:0016491 | oxidoreductase activity | [M] |
|  | GO:0008152 | metabolic process | [B] |
| - UPF0057 | GO:0016021 | integral to membrane | [C] |
| - UPF0079 |  |  |  |  |
| - UPF0560 |  |  |  |  |
| - UPF0561 |  |  |  |  |
| - V-set\_CD47 |  |  |  |  |

---

## Ascidiacea [eol|tol]

|  |  |  |  |
| --- | --- | --- | --- |
| **Pfam domain(s)** | **GO term acc** | **GO term** | **GO namespace** |
| - 4F5 |  |  |  |  |
| - A4\_EXTRA | GO:0005488 | binding | [M] |
|  | GO:0016021 | integral to membrane | [C] |
| - AATase | GO:0004026 | alcohol O-acetyltransferase activity | [M] |
|  | GO:0006066 | alcohol metabolic process | [B] |
| - AD |  |  |  |  |
| - Adeno\_IVa2 | GO:0019083 | viral transcription | [B] |
| - AGTRAP |  |  |  |  |
| - Aha1\_N | GO:0001671 | ATPase activator activity | [M] |
|  | GO:0051087 | chaperone binding | [M] |
|  | GO:0005737 | cytoplasm | [C] |
| - AHSA1 | GO:0006950 | response to stress | [B] |
| - Amidase\_2 | GO:0008745 | N-acetylmuramoyl-L-alanine amidase activity | [M] |
|  | GO:0009253 | peptidoglycan catabolic process | [B] |
| - ArgK |  |  |  |  |
| - Asparaginase | GO:0006520 | cellular amino acid metabolic process | [B] |
| - ATP-synt\_E | GO:0015078 | hydrogen ion transmembrane transporter activity | [M] |
|  | GO:0015986 | ATP synthesis coupled proton transport | [B] |
|  | GO:0000276 | mitochondrial proton-transporting ATP synthase complex, coupling factor F(o) | [C] |
| - ATP-synt\_G | GO:0015078 | hydrogen ion transmembrane transporter activity | [M] |
|  | GO:0015986 | ATP synthesis coupled proton transport | [B] |
|  | GO:0000276 | mitochondrial proton-transporting ATP synthase complex, coupling factor F(o) | [C] |
| - ATP\_synt\_H | GO:0015078 | hydrogen ion transmembrane transporter activity | [M] |
|  | GO:0015991 | ATP hydrolysis coupled proton transport | [B] |
|  | GO:0033179 | proton-transporting V-type ATPase, V0 domain | [C] |
| - ATS3 |  |  |  |  |
| - Axin\_b-cat\_bind |  |  |  |  |
| - BAF | GO:0003677 | DNA binding | [M] |
| - BolA |  |  |  |  |
| - Borrelia\_P83 |  |  |  |  |
| - BPD\_transp\_2 | GO:0005215 | transporter activity | [M] |
|  | GO:0006810 | transport | [B] |
|  | GO:0016020 | membrane | [C] |
| - BRCA-2\_helical | GO:0003697 | single-stranded DNA binding | [M] |
|  | GO:0005515 | protein binding | [M] |
|  | GO:0000724 | double-strand break repair via homologous recombination | [B] |
|  | GO:0006310 | DNA recombination | [B] |
|  | GO:0007090 | regulation of S phase of mitotic cell cycle | [B] |
|  | GO:0005634 | nucleus | [C] |
| - BRCA-2\_OB1 |  |  |  |  |
| - C1-set |  |  |  |  |
| - Calsarcin |  |  |  |  |
| - CbiA | GO:0042242 | cobyrinic acid a,c-diamide synthase activity | [M] |
|  | GO:0009236 | cobalamin biosynthetic process | [B] |
| - CBM\_4\_9 |  |  |  |  |
| - CDK2AP |  |  |  |  |
| - CENP-B\_N | GO:0003677 | DNA binding | [M] |
|  | GO:0000775 | chromosome, centromeric region | [C] |
| - CHD5 |  |  |  |  |
| - ClpS | GO:0030163 | protein catabolic process | [B] |
| - CMAS | GO:0008825 | cyclopropane-fatty-acyl-phospholipid synthase activity | [M] |
|  | GO:0008610 | lipid biosynthetic process | [B] |
| - Cmyb\_C |  |  |  |  |
| - COG4 |  |  |  |  |
| - Cohesin\_load |  |  |  |  |
| - Condensation |  |  |  |  |
| - CotH |  |  |  |  |
| - COX17 | GO:0005507 | copper ion binding | [M] |
|  | GO:0016531 | copper chaperone activity | [M] |
|  | GO:0006825 | copper ion transport | [B] |
|  | GO:0005758 | mitochondrial intermembrane space | [C] |
| - COX4 | GO:0004129 | cytochrome-c oxidase activity | [M] |
| - COX6B | GO:0004129 | cytochrome-c oxidase activity | [M] |
|  | GO:0005739 | mitochondrion | [C] |
| - Cpn10 | GO:0006457 | protein folding | [B] |
|  | GO:0005737 | cytoplasm | [C] |
| - CutA1 | GO:0010038 | response to metal ion | [B] |
| - CutC | GO:0005507 | copper ion binding | [M] |
|  | GO:0055070 | copper ion homeostasis | [B] |
| - Cystatin | GO:0004869 | cysteine-type endopeptidase inhibitor activity | [M] |
| - Dak1 | GO:0004371 | glycerone kinase activity | [M] |
|  | GO:0006071 | glycerol metabolic process | [B] |
| - Dak2 | GO:0004371 | glycerone kinase activity | [M] |
|  | GO:0006071 | glycerol metabolic process | [B] |
| - Dam | GO:0003677 | DNA binding | [M] |
|  | GO:0009007 | site-specific DNA-methyltransferase (adenine-specific) activity | [M] |
|  | GO:0006306 | DNA methylation | [B] |
| - DAP\_epimerase | GO:0008837 | diaminopimelate epimerase activity | [M] |
[truncated: 1,870,174 more chars]
