# Supplementary material for: Strong functional patterns in the evolution of eukaryotic genomes revealed by the reconstruction of ancestral protein domain repertoires
Source: Genome Biol. 2011 Jan 17;12(1):R4. doi: 10.1186/gb-2011-12-1-r4 (PMC3091302; doi:10.1186/gb-2011-12-1-r4)

Numbers of distinct domains per genome in extant species (for groups of species represented as triangles, these numbers are averages; species, or groups of species, which are mostly parasitic are shown in grey):

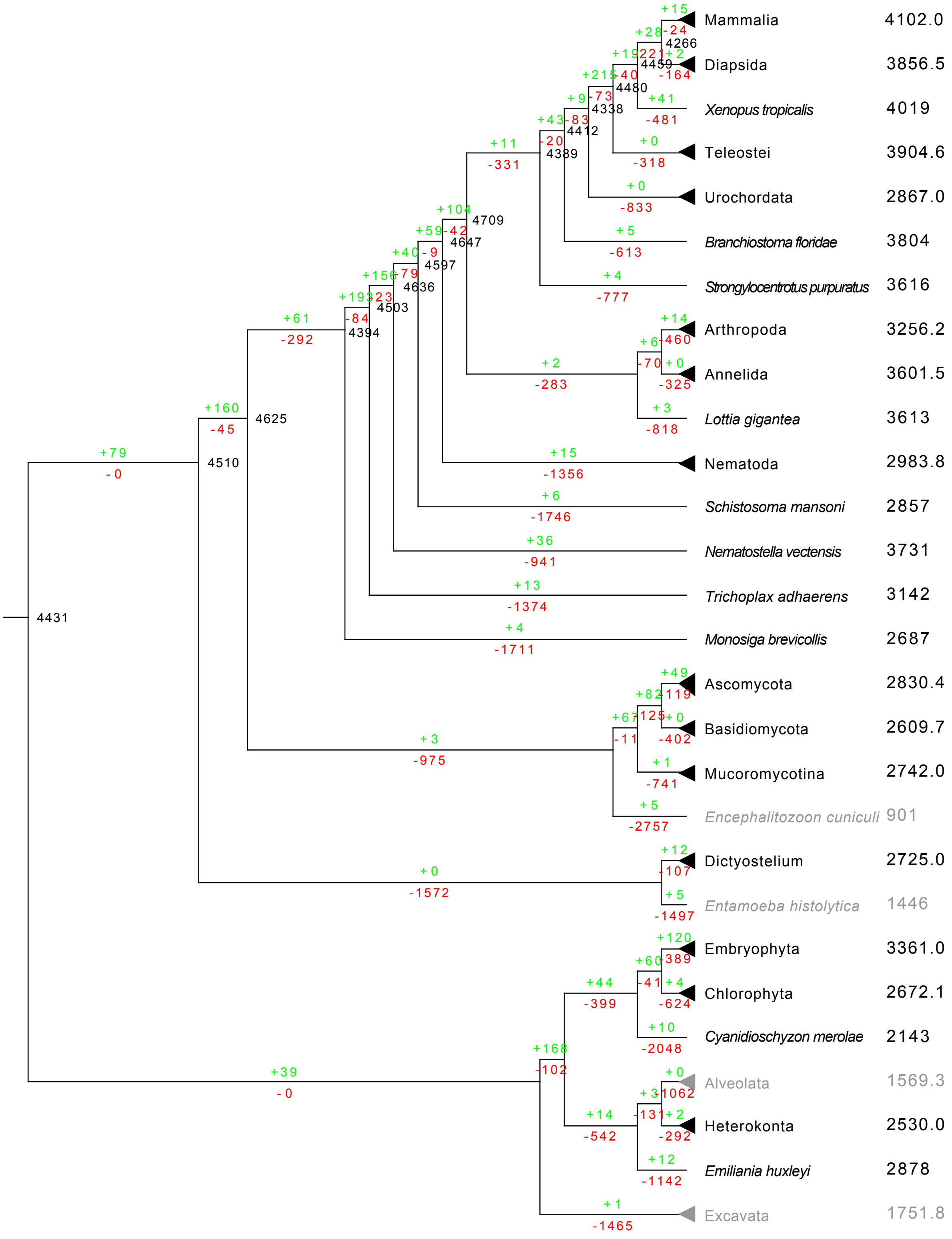

Supplement: Additional file 7 — Domain gain and loss counts during eukaryote evolution under a coelomata model. Summary of conditions used: protein predictions as listed in Additional file 1, domain models from Pfam 24.0, analyzed with HMMER 3.0b2, Pfam 'gathering' cutoffs. [file gb-2011-12-1-r4-S7.pdf]
