# Supplementary material for: Strong functional patterns in the evolution of eukaryotic genomes revealed by the reconstruction of ancestral protein domain repertoires
Source: Genome Biol. 2011 Jan 17;12(1):R4. doi: 10.1186/gb-2011-12-1-r4 (PMC3091302; doi:10.1186/gb-2011-12-1-r4)
Supplement: Additional file 8 — Domain gains and loss counts during eukaryote evolution under a 'crown group' model. Summary of conditions used: protein predictions as listed in Additional file 1, domain models from Pfam 24.0, analyzed with HMMER 3.0b2, Pfam 'gathering' cutoffs. [file gb-2011-12-1-r4-S8.pdf]

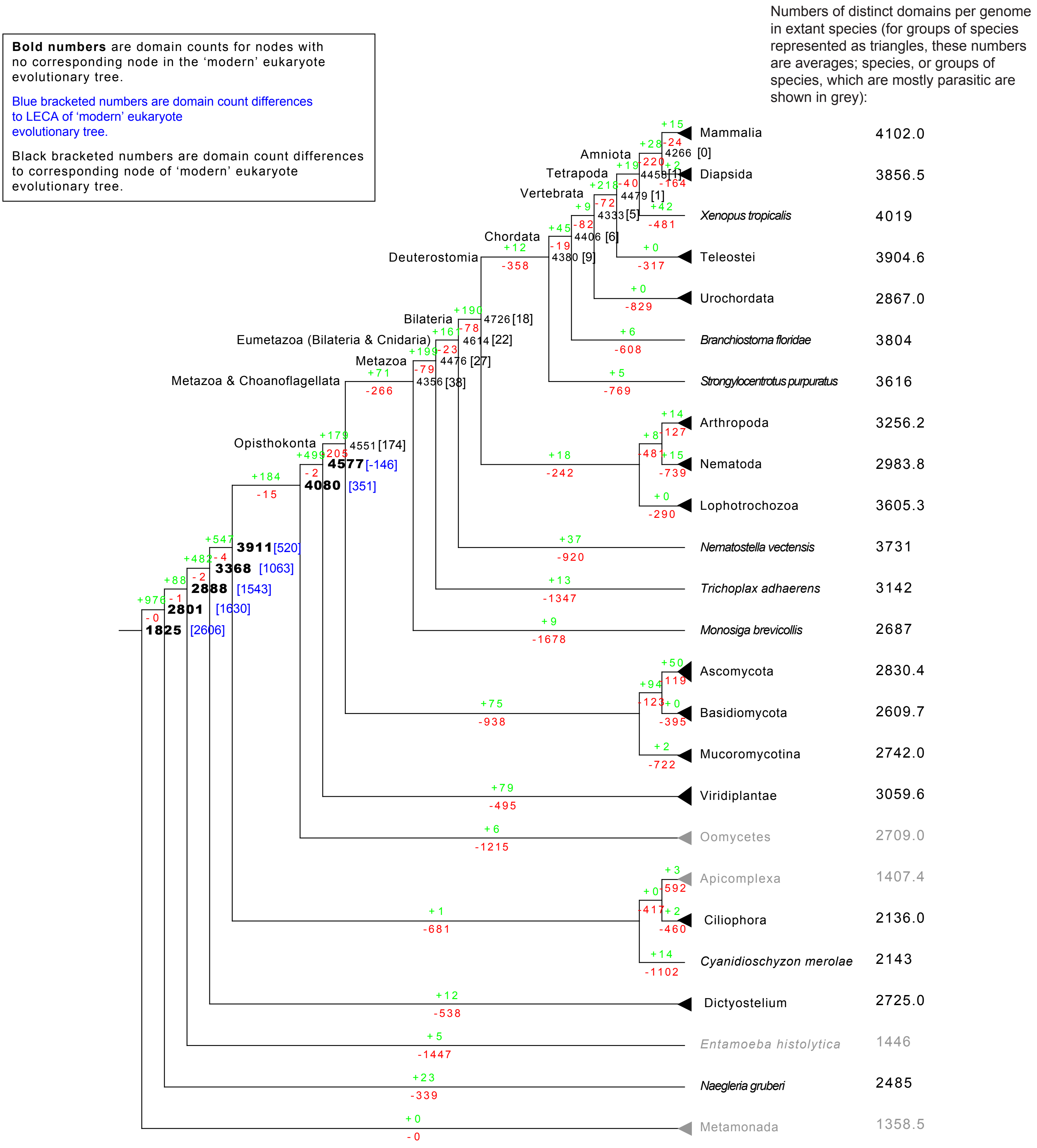

Bold numbers are domain counts for nodes with no corresponding node in the 'modern' eukaryote evolutionary tree.
Blue bracketed numbers are domain count differences to LECA of 'modern' eukaryote evolutionary tree.
Black bracketed numbers are domain count differences to corresponding node of 'modern' eukaryote evolutionary tree.
Numbers of distinct domains per genome in extant species (for groups of species represented as triangles, these numbers are averages; species, or groups of species, which are mostly parasitic are shown in grey):
Mammalia 4102.0
Diapsida 3856.5
Xenopus tropicalis 4019
Teleostei 3904.6
Urochordata 2867.0
Branchiostoma floridae 3804
Strongylocentrotus purpuratus 3616
Arthropoda 3256.2
Nematoda 2983.8
Lophotrochozoa 3605.3
Nematostella vectensis 3731
Trichoplax adhaerens 3142
Monosiga brevicollis 2687
Ascomycota 2830.4
Basidiomycota 2609.7
Mucoromycotina 2742.0
Viridiplantae 3059.6
Oomycetes 2709.0
Apicomplexa 1407.4
Ciliophora 2136.0
Cyanidioschyzon merolae 2143
Dictyostelium 2725.0
Entamoeba histolytica 1446
Naegleria gruberi 2485
Metamonada 1358.5
Amniota
Tetrapoda
Vertebrata
Chordata
Deuterostomia
Bilateria
Eumetazoa (Bilateria & Cnidaria)
Metazoa
Metazoa & Choanoflagellata
Opisthokonta
4266 [0]
4458 [1]
4479 [1]
4333 [5]
4406 [6]
4380 [9]
4726 [18]
4614 [22]
4476 [27]
4356 [38]
4551 [174]
4577 [-146]
4080 [351]
3911 [520]
3368 [1063]
2888 [1543]
2801 [1630]
1825 [2606]
