# Supplementary material for: Strong functional patterns in the evolution of eukaryotic genomes revealed by the reconstruction of ancestral protein domain repertoires
Source: Genome Biol. 2011 Jan 17;12(1):R4. doi: 10.1186/gb-2011-12-1-r4 (PMC3091302; doi:10.1186/gb-2011-12-1-r4)
Supplement: Additional file 9 — Table of enriched gained and lost GO terms evolution under a coelomata model. The two terms with the lowest P-values are shown. Summary of conditions used: protein predictions as listed in Additional file 1, domain models from Pfam 24.0, analyzed with HMMER 3.0b2, Pfam 'gathering' cutoffs, model of eukaryote evolution as shown in Additional file 7, 'pfam2go' mappings dated 2009/10/01, Ontologizer 2.0 with Topology-Elim algorithm. [file gb-2011-12-1-r4-S9.pdf]

	Enriched gained GO terms	P-value	Enriched lost GO terms	P-value
**Unikonta**	• protein import into peroxisome matrix, docking<br>• cAMP catabolic process	9.5E-3<br>1.9E-2		
**Opisthokonta**	• regulation of primary metabolic process	1.3E-2	• protein-heme linkage<br>• asparagine biosynthetic process	5.2E-3<br>1.0E-2
**Metazoa & Choanoflagellata**	• cell-cell signaling<br>• cell surface receptor linked signal transduction	2.2E-3<br>9.2E-3	• xylan catabolic process<br>• carbohydrate metabolic process	1.6E-5<br>3.1E-4
**Metazoa**	• regulation of transcription, DNA-dependent<br>• cell-matrix adhesion	1.2E-7<br>4.0E-4	• aromatic amino acid family biosynthetic process, prephenate pathway<br>• histidine biosynthetic process	1.1E-4<br>2.3E-3
**Bilaterian & Cnidaria**	• apoptosis<br>• peptide cross-linking	3.1E-4<br>4.7E-4	• protein folding<br>• transcription initiation	1.7E-3<br>3.8E-3
**Bilateria**	• multicellular organismal development<br>• cell surface receptor linked signal transduction	1.2E-2<br>1.7E-2	• branched chain family amino acid biosynthetic process<br>• histidine biosynthetic process	3.3E-4<br>2.3E-3
**Deuterostomia & Protostomia & Aschelminthes**	• mitochondrial ATP synthesis coupled electron transport<br>• developmental process	8.8E-4<br>5.3E-3	• DNA replication	1.3E-3
**Deuterostomia & Protostomia**	• mitochondrial electron transport, NADH to ubiquinone<br>• Wnt receptor signaling pathway	4.8E-4<br>3.2E-3	• DNA integration<br>• metal ion transport	9.2E-3<br>1.2E-2
**Deuterostomia**	• protein transport	8.2E-2	• cellular amino acid biosynthetic process<br>• phosphoenolpyruvate-dependent sugar phosphotransferase system	4.7E-4<br>2.7E-3
**Chordata**	• lipid catabolic process<br>• activation of MAPKK activity	3.2E-3<br>6.7E-3	• proteolysis	2.1E-2
**Urochordata & Vertebrata**	• antigen processing and presentation<br>• protein amino acid phosphorylation	5.5E-3<br>1.8E-2	• folic acid and derivative metabolic process<br>• oligosaccharide biosynthetic process	2.3E-3<br>3.0E-3
**Vertebrata**	• immune response<br>• G-protein coupled receptor protein signaling pathway	4.4E-11<br>1.6E-5	• DNA topological change<br>• carbohydrate metabolic process	2.0E-3<br>3.1E-3
**Tetrapoda**	• regulation of growth<br>• synaptic transmission	1.3E-2<br>2.0E-2	• valyl-tRNA aminoacylation<br>• response to water	4.3E-3<br>8.6E-3
**Amniota**	• immune response<br>• defense response	1.8E-3<br>2.0E-3	• regulation of transcription, DNA-dependent<br>• riboflavin biosynthetic process	9.2E-8<br>1.0E-3
**Mammalia**	• hemopoiesis<br>• reciprocal meiotic recombination	2.8E-3<br>8.3E-3	• aromatic amino acid family biosynthetic process	1.1E-2
