# Supplementary material for: Strong functional patterns in the evolution of eukaryotic genomes revealed by the reconstruction of ancestral protein domain repertoires
Source: Genome Biol. 2011 Jan 17;12(1):R4. doi: 10.1186/gb-2011-12-1-r4 (PMC3091302; doi:10.1186/gb-2011-12-1-r4)
Supplement: Additional file 10 — Table of enriched gained and lost GO terms under a 'crown group' model. The two terms with the lowest P-values are shown. Summary of conditions used: protein predictions as listed in Additional file 1, domain models from Pfam 24.0, analyzed with HMMER 3.0b2, Pfam 'gathering' cutoffs, model of eukaryote evolution as shown in Additional file 8, 'pfam2go' mappings dated 2009/10/01, Ontologizer 2.0 with Topology-Elim algorithm. [file gb-2011-12-1-r4-S10.pdf]

	Enriched gained GO terms	P-value	Enriched lost GO terms	P-value
all eukaryotes except Metamonada	• oxidation reduction<br>• folic acid and derivative biosynthetic process	2.0E-6<br>3.4E-4		
all eukaryotes except Metamonada, Naegleria	• sulfate assimilation<br>• GPI anchor biosynthetic process	1.6E-4<br>2.9E-4		
all eukaryotes except Metamonada, Naegleria, Entamoeba	• ATP synthesis coupled electron transport<br>• Mo-molybdopterin cofactor biosynthetic process	1.3E-5<br>9.5E-5	• polysaccharide biosynthetic process	8.0E-3
all eukaryotes except Metamonada, Naegleria, Entamoeba, Dictyostelium	• photosynthesis<br>• histidine biosynthetic process	3.9E-20<br>2.8E-8		
**Opisthokonta & Viridplantae & Oomyces**	• lipopolysaccharide biosynthetic process<br>• carbohydrate metabolic process	9.3E-4<br>2.9E-3	• asparagine biosynthetic process<br>• pathogenesis	2.5E-3<br>2.4E-2
**Opisthokonta & Viridplantae**	• protein secretion<br>• ciliary or flagellar motility	2.7E-6<br>3.5E-3		
**Opisthokonta**	• regulation of primary metabolic process	3.1E-2	• photosynthesis<br>• glycolipid biosynthetic process	2.0E-28<br>8.0E-3
**Metazoa & Choanoflagellata**	• signal transduction<br>• cell-cell signaling	2.9E-4<br>2.9E-3	• protein-chromophore linkage<br>• glucan biosynthetic process	5.4E-4<br>5.2E-3
**Metazoa**	• regulation of transcription, DNA-dependent<br>• cell-matrix adhesion	2.3E-7<br>4.6E-4	• histidine biosynthetic process<br>• tyrosine biosynthetic process	1.8E-3<br>9.5E-3
**Bilaterian & Cnidaria**	• apoptosis<br>• peptide cross-linking	3.1E-4<br>4.7E-4	• protein folding<br>• transcription initiation	1.8E-3<br>3.8E-3
**Bilateria**	• mitochondrial electron transport, NADH to ubiquinone<br>• Wnt receptor signaling pathway	8.3E-6<br>2.8E-4	• branched chain family amino acid biosynthetic process<br>• histidine biosynthetic process	3.36E-4<br>2.3E-3
**Deuterostomia**			• cellular amino acid biosynthetic process<br>• phosphoenolpyruvate-dependent sugar phosphotransferase system	6.7E-4<br>3.1E-3
**Chordata**	• lipid catabolic process<br>• activation of MAPKK activity	3.3E-3<br>6.8E-3	• proteolysis	1.6E-2
**Urochordata & Vertebrata**	• antigen processing and presentation<br>• response to stimulus	5.5E-3<br>8.6E-3	• folic acid and derivative metabolic process<br>• cellular amide metabolic process	2.2E-3<br>6.57E-3
**Vertebrata**	• immune response<br>• G-protein coupled receptor protein signaling pathway	5.14E-11<br>1.8E-5	• DNA topological change<br>• carbohydrate metabolic process	2.0E-3<br>3.0E-3
**Tetrapoda**	• regulation of growth<br>• synaptic transmission	1.4E-2<br>2.0E-2	• valyl-tRNA aminoacylation<br>• response to water	4.3E-3<br>8.6E-3
**Amniota**	• defense response<br>• immune response	1.7E-3<br>1.8E-3	• regulation of transcription, DNA-dependent<br>• riboflavin biosynthetic process	8.6E-8<br>1.0E-3
**Mammalia**	• hemopoiesis<br>• reciprocal meiotic recombination	2.8E-3<br>8.3E-3	• aromatic amino acid family biosynthetic process	1.1E-2
