# Supplementary material for: Strong functional patterns in the evolution of eukaryotic genomes revealed by the reconstruction of ancestral protein domain repertoires
Source: Genome Biol. 2011 Jan 17;12(1):R4. doi: 10.1186/gb-2011-12-1-r4 (PMC3091302; doi:10.1186/gb-2011-12-1-r4)

	Pfam 22.0 (9318 families) HMMER 2.3.2	Pfam 24.0 (11912 families) HMMER 3.0b3									
	Gathering cutoff, Pfam 22.0	Noise cutoff	Gathering cutoff	Gathering cutoff, without parasites	Trusted cutoff	1E-4 E-value cutoff	1E-6 E-value cutoff	1E-8 E-value cutoff	1E-10 E-value cutoff	1E-12 E-value cutoff	1E-18 E-value cutoff
LECA	3392	4520	4431	4344	4387	4995	4437	4210	4048	3904	3547
Chromalveolata	3056	4053	4008	3917	3964	4359	3950	3754	3612	3465	3043
Viridplantae (green plants)	3326	4249	4200	4182	4172	4569	4190	4026	3891	3779	3424
Dikarya (fungi)	2873	3759	3709	3658	3631	4071	3673	3525	3424	3325	3031
Metazoa (animals)	3472	4588	4503	4467	4462	5139	4555	4325	4168	4058	3695
Prostomia	3511	4571	4508	4487	4473	4960	4523	4338	4227	4122	3791
Deuterostomia	3443	4437	4389	4378	4355	4883	4478	4298	4184	4089	3774
Lophotrochozoa	3325	4247	4215	4209	4187	4552	4229	4083	3989	3865	3496
Ecdysozoa	3149	4079	4032	4014	4003	4352	4030	3883	3781	3697	3417
Human	3471	4244	4239	4239	4231	4331	4246	4182	4126	4075	3857
Deuterostome domain gains	11	11	11	13	9	19	15	16	21	16	17
Deuterostome domain losses	238	398	366	347	359	528	355	308	301	283	253
Ecdysozoa domain gains	4	7	8	7	8	12	10	9	7	7	10
Ecdysozoa domain losses	366	499	484	480	478	620	503	464	453	432	383

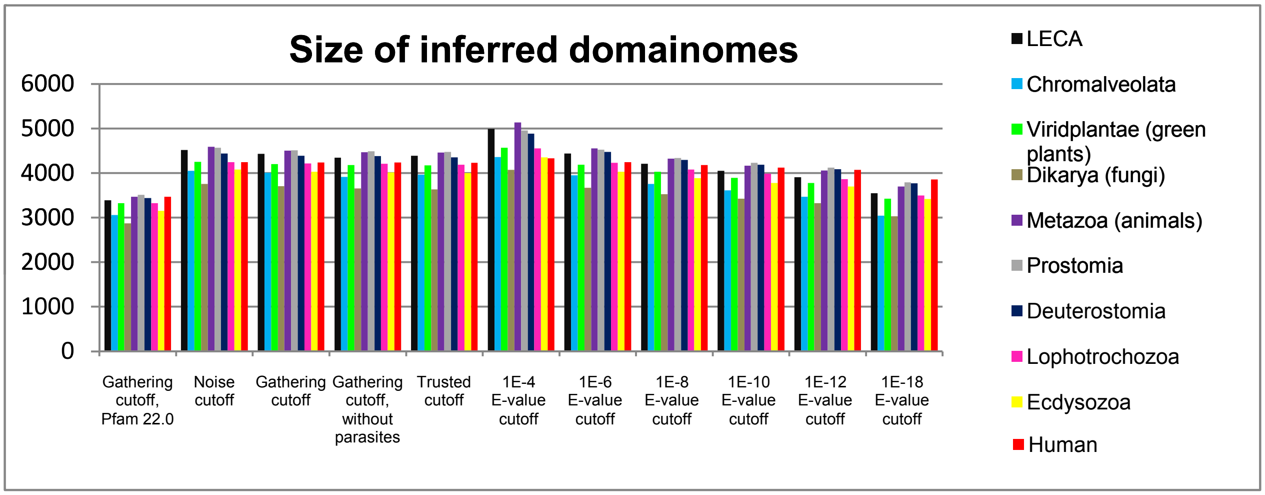

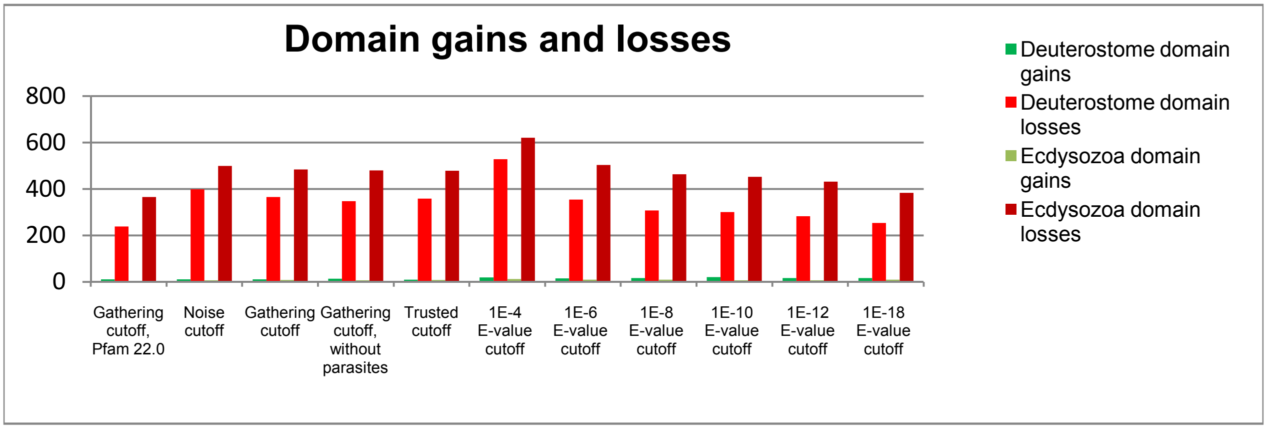

Supplement: Additional file 12 — Domain counts for a variety of cutoff values. [file gb-2011-12-1-r4-S12.pdf]
