# Supplementary material for: Strong functional patterns in the evolution of eukaryotic genomes revealed by the reconstruction of ancestral protein domain repertoires
Source: Genome Biol. 2011 Jan 17;12(1):R4. doi: 10.1186/gb-2011-12-1-r4 (PMC3091302; doi:10.1186/gb-2011-12-1-r4)
Supplement: Additional file 13 — Domain gains and losses during eukaryote evolution for a E-value cutoff of 10-8. Summary of conditions used: protein predictions as listed in Additional file 1, domain models from Pfam 24.0, analyzed with HMMER 3.0b2. [file gb-2011-12-1-r4-S13.pdf]

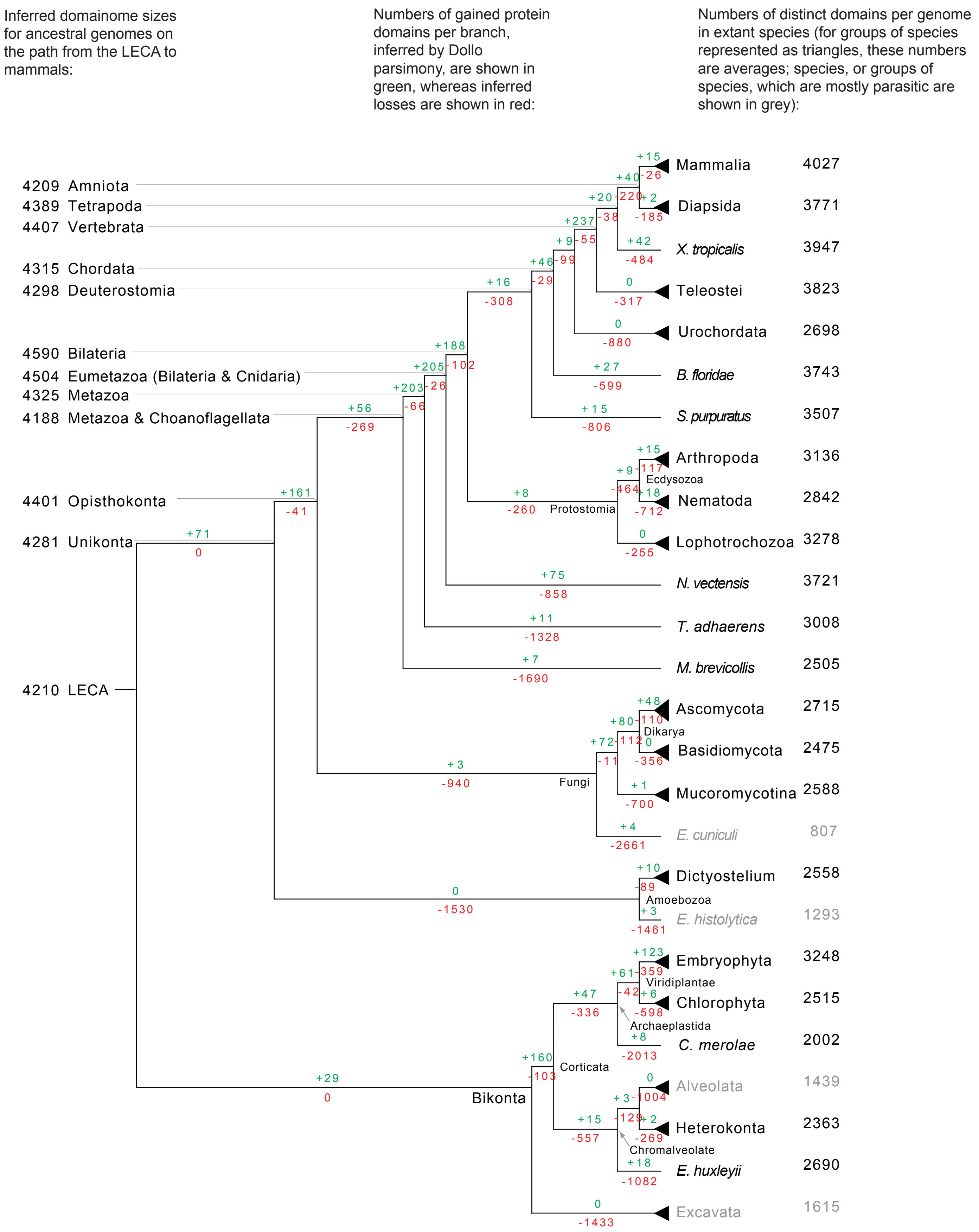
Inferred domainome sizes for ancestral genomes on the path from the LECA to mammals:
Numbers of gained protein domains per branch, inferred by Dollo parsimony, are shown in green, whereas inferred losses are shown in red:
Numbers of distinct domains per genome in extant species (for groups of species represented as triangles, these numbers are averages; species, or groups of species, which are mostly parasitic are shown in grey):
4209 Amniota
4389 Tetrapoda
4407 Vertebrata
4315 Chordata
4298 Deuterostomia
4590 Bilateria
4504 Eumetazoa (Bilateria & Cnidaria)
4325 Metazoa
4188 Metazoa & Choanoflagellata
4401 Opisthokonta
4281 Unikonta
4210 LECA
Mammalia 4027
Diapsida 3771
X. tropicalis 3947
Teleostei 3823
Urochordata 2698
B. floridae 3743
S. purpuratus 3507
Arthropoda 3136
Ecdysozoa
Nematoda 2842
Protostomia
Lophotrochozoa 3278
N. vectensis 3721
T. adhaerens 3008
M. brevicollis 2505
Ascomycota 2715
Dikarya
Basidiomycota 2475
Fungi
Mucoromycotina 2588
E. cuniculi 807
Dictyostelium 2558
Amoebozoa
E. histolytica 1293
Embryophyta 3248
Viridiplantae
Chlorophyta 2515
Archaeplastida
C. merolae 2002
Corticata
Alveolata 1439
Heterokonta 2363
Chromalveolate
E. huxleyii 2690
Bikonta
Excavata 1615
+15
+40 -26
+20 -220 +2
+237 -38 -185
+9 -55
+42 -484
+46 -99
+16 -29
-308
0 -317
0 -880
+188
+205 -102
+27 -599
+203 -26
+56 -66
-269
+15 -806
+15
+9 -117
-464 +18
+161
+8 -260
-712
-41
0 -255
+71
0
+75 -858
+11 -1328
+7 -1690
+48
+80 -110
+72 -112 0
+3 -11 -356
-940
+1 -700
+4 -2661
+10
0 -89
-1530 +3
-1461
+123
+61 -359
+47 -42 +6
-336 -598
+8
-2013
+160
+29 -103
0
0
+3 -1004
+15 -129 +2
-557 -269
+18
-1082
0
-1433
