# Supplementary material for: Strong functional patterns in the evolution of eukaryotic genomes revealed by the reconstruction of ancestral protein domain repertoires
Source: Genome Biol. 2011 Jan 17;12(1):R4. doi: 10.1186/gb-2011-12-1-r4 (PMC3091302; doi:10.1186/gb-2011-12-1-r4)
Supplement: Additional file 14 — Comparison of enriched gained and lost GO terms along path from Unikonta to Mammalia using different calculation methods and different approaches for multiple testing correction. The two terms with the lowest P-values are shown (calculated by the Ontologizer 2.0 software [63]), with the exception of terms marked by an asterisk, due to the relevance of these terms for this work. Prototypical regulatory terms are in red, prototypical metabolic terms are in blue. [file gb-2011-12-1-r4-S14.pdf]

1. Calculation method: Topology-Weighted

	Enriched gained GO terms	P-value	Enriched lost GO terms	P-value
**Unikonta**	• protein import into peroxisome matrix, docking<br>• cAMP catabolic process	9.5E-3<br>1.9E-2		
**Opisthokonta**	• regulation of transcription<br>• regulation of nucleobase, nucleoside, nucleotide and nucleic acid metabolic process	2.3E-2<br>2.8E-2	• protein-heme linkage<br>• asparagine biosynthetic process	5.2E-3<br>1.0E-2
**Metazoa & Choanoflagellata**	• cell communication<br>• galactosylceramide catabolic process	3.6E-6<br>9.2E-3	• xylan catabolic process<br>• hemicellulose metabolic process<br>• polysaccharide catabolic process *	1.6E-5<br>1.5E-4<br>3.2E-4
**Metazoa**	• regulation of transcription, DNA-dependent<br>• transcription, DNA-dependent	1.2E-7<br>1.8E-6	• aromatic amino acid family biosynthetic process, prephenate pathway<br>• histidine biosynthetic process	1.1E-4<br>2.3E-3
**Bilaterian & Cnidaria**	• apoptosis<br>• peptide cross-linking	3.1E-4<br>4.7E-4	• protein folding<br>• transcription initiation	1.7E-3<br>3.8E-3
**Bilateria**	• mitochondrial electron transport, NADH to ubiquinone<br>• mitochondrial ATP synthesis coupled electron transport	8.3E-6<br>1.1E-5	• branched chain family amino acid biosynthetic process<br>• cellular amino acid biosynthetic process<br>• water-soluble vitamin biosynthetic process *	3.3E-4<br>6.3E-4<br>5.0E-3
**Deuterostomia**	• protein transport<br>• protein localization	8.1E-2<br>8.6E-2	• dicarboxylic acid metabolic process<br>• phosphoenolpyruvate-dependent sugar phosphotransferase system	1.6E-3<br>3.2E-3
**Chordata**	• feeding behavior<br>• G-protein coupled receptor protein signaling pathway	4.3E-5<br>7.0E-4	• proteolysis	2.1E-2
**Urochordata & Vertebrata**	• antigen processing and presentation<br>• protein amino acid phosphorylation	5.5E-3<br>1.8E-2	• folic acid and derivative metabolic process<br>• group transfer coenzyme metabolic process	2.3E-3<br>5.8E-3
**Vertebrata**	• immune response<br>• cell surface receptor linked signal transduction	4.4E-11<br>2.0E-6	• DNA topological change	2.0E-3
**Tetrapoda**	• regulation of growth<br>• synaptic transmission	1.3E-2<br>2.0E-2	• valyl-tRNA aminoacylation<br>• response to water	4.3E-3<br>8.6E-3
**Amniota**	• immune response<br>• defense response	1.8E-3<br>2.0E-3	• regulation of transcription, DNA-dependent<br>• protein secretion<br>• riboflavin biosynthetic process *<br>• thiamin biosynthetic process *	9.2E-8<br>5.1E-5<br>1.0E-3<br>1.8E-3
**Mammalia**	• hemopoiesis<br>• reciprocal meiotic recombination	2.8E-3<br>8.3E-3	• aromatic amino acid family biosynthetic process<br>• chorismate metabolic process	1.1E-2<br>1.3E-2

2. Calculation method: Parent-Child-Union, multiple testing correction (MTC): Bonferroni

	Enriched gained GO terms	P-value (adjusted)	Enriched lost GO terms	P-value (adjusted)
**Unikonta**				
**Opisthokonta**				
**Metazoa & Choanoflagellata**	• cell communication<br>• biological regulation	1.3E-3<br>8.3E-2	• carbohydrate metabolic process<br>• polysaccharide metabolic process	3.5E-4<br>3.0E-2
**Metazoa**	• regulation of cellular process<br>• regulation of nitrogen compound metabolic process	6.4E-15<br>6.5E-6	• organic acid biosynthetic process	9.1E-2
**Bilaterian & Cnidaria**	• regulation of biosynthetic process	3.6E-2		
**Bilateria**	• electron transport chain<br>• phosphorus metabolic process<br>• regulation of cellular process *	2.0E-2<br>3.4E-2<br>4.1E-2		
**Deuterostomia**			• organic acid metabolic process<br>• cellular amino acid and derivative metabolic process	4.7E-3<br>2.7E-2
**Chordata**				
**Urochordata & Vertebrata**		5.5E-3<br>1.8E-2	• carbohydrate metabolic process	3.5E-2
**Vertebrata**	• immune system process<br>• regulation of cellular process	3.8E-8<br>4.9E-8	• carbohydrate metabolic process	3.3E-2
**Tetrapoda**				
**Amniota**	• response to stimulus<br>• immune system process	1.9E-2<br>8.3E-2	• regulation of macromolecule metabolic process<br>• regulation of gene expression	2.9E-3<br>1.4E-2
**Mammalia**				

3. Calculation method: Parent-Child-Intersection, multiple testing correction (MTC): Bonferroni

	Enriched gained GO terms	P-value<br><br>(adjusted)	Enriched lost GO terms	P-value<br><br>(adjusted)
**Unikonta**				
**Opisthokonta**				
**Metazoa & Choanoflagellata**	• cell communication<br>• biological regulation	1.0E-3<br>6.3E-2	• carbohydrate metabolic process	2.6E-4
**Metazoa**	• biological regulation	4.3E-13		
**Bilaterian & Cnidaria**				
**Bilateria**	• phosphorus metabolic process<br>• biological regulation	2.4E-2<br>3.4E-2		
**Deuterostomia**			• organic acid metabolic process<br>• cellular amino acid and derivative metabolic process	3.3E-3<br>8.0E-3
**Chordata**				
**Urochordata & Vertebrata**		5.5E-3<br><br>1.8E-2	• cellular nitrogen compound metabolic process<br>• carbohydrate metabolic process	2.2E-2<br><br>2.7E-2
**Vertebrata**	• immune system process<br>• biological regulation	2.2E-8<br>1.7E-7	• carbohydrate metabolic process	2.6E-2
**Tetrapoda**				
**Amniota**	• response to stimulus<br>• immune system process	1.6E-2<br>7.2E-2		
**Mammalia**				

4. Calculation method: Term-For-Term, multiple testing correction (MTC): Bonferroni

	Enriched gained GO terms	P-value (adjusted)	Enriched lost GO terms	P-value (adjusted)
**Unikonta**				
**Opisthokonta**				
**Metazoa & Choanoflagellata**	• cell communication<br>• signal transduction	1.0E-3<br>3.4E-2	• carbohydrate metabolic process<br>• xylan metabolic process	1.5E-4<br>7.6E-3
**Metazoa**	• regulation of cellular process<br>• regulation of biological process<br>• regulation of RNA metabolic process *	5.2E-11<br>3.0E-10<br>5.1E-5	• aromatic amino acid family biosynthetic process, prephenate pathway	2.6E-2
**Bilaterian & Cnidaria**				
**Bilateria**	• mitochondrial electron transport, NADH to ubiquinone<br>• ATP synthesis coupled electron transport	3.7E-3<br>6.1E-2	• organic acid biosynthetic process	5.1E-2
**Deuterostomia**			• organic acid metabolic process<br>• cellular amino acid and derivative metabolic process	4.8E-3<br>9.9E-3
**Chordata**	• feeding behavior<br>• digestion	9.1E-3<br>2.7E-2		
**Urochordata & Vertebrata**		5.5E-3<br>1.8E-2	• carbohydrate metabolic process	3.5E-2
**Vertebrata**	• immune response<br>• biological regulation	2.2E-8<br>9.0E-6	• carbohydrate metabolic process	1.3E-2
**Tetrapoda**				
**Amniota**	• response to stimulus<br>• immune response	4.6E-2<br>9.1E-2	• regulation of RNA metabolic process<br>• transcription, DNA-dependent	4.9E-5<br>2.5E-4
**Mammalia**	• system development	2.7E-2		

5. Calculation method: Term-For-Term, multiple testing correction (MTC): Westfall-Young-Single-Step

	Enriched gained GO terms	P-value (adjusted)	Enriched lost GO terms	P-value (adjusted)
**Unikonta**				
**Opisthokonta**				
**Metazoa & Choanoflagellata**	• cell communication<br>• signal transduction	2.0E-3<br>4.2E-2	• carbohydrate metabolic process<br>• xylan metabolic process	0<br>6.0E-3
**Metazoa**	• biological regulation<br>• transcription<br>• signal transduction *	0<br>3.8E-2<br>4.6E-2	• aromatic amino acid family biosynthetic process, prephenate pathway	6.4E-2
**Bilaterian & Cnidaria**	• programmed cell death	7.6E-2		
**Bilateria**	• mitochondrial electron transport, NADH to ubiquinone<br>• ATP synthesis coupled electron transport	2.0E-3<br>3.2E-2	• organic acid biosynthetic process	6.8E-2
**Deuterostomia**			• organic acid metabolic process<br>• cellular amino acid and derivative metabolic process	4.0E-3<br>8.0E-3
**Chordata**	• feeding behavior<br>• digestion	2.0E-2<br>5.8E-2		
**Urochordata & Vertebrata**				
**Vertebrata**	• biological regulation<br>• cell surface receptor linked signal transduction<br>• antigen processing and presentation	0<br>2.0E-3<br>6.0E-3	• carbohydrate metabolic process	2.0E-2
**Tetrapoda**				
**Amniota**			• regulation of RNA metabolic process<br>• regulation of macromolecule metabolic process	0<br>2.0E-3
**Mammalia**	• system development	4.4E-2		

6. Calculation method: Term-For-Term, multiple testing correction (MTC): Westfall-Young-Step-Down

	Enriched gained GO terms	P-value (adjusted)	Enriched lost GO terms	P-value (adjusted)
**Unikonta**				
**Opisthokonta**				
**Metazoa & Choanoflagellata**	• cell communication<br>• signal transduction	0<br>3.6E-2	• carbohydrate metabolic process<br>• xylan metabolic process	1.0E-3<br>8.0E-3
**Metazoa**	• regulation of cellular process<br>• transcription<br>• signal transduction *	0<br>3.9E-2<br>5.4E-2	• aromatic amino acid family biosynthetic process, prephenate pathway	4.8E-2
**Bilaterian & Cnidaria**	• programmed cell death	7.1E-2		
**Bilateria**	• mitochondrial electron transport, NADH to ubiquinone<br>• ATP synthesis coupled electron transport	1.0E-3<br>2.7E-2	• organic acid metabolic process<br>• organic acid biosynthetic process	1.0E-3<br>5.0E-3
**Deuterostomia**			• organic acid metabolic process<br>• cellular amino acid and derivative metabolic process	4.8E-3<br>9.9E-3
**Chordata**	• feeding behavior<br>• digestion	2.8E-2<br>6.1E-2		
**Urochordata & Vertebrata**				
**Vertebrata**	• biological regulation<br>• multicellular organismal process<br>• antigen processing and presentation *	1.0E-3<br>5.0E-3<br>9.0E-3	• carbohydrate metabolic process	3.2E-2
**Tetrapoda**				
**Amniota**	• response to stimulus	9.9E-2	• regulation of RNA metabolic process<br>• RNA biosynthetic process	0<br>1.0E-3
**Mammalia**	• system development	5.5E-2		
